# A novel post-developmental role of the Hox genes underlies normal adult behavior

**DOI:** 10.1073/pnas.2209531119

**Published:** 2022-12-01

**Authors:** A. Raouf Issa, Jonathan A. C. Menzies, Aishwarya Padmanabhan, Claudio R. Alonso

**Affiliations:** ^a^Sussex Neuroscience, School of Life Sciences, University of Sussex, Brighton BN1 9QG, UK

**Keywords:** Drosophila, Hox genes, neuron, post-mitotic, flight

## Abstract

The molecular mechanisms underlying the stability of mature neurons and neural circuits are poorly understood. Here we explore this problem and discover that the *Hox* genes are a component of the genetic program that maintains normal neural function in adult *Drosophila*. We show that post-developmental downregulation of the *Hox* gene *Ultrabithorax* (*Ubx*) in adult neurons leads to substantial anomalies in flight. Mapping the cellular basis of these effects reveals that *Ubx* is required within a subset of dopaminergic neurons, and cell circuitry analyses in combination with optogenetics allow us to link these dopaminergic neurons to flight control. Functional imaging experiments show that *Ubx* is necessary for normal dopaminergic activity, and neuron-specific RNA-sequencing defines two previously uncharacterized ion channel-encoding genes as potential mediators of *Ubx* behavioral roles. Our study thus reveals a novel role of the *Hox* system in controlling adult behavior and neural function. Based on the broad evolutionary conservation of the Hox system across distantly related animal phyla, we predict that the Hox genes might play neurophysiological roles in adult forms of other species, including humans.

## Significance

At the end of development, cells must deactivate the genetic programs that guided their differentiation and switch on the molecular code script that ensures cellular stability and physiology. Within the nervous system, while there is a substantial understanding of how genes influence neuro-developmental processes, the genetic programs underlying cell function and stability in adult post-mitotic neurons remain largely unknown. Here we investigate this problem in *Drosophila* and discover that the *Hox* genes—which encode a family of evolutionarily-conserved developmental regulators, key for axial patterning—are essential for the normal physiology of post-mitotic neurons and adult behavior. Based on the evolutionary conservation of the *Hox* genes, we suggest that they may also play key neurophysiological roles in the adult forms of other species, including humans.

At the end of the developmental process, individual neurons must deactivate the genetic programs that guided patterning and differentiation, and switch on molecular programs that ensure neural maintenance and physiology. While considerable efforts have been invested into the decoding of neural developmental programs, less is known about the identity and influence of genetic systems in the post-mitotic neuronal state of the adult organism (1, 2).

**Fig. 1.**
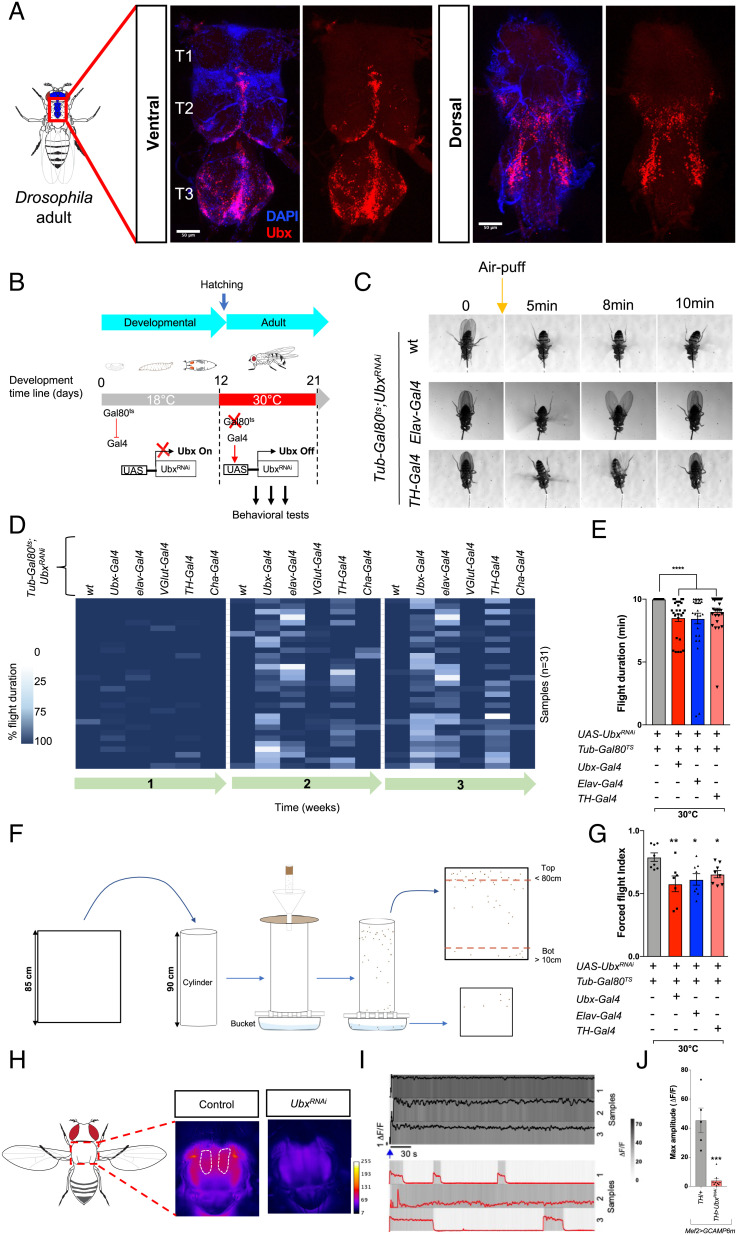
*Ubx* is necessary for normal flight maintenance in adult *Drosophila*. (*A*) Confocal images of the whole VNC of adult *Drosophila* showing expression of Ubx protein in specific regions of the adult VNC (T1-T3, indicate the respective thoracic segments). (*B*) Strategy for conditional neuronal downregulation of *Ubx* in the adult. Flies expressing *Ubx^RNAi^* under temperature-sensitive Gal80ts repression, were exposed to elevated temperatures (30°C) upon hatching to allow active downregulation of *Ubx*. At permissive temperature (18°C) Gal80ts protein is functional and represses expression of *Ubx^RNAi^* (‘Ubx ON’); conversely, expression of *Ubx^RNAi^* is activated in cells at temperatures above 30°C (‘Ubx OFF’). Behavioral experiments were performed on adult males and females in Ubx ON/OFF modes. (*C*–*E*) Evaluation of flight performance on tethered *Drosophila*. (*C*) Representative flight duration is indicated by video snapshots of tethered WT, pan-neuronal (*Tub-Gal80ts*;*Elav-Gal4*, *Ubx^RNAi^*), and dopaminergic (*Tub-Gal80ts*;*TH-Gal4*, *Ubx^RNAi^*) *Ubx^RNAi^* expressing flies, at 30°C. The orange arrow indicates the induction stimulus (air-puff), and WT flies are able to fly above 10 min compared to *Ubx^RNAi^* expressing flies. (*D* and *E*) Flight duration represented as a heat map, and as averages of flight duration at week 2, for flies expressing *Ubx^RNAi^* in different nerve cells (*Ubx/elav/Vglut/TH/Cha>Ubx^RNAi^*) compared to controls (*UAS-Ubx^RNA^*^i^) under Gal80ts repression (week 1 = 6–7 d; week 2 = 8–10 d; week 3 = 14–15 d; n > 20). (*F*) Cartoon illustrating the forced flight test procedure (for details, see *Materials and Methods*). Flies are shown as brown dots on the cylinder and membrane. (*G*) Average forced flight index at week 2 of flies expressing *Tub-Gal80ts*; *Ubx^RNAi^* in different neuronal populations and reared at 30°C compared to controls (*Tub-Gal80ts*; *Ubx^RNAi^*) (Each data point represents a group of n > 12 flies). (*H*) Representative raw images of GCaMP fluorescence (warmer color) in flight muscles. (*I*) Representative calcium signal traces in dorsal longitudinal flight muscle (Dlm) from control (*Mef2^LexA^,GCaMP6m^LexAOP^/Tub-Gal80^ts^;*TH-Gal4/FLP) and experimental (*Mef2^LexA^,GCaMP6m^LexAOP^/Tub-Gal80^ts^;* TH-Gal4/Ubx^RNAi^) flies. (*J*) Normalized mean of GCaMP signals maximum during sustained flight (n = 5–10). All error bars represent SEM. Significant values in all figures based on Mann–Whitney U test or one-way ANOVA with the post hoc Tukey–Kramer test: ^∗^*P* < 0.05, ^∗∗^*P* < 0.01, ^∗∗∗^*P* < 0.001, and ^∗∗∗∗^*P* < 0.0001.

Recent observations in our laboratory suggest that overexpression of particular transcription factors in post-mitotic neurons impacts on neural physiology (3–6). Among these factors are the *Hox* genes, which encode a family of developmental regulators whose activities control cell differentiation programs in animals as diverse as insects and mammals (7–9). *Hox* gene expression patterns have been studied in great detail during embryogenesis (10, 11), but recent data from our laboratory and elsewhere (5, 12) show that *Hox* genes are also expressed in the adult *Drosophila* nervous system (Fig. 1*A*) suggesting the plausibility that these genes might perform functions after development has concluded. To explore this notion, we hypothesized that the *Hox* genes may contribute to the genetic program underlying brain stability and used the *Drosophila* adult as a system to explore this notion, seeking to establish the biological roles of adult post-mitotic neural *Hox* gene expression. Our observations reveal that post-mitotic *Hox* gene expression in *Drosophila* is essential for normal adult behavior through effects in a defined set of dopaminergic neurons in the ventral nerve cord.

## *Ubx* is Required in the Nervous System for Normal Flight Maintenance in Adult *Drosophila*

To determine the role of the *Hox* genes in the adult without affecting their previous developmental functions, we applied a conditional expression strategy that maintains normal *Hox* expression during development and reduces expression specifically after eclosion (Fig. 1*B*). For this, we used the Gal80/Gal4 system (13, 14) to drive tissue-specific RNA interference (*RNAi*) constructs (*SI Appendix*, Fig. S1 *A*–*G*) designed to reduce the expression of one of the posterior *Drosophila Hox* genes—*Ultrabithorax* (*Ubx)* (15)—exclusively in the adult. quantification of Ubx expression in normal and RNAi conditions demonstrates that RNA interference leads to a substantial reduction of Ubx expression (*SI Appendix*, Fig. S1 *A*–*E*). The analysis of the behavioral impact of these perturbations during the first 3 wk of adult life—while driving *Ubx^RNAi^* pan-neuronally (*elav-Gal4*) or within the *Ubx* expression domain (*Ubx-Gal4*)—results in no detectable changes in general fly locomotion, as assessed through standard climbing assays (*SI Appendix*, Fig. S2 *A*–*C*). This first result suggested that reduction of *Ubx* function in the adult may not impact the normal physiology of motor circuits underlying adult locomotion on substrate. In contrast, tethered flight experiments (16, 17) reveal that RNAi-mediated knockdown of *Ubx* expression does impair flight maintenance (Fig. 1 *C*–*E* and *SI Appendix*, Fig. S2 *D*–*F*, *J*, and *L* and Movie S1) in 2-wk-old flies: expression of *Ubx*/*elav*-driven *UAS-Ubx^RNAi^* leads to a marked deficit in the ability of flies to maintain flight following a single air-puff stimulus (NB: similar effects on flight were confirmed when using a different RNAi line (see below) targeting *Ubx* (*SI Appendix*, Fig. S1*H*)). Two other independent methods to assess flight performance: forced flight (17) (Fig. 1 *F* and *G* and *SI Appendix*, Fig. S2 *G*, *K*, and *M*) and takeoff tests (17, 18) (*SI Appendix*, Fig. S2 *I* and Movies S10 and S11), provide further confirmation that normal expression of *Ubx* is required for normal flight in adult flies, revealing a novel role of the *Hox* system in the control of behavior in the fully formed organism. Experiments conducted with an independent RNAi line (*Ubx^RNAi-2^*) further confirm these behavioral observations (*SI Appendix*, Fig. S1*I*). Furthermore, cell-specific conditional Ubx knockout achieved via genome edition through CRISPR-Cas9 (*Ubx^gRNA^*) (*SI Appendix*, Fig. S3 *A*–*G*) leads to behavioral effects similar to those observed after RNAi treatments (*SI Appendix*, Fig. S3 *H*–*K*), providing compelling evidence about the links between reduction of Ubx expression in the adult nervous system and a flight maintenance phenotype.

## Modulation of Activity in Ubx-Expressing (*Ubx*^+^) Dopaminergic Neurons Affects Flight

*Ubx* is expressed across extensive domains on its dorsal and ventral aspects of the adult ventral nerve cord (VNC)—the neural equivalent of the mammalian spinal cord (19) (Fig. 1*A*, and previous observations (5, 12)). To investigate the cellular basis of Ubx effects on adult flight, we first mapped the expression of Ubx within the main neuronal types in the adult nervous system and, then, systematically depleted Ubx from these neurons and determined the behavioral impact of these perturbations.

**Fig. 2.**
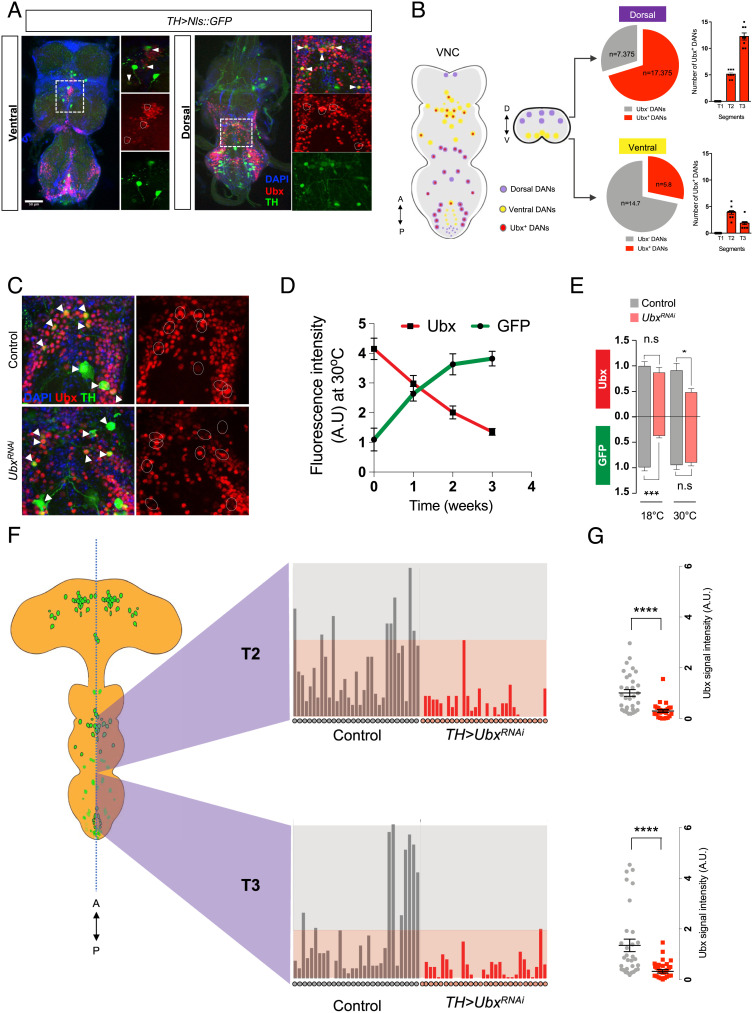
Ubx is expressed in adult VNC Dopaminergic (TH) neurons. (*A*) Confocal images of the VNC of adult *Drosophila* showing expression of Ubx in TH neurons. TH neurons are labeled by GFP driven by *TH-Gal4*. (*B*) Ubx positive TH neurons in the VNC. At the *Left*, a cartoon showing the presence of Ubx positive TH neurons (red dot circles) in both ventral (yellow) and dorsal (purple) sides of VNC. Ubx is expressed in ~29% (5 ± 0.9) (ventral) and 70% (17 ± 0.4) (dorsal) of dopamine neurons in the VNC (n = 8–10) (*Right*). (*C*–*E*) Conditional reduction of Ubx expression by *Ubx^RNAi^* in TH neurons. (*C*) Confocal imaging of a region of the adult VNC with high Ubx protein expression, showing the effects of *Ubx^RNAi^* treatment at 30°C on Ubx expression. Genotypes: control (*TH-Gal4; UAS-Nls::GFP, UAS-FLP*); *Ubx^RNAi^* (*TH-Gal4; UAS-Nls::GFP,Tub-Gal80ts; UAS-Ubx^RNAi^*). Note the apparent decrease in red signal within TH neurons (white circles) (flies were 8–10 d old, n = 5–6). (*D*) Kinetics of Ubx and GFP expression in adult flies expressing *Ubx^RNAi^* in TH neurons (*TH-Gal4; UAS-NLS::GFP,Tub-Gal80ts; UAS-Ubx^RNAi^*) in non-permissive conditions (30°C) over time (1–3 wk). Note that as time elapses the reduction in Ubx expression becomes more pronounced, and, in contrast, expression of GFP becomes stronger. (*E*) Quantification of Ubx expression in normal and *Ubx^RNAi^* conditions at permissive (18°C) and non-permissive (30°C) temperatures for Gal80ts repression (at week 2). Note that, at low temperature, Ubx levels are not different between control and *Ubx^RNAi^* lines; in contrast, GFP expression is highly reduced due to Gal80ts repression. At high temperatures, Ubx expression is significantly downregulated. (*F*) Quantification of Ubx in individual nuclei in the adult VNC. (*Left*) Diagram of the adult VNC (anterior to the top) illustrating the approximate positions of TH neurons (green). (*Middle*) Quantification of Ubx expression in individual nuclei in T2 (*Top*) and T3 (*Bottom*) of control (grey) and TH>UbxRNAi (red) dopaminergic neurons, showing a clear reduction of Ubx expression in the population of TH nuclei expressing the UbxRNAi construct. (*G*) Expression levels of Ubx protein in individual TH neurons with normal (grey) or down-regulated (red) Ubx expression in T2 (top) and T3 (*Bottom*) regions of the adult VNC demonstrating a significant reduction in Ubx expression under UbxRNAi treatment in both segments. Error bars represent SEM. Significant values in all figures based on Mann–Whitney *U* test: ^∗^*P* < 0.05, ^∗∗∗^*P* < 0.001, and ^∗∗∗∗^*P* < 0.0001.

Expression analyses show that *Ubx* is detected within the cholinergic (*Cha-Gal4*), glutamatergic (*VGlut-Gal4*), and dopaminergic (*TH-Gal4*) adult systems (Fig. 2*A* and *SI Appendix*, Fig. S2 *N *and *O*). Downregulation experiments—induced by neuron-specific RNAi expression conditionally activated at eclosion—show that reduction of *Ubx* within the dopaminergic (TH) domain is sufficient to phenocopy the effects observed within the pan-neural domain (*elav-Gal4*) (Fig. 1 *C*–*G* and *SI Appendix*, Fig. S2 *D*–*M*); in contrast, no significant effects are detected after *Ubx* reduction in either glutamatergic or cholinergic neurons (Fig. 1*D* and *SI Appendix*, Fig. S2 *D*–*M*). These observations suggest that dopaminergic neurons (here on termed TH neurons) are sensitive to reductions in *Ubx* input, indicating that normal *Ubx* expression is required for the normal function of these neurons for normal flight. Furthermore, fluorescence imaging of flight muscle (17, 20) expressing a genetically encoded calcium reporter (GCaMP6m) (Fig. 1 *H*–*J* and Movies S2 and S3) shows that *TH*>*Ubx^RNAi^* leads to a significant reduction in flight muscle activity. Altogether, these data indicate that normal expression of *Ubx* in dopaminergic neurons is required for normal flight maintenance in adult flies.

To gain further insight into how Ubx might affect the biology of adult dopaminergic neurons, we labeled the TH domain using nuclear GFP (NLS::GFP) under a dopaminergic driver (*TH-Gal4*) (Fig. 2*A*) and conducted a detailed survey of *Ubx* expression within the TH territory. The results of this experiment show that Ubx protein was expressed in approximately ~30% (5 ± 0.9) of dorsal, and 60% (17 ± 0.5) of ventral adult dopaminergic neurons within the VNC region (Fig. 2*B* and Movie S12). Furthermore, dopaminergic-specific conditional reduction of *Ubx* expression triggered at eclosion time (*TH-Gal4; tub-Gal80^ts^; UAS-Ubx^RNAi^*) resulted in ~50% downregulation of *Ubx* expression within the TH domain after 2 wk of treatment (Fig. 2 *C*–*G*). These expression and functional analyses confirm that Ubx is normally expressed in adult TH neurons and that post-developmental reduction of *Ubx* expression leads to a substantial reduction in protein formed within the dopaminergic system.

## Normal Neural Activity Levels of Ubx^+^ Dopaminergic Neurons Are Necessary for Flight

We then examined the relation between the activity of the dopaminergic system and flight control. For this, we artificially increased or reduced neural activity in TH neurons using optogenetics (21) and examined the impact of these treatments on flight maintenance.

**Fig. 3.**
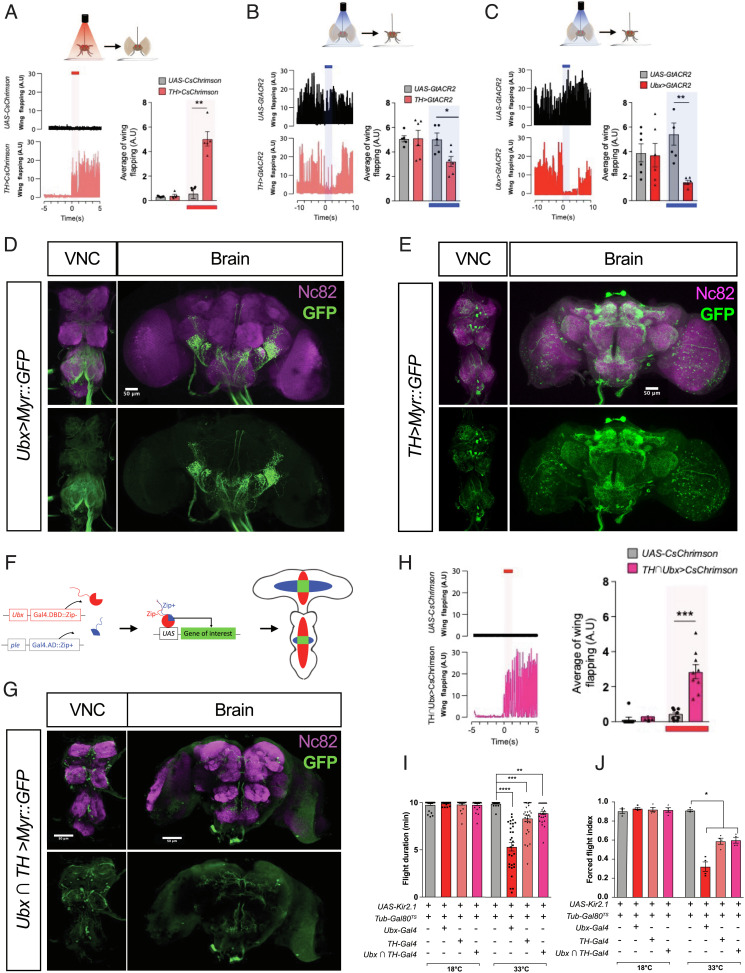
Modulation of neural activity of Ubx^+^ TH neurons affects flight. (*A*) Optogenetic activation (red shade) of TH neurons expressing channelrhodopsin CsChrimson (*TH*>*CsChrimson*) induces spontaneous flight (*Top*: Cartoon representation of experimental results) (*B* and *C*). Optogenetic inhibition (blue shade) of TH neurons (*TH>GtACR2*) (b) and Ubx^+^ cells (*Ubx*>*GtACR2*) (c) by expressing GtACR2 reduces flight. In each figure, *Left* panels represent wing flapping frequency, and *Right* panels depict a histogram of average frequencies. Control flies are *UAS-CsChrimson* and *UAS-GtACR* (n = 6). (*D *and *E*) Confocal images showing TH (d) and Ubx cells (e) in VNC and brain. (*F*) Schematic illustration of the Split-Gal4 system based on the complementation between the two functional domains of Gal4, the DNA-binding (DBD) and transcription-activation (AD) domains. Each domain is fused to a heterodimerizing leucine zipper (Zip^+^ or Zip^−^) that promotes the fusion of the two domains when expressed in the same cell reconstituting transcriptional activity. This technique was used to generate Ubx*∩*TH-Gal4 lines. (*G*) Confocal images of the fly VNC (ventral side) and brain (anterior side) showing the UAS-Myr::GFP expression pattern driven by *Ubx^Gal4.DBD^ ∩ ple^Gal4.AD^* (Ubx*∩*TH-Gal4) defines a subset of TH neurons that express Ubx. (*H*) Flight patterns of flies before and after optogenetic activation of Ubx^+^ TH cells, (n = 8–9). (*I* and *J*) Flies with Ubx positive neurons inhibited by expression of the potassium channel encoded by the *Homo sapiens* KCNJ2 gene (Kir2.1) show reduced flight duration (*I*) and forced flight index (*J*); a reduction is also observed when Kir2.1 is expressed in the TH-Gal4 domain, and, notably, in the Ubx*∩*TH intersectional domain. See also Movie S4 (related to Fig. 3*A*), Movie S5 (related to Fig. 3*B*), Movie S6 (related to Fig. 3*C*), and Movie S7 (related to Fig. 3*H*). Error bars represent SEM. Significant values in all figures based on Mann–Whitney *U* test or one-way ANOVA with the post hoc Tukey–Kramer test: ^∗^*P* < 0.05, ^∗∗^*P* < 0.01, and ^∗∗∗^*P* < 0.001.

We, firstly, activated dopaminergic neurons through expression of CsChrimson (21) and monitored effects on flight, observing that optogenetic activation of TH neurons (N.B. in the absence of an air puff) is sufficient to initiate flight (Fig. 3*A* and Movie S4). Secondly, we optogenetically inhibited TH neurons using GtACR (22) and observed that this is sufficient to significantly reduce (yet not prevent) wing flapping (Fig. 3*B* and Movie S5). These results demonstrate that the modulation of neural activity of TH neurons affects flight. Similar flight suppression effects were also observed upon optogenetic treatment of flies expressing GtACR within the Ubx domain (Fig. 3*C* and Movie S6) (NB: expression of CsChrimson within the Ubx domain leads to lethality). These observations demonstrate a functional link between the activity of the dopaminergic system and flight maintenance.

Reporter expression analyses using Ubx-Gal4 show that some TH neurons do not seem to activate Ubx transcription: the Ubx domain is exclusively located in the VNC with projections into the brain, while TH neurons are present in both VNC and brain; these experiments also show that Ubx is expressed in other neural cell types and not only in TH neurons (Fig. 3 *D* and *E* and *SI Appendix*, Fig. S2 *N *and *O* and Movies S13 and S14). Therefore, it was of great interest to us to establish the roles of those particular TH neurons that express Ubx (termed *Ubx^+^* TH neurons) in connection to flight control. For this, we developed a split-Gal4 approach (23) expressing complementary forms of the Gal4 transcriptional activator, one bearing the DBD and the other encoding the activation domain (AD) from two distinct promoters: *Ubx* (Gal4.DBD::Zip^-^) and *pale* (*ple*, the gene that encodes TH) (Gal4.AD::Zip^+^) with the view of reconstituting functional Gal4 protein only at the intersection between the *Ubx* and *TH* transcriptional domains (Fig. 3 *F* and *G* and *SI Appendix*, Fig. S4*A*). This new genetic tool enabled us to develop a range of experiments to test the functional roles of *Ubx^+^* TH neurons (Movie S15) in relation to flight control. First, optogenetic activation of TH∩Ubx (split-Gal4, “split”) neurons is sufficient to trigger flight in the absence of an air puff (Fig. 3*H* and Movie S7). Second, FlpOut-induced expression of red-activatable channelrhodopsin (ReaChR) only within the Ubx^+^ TH domain (via *TH>LexA;LexAOP-FLP* and under *Ubx-Gal4* induction) (*SI Appendix*, Fig. S4*B*) triggers flight under red-light illumination. This further demonstrates that the activity of Ubx^+^ dopaminergic neurons is linked to flight control (*SI Appendix*, Fig. S4 *C* and *D*). Third, thermogenetic inhibition of neural activity mediated through expression of the inward-rectifier potassium ion channel Kir2.1/KCNJ2 (24) leads to a significant reduction in flight duration and forced flight index when applied to TH∩Ubx neurons (Fig. 3 *I* and *J*). In sum, these experiments indicate that activity levels of Ubx^+^ TH neurons are directly related to flight regulation: inhibition of these neurons halts flight, while their stimulation, triggers flight.

## *Ubx* Affects the Activity of Dopaminergic Neurons

**Fig. 4.**
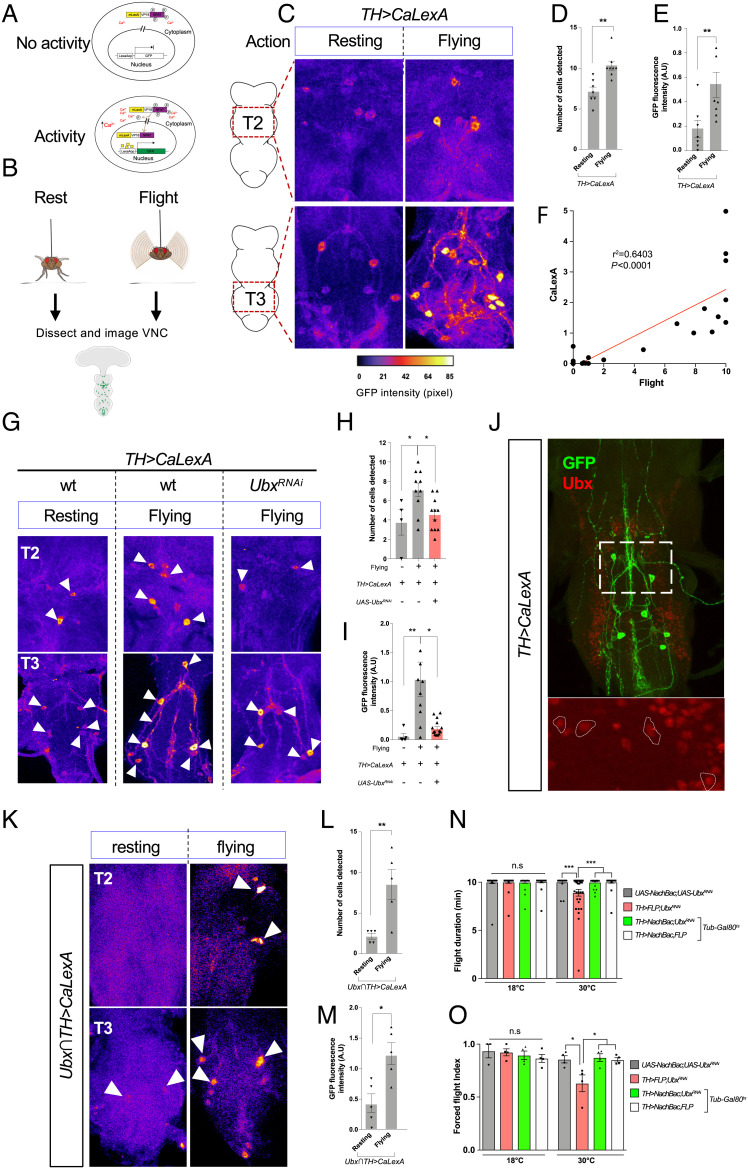
Levels of neural activity of *Ubx^+^* TH neurons during flight. (*A*) Schematic illustration of the CaLexA system, which records neuronal activity based on Ca^2+^-NFAT interaction. Cartoon representation of the CaLexA system in the absence (*Top* panel) and presence of neural activity (*Bottom* panel); in the latter, Ca^2+^ accumulation dephosphorylates NFAT, triggering the transport of the transcription factor mLexA-VP16-NFAT into the nucleus, where the chimeric transcription factor LexA binds to the LexA operator (LexAop), and subsequently induces expression of the GFP reporter gene. (*B*) Experimental protocol. Resting and flying *Drosophila* were collected for dissection and their VNCs were immunostained and imaged. (*C*) Representative images of TH neurons in the VNCs of resting and flying wild-type *Drosophila* (TH>CaLexA), immunolabelled with GFP (warmer color). Note that resting flies show low GFP intensity/CaLexA signals while flying flies show very high signal intensity. (*D* and *E*) Quantification of GFP positive TH neurons (*D*) and GFP intensity (*E*) of the VNC thoracic segments 2 (T2) and 3 (T3) in *TH*>*CaLexA* flies in different conditions (resting: n = 7; flying: n = 8). (*F*) Increase in GFP intensity is correlated with flight (n = 21). (*G*) Representative images of TH neurons in the VNCs of control WT *Drosophila* (*TH>CaLexA*) and TH neurons expressing *Ubx^RNAi^* (*TH>CaLexA; Ubx^RNAi^*). (*H* and *I*) Quantification of GFP positive TH neurons (*H*) and GFP intensity (*I*) of VNC in segments T2 and T3 in *TH*>*CaLexA* and *TH*>*CaLexA; Ubx^RNAi^* (n > 5). (*J*) GFP-positive TH neurons are also Ubx positive. (*K*) Representative images of the Ubx*∩*TH intersectional domain in the VNC of resting and flying *Drosophila* (*Ubx∩TH*>*CaLexA*). (*L* and *M*) Quantification of GFP positive cells (*L*) and GFP intensity (*M*) of VNC in T2 and T3 segments in *Ubx∩TH*>*CaLexA* (n = 5). (*N* and *O*) Increase in TH neurons activity generated by conditionally expressing the voltage-gated bacterial Na^+^ channel (NaChBac) rescues the flight deficit resulting from Ubx downregulation. Averages of flight duration (*N*) and forced flight index (*O*) of flies after heat shock (at 30°C). Error bars in figures represent SEM. Significant values in all figures based on Mann–Whitney *U* test or one-way ANOVA with the post hoc Tukey–Kramer test: ^∗^*P* < 0.05, ^∗∗^*P* < 0.01, ^∗∗∗^*P* < 0.001.

Building on our neuronal activity manipulations, and given that *Ubx* reduction within TH-neurons does not induce a visible change in the number and size of TH neurons (*SI Appendix*, Fig. S5 *A*–*C*), we considered the model that *Ubx* might be affecting the function rather than the integrity of dopaminergic neurons. To test this idea, we first sought to determine the natural patterns of activity of TH neurons in relation to normal flight, and how these were affected when *Ubx* expression was reduced, exploring the possibility that changes in *Hox* expression in the adult, might affect neural physiology. For this, we made use of the CaLexA system (calcium-dependent nuclear import of LexA) (Fig. 4*A*) (25), which acts as a molecular recorder device of neural activity in the tissue/cellular context of choice providing a platform to correlate neuronal activity patterns with specific behaviors (26). In a first series of experiments, we drove the CaLexA reporter from the *TH-Gal4* driver and observed that after 30 min of constant flight there is a significant increase in CaLexA signal in the dopaminergic system when compared to a resting condition (Fig. 4 *B*–*E*). Indeed, CaLexA signal intensity within the TH domain is positively correlated with flight duration (Fig. 4*F*), indicating that the dopaminergic system is active during flight. In a second series of experiments, we reduced *Ubx* expression (using *Ubx^RNAi^*) within TH neurons and observed a significant decrease in neural activity in both the second (T2) and third (T3) thoracic segments (Fig. 4 *G*–*I*) (NB: although these experiments were conducted across the whole TH domain, expression of *Ubx* could have only been reduced in those cells that normally express the gene). These data strongly support the notion that a reduction of *Ubx* expression in dopaminergic neurons leads to a decrease in neural activity within this domain.

Interestingly, although we can indeed detect dopaminergic neurons in the T1 segment, as well as in the brain (Fig. 3*E*), we do not observe any CaLexA signal after flight in these regions (*SI Appendix*, Fig. S5*D*). Further results indicate that flight-related dopaminergic activity takes place primarily within the VNC in segments T2–T3 (Fig. 4*G*), the thoracic regions where neural expression of *Ubx* is prominent (5, 12). Expression of the CaLexA reporter within the intersectional domain TH∩Ubx reveals that even within this restricted aspect of the dopaminergic system, there are significant differences in CaLexA signal detected after flight, when compared with a resting control (Fig. 4 *K*–*M*). Furthermore, expression of the bacterial voltage-gated sodium channel NaChBac (27, 28) within dopaminergic neurons expressing reduced levels of *Ubx*, leads to a rescue of the flight phenotype observed after TH-driven *Ubx^RNAi^*, as assessed by two independent behavioral tests: tethered flight (Fig. 4*N* and *SI Appendix*, Fig. S5*E*) and forced flight (Fig. 4*O*); this latter observation strongly suggests that a reduction of Ubx affects the physiological properties of the neuron, rather than affecting their integrity. Altogether, the data above indicate that normal *Ubx* expression within the thoracic dopaminergic system is required for flight-related neural activity in TH neurons.

## Dopaminergic Neurons Modulate Flight through Direct Interaction with Flight Motoneurons

**Fig. 5.**
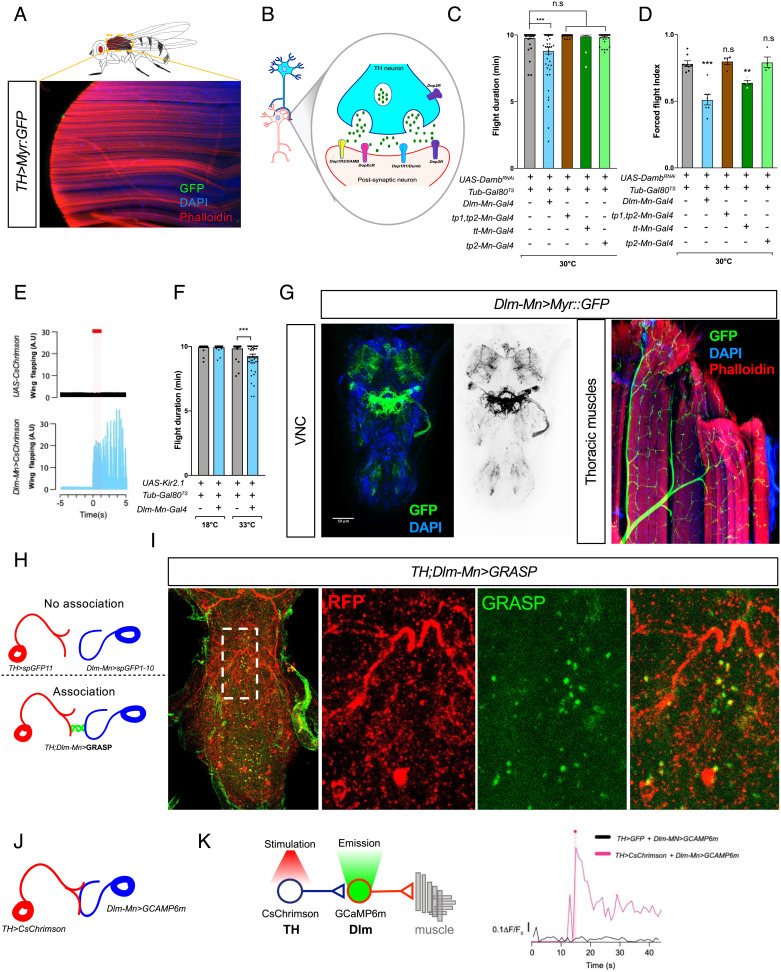
TH neurons modulate flight through direct contact with flight motoneurons. (*A*) Confocal image of thoracic muscles of fly expressing myristoylated GFP in dopaminergic neurons (*TH>Myr:GFP*). No GFP signal can be observed. (*B*) Schematics of a dopaminergic synapse and receptors. (*C* and *D*) Average flight duration (*C*) and force flight (*D*) at 30°C of flies with downregulated expression of dopamine receptors (*DAMB^RNAi^*) in specific flight motor neurons. (*E*) Red light-evoked (red shade) activation of  Dlm motoneuron expressing CsChrimson (*Dlm-Mn>CsChrimson*) induces spontaneous flight (n ≥ 15). See also Movie S8. (*F*) Flies with Dlm neurons inhibited by expression of Kir2.1 show reduced flight duration. (*G*) Representative confocal images showing the projections of Dlm motoneurons within the VNC (*Left* panel) and longitudinal flight muscles (*Right* panel). (*H*) Cartoon illustrating the principle underlying GFP Reconstitution Across Synaptic Partners (GRASP). GFP is reconstituted when two complementary segments of GFP associate on the extracellular surfaces of adjacent neurons. (*I*) Confocal image of GRASP reconstitution in the VNC of *Drosophila TH;Dlm-Mn>GRASP* (*TH-LexA>LexAOP:RFP,LexAOP:spGFP11 & Dlm-Mn-Gal4>UAS:spGFP1-10*). GRASP fluorescence reveals structural links between TH and Dlm neurons in the VNC. (*J*) Cartoon illustrating the experimental approach to determine a functional connection between TH and Dlm neurons. (*K*) Red light-evoked (red shade) activation of TH neuron expressing CsChrimson (*TH>CsChrimson*) induces spontaneous activity of Dlm motoneurons in *Drosophila* TH;Dlm-Mn>CsChrimson;GCAMP6 (*TH-LexA,LexAOP:CsChrimson;Dlm-Mn-Gal4,UAS:GCAMP6m*) compared to controls. See also Movie S9. Error bars represent SEM. Significant values in all figures based on Mann–Whitney *U* test or one-way ANOVA with the post hoc Tukey–Kramer test: ^*^P < 0.01, ^∗∗∗^*P* < 0.001.

We next probed the structural and functional relationships between the dopaminergic system and the flight motor system underlying normal flight. For this, we expressed membrane-bound GFP in all TH neurons, and traced the projections of these labeled TH neurons observing that they do not produce any direct contacts with the flight motor system (Fig. 5*A*) indicating that dopaminergic neurons must be communicating with the flight muscle system indirectly. To further explore this idea, we disabled the dopaminergic pathway specifically in those motor neurons that innervate the muscle field in the thorax (directly controlling wing flapping), and whose activity triggers flight (*SI Appendix*, Fig. S6*A*) (17) (hereon termed “flight-MNs”). To accomplish this, we reduced the expression of dopamine receptors (Fig. 5*B*) specifically in flight-MNs. These manipulations tested the effects of the four known dopamine receptor genes: Damb/Dop1R2, Dumb/Dop1R1, DopR2, and DopEcR (29, 30) and led us to discover that expression of *UAS-Damb^RNAi^* (known to reduce expression of the gene encoding Dop1R2/DAMB (30, 31)) under control of dorsal longitudinal muscle (*Dlm*) motor neuron driver, *Dlm-Mn-Gal4* (*VT021842.GAL4.attp2*) affects flight maintenance (Fig. 5 *C*–*E* and *SI Appendix*, Fig. S6 *B*–*D*) providing support to the notion that thoracic TH neurons are pre-synaptic to flight command Dlm motor neurons. In contrast, reduction of expression of this same dopamine receptor in other motor neurons involved in flight control, as driven by the *tp1-Mn-Gal4, tp2-Mn-Gal4, tt-Mn-Gal4*, *and tp2-Mn-Gal4* lines (Fig. 5*C* and *SI Appendix*, Fig. S6 *E* and *F*), or other dopamine receptors in flight motor neurons, do not significantly alter flight duration as assessed in tethered flight experiments (*SI Appendix*, Fig. S6 *B*–*D*). These observations suggest that TH neurons control the activity patterns of Dlm neurons and that these motor neurons are directly related to flight control. To investigate this further, we thermogenetically inhibited neural activity of Dlm neurons using Kir2.1/KCNJ2 (Fig. 5*F* and *SI Appendix*, Fig. S6*G*), or blocked synaptic transmission of these neurons by expressing a dominant-negative form of the dynamin GTPase, *shibire^TS^* (*SI Appendix*, Fig. S6 *H *and *I*) leading to a significant reduction in flight duration. This indicates that the Dlm motor neurons are directly involved in flight maintenance. Optogenetic activation of Dlm neurons using CsChrimson further confirms our model. (Fig. 5*E* and Movie S8). These data are consistent with previous observations on the links between the activity of Dlm neurons with the frequency and the number of wing beat cycles during courtship, and flight ability (tested by voluntary takeoff and “drop” assays) (17). Finally, membrane-tagged GFP expressed in Dlm neurons shows that these motor neurons innervate the longitudinal muscles in the fly thorax (Fig. 5*G*). These analyses strongly support the model of a direct neuronal link between the dopaminergic system and the flight muscles controlling flight.

To confirm structural connectivity between the TH neurons and Dlm neurons, we used the GRASP system (Fig. 5*H*) (32, 33) expressing complementary forms of GFP in both putative pre-synaptic (TH-Gal4) and post-synaptic (DLM-Mn-Gal4) elements, and observed reconstitution of functional GFP signal in both T2 and T3 segments (Fig. 5*I* and *SI Appendix*, Fig. S6 *J* and *K*) providing strong structural evidence of a direct link between the dopaminergic system, and the Dlm neurons that command flight. Furthermore, optogenetic stimulation of TH neurons leads to activation of Dlm motor neurons (Fig. 5 *J* and *K* and Movie S9) demonstrating a functional, physiological coupling between these neurons. Altogether, this comprehensive dataset provides a cellular framework to explain how Ubx-dependent reductions in the activity of the dopaminergic system relate to alterations in flight.

## *Solute carrier* (*SLC*) Symporter Genes Are Differentially Expressed in Response to Ubx Downregulation in TH Neurons

**Fig. 6.**
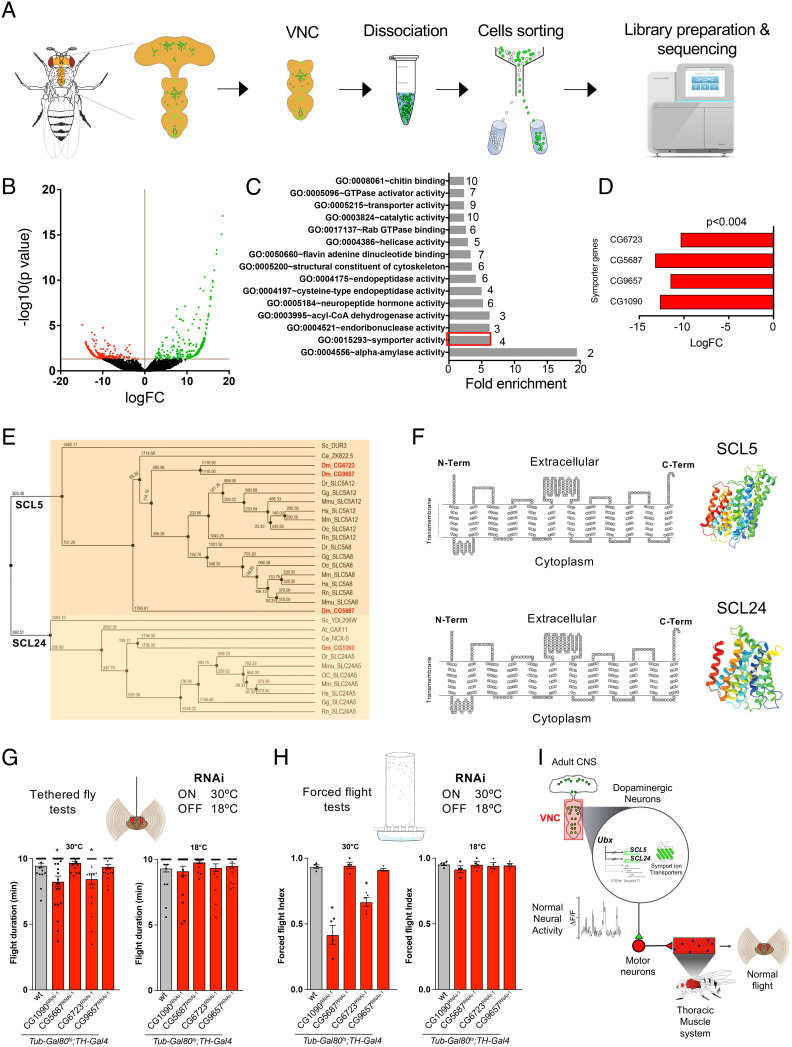
SLC gene symporter genes are differentially expressed in response to Ubx downregulation in TH neurons. (*A*) Experimental design of our neuron-specific transcriptomic experiment. (*B*) Volcano plot depicting differentially expressed genes (DEGs). Red and green dots represent down- and upregulated genes, respectively. (*C*) Gene Ontology (GO) enrichment analysis. GO molecular functions of DEGs show the top four genes (red bar) predicted to have symporter activity (involvement in calcium, potassium, sodium and/or solute co-transport). (*D*) The four differentially expressed symporter genes are strongly downregulated. None of these genes has been previously characterized in flies. (*E*) Dendrogram of the differentially expressed symporter genes detected in *Drosophila* showing their relation to other gene families across the animal kingdom. The analysis suggests that the differentially expressed symporter genes belong to the human SLC5 and SLC24 protein families. Abbreviations are: *Saccharomyces cerevisiae* (Sc), *Arabidopsis thaliana* (At), *Danio rerio* (Dr), *Gallus gallus* (Gg), *Homo sapiens* (Hs), *Macaca mulatta* (Mm), *Oryctolagus cuniculus* (Oc), *Rattus norvegicus* (Rn), *Mus musculus* (Mmu) *Drosophila melanogaster* (Dm), *Caenorhabditis elegans* (Ce) (values near/above internodes correspond average of branch lengths from T-Coffee analyses). (*F*) Structural predictions for the symporter proteins encoded by differentially expressed symporter genes based on human SLC5A8 and SLC24A5. *Left* panel: SACS MEMSAT2 graphs predicting 13 and 12 transmembrane domains for symporter proteins SLC5 and SLC24, respectively. *Right* panel: predicted 3D structures of SLC5 and SLC24 proteins. (*G* and *H*) Conditional downregulation of symporter genes CG1090 and CG6723 in TH neurons affects flight maintenance in tethered flies (*G*) and ability in forced flight experiments (*H*). (*I*) Proposed cellular and molecular model of Ubx-dependent control of flight behavior in *Drosophila*. Error bars in figures represent SEM. Significant values in all figures based on Mann–Whitney *U* test or one-way ANOVA with the post hoc Tukey–Kramer test: ^∗^*P* < 0.05.

To advance the understanding of the mechanisms that link a reduction in *Ubx* expression with the observed physiological change in TH neurons with impact on flight performance (Figs. 1 and 4), we conducted an RNA-sequencing experiment aimed at determining the transcriptome of TH neurons from the adult VNC with and without normal levels of Ubx. For this, we used fluorescence-activated cell sorting (FACS) to isolate populations of TH neurons from the VNC expressing normal or downregulated *Ubx* (*TH>Ubx^RNAi^*), extracted RNA, and compared the resulting transcriptomes using RNA-seq (Fig. 6*A*). Using edgeR analysis (34), we identified 233 differentially expressed genes (DEGs) (out of 5,708 total genes detected) in *TH>Ubx^RNAi^* neurons relative to wild-type (wt) neurons (*P* value < 0.01; Dataset S1) (Fig. 6*B*). Using the DAVID platform (Database for Annotation, Visualization and Integrated Discovery (35)), we established the functional biological properties of the 233 DEGs.

As a first step in the search for candidate genes that might perform Ubx-dependent neurophysiological roles in TH neurons, we considered the top-ten DEGs (Fold change, FC > 5), and here, our attention was immediately caught by the second gene class with the highest differential expression, which included four previously uncharacterized ion symporter genes that belong to the solute carrier (*SLC*) gene superfamily, predicted to regulate sodium, calcium, or potassium transport (Fig. 6*C*) (see below) (i.e., gene ontology (GO) terms enriched, *P* value < 0.05). Given that the concentration of ions inside and outside the neuron can directly affect its activity and function, we decided to focus on these symporter genes and test their role in flight control. These genes are substantially downregulated in response to a reduction of *Ubx* expression (≥10 FC, *P *< 0.004; Fig. 6*D*), and include: *CG1090*, *CG5687*, *CG9657*, and *CG6723* (Fig. 6*D*). Gene tree (Fig. 6*E*), protein alignment analysis (*SI Appendix*, Figs. S7 and S8), and protein structural predictions (Fig. 6*F* and *SI Appendix*, Figs. S7 and S8) using EMBL-EBI Clustal Omega, Jalview program (36) and JPred secondary structure prediction programs(37) for these symporter genes reveal that they belong to two independent lineages within the *SLC* family: *SLC5* and *SLC2*4 (38–41). Although these genes have not been previously characterized in flies, *SLC24* genes have been shown to encode a diverse group of Na^+^/Ca^2+^-K^+^ exchangers (NCKX) (39) in other species and have been previously shown to play roles in nutrient sensing and sleep control in insects and mammals (38, 39, 41, 42), and their dysfunction is correlated with neurological disease (38, 43, 44). Furthermore, expression data from the FlyAtlas expression database (45) reveal that these four symporter genes are also primarily expressed in the *Drosophila* CNS, including the VNC (Dataset S2). Put together, these features suggest the hypothesis that SLC genes might play functional roles in the fly nervous system and represent biochemical mediators through which Ubx exerts its roles on flight. To test whether normal expression of these symporter genes in TH neurons is required for flight maintenance, we examined flight behavior (tethered flight and forced flight) following TH-specific RNAi-mediated downregulation of these genes in normal flies. Our results show that TH-specific expression of RNAi constructs against *CG1090* and *CG6723* leads to an impairment of flight maintenance and ability in wild-type flies (Fig. 6 *G* and *H* and *SI Appendix*, Fig. S9) indicating that normal expression of these symporter genes in TH neurons is necessary for normal flight. Finally, we combined FACS cell-sorting and RT-PCR assays to confirm the expression of *CG1090* and *CG6723* in different neuronal subpopulations including sensory, glutamatergic, and dopaminergic neurons (*SI Appendix*, Fig. S10), as well as their reduction in the presence of their respective RNAi constructs (*SI Appendix*, Fig. S11) or under reduced *Ubx* expression (*SI Appendix*, Fig. S12). Based on the evidence presented above, and considering the central roles played by ion dynamics in normal and abnormal neural physiology, the *SLC* genes emerge as a promising model to understand the impact of *Hox* regulation on neural function in the adult. Altogether, these experiments suggest a molecular framework that links *Ubx* gene regulatory roles in TH neurons to flight control (Fig. 6*I*).

Our study shows that once development has concluded, the *Hox* genes contribute to the genetic control of neural function in the adult, enabling normal behavior. Our investigation on Ubx post-developmental function shows that its expression in the adult nervous system is required for normal flight. We map the focus of action of Ubx to the dopaminergic system in the VNC and show that a reduction of Ubx in these neurons leads to a reduction of neural activity. Cell circuitry analysis determines that dopaminergic neurons are directly linked to a system of motorneurons that controls flight muscles.

The transcriptomic identification of genes sensitive to a conditional decrease in Ubx within the dopaminergic system highlights the *SLC* system. To gain further understanding on the mechanisms underlying *Ubx* neural roles in the adult, future experiments will map and elucidate the contributions of the *SLC* gene system to flight control and neural function—for instance, through expression of corrective upregulation of each *SLC* gene in a conditionally reduced *Ubx* expression background—and establish the exact molecular pathways that link *Ubx* to *SLC* gene regulation, which might be direct, or indirect. While based on what we currently know about their molecular identity, expression profiles, and functional roles, the *SLC* genes do show high promise as potential mediators of the phenotypes observed, but further work is needed to investigate the roles of these genes in neurons in higher detail (including physiological and structural analyses). In addition, we shall probe the physiological and behavioral contributions of other differentially expressed genes detected in our transcriptomic analysis, considering putative additive as well as combinatorial effects that might emerge from parallel *Ubx*-dependent regulatory processes acting on multiple loci.

Our work reveals that normal *Ubx* expression in the adult nervous system is required for normal flight, revealing a novel post-developmental role of the *Hox* genes in adult behavior. These observations open a new avenue to investigate the molecular programs that maintain normal adult neural physiology. Based on the broad evolutionary conservation of the *Hox* system across distantly related animal taxa, we predict that the *Hox* genes might play neurophysiological roles in the adult forms of other species, including humans.

## Materials and Methods

Detailed experimental materials and methods can be found in the *SI Appendix*, *Materials and Methods*.

### Behavioral Assays.

Our study uses a range of quantitative behavioral tests to evaluate the impact of changes in gene expression. In brief, for tethered fly tests (46, 47), a brief air puff was delivered to a fly suspended from a thin metal wire (attached via UV-activated glue (BONDIC) applied to the thorax) and flight maintenance capacity of individual flies was recorded for 10 min. For the takeoff assays, flies were put in a circular arena covered with glass and flies capable of flight were scored after lifting the glass cover. Negative geotaxis was assessed using climbing assays (48, 49): A group of flies were put in a vertical glass column, and the climbing performance for each column was calculated for 25 s after a startle induction; the distribution of flies at the top and bottom of the column was used to generate the score.

### Conditional Expression Tests.

We conditionally expressed *Ubx^RNAi^*, *Kir2.1*, *Nachbac* or other transgenes in a *Tub-Gal80^t^*^s^ background, shifting animals from permissive (18°C) to non-permissive (30°C) temperature upon eclosion. All behavioral assays (see above) were performed at room temperature (25°C).

### Immunohistochemistry.

VNC were dissected in 1× PBS. Tissues were then fixed for 1 h in 4% formaldehyde in 1× PBS at room temperature. After fixation, brains and VNCs were washed three times (30 min per washing) in PBS with 0.3% Triton X-100 (PBTx) and incubated at 4°C overnight with primary antibodies. The following primary antibodies were used: mouse monoclonal anti-Ubx (FP3.38 (5) 1:500) from the Developmental Studies Hybridoma Bank), rabbit anti-TH (Novusbio), mouse anti-nc82, and chicken anti-GFP (Abcam Probes, 1:3,000). The secondary antibodies were anti-mouse Alexa Fluor 555 (Invitrogen Molecular Probes, 1:1,000), anti-rabbit Alexa Fluor 647 (Invitrogen Molecular Probes, 1:1,000), and anti-chicken Alexa Fluor 488 (Invitrogen Molecular Probes, 1:1,000). Images were acquired with a Leica SP8 confocal microscope, processed, and analyzed using FIJI ImageJ (NIH).

### Optogenetic Experiments.

Optogenetic experiments were conducted by adapting the Flypi device (50). For neuronal activation (CsChrimson, Pwr_590_) and inhibition (GtACR, Pwr_470_) a Neopixel 12 light-emitting diodes ring was positioned face-down around the infrared camera objective, about 3 cm above the tethered flies. Flies were recorded with “lights off” (in the dark) and the response of each animal (males and females) was analyzed during the following “light on” period. We exposed flies to approximately 4.9 W/cm^2^ for stimuli between 500–1,000 ms using a custom-written Graphical User Interface (50). For all optogenetic activation experiments, adult flies upon eclosion were kept for 7–8 d before the experiment on food containing 0.5 mM all-trans retinal (Sigma).

Further experimental details on all the methods above, as well as information on fly stocks, calcium activity recordings, RNA analysis, and other methodologies can be found in *SI Appendix*, *Materials and Methods* section.

## Supplementary Material

Appendix 01 (PDF)

Dataset S01 (PDF)

Dataset S02 (PDF)

Movie S1. Assessing flight in normal and post-developmentally reduced Ubx conditions. Related to Fig. 1. Movie showing a top-view of four individuals: two on the left kept at 30°C (deactivated Gal80ts), and two on right kept at 18°C (active Gal80ts). At each temperature condition, the individual on the left include a TH-Gal4 driver; all individuals include a *UAS-Ubx^RNAi^* construct and a *Tub-Gal80ts* construct (labelled). Flight is triggered by a gentle air puff applied simultaneously to all individuals.

Movie S2. Muscle activity during sustained flight in normal conditions. Related to Fig. 1. Fluorescence imaging of thoracic flight muscles (dorsal longitudinal muscles) expressing the calcium reporter GCaMP6m (green signal) in a normal fly during sustained flight.

Movie S3. Muscle activity during sustained flight in post-developmentally reduced Ubx conditions. Related to Fig. 1. Fluorescence imaging of thoracic flight muscles (dorsal longitudinal muscles) expressing the calcium reporter GCaMP6m (green signal) in postdevelopmentally reduced Ubx conditions (*TH>Ubx^RNAi^*) during sustained flight.

Movie S4. Optogenetic activation of TH neurons triggers flight. Related to Fig. 3. Adult fly expressing CsChrimson in TH neurons (left) and a control fly (right). Optogenetic activation of CsChrimson by red light triggers flight only in the specimen on the left.

Movie S5. Optogenetic inhibition of TH neurons reduces flight performance. Related to Fig. 3. Adult fly expressing GtACR in TH neurons (left) and a control fly (right). Optogenetic inhibition of GtACR by blue light leads to a reduction in wing beat frequency only in the specimen on the left.

Movie S6. Optogenetic inhibition applied within the Ubx domain reduces flight performance. Related to Fig. 3. Adult fly expressing GtACR within the *Ubx* domain (left) and a control fly (right). Optogenetic inhibition of GtACR by blue light stops flight instantly.

Movie S7. Optogenetic activation of Ubx^+^ TH neurons triggers flight. Related to Fig. 3. Adult fly expressing CsChrimson in Ubx^+^ TH neurons (intersection of Ubx and TH domains using the split-Gal4 approach) (left) and a control fly (right). Optogenetic activation of CsChrimson by red light triggers flight only in the specimen on the left.

Movie S8. Optogenetic activation of Dlm motor neurons triggers flight. Related to Fig. 5. Adult fly expressing CsChrimson in Dlm motor neurons (left) and a control fly (right). Optogenetic activation of CsChrimson by red light triggers flight only in the specimen on the left.

Movie S9. Optogenetic activation of TH neurons triggers activity in Dlm motor neurons. Related to Fig. 5. Fluorescence imaging of a fly expressing GCaMP6m in Dlm motor neurons projecting into the Dlm muscles. When TH neurons expressing CsChrimson are activated by red light, activity in the projections of Dlm motor neurons is triggered (green signal).

Movie S10. Simple take-off response in normal wild type adult *Drosophila*. Related to figure 1. The movie shows a top- view of a take-off behavioural arena. Lifting the glass plate triggers an escape reflex, with most flies immediately taking-off from the arena, and swiftly moving away from the centre of the field of view (for quantifications see Figure S2).

Movie S11. Simple take-off response in adult Drosophila with post-developmental downregulation of *Ubx* expression. Related to figure 1. The movie shows a top-view of a take-off behavioural arena. Lifting the glass plate triggers an escape reflex in normal flies; in contrast, flies in which Ubx had been post-developmentally downregulated (TH>UbxRNAi) do not show this reflex, and remain close to centre of the arena. Note that even when prompted by a paintbrush, flies do not show a standard take-off response (for quantifications see Figure S2).

Movie S12. Ubx expression in TH neurons. The movie displays the VNC of adult *Drosophila* showing expression of Ubx (red) in TH neurons within T2 and T3 VNC segments labelled by GFP driven by TH-Gal4.

Movie S13. TH neuron patterns in the whole-VNC and brain. The movie displays whole-VNC and brain expression of TH-Gal4 driving Myr::GFP (green) and anti-nc82 neuropil counterstaining (magenta). This shows that TH neuron soma and projections/axons are located in the brain as well as in the VNC.

Movie S14. Ubx cell patterns in the whole-VNC and brain. The movie displays whole-VNC and brain expression of Ubx-Gal4 driving Myr::GFP (green) and anti-nc82 neuropil counterstaining (magenta). This indicates that Ubx cell soma are exclusively located in the VNC T2 and T3 segments and send axons in the VNC as well brain.

Movie S15. Ubx positive TH neurons in the whole-VNC. The movie displays whole-VNC expression of *UbxGal4.^DBD^* ∩ *ple^Gal4.AD^* (Ubx∩TH-Gal4) driving mcd8::GFP (green) and anti-nc82 neuropil counterstaining (magenta). This shows that Ubx+ TH neurons soma are exclusively located in the VNC T2 and T3 segments and have their axons around the neuromeres and the medial axis of VNC. This also demonstrates that Ubx+ TH neurons have their axons both in the VNC and brain.

## Data Availability

All study data are included in the article and/or *SI Appendix*.
