# Supplementary material for: A novel post-developmental role of the Hox genes underlies normal adult behavior
Source: Proc Natl Acad Sci U S A. 2022 Dec 1;119(49):e2209531119. doi: 10.1073/pnas.2209531119 (PMC9894213; doi:10.1073/pnas.2209531119)
Supplement: Supplementary file 1 — Appendix 01 (PDF) [file pnas.2209531119.sapp.pdf]

PNAS

Supporting Information for

A novel post-developmental role of the Hox genes underlies normal adult behaviour

A. Raouf Issa, Jonathan Menzies[#], Aishwarya Padmanabhan[#] and Claudio R. Alonso[*]

[#]*Equal contribution*

Corresponding author: Claudio R. Alonso
Email: c.alonso@sussex.ac.uk

This PDF file includes:

Supporting Materials and Methods
Figures S1 to S12
Legends for Movies 1 to 15
Legends for Datasets S1 to S2
SI References

Other supporting materials for this manuscript include the following:

Movies 1 to 15
Datasets S1 to S2

SUPPORTING MATERIALS AND METHODS

Fly rearing and stocks

Drosophila cultures were reared at 25℃ in standard culture tubes or bottles, and on corn agar media supplemented with water, cornmeal, molasses, yeast, nipagin, and propionic acid. Stocks and crosses were reared on standard medium (water, agar, cornmeal, molasses, yeast, nipagin, propionic acid) in standard tubes or bottles, and maintained at 25˚C in a temperature-controlled incubator at 50% humidity with a 12 h/12 h cycle of alternating light and dark. The following Gal4 drivers from the Bloomington *Drosophila* Stock Center (BDSC) were used: pan-neuronal Gal4 (Elav-Gal4), cholinergic (Cha-Gal4)(BDSC, 60317), Glutamatergic (VGlut-Gal4) (BDSC, 26160) and Ubx-Gal4 (a gift from Ernesto Sánchez-Herrero (1)). The dopaminergic GAL4 (TH-GAL4) was kindly provided by Serge Birman (CNRS, ESPCI Paris Tech, France) (2). In addition, GAL4 drivers for specific sets of motoneurons that innervate the flight muscles(3) were used, including: VT021842.GAL4 (VDRC, 203853*),* VT033051.GAL4 (VDRC, 200860), VT040232.p65ADZp.attp40 (BDSC, 71651), VT029310.ZpGAL4DBD (DBSC, 74584), VT045969.p65ADZp.attp40 (BDSC, 73438), and VT042475.ZpGAL4DBD.attp2 (BDSC, 72758). We combined UbxDBD/R32D08-Gal4.DBD(BDSC, 68670), ple-p65.Gal4.AD (BDSC, 70861) to make split-Gal4 lines. We used UAS strains that express a panel of dopamine receptor RNA interference constructs: DR2/Dop1R2/DAMB (VDRC, 105324, BDSC, 26018), DR1/Dumb/Dop1R1 (VDRC, 107058), D2R/Dop2R (VDRC, 11470, 11471), DopEcR (VDRC, 103494), UAS-CG1090-RNAi-1 (VDRC,26783), UAS-CG1090-RNAi-2 (VDRC,26784), UAS-CG5687-RNAi-1 (VDRC,33024), UAS-CG5687-RNAi-2(VDRC,33262), UAS-CG6723-RNAi-1(VDRC,13769), UAS-CG9656- RNAi-

1(VDRC,43922), UAS-CG9656- RNAi-2 (VDRC,10736), and Ubx-RNAi (31913 and 34993, referred respectively to as Ubx-RNAi-1 and UbxRNAi-2 in the article), as well as mutant Ubx (1, 4) and a Tub-GAL80^{ts} (BDSC, 7018 and 7108). *UAS-n-syb::spGFP$_{1-10}$*, *LexAop-CD4::spGFP$_{11}$/CyO* and *LexAop-n-syb::spGFP$_{1-10}$*, *UAS-CD4::spGFP$_{11}$* (GRASP)(BDSC, 58755 and 58754)(5). The Mef2-LexA line was a kind gift of Scott Waddell (Centre for Neural Circuits and Behaviour, University of Oxford, Oxford)(6). TH-LexA (originally produced by Ronald L. Davis) was provided by Serge Birman (CNRS, ESPCI Paris Tech, France) (7), and UAS-GtACR2 was obtained from Adam Claridge-Chang (Program in Neuroscience and Behavioral Disorders, Duke-NUS Medical School, Singapore) (8), 8XLexAop2-FLPL (BDSC, 55819), UAS-CsChrimson (BDSC, 55819 and 55135), UAS-NaChBac (BDSC, 9469), UAS>stop>ReaChR(BDSC, 53743) (9), UAS-Kir2.1 (BDSC, 6595), UAS-Myr::GFP (BDSC, 31913), UAS-Kir2.1 (BDSC, 6595), UAS-UbxFLP (42719, 42720) and UAS-Shibire^{ts} (BDSC, 44222)(10), LexAop-FLPL (BDSC,55819), LexAop-CsChrimson (II)(BDSC,5513),LexAop-CsChrimson (III)(BDSC,55139), LexAop-GCAMP6m (BDSC,44275), UAS-FLP (BDSC,4539), and UAS-SytGFP (BDSC,6925). UAS-Nls:GFP (BDSC, 4776); lexAop-CD8-GFP-2A-CD8-GFP;UAS-mLexA-VP16-NFAT,LexAop-rCD2-GFP (CaLexA) (BDSC, 66542), UAS-GAMP6 (BDSC, 42748) (11). w^{1118} was used as control strain. Wild-type, w^{1118} flies served as control in all experiments or were used to generate heterozygous flies. For silencing neurons by expressing *Kir2*.1 or *shibire*^{ts}, eggs, embryos, larvae and pupae were raised at 18 °C and the freshly hatched flies were transferred to 33°C for behavioural tests after 10 days. All the experiments were performed at 25˚C. For optogenetic experiments, flies were transferred to vials containing retinal food (regular fly food mixed with 0.5 mM

all-trans retinal (Sigma) after collection and reared in the dark for 7-8 days before the experiment. Flies were flipped to fresh food media one day before a behavioural test. Both females and males were used in this study.

Flight assays

For tests conducted on tethered *Drosophila*, we followed the protocol described by Dickson et col.,(12) and Mauss et col. (13). In brief, flies were anaesthetised on ice for 15 min and tethered to the tip of a thin metal wire (Insect pins 0.1mm, Agar Scientific) with UV-activated glue (BONDIC). After 20 min of acclimatisation at room temperature, a brief air-puff was delivered to the animal, and subsequently, videos were recorded during 10 min via a Basler ACE acA1300-200uc Colour USB 3.0 Camera and TECHSPEC® 6mm UC Series Fixed Focal Length Lens, with a resolution of 1,280 x 1,024 pixels at 50 Hz to measure flight duration for each genotype. For forced flight assays, we proceeded as previously described (Fig. 1F) (14). We tapped groups of (n ≥12) flies (males/females) through a funnel in a transparent plastic cylinder (90 cm high, 13.5 cm inner diameter) into which we introduced a folded polyacrylamide sheet (90 cm high) coated with Tangle-Trap (The Tanglefoot Company), an adhesive designed to capture live insects. A bucket with water was placed underneath the cylinder. Five minutes after the flies were released the plastic was removed from the cylinder, unfolded coated faceup on a flat white surface, and its top view captured by a digital camera. Lastly, FIJI image J software was used to automate the scoring of flight performance. The polyacrylamide sheet was divided in 3 zones: top> 80 cm, bottom < 2 cm, and bottom<medium>top, and forced flight index was

scored according to the following formula: $(n_{top}-n_{bot})/2n_{tot}$, where n_{tot} is the total number of flies, and n_{top} and n_{bot} the number of flies at the top and at the bottom, respectively. The number of flies landing in the bucket were included in the 'bottom' group. For take-off of voluntary flight assays, 4-6 flies were introduced one by one into 5 cm diameter circular arena with sloped edges (11°) covered with glass that was pre-coated with Sigmacote (SIGMA-ALDRICH). This arena triggers an escape reflex to the point that lifting the glass cover allows flies to immediately take-off from the open container. We next, lifted the lid of the arena and after 2 min take-off was noted as 'successful' for flies that did not land within a radius of 7 cm.

Climbing assays

Startle-induced negative geotaxis (SING) test was used as climbing assay, and was monitored as previously described (15, 16).A group of 10-20 flies were introduced in a vertical glass column (15cm long, 1.5cm inner diameter). Each group was assayed 3 times at 10 min intervals. The climbing performance for each column was calculated as follows: $(n_{tot} + n_{top} - n_{bot})/2n_{tot}$, where n_{tot} is the total number of flies, and n_{top} and n_{bot} the number of flies at the top and at the bottom, respectively. Results are the mean and SEM of the scores obtained with the 5 groups of flies per genotype.

Imaging calcium activity in thoracic muscles and CaLexA assays

Calcium activity recording in thoracic muscles was conducted on a tethered fly, using epifluorescence microscopy (Leica DM6000B). Data was analysed using FIJI Image J software (NIH), and Igor Pro 8 (Wavemetrics) (17) to quantify calcium signal and generate traces, respectively. The (CaLexA (Calcium-dependent nuclear import of LexA) technique (18) was employed to measure calcium activity in dopamine and Ubx^+ neurons. Briefly, CaLexA expressing flies were raised at 18ºC. After eclosion temperature was shifted to 30ºC, and males and females were separated and put into fresh vials. To restrict fly movement, a cotton plug was placed close to the surface of food and vials were covered by aluminium foil. The day before the experiment animals were moved to 25ºC for acclimatisation. The day of the experiment animals were cold-anaesthetised on ice for 15 min prior to tethering. A group of tethered flies were allowed to hold on the ground with their legs to prevent spontaneous flight (resting condition) in contrast to others, which were kept on suspension ('flying' condition). A single blown air-puff stimulus was delivered to the animals to trigger flight (a single brief air-puff stimulus is sufficient to trigger flight in tethered *Drosophila*). After 30 min of flight, or cessation of the flight, animal VNCs were dissected and processed for immunohistochemistry as described above. Samples were visualized with a Leica SP6 confocal microscope and images processed in FIJI ImageJ. All settings were kept constant between experimental conditions. For CaLexA experiments, GFP fluorescence was quantified in a region of interest (ROI) based on single optical sections from whole-mount fly VNCs; average GFP signal for each individual sample was calculated by dividing total GFP fluorescence intensity by the number of visible cells (GFP/number of cells).

RNA extraction and reverse transcription

RNA was extracted from FACS-sorted GFP-positive neurons (300-400 cells) suspended into 150μL of trizol (Invitrogen) or dissected adult VNC samples (10-15 VNCs) dissolved in 500 μl TRIzol. Following addition to TRIzol, homogenisation of the sample was performed with an electric pestle for 1 min. RNA was separated through the addition of 100 μl RNase-free chloroform, mixing by vortex for 30 secs and centrifugation at 12000 x g for 10 mins at 4℃. The upper aqueous phase was aspirated and 200 μl was mixed with 250 μl isopropanol and 1ul GlycoBlue coprecipitant prior to a 10-min incubation at room temperature. Samples were then centrifuged at 12000 x g for 10 mins at 4℃ and the resulting RNA pellet was washed twice with 750 μl 70% ethanol before being air dried for 3 minutes and resuspended in 14 μl RNase-free water. Reverse transcription was performed using the SuperScript IV First-Strand Synthesis System with ezDNase Enzyme (Invitrogen) as per the protocol provided by the manufacturer. Up to 500 ng total RNA were first treated with the ezDNase enzyme to remove genomic DNA prior to reverse transcription with the SuperScript IV enzyme and oligo(dT) primer. Complementary DNA (cDNA) made was stored at -20℃. A control reaction without the reverse transcriptase enzyme (no-RT control) was performed for each sample to confirm that genomic DNA was effectively removed during the DNase step and therefore would not significantly influence downstream applications.

PCR and Real-time PCR

PCR was performed in a BioRad T100 Thermal Cycler and with standard Taq DNA polymerase (New England Biolabs) using a single cycle (5 min) of denaturation at 94°C;

30 s of denaturation at 94°C, 45 s of annealing at 60°C and 45 s of elongation at 72°C, repeated 40 times; and a single cycle 5 min of extension at 72°C. The following primers were used for *CG1090*, forward: 5'-GGATCGTCTACTGGACGCAG; reverse: 5'-ATCCAGACTGGCCACAAAGT (506bp amplicon); for *CG6723*, forward: 5'-GGATCGTCTACTGGACGCAG; reverse: 5'-ACTAGGCACACGCAGAGAAC (513bp amplicon). For Real-time PCR, experiments were performed with cDNA made from dissected adult VNC tissue. Reactions were performed using LightCycler SYBR Green I reagents (Roche) and run in MicroAmp Optical 96-well reaction plates (Applied Biosystems) in a QuantStudio 3 Real-Time PCR System (Applied Biosystems). All reactions were run with from three biological replicates and any groups compared in downstream analysis were run at the same time. Primer efficiency was determined by a standard curve of 6 cDNA dilutions and only those with efficiencies between 1.9 and 2.2 were used. The amplification program included 10 s of denaturation at 95°C, 20 s annealing at 60°C and 20s elongation at 72°C, repeated 40 times. In all experiments, the reference gene Actin42A was used to normalise for differences in sample quantities across genotypes tested. The following primer were used: for *actin*, forward: 5'-GCGTCGGTCAATTCAATCTT; reverse: 5'-AAGCTGCAACCTCTTCGTCA (138 bp amplicon); for *CG1090*, forward: 5'-AGTTCCGTTCTGGACACAACA; reverse: 5'-CTGTGGAAACTGCTCGATAGC (135 bp amplicon); and for *CG6723*, forward: 5'-TCCTGCTCTGCATCGGTATTG; reverse: 5'-GGTATGACCAACATCTTTCGCC (100 bp amplicon).

RNA sequencing

Drosophila VNCs were dissected and prepared for FACS using a protocol adapted from DeSalvo et al., 2014 (19). In brief, adult VNCs we dissected in a 30-min window in ice cold PBS (ref: D8537; Sigma-Aldrich), transferred into a dissociation solution of collagenase A (ref: 10103578001; Roche)/PBS with final concentration of collagenase A of 1.5mg/ml, and incubated at 37°C for 30 minutes. Tissues were fragmented by pipetting up/down (10-20 times) and the resulting cell suspension was filtered through a 35µm nylon mesh into a cytometry tube. GFP positive neurons were immediately sorted by flow cytometry (BD FACS Melody) into 5 µl of PBS and flash frozen in liquid Nitrogen. Flash frozen cell samples were sent to the *Cambridge Genomic Services* for library preparation using the SMART-Seq® v4 Ultra® Low Input RNA Kit (Takara Bio USA, Inc) and after checking library composition to evaluate the representation of different RNA biotypes, we proceeded to RNA sequencing (NextSeq 74 cycle high output 15-20 M reads per sample) and bioinformatic analysis using Drmelanogaster6 as reference for processing and alignment. Data was extracted as Total read count per gene, read per gene Length (Kp) – RPK, and RPK/million Mapped reads – RPKM. We used a *generalised linear model* edgeR approach to make pairwise comparisons between groups. We then adjusted for multiple testing via the FDR (Benjamini-Hochberg) approach used by edgeR. The comparisons between genotypes have been performed without outlier pairing and low-features pairing samples. We used the Pearson's R correlation coefficient for the analysis between all samples, and variable correlation for the individual sample groups. For reads counting, the number of reads that mapped to genomic features was calculated using HTSeq v0.6.1; reads with a mapping quality of less than 10, or those which mapped to

multiple loci, or to overlapping gene regions were discarded to avoid ambiguity and false positives. Lastly, fold-change between samples were calculated as a ratio of experimental samples (Tub-Gal80ts,TH>Ubx^{RNAi},GFP) over wild-type (Tub-Gal80ts,TH>GFP) ones (experimental/wild-type).

Dendrogram generation and homology analysis

We used EMBL-EBI Clustal Omega (http://www.ebi.ac.uk/Tools/msa/clustalw2/) to conduct protein sequence alignment of symporter genes. We used the Jalview Version 2-a program and the T-Coffee method for multiple sequence alignments and visualisation, and for phylogenetic tree generation (20). From Jalview we applied JPred 4 programs (21) to produce secondary structure prediction. Subsequently, Jmol and Chimera viewers inside the Jalview desktop, and SACS MEMSAT2 (22) were employed to generate predicted molecular structures of SLC5 and SLC24 clusters.

Quantification and statistical analysis

Statistical analyses of data were performed in GraphPad Software Prism using Mann-Whitney U test or one-way ANOVA with the post-hoc Tukey-Kramer test. Error bars in figures represent SEM. Significant values in all Fig.s: $^{*}p < 0.05$, $^{**}p < 0.01$, $^{***}p < 0.001$.

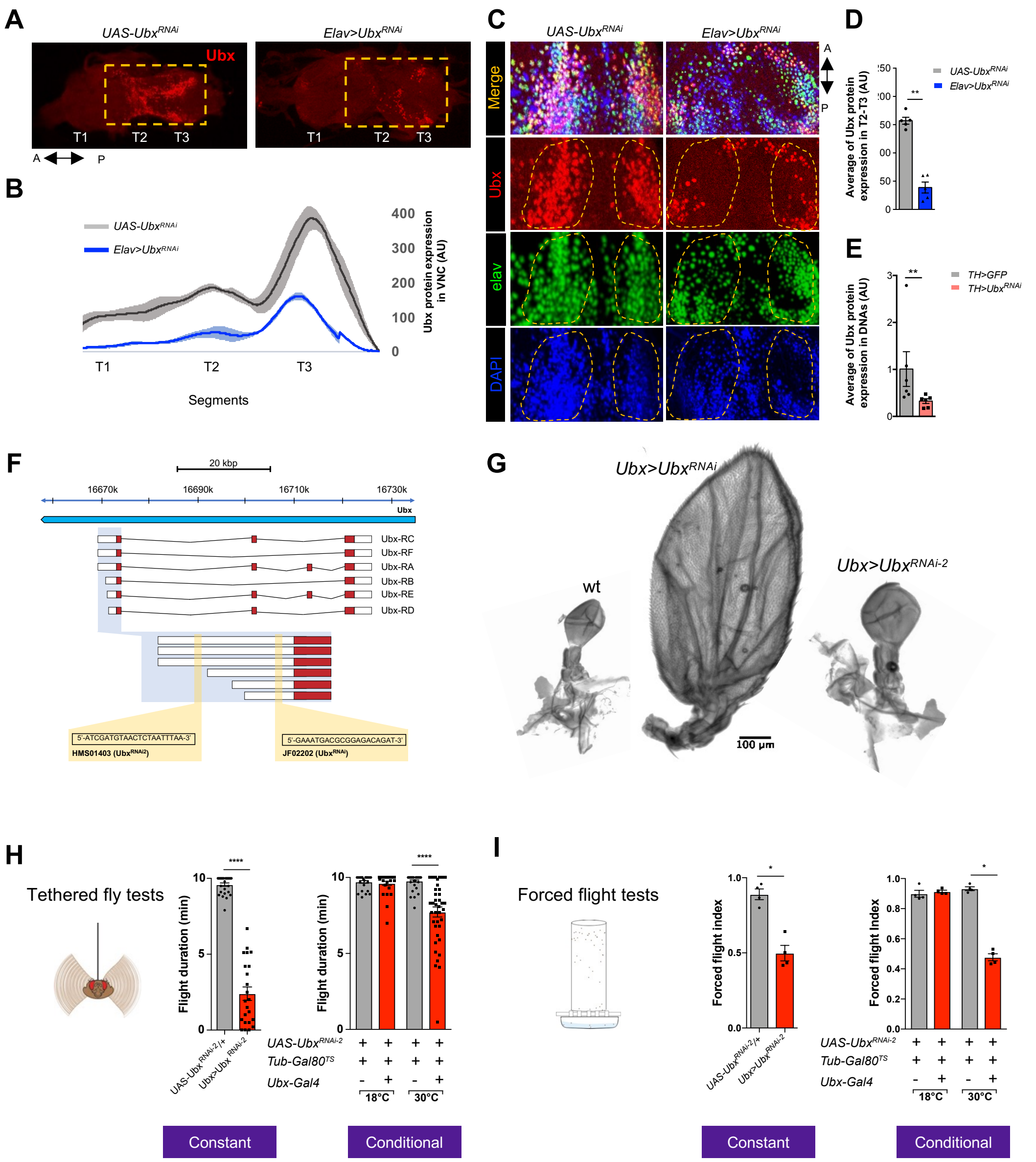

Fig. S1. Effects of Ubx^{RNAi} constructs on Ubx expression and function. Related to figure 1. (**A-D**) Analysis of Ubx protein expression in adult VNCs dissected from control (*UAS-Ubx^{RNAi}*) and experimental flies expressing *Ubx^{RNAi}* pan-neuronally (*Elav>Ubx^{RNAi}*). (**A**) Immunolabelling of Ubx protein (red) in VNCs (anterior to the left) showing a reduction of Ubx signal intensity in *Elav>Ubx^{RNAi}* compared to the control *UAS-Ubx^{RNAi}*. The thoracic segmental register is indicated (T1-T3). (**B**) Profile quantification of Ubx protein across VNC samples (as shown in A) showing a marked reduction in Ubx protein levels when *Ubx^{RNAi}* treatment is applied. (**C**) High-magnification of a VNC region across T2-T3 segments (anterior to the top) from control (left) and *Ubx^{RNAi}* (right) samples showing Ubx (red), Elav (green) and DAPI (blue) signals within dashed areas. Note the marked decrease in Ubx protein intensity when *Ubx^{RNAi}* is expressed in all neurons. (**D**) Quantification of Ubx protein expression in control and pan-neuronally expressed *Ubx^{RNAi}* conditions showing a statistically significant reduction of Ubx protein expression in *Ubx^{RNAi}* samples. (**E**) Quantification of Ubx protein expression in control and experimental samples expressing *Ubx^{RNAi}* in dopaminergic neurons (DANs) by means of the *TH-Gal4* driver showing a statistically significant reduction of Ubx protein expression in *Ubx^{RNAi}* samples. (**F**) Diagram of the *Ubx* locus, indicating the spectrum of RNA isoforms produced by the gene and the genomic location of different *Ubx^{RNAi}* targets (*Ubx^{RNAi}* and *Ubx$^{RNAi\text{-}2}$*). (**G**) Comparative effects of different *Ubx^{RNAi}* constructs on haltere morphology. Note the substantial increase in haltere size caused by *Ubx^{RNAi}* expression, in close resemblance of classic *loss-of-function Ubx* phenotypes. (**H-I**) Effect of a second *Ubx^{RNAi}* (*Ubx$^{RNAi\text{-}2}$*) on adult flight within the Ubx transcriptional domain (*Ubx-Gal4*), as observed in: tethered fly tests (**H**) and forced flight experiments (**I**). Quantification of average flight duration (**H**) and forced flight indexes (**I**) of flies expressing *Ubx$^{RNAi\text{-}2}$* either throughout development (constant) or conditionally after adult eclosion (conditional) shows that when *Ubx^{RNAi}* is activated flight performance is significantly reduced. Error bars in figures represent SEM. Significant values in all figures based on Mann-Whitney U test or one-way ANOVA with the post hoc Tukey-Kramer test: $*p<0.05$, $**<0.01$ and $***p<0.001$.

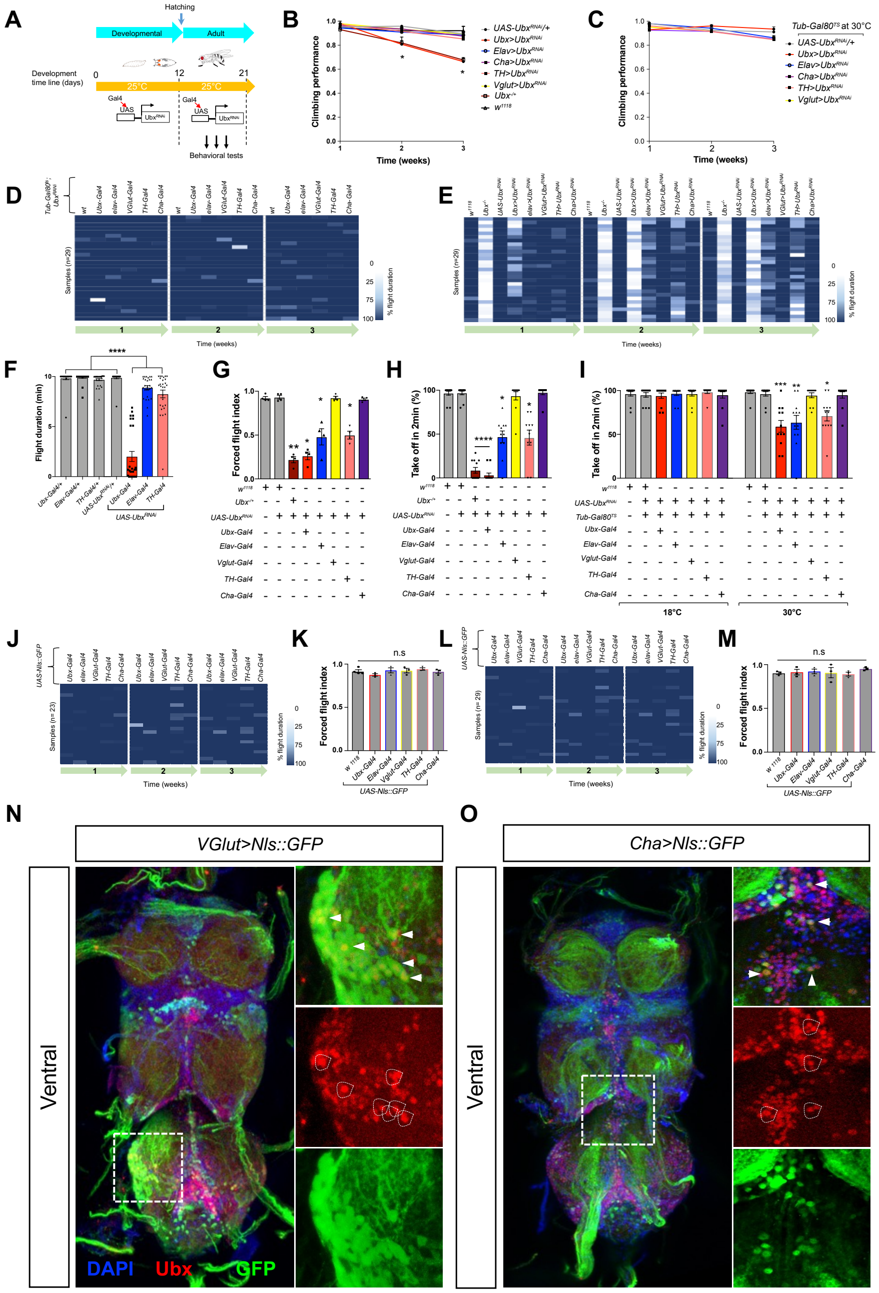

Fig. S2. Ubx downregulation affects flight behaviour. Related to figure 1. (**A**) Strategy for conditional neuronal downregulation of *Ubx* in the adult. (**B**) Climbing performance for non-conditional ("constant") neuronal downregulation of *Ubx* in the adult. (**C**) Climbing performance for conditional neuronal downregulation of *Ubx* in the adult. (**D**) Flight duration in tethered fly experiments represented as a "heat map" (i.e. long flight duration coded in blue, absence of flight coded in white) for flies expressing Ubx^{RNAi} in different neuronal types under temperature-sensitive *Gal80ts* repressor control at 18°C. (**E-G**) Evaluation of flight performance under non-conditional ("constant") neuronal downregulation of *Ubx*. (**E**) Flight duration represented as a heat map (as above); (**F**) Tethered fly tests; (**G**) Forced flight tests. Note that developmental downregulation of Ubx leads to pronounced effects on flight triggered by RNAi expression in DANs but not in glutamatergic or cholinergic neurons. (**H-I**) Take-off ability for non-conditional (constant) (**H**) and conditional (**I**) neuronal downregulation of *Ubx.* See also Movies 10 and 11. (**J-M**) Flies that only express GFP in distinct neuronal populations do not show significant changes in flight performance. (**J-I**) Flight duration in tethered fly tests (left) represented as a heat map (coded as above) and average forced flight index (right) in non-conditional ("constant") conditions (**J-K**), and conditionally-induced GFP expression at 30°C (**L-M**). (**N-O**) Confocal images of the whole VNC of adult *Drosophila* showing expression of Ubx protein in glutamatergic (**N**) and cholinergic (**O**) neurons. Error bars in figures represent SEM. Significant values in all figures based on Mann-Whitney U test or one-way ANOVA with the post hoc Tukey-Kramer test: $*p < 0.05$, $**p < 0.01$, $***p < 0.001$.

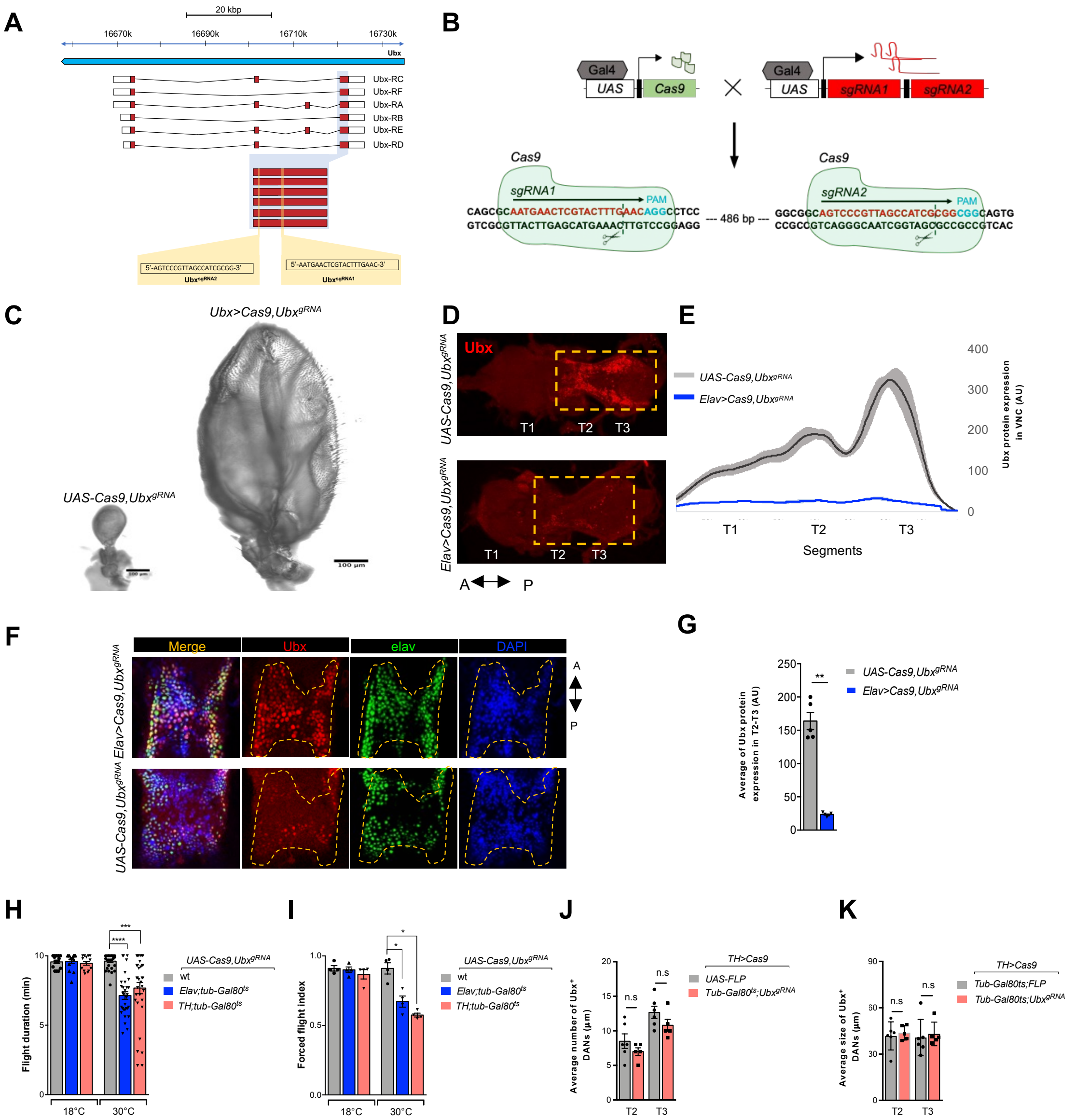

Fig. S3. Effects of Ubx CRISPR-Cas9 knock-out on haltere development, neural Ubx expression and behaviour. Related to figure 1. (**A**) Ubx gene structure and gRNA targets. (**B**) The transgenic line used expresses two (short-guide) sgRNAs under UAS control which target two distinct segments within Ubx exon 1, present in all mRNA isoforms. (**C**) Effects of Ubx KO (*Ubx>Cas9,Ubx^{gRNA}*) on haltere morphology compared to control flies (*UAS-Cas9,Ubx^{gRNA}*). Note the substantial enlargement of the haltere in the UbxKO line, in line with a predicted Ubx *loss-of-function* phenotype. (**D-E**) Global expression of Ubx (red) across tagma (T1-T2-T3) in a control VNC (top, *UAS-Cas9, UbxgRNA*) and in a VNC with pan-neural expression of *Ubx^{gRNA}* (bottom, *Elav>Cas9,Ubx^{gRNA}*). Profile quantification shows that there is a substantial reduction in Ubx protein expression after Cas9 treatment (see graph, right). (**F**) High magnification of the VNC across T2-T3 segments showing Ubx protein expression (red) within equivalent areas (dashed lines) in *elav>Cas9,Ubx^{gRNA}* and control specimens showing a marked reduction of Ubx expression in cas9 samples (elav expression (green) and DAPI (blue) are also shown). (**G**) Quantification of Ubx expression in control (*UAS-Cas9*, *UbxgRNA*) and Ubx KO (*Elav>Cas9, UbxgRNA*) demonstrates a sharp reduction in Ubx expression. (**H-I**) Flight maintenance (**H**) and forced flight performance (**I**) are reduced when Cas9,Ubx^{gRNA} is expressed conditionally in the adult under the control of Elav or TH drivers. (**J-K**) Ubx KO in TH neurons does not lead to a reduction in the number (**J**) or size of TH neurons (**K**) in VNC thoracic segments T2-T3. Error bars in figures represent SEM. (Significant values in all figures based on Mann-Whitney U test: ∗p<0.05; ∗∗p< 0.01; ∗∗∗p< 0.001; ∗∗∗∗p< 0.0001).

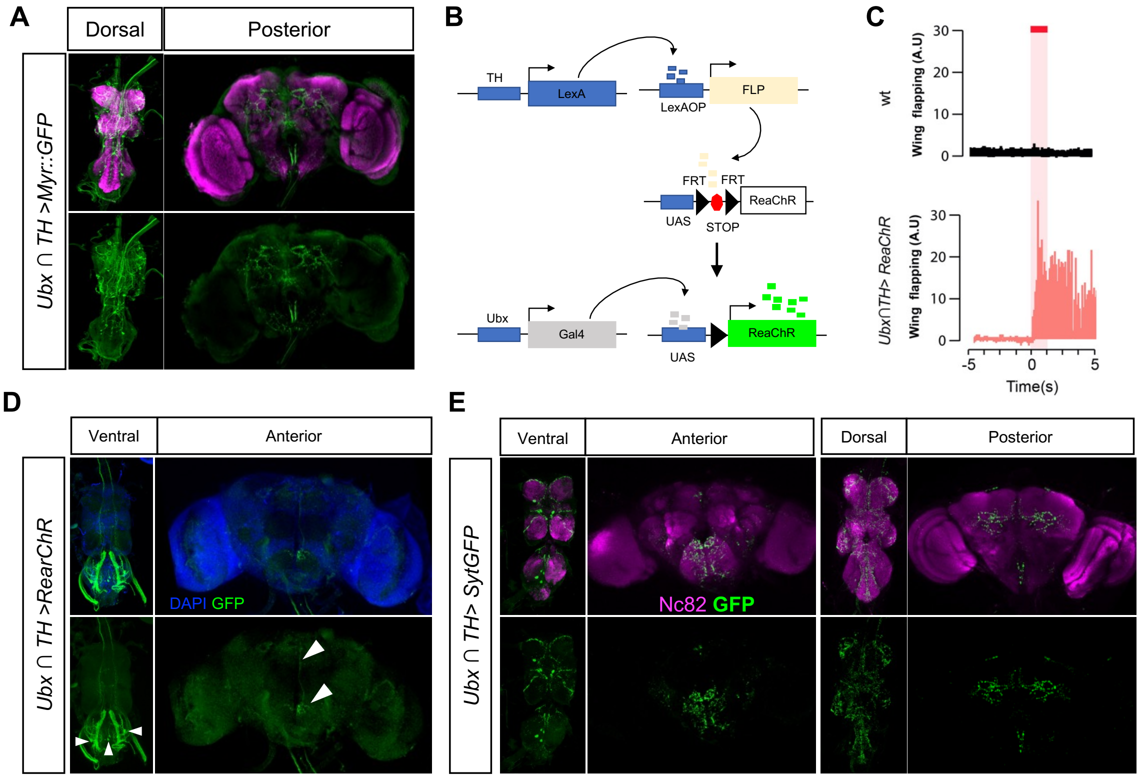

Fig. S4. Ubx+ TH neurons in the VNC are required for flight. Related to figure 3. (**A**) Confocal images of the VNC (left, dorsal view) and brain (right, posterior view) showing UAS-Myr::GFP expression as driven by Ubx∩TH (UbxGal4.DBD ∩ pleGal4.AD; UAS-Myr:GFP). (**B-D**) Activation of Ubx positive TH neurons using the FlpOut method. Schematic illustration of the FlpOut method used to activate Ubx+ TH neurons (**B**) Transcriptional inputs from TH-LexA and Ubx-Gal4 were combined to express channelrhodopsin (ReaChR, green rectangle) at the intersectional transcriptional domain. After heat-shock, FRT-mediated removal of a stop codon (red box) leads to expression of ReaChR only in Ubx+ TH neurons. (**C**) Red light-evoked (red shade) activation of Ubx positive TH neurons expressing channelrhodopsin ReaChR, Ubx∩TH>ReaChR (TH-LexA,FLP-LexAOP & Ubx>ReaChR) (without stop codon, bottom panel) induces spontaneous flight compared to control conditions (TH-LexA,FLP-LexAOP & UAS>stop>ReaChR;Ubx-Gal4)(with stop codon, top panel). (**D**) Expression of ReaChR (Green signal, labelled by anti-GFP antibody) in Ubx positive TH neurons in the VNC (left panel) and brain (right) after light-evoked activation shows a successful transcriptional activation of the UAS-ReaChR transgene at the Ubx∩TH intersectional domain. (**E**) Expression of the post-synaptic marker synaptotagmin-GFP (sytGFP) as driven by Ubx∩TH (UbxGal4.DBD∩pleGal4.AD; UAS-SytGFP) in the VNC and brain. NB: Nc82 signal (purple) demarks the neuropil providing a reference for areas of sytGFP expression.

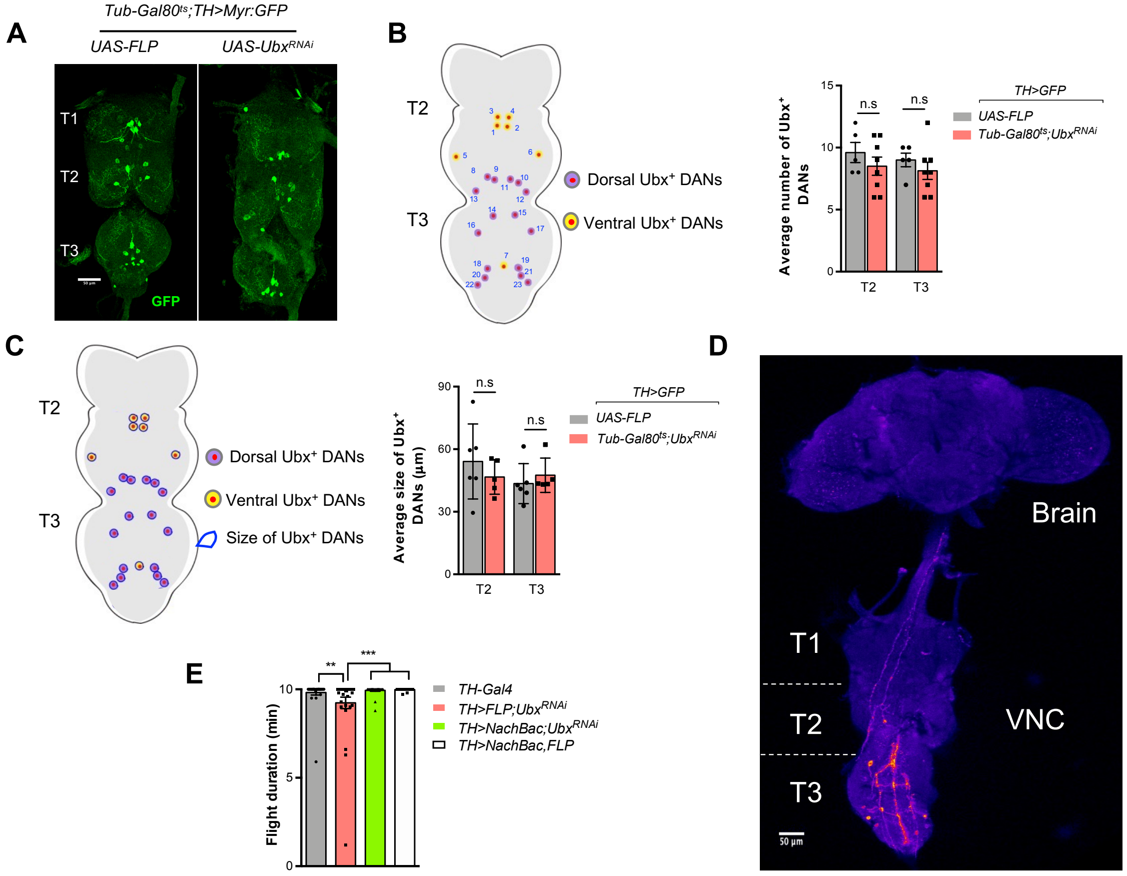

Fig. S5. Effects of Ubx downregulation on dopaminergic neuron integrity and activity. Related to figure 4. (**A-C**) Anatomical analysis of TH neurons labelled via GFP (**A**) shows that Ubx downregulation does not lead to significant changes in the number (**B**) or size (**C**) of TH neurons in VNC thoracic segments T2 or T3 (Genotypes: *TH-Gal4;UAS-Myr:GFP,Tub-Gal80ts;UAS-FLP* and *TH-Gal4;UAS-Myr:GFP,Tub-Gal80ts;UAS-Ubx^{RNAi}*). (**D**) Flight induces an increase in CaLexA signal in TH neurons within the VNC (see bright pink signal), but, not in the brain. (**E**) Expression of NaChBac in TH neurons rescues the flight deficits induced by Ubx downregulation. (Error bars in figures represent SEM. Significant values in all figures based on Mann-Whitney U test or one-way ANOVA with the post hoc Tukey-Kramer test: $**p< 0.01$; $***p< 0.001$).

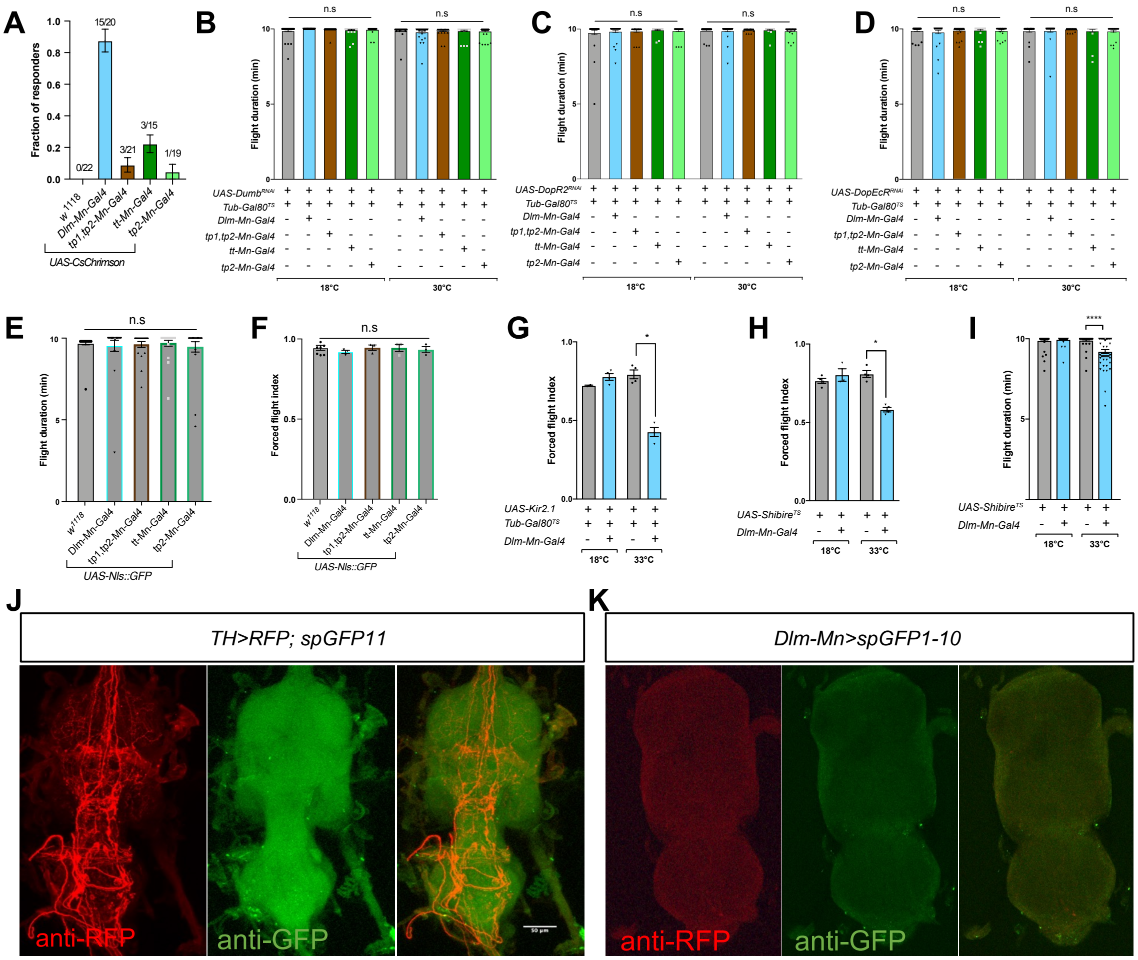

Fig. S6. A subset of flight motor neurons conveys TH neuron signals. Related to figure 5. (**A**) Red light-evoked activation of flight muscle motoneurons expressing CsChrimson. The graph shows the fraction of each fly population that responds to light. Note the magnitude of the effects caused by activation of Dlm neurons. (**B-D**) Evaluation of flight performance under conditional neuronal downregulation of different dopaminergic receptors: Dop1R1/Dumb (**B**), DopR2 (**C**), or DopEcR (**D**). (**E-F**) Flies that express GFP in distinct flight motoneurons do not show significant changes in flight duration (**E**) and average forced flight index (**F**) compared with control wt (w1118) flies. (**G**) Reduction in the activity of Dlm neurons via expression of Kir2.1 leads to a flight deficient phenotype. (**H-I**) Expression of *Shibire*^{TS} in Dlm neurons reduces average duration of flight and forced flight. (**J-K**) GRASP Fluorescence was not detected in *TH-LexA/LexAop-rCD2-RFP;LexAop-CD4-spGFP11* (**J**) and *Dlm-Gal4/lexAop2-CD4::*spGFP1-10 control VNCs (**K**). Error bars in figures represent SEM; n > 20. Significant values in all figures based on Mann-Whitney U test or one-way ANOVA with the post hoc Tukey-Kramer test: $*p < 0.05$, $****p < 0.0001$.

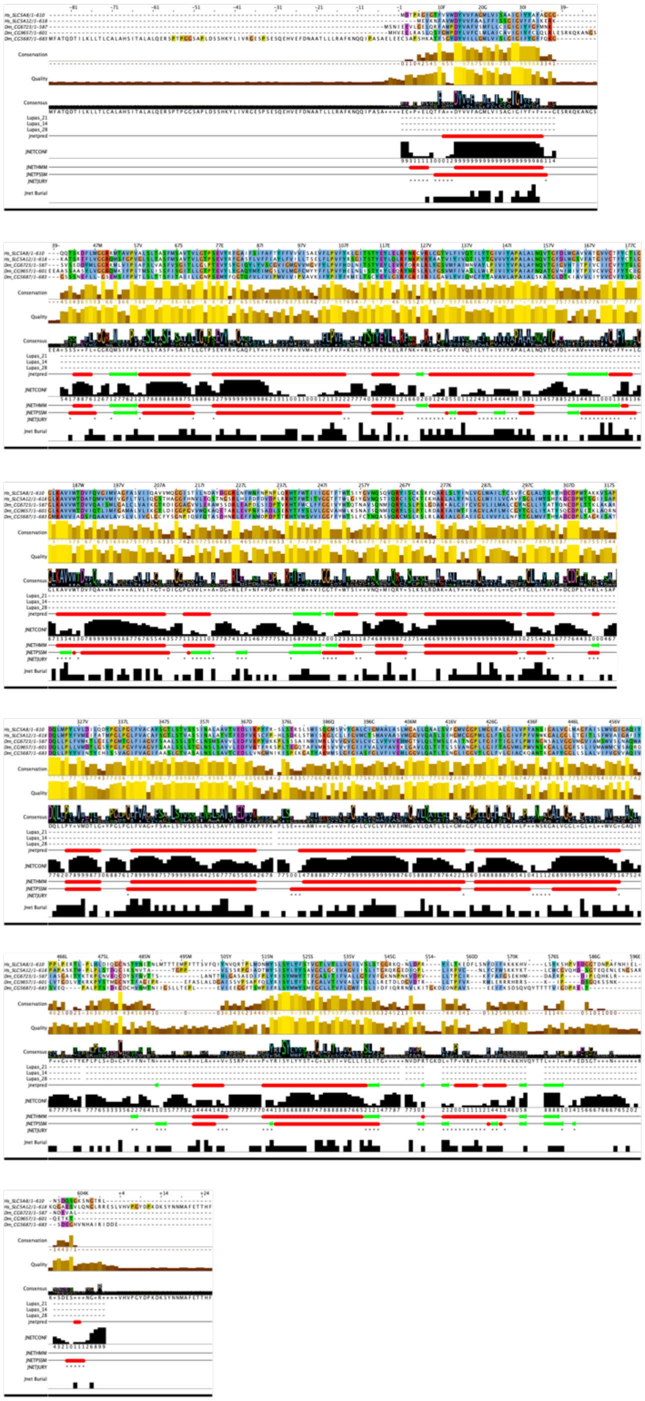

Fig. S7. Structural alignment of *Drosophila* (Dm) SLC genes orthologous to human (hs) SLC5A12 and SLC5A8 genes. Related to figure 6. (Top) Sequence alignment produced by the Jalview program of SLC genes from Drosophila and human. The colour code represents conservation of distinct aminoacid residues in the alignment. Blue:hydrophobic, red:positive charge, magenta:negative charge, green:polar, pink:cysteines, orange:glycines, yellow:prolines, cyan:aromatic. (Bottom) JPred-Jalview secondary structure prediction. Helices are marked as red tubes, and sheets as dark green arrows JNetPRED: consensus prediction. JNetCONF: confidence estimates for the prediction. High values mean high confidence. Lupas_21, Lupas_14, Lupas_28: Coiled-coil predictions for the sequence. Jnet Burial: Prediction of Solvent Accessibility. JNetALIGN: Alignment based prediction. JNetHMM: HMM profile-based prediction. JNETPSSM: PSSM based prediction. A '*' in this annotation indicates that JNETJURY was invoked to rationalise discrepant primary predictions.

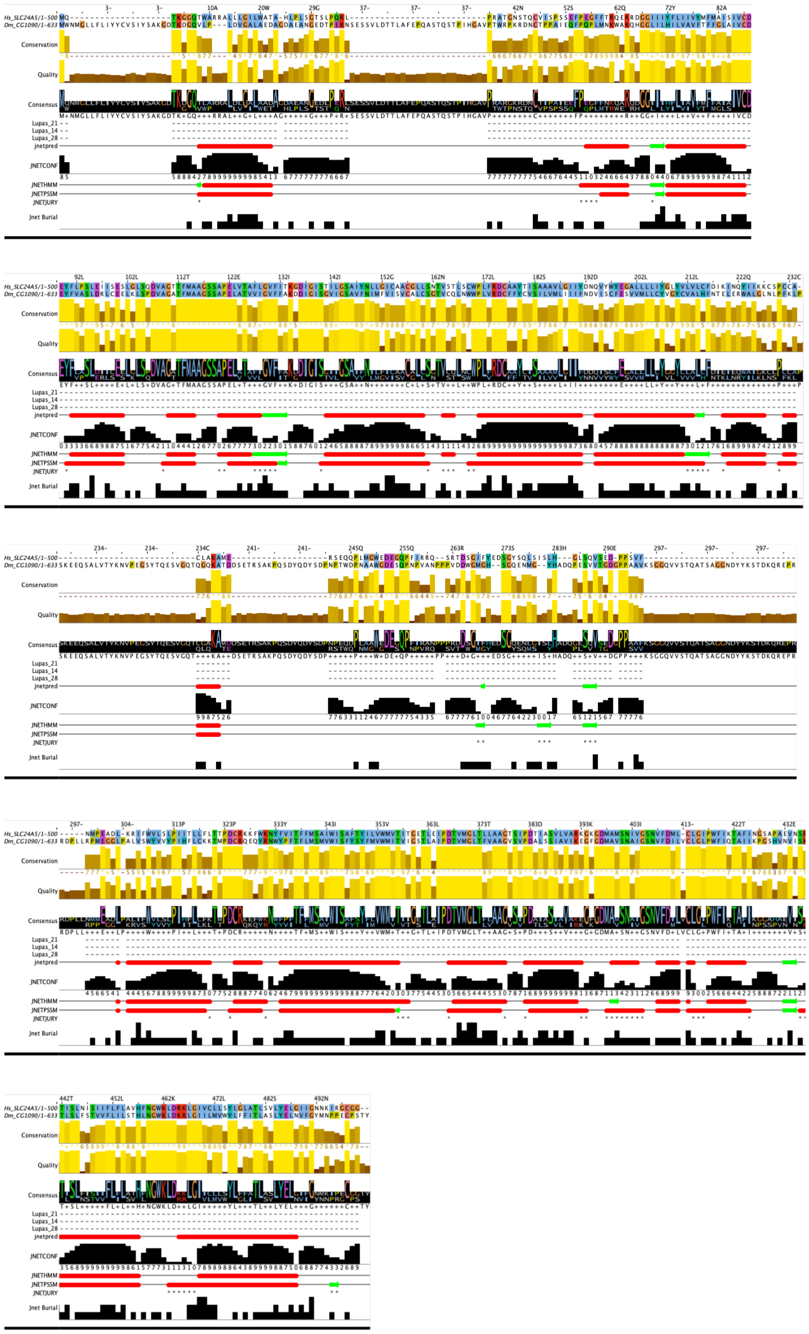

Fig. S8. Structural alignment of *Drosophila* (Dm) SLC genes orthologous to the human (hs) SLC24A5 gene. Related to figure 6. (Top) Sequence alignment produced by the Jalview program of SLC genes from Drosophila and human. The colour code represents conservation of distinct aminoacid residues in the alignment. Blue:hydrophobic, red:positive charge, magenta:negative charge, green:polar, pink:cysteines, orange:glycines, yellow:prolines, cyan:aromatic. (Bottom) JPred-Jalview secondary structure prediction. Helices are marked as red tubes, and sheets as dark green arrows JNetPRED: consensus prediction. JNetCONF: confidence estimates for the prediction. High values mean high confidence. Lupas_21, Lupas_14, Lupas_28: Coiled-coil predictions for the sequence. Jnet Burial: Prediction of Solvent Accessibility. JNetALIGN: Alignment based prediction. JNetHMM: HMM profile-based prediction. JNETPSSM: PSSM based prediction. A '*' in this annotation indicates that JNETJURY was invoked to rationalise discrepant primary predictions.

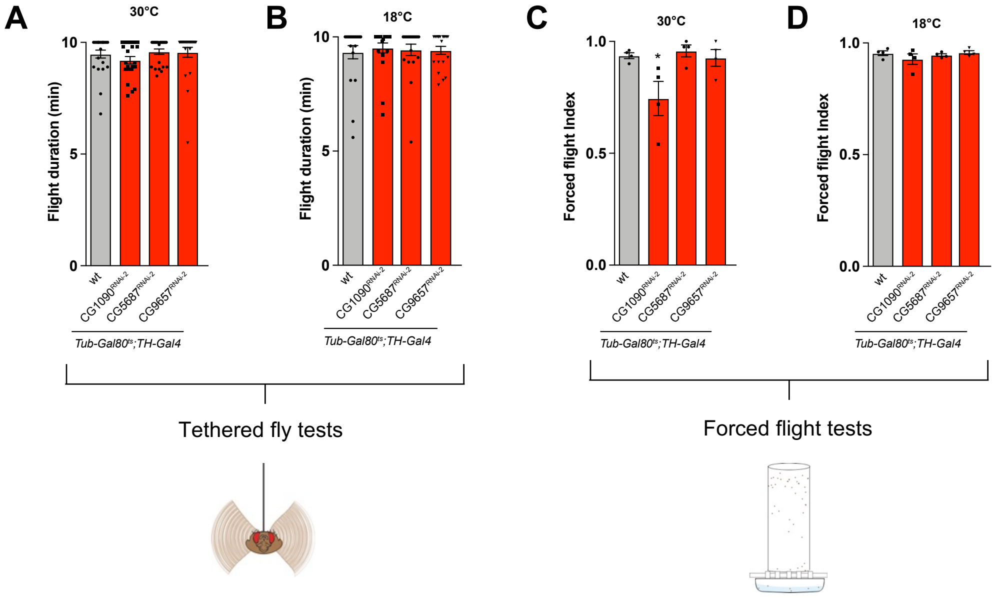

Fig. S9. Conditional downregulation of symporter genes in TH neurons affect flight behaviour. Related to figure 6. (**A-B**) Flight maintenance at restrictive (30°C) (**A**) and non-restrictive (18°C) (**B**) temperatures. (**C-D**) Forced flight at restrictive (30°C) (**C**) and non-restrictive (18°C) (**D**) temperatures.

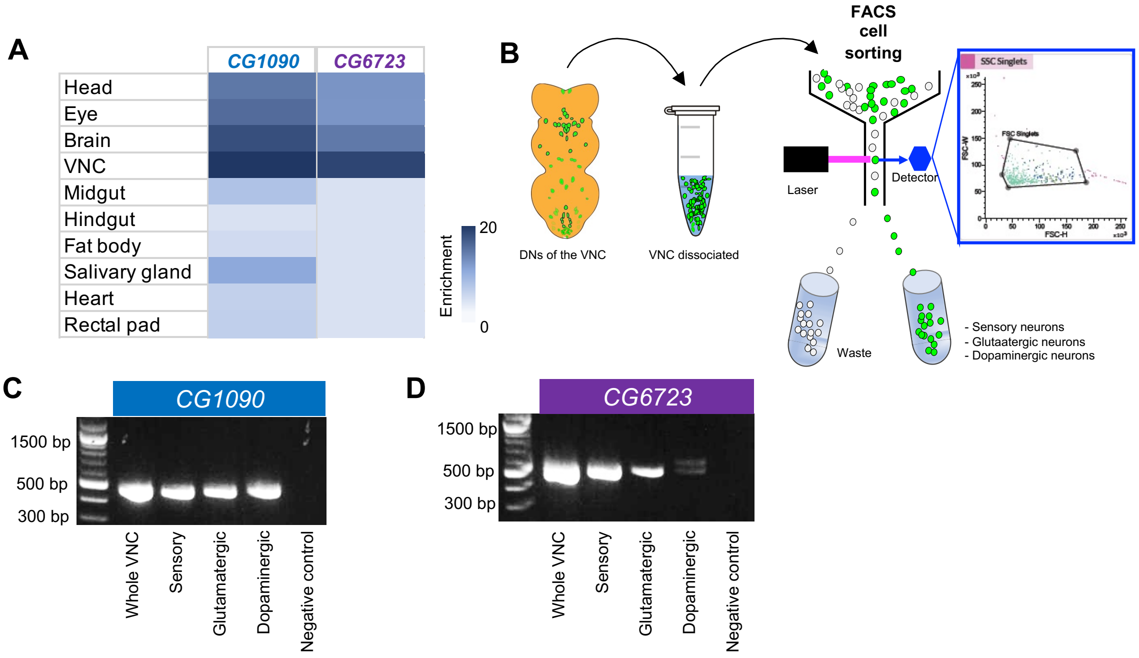

Fig. S10. Tissue specific expression of SLC genes in *Drosophila*. Related to figure 6. (**A**) Tissue distribution of *CG1090* and *CG6723* transcripts shows that both genes are highly expressed in the nervous system, especially in the VNC (FlyAtlas data). (**B-D**) Neuron-specific expression analysis of *CG1090* and *CG6723*. Schematic representation of the cell sorting FACS protocol used to collect different neuronal populations for PCR analysis, via neuron-specific expression of GFP using whole tissue (Whole VNC), *109(2)90-Gal4* (sensory neurons), *OK371-Gal4* (glutamatergic neurons) and *TH-Gal4* (dopaminergic neurons). PCR results show expression of *CG1090* (**C**) and *CG6723* (**D**) in all neuronal populations analysed, including sensory, glutamatergic and dopaminergic neurons. Whilst for *CG1090* we observe comparable and overall high levels of expression across all neural types analysed, in the case of *CG6723* we note that expression is high in sensory, moderate in glutamatergic and low in dopaminergic neurons (analysis of the latter also shows weak signal of a slightly larger, not fully processed transcript, which includes an extra 57-mer segment due to intron retention). The purity and identity of each specific neuronal population was confirmed by testing expression of *futsch*, *vGlut*, and *pale* (*ple*) genes for sensory, glutamatergic and dopaminergic neurons, respectively.

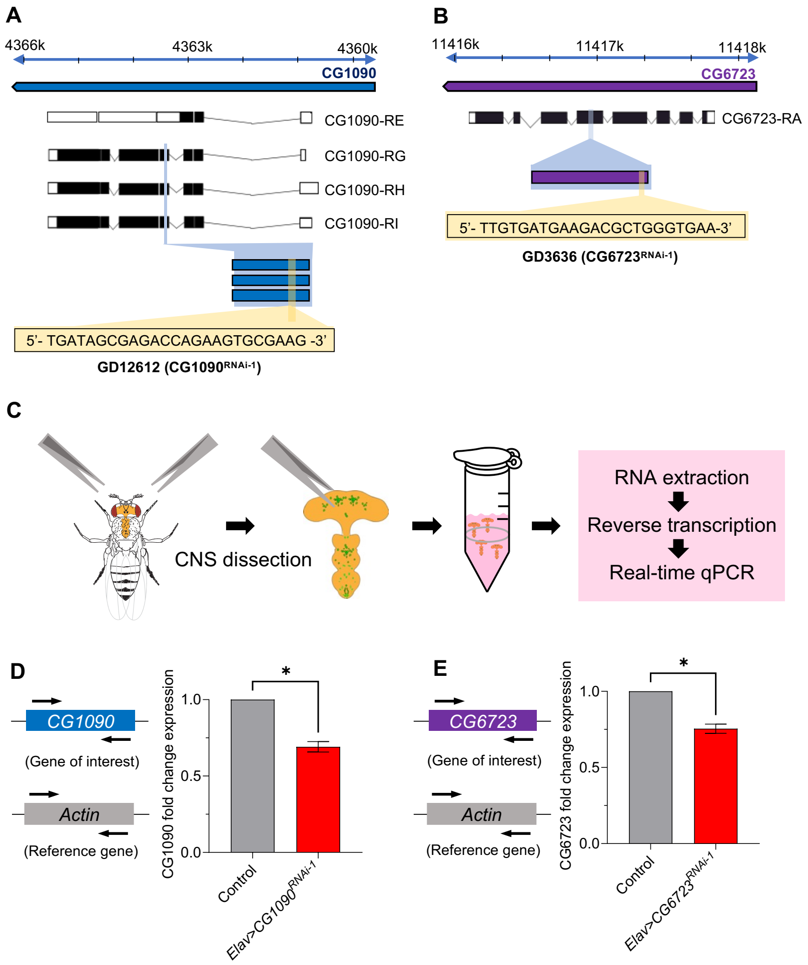

Fig. S11. Gene structure of *CG1090* and *CG6723* genes and functional validation of RNAi treatments. (**A-B**) Genomic loci targeted by RNA interference constructs against *CG1090* (**A**) and *CG6723* (**B**). (**C**) Experimental pipeline used to validate the action of gene-specific RNAi constructs. Manual dissection of adult CNS tissue, was followed by RNA extraction and qPCR expression analysis. (**D**) qPCR validation of *CG1090* downregulation in the experimental *Elav-Gal4; UAS-CG1090*$^{RNAi-1}$ condition compared to a control line (*Elav-Gal4*). Double delta Ct analysis was applied to qPCR data to calculate fold-change expression between conditions, using *actin* (*Act42A)* as a reference gene. (**E**) qPCR validation of *CG6723* downregulation in the experimental *Elav-Gal4; UAS-CG6723*$^{RNAi-1}$ condition, as done for *CG1090* in panel D. Error bars in figures represent SEM. Mann-Whitney U tests were used to determine significance $*p < 0.05$.

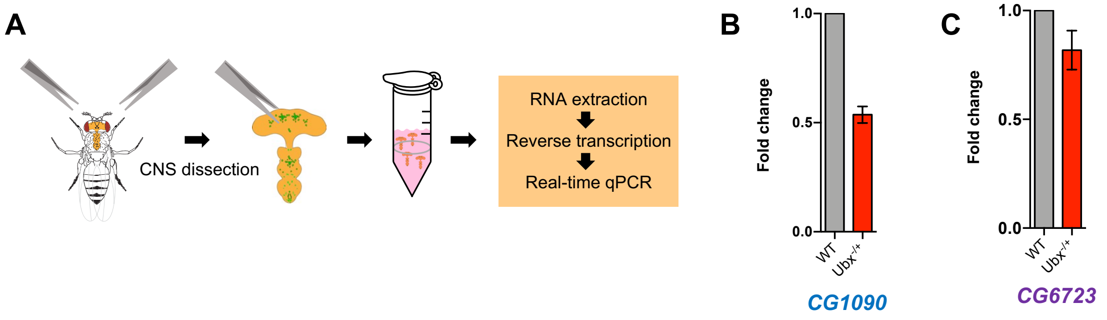

Fig. S12. Genetic reduction of Ubx input downregulates CG1090 and CG6723 genes. Related to figure 6. (**A**) pipeline used to obtain samples from tissue. (**B-C**) qPCR results revealing the expression levels of *CG1090* (**B**) and *CG6723* (**C**) in the VNC tissue samples of wild-type and heterozygous Ubx KO flies. This analysis shows that a decrease in *Ubx* expression leads to a substantial and modest downregulation of *CG1090* and *CG6723*, respectively.

LEGENDS FOR MOVIES

Movie 1 (separate file). Assessing flight in normal and post-developmentally reduced Ubx conditions. Related to Fig. 1. Movie showing a top-view of four individuals: two on the left kept at 30ºC (deactivated Gal80ts), and two on right kept at 18ºC (active Gal80ts). At each temperature condition, the individual on the left include a TH-Gal4 driver; all individuals include a *UAS-Ubx*^{RNAi} construct and a *Tub-Gal80ts* construct (labelled). Flight is triggered by a gentle air puff applied simultaneously to all individuals.

Movie 2 (separate file). Muscle activity during sustained flight in normal conditions. Related to Fig. 1. Fluorescence imaging of thoracic flight muscles (dorsal longitudinal muscles) expressing the calcium reporter GCaMP6m (green signal) in a normal fly during sustained flight.

Movie 3 (separate file). Muscle activity during sustained flight in post-developmentally reduced Ubx conditions. Related to Fig. 1. Fluorescence imaging of thoracic flight muscles (dorsal longitudinal muscles) expressing the calcium reporter GCaMP6m (green signal) in post-developmentally reduced Ubx conditions (*TH>Ubx*^{RNAi}) during sustained flight.

Movie 4 (separate file). Optogenetic activation of TH neurons triggers flight. Related to Fig. 3. Adult fly expressing CsChrimson in TH neurons (left) and a control fly (right). Optogenetic activation of CsChrimson by red light triggers flight only in the specimen on the left.

Movie 5 (separate file). Optogenetic inhibition of TH neurons reduces flight performance. Related to Fig. 3. Adult fly expressing GtACR in TH neurons (left) and a control fly (right). Optogenetic inhibition of GtACR by blue light leads to a reduction in wing beat frequency only in the specimen on the left.

Movie 6 (separate file). Optogenetic inhibition applied within the Ubx domain reduces flight performance. Related to Fig. 3. Adult fly expressing GtACR within the *Ubx* domain (left) and a control fly (right). Optogenetic inhibition of GtACR by blue light stops flight instantly.

Movie 7 (separate file). Optogenetic activation of Ubx^{+} TH neurons triggers flight. Related to Fig. 3. Adult fly expressing CsChrimson in Ubx^{+} TH neurons (intersection of Ubx and TH domains using the split-Gal4 approach) (left) and a control fly (right). Optogenetic activation of CsChrimson by red light triggers flight only in the specimen on the left.

Movie 8 (separate file). Optogenetic activation of Dlm motor neurons triggers flight. Related to Fig. 5. Adult fly expressing CsChrimson in Dlm motor neurons (left) and a control fly (right). Optogenetic activation of CsChrimson by red light triggers flight only in the specimen on the left.

Movie 9 (separate file). Optogenetic activation of TH neurons triggers activity in Dlm motor neurons. Related to Fig. 5. Fluorescence imaging of a fly expressing GCaMP6m in Dlm motor neurons projecting into the Dlm muscles. When TH neurons expressing CsChrimson are activated by red light, activity in the projections of Dlm motor neurons is triggered (green signal).

Movie 10 (separate file). Simple take-off response in normal wild type adult *Drosophila*. Related to figure 1. The movie shows a top- view of a take-off behavioural arena. Lifting the glass plate triggers an escape reflex, with most flies immediately taking-off from the arena, and swiftly moving away from the centre of the field of view (for quantifications see Figure S2).

Movie 11 (separate file). Simple take-off response in adult *Drosophila* with post-developmental downregulation of Ubx expression. Related to figure 1. The movie shows a top-view of a take-off behavioural arena. Lifting the glass plate triggers an escape reflex in normal flies; in contrast, flies

in which *Ubx* had been post-developmentally downregulated (*TH>UbxRNAi*) do not show this reflex, and remain close to centre of the arena. Note that even when prompted by a paintbrush, flies do not show a standard take-off response (for quantifications see Figure S2).

Movie 12 (separate file). Ubx expression in TH neurons. The movie displays the VNC of adult *Drosophila* showing expression of Ubx (red) in TH neurons within T2 and T3 VNC segments labelled by GFP driven by TH-Gal4.

Movie 13 (separate file). TH neuron patterns in the whole-VNC and brain. The movie displays whole-VNC and brain expression of TH-Gal4 driving Myr::GFP (green) and anti-nc82 neuropil counterstaining (magenta). This shows that TH neuron soma and projections/axons are located in the brain as well as in the VNC.

Movie 14 (separate file). Ubx cell patterns in the whole-VNC and brain. The movie displays whole-VNC and brain expression of Ubx-Gal4 driving Myr::GFP (green) and anti-nc82 neuropil counterstaining (magenta). This indicates that Ubx cell soma are exclusively located in the VNC T2 and T3 segments and send axons in the VNC as well brain.

Movie 15 (separate file). Ubx positive TH neurons in the whole-VNC. The movie displays whole-VNC expression of $Ubx^{Gal4.DBD} \cap ple^{Gal4.AD}$ (Ubx∩TH-Gal4) driving mcd8::GFP (green) and anti-nc82 neuropil counterstaining (magenta). This shows that Ubx^{+} TH neurons soma are exclusively located in the VNC T2 and T3 segments and have their axons around the neuromeres and the medial axis of VNC. This also demonstrates that Ubx^{+} TH neurons have their axons both in the VNC and brain.

LEGENDS FOR DATASETS

Dataset S1 (separate file). Transcriptomic analysis of normal and Ubx-downregulated TH neurons in the adult VNC. The transcriptome of TH neurons in the VNC of wild-type adult *Drosophila* flies (*TH-Gal4; UAS-GFP(II); UAS-GFP(II) – termed TH_GFP*) as compared with the transcriptome of VNC TH neurons expressing *UbxRNAi* exclusively in adults (*TH-Gal4; UAS-GFP(II); UAS-UbxRNAi(III) – termed TH_UbxRi*). A generalised linear model edgeR approach was used to make pairwise comparisons between groups, and adjusted for multiple testing via the FDR (Benjamini-Hochberg) approach used by edgeR. A table of genes is shown for each comparison with the following information in successive columns (A-K), as follows: (A) *FlyBase ID*; (B) *Gene name*; (C) *logFC* (which indicates the log (in base 2) difference between the two groups); (D) logCPM (log-average abundance across samples); (E) LR (Likelihood Ratio test); (F) PValue (p-value); (G) FDR (adjusted p-value for multiple testing); (H-K) Normalised log CPM values for each gene, in each sample (NB: comparisons have been performed without outlier pairing and low-features pairing samples).

Dataset S2 (separate file). Tissue- and stage-specific expression data on differentially regulated SCL5/24 symporter genes in VNC TH neurons with normal or reduced *Ubx* expression. Tissue- and stage-specific expression of SCL5/24 symporter genes extracted from the Fly Atlas database (http://flyatlas.gla.ac.uk/FlyAtlas2/index.html). Data includes information on *SCL5* symporter genes *CG9657* (sheet No. 1), *CG5687* (sheet No. 2) and *CG6723* (sheet No. 3), and *SCL24* symporter gene *CG1090* (sheet No. 4).

SI REFERENCES

1. L. de Navas, D. Foronda, M. Suzanne, E. Sánchez-Herrero, A simple and efficient method to identify replacements of P-lacZ by P-Gal4 lines allows obtaining Gal4 insertions in the bithorax complex of Drosophila. *Mech Dev* **123**, 860-867 (2006).
2. T. Riemensperger *et al.*, A single dopamine pathway underlies progressive locomotor deficits in a Drosophila model of Parkinson disease. *Cell Rep* **5**, 952-960 (2013).
3. A. O'Sullivan *et al.*, Multifunctional Wing Motor Control of Song and Flight. *Curr Biol* **28**, 2705-2717 e2704 (2018).
4. E. B. Lewis, Control of body segment differentiation in Drosophila by the bithorax gene complex. *Prog Clin Biol Res* **85 Pt A**, 269-288 (1982).
5. L. J. Macpherson *et al.*, Dynamic labelling of neural connections in multiple colours by trans-synaptic fluorescence complementation. *Nat Commun* **6**, 10024 (2015).
6. J. L. Pitman *et al.*, A pair of inhibitory neurons are required to sustain labile memory in the Drosophila mushroom body. *Curr Biol* **21**, 855-861 (2011).
7. J. A. Berry, I. Cervantes-Sandoval, M. Chakraborty, R. L. Davis, Sleep Facilitates Memory by Blocking Dopamine Neuron-Mediated Forgetting. *Cell* **161**, 1656-1667 (2015).
8. K. Ahmad *et al.*, Commonalities in Biological Pathways, Genetics, and Cellular Mechanism between Alzheimer Disease and Other Neurodegenerative Diseases: An In Silico-Updated Overview. *Curr Alzheimer Res* **14**, 1190-1197 (2017).
9. H. Inagaki *et al.*, Optogenetic control of Drosophila using a red-shifted channelrhodopsin reveals experience-dependent influences on courtship. *Nat Methods* **11**, 325-332 (2014).
10. T. Kitamoto, Conditional modification of behavior in Drosophila by targeted expression of a temperature-sensitive shibire allele in defined neurons. *J Neurobiol* **47**, 81-92 (2001).
11. T. Chen *et al.*, Ultrasensitive fluorescent proteins for imaging neuronal activity. *Nature* **499**, 295-300 (2013).
12. J. A. Bender, M. H. Dickinson, Visual stimulation of saccades in magnetically tethered Drosophila. *J Exp Biol* **209**, 3170-3182 (2006).
13. V. Haikala, M. Joesch, A. Borst, A. S. Mauss, Optogenetic control of fly optomotor responses. *J Neurosci* **33**, 13927-13934 (2013).
14. D. T. Babcock, B. Ganetzky, An improved method for accurate and rapid measurement of flight performance in Drosophila. *J Vis Exp* 10.3791/51223, e51223 (2014).
15. T. Rival *et al.*, Decreasing glutamate buffering capacity triggers oxidative stress and neuropil degeneration in the Drosophila brain. *Curr Biol* **14**, 599-605 (2004).

16. A. Issa *et al.*, The lysosomal membrane protein LAMP2A promotes autophagic flux and prevents SNCA-induced Parkinson disease-like symptoms in the Drosophila brain. *Autophagy* **14**, 1898-1910 (2018).
17. T. Baden *et al.*, The functional diversity of retinal ganglion cells in the mouse. *Nature* **529**, 345-350 (2016).
18. K. Masuyama, Y. Zhang, Y. Rao, J. Wang, Mapping neural circuits with activity-dependent nuclear import of a transcription factor. *J Neurogenet* **26**, 89-102 (2012).
19. M. K. DeSalvo *et al.*, The Drosophila surface glia transcriptome: evolutionary conserved blood-brain barrier processes. *Front Neurosci* **8**, 346 (2014).
20. A. M. Waterhouse, J. B. Procter, D. M. Martin, M. Clamp, G. J. Barton, Jalview Version 2--a multiple sequence alignment editor and analysis workbench. *Bioinformatics* **25**, 1189-1191 (2009).
21. A. Drozdetskiy, C. Cole, J. Procter, G. J. Barton, JPred4: a protein secondary structure prediction server. *Nucleic Acids Res* **43**, W389-394 (2015).
22. D. T. Jones, W. R. Taylor, J. M. Thornton, A model recognition approach to the prediction of all-helical membrane protein structure and topology. *Biochemistry* **33**, 3038-3049 (1994).
