# Supplementary material for: A novel post-developmental role of the Hox genes underlies normal adult behavior
Source: Proc Natl Acad Sci U S A. 2022 Dec 1;119(49):e2209531119. doi: 10.1073/pnas.2209531119 (PMC9894213; doi:10.1073/pnas.2209531119)
Supplement: Supplementary file 2 — Dataset S01 (PDF) [file pnas.2209531119.sd01.pdf]

FlyBase ID	GeneName	logFC	logCPM	LR	PValue	FDR
FBgn008535	CG34324	18.4833701	10.659439	73.9507091	8.01E-18	4.57E-14
FBgn002914	NtR	18.1796686	10.2583704	63.3538446	1.73E-15	4.93E-12
FBgn025989	NimC1	17.0601149	9.1129698	55.733067	8.30E-14	1.58E-10
FBgn003117	whe	16.9683371	9.03897832	53.2516377	2.93E-13	4.19E-10
FBgn002893	NimB5	16.4703777	8.57860415	46.1688095	1.08E-11	1.24E-08
FBgn008524	CG34220	14.1863684	12.0065163	44.2084765	2.95E-11	2.81E-08
FBgn001403	Sr-CI	16.0904701	8.18943786	41.5506427	1.15E-10	9.37E-08
FBgn003658	CG4950	16.1102612	8.24578327	40.4165397	2.05E-10	1.46E-07
FBgn026162	CG42711	16.1448622	8.30690464	38.148808	6.55E-10	4.16E-07
FBgn002858	lcs	13.1785236	10.0612093	37.1216743	1.11E-09	6.33E-07
FBgn003620	Muc68D	15.7077028	7.81012908	36.8586954	1.27E-09	6.45E-07
FBgn003507	Ance-5	13.9259512	8.08938615	36.7309819	1.36E-09	6.45E-07
FBgn003993	fd102C	15.5439936	7.73062194	34.6063392	4.04E-09	1.77E-06
FBgn002758	CG4757	15.6588252	7.72797087	34.2455099	4.86E-09	1.98E-06
FBgn003835	CG5399	15.6207366	7.65843177	33.3081445	7.87E-09	2.99E-06
FBgn004157	AttC	15.5813178	7.70171121	32.8173139	1.01E-08	3.61E-06
FBgn002839	TotA	15.4163946	7.46289377	29.2585989	6.33E-08	2.02E-05
FBgn003132	CG5397	15.3890517	7.58580845	29.2459827	6.37E-08	2.02E-05
FBgn003756	CG11672	15.3879781	7.4373512	29.0876837	6.92E-08	2.08E-05
FBgn005132	CG31326	12.7436149	7.8655248	28.6583157	8.63E-08	2.46E-05
FBgn004009	lectin-28C	9.48664674	9.05956908	28.3597637	1.01E-07	2.74E-05
FBgn003250	CG16826	7.61570837	9.85885126	27.1320563	1.90E-07	4.93E-05
FBgn003191	CG5958	15.1502176	7.39485787	25.4718533	4.49E-07	0.0001114
FBgn003487	CG3906	12.2309229	11.3477253	25.0519676	5.58E-07	0.00013273
FBgn001503	Cyp9b2	15.0917025	7.14628293	24.432043	7.70E-07	0.00017576
FBgn003439	MFS15	15.0370422	7.30327044	23.9766609	9.75E-07	0.00021407
FBgn028346	Drs	7.45909398	8.91320205	23.7400073	1.10E-06	0.00023311
FBgn003476	CG4250	8.21622273	11.2926306	22.8522474	1.75E-06	0.00035664
FBgn001401	Rel	11.6612768	7.56048146	22.5755173	2.02E-06	0.00039768
FBgn001501	Ag5r	13.1457448	7.24923208	22.5058807	2.10E-06	0.00039861
FBgn004006	yip7	14.9091696	7.07690383	22.1604031	2.51E-06	0.00046178
FBgn003131	CG5080	8.66465062	9.62561033	21.7882671	3.04E-06	0.00054307
FBgn004039	CG14629	9.07916361	9.1109189	21.294747	3.94E-06	0.00068117
FBgn005310	CG33109	14.728629	7.09585105	20.1311838	7.23E-06	0.00119137
FBgn003412	jtb	14.7038148	7.02833769	20.0981267	7.36E-06	0.00119137
FBgn001376	Crz	14.5477992	6.94103763	20.0577365	7.51E-06	0.00119137
FBgn005121	CG31211	-14.854339	6.90008075	19.8806233	8.24E-06	0.00127167
FBgn000388	betaTub60D	11.2618234	9.0878364	19.7495601	8.83E-06	0.00132609
FBgn003506	Eps-15	-9.8677294	7.36993963	18.6343531	1.58E-05	0.00231747
FBgn004010	lectin-24Db	12.7957309	7.01798976	18.3870907	1.80E-05	0.00257253
FBgn003891	fit	12.9515058	6.91556594	18.1385038	2.05E-05	0.00278679
FBgn003623	CG14125	14.5978589	6.76418481	18.1376988	2.05E-05	0.00278679
FBgn026645	CG45080	6.71497781	8.93128593	18.0969531	2.10E-05	0.00278679
FBgn003336	Ance-4	14.5827589	6.82808677	18.0165754	2.19E-05	0.0028409
FBgn003033	Karl	10.3081356	7.29544198	17.2292239	3.31E-05	0.00420237

FBgn026669	Svil	7.55002148	7.78219944	16.3469732	5.27E-05	0.00654439
FBgn003127	CG13947	14.3496961	6.68832148	16.0705295	6.10E-05	0.00729206
FBgn003173	CG11030	-12.603206	6.83889875	16.0614173	6.13E-05	0.00729206
FBgn000403	y	14.4577946	6.53101744	15.2222388	9.56E-05	0.01113308
FBgn000029	Col4a1	7.98354803	8.02900311	14.980591	0.00010862	0.01240037
FBgn002849	CG3530	-11.926608	6.80273152	14.919249	0.00011221	0.01255896
FBgn003036	regucalcin	5.21234528	11.0010585	14.0492027	0.00017809	0.01954873
FBgn026383	Gen	14.1445464	6.42475468	13.5284064	0.00023498	0.02530685
FBgn001592	crq	8.21692196	8.11513475	13.4323927	0.00024732	0.02614227
FBgn003433	CG15067	5.03056921	8.60789453	13.3101607	0.00026397	0.02729448
FBgn003636	CG10725	14.0071807	6.46435643	13.2833022	0.00026778	0.02729448
FBgn000294	ninaE	14.2427399	6.28618559	13.1165392	0.0002927	0.0293111
FBgn025081	Jon65Aiv	13.95233	6.47035776	13.0555263	0.00030239	0.02975943
FBgn003853	AttD	14.1346712	6.34313585	12.991402	0.00031293	0.02979538
FBgn003014	Gga	14.154143	6.32383151	12.9897783	0.0003132	0.02979538
FBgn028442	Psn	-12.371325	6.55267045	12.9090687	0.00032699	0.03059806
FBgn002976	CG15784	14.1167836	6.30725462	12.814721	0.0003439	0.03136718
FBgn003340	CG1968	14.2009124	6.26974705	12.8022435	0.0003462	0.03136718
FBgn003682	CG3902	9.09990153	7.12598266	12.6358967	0.00037841	0.03346281
FBgn008519	CG34166	10.5218817	7.12577267	12.6228564	0.00038106	0.03346281
FBgn003562	mthl2	14.1447519	6.21679911	12.404514	0.0004283	0.03653929
FBgn003277	fon	14.1530627	6.20587973	12.3918513	0.00043121	0.03653929
FBgn002995	CG9657	-11.542763	6.60061799	12.3738248	0.0004354	0.03653929
FBgn002983	Tsp5D	-9.4688458	7.13988287	12.346991	0.0004417	0.03653929
FBgn003722	CG1092	6.61887369	8.53482519	12.1647377	0.00048702	0.03971261
FBgn005120	CG31205	14.0541222	6.17547231	12.1265128	0.0004971	0.03996403
FBgn000007	Amy-p	14.100297	6.16262896	12.058121	0.00051567	0.04088136
FBgn003220	Hand	13.8477578	6.27533737	12.0269563	0.00052437	0.04100114
FBgn002568	Amnionless	13.8506991	6.26031593	11.9643638	0.00054228	0.04166662
FBgn003875	CG4783	14.097307	6.14342589	11.9398122	0.00054947	0.04166662
FBgn003766	beag	-12.071944	6.54266895	11.9219081	0.00055478	0.04166662
FBgn000442	LysP	13.7851526	6.27838334	11.897027	0.00056224	0.04167849
FBgn002351	eIF2Bepsilon	-13.994734	6.17374052	11.5767545	0.00066781	0.04887028
FBgn003738	CG1213	7.90826913	7.11253002	11.4631661	0.00070989	0.05129198
FBgn002910	alpha-Catr	-14.077201	6.13155497	11.4289615	0.00072308	0.05159182
FBgn003004	CG15347	13.9142303	6.10224315	11.3068247	0.00077223	0.05441819
FBgn008536	CG34331	13.6797245	6.17839016	11.2632734	0.00079056	0.05503047
FBgn002643	Grip75	13.9391214	6.07771506	11.2172829	0.00081039	0.05573144
FBgn000004	Act42A	4.13671329	9.57238364	11.1267499	0.00085092	0.05782174
FBgn026247	CG43066	-8.8003929	7.22671249	10.9247607	0.00094888	0.0636389
FBgn003348	egr	13.7507291	6.07434061	10.9054449	0.00095882	0.0636389
FBgn003456	CG15651	-13.974655	6.02570153	10.8493593	0.0009883	0.06441418
FBgn026489	CG44085	9.49421157	6.80669446	10.8404411	0.00099307	0.06441418
FBgn004060	CG3348	10.920589	6.51382878	10.8086357	0.00101028	0.06463471
FBgn003926	Smg6	-11.616083	6.41245341	10.7870627	0.00102212	0.06463471
FBgn003967	Obp99a	13.5543152	6.14099789	10.7720535	0.00103044	0.06463471

FBgn003588	CG7120	13.4780043	6.16026497	10.6208527	0.0011182	0.06926303
FBgn003122	CG4822	10.5729183	6.55971375	10.6038945	0.0011285	0.06926303
FBgn002436	CG11417	12.2602748	6.21314396	10.5139844	0.00118475	0.07194177
FBgn003973	Tace	-13.911526	5.96548505	10.4487657	0.00122731	0.07374222
FBgn002898	Spn42Dd	13.8578632	5.90573798	10.2737648	0.00134935	0.07969482
FBgn003177	Fbw5	11.5113045	6.3427197	10.2583959	0.00136064	0.07969482
FBgn001964	AANAT1	-13.889797	5.93833468	10.2480776	0.00136827	0.07969482
FBgn004357	PGRP-SB1	13.5351165	5.97577656	10.2022118	0.00140272	0.08034756
FBgn000451	Gad1	-3.6600857	10.8389161	10.1957713	0.00140763	0.08034756
FBgn008524	CG34215	6.75219673	7.20866588	10.1489924	0.0014438	0.08159622
FBgn002991	CG4557	-8.3319672	6.77793954	10.1222683	0.00146489	0.08197617
FBgn002909	ced-6	4.23861738	8.54658517	10.1010142	0.00148188	0.08212191
FBgn003523	Pcyt2	-8.730193	7.20737852	10.0472937	0.00152572	0.08341242
FBgn026288	CG43236	13.3587067	6.08222088	10.0368532	0.00153439	0.08341242
FBgn004481	TotC	13.6836024	5.86690406	9.90527783	0.00164805	0.08874618
FBgn003827	CG7265	-13.838328	5.88740365	9.86867468	0.00168117	0.08922672
FBgn026093	scny	10.5312754	6.43509411	9.86094674	0.00168824	0.08922672
FBgn003705	ICA69	-13.800171	5.86698953	9.81086349	0.00173484	0.09084834
FBgn003676	CG5506	13.7198939	5.7782376	9.75244103	0.00179085	0.09292871
FBgn005400	NimB3	13.5258865	5.89645646	9.69122622	0.0018515	0.09521044
FBgn002854	NimB4	13.6549546	5.75581005	9.57451171	0.00197297	0.10035854
FBgn000335	sei	-11.948468	6.12956931	9.5617137	0.00198678	0.10035854
FBgn003384	bbc	-7.3401366	8.83432846	9.53102626	0.00202027	0.10102292
FBgn003400	CG8155	-13.440686	5.93696483	9.48036687	0.00207682	0.10102292
FBgn000394	Ubx	4.12386	8.47080252	9.47797664	0.00207953	0.10102292
FBgn003249	CG5945	12.1376348	6.10033295	9.47684143	0.00208082	0.10102292
FBgn003684	Sfxn2	-13.696942	5.78118811	9.45948996	0.0021006	0.10102292
FBgn025971	CG42369	8.07372025	7.12068011	9.45467247	0.00210612	0.10102292
FBgn003566	Jon65Aiii	13.5971255	5.72751689	9.42715432	0.00213796	0.10169553
FBgn003248	CG16812	-11.946024	6.09441411	9.3950206	0.00217576	0.10263812
FBgn003073	CG9915	-13.691976	5.74078379	9.34892371	0.00223117	0.10437776
FBgn006790	IM14	6.39131126	7.20734843	9.33277937	0.00225091	0.10437776
FBgn003076	CG13012	13.33706	5.80079511	9.31312712	0.00227518	0.10437776
FBgn002041	Idgf1	6.91521147	7.0042956	9.30461138	0.00228578	0.10437776
FBgn006349	GstE9	12.1467438	6.01381084	9.23107225	0.00237942	0.10707599
FBgn003470	MED16	-13.606612	5.74414583	9.21957744	0.00239441	0.10707599
FBgn004171	yellow-f	13.4676031	5.69277099	9.20341016	0.00241565	0.10707599
FBgn003128	CG3862	-13.653961	5.70279055	9.20018828	0.0024199	0.10707599
FBgn003805	CG5961	-13.64493	5.69432886	9.17018478	0.0024599	0.10800842
FBgn003398	CG7639	-13.489813	5.7207764	9.08325883	0.0025796	0.11239965
FBgn003590	CG6765	-13.609721	5.65828423	9.05634382	0.00261785	0.11320215
FBgn003233	CG16854	-13.575154	5.62520368	8.95562039	0.00276617	0.11871663
FBgn003516	Gr61a	-13.470731	5.61406768	8.82640791	0.00296902	0.12647149
FBgn003378	CG13323	13.4242344	5.478449	8.79890232	0.00301412	0.12677915
FBgn002578	Cdc16	13.1754852	5.69684444	8.79494317	0.00302067	0.12677915
FBgn003414	Lst	13.3750386	5.44028636	8.69258705	0.00319507	0.13279739

FBgn003335	spab	-4.2462282	9.60772727	8.68375473	0.00321059	0.13279739
FBgn002637	Rep	13.2714195	5.49599819	8.64852955	0.00327325	0.13441536
FBgn026517	PIG-V	13.2628303	5.42000741	8.57302134	0.00341181	0.13910417
FBgn003785	CG14695	13.3043576	5.37553182	8.54023515	0.00347381	0.14062761
FBgn005044	Opbp	-13.328877	5.5148663	8.52411711	0.00350471	0.14087936
FBgn003422	CG4847	13.2014752	5.43988111	8.49690785	0.00355751	0.14114675
FBgn003430	CG5742	-13.415358	5.46437699	8.49084744	0.00356938	0.14114675
FBgn004077	CG17977	-13.387072	5.43679534	8.46318435	0.00362407	0.14114675
FBgn003515	CG13894	-13.363327	5.42334377	8.46314981	0.00362414	0.14114675
FBgn002757	Nep2	-13.370725	5.42579881	8.4577069	0.003635	0.14114675
FBgn003948	CG5880	-13.361873	5.41882049	8.43982185	0.00367092	0.14157854
FBgn003586	CG13671	-13.241182	5.5615651	8.39983656	0.00375255	0.14300395
FBgn000424	DptA	13.1072247	5.4547565	8.38168671	0.00379021	0.14300395
FBgn004081	Nplp2	3.28119294	10.7441074	8.37419952	0.00380585	0.14300395
FBgn003458	CG9394	-7.0879212	9.4950177	8.37312908	0.00380809	0.14300395
FBgn003789	CG6723	-10.412346	6.4851425	8.31155378	0.00393937	0.14696674
FBgn002352	CG3191	-13.276857	5.33670187	8.24352541	0.00408976	0.15127206
FBgn001377	Cp1	3.35245009	11.4285211	8.23554963	0.00410777	0.15127206
FBgn003854	CG7379	-13.245801	5.3127176	8.2018446	0.00418478	0.15198318
FBgn003259	CG17904	7.69256014	7.65089489	8.20159338	0.00418536	0.15198318
FBgn003529	CG5687	-13.26463	5.32857347	8.19223376	0.00420701	0.15198318
FBgn002572	alphaCOP	-9.9257818	6.33098962	8.18080843	0.00423359	0.15198318
FBgn003873	CG11407	-13.17931	5.32046554	8.13832078	0.00433394	0.15436823
FBgn003035	Sclp	12.9371267	5.41366393	8.12990014	0.00435412	0.15436823
FBgn003902	Nepl15	-8.9266677	6.94543887	8.05698981	0.00453284	0.15971277
FBgn000111	Glt	8.74312967	6.99056976	7.97313738	0.00474766	0.16625542
FBgn003763	CCT7	4.14726295	7.98605146	7.95203992	0.00480332	0.16717899
FBgn003532	CG13806	12.9877931	5.20111649	7.93626732	0.00484537	0.16762037
FBgn008399	CG34155	-11.66496	5.80473828	7.87738537	0.00500568	0.17112695
FBgn001160	ine	-12.948533	5.27485306	7.8762798	0.00500874	0.17112695
FBgn028443	CCT8	-12.955634	5.31552539	7.86622383	0.00503667	0.17112695
FBgn002786	Nup107	-13.075733	5.20688715	7.83915699	0.00511264	0.17268032
FBgn005038	CG30380	12.6674263	5.39186116	7.77550908	0.00529593	0.17610888
FBgn001204	AttA	12.7961244	5.22725104	7.77458342	0.00529864	0.17610888
FBgn003683	CG14075	12.9969647	5.11917942	7.77183391	0.00530672	0.17610888
FBgn003656	ClC-c	8.90995627	6.7155055	7.73508274	0.00541581	0.17840217
FBgn003279	Hasp	-4.5624952	9.31794432	7.72759029	0.00543833	0.17840217
FBgn003212	CG17855	12.6875569	5.25835769	7.67928013	0.00558585	0.18164543
FBgn003224	Wdfy2	6.65026153	8.39933472	7.67444223	0.00560084	0.18164543
FBgn003350	CG12913	-13.097758	5.1479616	7.65660848	0.00565647	0.18241329
FBgn003933	alrm	-7.0474148	7.93235028	7.64274628	0.00570011	0.18278769
FBgn003737	CG1236	-13.081115	5.13477748	7.61581458	0.00578586	0.18450111
FBgn000330	ry	-12.885076	5.20076644	7.59921226	0.00583938	0.18490481
FBgn005349	CG33494	8.60672236	6.73023997	7.5776036	0.0059098	0.18490481
FBgn003601	CG3335	-13.029896	5.13618362	7.56021625	0.00596708	0.18490481
FBgn003490	CG5543	10.5480996	5.95755012	7.55370337	0.00598869	0.18490481

FBgn003702	Pex14	8.35267959	7.27650259	7.55249735	0.0059927	0.18490481
FBgn008524	CG34212	8.86574955	8.22898913	7.55244057	0.00599289	0.18490481
FBgn005177	CG31777	11.5730673	5.64603618	7.54128062	0.00603012	0.18505326
FBgn006503	Arpc3B	13.0248876	5.07331124	7.50442809	0.00615475	0.18786793
FBgn004586	btz	-13.004661	5.11267099	7.47847539	0.00624409	0.18958127
FBgn003382	CG10799	7.80759805	7.02732329	7.46788472	0.00628093	0.18969075
FBgn005270	Cubn	5.35640941	7.43203643	7.44861612	0.00634852	0.19072303
FBgn001150	SrpRbeta	-11.558569	5.69601396	7.37935424	0.00659769	0.19717062
FBgn003723	CG1090	-12.727502	5.1824456	7.36419749	0.00665353	0.1976157
FBgn003473	CG4610	-12.607768	5.32563355	7.35656824	0.00668182	0.1976157
FBgn003509	Nplp1	-8.1104777	7.64526367	7.3416347	0.00673755	0.19823691
FBgn005349	CG33493	11.5164793	5.57871877	7.32546296	0.00679845	0.19892254
FBgn003095	CG6891	11.0772227	6.06709778	7.31057121	0.00685501	0.19892254
FBgn003683	CG3819	12.85312	4.99150499	7.30728442	0.00686756	0.19892254
FBgn000311	pn	-12.993722	5.0699227	7.29875092	0.00690026	0.19892254
FBgn026308	CG43351	12.5700509	5.13782661	7.28897497	0.00693791	0.19900282
FBgn003469	Plekhm1	-9.3219746	6.17642888	7.25615792	0.00706583	0.20059123
FBgn003704	CG10584	-12.890374	5.09285349	7.25563846	0.00706787	0.20059123
FBgn002708	LeuRS-m	-11.512031	5.60175352	7.241742	0.00712278	0.20059123
FBgn002896	gammaCOP	7.56426035	7.47510966	7.23895457	0.00713385	0.20059123
FBgn002960	CG3091	10.658771	5.83847005	7.21963732	0.00721103	0.20176738
FBgn001576	Msr-110	-3.9556563	8.05688788	7.20656968	0.00726372	0.20225029
FBgn003829	Spn88Eb	12.6511792	5.07425865	7.16002871	0.0074546	0.20655757
FBgn000012	Arr1	12.8565524	4.93533665	7.1468418	0.00750961	0.20707662
FBgn026045	CG4806	-12.755811	5.0907574	7.1378328	0.00754743	0.20711894
FBgn000007	Amy-d	12.8481614	4.8991927	7.0121428	0.00809587	0.22110642
FBgn005135	CG31357	-12.828229	4.9516469	6.98004098	0.00824237	0.22403552
FBgn003201	CG7806	-12.788908	4.98248729	6.95097557	0.00837735	0.22559213
FBgn003727	CG1129	-12.877442	4.93209734	6.95069136	0.00837869	0.22559213
FBgn003516	Dci	10.1475751	5.92400729	6.92369182	0.00850612	0.22794802
FBgn001169	EbpIII	4.58078258	7.45110588	6.91218343	0.00856104	0.2283477
FBgn005363	CG33635	12.8126297	4.8627048	6.90252955	0.00860739	0.22851614
FBgn003493	CG3803	-12.790813	4.94342728	6.89140103	0.00866114	0.22887854
FBgn005016	Brca2	-12.718016	4.96766407	6.86262598	0.00880171	0.23152149
FBgn003170	TotM	12.4109044	5.01064131	6.83535749	0.00893708	0.23400393
FBgn001605	Sema1b	6.06611598	7.2598311	6.80637939	0.00908328	0.23585846
FBgn002950	Tsp42Ed	3.26409523	8.79134735	6.80494993	0.00909055	0.23585846
FBgn003181	Rchy1	-12.769147	4.91667113	6.77826916	0.00922743	0.23832663
FBgn003788	GCC88	-11.2899	5.54363218	6.74698567	0.00939062	0.24144892
FBgn002758	CG5867	9.86090711	6.13792183	6.72839811	0.00948898	0.24288379
FBgn003164	hoe2	-11.2908	5.4556924	6.70142145	0.00963361	0.24548498
FBgn003725	Fip1	-12.770364	4.87486412	6.69008312	0.00969507	0.24595312
FBgn002843	Ercc1	12.6154573	4.80107163	6.61599926	0.01010666	0.25470514
FBgn003755	Ada2b	-12.785506	4.83687432	6.612014	0.0101293	0.25470514
FBgn003052	CtsB1	2.9724992	10.5540848	6.60309588	0.01018016	0.25486122
FBgn002563	CG13366	-11.065356	5.29023525	6.59006984	0.01025492	0.25561159

FBgn003736	CG1239	-12.747518	4.82200454	6.56440068	0.01040388	0.25714229
FBgn005281	CG32815	-12.336336	4.97315322	6.563965	0.01040642	0.25714229
FBgn003201	CG8086	8.29735044	6.48214604	6.5005027	0.0107844	0.26514866
FBgn002762	Acf	-10.811028	5.5200084	6.49409429	0.01082334	0.26514866
FBgn002847	Nab2	-12.621287	4.82663056	6.45657213	0.01105427	0.26964859
FBgn003433	GstE1	3.2334067	9.58976772	6.39306621	0.0114567	0.2774163
FBgn003369	EndoG	-12.438016	4.87226785	6.38549743	0.01150565	0.2774163
FBgn003301	CG10395	-11.117579	5.22774762	6.376032	0.01156718	0.2774163
FBgn026376	CG43680	12.1335581	4.89691334	6.35285691	0.01171924	0.2774163
FBgn002041	Idgf2	3.37804349	8.94831689	6.35225026	0.01172325	0.2774163
FBgn003858	Non3	-12.66897	4.75502426	6.35208139	0.01172437	0.2774163
FBgn000040	Cyt-b5-r	7.94649057	6.98273703	6.35137052	0.01172907	0.2774163
FBgn003034	ATP7	12.482927	4.73088553	6.34646888	0.01176152	0.2774163
FBgn004069	Teh3	-12.661223	4.71970934	6.31234203	0.01199004	0.27867845
FBgn003305	CCHa2-R	12.6149327	4.66800356	6.30912919	0.01201178	0.27867845
FBgn003390	eIF3m	-7.6009223	7.58508549	6.30542122	0.01203693	0.27867845
FBgn003549	CG14989	-3.4598485	10.9543827	6.3010323	0.01206677	0.27867845
FBgn003637	Abp1	-12.548157	4.78290594	6.29651454	0.01209756	0.27867845
FBgn003922	CG5805	-12.644267	4.72999467	6.29499093	0.01210796	0.27867845
FBgn003111	CG1812	-12.626605	4.73769276	6.28544516	0.01217335	0.27905815
FBgn003342	CG1888	-10.057369	5.4560678	6.27707848	0.01223096	0.27925722
FBgn000363	su(w[a])	-8.3180606	6.26492507	6.22986429	0.01256134	0.2856578
FBgn003956	CG4849	-10.004866	5.41806864	6.21583449	0.01266127	0.28601782
FBgn003807	Gnmt	12.3380582	4.71282886	6.20721989	0.01272303	0.28601782
FBgn002795	MBD-like	-12.575643	4.70658521	6.20659906	0.01272749	0.28601782
FBgn005119	CG31199	10.8522874	5.21263985	6.14291301	0.01319398	0.29533815
FBgn003153	Snx1	7.51837099	7.05277729	6.13486315	0.01325418	0.29539068
FBgn003825	CG3505	12.3442608	4.68276984	6.1287848	0.01329983	0.29539068
FBgn003722	CG9766	-11.001262	5.23549565	6.10230946	0.01350053	0.29776752
FBgn008625	CG6084	2.89022664	9.75335909	6.10091703	0.01351118	0.29776752
FBgn026157	CoRest	-12.438724	4.70720993	6.0926004	0.01357492	0.2980216
FBgn004345	CG12084	-9.6070141	5.52508428	6.03301806	0.01404071	0.30706648
FBgn002640	NiPp1	-12.234409	4.76458321	6.00639358	0.01425413	0.30895816
FBgn026628	jnj	12.4295009	4.5402686	6.00390671	0.01427424	0.30895816
FBgn002539	Scgdelta	-10.106538	5.46927117	5.99596271	0.01433865	0.30895816
FBgn001566	Dref	-11.038016	5.12445726	5.99373718	0.01435676	0.30895816
FBgn003141	CG3609	3.61859557	8.48506454	5.98071142	0.01446317	0.30895816
FBgn026319	Acn	-5.7586649	7.45607006	5.97693922	0.01449414	0.30895816
FBgn003164	Mon1	-11.032892	5.10047455	5.97548466	0.01450609	0.30895816
FBgn002838	fal	-12.53193	4.58511969	5.95978924	0.01463579	0.31056168
FBgn001343	Cdc2rk	-10.43752	5.22196776	5.94689121	0.01474326	0.31098903
FBgn005405	CG34054	11.9780973	4.7200068	5.94181581	0.01478578	0.31098903
FBgn003030	HP5	7.73624861	6.85208488	5.93617969	0.01483314	0.31098903
FBgn003503	CG3548	-12.461035	4.62757987	5.93134774	0.01487386	0.31098903
FBgn000050	Dsk	12.3037677	4.39847149	5.89559144	0.01517883	0.31580187
FBgn026212	Sec24CD	8.00641253	6.34752924	5.89143457	0.0152147	0.31580187

FBgn002752	goe	-10.872005	5.20371698	5.87527016	0.01535502	0.31662478
FBgn026376	CG43679	12.1982363	4.5586632	5.87409286	0.01536529	0.31662478
FBgn003698	CG13248	-6.5549292	7.62422217	5.86403554	0.01545332	0.3168913
FBgn003811	CG8031	-12.394341	4.57796814	5.85994751	0.01548926	0.3168913
FBgn008612	snama	10.20558	5.24025098	5.8502966	0.01557443	0.3174958
FBgn002090	Rtc1	-12.4688	4.52565136	5.83819706	0.01568189	0.31854881
FBgn003598	mRRF1	-12.382254	4.48059921	5.80469657	0.01598343	0.32242285
FBgn003215	Etl1	-10.255573	5.54503027	5.80446003	0.01598558	0.32242285
FBgn002869	Rpn5	5.21831971	7.30881188	5.77916578	0.01621724	0.32494254
FBgn003207	Argl	12.1722413	4.39064657	5.77767889	0.01623096	0.32494254
FBgn001999	Gcn2	-12.371481	4.53043005	5.77223839	0.01628128	0.32494254
FBgn002499	Ugalt	-10.998112	5.06137831	5.75810392	0.01641277	0.32642549
FBgn026198	CG42807	8.83381168	9.29205065	5.73987561	0.01658397	0.32824389
FBgn003760	CG8036	9.00405251	5.68760434	5.73614644	0.01661922	0.32824389
FBgn003123	CG3645	-12.244413	4.48360879	5.69741574	0.01698991	0.33440831
FBgn005236	tut	10.6468822	5.06362257	5.64935661	0.01746178	0.34169615
FBgn003831	CG14864	-10.479951	5.45296945	5.6475376	0.0174799	0.34169615
FBgn003597	PGRP-LF	11.9195581	4.48617065	5.63565424	0.01759878	0.34202173
FBgn008613	Dbp21E2	-12.2396	4.37409867	5.63389995	0.0176164	0.34202173
FBgn002038	Dredd	-12.202457	4.38034919	5.62263268	0.01773	0.34306041
FBgn002760	CG8611	-12.049768	4.61549334	5.60552806	0.0179039	0.34357413
FBgn000455	Akh	12.1856431	4.24742881	5.59666036	0.01799475	0.34357413
FBgn003612	CG6310	8.3689316	6.61759476	5.59546815	0.018007	0.34357413
FBgn001022	Gel	3.24632309	8.83971423	5.59276225	0.01803483	0.34357413
FBgn028597	prg	-12.191935	4.35824208	5.5905617	0.01805751	0.34357413
FBgn003894	rdhB	5.62847329	7.36495327	5.58216312	0.0181443	0.34407859
FBgn003495	Pask	-11.994384	4.60582065	5.55388029	0.01843976	0.34716139
FBgn002431	Plap	-12.240278	4.32048507	5.54981773	0.01848261	0.34716139
FBgn003514	BORCS6	-12.130645	4.3574688	5.54149323	0.01857073	0.34716139
FBgn002030	drongo	5.49534454	7.06553169	5.53361474	0.01865453	0.34716139
FBgn003846	Irc	4.53909933	7.75617342	5.53037128	0.01868914	0.34716139
FBgn003234	CG6201	-10.465702	5.15188978	5.52665126	0.01872892	0.34716139
FBgn003697	Rbbp5	-10.861158	5.07188377	5.52630686	0.01873261	0.34716139
FBgn003538	Or63a	-12.217307	4.30717165	5.50996322	0.01890845	0.34865439
FBgn000278	Mp20	10.6443293	5.0149745	5.50747821	0.01893533	0.34865439
FBgn008531	CG34288	10.7312335	4.90560587	5.49865785	0.01903108	0.3492906
FBgn003263	SPH93	11.8055055	4.36368129	5.48928874	0.01913333	0.34945515
FBgn003292	Coq3	-12.22	4.28353193	5.48662637	0.01916249	0.34945515
FBgn003417	ste24a	-9.9422962	5.17215602	5.47513338	0.01928889	0.35064005
FBgn003248	CG16972	-10.50436	5.0212591	5.46751138	0.01937319	0.35105456
FBgn002971	CG15912	-11.887655	4.52848498	5.44798383	0.01959092	0.35113181
FBgn003399	CG10265	-12.164082	4.2272964	5.43195722	0.01977151	0.35113181
FBgn003768	CG9356	-10.839238	4.92855748	5.42520357	0.01984812	0.35113181
FBgn005173	AspRS-m	-11.91234	4.35956958	5.41855524	0.01992384	0.35113181
FBgn005110	CG31103	-12.113409	4.29570704	5.41756326	0.01993516	0.35113181
FBgn003281	CG13077	10.9180131	4.8185026	5.40653879	0.02006146	0.35113181

FBgn001399	Nrx-IV	-5.4746861	6.71028861	5.40604071	0.02006718	0.35113181
FBgn003337	Mys45A	-9.7367502	5.27417547	5.4010506	0.02012463	0.35113181
FBgn002988	pigs	4.44444651	7.1195235	5.39924054	0.02014552	0.35113181
FBgn003771	CG9393	-12.155392	4.251114	5.3986696	0.02015211	0.35113181
FBgn025078	vari	-10.825789	4.91315852	5.39331974	0.02021398	0.35113181
FBgn003885	CG5745	-12.142758	4.20676009	5.39170581	0.02023268	0.35113181
FBgn001038	Dro	8.4055712	7.89368189	5.38702649	0.02028701	0.35113181
FBgn000286	MtnB	11.7966582	4.30524945	5.38354124	0.02032758	0.35113181
FBgn001486	Mtk	7.74097382	7.48059045	5.38274734	0.02033683	0.35113181
FBgn003353	Git	-12.093899	4.27971166	5.38061425	0.02036171	0.35113181
FBgn001607	vkg	3.46229818	7.77634046	5.36118371	0.02058979	0.35399561
FBgn003222	CG5022	-10.028227	5.30353593	5.35017625	0.02072018	0.35516746
FBgn001089	sinu	-12.041269	4.24653516	5.32688764	0.02099885	0.35738034
FBgn003912	CG13601	-11.971864	4.29802768	5.32498667	0.02102177	0.35738034
FBgn003931	CG11892	-12.116047	4.17254104	5.32123995	0.02106702	0.35738034
FBgn008634	ALiX	-6.040395	7.88877527	5.31453039	0.02114829	0.35738034
FBgn002745	Dnz1	10.9036751	4.77850768	5.31337468	0.02116233	0.35738034
FBgn001128	Obp19d	12.0039534	4.07060403	5.30715704	0.02123799	0.35760007
FBgn002563	CG17829	-10.820719	4.9183392	5.30143831	0.02130782	0.35772074
FBgn002039	Nmt	-10.175122	5.64855831	5.29205555	0.02142292	0.35859831
FBgn004006	yip2	3.83089094	7.78166286	5.28012006	0.02157026	0.36000891
FBgn003161	HP6	11.9444975	4.07391078	5.27452003	0.02163975	0.36011578
FBgn003448	Hacl	-8.3155745	6.135803	5.26457979	0.02176368	0.36077043
FBgn003103	CG14220	-10.308657	4.99532966	5.25667022	0.02186281	0.36077043
FBgn003872	Nup58	-11.944796	4.20594412	5.25620091	0.02186871	0.36077043
FBgn003141	CG3597	11.9581825	4.05480691	5.24793506	0.02197283	0.36144358
FBgn004622	Wdr33	-12.061065	4.12338981	5.23407856	0.02214854	0.36293371
FBgn003849	beat-IIa	-7.6871882	6.87436444	5.22856075	0.02221891	0.36293371
FBgn003086	CG8173	-11.923365	4.19145513	5.22383028	0.02227943	0.36293371
FBgn002751	ana2	-11.978167	4.15514313	5.22084119	0.02231775	0.36293371
FBgn025971	DIP-epsilon	-10.691107	4.81205354	5.18862935	0.02273514	0.36680839
FBgn026200	CG42821	11.8448604	3.95082111	5.17786057	0.02287648	0.36680839
FBgn003228	CG17107	11.6835425	4.10716975	5.17672906	0.02289138	0.36680839
FBgn003583	ldbr	-11.899278	4.16683851	5.17593293	0.02290187	0.36680839
FBgn005138	CG31388	-12.001122	4.11992441	5.17313248	0.02293882	0.36680839
FBgn003188	MME1	-12.015423	4.07726036	5.17048995	0.02297374	0.36680839
FBgn003630	CG10973	-11.958489	4.1233893	5.16806321	0.02300585	0.36680839
FBgn001532	Ubc2	-8.7089031	5.3225976	5.15935318	0.02312151	0.36762552
FBgn003950	CG3368	-8.363247	5.67456046	5.14569076	0.02330414	0.36883496
FBgn003594	ValRS-m	-9.7882986	5.13644202	5.14400252	0.02332681	0.36883496
FBgn003264	mEFTs	-8.1027007	6.38938002	5.12099818	0.02363802	0.37206904
FBgn003367	rho-7	-11.919533	4.07188009	5.11507233	0.02371888	0.37206904
FBgn003968	Obp99b	11.7509251	4.05859932	5.11448573	0.0237269	0.37206904
FBgn003876	PIG-L	-10.370735	5.01793484	5.10084389	0.02391421	0.37397887
FBgn003814	GILT1	11.7599376	3.85054683	5.09391403	0.02400994	0.37445015
FBgn003011	CG17754	-9.8505764	5.00268392	5.07453951	0.02427972	0.37712841

FBgn003855	Alg1	-11.864587	4.07749143	5.07106674	0.02432841	0.37712841
FBgn003620	CG10361	-11.884149	4.07337494	5.06740283	0.02437989	0.37712841
FBgn000052	e	-9.8582937	5.11181447	5.04076468	0.02475756	0.37895395
FBgn005176	CG31769	10.1805542	5.21606977	5.04027119	0.02476461	0.37895395
FBgn003454	Fem-1	-7.527947	7.16128714	5.03194072	0.024884	0.37895395
FBgn003074	CG9992	-11.815732	4.0731561	5.03136034	0.02489234	0.37895395
FBgn003322	CG1882	-11.878878	3.9364327	5.03025157	0.02490828	0.37895395
FBgn001010	mAcon1	-7.4535146	7.75935291	5.02250468	0.02501995	0.37895395
FBgn003096	CG7322	9.87133823	5.13555549	5.02211242	0.02502562	0.37895395
FBgn005350	CG33506	-11.860464	3.96909653	5.02187731	0.02502902	0.37895395
FBgn005005	Tmem18	-11.810689	4.04948749	5.01086533	0.02518871	0.3790125
FBgn003758	CG7352	-9.8021792	5.05009077	5.00911626	0.02521418	0.3790125
FBgn003197	Tg	-10.707383	4.74818359	5.00788698	0.02523209	0.3790125
FBgn000351	Sry-delta	9.08737412	5.76416571	4.99591584	0.0254072	0.38055815
FBgn003396	CG10151	-9.0947325	5.81830475	4.99175761	0.02546833	0.38055815
FBgn003602	CG8336	-10.673723	4.84517509	4.98080529	0.02563005	0.38197474
FBgn003118	CG14613	-11.784497	3.96452864	4.9700795	0.02578946	0.38332643
FBgn002051	Paics	4.71431387	6.96465546	4.96568592	0.02585506	0.38332643
FBgn003701	Pex16	-11.785527	3.88484612	4.95422973	0.02602692	0.38487472
FBgn003266	CG15152	11.4860501	3.96263257	4.94327686	0.02619234	0.38632002
FBgn002917	sotv	-8.606013	5.2243695	4.92545425	0.02646384	0.38931861
FBgn003492	Upf3	-11.732452	3.84060972	4.91702737	0.02659324	0.39021642
FBgn026076	CG42566	11.5101937	3.78071152	4.90892162	0.02671832	0.39022579
FBgn003614	CG14141	-4.9509388	8.06660479	4.90812754	0.0267306	0.39022579
FBgn005218	Ccn	-10.617571	4.81256555	4.88547877	0.02708352	0.39222762
FBgn001966	lncRNA:roX1	-6.8203538	9.953207	4.88406763	0.02710567	0.39222762
FBgn003842	ema	-7.8384249	5.69952	4.88371957	0.02711114	0.39222762
FBgn003041	Aven	-11.681181	3.74731921	4.88171799	0.02714259	0.39222762
FBgn003866	Smu1	-11.627789	3.87305744	4.86821756	0.02735574	0.39430953
FBgn002352	CG3071	-11.618922	3.67832696	4.85614066	0.02754789	0.39516856
FBgn000027	CecA1	11.3757661	3.65333468	4.85291111	0.02759951	0.39516856
FBgn005224	CG32243	10.5849848	4.88323095	4.85144202	0.02762303	0.39516856
FBgn003368	CG8407	-11.564102	3.62686917	4.835815	0.02787446	0.39764143
FBgn006509	RNaseMRP:F	11.3423841	3.51480316	4.83206108	0.02793522	0.39764143
FBgn006296	lncRNA:CR33	11.307273	3.59449749	4.82516589	0.02804717	0.39796963
FBgn026498	lncRNA:CR44	10.0652856	4.84419273	4.81911324	0.02814582	0.39796963
FBgn000114	GstD1	2.56611598	9.50867732	4.81711996	0.02817839	0.39796963
FBgn003911	PTPMT1	5.42591647	6.56701489	4.8101537	0.02829252	0.39796963
FBgn003515	CG3386	-11.553953	3.74541137	4.80927942	0.02830688	0.39796963
FBgn003195	r2d2	-11.518241	3.58679046	4.80065127	0.02844898	0.39837758
FBgn000004	Act57B	10.2163133	4.94429185	4.79904711	0.02847548	0.39837758
FBgn003400	CG8152	-11.512896	3.69047501	4.78212225	0.02875666	0.39973085
FBgn003902	CG17141	-11.47624	3.53872187	4.77697519	0.02884274	0.39973085
FBgn003618	RIOK1	-10.526608	4.64254285	4.77435298	0.0288867	0.39973085
FBgn003447	Obp56d	10.0655471	4.75909162	4.7739275	0.02889384	0.39973085
FBgn000071	FMRFa	-3.6786351	7.78601264	4.76639635	0.02902051	0.39973085

FBgn003033	CG1578	5.99908116	6.62338566	4.76563782	0.0290333	0.39973085
FBgn002732	CCT6	-8.4743554	5.73988166	4.75981862	0.02913162	0.39973085
FBgn002869	Rh50	-4.9449624	7.24257501	4.75976936	0.02913245	0.39973085
FBgn003756	CG9630	11.0761176	3.54533208	4.75530389	0.02920814	0.39980824
FBgn002546	slv	-11.435074	3.5740916	4.74711312	0.02934749	0.39991376
FBgn003615	CG6175	-11.454065	3.78000954	4.74426866	0.02939605	0.39991376
FBgn003954	CROT	-11.428712	3.50689593	4.73007875	0.02963953	0.39991376
FBgn003458	Cht9	11.1024892	3.17353982	4.72281121	0.02976504	0.39991376
FBgn003244	CCT4	7.9862644	6.79441148	4.72274846	0.02976613	0.39991376
FBgn003102	CG12204	-11.402454	3.64872295	4.72224923	0.02977477	0.39991376
FBgn003892	Idh3b	-6.1982144	8.62207984	4.72026869	0.02980909	0.39991376
FBgn003181	CG9527	-10.488487	4.62613536	4.7190842	0.02982963	0.39991376
FBgn000202	Catsup	5.98886327	6.57046227	4.71811746	0.0298464	0.39991376
FBgn003619	crim	-11.39966	3.87143553	4.70366005	0.03009846	0.40229376
FBgn003675	CG5577	-11.352279	3.56167362	4.69809668	0.03019604	0.40229376
FBgn026251	Vha44	-4.3425865	8.20110359	4.69209687	0.03030165	0.40229376
FBgn003229	CG12299	-8.7708378	5.08016673	4.69185337	0.03030594	0.40229376
FBgn003266	CG5758	-4.2527872	7.70888496	4.6709104	0.03067766	0.40436859
FBgn026125	rgn	-5.7818781	6.43701462	4.67038593	0.03068703	0.40436859
FBgn026782	da	-6.828156	6.00471119	4.66986491	0.03069634	0.40436859
FBgn003128	PGAP2	-11.344571	3.75657858	4.66628622	0.03076038	0.40436859
FBgn002795	MTA1-like	6.89009937	6.73104094	4.66315829	0.03081646	0.40436859
FBgn002490	Taf7	-10.47037	4.56077201	4.64627508	0.03112101	0.40677475
FBgn000342	sIgA	-7.1964526	8.33274638	4.64482998	0.03114722	0.40677475
FBgn003773	side-VII	-9.8194296	5.105092	4.64057653	0.03122451	0.40677475
FBgn003983	CG1750	-11.213097	3.63913683	4.63726168	0.03128488	0.40677475
FBgn002538	Rab27	-10.000372	4.75742072	4.62646969	0.03148228	0.40841102
FBgn005109	LpR2	3.62383561	7.42645945	4.60822408	0.03181897	0.40962599
FBgn003109	CG9581	-10.52582	4.55504929	4.60615913	0.03185731	0.40962599
FBgn003554	Syx17	-10.539096	4.56391966	4.60598813	0.03186049	0.40962599
FBgn003366	CG13185	10.1518879	4.84928966	4.6058535	0.03186299	0.40962599
FBgn005029	RIC-3	-8.8800169	5.53289385	4.59835834	0.03200259	0.40977355
FBgn002619	lncRNA:noe	-1.6100339	16.0761827	4.59753044	0.03201805	0.40977355
FBgn003599	CG3529	-7.3307863	7.1884722	4.5783011	0.0323793	0.41157479
FBgn002026	pcm	-9.5444951	4.9896902	4.57679744	0.03240773	0.41157479
FBgn003723	CG1103	-11.149256	3.21427724	4.57531607	0.03243576	0.41157479
FBgn003614	Plod	10.0853085	4.64966129	4.57038528	0.03252925	0.41157479
FBgn003357	CG13229	-10.409482	4.78604215	4.56854621	0.03256419	0.41157479
FBgn003382	CG4716	2.89481764	8.09472757	4.5655654	0.03262091	0.41157479
FBgn005145	pch2	-10.417595	4.52931991	4.55758142	0.03277332	0.41157479
FBgn003388	CG6701	8.01582261	6.10730904	4.55652552	0.03279354	0.41157479
FBgn004073	CG15068	8.70421639	5.61950641	4.55578448	0.03280773	0.41157479
FBgn000556	sv	-10.343481	4.5387784	4.54490735	0.03301681	0.41328935
FBgn003641	CG13482	10.8347895	2.90860134	4.53740266	0.03316187	0.41419688
FBgn003145	aph-1	-11.065302	3.14266434	4.52512339	0.03340067	0.41527044
FBgn026748	Ptp61F	3.82010787	7.530742	4.52262342	0.0334495	0.41527044

FBgn026293	Rabex-5	7.78414186	6.70899955	4.52177557	0.03346608	0.41527044
FBgn003289	twit	-7.3648032	6.79891564	4.50865523	0.03372375	0.41755995
FBgn002848	CG17841	-6.5516958	7.03751456	4.49988636	0.03389711	0.41879801
FBgn000336	sev	-9.028527	5.71755427	4.48402472	0.03421306	0.42059878
FBgn003729	plh	-11.003092	3.11853325	4.48194054	0.03425481	0.42059878
FBgn002558	IM2	8.55897697	5.81276647	4.48148605	0.03426392	0.42059878
FBgn003088	CG6847	-9.7602501	5.33181178	4.44945447	0.0349124	0.42763941
FBgn003585	CG8005	-10.425694	4.47094644	4.43624649	0.03518352	0.43003754
FBgn026773	lncRNA:CR46	-10.099618	4.69694935	4.42880818	0.03533718	0.43099272
FBgn005155	CG31550	-9.109233	5.54139274	4.42225588	0.03547311	0.43172816
FBgn003070	Lrp4	-3.9031934	7.15483249	4.4082787	0.03576491	0.4343492
FBgn003865	EndoA	-5.0274552	7.4954739	4.40467006	0.03584066	0.4343492
FBgn026744	lncRNA:CR45	-10.832155	3.00947853	4.39275641	0.03609192	0.43558669
FBgn003567	CG13295	-10.85243	2.92207676	4.39259222	0.03609539	0.43558669
FBgn008537	CG34348	-10.365667	4.394681	4.38426902	0.03627204	0.43679497
FBgn003633	AdenoK	-10.061747	4.71901471	4.36199457	0.03674925	0.4408069
FBgn003313	Tsp42Ep	10.5058538	2.67298666	4.36052497	0.03678097	0.4408069
FBgn003900	wfs1	-9.5472828	4.70750529	4.35793759	0.03683688	0.4408069
FBgn004209	CG13773	8.69049133	5.85302626	4.34185129	0.03718647	0.44361266
FBgn026138	Gpa2	-10.767585	2.93017555	4.33836633	0.03726266	0.44361266
FBgn003424	Dcr-2	5.2050393	6.6395599	4.33645594	0.0373045	0.44361266
FBgn001401	Rac2	3.6353389	7.37367064	4.33165042	0.03740996	0.44394184
FBgn002588	Inos	3.05175657	11.7155348	4.32454563	0.03756644	0.44402856
FBgn003543	Drsl5	10.4633938	4.22677126	4.32425563	0.03757285	0.44402856
FBgn003650	CG7275	-9.4066021	5.1874501	4.30974936	0.03789454	0.44690508
FBgn002887	CG15270	-8.5135495	5.16964863	4.29993571	0.03811381	0.44856423
FBgn000027	CecA2	10.4284475	2.53680256	4.28754567	0.03839255	0.45091496
FBgn003927	PQBP1	-9.0512946	4.94509445	4.26482123	0.03890934	0.45571558
FBgn003411	Atg9	7.14367335	6.75315462	4.26256862	0.03896097	0.45571558
FBgn003960	CG1523	-9.0441187	4.94308546	4.24714374	0.0393164	0.4588591
FBgn000005	Gart	-10.540937	2.72382637	4.23790778	0.03953085	0.4588591
FBgn003915	GatB	-9.9706552	4.570322	4.23736195	0.03954357	0.4588591
FBgn002499	Spg7	-10.183801	4.70693675	4.23703102	0.03955128	0.4588591
FBgn003326	Cul4	-7.7147406	5.29400167	4.227401	0.03977631	0.46053377
FBgn003086	CG8188	-6.9767445	7.40523234	4.21364197	0.04010015	0.46260002
FBgn003214	CG13117	7.65442177	5.925102	4.21293554	0.04011686	0.46260002
FBgn003377	CG17574	3.07530153	8.5501633	4.19505215	0.04054211	0.46656118
FBgn004034	CG3703	-7.535293	6.89986444	4.18042027	0.04089356	0.46837665
FBgn005222	Pex23	-8.3105935	5.50637158	4.18013761	0.04090038	0.46837665
FBgn002302	CRMP	-9.2492281	5.30246497	4.17824703	0.04094603	0.46837665
FBgn026610	CG44837	6.75902221	6.77867151	4.16870476	0.04117727	0.46939446
FBgn003734	CG2926	-8.631795	4.83432353	4.16779114	0.04119948	0.46939446
FBgn000459	pros	-2.4156742	9.9398417	4.14625963	0.0417266	0.47445305
FBgn003873	CG4662	-8.1776531	6.10986906	4.1297953	0.04213445	0.47813802
FBgn025998	Mppe	-10.074916	4.18602678	4.1140874	0.04252746	0.4816404
FBgn003359	Listericin	8.18513795	6.24080595	4.10121375	0.04285244	0.48336038

FBgn003547(	Ccz1	-9.9505316	4.59414304	4.09874807	0.04291498	0.48336038
FBgn003375	CG8818	-7.3298068	7.43489436	4.09507488	0.04300833	0.48336038
FBgn002586	Cortactin	5.00175025	7.23051196	4.09458082	0.0430209	0.48336038
FBgn003059	CG9512	5.70090786	6.54427224	4.09136815	0.04310274	0.48336038
FBgn003970	Atg16	-9.3433716	4.77836164	4.08016108	0.04338952	0.48474541
FBgn028622	Fum1	-6.9227163	7.54195707	4.07767711	0.04345336	0.48474541
FBgn005264	Coq8	-8.8647022	5.23198665	4.07660196	0.04348102	0.48474541
FBgn002846	CG11070	-9.3705724	4.93056993	4.05746328	0.04397652	0.48906315
FBgn003931	CG11893	-10.232583	2.34592946	4.05504005	0.04403967	0.48906315
FBgn025978	pzg	-8.7170245	5.66229166	4.03722725	0.04450689	0.4932919
FBgn004073	CG15065	7.81777461	5.35873983	4.029735	0.04470496	0.49422717
FBgn003905	Rassf	-8.9784048	4.80343829	4.02749189	0.04476444	0.49422717
FBgn000125	Ldh	7.88835046	6.12948229	4.01024052	0.0452247	0.49834475
FBgn003713	P5CDh1	-6.1353196	8.69457092	3.98743007	0.04584093	0.50416192
FBgn001042	TfIIFbeta	-9.7790481	4.15989819	3.97965447	0.04605301	0.50498746
FBgn003842	CG14882	-9.8783748	4.56963702	3.97537139	0.04617027	0.50498746
FBgn026377	CG43693	-7.301101	5.69542337	3.97496533	0.0461814	0.50498746
FBgn002065	ArfGAP1	-10.07764	4.13385865	3.96106469	0.04656424	0.50820019
FBgn003987	sip3	-8.6869163	4.76504616	3.93546074	0.04727819	0.5143693
FBgn003874	Arc42	5.62241237	6.61342104	3.93146867	0.04739054	0.5143693
FBgn003347	CG12128	8.42895006	5.87413995	3.93113887	0.04739983	0.5143693
FBgn000492	eIF2beta	2.69657726	8.21252652	3.90850873	0.04804221	0.52001214
FBgn003991	CG1909	-2.9321931	7.9770653	3.90641745	0.04810203	0.52001214
FBgn001083	l(3)04053	-7.8722503	4.97863279	3.89259861	0.04849933	0.52331599
FBgn026646	Nsf2	4.11210997	7.77958451	3.8863171	0.04868107	0.52342434
FBgn003048	CG1662	6.59051949	6.63666881	3.88591343	0.04869277	0.52342434
FBgn000485	elB	-9.7407628	4.37492405	3.87270813	0.04907727	0.52559786
FBgn001552	nonA-l	-9.4452456	4.62779675	3.86867585	0.04919532	0.52559786
FBgn003543	ZnT63C	6.64111945	6.00080935	3.86715654	0.04923988	0.52559786
FBgn003761	CG8159	-9.9139339	4.0163274	3.86386734	0.04933648	0.52559786
FBgn003240	Ced-12	7.26767124	6.53015948	3.86217544	0.04938626	0.52559786
FBgn003292	CG9248	-9.7617086	4.44711004	3.86009763	0.04944745	0.52559786
FBgn002899	sds22	6.65761604	7.19863434	3.85373261	0.04963541	0.52588159
FBgn004622	CG17230	-9.3382775	4.57830738	3.85080635	0.04972208	0.52588159
FBgn000264	mal	8.69002506	4.98396217	3.84984676	0.04975054	0.52588159
FBgn001562	Cnx99A	2.38469947	8.67248034	3.84247564	0.04996969	0.52722178
FBgn002658	Hmg-2	-10.010283	4.09035647	3.83742974	0.0501203	0.52783516
FBgn003816	Lkb1	-9.4307177	4.39398564	3.8272363	0.05042602	0.53007678
FBgn026260	CG43134	9.56118518	4.12020061	3.82091954	0.05061645	0.53110057
FBgn003666	CG9674	-5.9453198	6.23041943	3.81057342	0.05093001	0.53341011
FBgn003970	Vps16B	-9.8590811	3.89414285	3.79250587	0.05148252	0.53804645
FBgn004213	CG18809	-8.9019954	4.32018387	3.78790721	0.05162416	0.53804645
FBgn002350	Ocrl	7.3117847	5.47844574	3.78689217	0.05165548	0.53804645
FBgn025084	26-29-p	2.40464744	8.71344854	3.78278092	0.05178253	0.53838741
FBgn003016	CG9691	2.57257144	8.13114918	3.77573716	0.05200098	0.53967565
FBgn000569	Aef1	-9.2492622	4.63473737	3.76581258	0.05231044	0.54190193

FBgn003629	CG10654	-5.2773524	6.78106468	3.74278127	0.0530361	0.54764664
FBgn001503	Cyp4p1	8.27523174	6.40811654	3.74212696	0.05305687	0.54764664
FBgn026127	lqfR	7.29923204	7.17737745	3.73790433	0.05319112	0.54804135
FBgn003100	CG8010	-8.2915871	5.53822949	3.72645482	0.05355695	0.54984694
FBgn002862	qsm	-8.4475726	4.62035484	3.7263903	0.05355902	0.54984694
FBgn001965	toy	-7.8888735	4.90287504	3.71134335	0.05404388	0.55382848
FBgn003176	CG9135	-8.9321501	4.75280403	3.69611524	0.05453931	0.55790392
FBgn003303	CG8245	-6.820402	6.95151163	3.6893493	0.05476098	0.55916932
FBgn003172	DIP-eta	-7.9521157	4.92361435	3.68142792	0.05502171	0.56082843
FBgn003245	spict	7.40995555	6.12367898	3.67487198	0.0552385	0.56203446
FBgn002852	CG5888	-8.0812672	5.63612501	3.66305782	0.05563145	0.56502542
FBgn004482	Pak3	-8.5525591	4.89305759	3.65125982	0.05602682	0.56803032
FBgn005248	Sk2	-6.3018698	5.79835279	3.64798744	0.05613701	0.56813837
FBgn003183	CG13766	-9.2580229	4.92572921	3.64344434	0.05629037	0.56868216
FBgn000120	hook	-5.6270195	7.06595523	3.63767708	0.05648569	0.5691089
FBgn003743	CG18048	8.53403713	5.99023218	3.63631243	0.05653202	0.5691089
FBgn004503	Proc	-8.3077907	5.67042503	3.62899333	0.05678115	0.5697384
FBgn003338	CG8046	4.91543479	7.23063735	3.62861168	0.05679418	0.5697384
FBgn003034	CG10353	-6.7488811	5.2646784	3.61817725	0.05715146	0.5723167
FBgn026273	Src64B	-6.7795265	9.91214513	3.59673335	0.05789323	0.57769495
FBgn003587	CG7083	-7.3030307	6.32006562	3.59050076	0.05811073	0.57769495
FBgn004082	CG13315	3.37776002	8.05039677	3.59015281	0.0581229	0.57769495
FBgn026737	SelR	-6.8466072	7.56077927	3.58620954	0.05826099	0.57769495
FBgn003426	HPS4	-7.1072667	6.98495302	3.58459701	0.05831756	0.57769495
FBgn003418	CG8963	-8.4440302	5.93854049	3.58190553	0.05841211	0.57769495
FBgn003157	Cep97	-8.6794379	4.88482081	3.5794792	0.05849749	0.57769495
FBgn002981	GAA1	-7.1790365	7.13300164	3.57717672	0.05857863	0.57769495
FBgn003868	CG11779	-8.370261	5.74961561	3.57658788	0.0585994	0.57769495
FBgn005210	SCaMC	-5.1423857	6.81786954	3.5641722	0.05903917	0.58102687
FBgn001374	alien	7.0562672	7.81742385	3.55379817	0.05940932	0.58366329
FBgn005234	CG32344	-9.5625643	3.62108333	3.53652745	0.06003102	0.58843922
FBgn003368	CG18343	8.27422171	5.71654801	3.53457809	0.06010162	0.58843922
FBgn008700	bbg	-8.3725799	4.32070248	3.51349478	0.06087093	0.59495077
FBgn003390	CysRS-m	-9.6892933	4.29329901	3.50221615	0.06128677	0.59663645
FBgn002625	eIF5B	6.55308678	5.75476651	3.50069927	0.06134292	0.59663645
FBgn003003	PIG-T	5.9534274	6.05008685	3.50032001	0.06135697	0.59663645
FBgn003430	CG5098	-6.9255997	5.1068302	3.49171304	0.06167673	0.59802922
FBgn002854	NimB2	5.3158741	8.91630274	3.4908272	0.06170974	0.59802922
FBgn003753	CG2767	4.52883923	6.77049211	3.48552287	0.0619078	0.59893175
FBgn003442	CG7137	-7.8476255	5.10902646	3.48137297	0.06206323	0.59913936
FBgn026479	lncRNA:CR44	-7.5750437	5.8838212	3.47934873	0.06213919	0.59913936
FBgn002579	TBPH	-7.6872958	5.64989367	3.47649671	0.06224639	0.59916084
FBgn003677	CG13698	-9.6791747	3.94868238	3.46594832	0.06264458	0.60197853
FBgn026700	unc-104	-2.7955336	8.09892641	3.46268036	0.06276849	0.60215552
FBgn003397	Ciao1	-8.7316643	4.2691105	3.44271119	0.06353136	0.60845134
FBgn003008	CCT2	9.59605353	3.62870121	3.43514842	0.06382285	0.60952137

FBgn003147	Prx6005	-8.1377615	4.77433449	3.43427379	0.06385665	0.60952137
FBgn003291	tadr	-9.2535378	4.15577741	3.42780872	0.06410711	0.61089044
FBgn003142	CG9886	-9.2483618	4.18969027	3.42435004	0.06424153	0.61115105
FBgn000404	Yp3	2.42558061	10.1060409	3.38292287	0.06587513	0.62561555
FBgn003779	TAF1B	-7.4389276	5.1016355	3.38027168	0.06598118	0.62561555
FBgn003651	CG16979	-8.7381746	4.80351535	3.36755373	0.06649242	0.62941746
FBgn002424	chico	6.37304436	6.9761399	3.34343628	0.06747356	0.63764749
FBgn001062	CCT5	6.79328411	7.71528065	3.31845265	0.06850628	0.64633692
FBgn003373	DUBAI	5.67217456	6.54342844	3.31140055	0.06880083	0.64675153
FBgn002998	CG10778	-9.2539844	4.33733895	3.30978155	0.06886865	0.64675153
FBgn005170	CG31706	-6.0229353	6.58440166	3.30926873	0.06889014	0.64675153
FBgn002215	l(2)k05819	-8.2144525	4.62908975	3.29983224	0.06928696	0.64859616
FBgn002832	Pdha	-2.7980496	8.05316662	3.29919403	0.06931389	0.64859616
FBgn003432	IM23	4.70376397	6.34248894	3.2905603	0.06967924	0.6509478
FBgn003398	ckn	-6.7828285	5.38124054	3.28554603	0.06989238	0.65187204
FBgn003817	Adgf-D	-6.2145966	6.78025465	3.27081807	0.07052245	0.65576696
FBgn025948	Mob2	-5.9606937	6.90132491	3.27041555	0.07053975	0.65576696
FBgn002708	LysRS	-9.4486468	3.78162488	3.26274572	0.07087037	0.6568804
FBgn003718	CG14450	-9.0604456	4.47854123	3.26229873	0.07088969	0.6568804
FBgn002911	ScsbetaG	-6.8239205	6.81455868	3.25753285	0.07109602	0.6577246
FBgn003431	CG10916	6.8730947	6.56192575	3.25468498	0.07121962	0.65780194
FBgn002709	ArgRS	-9.1268159	4.44656754	3.24752781	0.07153127	0.65809556
FBgn000458	Cbp53E	-3.1155809	10.7693647	3.24523834	0.07163127	0.65809556
FBgn002418	vig	-2.713038	7.92309697	3.24459388	0.07165945	0.65809556
FBgn003440	DptB	7.8546587	7.09459405	3.24337923	0.07171259	0.65809556
FBgn003025	CG1552	-2.1071322	8.9478778	3.23877715	0.0719143	0.65888732
FBgn003935	jigr1	-5.423869	7.22531434	3.23355514	0.07214392	0.65993183
FBgn005134	CG31344	-8.4683636	5.75623856	3.23056798	0.07227562	0.66007876
FBgn006509	snRNA:7SK	3.13220026	7.68935132	3.2099461	0.0731919	0.66559514
FBgn003881	Pus1	7.59740245	5.74281961	3.20957446	0.07320852	0.66559514
FBgn003471	wdp	-8.3699695	3.90263679	3.2091066	0.07322946	0.66559514
FBgn003186	Nlg2	-7.2739454	6.78082007	3.19844599	0.07370828	0.6688821
FBgn003960	CG1646	-6.1895761	5.56809915	3.19116782	0.0740371	0.67079966
FBgn001413	bif	5.96562313	5.95994167	3.17843076	0.07461636	0.67419715
FBgn002572	Ate1	6.62943264	5.54218186	3.1756376	0.07474403	0.67419715
FBgn000112	Galphao	-3.5645651	8.40386342	3.17514812	0.07476643	0.67419715
FBgn002840	Syt4	-3.8308707	7.17076566	3.16572131	0.07519921	0.67552132
FBgn003219	CG5734	-8.2862924	5.88690633	3.16526844	0.07522007	0.67552132
FBgn004158	AttB	8.77169021	5.19594161	3.1642214	0.07526832	0.67552132
FBgn004379	phu	4.162317	6.57719453	3.16028397	0.07545006	0.67575423
FBgn003605	nudE	-4.2566876	7.54037104	3.15628909	0.07563493	0.67575423
FBgn003027	CG15202	-8.6907633	3.43271137	3.15562418	0.07566575	0.67575423
FBgn026263	dati	-7.3546275	5.43709959	3.15342406	0.07576782	0.67575423
FBgn003326	Nup50	-8.8783933	3.80732359	3.14961994	0.07594465	0.67627467
FBgn004506	bwa	-9.2003818	3.53830082	3.14336209	0.07623651	0.67707043
FBgn002982	CG3726	6.41681555	6.34811103	3.14261891	0.07627125	0.67707043

FBgn000256	Rab32	3.43827024	7.49747964	3.13748485	0.07651171	0.67815039
FBgn025914	CG42255	4.08275855	6.59653552	3.12660305	0.07702408	0.6812994
FBgn003502	Fcp1	-8.2197794	5.60470866	3.12487651	0.07710571	0.6812994
FBgn026398	spoon	-8.8958147	4.54179939	3.12118768	0.07728044	0.68178785
FBgn003980	Npc2g	6.35093196	6.39399146	3.11729039	0.0774655	0.68236584
FBgn003956	CG4951	-8.1027193	3.97690693	3.1110295	0.07776379	0.68393795
FBgn004115	hoe1	-7.915311	6.30363735	3.10819762	0.07789912	0.68407412
FBgn000577	noc	-7.6735261	4.45618624	3.09794655	0.07839112	0.68733716
FBgn001375	Mtor	7.71369251	4.19323869	3.07917466	0.07930076	0.69424652
FBgn003432	IM1	8.07103405	5.01870963	3.07099984	0.07970044	0.69634381
FBgn001055	Phb2	-6.6290071	7.80909898	3.06720526	0.0798867	0.69634381
FBgn000117	hay	-8.5091435	3.25991007	3.06680639	0.07990631	0.69634381
FBgn003520	sturkopf	5.45788041	6.45733827	3.06056179	0.08021393	0.69739924
FBgn026164	CG42724	-8.2488014	5.01731533	3.05939046	0.08027178	0.69739924
FBgn001126	Sema2a	5.62356119	6.87095757	3.04693539	0.08088965	0.70073043
FBgn002228	l(2)09851	7.50044948	6.26802859	3.04671295	0.08090073	0.70073043
FBgn003472	Rtf1	-8.4907189	5.80556506	3.0402112	0.08122535	0.7024762
FBgn002352	CG3587	7.85859019	5.95144743	3.03239564	0.08161743	0.7038452
FBgn026791	lncRNA:CR46	6.75688143	5.35750617	3.03214053	0.08163026	0.7038452
FBgn000277	mle	-6.5155173	5.20271272	3.0218476	0.08214983	0.70634298
FBgn025921	PMCA	-3.2719326	7.62188739	3.02149992	0.08216744	0.70634298
FBgn003403	CG8204	-8.6068389	2.63094003	3.01890115	0.08229921	0.70641189
FBgn002847	Non1	5.31994754	7.09862694	3.01513126	0.08249078	0.70699302
FBgn005259	be	4.87751454	6.3760423	3.0108428	0.08270928	0.70780292
FBgn004060	CG6503	3.88624384	6.68561659	3.00515092	0.08300025	0.70922966
FBgn006349	GstE6	3.73989437	6.97880725	3.0006259	0.08323236	0.709268
FBgn003449	Mpcp1	-8.5461909	3.78079055	2.99661909	0.08343847	0.709268
FBgn026086	dnr1	-8.9666701	4.63435414	2.99249178	0.08365136	0.709268
FBgn026541	CG44325	2.34265885	8.47059622	2.99191596	0.08368111	0.709268
FBgn003354	CG12338	5.9395975	6.35905694	2.99187798	0.08368307	0.709268
FBgn003578	ppk26	7.30459258	6.00588157	2.99057769	0.08375029	0.709268
FBgn002353	CG3156	-8.2032092	4.56812852	2.9808463	0.08425521	0.71236799
FBgn003480	CG3831	6.92912926	6.44532969	2.97872062	0.08436594	0.71236799
FBgn000464	ogre	-3.7776975	6.81652926	2.9735399	0.08463647	0.71258682
FBgn003564	CG10483	-9.0172031	3.53305616	2.97313872	0.08465745	0.71258682
FBgn026510	Oseg1	-8.559305	3.48996158	2.97105853	0.08476637	0.71258682
FBgn002999	CG2233	2.12300858	9.12017039	2.95540518	0.08559085	0.71771134
FBgn026172	fwe	-2.0232964	8.85270276	2.9547143	0.08562744	0.71771134
FBgn000339	shi	-1.88707	10.1806476	2.9455384	0.086115	0.71939231
FBgn002842	Kap3	-8.3189556	4.70969175	2.94174771	0.08631729	0.71939231
FBgn002857	qtc	-6.9835164	7.01942322	2.94147268	0.08633199	0.71939231
FBgn000277	Mlc2	2.55967266	7.92336596	2.94147026	0.08633212	0.71939231
FBgn003934	Ssadh	-2.0205588	8.86857957	2.92592113	0.08716747	0.7252943
FBgn003058	CG14411	-6.1742102	5.69948852	2.91187106	0.08792981	0.73057256
FBgn001755	Rca1	-8.1021054	4.06848005	2.90247098	0.08844388	0.73377568
FBgn003642	Gbs-70E	7.8012674	3.39115079	2.89245359	0.0889953	0.7372236

FBgn003591[illegible]	CG6282	-6.4440218	4.9390737	2.88947755	0.08915983	0.7372236
FBgn003655[illegible]	mRpS31	-7.0965286	6.81272525	2.88687622	0.08930392	0.7372236
FBgn003904[illegible]	CG17121	-8.7991191	3.96731656	2.88557506	0.08937609	0.7372236
FBgn026152[illegible]	lic	7.01193899	6.54903681	2.88178863	0.08958646	0.73789253
FBgn003915[illegible]	TBC1d7	-8.7455046	3.89360401	2.8739877	0.09002157	0.7383248
FBgn003537[illegible]	AhcyL1	-5.0037627	6.57997832	2.87392022	0.09002534	0.7383248
FBgn001334[illegible]	nSyb	-3.1880704	8.80074406	2.87389072	0.09002699	0.7383248
FBgn002970[illegible]	CG3626	-8.6552603	2.92009625	2.8627597	0.09065185	0.74238273
FBgn000437[illegible]	fwd	4.92819749	6.297991	2.85484441	0.09109905	0.74497621
FBgn003695[illegible]	CG17233	-5.8979467	5.5031709	2.84824532	0.09147372	0.74577947
FBgn026311[illegible]	5-HT1B	-9.0010733	4.02024683	2.84819235	0.09147674	0.74577947
FBgn003964[illegible]	jus	-5.7483308	6.29009503	2.84535346	0.09163844	0.74577947
FBgn003771[illegible]	P58IPK	6.88848101	6.21441278	2.84392536	0.0917199	0.74577947
FBgn005310[illegible]	CG33107	5.8338131	6.77451396	2.83017002	0.09250857	0.75016992
FBgn003159[illegible]	CG3702	-7.3635744	6.25113255	2.82992451	0.09252271	0.75016992
FBgn008545[illegible]	CG34423	8.35214779	5.45008726	2.82086843	0.09304605	0.75334305
FBgn004379[illegible]	CG12219	-7.7745013	4.14451426	2.80328344	0.09407148	0.7605666
FBgn003034[illegible]	CG10352	7.54658859	6.10222316	2.79714349	0.09443241	0.76185561
FBgn000054[illegible]	ecd	6.65290842	6.53482829	2.79486762	0.09456658	0.76185561
FBgn003452[illegible]	Nnf1a	7.61660115	5.60406916	2.79377046	0.09463133	0.76185561
FBgn001434[illegible]	mirr	-6.8990629	6.37262734	2.79044851	0.09482768	0.76236115
FBgn003202[illegible]	CG7627	8.16919474	2.94786259	2.78579373	0.09510356	0.76331417
FBgn026374[illegible]	CG43673	7.76419044	6.2450647	2.78268578	0.09528825	0.76331417
FBgn003470[illegible]	CG6758	-7.2598624	4.25895743	2.7777876	0.09558012	0.76331417
FBgn026319[illegible]	Galk	4.32643281	6.76611745	2.77688448	0.09563404	0.76331417
FBgn003228[illegible]	CG17108	3.27607942	7.33919768	2.77399235	0.09580693	0.76331417
FBgn000199[illegible]	wek	-8.0535439	3.13377124	2.77235915	0.09590472	0.76331417
FBgn003611[illegible]	CG7888	-2.8611226	7.22989392	2.77188256	0.09593327	0.76331417
FBgn026198[illegible]	l(2)gd1	4.98961951	6.42986281	2.76979158	0.09605867	0.76331417
FBgn002852[illegible]	CG15293	3.55196003	7.01510395	2.76827429	0.09614977	0.76331417
FBgn002756[illegible]	Tps1	3.45103427	6.79889904	2.7650485	0.09634377	0.76379198
FBgn002182[illegible]	DCTN2-p50	-5.2231968	6.8758849	2.75711627	0.09682262	0.76652362
FBgn026605[illegible]	Patr-1	-6.7306562	5.99990989	2.74917728	0.09730449	0.76816199
FBgn026399[illegible]	Ack-like	-6.1943141	5.02398829	2.7478645	0.09738442	0.76816199
FBgn003087[illegible]	Ucp4A	-6.5959977	7.07125604	2.74706222	0.0974333	0.76816199
FBgn003313[illegible]	Tsp42En	-8.6460451	3.48192811	2.74312539	0.09767357	0.76899408
FBgn026735[illegible]	PI4KIIIalpha	-6.5294078	4.69319493	2.73914355	0.09791723	0.76982931
FBgn003991[illegible]	Gat	-3.1424127	7.04750195	2.73698849	0.09804939	0.76982931
FBgn003539[illegible]	scramb2	2.4999291	7.77797492	2.72867849	0.09856081	0.77086924
FBgn026127[illegible]	Opa1	-6.9314549	6.71550138	2.72664631	0.09868632	0.77086924
FBgn003572[illegible]	CG10077	-1.8508827	9.45795224	2.72569107	0.09874537	0.77086924
FBgn003664[illegible]	CG4098	-8.3290971	3.27267279	2.72520704	0.09877531	0.77086924
FBgn002539[illegible]	inc	-7.5474404	3.26545941	2.71955364	0.09912573	0.77086924
FBgn003548[illegible]	CG11593	4.51703758	6.45617146	2.71744394	0.09925685	0.77086924
FBgn025993[illegible]	CG42458	-7.2122336	5.27293607	2.71692636	0.09928904	0.77086924
FBgn003188[illegible]	Caper	-4.4135266	7.24997236	2.71562377	0.09937012	0.77086924

FBgn003870	CG5316	-7.671601	5.96484005	2.71309286	0.09952786	0.77086924
FBgn003908	beat-IV	-6.5319871	5.61998614	2.71302093	0.09953235	0.77086924
FBgn000491	Hnf4	8.48025829	3.27761759	2.70673321	0.09992543	0.77286498
FBgn002347	Taldo	2.2233227	8.36300919	2.70278236	0.10017329	0.77301553
FBgn004432	CG32052	-8.0071538	5.0549425	2.69927477	0.1003939	0.77301553
FBgn002749	Madm	-8.5099688	3.9983228	2.69741511	0.10051109	0.77301553
FBgn004073	IM3	4.30057221	6.43005491	2.69267319	0.10081056	0.77301553
FBgn002568	scf	2.11387558	8.5834997	2.69239052	0.10082844	0.77301553
FBgn004096	CG18661	-8.1681647	4.28535777	2.68990005	0.10098615	0.77301553
FBgn005211	CG32112	-7.9985506	4.90374154	2.68966651	0.10100095	0.77301553
FBgn002636	Sara	-7.3842884	5.30024237	2.68923498	0.10102831	0.77301553
FBgn003958	Moca-cyp	-7.3031351	6.47618773	2.68143011	0.10152452	0.77402165
FBgn001090	msn	3.26205191	6.88265969	2.68022857	0.10160115	0.77402165
FBgn000130	Khc	-5.3743042	6.61573992	2.67890372	0.10168571	0.77402165
FBgn003887	CG3308	-6.3187723	7.31113608	2.67864526	0.10170222	0.77402165
FBgn026252	ver	6.77028315	5.42939897	2.67437304	0.10197548	0.77506795
FBgn003488	Eglp4	7.17652496	6.32143282	2.66983561	0.10226659	0.77624691
FBgn002454	Neos	-7.4984602	5.45945183	2.66434304	0.10262019	0.77789647
FBgn003493	Orcokinin	2.73610424	11.6250713	2.65672118	0.10311309	0.78059618
FBgn000052	dx	-8.0703518	2.65254928	2.65319273	0.10334215	0.78099234
FBgn026366	lncRNA:CR43	8.66115313	3.65504863	2.65170236	0.10343907	0.78099234
FBgn001664	PTP-ER	-6.3320509	5.29161212	2.64842136	0.10365279	0.78157212
FBgn003627	INPP5E	-8.1232756	3.74687951	2.64201112	0.10407173	0.78307058
FBgn003469	Synj	-6.9724367	5.60057931	2.64114545	0.10412844	0.78307058
FBgn003156	IM33	5.62709279	5.11370457	2.63909264	0.10426308	0.78307058
FBgn002760	pdgy	-5.9056191	6.11098741	2.63275424	0.10467999	0.78516866
FBgn026475	CG44008	8.09896076	5.0309733	2.62946858	0.10489682	0.78576251
FBgn003873	CG4572	2.06707055	8.66668299	2.62025041	0.10550779	0.78872067
FBgn003033	Tango10	-5.8080339	5.07366307	2.61934397	0.10556808	0.78872067
FBgn003315	Gadd45	3.90972448	6.81463894	2.59982794	0.10687532	0.79744355
FBgn003524	CG13919	-8.0872544	2.70130363	2.59142921	0.10744335	0.79868833
FBgn001400	Hf	5.48045429	5.73824877	2.58941936	0.10757977	0.79868833
FBgn026074	mfas	-5.9965804	6.22387037	2.58882029	0.10762047	0.79868833
FBgn003576	mrva	2.6203395	9.46492267	2.58531043	0.10785926	0.79868833
FBgn003763	CG8379	4.50269946	6.92941596	2.58501781	0.1078792	0.79868833
FBgn003138	Npc2a	2.30186375	8.31564619	2.5821419	0.10807533	0.79868833
FBgn000417	Csp	-6.7937102	7.10507107	2.58172333	0.10810391	0.79868833
FBgn003434	CG5174	2.00107492	8.3790842	2.58087962	0.10816154	0.79868833
FBgn003001	Gbeta5	-1.8324598	9.18122051	2.57690968	0.10843317	0.79965961
FBgn001177	Hem	-8.0368456	5.30261282	2.57044828	0.10887687	0.80189574
FBgn026094	Rbp1	-2.4530254	8.13526914	2.56643052	0.10915378	0.80289919
FBgn026072	lncRNA:CR42	-8.4349837	4.15746705	2.5592572	0.10965009	0.80471701
FBgn001996	Mitofilin	-5.0206472	6.8562334	2.5551467	0.10993561	0.80471701
FBgn003783	CG14687	-2.7410069	7.26751161	2.5542525	0.10999783	0.80471701
FBgn001487	Psi	-6.94222	4.77520447	2.55186719	0.11016399	0.80471701
FBgn026104	stj	-8.2299417	5.05151166	2.55100346	0.11022423	0.80471701

FBgn026079	2mit	-8.3918787	3.52324585	2.55068002	0.1102468	0.80471701
FBgn003156	CG2818	-6.5042757	5.04269235	2.54395386	0.11071722	0.80711866
FBgn026359	Vha68-2	2.66153755	8.03798354	2.54115423	0.11091368	0.80751949
FBgn003879	MFS9	7.18935151	6.48868665	2.5380549	0.11113162	0.80807549
FBgn003684	Ugt316A1	4.12489325	7.10644949	2.53493996	0.11135112	0.80864148
FBgn026610	CG44838	-6.1480278	5.47973558	2.52923711	0.11175424	0.80962887
FBgn003270	CG10376	6.7017439	6.46482662	2.52900369	0.11177077	0.80962887
FBgn003130	Iris	8.22336782	5.59463653	2.52530306	0.11203326	0.81050167
FBgn000278	mod	-6.4142778	6.50048375	2.52194219	0.11227223	0.81120238
FBgn002062	RN-tre	6.59439631	6.24761351	2.51606732	0.11269131	0.81263627
FBgn001132	Uch-L5	-7.5472606	5.5713804	2.51517077	0.11275542	0.81263627
FBgn026675	Esyt2	4.17773759	6.416741	2.51027256	0.11310637	0.81413764
FBgn026212	uex	-3.7133837	7.28937729	2.50646343	0.11338012	0.81417988
FBgn003199	CG8475	-6.2408625	4.82810201	2.50622173	0.11339751	0.81417988
FBgn008540	side-V	-8.2059389	3.41349793	2.50315521	0.11361846	0.81474144
FBgn003495	Xxylt	-6.3993805	6.99539019	2.496606	0.11409194	0.81711014
FBgn003524	CG13928	-5.8346253	7.92867229	2.49437646	0.11425362	0.81724268
FBgn003439	CG15098	2.14489347	9.34480413	2.47894685	0.11537949	0.82400267
FBgn003625	eIF3l	-4.7066547	6.67261462	2.47747653	0.11548741	0.82400267
FBgn002483	AP-1mu	-5.1282301	7.12695919	2.46689292	0.11626756	0.82853336
FBgn002765	glob1	7.39117229	5.97396503	2.4587764	0.11686979	0.8317865
FBgn003051	Nadsyn	-6.1468303	5.02588233	2.45421253	0.11720994	0.83316852
FBgn000055	eEF1alpha2	-2.3318931	9.01591681	2.44951784	0.11756097	0.83449406
FBgn003975	CG9743	7.67479108	6.19447088	2.44781206	0.11768881	0.83449406
FBgn003316	Dhx15	6.18576938	6.08918201	2.44113932	0.11819036	0.83636514
FBgn003327	Gasz	4.70827703	6.19797399	2.44040441	0.11824574	0.83636514
FBgn003480	CG9896	-7.6239321	4.45133819	2.43650176	0.11854032	0.83741108
FBgn005040	Tango11	-5.8728292	7.36681472	2.42659494	0.11929177	0.84113844
FBgn003428	dpr13	-6.9403867	6.38255687	2.42566377	0.11936267	0.84113844
FBgn003106	Pmp70	-7.5834352	5.34054823	2.41499377	0.12017845	0.84584289
FBgn003074	AnxB11	6.75935727	5.31628492	2.41113473	0.12047501	0.8468859
FBgn003195	Herp	-7.648869	5.52215261	2.40584351	0.12088295	0.84870834
FBgn003083	CG5613	6.69864041	5.94553228	2.40077655	0.12127504	0.85041511
FBgn003512	Tudor-SN	-7.4312094	5.96221293	2.39583716	0.12165861	0.85205807
FBgn002915	Men-b	-6.6325465	7.11308524	2.39010235	0.12210563	0.85414088
FBgn026622	lncRNA:CR44	-7.6402263	5.83190877	2.38742732	0.12231478	0.85421061
FBgn000331	sax	-5.5284402	5.2739767	2.38477967	0.12252217	0.85421061
FBgn026158	pdm3	-6.0096693	5.07116746	2.3842391	0.12256456	0.85421061
FBgn003040	CG1824	-6.6140909	6.48883008	2.38101546	0.12281769	0.85493094
FBgn003441	CG15111	4.54704916	6.6493463	2.37497572	0.12329352	0.85719779
FBgn003506	TyrRS-m	7.396045	5.65964818	2.37172222	0.12355068	0.85794074
FBgn003520	Herc4	-6.4209694	5.05154981	2.36048221	0.12444372	0.86112628
FBgn002643	Eaat1	-3.1236994	6.84131363	2.35805362	0.12463761	0.86112628
FBgn003917	CG13609	-7.7518995	2.89669958	2.35745547	0.12468542	0.86112628
FBgn004624	CG5938	-4.4446946	6.93258913	2.35366738	0.12498866	0.86112628
FBgn003070	MSBP	-6.1736567	7.68966858	2.35273424	0.12506348	0.86112628

FBgn026185	aPKC	-4.4276247	6.32444545	2.35070484	0.12522638	0.86112628
FBgn008685	CG17078	-6.3977813	4.72415953	2.34913492	0.12535255	0.86112628
FBgn003356	CG18004	7.38854248	5.58986265	2.34910336	0.12535509	0.86112628
FBgn003299	CG17486	-6.9713441	6.46518034	2.34835734	0.1254151	0.86112628
FBgn002037	TM4SF	-1.7854967	10.0030131	2.34707837	0.12551806	0.86112628
FBgn002672	fat-spondin	3.07946413	8.7270802	2.34138974	0.12597712	0.8627746
FBgn001087	l(3)05822	5.20034084	6.4322379	2.34035751	0.12606062	0.8627746
FBgn001376	Idgf6	1.80706838	9.21611014	2.33542343	0.1264606	0.86447557
FBgn005145	lncRNA:CR31	-1.5676994	12.1589151	2.32902747	0.12698119	0.86569348
FBgn002562	CG4045	7.57115594	5.48511076	2.32682846	0.12716072	0.86569348
FBgn000326	Rm62	-1.7784791	9.35021553	2.32322953	0.12745516	0.86569348
FBgn003610	CG6418	7.12408082	5.83744108	2.32235184	0.12752708	0.86569348
FBgn003456	dgt3	-6.8473844	3.40328103	2.32122702	0.12761932	0.86569348
FBgn003055	mRNA-cap	-7.4936591	4.92559378	2.32026893	0.12769795	0.86569348
FBgn003657	CG5027	-7.1235211	6.1967313	2.320239	0.1277004	0.86569348
FBgn003103	Tao	3.01555663	7.09815125	2.30884743	0.1286394	0.87018164
FBgn028424	eEF1alpha1	1.97881891	10.7746186	2.30850968	0.12866736	0.87018164
FBgn000469	Xpc	-6.1899611	6.96448686	2.30233517	0.12917966	0.87261242
FBgn003331	beta3GalTII	-7.3228137	4.03950352	2.29287117	0.1299693	0.87541934
FBgn028425	bsf	-7.3805549	5.96416164	2.28919003	0.13027789	0.87541934
FBgn004027	Spt5	-5.9740246	5.19851888	2.28910793	0.13028479	0.87541934
FBgn003071	CG9170	-7.8161534	5.92689214	2.28902346	0.13029188	0.87541934
FBgn000021	brm	-6.3627877	4.14439443	2.28599708	0.13054622	0.87541934
FBgn002567	CycK	-7.7958689	5.15284865	2.28530017	0.13060487	0.87541934
FBgn003398	ADPS	-7.1149132	6.11827372	2.28454141	0.13066876	0.87541934
FBgn026127	grp	-6.6316468	6.52000907	2.28078154	0.13098585	0.87651495
FBgn003945	CG6420	-7.4671108	4.41321749	2.27226904	0.13170695	0.87792823
FBgn001035	Cds	-4.4812027	8.4086632	2.27201291	0.13172871	0.87792823
FBgn003512	mRpL17	-5.9970381	7.1772158	2.27012383	0.13188936	0.87792823
FBgn001612	Acer	3.17723572	6.72382131	2.26928854	0.13196047	0.87792823
FBgn003111	GstT3	5.23856223	5.08958635	2.26806765	0.13206448	0.87792823
FBgn000468	Mlc-c	2.36279204	7.74725831	2.26741758	0.13211989	0.87792823
FBgn003968	Obp99c	2.74130534	7.07075623	2.26292984	0.13250317	0.87945126
FBgn003562	Eaf6	7.38064044	5.06331703	2.25927561	0.13281618	0.88050491
FBgn003531	CG9018	-6.2456085	4.30385982	2.25684444	0.13302488	0.88079231
FBgn003732	POLDIP2	-6.84742	6.41783429	2.25154139	0.13348139	0.88079231
FBgn005139	MED7	2.3944188	7.42554305	2.25049329	0.13357182	0.88079231
FBgn004213	Gdap2	-4.1333144	6.70024801	2.24234301	0.13427738	0.88079231
FBgn002760	CG8230	-6.6103092	6.34302552	2.24143421	0.13435631	0.88079231
FBgn003159	Vps53	-6.5581398	5.40930787	2.240984	0.13439543	0.88079231
FBgn000003	nAChRbeta1	-2.2259219	8.99410394	2.23869551	0.13459449	0.88079231
FBgn000027	Pka-C1	-6.2515827	7.53368135	2.23820993	0.13463677	0.88079231
FBgn003630	Pmm2	7.25758939	5.98296995	2.23769754	0.1346814	0.88079231
FBgn026359	lost	-1.7002386	9.67920464	2.23725424	0.13472002	0.88079231
FBgn003464	CG10082	-7.329315	5.48202248	2.23503942	0.13491318	0.88079231
FBgn001076	simj	-5.1961966	5.36445472	2.23436598	0.13497198	0.88079231

FBgn003921	atl	-4.362285	6.48279213	2.23382433	0.13501929	0.88079231
FBgn001120	pelo	-7.495024	5.01438191	2.23356803	0.13504168	0.88079231
FBgn003893	CG5778	5.15115083	5.16025977	2.23205278	0.13517415	0.88079231
FBgn003715	CG7133	6.68746033	5.83293615	2.22452793	0.13583417	0.88408374
FBgn026269	fzr	-4.1605647	6.59801366	2.21152786	0.13698295	0.88971174
FBgn003517	CG12502	-7.7173366	3.85175707	2.21121618	0.13701062	0.88971174
FBgn003026	Klp10A	3.71988763	6.31386988	2.20894577	0.13721241	0.89000959
FBgn000404	Yp1	2.01613	9.59608287	2.20417395	0.13763761	0.89091215
FBgn003245	MRP	5.08167617	5.40392011	2.20082591	0.13793682	0.89091215
FBgn003171	CG14015	-6.868818	6.82104634	2.20062713	0.1379546	0.89091215
FBgn002756	CG5498	-6.458687	5.61934913	2.20038936	0.13797588	0.89091215
FBgn002605	Rlip	-6.6626939	7.2965185	2.19742622	0.13824139	0.89119887
FBgn000025	cact	5.23973108	6.12022272	2.19641028	0.13833255	0.89119887
FBgn002608	cib	2.42948847	7.52416244	2.1897736	0.13892974	0.89335593
FBgn028424	betaTub56D	-1.5273639	10.7137702	2.18921213	0.13898039	0.89335593
FBgn003097	CG7332	-4.8501501	6.98182488	2.18397336	0.13945403	0.8953921
FBgn003508	CG2765	-5.4842403	6.37803769	2.18188347	0.13964348	0.89560108
FBgn003629	CG10681	-7.6916655	3.34369678	2.17828498	0.13997036	0.89655917
FBgn026156	CG42675	5.28424715	5.59863081	2.17533043	0.14023939	0.89655917
FBgn001550	Cul1	-4.7051953	5.99727812	2.17505969	0.14026407	0.89655917
FBgn008668	l(3)L1231	-6.7844142	6.40472013	2.17200596	0.1405428	0.89678005
FBgn002568	CG3558	7.37114248	2.54500239	2.17107215	0.14062816	0.89678005
FBgn005171	CG31710	7.11517992	5.66814513	2.16782677	0.14092527	0.89678005
FBgn026474	CG44002	6.84351463	5.55821532	2.16638716	0.14105728	0.89678005
FBgn003685	Aldh7A1	-6.603607	2.87501765	2.16520966	0.14116537	0.89678005
FBgn000327	RpLP2	1.59546992	10.142113	2.16438318	0.14124129	0.89678005
FBgn003283	CG10680	7.27525137	6.43300908	2.15827913	0.14180342	0.89781464
FBgn000066	Actn	-5.104591	5.73252827	2.15814128	0.14181615	0.89781464
FBgn000010	Appl	-2.561517	7.65783657	2.1566234	0.14195634	0.89781464
FBgn003752	mRpS9	-7.794881	4.16570914	2.15482118	0.14212299	0.89781464
FBgn002449	Bin1	7.30308059	5.6698912	2.15408977	0.14219069	0.89781464
FBgn003114	Stt3A	-5.8076211	6.36175886	2.15042259	0.14253065	0.89896682
FBgn002450	Sec13	2.38927998	7.38033429	2.1470181	0.14284709	0.89925251
FBgn003032	Amun	-5.0918928	5.31167676	2.14469419	0.14306354	0.89925251
FBgn003460	ASPP	-7.8641361	4.41025908	2.14200448	0.14331453	0.89925251
FBgn003152	CG8837	-6.7990942	5.98613573	2.14199007	0.14331587	0.89925251
FBgn005205	scramb1	5.38247791	7.36953231	2.13877945	0.14361612	0.89925251
FBgn003848	m-cup	-6.3844282	3.18358119	2.13509353	0.14396168	0.89925251
FBgn028351	Pal1	-7.2522129	4.94429764	2.13372319	0.14409039	0.89925251
FBgn003489	sigmar	-6.7459884	6.18614591	2.13330726	0.14412949	0.89925251
FBgn000114	Gs2	-1.8209612	10.0777613	2.12863704	0.14456926	0.89925251
FBgn002499	eIF2Bbeta	-2.7908395	6.9681742	2.12747725	0.1446787	0.89925251
FBgn026652	stai	-2.5663045	7.42631356	2.12439626	0.14496989	0.89925251
FBgn003087	CG6769	2.96289019	7.074226	2.12437054	0.14497233	0.89925251
FBgn003046	Coq5	-7.3926085	4.66072728	2.12372663	0.14503327	0.89925251
FBgn003517	dpr20	-5.9058906	5.46422305	2.12359436	0.14504579	0.89925251

FBgn002498	CG4293	-7.5441578	3.5547479	2.12245382	0.14515382	0.89925251
FBgn026143	SdhA	-1.7097312	9.41213566	2.12205797	0.14519134	0.89925251
FBgn003312	Cyp6u1	-6.4986472	5.45271301	2.12004242	0.14538252	0.89925251
FBgn003689	Lon	-7.8804423	4.53260916	2.11973487	0.14541172	0.89925251
FBgn003569	Sf3b6	2.45969163	7.2278897	2.11717347	0.14565514	0.89978304
FBgn002351	Pgam5	7.6694558	5.86827156	2.1085753	0.14647563	0.90237463
FBgn002898	Spn55B	6.48135276	6.24424793	2.10824164	0.14650758	0.90237463
FBgn003515	ebd1	-7.3962551	5.21787522	2.10780985	0.14654893	0.90237463
FBgn003727	eIF3f1	-5.1737342	7.03395715	2.1050601	0.14681258	0.9030239
FBgn003008	CG7766	-6.37435	5.92023431	2.1023054	0.14707723	0.90304341
FBgn002176	nudC	-6.9890805	5.92512349	2.10161727	0.14714343	0.90304341
FBgn003885	RhoGAP93B	-7.1336245	5.4662668	2.10009106	0.14729037	0.90304341
FBgn003599	CG3552	6.63514169	5.27507236	2.09802817	0.14748924	0.90329245
FBgn002990	xit	-5.7435748	6.19304283	2.09139509	0.14813075	0.90624898
FBgn003744	gzl	-4.7170261	6.25430639	2.08808841	0.14845172	0.90724029
FBgn003644	bmm	-6.4746669	4.1086373	2.08204998	0.1490399	0.90986069
FBgn026064	Antp	-7.6841936	5.90694637	2.07775774	0.14945959	0.91027894
FBgn003688	CG14100	-5.6486019	6.95483377	2.07557795	0.14967324	0.91027894
FBgn003838	CG4287	-7.6443119	5.29605698	2.07532907	0.14969765	0.91027894
FBgn002352	CG2918	-4.3061088	7.02475996	2.07483322	0.14974631	0.91027894
FBgn000031	cin	6.65258683	5.91805083	2.06846602	0.1503727	0.91220718
FBgn002216	Gpo1	-6.7161867	6.83820993	2.06836019	0.15038314	0.91220718
FBgn002995	Pdp	-6.275766	6.27487386	2.06257646	0.15095475	0.91307552
FBgn001368	mt:lrRNA	-1.0127626	16.7561517	2.06114896	0.15109621	0.91307552
FBgn026618	Vamp7	2.86917798	7.49326071	2.05896252	0.15131316	0.91307552
FBgn003166	CG8891	2.40213006	7.26876653	2.0586654	0.15134267	0.91307552
FBgn001128	Snap25	-1.8988181	10.8507848	2.05821246	0.15138767	0.91307552
FBgn002352	CG3078	-3.120955	6.79519499	2.05722248	0.15148608	0.91307552
FBgn003776	FBXO11	-6.443752	4.94642048	2.04381677	0.15282577	0.9187233
FBgn002496	GluClalpha	-4.8616895	6.36998811	2.04361382	0.15284615	0.9187233
FBgn003436	zda	1.87510516	8.04468933	2.04109049	0.15309986	0.9187233
FBgn002869	Rpn2	3.78609377	6.44271143	2.04030372	0.15317906	0.9187233
FBgn003837	Hmt-1	-5.3156164	4.71215491	2.03981925	0.15322785	0.9187233
FBgn001500	betaggt-I	6.44648518	6.06734157	2.03488946	0.15372536	0.91896757
FBgn002883	CSN6	7.18010151	6.0903891	2.03439455	0.15377541	0.91896757
FBgn003912	Ndc1	-6.4126152	5.44536604	2.03250028	0.15396713	0.91896757
FBgn001966	lncRNA:roX2	-7.0305809	6.34961984	2.02971259	0.15424978	0.91896757
FBgn005206	CG32066	3.70426998	6.44193782	2.02949016	0.15427235	0.91896757
FBgn026784	Syx7	6.17195126	6.91244604	2.02887805	0.1543345	0.91896757
FBgn003026	CDK2AP1	-7.9108253	4.34695049	2.02507113	0.15472168	0.91896757
FBgn005176	CG31760	-6.7394974	4.97160791	2.02437794	0.1547923	0.91896757
FBgn003293	Atg18b	3.59837483	6.73061777	2.02372824	0.15485852	0.91896757
FBgn005226	CG32262	-7.1418673	3.20003115	2.0235317	0.15487856	0.91896757
FBgn003138	CG4238	-5.7346267	4.2840235	2.02184092	0.15505107	0.91903581
FBgn003366	Zip48C	3.29214803	6.80085802	2.01391228	0.15586294	0.92288966
FBgn003036	Tango4	4.61345208	5.69790854	2.01058445	0.15620513	0.92358198

FBgn026495	Src42A	-5.9667194	6.69988565	2.00962967	0.15630347	0.92358198
FBgn026797	vib	-4.5395305	6.50080721	2.0059689	0.15668116	0.9248563
FBgn003176	CG9098	-7.0153657	6.11158188	2.00330096	0.15695707	0.92551418
FBgn003963	Atg14	-6.5563801	5.51678695	2.00175792	0.1571169	0.92551418
FBgn003747	Tailor	7.12131445	5.73528282	1.9987962	0.15742419	0.92614478
FBgn002560	Faf2	-6.8009791	6.17522737	1.99760039	0.15754846	0.92614478
FBgn003279	CG10132	-4.835832	5.07012449	1.98888973	0.15845702	0.93052742
FBgn005222	gogo	-6.9429096	2.47837917	1.98439848	0.1589278	0.93214001
FBgn003636	JMJD7	7.07086059	5.29350331	1.98177613	0.15920342	0.93214001
FBgn002996	CG10932	5.78382151	5.24386355	1.98160398	0.15922153	0.93214001
FBgn003149	CG17260	7.00657643	5.67286087	1.97999783	0.15939064	0.93217392
FBgn001397	Gycalpha99B	-1.6675441	8.64504891	1.97750392	0.15965361	0.93249741
FBgn003443	EndoB	-6.7469124	6.91538941	1.97637626	0.15977268	0.93249741
FBgn002498	Rilpl	-4.416721	6.89048282	1.97474872	0.15994471	0.93254793
FBgn003969	IntS11	7.17615822	6.78872347	1.96753492	0.16070976	0.93538535
FBgn003610	CG6409	5.6786266	5.84399302	1.96707088	0.16075912	0.93538535
FBgn001003	GstD3	-7.0341387	3.16477089	1.96230864	0.16126663	0.93738281
FBgn003388	CG13344	-5.6303445	6.65946923	1.95352253	0.16220777	0.94100525
FBgn004118	Tep2	5.94809441	6.33808177	1.95341297	0.16221955	0.94100525
FBgn002455	mEFTu1	-3.6291517	7.77496338	1.95058944	0.16252335	0.94181042
FBgn000438	Klp64D	-6.610084	6.94124093	1.94309064	0.16333335	0.94554438
FBgn003849	Brf	-6.6725692	4.9663835	1.94115665	0.163543	0.94579884
FBgn008258	tmod	-2.3049718	7.50470328	1.93747214	0.16394327	0.94715403
FBgn000444	Ten-m	3.45262448	6.44401416	1.93256342	0.16447827	0.94763283
FBgn003202	CG7781	-6.5791651	8.59181549	1.93015749	0.16474122	0.94763283
FBgn000438	Klp68D	-1.958398	8.20950051	1.92843527	0.16492974	0.94763283
FBgn002708	HisRS	-7.3579215	6.06828485	1.92673393	0.16511621	0.94763283
FBgn002760	CG2082	-6.1899261	7.05218509	1.92660318	0.16513055	0.94763283
FBgn005228	Drsl4	7.57492742	4.79984338	1.92122251	0.16572195	0.94763283
FBgn003147	CG2991	-2.9676247	6.77164917	1.91921891	0.16594279	0.94763283
FBgn003626	CG6910	2.63358448	11.0678973	1.91885114	0.16598337	0.94763283
FBgn003787	CG6693	-6.2726114	7.02328781	1.91795926	0.16608181	0.94763283
FBgn026757	lncRNA:CR45	-7.1657971	2.58482903	1.91604746	0.16629304	0.94763283
FBgn000539	Yp2	1.78461829	9.91870487	1.91593537	0.16630544	0.94763283
FBgn001697	snRNP-U1-70	5.61728926	5.89535781	1.91568121	0.16633354	0.94763283
FBgn002987	CG3918	-6.6392682	5.02752943	1.91198817	0.16674258	0.94763283
FBgn002966	CG14270	-5.818848	4.01765494	1.91033677	0.16692585	0.94763283
FBgn002864	aralar1	-7.0256812	5.71525092	1.91027732	0.16693246	0.94763283
FBgn003590	GstO3	7.17376466	6.81445725	1.90825566	0.16715715	0.94763283
FBgn003080	CG13001	-6.9041054	4.25550465	1.9073084	0.16726255	0.94763283
FBgn026179	snRNP-U1-C	6.82385858	5.82390441	1.90689195	0.16730892	0.94763283
FBgn002064	KrT95D	-6.9367861	6.13784702	1.90387472	0.16764526	0.94763283
FBgn001001	CanB	-1.7415744	9.0959428	1.9030099	0.1677418	0.94763283
FBgn003176	CG9107	-7.2513995	3.93693077	1.9027242	0.16777371	0.94763283
FBgn000072	for	7.93394195	3.93968758	1.90214424	0.16783851	0.94763283
FBgn004117	ago	-5.8759537	5.65498442	1.90190892	0.16786481	0.94763283

FBgn003935	RpL27	1.70689931	8.53271018	1.90060528	0.16801059	0.94763283
FBgn026085	Ykt6	6.36997788	6.98501463	1.89474839	0.16866732	0.9503979
FBgn000459	Fur2	-5.8776003	5.09991152	1.88943622	0.16926552	0.95282802
FBgn003517	CG13907	4.99502394	4.91203732	1.88013741	0.17031853	0.95781101
FBgn005315	CG33156	-6.8549388	6.26631533	1.8737476	0.17104648	0.96025926
FBgn002976	CG16756	4.73677791	5.42157033	1.87336358	0.17109034	0.96025926
FBgn002981	CG15765	-5.3744803	8.36309167	1.87047351	0.17142086	0.96116921
FBgn003130	CG4577	-5.5446586	8.20591506	1.86685256	0.171836	0.96242826
FBgn003051	CG12177	-2.5601983	6.96758798	1.86557602	0.17198263	0.96242826
FBgn004211	Cpr65Au	6.64532608	4.15799309	1.86368136	0.17220052	0.96246266
FBgn003348	dmpd	-7.3907724	4.70123181	1.86139605	0.17246376	0.96246266
FBgn026733	rn	-5.6189959	3.85407766	1.86101146	0.17250811	0.96246266
FBgn003631	Ent3	-6.0841078	6.4717599	1.85866556	0.17277889	0.96246266
FBgn000022	bsk	-6.2887667	8.22087276	1.85820722	0.17283186	0.96246266
FBgn003706	CG9391	1.74948661	8.11739221	1.85350843	0.17337589	0.9628116
FBgn003171	Oscillin	5.80381678	6.3641495	1.84889373	0.17391212	0.9628116
FBgn005391	CG33914	-5.8694939	3.30465891	1.84657855	0.17418186	0.9628116
FBgn002049	CtBP	-4.4091935	7.12564644	1.84652812	0.17418774	0.9628116
FBgn003444	cer	3.65554002	6.22916234	1.84552289	0.17430501	0.9628116
FBgn000316	put	-6.0744758	6.03092823	1.84542548	0.17431638	0.9628116
FBgn000031	cl	2.27737185	7.23622119	1.84483707	0.17438507	0.9628116
FBgn003115	bves	-6.7432883	5.93178865	1.84210474	0.17470446	0.9628116
FBgn001528	Rpt2	1.93765075	7.72904121	1.84190167	0.17472822	0.9628116
FBgn001167	Mvl	4.3834743	5.72630088	1.8411221	0.17481949	0.9628116
FBgn001522	Fer2LCH	1.41325601	10.5902065	1.84049854	0.17489253	0.9628116
FBgn002870	Nhe3	-3.305829	7.70578991	1.83774188	0.17521584	0.9628116
FBgn003324	CG8726	5.57540109	6.7272408	1.83687152	0.17531807	0.9628116
FBgn001692	Pino	-4.6663226	5.15168473	1.83507385	0.17552942	0.9628116
FBgn003136	CG31937	7.11218637	6.89125094	1.83452389	0.17559414	0.9628116
FBgn003373	Dyb	2.19760521	7.66648435	1.83313098	0.17575818	0.9628116
FBgn000292	Atpalpha	-1.4474553	10.1654645	1.83309826	0.17576203	0.9628116
FBgn003756	mRpL1	-6.1981426	7.47467982	1.83057639	0.17605948	0.96351631
FBgn005819	CG40191	-6.3459987	6.87671495	1.82840844	0.17631565	0.96397744
FBgn002656	SPARC	1.58888181	10.7241108	1.82700673	0.17648151	0.96397744
FBgn001538	drl	-6.9297047	5.35331322	1.82329028	0.17692212	0.96546031
FBgn003775	CG9467	2.92640662	6.77251439	1.81885471	0.17744966	0.96661566
FBgn026499	CG44142	7.53635045	5.6425375	1.81866278	0.17747253	0.96661566
FBgn026265	Myc	4.29767476	5.25749183	1.81664538	0.17771311	0.96700325
FBgn000327	RpL4	1.57084281	8.98059544	1.8129741	0.17815187	0.9684675
FBgn026156	Lmpt	-4.6318209	6.49472188	1.80721354	0.17884285	0.97129877
FBgn003638	CG8745	-4.2509275	5.63268704	1.80237389	0.17942577	0.97266927
FBgn002995	Rab39	-6.6717561	6.03843593	1.80228902	0.17943601	0.97266927
FBgn000346	sol	-5.098085	4.82155118	1.79985821	0.17972964	0.97281241
FBgn003616	chrb	-3.4904623	6.72303589	1.79820536	0.17992961	0.97281241
FBgn003160	CG15432	6.36411048	5.47939102	1.79586597	0.18021308	0.97281241
FBgn001061	Hmgs	-6.4143084	6.2858126	1.79535042	0.18027563	0.97281241

FBgn001172	tsr	1.48483123	9.98115893	1.79421982	0.18041286	0.97281241
FBgn003137	CG7289	4.71670146	6.06381571	1.79273525	0.18059326	0.97281241
FBgn005134	CG31345	-7.0529927	5.96977631	1.79222402	0.18065542	0.97281241
FBgn005273	Setd3	-5.7077864	3.28953909	1.78706437	0.18128425	0.97527851
FBgn003708	Syx6	-6.1913674	8.34807472	1.78488178	0.18155101	0.97543707
FBgn026139	Rpn3	-4.2600794	7.18092043	1.78264682	0.18182465	0.97543707
FBgn003277	CG17544	3.42177464	6.42371665	1.78263259	0.18182639	0.97543707
FBgn003994	CG17167	-6.672611	3.7037991	1.77648696	0.1825813	0.97796691
FBgn025084	Prosalpha6	2.17710464	7.36009336	1.7725734	0.18306392	0.97796691
FBgn002729	temp	-6.0864029	6.65644655	1.77192397	0.18314415	0.97796691
FBgn003475	CG2852	1.39181043	10.573105	1.77000621	0.18338131	0.97796691
FBgn000488	scrt	-5.2542423	6.84944313	1.76948061	0.18344637	0.97796691
FBgn003117	CG1486	-7.3635905	4.18234594	1.76838585	0.18358197	0.97796691
FBgn002605	Mhcl	3.22908813	6.39771732	1.76751184	0.18369031	0.97796691
FBgn003855	Odj	5.12025492	5.91850718	1.76616445	0.18385747	0.97796691
FBgn025914	CG42258	-6.2589105	4.98535173	1.76561047	0.18392625	0.97796691
FBgn003654	CG10516	-7.3474111	4.49994283	1.76492585	0.18401129	0.97796691
FBgn003540	Asciz	6.44329706	5.68461589	1.76170236	0.18441232	0.97889071
FBgn003333	Sec31	-4.4057555	5.91539264	1.7594981	0.18468713	0.97889071
FBgn003544	CG12012	1.97396184	7.61412653	1.75884611	0.18476851	0.97889071
FBgn002076	Spred	-6.6115382	6.5010436	1.75802467	0.18487109	0.97889071
FBgn003128	Pex12	6.66692736	4.96477226	1.75146441	0.18569274	0.98048144
FBgn003999	conu	-6.8609189	6.44029799	1.74954656	0.18593375	0.98048144
FBgn003962	CG11837	6.82770547	6.0322469	1.74923342	0.18597313	0.98048144
FBgn000123	hth	-2.2060337	7.92740653	1.74842646	0.18607467	0.98048144
FBgn003240	firl	6.40777881	5.72855338	1.74818246	0.18610539	0.98048144
FBgn003399	GPHR	-5.908563	6.66269491	1.74741406	0.18620215	0.98048144
FBgn003918	CG5746	-5.7541328	6.19836461	1.74180856	0.18690983	0.982079
FBgn005219	NUCB1	-2.5033349	8.56126721	1.74035502	0.18709385	0.982079
FBgn001552	otp	-5.9824145	5.65891493	1.73995389	0.18714467	0.982079
FBgn026124	Psa	-4.0027467	6.24715853	1.73782306	0.1874149	0.982079
FBgn003926	CG11791	2.90628444	6.6727819	1.73705631	0.18751224	0.982079
FBgn001048	NAT1	-2.6509336	6.90274764	1.73685463	0.18753786	0.982079
FBgn003540	CG11537	-4.8325324	6.60162801	1.73228257	0.18811965	0.98422272
FBgn004083	CG8012	7.11439353	4.3295214	1.72986404	0.18842826	0.98493454
FBgn003736	Atg17	-5.6379721	3.12180459	1.72444336	0.18912209	0.98696945
FBgn026691	CG32486	-2.8065279	6.81649509	1.72233374	0.18939292	0.98696945
FBgn002025	Ran	-5.419528	7.6529656	1.72079955	0.18959016	0.98696945
FBgn005185	Tap42	7.00382017	5.32854328	1.71891562	0.18983269	0.98696945
FBgn002868	Rpt3	1.53696898	8.78055927	1.71759189	0.19000332	0.98696945
FBgn004034	TRAM	5.33563117	5.23077928	1.71669873	0.19011855	0.98696945
FBgn000410	Nrt	-6.9326972	4.97008669	1.71632897	0.19016627	0.98696945
FBgn003020	PPP4R2r	-3.6112941	6.09955004	1.71606125	0.19020084	0.98696945
FBgn003819	CG3061	-5.2760825	7.83448327	1.71431293	0.19042675	0.9872442
FBgn004029	Nhe2	-7.2760849	4.6630666	1.71048682	0.19092222	0.98841951
FBgn000034	comt	-1.55234	8.90686223	1.70903252	0.19111094	0.98841951

FBgn002678	Nhe1	-6.5467332	6.1085381	1.70841358	0.19119133	0.98841951
FBgn003698	RhoBTB	-5.8488196	5.66739142	1.70722274	0.1913461	0.98841951
FBgn003043	CG4404	-7.0053282	5.43229124	1.70387299	0.19178225	0.98977676
FBgn000028	Cp190	4.00212368	5.6623876	1.69942769	0.19236284	0.990534
FBgn003734	CG1427	-4.966406	6.83395136	1.69934377	0.19237382	0.990534
FBgn001157	cpb	4.42825295	6.34327776	1.6987649	0.19244958	0.990534
FBgn003819	Cyp6d5	7.24958472	6.01585176	1.69657638	0.19273632	0.99111615
FBgn003145	Tnpo-SR	5.69667526	4.32437579	1.69237853	0.19328772	0.99244853
FBgn000404	yrt	6.46606766	3.10992231	1.6905427	0.19352945	0.99244853
FBgn025087	ttm50	6.47080614	6.34997351	1.68991966	0.19361156	0.99244853
FBgn000124	Idh	1.90397562	7.68345095	1.68840348	0.19381156	0.99244853
FBgn003681	CG14074	-6.5596323	3.78045564	1.68751584	0.19392876	0.99244853
FBgn000008	AnxB9	1.70382871	8.13906433	1.68668421	0.19403864	0.99244853
FBgn002755	CG6495	-6.860719	5.64819011	1.68528702	0.19422341	0.99250423
FBgn003491	eIF6	-4.3517619	5.31638763	1.68376095	0.19442546	0.99264805
FBgn003203	CG17294	-4.8086084	6.97253754	1.67936067	0.19500943	0.99348817
FBgn005226	CG32264	-3.4355519	7.34877307	1.67864887	0.19510408	0.99348817
FBgn003047	CG1640	-1.6599368	8.14055211	1.67858811	0.19511217	0.99348817
FBgn003931	CG10514	-6.0231203	5.86695978	1.67595118	0.19546332	0.99355346
FBgn003121	Zir	-5.274452	5.48752965	1.67522445	0.19556022	0.99355346
FBgn002898	Spt6	-7.4553639	4.95886779	1.67321153	0.19582893	0.99355346
FBgn003987	CG1896	-5.3285074	4.3107849	1.67155064	0.19605097	0.99355346
FBgn002983	MCTS1	-6.2294024	6.80905963	1.67045394	0.19619774	0.99355346
FBgn002997	mahe	2.03927913	7.45179929	1.66949722	0.19632589	0.99355346
FBgn026183	CG42764	-6.4536459	5.07510242	1.66936634	0.19634343	0.99355346
FBgn003930	CG10425	5.76704865	6.04599923	1.66612072	0.19677894	0.99430596
FBgn000558	Rab3	-4.8078824	7.66765608	1.66501024	0.19692821	0.99430596
FBgn003986	CG1792	6.43506563	5.67624866	1.66436705	0.19701472	0.99430596
FBgn002989	CG3184	-7.1998459	4.33945578	1.66195432	0.19733966	0.99506607
FBgn002038	pug	7.03759209	4.47526193	1.65992498	0.19761345	0.99556716
FBgn003101	kek5	-4.969955	4.62428103	1.657352	0.19796123	0.99643978
FBgn001181	oaf	-7.2641887	3.887783	1.65291467	0.19856269	0.99858665
FBgn002418	syd	-4.4678587	5.18882567	1.64976779	0.19899054	0.99985739
FBgn005049	Rpe	3.33864263	6.29417948	1.64566918	0.19954941	1
FBgn003178	WDR79	-6.4406906	6.00978446	1.64453598	0.19970425	1
FBgn002640	Noa36	-4.2018715	6.45170663	1.64238703	0.19999827	1
FBgn002308	fray	-6.1691695	4.43806323	1.64025611	0.20029033	1
FBgn003017	CG2972	3.58763103	6.04089057	1.63538546	0.20095978	1
FBgn003321	U2A	-6.2302594	4.41683229	1.6321713	0.20140299	1
FBgn003789	CG5214	-1.4783281	8.84199996	1.63139519	0.20151018	1
FBgn003327	RagC-D	-3.4700684	6.45150994	1.63031859	0.20165899	1
FBgn003492	CG13562	6.79793962	5.65021249	1.63023632	0.20167037	1
FBgn003746	CG1105	-5.9691717	6.39543491	1.62765554	0.20202763	1
FBgn002076	Alas	-5.479521	5.06245236	1.62346861	0.20260882	1
FBgn003438	Cyp12b2	-6.9788076	4.58169351	1.62301557	0.20267182	1
FBgn003131	Tango14	6.96165911	4.91571238	1.61864633	0.20328064	1

FBgn001487!	Set	-4.7339222	6.23550214	1.61509383	0.20377724	1
FBgn000461!	GluRIA	-5.383206	5.70028231	1.61020559	0.20446291	1
FBgn002761(	Ythdc1	-6.427587	5.55337629	1.60958374	0.20455033	1
FBgn003836(	CG9590	-6.2012613	6.14821634	1.60943216	0.20457164	1
FBgn003010!	CG12121	3.39474497	6.18530203	1.60917061	0.20460843	1
FBgn001577	nrv2	-1.3552348	10.3411159	1.60871002	0.20467323	1
FBgn003528:	CG12024	-6.6853232	6.38113611	1.60761095	0.20482795	1
FBgn003528	CG13937	-3.4156091	6.9154863	1.60671631	0.204954	1
FBgn026288	lncRNA:CR43	3.75013847	5.8858498	1.60349506	0.20540861	1
FBgn001043:	emp	6.90251008	5.8253436	1.60263838	0.20552972	1
FBgn003648:	FucTA	-5.8194667	6.07356408	1.60164444	0.20567033	1
FBgn003943(	CG5455	-6.6269069	5.59422277	1.60010588	0.20588821	1
FBgn026074:	GC1	-6.5450932	5.67444235	1.59286846	0.20691679	1
FBgn003688:	CG9330	-4.77055	6.24244079	1.59200539	0.20703985	1
FBgn002197!	l(2)k09913	-1.63454	8.21732352	1.59179359	0.20707007	1
FBgn003431:	CG5721	6.8746605	6.33854254	1.58907751	0.20745798	1
FBgn003212!	jp	-5.2241339	4.88962856	1.58706487	0.20774598	1
FBgn001996:	Khc-73	-4.7410984	5.48835439	1.5846921	0.20808613	1
FBgn000329:	ru	-7.0574384	3.98892602	1.58446773	0.20811832	1
FBgn000441!	me31B	-3.1000309	7.89739372	1.58260212	0.20838628	1
FBgn006244(	EMRE	-6.1696273	7.26119623	1.57980715	0.20878847	1
FBgn026708:	lncRNA:CR45	6.29583487	4.06792665	1.57803093	0.20904455	1
FBgn002054:	kraken	-5.0743294	6.24999022	1.57801377	0.20904703	1
FBgn003980	Npc2h	7.05389592	4.5929868	1.57308594	0.20975943	1
FBgn003331	CG8635	-6.3885618	6.53915042	1.57195768	0.20992294	1
FBgn002432!	Mekk1	-6.6223336	2.67690493	1.57041993	0.21014604	1
FBgn003692(	CG8004	-6.0325495	6.51242333	1.56962264	0.21026182	1
FBgn026739(	dop	-5.3179334	5.08486046	1.56935095	0.2103013	1
FBgn003699(	mRpL15	-6.357213	5.50648492	1.5690861	0.21033978	1
FBgn000490	14-3-3zeta	-1.321551	10.5888091	1.56907597	0.21034126	1
FBgn003060:	Lsd-2	3.53106706	6.13283344	1.56884818	0.21037436	1
FBgn005197	CG31974	-3.2111219	6.21257716	1.56812832	0.21047904	1
FBgn003034	p24-1	2.02272566	7.38782639	1.56697986	0.21064615	1
FBgn003163	mxt	-4.4228275	5.92891868	1.56561293	0.21084527	1
FBgn003534:	CG16758	3.25913086	6.09763877	1.56467863	0.21098149	1
FBgn000004(	Act87E	3.23450795	6.13168314	1.55989363	0.2116808	1
FBgn026287:	milt	-4.4543299	5.16226462	1.55809516	0.21194434	1
FBgn028364!	elgi	-6.2957478	6.11430082	1.55570362	0.2122954	1
FBgn003682:	CG6841	2.88816919	6.47781258	1.55167691	0.21288805	1
FBgn003067(	Pis	-5.3146409	8.22505315	1.55026346	0.21309655	1
FBgn026049!	qvr	-1.8848939	8.47681761	1.54844965	0.21336446	1
FBgn003162!	Clect27	-6.5346998	3.37314047	1.54575876	0.21376266	1
FBgn002840	Drep3	-6.4924572	3.74045277	1.5448345	0.21389963	1
FBgn004211	CG18766	-5.9295789	7.11533974	1.54408534	0.21401073	1
FBgn002909:	cathD	1.35007651	10.9416308	1.54316443	0.2141474	1
FBgn003706(	CG10508	-3.9340037	5.77488211	1.5420508	0.21431281	1

FBgn003085	CG8289	-2.2713217	7.06855645	1.54116568	0.21444438	1
FBgn002090	Scp2	-4.2895057	7.86443805	1.54035883	0.2145644	1
FBgn003295	CG2225	-4.6038595	4.41074918	1.53873308	0.21480648	1
FBgn005320	dpr11	-6.6364181	3.32143287	1.53861507	0.21482407	1
FBgn001698	smid	-7.0463641	3.71134924	1.53768857	0.21496219	1
FBgn003052	CG11103	-5.9937619	5.23920078	1.53686309	0.21508533	1
FBgn003668	CG7728	3.35936129	6.75566893	1.53654927	0.21513217	1
FBgn001998	mGluR	-7.2627908	5.52627302	1.53437682	0.21545675	1
FBgn026079	Gprk1	-6.1584376	8.44961678	1.5331877	0.21563466	1
FBgn003313	Tsp42Ek	-5.3989913	4.32624233	1.53300613	0.21566184	1
FBgn002586	Crag	-7.1358484	3.58214541	1.53129689	0.21591791	1
FBgn003035	CG10362	-4.1464011	5.31106405	1.5303329	0.21606249	1
FBgn002581	Mgstl	6.9992353	4.106648	1.52922579	0.21622868	1
FBgn003419	CG11400	2.8473397	6.54375386	1.52850912	0.21633634	1
FBgn001394	Elal	2.70121982	6.64805288	1.52546172	0.21679484	1
FBgn003929	Sil1	-4.7851139	5.319298	1.52206516	0.21730724	1
FBgn003986	CG1815	-4.5652744	4.39889541	1.51982007	0.21764672	1
FBgn000408	Dhfr	6.85242927	4.57953587	1.51591542	0.21823866	1
FBgn002352	CG2924	-6.8026542	5.95363235	1.51558275	0.21828918	1
FBgn001522	Fer1HCH	1.24660799	11.3087745	1.50993935	0.21914834	1
FBgn005110	TTLL5	-5.1029884	5.09465795	1.50868608	0.21933969	1
FBgn026126	plx	-6.1582689	5.91609681	1.50768168	0.21949318	1
FBgn002756	cert	-5.6103888	5.38325744	1.50744776	0.21952895	1
FBgn003418	Ehbp1	-4.6843819	5.42328647	1.50672818	0.21963902	1
FBgn003067	CG8191	6.57955214	4.78650495	1.50459846	0.21996517	1
FBgn026393	dally	-4.1424498	5.28210327	1.50442219	0.21999219	1
FBgn001377	Cyp6a9	3.84370702	5.3111047	1.50175975	0.22040079	1
FBgn002429	Spn43Ab	-5.6463371	6.47337384	1.50132835	0.22046709	1
FBgn002869	Rpn12	2.34825313	6.89966045	1.49924546	0.2207875	1
FBgn001053	Ccs	6.10858689	5.2221127	1.49630887	0.22124018	1
FBgn003336	CG13743	2.57244806	6.63213353	1.49618506	0.22125929	1
FBgn002583	RhoGAP1A	-5.2128195	3.80803506	1.49528999	0.2213975	1
FBgn003496	CG3163	-6.4104802	5.92673797	1.4934589	0.22168057	1
FBgn000567	Vha55	-1.3478804	9.51959549	1.49088812	0.22207871	1
FBgn003843	Der-2	6.37083336	5.26477196	1.48789296	0.22254367	1
FBgn003533	CG1317	-5.327647	6.36025451	1.48736937	0.22262507	1
FBgn026429	Det	6.82378356	4.83380078	1.48069027	0.22366656	1
FBgn005172	CG31729	2.20122848	7.01449196	1.47700812	0.22424322	1
FBgn026345	nwk	-5.7468627	5.57986313	1.47666268	0.22429741	1
FBgn005392	CG33926	7.39031461	4.50462825	1.47559989	0.22446423	1
FBgn003699	CG5910	-6.7922707	4.15713341	1.4751598	0.22453336	1
FBgn003812	Ravus	3.33020729	6.14881045	1.4716185	0.2250905	1
FBgn003502	CG11414	-5.9940242	6.26247002	1.46912881	0.2254832	1
FBgn002710	Inx2	-3.4307467	5.78608601	1.46847453	0.22558653	1
FBgn003472	babos	-6.0259345	5.19936427	1.46691555	0.22583298	1
FBgn003041	CG4004	6.50566547	5.86390936	1.46678338	0.22585389	1

FBgn004057	CG17193	-6.5463224	5.97526305	1.46658363	0.22588549	1
FBgn003613	FoxK	-5.6955136	6.11505735	1.46390172	0.22631034	1
FBgn003164	CG3036	5.99282144	5.55867052	1.4638377	0.22632049	1
FBgn003151	CG9664	7.07005738	4.08186768	1.46323105	0.22641673	1
FBgn003497	Gpat4	-2.707868	6.61931068	1.46007768	0.22691776	1
FBgn005008	CG30089	-7.1375557	3.67358733	1.45082327	0.2283959	1
FBgn000118	Hex-A	-1.5187322	8.18413987	1.45015534	0.22850303	1
FBgn003345	Sting	1.98728117	7.32265703	1.45006152	0.22851808	1
FBgn003673	CG7564	-6.7470937	5.7602336	1.44931733	0.22863752	1
FBgn003713	Syn1	-5.819709	4.64038831	1.44797438	0.22885326	1
FBgn003926	Ythdf	-6.5863498	5.33529701	1.4468138	0.2290399	1
FBgn001182	Pp2B-14D	-5.4706852	5.86048497	1.44564105	0.22922867	1
FBgn026597	ttv	-6.9824144	5.31603896	1.44423642	0.22945502	1
FBgn003167	CG31650	-6.5816641	5.59489722	1.44360196	0.22955735	1
FBgn003098	Obp18a	6.93376591	5.26486714	1.44244347	0.22974434	1
FBgn003003	CG10555	-5.0766675	4.45397621	1.44157802	0.22988415	1
FBgn003080	Rcp	6.21618762	4.94603653	1.44146293	0.22990275	1
FBgn003110	Syx16	-5.3257074	6.99522995	1.4412574	0.22993597	1
FBgn003953	CG5590	1.67934447	8.06324375	1.43950788	0.23021899	1
FBgn003346	CG1513	-6.6263804	5.86350049	1.43803149	0.23045815	1
FBgn003990	Hcf	-3.3062153	6.00910299	1.43629363	0.23074006	1
FBgn003976	CG2218	-6.913257	5.15629068	1.43593874	0.23079767	1
FBgn000564	Rox8	5.54205932	5.39148477	1.43576679	0.2308256	1
FBgn008538	shakB	-4.7143776	6.56509206	1.43318945	0.23124462	1
FBgn000286	MtnA	1.39792766	8.80640671	1.4306152	0.23166406	1
FBgn005348	dpr7	-5.1519925	8.22850333	1.42959497	0.23183055	1
FBgn003535	CG16986	-6.2921374	6.40492896	1.42650842	0.23233511	1
FBgn004178	SCAR	-6.0120968	5.97603556	1.42607501	0.23240607	1
FBgn026359	Lpin	-4.1254777	6.76175042	1.42307258	0.23289833	1
FBgn002535	Mtpbeta	-6.1224753	5.313671	1.4214207	0.2331697	1
FBgn003068	mRpL3	-6.1092778	6.33601716	1.41690093	0.23391417	1
FBgn026393	ebi	-6.8758118	5.97963107	1.41680083	0.23393069	1
FBgn001171	Sep-01	6.31582373	6.15341463	1.4162088	0.23402843	1
FBgn003723	Sfxn1-3	1.58850504	8.0330576	1.41246573	0.2346475	1
FBgn003550	Teh2	-7.0263624	5.65333543	1.41232611	0.23467063	1
FBgn003778	CAH7	-2.7747033	7.81389115	1.40915357	0.23519696	1
FBgn003909	GILT3	-5.109437	5.10978021	1.40781392	0.23541964	1
FBgn003199	SLC5A11	-6.3824036	4.06104517	1.40700646	0.23555397	1
FBgn003008	Arfrp1	-6.218729	5.50079494	1.40512763	0.23586692	1
FBgn005312	CG33129	-2.4756019	7.72185671	1.40464687	0.23594708	1
FBgn003435	GstE11	4.49295913	4.61339058	1.40302657	0.23621748	1
FBgn003443	mip40	6.5578364	6.54976578	1.40120211	0.2365224	1
FBgn000011	Argk	-1.1182771	12.4655951	1.39842025	0.23698825	1
FBgn003055	mRpL38	-3.832947	6.65919097	1.39723036	0.23718785	1
FBgn003476	RYBP	6.11610745	5.8128238	1.39412232	0.23771017	1
FBgn003222	CG5037	-6.5303398	5.93711975	1.38938835	0.23850842	1

FBgn003747[illegible]	CG10068	6.86994807	4.24882161	1.38911717	0.23855425	1
FBgn004058[illegible]	CG5791	7.11407029	5.82278885	1.38900492	0.23857322	1
FBgn003168[illegible]	CG5828	3.69874615	5.45733735	1.38696973	0.23891751	1
FBgn003338[illegible]	Hydr1	-3.6647814	5.38528905	1.38584183	0.23910857	1
FBgn003583[illegible]	CG7550	-5.9338259	6.1160938	1.38558584	0.23915196	1
FBgn000121[illegible]	Hrb98DE	-2.8283583	6.72048217	1.38429472	0.23937095	1
FBgn003086[illegible]	Socs16D	3.60866724	5.7305044	1.38402557	0.23941664	1
FBgn002987[illegible]	Marf	-2.2289124	7.25966141	1.38362326	0.23948494	1
FBgn003353[illegible]	CG11883	-5.7565703	5.74616922	1.3831455	0.23956608	1
FBgn002093[illegible]	Dgkepsilon	-6.1173114	5.89008355	1.38195197	0.23976894	1
FBgn004134[illegible]	Pcyt1	-3.7327415	6.63578801	1.38144883	0.23985451	1
FBgn001669[illegible]	Pitslre	-3.2865033	6.60183921	1.37650477	0.24069741	1
FBgn003787[illegible]	Tctp	1.39069224	9.07268074	1.37646236	0.24070466	1
FBgn001369[illegible]	mt:tRNA:Ile-	4.99826	2.29694756	1.37327381	0.24125019	1
FBgn026075[illegible]	CG42554	-6.3891627	5.46609627	1.37306178	0.24128652	1
FBgn008525[illegible]	CG34228	-6.5090041	4.81580302	1.37092262	0.24165343	1
FBgn026322[illegible]	Hk	-3.1990946	6.995228	1.37015663	0.24178498	1
FBgn003497[illegible]	CG3257	-1.7798953	8.01582206	1.36818113	0.24212464	1
FBgn003701[illegible]	CG4042	-4.9758418	5.40333417	1.36639991	0.24243139	1
FBgn003921[illegible]	CG6695	-4.5826901	3.56080572	1.36603965	0.24249349	1
FBgn002759[illegible]	CG17712	6.0515146	5.77984573	1.3649863	0.24267518	1
FBgn003923[illegible]	sosie	-6.0734398	5.15163731	1.36492317	0.24268607	1
FBgn001032[illegible]	Tbh	-5.2404841	4.85252738	1.36304268	0.24301085	1
FBgn003418[illegible]	CG9646	-3.1276798	6.87664031	1.36142095	0.24329137	1
FBgn028424[illegible]	Adf1	2.21484885	7.00926809	1.35820296	0.24384917	1
FBgn008539[illegible]	CG34367	-4.0372112	4.99655829	1.35751364	0.24396886	1
FBgn004091[illegible]	schlank	-6.6543755	5.27006056	1.35672654	0.24410561	1
FBgn026040[illegible]	elav	-4.2671923	5.09430669	1.35614483	0.24420673	1
FBgn003625[illegible]	RhoGAP68F	-6.4357914	4.84581991	1.35415	0.24455392	1
FBgn003394[illegible]	Had2	6.91342888	8.88001182	1.35397284	0.24458478	1
FBgn003381[illegible]	CG4676	-6.5143524	5.69082818	1.35162968	0.24499342	1
FBgn003272[illegible]	CG10623	-6.5956983	4.76519335	1.35143734	0.245027	1
FBgn003880[illegible]	CG16953	-5.095801	6.19388108	1.35028941	0.24522753	1
FBgn002587[illegible]	wrapper	2.94212306	6.57098752	1.3494741	0.24537008	1
FBgn003953[illegible]	unc80	-4.0516459	5.60647523	1.34491655	0.24616878	1
FBgn003650[illegible]	CG7739	-4.5206061	7.38389113	1.34472621	0.24620221	1
FBgn008677[illegible]	nAChRalpha7	-6.342009	6.92173431	1.34350086	0.24641753	1
FBgn003523[illegible]	CG13917	-5.4498803	6.23821498	1.3425643	0.24658225	1
FBgn006349[illegible]	GstE2	-1.7765266	7.62499366	1.34243304	0.24660535	1
FBgn008670[illegible]	pix	-2.5843041	7.35821868	1.34192587	0.24669462	1
FBgn003239[illegible]	Hacd1	2.23803772	6.85593232	1.34172116	0.24673067	1
FBgn003702[illegible]	CG3618	-6.0181648	6.8112266	1.34006935	0.24702174	1
FBgn000416[illegible]	5-HT1A	-4.2078445	5.11889254	1.33897161	0.24721541	1
FBgn001537	chn	-3.5320174	6.00107407	1.33890333	0.24722746	1
FBgn002637[illegible]	RhoGAPp190	5.27577811	5.03385549	1.33685855	0.24758873	1
FBgn026640[illegible]	CG45049	-6.1603184	6.54865574	1.33598106	0.24774396	1

FBgn026485	AP-2alpha	1.66238479	7.68876536	1.33515824	0.24788963	1
FBgn005320	CG33203	-6.2025543	4.8947296	1.33512282	0.2478959	1
FBgn003544	CG14971	-5.7189056	6.37508635	1.33055799	0.24870598	1
FBgn000374	Treh	-2.2977143	6.83049739	1.33035549	0.24874199	1
FBgn003190	CG5149	5.91965895	6.19846744	1.32913236	0.24895963	1
FBgn002181	Vps28	1.6345881	7.71056743	1.32792973	0.24917386	1
FBgn008396	Nlg3	-5.055791	5.86095921	1.32461993	0.2497646	1
FBgn000314	pr	6.70976906	6.30662241	1.32399142	0.24987697	1
FBgn000440	Pep	-1.5577109	8.65860876	1.32379073	0.24991286	1
FBgn002976	SPR	-6.4884568	5.52804804	1.32214241	0.25020792	1
FBgn003755	CG9667	2.75638018	6.59320452	1.32193162	0.25024568	1
FBgn004073	CG16836	3.56701749	5.67769891	1.32171366	0.25028473	1
FBgn002236	Pur-alpha	1.98465241	8.5620343	1.3213488	0.25035013	1
FBgn003948	CG6066	5.97917347	4.70235062	1.32086956	0.25043605	1
FBgn003479	asrij	-6.3543787	6.06212556	1.320658	0.25047399	1
FBgn003893	CG7084	-4.1378589	5.59747176	1.31865375	0.2508338	1
FBgn003308	PGAP3	-5.9552123	6.50704618	1.31836921	0.25088494	1
FBgn003711	ORMDL	6.24423582	6.29337925	1.31655004	0.25121215	1
FBgn001989	Smg5	-4.8990431	5.43029379	1.31429028	0.25161934	1
FBgn003577	Sh3beta	2.40900405	6.77062502	1.31372082	0.25172209	1
FBgn003988	RhoGAP100F	-4.906726	5.74696313	1.31372018	0.2517222	1
FBgn002277	Oat	-4.8412941	4.27476328	1.31094388	0.25222383	1
FBgn003909	GILT2	-3.8821032	5.03008756	1.31015135	0.25236725	1
FBgn025916	mnb	4.85735982	6.17878506	1.30804339	0.25274922	1
FBgn004053	CG8369	1.29273345	9.44148059	1.30573425	0.25316845	1
FBgn003629	CG10638	6.6029041	6.05228038	1.30567704	0.25317885	1
FBgn000405	z	3.45788897	5.92975959	1.30227538	0.25379802	1
FBgn003379	GLaz	5.92259869	5.44929847	1.30217729	0.2538159	1
FBgn002638	Prosbeta3	1.39233882	8.52917295	1.30201448	0.25384558	1
FBgn003153	Pgant2	-6.0618629	5.40684086	1.30014819	0.25418616	1
FBgn003343	Mmp2	6.32891668	5.79894538	1.29866014	0.25445811	1
FBgn003342	CG12929	-6.0255099	5.59599795	1.29537899	0.25505903	1
FBgn003653	DCP2	-4.8786451	5.96027879	1.29495149	0.25513746	1
FBgn000110	Galphai	3.82434691	5.67119722	1.29157416	0.25575806	1
FBgn003028	GCS1	2.7532082	6.65125272	1.29124307	0.255819	1
FBgn000120	Hn	5.15516436	5.36507966	1.29108541	0.25584802	1
FBgn000350	Btk29A	5.61995907	4.78242549	1.28910483	0.25621299	1
FBgn026460	Fife	-5.6451867	5.66554398	1.28748292	0.25651234	1
FBgn001133	vers	-6.2933939	5.9276019	1.28577512	0.25682801	1
FBgn002988	CG3198	-3.3258436	5.61627713	1.28328612	0.25728893	1
FBgn026278	Mdh1	1.23817814	9.88979705	1.2829458	0.25735203	1
FBgn003773	Pnn	6.05225005	6.01165892	1.28280222	0.25737866	1
FBgn003723	CG9795	-4.3560317	4.21431633	1.27961506	0.25797062	1
FBgn002090	Scp1	-6.4011175	4.25872615	1.27676536	0.25850133	1
FBgn003233	Wdr59	-4.2436454	4.67006004	1.27299358	0.25920584	1
FBgn003963	alpha-Man-II	-2.9408827	6.34727747	1.27269178	0.25926231	1

FBgn003240	CG6712	-6.1317156	5.08041771	1.27250312	0.25929762	1
FBgn003274	CG10470	2.13420266	7.0252598	1.27130442	0.25952212	1
FBgn003498	Naa35	-6.1068664	4.81966535	1.27084625	0.25960798	1
FBgn005167	CG31674	3.76588314	4.90974901	1.27079831	0.25961697	1
FBgn004576	CHKov1	6.70092801	4.34652019	1.27044501	0.25968321	1
FBgn026063	gammaTub2	4.4980996	6.16439961	1.26830261	0.26008535	1
FBgn003119	CG12576	1.51890822	7.97192807	1.26770795	0.26019711	1
FBgn026429	Cyt-b5	1.38458319	8.69014829	1.26648853	0.26042646	1
FBgn026086	Trs23	6.45865734	5.27323781	1.2644806	0.26080467	1
FBgn003905	Cow	-2.3751426	7.6604925	1.26311261	0.26106274	1
FBgn003009	fend	-3.1385389	6.57514977	1.26161503	0.26134561	1
FBgn028681	SRPK	-1.9186735	7.27244425	1.25999946	0.26165119	1
FBgn001998	Gs1l	-1.5952749	7.76293415	1.25914284	0.2618134	1
FBgn008540	smal	-5.5277007	5.41044195	1.25475849	0.26264557	1
FBgn003609	CG8009	-4.8685617	6.18377174	1.25455734	0.26268383	1
FBgn002708	ThrRS	-5.4612742	7.18828838	1.25437895	0.26271777	1
FBgn003971	CG15514	5.92716221	5.67666213	1.25203424	0.2631643	1
FBgn003502	CG13585	1.46324644	8.13241178	1.25055364	0.26344676	1
FBgn002455	Dph5	5.90625804	5.77746435	1.25027154	0.26350062	1
FBgn003395	jef	-3.1071198	6.23401336	1.24928278	0.26368951	1
FBgn001579	Rab6	1.68369685	8.00855725	1.24833678	0.26387038	1
FBgn002868	Rpn6	1.42221732	8.10744685	1.24618922	0.26428157	1
FBgn003274	Sidpn	-4.3871931	6.23661634	1.24360731	0.26477697	1
FBgn005217	Ndfip	-4.8858135	6.44046837	1.24284635	0.2649232	1
FBgn003765	Kdm2	3.70276874	6.07830281	1.24228271	0.26503157	1
FBgn003347	CG12129	6.31826895	6.03228519	1.24155973	0.26517067	1
FBgn003069	CG15602	6.10062592	6.2424573	1.24088808	0.26529997	1
FBgn003119	CG17598	-6.8632079	3.83564162	1.23748655	0.26595601	1
FBgn001606	side	-4.4739871	5.03945963	1.23727948	0.26599601	1
FBgn003392	Arc1	1.18561377	11.1405563	1.23707719	0.2660351	1
FBgn001081	unc-45	2.53038448	6.6626783	1.23342875	0.26674126	1
FBgn002982	Shmt	-4.1857184	5.57672143	1.23341345	0.26674423	1
FBgn003546	PIG-B	-6.0285393	3.48694126	1.23336686	0.26675326	1
FBgn003175	lid	-3.9784732	4.95119702	1.23262714	0.26689673	1
FBgn008677	step	-6.2752707	5.91914209	1.23222114	0.26697552	1
FBgn003696	SCCRO4	4.56984164	5.35992968	1.23085314	0.26724119	1
FBgn002587	Timp	1.41600305	8.50738268	1.23051002	0.26730788	1
FBgn003429	CG5773	3.25836226	6.05459487	1.23007788	0.26739189	1
FBgn003889	Archease	2.53133633	6.47221707	1.23003723	0.2673998	1
FBgn003147	CG3104	-5.7333586	6.47860719	1.22850878	0.26769724	1
FBgn003164	CG3008	-3.7532342	5.6457447	1.22761091	0.26787216	1
FBgn003069	CG8578	5.5452345	5.87890207	1.22728439	0.26793581	1
FBgn003132	Tgt	-6.1245076	5.66526273	1.22720299	0.26795168	1
FBgn003211	FucTB	3.29174493	6.43669122	1.22570158	0.26824461	1
FBgn003992	PIP4K	-6.0249761	7.0360737	1.22386423	0.26860363	1
FBgn003415	CG5522	-4.9424012	4.7142348	1.22333832	0.2687065	1

FBgn001607	wun	-6.7171135	5.86914918	1.22244157	0.26888203	1
FBgn003247	CG9928	3.05878645	6.06306912	1.2209192	0.26918033	1
FBgn008690	CG31751	5.28231492	6.62178936	1.21994077	0.26937228	1
FBgn002842	Jhl-21	-5.8376276	6.30580598	1.21785704	0.26978162	1
FBgn004023	bor	-4.6709854	6.46729903	1.2173643	0.26987853	1
FBgn028425	Ttd14	-3.6563727	6.52227693	1.21688504	0.26997283	1
FBgn003984	CG11334	6.08101874	5.68423677	1.21614718	0.2701181	1
FBgn003962	CG1951	-5.9469998	5.30035501	1.21302667	0.27073352	1
FBgn001376	Cdk5	-5.8962276	5.52995165	1.21073732	0.27118615	1
FBgn005019	CG30197	1.31338496	8.51856378	1.20965465	0.27140053	1
FBgn001028	Taf4	-6.4643051	3.3665085	1.20833888	0.27166135	1
FBgn026576	zyd	-4.8676659	6.30292257	1.20520852	0.27228314	1
FBgn000443	Galphaq	5.34431206	5.51808247	1.20489328	0.27234586	1
FBgn003846	CG17556	-6.2428951	4.64429874	1.20397772	0.27252811	1
FBgn026546	Traf6	-4.1915629	4.89999442	1.20344886	0.27263345	1
FBgn003946	CG6330	-4.9055534	6.29720773	1.20255622	0.27281137	1
FBgn003701	CG4825	5.12113258	6.17057681	1.20229543	0.27286338	1
FBgn008700	apolpp	1.4422719	8.15439894	1.20191833	0.2729386	1
FBgn000291	na	-5.9248193	3.02527109	1.20088062	0.27314574	1
FBgn001078	l(3)02640	-6.3312987	5.19562831	1.20033999	0.27325374	1
FBgn005136	Jupiter	1.27999661	8.9787666	1.198909	0.27353984	1
FBgn001042	TfIIS	5.69940207	6.25427951	1.19756545	0.27380881	1
FBgn003523	Pex10	5.79828419	5.38338556	1.19718915	0.2738842	1
FBgn003924	CG11168	-3.5849721	5.05206873	1.19667594	0.27398707	1
FBgn003050	tth	3.14148518	6.04728514	1.19538151	0.27424673	1
FBgn003993	Gyf	-3.4369632	6.35728394	1.19276086	0.27477337	1
FBgn025999	CG42489	6.75791463	4.55230005	1.19231153	0.27486379	1
FBgn000197	stc	-4.8538139	3.70736244	1.19130946	0.27506558	1
FBgn002455	flfl	-5.2980922	5.50311829	1.19117659	0.27509235	1
FBgn003400	mRpL41	-5.9386542	6.15524923	1.18897901	0.2755356	1
FBgn003523	CG12004	2.12056454	7.17503457	1.18832092	0.27566851	1
FBgn002995	CG12155	-5.583086	5.65977636	1.18807253	0.2757187	1
FBgn008690	sls	4.98476606	5.06614221	1.18659738	0.27601698	1
FBgn028368	Ge-1	-4.6121336	4.23595683	1.18625184	0.27608691	1
FBgn004001	CG17493	-1.7171242	7.47038347	1.18615396	0.27610672	1
FBgn003969	CG7789	-6.1011392	6.12035428	1.18264807	0.27681756	1
FBgn008538	bma	-6.2932114	4.03736787	1.17991492	0.27737332	1
FBgn003880	CG5191	-6.2750865	5.38420215	1.17852681	0.27765611	1
FBgn002582	JTBR	-5.7848165	5.07277787	1.17845561	0.27767063	1
FBgn002663	nes	6.42093695	5.47822471	1.17708414	0.27795041	1
FBgn003041	CG2540	-5.2503918	5.42571511	1.1738415	0.27861332	1
FBgn003858	CG14314	-5.3373109	6.54492535	1.17327709	0.27872891	1
FBgn026377	fok	-3.3628474	6.86505743	1.17326866	0.27873064	1
FBgn025917	corn	-3.6297503	5.23367756	1.17213041	0.27896393	1
FBgn003223	dpr19	-6.2353779	6.56522141	1.1719602	0.27899884	1
FBgn002971	CG11444	1.66973137	7.54156727	1.17181588	0.27902844	1

FBgn003283	CG16772	-4.9177386	5.51349381	1.17156061	0.27908081	1
FBgn002868	Rpn7	-5.769696	6.49217285	1.17085409	0.27922582	1
FBgn005161	CG31612	-6.1485957	3.43985347	1.17064369	0.27926902	1
FBgn001121	blw	-1.1250218	10.8429786	1.17059871	0.27927826	1
FBgn000388	alphaTub84D	-1.5830819	8.46288439	1.1668272	0.28005413	1
FBgn003627	Sms	-6.3588657	5.41408216	1.16597115	0.28023061	1
FBgn026492	smt3	1.22385956	9.39574697	1.16582442	0.28026087	1
FBgn028678	Etf-QO	3.44614189	5.45232943	1.16500607	0.28042974	1
FBgn002687	CG13364	1.33954772	8.32200638	1.1643316	0.28056901	1
FBgn026286	S6kII	-5.5316709	4.71111217	1.16403254	0.28063079	1
FBgn002235	Tsf1	1.25644368	11.1562599	1.16313465	0.28081639	1
FBgn005217	Krn	-5.9202702	3.47879005	1.16311338	0.28082079	1
FBgn001022	Nmdmc	-4.9344844	5.02428413	1.16197314	0.2810567	1
FBgn002656	CG1307	-6.2019662	6.21028994	1.16181866	0.28108869	1
FBgn003294	nrv3	-1.6122566	11.0355216	1.16136686	0.28118225	1
FBgn003104	CG12237	-5.488211	6.85475502	1.16090808	0.28127729	1
FBgn008521	CG34183	6.53557977	4.50254831	1.15439985	0.28263	1
FBgn002762	Pfrx	-4.221876	4.52772713	1.15402206	0.28270877	1
FBgn002209	Vha36-1	-1.3913502	8.49043459	1.1536833	0.28277943	1
FBgn003849	beat-IIb	-5.759756	4.79473479	1.15367026	0.28278215	1
FBgn003219	CG5708	-6.3077929	6.33843182	1.15330094	0.28285921	1
FBgn003074	CG9917	1.6511973	7.47145846	1.15053697	0.28343677	1
FBgn026370	CG43658	-4.4628977	3.26712733	1.15047146	0.28345048	1
FBgn004033	CG3021	-5.7612673	5.61066287	1.15006247	0.28353607	1
FBgn000063	CG17716	-4.6589872	5.47701208	1.14902961	0.28375238	1
FBgn003297	CG3651	-6.6939755	5.89023008	1.14888384	0.28378292	1
FBgn026079	ctrip	3.37692528	5.16452724	1.14633114	0.28431849	1
FBgn003869	unc79	-3.5817566	5.27001037	1.14513678	0.28456951	1
FBgn003511	thoc7	-4.2571984	6.82064157	1.14464509	0.28467293	1
FBgn003324	ACC	-4.366449	5.01242826	1.14264668	0.28509376	1
FBgn003259	Prosbeta4	1.87875903	7.07014754	1.14166124	0.28530157	1
FBgn003058	CG14408	-6.8754572	4.09821491	1.14157222	0.28532036	1
FBgn002989	CG14440	-4.0226494	5.13183136	1.14109101	0.28542191	1
FBgn001021	alpha-Cat	-1.3762397	8.1196836	1.14098502	0.28544428	1
FBgn003624	CG11560	6.13343145	5.69950247	1.13815457	0.28604261	1
FBgn002437	E2f2	2.0566148	6.84931559	1.13775481	0.28612725	1
FBgn003552	Ctl1	2.05957617	6.87164528	1.13501692	0.28670775	1
FBgn026216	CG9932	3.90267919	5.93466369	1.13223209	0.28729973	1
FBgn000259	RpS5a	1.30150678	8.55151529	1.13113076	0.28753427	1
FBgn026313	mEFG1	3.90632896	5.48464812	1.1299042	0.28779577	1
FBgn003842	CG14894	-5.7930846	6.23040325	1.12667483	0.28848572	1
FBgn003525	CG7970	5.89830538	5.36889765	1.12604959	0.28861954	1
FBgn003676	CG5567	6.03271525	3.5409182	1.12563111	0.28870915	1
FBgn003015	CG9689	1.42276384	8.796464	1.1204895	0.28981308	1
FBgn002855	gammaSnap	-1.6051174	7.68950325	1.12002371	0.28991335	1
FBgn003882	CG17271	5.77132627	5.83730672	1.11870359	0.29019778	1

FBgn000116	h	5.48636472	5.79616428	1.11715804	0.29053123	1
FBgn001176	Fdh	3.94434197	6.33900605	1.11563034	0.2908613	1
FBgn002761	Dic1	-3.3040033	6.71199776	1.11507342	0.29098175	1
FBgn003627	Ncc69	-3.7212336	5.70563586	1.11379655	0.29125815	1
FBgn006608	RpL41	1.24170769	9.22628653	1.11256926	0.29152413	1
FBgn000127	ix	5.99053752	4.78547673	1.11178481	0.2916943	1
FBgn003694	Ssk	-4.428246	5.40949173	1.11047786	0.2919781	1
FBgn025922	C3G	-4.3174251	5.03902877	1.10936489	0.29222005	1
FBgn003492	DCP1	2.36074244	6.53907763	1.10764296	0.29259489	1
FBgn003285	Arpc2	6.09999661	5.98994573	1.10713018	0.29270664	1
FBgn003744	Dmtn	-4.3085175	5.61176272	1.10707465	0.29271874	1
FBgn008637	lap	-4.2841437	6.41984587	1.10395879	0.29339896	1
FBgn003242	CG5446	1.64254465	7.40534724	1.10349823	0.29349967	1
FBgn000061	exd	-1.914765	7.13811528	1.10288982	0.29363278	1
FBgn003419	CG6984	3.83097162	4.56039668	1.10275252	0.29366284	1
FBgn004669	Stlk	-5.8627936	7.08464011	1.09846877	0.29460239	1
FBgn003057	CG14414	5.34518228	4.79367466	1.09827523	0.29464493	1
FBgn004160	cpx	-1.3764098	10.3528416	1.09758941	0.29479574	1
FBgn004029	Myt1	-5.9595524	4.59461594	1.09732683	0.2948535	1
FBgn000033	cni	-4.2707147	6.45256587	1.09686548	0.29495503	1
FBgn003248	CG9426	-5.0826292	4.95862577	1.0956998	0.29521176	1
FBgn026470	RhoGEF3	-3.849391	5.27424276	1.09495228	0.29537654	1
FBgn000457	Rop	-1.1480934	9.5058395	1.09488286	0.29539185	1
FBgn028425	LeuRS	-4.2458451	5.62797873	1.09178774	0.29607544	1
FBgn003200	Pvr	2.1606921	7.22931654	1.09002281	0.29646615	1
FBgn026098	RfC4	-3.5447926	5.52479541	1.08951647	0.29657837	1
FBgn005826	MFS17	-4.3707899	7.72678114	1.08937745	0.29660919	1
FBgn003773	CG12948	-6.4340893	4.53521226	1.08852811	0.29679756	1
FBgn002950	CHES-1-like	-3.8647471	4.89275155	1.08831491	0.29684487	1
FBgn003691	Grasp65	-5.1481571	5.43414678	1.08827727	0.29685323	1
FBgn028596	CG46339	2.19249414	6.6984951	1.08672198	0.29719865	1
FBgn002737	MRG15	-5.6366298	5.4298561	1.08664189	0.29721646	1
FBgn003860	Nup43	5.58320818	5.94579314	1.08631722	0.29728864	1
FBgn026145	hpo	4.02984838	3.69674432	1.08356866	0.29790059	1
FBgn002759	GstE12	1.48546802	8.54566186	1.08319248	0.29798447	1
FBgn026552	Smr	-4.4473603	5.81605939	1.08182145	0.29829044	1
FBgn003793	CG14715	1.64317319	7.38750138	1.0777284	0.29920628	1
FBgn003930	RpS27	1.24774002	8.73958153	1.07717419	0.29933057	1
FBgn004028	SMC1	-5.9150771	5.16019164	1.07621779	0.29954521	1
FBgn002022	Cbl	-6.067264	4.7855991	1.07306306	0.3002546	1
FBgn001173	Wee1	-1.8050138	7.10455022	1.07306254	0.30025471	1
FBgn001026	Rbp9	2.67712593	6.91911925	1.07292393	0.30028593	1
FBgn028342	FASN1	-3.5512946	5.66564526	1.07201341	0.30049111	1
FBgn008701	Karybeta3	-3.3262014	5.22752851	1.07052273	0.3008274	1
FBgn028592	Fas1	-1.1467022	9.59024611	1.07036884	0.30086215	1
FBgn026079	p120ctn	-5.3140728	7.30193155	1.06919444	0.30112747	1

FBgn024351(	Vrp1	5.8716546	6.66039809	1.068223	0.30134717	1
FBgn001529:	Srp19	5.67871857	5.41405969	1.06666208	0.30170062	1
FBgn003828(	CG6966	-6.0227552	4.19550075	1.06620844	0.30180344	1
FBgn004034	Lztr1	-6.2620949	5.88073074	1.06586084	0.30188226	1
FBgn002435	Stip1	5.60720376	6.74037788	1.06545687	0.30197389	1
FBgn004582	vsg	1.49759532	9.81071847	1.06533487	0.30200157	1
FBgn026193	dikar	-3.3357866	5.00078128	1.06419116	0.30226122	1
FBgn001043	cora	-1.9297137	7.04141933	1.06310892	0.30250718	1
FBgn002917(	eEF1gamma	1.31962557	8.10742429	1.06016683	0.30317714	1
FBgn002488!	Kap-alpha1	-5.7465515	6.40893443	1.05743278	0.30380144	1
FBgn000461	Plc21C	-6.2495707	5.05355072	1.05678463	0.30394968	1
FBgn003496	MAN1	-4.043065	4.75923429	1.05588427	0.30415577	1
FBgn002354	Nmd3	-4.0237333	5.56578503	1.05551222	0.30424098	1
FBgn002480	DIP1	-1.6619889	7.31604672	1.05479819	0.3044046	1
FBgn003465(	NC2alpha	3.27002577	5.3122341	1.05360893	0.30467738	1
FBgn026085(	Membrin	1.90533383	6.9205193	1.05330713	0.30474665	1
FBgn003166	CG3792	-6.036628	6.51476518	1.0532612	0.3047572	1
FBgn000351	sta	1.19531969	9.85488974	1.04998859	0.30550972	1
FBgn005166	wry	-4.0390323	5.99477481	1.04873572	0.30579844	1
FBgn026658	pit	-5.1460928	3.94242426	1.04869743	0.30580727	1
FBgn003710	CRIF	-4.7738278	7.26730821	1.04867337	0.30581282	1
FBgn026469	mgr	1.52202104	7.70311042	1.04756771	0.30606793	1
FBgn005355	mim	-6.1547267	4.32875954	1.04669575	0.30626931	1
FBgn003692	Gabat	-1.0770145	10.2354378	1.04645008	0.30632608	1
FBgn000459	CycC	-5.6390154	5.01835436	1.04592806	0.30644676	1
FBgn003363	Sod3	-6.0879476	6.59574128	1.04583007	0.30646942	1
FBgn003504	CG13594	2.06564186	7.00419706	1.04496635	0.30666924	1
FBgn003591	Zasp66	2.35295597	6.43392409	1.04472279	0.30672562	1
FBgn003955	BCAS2	-6.3176829	5.49885826	1.04454098	0.30676771	1
FBgn000397	vls	-4.1780763	4.73396199	1.04354103	0.30699935	1
FBgn000001	Abd-B	-2.9832819	5.77449812	1.04232902	0.30728042	1
FBgn002468!	fws	-3.092577	5.36561159	1.04110358	0.30756495	1
FBgn003440	MetRS	-5.8992143	4.40576615	1.04104206	0.30757924	1
FBgn002345	Rbcn-3A	-3.1687435	5.4849154	1.04021932	0.30777047	1
FBgn003047	CG15747	-3.6218475	5.51175139	1.0394769	0.30794316	1
FBgn005252	parvin	-5.0791036	5.21882777	1.0356497	0.3088354	1
FBgn026093	tay	-6.5155559	3.64957746	1.03434843	0.30913953	1
FBgn026272	Pal2	-1.2043821	10.4883489	1.03420751	0.30917249	1
FBgn003710	CG7166	-6.0364648	6.37106447	1.0337711	0.30927459	1
FBgn028350!	Phm	1.12746445	9.17370943	1.03187142	0.30971953	1
FBgn003950!	bigmax	-5.9725642	5.4329045	1.03182311	0.30973086	1
FBgn003878	Sirt2	-3.2924378	5.87112237	1.03065047	0.31000594	1
FBgn028365	muc	-1.4260544	7.85470971	1.03043386	0.31005679	1
FBgn000030	ChAT	-3.687572	4.7942581	1.03024895	0.3101002	1
FBgn008675	cbs	-5.9143521	5.976885	1.02918889	0.31034925	1
FBgn005041(	Rpi	-6.0242422	5.22825104	1.02895298	0.31040471	1

FBgn003937	CG5886	-4.3379095	5.70607912	1.0289373	0.3104084	1
FBgn003669	Papst2	4.60477213	6.04672016	1.02509908	0.31131254	1
FBgn001181	nmo	-6.2083543	5.64520687	1.02286595	0.31184016	1
FBgn008669	Bre1	-4.8325879	4.78415842	1.02209039	0.31202368	1
FBgn002765	jdp	-4.0866703	7.17304989	1.02172779	0.31210952	1
FBgn003726	CG9775	-5.5369839	5.76132725	1.02126536	0.31221905	1
FBgn003746	CG1965	-5.0480356	3.77260859	1.02082869	0.31232252	1
FBgn001122	jar	-5.7052146	5.77482636	1.01999787	0.31251952	1
FBgn003320	CG12107	-5.5667711	5.14897159	1.01937587	0.3126671	1
FBgn005247	Usp10	-4.4472022	3.41658209	1.01927295	0.31269153	1
FBgn000464	svr	-1.2164194	8.64462292	1.01899863	0.31275665	1
FBgn003186	Rat1	-3.4938933	5.26128634	1.01821739	0.31294222	1
FBgn003737	Sym	-4.9992183	5.45861615	1.01618025	0.31342678	1
FBgn026526	Vha68-1	-1.0631784	10.3864732	1.01590351	0.31349268	1
FBgn002868	spt4	6.16781482	5.08678178	1.01554965	0.31357698	1
FBgn003194	CG7149	3.41810635	5.76476399	1.01532785	0.31362983	1
FBgn026075	Mulk	-6.225029	5.89189361	1.01375155	0.31400576	1
FBgn003752	CG2993	-1.3563459	7.87582389	1.01335782	0.31409975	1
FBgn003403	CG8195	-5.6566515	5.03563072	1.01331881	0.31410907	1
FBgn003324	CG11191	-5.7734195	6.40172119	1.01274765	0.31424549	1
FBgn003963	CG14512	5.78493674	4.28927683	1.01213829	0.31439112	1
FBgn003139	CG10874	-6.0771805	4.45023963	1.01161293	0.31451674	1
FBgn002756	Cand1	-4.2266769	3.8043001	1.01129779	0.31459213	1
FBgn003735	RpL13A	1.11961499	10.0159985	1.00962721	0.31499217	1
FBgn003774	CG9471	-5.4244614	6.26737761	1.0085664	0.31524653	1
FBgn003148	CG9641	-4.6528346	5.39514191	1.00807645	0.3153641	1
FBgn003387	CG13339	-5.8109658	5.33490936	1.00750956	0.31550021	1
FBgn002869	Rpn11	1.95071736	7.23704571	1.00665795	0.31570482	1
FBgn000024	by	5.74185502	4.57052706	1.00655763	0.31572894	1
FBgn002176	scu	1.86677355	7.67774737	1.00364193	0.31643087	1
FBgn001579	Rab19	-6.0799577	4.63622207	1.00203437	0.31681875	1
FBgn003761	CG11755	5.75239268	4.27189234	0.99993793	0.31732553	1
FBgn003015	CG9686	0.9907086	11.1427071	0.99968765	0.3173861	1
FBgn001579	Rab18	-4.6109526	6.14961253	0.99904423	0.31754189	1
FBgn000323	ref(2)P	1.22046858	8.69839925	0.99827975	0.31772712	1
FBgn001041	Sdc	-1.2513477	8.42420362	0.99794349	0.31780864	1
FBgn026383	Rcd5	-4.5879076	5.24104889	0.99789479	0.31782044	1
FBgn004065	IM4	4.11483454	4.11669377	0.99652465	0.3181529	1
FBgn025924	brp	-3.4125271	6.48450029	0.99543976	0.31841647	1
FBgn001372	pnut	1.80104961	8.36488831	0.99437111	0.31867638	1
FBgn003676	CG7430	-1.2252508	8.61889394	0.99382787	0.31880861	1
FBgn026613	Dop1R2	-4.1134653	2.7920815	0.99369108	0.31884191	1
FBgn026391	Ent2	1.68800754	7.71677426	0.99358654	0.31886737	1
FBgn005262	AMPdeam	2.03353116	7.57619458	0.99263373	0.31909952	1
FBgn026496	Pcf11	-4.0478139	6.08911118	0.99220527	0.31920398	1
FBgn003999	CG17691	-5.2900779	7.81488357	0.99176222	0.31931205	1

FBgn003803	Sccpdh2	6.34431261	4.61236406	0.99050528	0.31961891	1
FBgn002870	Mst85C	-5.6775922	6.03606403	0.98800679	0.32023002	1
FBgn003261	CG13284	2.74660738	5.78038772	0.98773819	0.32029581	1
FBgn005196	TBCC	-5.9328274	6.56905138	0.98732656	0.32039667	1
FBgn000411	nAChRbeta2	-5.952089	7.86900343	0.98611502	0.32069376	1
FBgn026782	PRAS40	-5.9205438	5.60500557	0.98603699	0.3207129	1
FBgn002733	jim	-1.3740307	8.70325457	0.98320252	0.32140943	1
FBgn005319	CG33199	-6.0472834	6.12782149	0.98305905	0.32144474	1
FBgn003160	Dim1	-6.4019798	4.69018309	0.9828297	0.3215012	1
FBgn002561	Torsin	-5.1601222	4.92746067	0.9824708	0.32158957	1
FBgn005120	CG31200	4.6229619	5.39381866	0.98231391	0.32162821	1
FBgn003517	CG12038	-4.9363358	7.09396755	0.9821445	0.32166994	1
FBgn003479	CG3700	-5.8866618	4.17070582	0.98191473	0.32172655	1
FBgn003803	CG5196	2.79108466	5.77868565	0.98091649	0.32197264	1
FBgn003700	mTerf3	-3.047735	5.23935174	0.98037731	0.32210567	1
FBgn003969	CG7834	1.6794116	7.80942067	0.98032193	0.32211934	1
FBgn002353	CG17778	-5.8138512	5.64193173	0.98026134	0.3221343	1
FBgn003794	CG12594	-2.7168841	5.81857487	0.97906639	0.32242941	1
FBgn002314	Uba1	-2.9403032	6.12452081	0.97826024	0.3226287	1
FBgn003702	HIPP1	-6.3186657	6.06800656	0.97708165	0.32292036	1
FBgn003124	CG11601	2.16178853	6.62280885	0.97694586	0.32295399	1
FBgn005181	DIP-kappa	-5.6689371	4.84478346	0.97636402	0.32309812	1
FBgn003701	CG4858	1.418934	7.69311671	0.97558978	0.32329005	1
FBgn003361	CG7745	-4.4152523	6.6236779	0.97544375	0.32332627	1
FBgn005171	CG31712	-2.4881825	6.77969348	0.97515333	0.32339831	1
FBgn000252	lace	-4.0902684	5.46560229	0.9741761	0.32364088	1
FBgn028353	Vha26	-1.0903494	9.29731106	0.97388972	0.32371201	1
FBgn003061	CG15027	6.33978178	5.09062494	0.97244628	0.32407084	1
FBgn001668	Nurf-38	1.15470085	8.76533185	0.97233139	0.32409943	1
FBgn002999	CG2258	-5.078479	6.29496698	0.97143103	0.32432355	1
FBgn000122	Hsf	-6.565856	4.48626395	0.96962598	0.32477348	1
FBgn003892	CG13408	-6.173506	6.117261	0.96756344	0.3252886	1
FBgn003003	Miga	4.36919147	4.14708509	0.9675459	0.32529299	1
FBgn003005	CG12081	-3.2296932	5.56724158	0.96700246	0.3254289	1
FBgn000058	E(Pc)	2.1199353	6.66150216	0.96606874	0.32566259	1
FBgn026519	Snp	-5.3849247	4.23007215	0.96498811	0.32593332	1
FBgn005247	CG32473	-4.6368324	5.77731731	0.96465133	0.32601775	1
FBgn003648	Prp31	-5.7516603	5.83910242	0.96422975	0.32612349	1
FBgn000003	mAChR-A	-5.0228923	4.5804277	0.96378213	0.32623581	1
FBgn001493	CG2846	2.6010131	6.26758904	0.96318936	0.32638463	1
FBgn002711	miple1	-1.1533927	10.9770434	0.96277058	0.32648982	1
FBgn003189	CG4497	-6.3108481	4.06074185	0.96268357	0.32651168	1
FBgn003159	CG17612	-4.8224312	5.88544898	0.96043316	0.32707777	1
FBgn025997	Cndp2	2.18738491	7.91874197	0.96035108	0.32709844	1
FBgn026297	cnc	3.08663711	5.00302467	0.96011495	0.32715792	1
FBgn003224	CG5355	-3.6347118	5.45024829	0.95981523	0.32723344	1

FBgn003083	CG5445	-5.7260997	6.35205958	0.95959534	0.32728885	1
FBgn003271	CG17321	-6.0169736	5.21358295	0.95883817	0.32747977	1
FBgn004713	CG32276	1.35899905	7.78764741	0.95811674	0.32766182	1
FBgn003166	mRpL28	-6.120578	6.85306241	0.95561447	0.32829429	1
FBgn002495	Irp-1B	-5.5177398	5.60901964	0.95427592	0.32863328	1
FBgn004097	CG16978	3.2762845	5.70471769	0.95199125	0.32921296	1
FBgn002989	CG14442	-5.6728243	5.68175808	0.9518959	0.32923718	1
FBgn000030	chic	1.13961455	9.49753136	0.95070203	0.32954067	1
FBgn026180	cu	-2.6750904	6.57422603	0.94975513	0.32978163	1
FBgn026180	CG42748	-2.7229344	6.0460431	0.9466828	0.3305651	1
FBgn003548	Mul1	-5.3567671	5.26103211	0.94573563	0.33080714	1
FBgn003516	CG13901	-5.725675	5.43732796	0.94452087	0.3311179	1
FBgn003069	mRpS30	-4.0536797	6.25365667	0.94447151	0.33113053	1
FBgn002027	loco	-3.788223	2.81217068	0.94405744	0.33123655	1
FBgn003008	CG6999	-5.8682293	4.8460665	0.94325971	0.33144094	1
FBgn003043	hec	-3.5641938	4.38781733	0.94285524	0.33154463	1
FBgn000273	mago	-5.8197416	6.66291247	0.94275534	0.33157025	1
FBgn028347	S6KL	-4.5291548	5.77476278	0.9421294	0.33173081	1
FBgn001552	RpS21	1.85491269	6.90841438	0.94118197	0.33197406	1
FBgn003571	SP1173	2.76490711	6.04321526	0.94083226	0.3320639	1
FBgn003791	fabp	2.5322147	6.12867884	0.94044912	0.33216237	1
FBgn003854	CG17803	-4.3316829	5.86443726	0.93947326	0.33241334	1
FBgn003087	CG15814	1.85943813	6.99957789	0.93923085	0.33247573	1
FBgn002563	CG13367	-5.2461191	5.19256589	0.93778288	0.33284868	1
FBgn002298	qkr54B	-4.1960224	4.69050925	0.93442624	0.3337154	1
FBgn026397	Caf1-55	-3.6756501	4.06103108	0.93378855	0.3338804	1
FBgn026198	Ire1	-5.8014004	2.85955319	0.9333204	0.33400161	1
FBgn003450	CG11200	3.29185059	5.85341132	0.93293808	0.33410063	1
FBgn004037	G9a	-5.9921017	5.56055568	0.93249498	0.33421545	1
FBgn027543	PheRS-m	-5.9572427	6.36122747	0.93223744	0.3342822	1
FBgn003514	MED30	-5.5334623	3.98960412	0.93108648	0.33458076	1
FBgn002756	CG8108	-2.9402354	5.60634614	0.93009547	0.33483812	1
FBgn002608	tyf	-4.86116	4.31316379	0.92891932	0.3351439	1
FBgn003479	YME1L	1.74910805	7.09668906	0.92778658	0.33543874	1
FBgn003789	Dtd	5.61246227	5.45175565	0.92739897	0.33553972	1
FBgn003701	cmpy	-3.9320714	5.14413252	0.92699096	0.33564605	1
FBgn001691	Stat92E	1.84138732	7.24857137	0.9266798	0.33572717	1
FBgn003490	sona	2.98415445	5.30417179	0.92557912	0.33601433	1
FBgn003553	DopEcR	-5.8151254	6.64025998	0.92543233	0.33605265	1
FBgn002896	deltaCOP	-1.7628426	6.94563807	0.92504131	0.33615476	1
FBgn001205	CalpA	-4.3607501	6.39756325	0.9247781	0.33622352	1
FBgn026641	ringer	-4.8760358	7.25990341	0.92421755	0.33637001	1
FBgn005147	CG31475	-4.746811	5.16099696	0.9227957	0.33674198	1
FBgn028365	CG46301	-4.9738092	4.34561466	0.92114052	0.33717569	1
FBgn008675	chinmo	-1.4934072	7.6852518	0.92097264	0.33721972	1
FBgn003001	GIIIspla2	-5.668991	5.42448599	0.91759418	0.33810746	1

FBgn005201	CG32017	-3.7540122	8.83979083	0.9167748	0.33832323	1
FBgn002673	CG6171	-5.5980217	5.92057966	0.91642806	0.3384146	1
FBgn026645	CTPsyn	-4.3895512	7.00906523	0.91559484	0.33863429	1
FBgn001524	Hsp60A	-1.1833139	10.419504	0.9150277	0.33878393	1
FBgn002659	Fie	-1.121638	8.61251189	0.91468387	0.3388747	1
FBgn003387	CG6357	1.03879093	9.74028457	0.91462139	0.33889119	1
FBgn003756	CR18228	3.17309694	5.03168382	0.91378871	0.33911116	1
FBgn000156	Hlc	-5.990935	4.99124808	0.91346413	0.33919695	1
FBgn003737	CG2046	5.88275372	5.9938295	0.91287562	0.33935258	1
FBgn008539	Dgk	-4.6613426	3.77002305	0.91249638	0.33945292	1
FBgn005254	CG32544	-1.4089601	7.59714638	0.91244055	0.3394677	1
FBgn003251	loqs	-1.9663403	7.17838603	0.9122966	0.3395058	1
FBgn003026	CG1537	-5.5056023	6.33920132	0.91213305	0.33954909	1
FBgn003105	Arp10	-5.3724473	6.52832403	0.91191041	0.33960804	1
FBgn004037	CG13760	-2.2479592	6.41683827	0.91064561	0.33994318	1
FBgn002874	CG5033	4.58264098	5.54151538	0.91018259	0.34006598	1
FBgn001203	AcCoAS	-1.4873257	9.07674882	0.90932485	0.34029362	1
FBgn002432	Mkk4	2.21070517	6.40169479	0.90920759	0.34032476	1
FBgn008700	rtp	5.9543531	3.9142634	0.90860871	0.34048384	1
FBgn001603	lbm	-4.8358126	5.16583667	0.9083687	0.34054762	1
FBgn003423	CG4853	-4.7001416	6.29932088	0.9070801	0.34089034	1
FBgn005038	CG30389	-2.580222	5.88352148	0.90679418	0.34096645	1
FBgn005146	CG31469	4.20136109	5.98874043	0.9067469	0.34097904	1
FBgn002202	eIF3h	-3.4768009	6.72073784	0.90503351	0.34143562	1
FBgn003068	Graf	4.5066302	4.98803132	0.90418635	0.34166167	1
FBgn003354	CG7637	2.46042524	6.04323563	0.90232237	0.34215976	1
FBgn003977	CDase	-5.4331568	5.00250419	0.90163688	0.34234318	1
FBgn002705	STUB1	1.49559627	7.48486901	0.90072044	0.34258861	1
FBgn002857	phtf	-3.6640255	5.61669876	0.90002566	0.34277483	1
FBgn003655	CG5830	-6.3542578	4.33233741	0.89948753	0.34291916	1
FBgn003183	CG9596	-5.5331129	4.48261706	0.89891422	0.34307302	1
FBgn025082	gish	5.61902829	6.66852264	0.89819478	0.34326622	1
FBgn002849	CG30116	-6.1842595	4.99455965	0.89805424	0.34330398	1
FBgn001022	GstS1	1.64860811	7.07283666	0.8976955	0.34340038	1
FBgn002874	Dhit	-4.6978816	5.29765371	0.89657608	0.34370145	1
FBgn003183	CG11050	-5.9819043	6.4956794	0.89611854	0.34382461	1
FBgn003248	mRF1	-5.5829902	4.68382306	0.89582471	0.34390373	1
FBgn003981	CG11317	-5.9493068	6.77721984	0.89578549	0.3439143	1
FBgn003965	Brd8	2.81285081	6.30032967	0.8949888	0.34412896	1
FBgn026733	KdelR	1.65600877	7.11350216	0.89405231	0.34438153	1
FBgn003318	CG1620	-4.6048569	7.52639589	0.89346241	0.34454075	1
FBgn002571	CG13920	-5.616501	6.90574604	0.89316438	0.34462123	1
FBgn002869	Rpn1	1.60463987	7.11753151	0.89263569	0.34476406	1
FBgn002428	Srp54	-2.5887957	6.41335201	0.89172494	0.34501029	1
FBgn003712	Rpb8	2.03104305	6.54769654	0.89138003	0.34510361	1
FBgn000327	RpII215	2.2929828	6.41607465	0.89034962	0.34538258	1

FBgn003823	mRpL11	-6.0872183	4.60876265	0.89019678	0.34542399	1
FBgn001133	Stt3B	1.65347816	7.14097014	0.8901772	0.34542929	1
FBgn026094	lsn	-5.7002007	6.26638741	0.8901235	0.34544384	1
FBgn003300	CG3107	-4.8110147	6.23769478	0.890106	0.34544859	1
FBgn003851	Prx3	-5.2280165	6.92464961	0.88944533	0.34562766	1
FBgn003239	dgt2	-5.1776204	4.55964163	0.88841954	0.34590596	1
FBgn000054	E(bx)	-3.7605213	5.40168681	0.88837467	0.34591814	1
FBgn003066	CG8128	1.5612178	7.38001283	0.88800881	0.34601747	1
FBgn003073	CG9911	1.26959701	7.9662678	0.88679271	0.34634794	1
FBgn003797	Tk	-5.245004	5.97808672	0.88519785	0.34678197	1
FBgn001573	Hmu	-6.1600271	5.81422492	0.88486729	0.34687202	1
FBgn003525	Vta1	-5.6100707	4.82840781	0.88380083	0.34716277	1
FBgn004005	CG17698	-5.9692969	6.78635609	0.88056305	0.34804751	1
FBgn002295	yps	1.19338277	8.09572339	0.88026474	0.34812918	1
FBgn003514	Cypl	-5.6587373	5.13195461	0.87997674	0.34820805	1
FBgn003938	SppL	-5.1837634	5.27342057	0.87983057	0.34824809	1
FBgn003927	Nepl16	-6.2298468	6.24801301	0.87979216	0.34825861	1
FBgn003699	CG5969	-6.0143786	4.96109173	0.87850728	0.34861085	1
FBgn028352	lola	-1.3290573	7.85239434	0.87797147	0.34875788	1
FBgn002980	lin-52	-5.5060509	6.20939005	0.8776773	0.34883864	1
FBgn003917	Spase22-23	2.04427365	6.5178164	0.87678084	0.3490849	1
FBgn000206	AspRS	5.4063976	4.2849324	0.87632977	0.3492089	1
FBgn003153	CG12795	-6.2269735	4.88862032	0.87608553	0.34927607	1
FBgn026199	Ca-Ma2d	-5.6441506	2.68304155	0.87530551	0.3494907	1
FBgn001332	RpL11	1.37728127	7.78897914	0.87481119	0.3496268	1
FBgn003451	CG18067	1.13790671	10.0697719	0.87431261	0.34976416	1
FBgn026287	lute	-3.2611248	5.09248085	0.87429074	0.34977018	1
FBgn001011	hdc	1.89913802	6.85201773	0.87394926	0.3498643	1
FBgn003594	CG5021	-5.1125812	7.23460124	0.87373143	0.34992436	1
FBgn003331	Gle1	-3.6053391	5.5264889	0.87260989	0.35023379	1
FBgn003946	Tsp97E	-5.764231	8.53975514	0.87191998	0.35042433	1
FBgn000004	Act79B	-3.2320771	5.62353933	0.87175676	0.35046942	1
FBgn003538	CG2162	-5.1145241	6.07423231	0.87164991	0.35049895	1
FBgn002498	CG3719	1.91432134	6.65349694	0.86937077	0.35112956	1
FBgn003828	dpr9	-3.4602349	6.36720235	0.86913375	0.35119524	1
FBgn003908	Irk2	-5.8185095	6.12890956	0.86896593	0.35124174	1
FBgn003294	Lamp1	0.99463559	9.82127522	0.86893794	0.3512495	1
FBgn001669	Ptpa	-5.2663321	6.53122354	0.86853172	0.35136211	1
FBgn003224	Bug22	-5.4488981	5.37442076	0.86814602	0.35146908	1
FBgn001038	htl	-3.2559555	4.40624476	0.86771137	0.35158968	1
FBgn003278	Rab9	-4.5770485	4.76088321	0.86760271	0.35161984	1
FBgn003725	CG14650	-5.6010124	5.75629173	0.86700468	0.35178588	1
FBgn003648	Pex3	-6.2159726	5.65409674	0.86611381	0.35203342	1
FBgn003195	CG14535	-4.9116344	3.88876288	0.86611079	0.35203426	1
FBgn003629	nst	-5.7954845	5.48524388	0.86602461	0.35205822	1
FBgn001120	fbl	-5.8565208	4.84711538	0.86572516	0.35214149	1

FBgn026666	Exo70	-5.7161949	4.67985714	0.86565207	0.35216182	1
FBgn026376	lncRNA:CR43	3.30154515	5.36190207	0.86456324	0.35246484	1
FBgn008547	Zdhhc8	-3.066714	4.84917265	0.86390787	0.35264741	1
FBgn003420	Gbp2	3.56467359	3.6663653	0.86370671	0.35270347	1
FBgn003880	TFAM	-5.5319097	4.29598902	0.86301427	0.35289654	1
FBgn002985	CG11700	3.64252856	3.3733406	0.86271545	0.35297991	1
FBgn004030	Jafrac1	1.18457285	8.06320893	0.8623717	0.35307584	1
FBgn003923	CG7006	-5.4859004	5.67229318	0.86022826	0.35367482	1
FBgn005222	CG32225	-1.2136813	7.98209835	0.85995838	0.35375034	1
FBgn003312	Tsp42Ef	-2.7006512	6.17737013	0.85959412	0.3538523	1
FBgn003457	CG9346	-5.4251173	3.46079608	0.85957747	0.35385696	1
FBgn001026	Dsor1	-5.6660702	5.89795996	0.85943798	0.35389602	1
FBgn001759	klg	-5.2581851	6.81228396	0.85826982	0.35422332	1
FBgn003204	Dh31	-4.8451187	7.49216904	0.85672051	0.35465806	1
FBgn002353	CG32809	-4.746141	4.63943138	0.85482442	0.35519111	1
FBgn002894	Cyp28a5	-1.6075768	7.0726587	0.85474702	0.35521289	1
FBgn026467	Eogt	-5.0901354	5.16815572	0.85462116	0.35524831	1
FBgn008538	CG34351	-5.7819589	3.92239672	0.85187741	0.35602177	1
FBgn003096	Atg101	-4.4974686	6.04058946	0.84874849	0.35690662	1
FBgn003808	CG10126	6.60885969	5.71862371	0.84652555	0.35753711	1
FBgn003029	Pa1	-5.810028	6.21297229	0.84533656	0.35787496	1
FBgn003610	galla-2	1.52116836	7.16647961	0.84486889	0.35800797	1
FBgn000026	Cat	2.10418212	8.37331746	0.84464462	0.35807178	1
FBgn003245	CG6180	1.36681548	7.56214142	0.84375352	0.35832547	1
FBgn001395	Fkbp12	1.05992078	8.75652175	0.84323231	0.35847396	1
FBgn004711	CG31142	6.25388886	4.7624401	0.84230805	0.3587375	1
FBgn002312	aay	-3.2638171	4.32417765	0.8422533	0.35875312	1
FBgn003457	cpa	1.80353619	7.00473569	0.8407406	0.35918505	1
FBgn003737	jagn	-5.7195515	5.67734482	0.84067648	0.35920337	1
FBgn000463	drk	1.79089718	6.97282579	0.83953861	0.35952876	1
FBgn002757	CG5815	-6.4411796	4.43188031	0.83812955	0.35993227	1
FBgn002545	CycT	-2.5990676	5.52292576	0.83688186	0.36029009	1
FBgn025922	CG42322	-4.7992603	4.89617952	0.83629888	0.36045744	1
FBgn000386	tsh	-4.3072582	5.41821228	0.83600208	0.36054269	1
FBgn003590	ZC3H3	-5.6487607	5.52613573	0.83545367	0.36070027	1
FBgn003264	Sgt	-4.2727915	6.26183794	0.83459589	0.36094694	1
FBgn003104	Rcd-1	-4.8027591	6.85621085	0.83240261	0.36157871	1
FBgn004047	cid	-4.9594492	3.96845838	0.83180451	0.36175125	1
FBgn003958	beat-VI	-6.058685	4.35857431	0.8293279	0.36246695	1
FBgn003413	RpS15	1.05578907	9.48862449	0.82914484	0.36251992	1
FBgn003105	Ubqn	-2.0036034	7.57701866	0.82914313	0.36252042	1
FBgn003710	CG11307	-3.0806859	5.49183002	0.82890487	0.36258939	1
FBgn003221	Usp14	-5.4994787	6.75114257	0.82784508	0.36289639	1
FBgn002065	Trxr-1	-1.3338447	7.596963	0.82776429	0.36291981	1
FBgn005252	CG32521	-0.9761945	10.1250103	0.82763204	0.36295814	1
FBgn000341	sina	-4.1391268	5.64049738	0.82743283	0.36301591	1

FBgn000056	Eip55E	-5.3982811	6.10472574	0.82662862	0.36324922	1
FBgn002994	CG2059	-5.6276848	6.34493954	0.82655194	0.36327147	1
FBgn000262	RpL32	1.43744896	8.41613952	0.82630799	0.36334229	1
FBgn003319	Vps13	4.4562714	4.06438283	0.8258615	0.36347196	1
FBgn003753	PIG-H	5.96626206	5.00213272	0.82533837	0.36362396	1
FBgn003498	cN-IIIB	-3.775626	6.6107968	0.82444663	0.36388328	1
FBgn003265	Oli	-5.9891039	5.94735681	0.82260705	0.36441903	1
FBgn026427	Sxl	-2.2878149	7.08175859	0.8225769	0.36442782	1
FBgn001667	Ipp	-1.0567681	8.76162084	0.82141649	0.36476634	1
FBgn003929	Alg9	2.84996002	6.10626567	0.81930548	0.36538331	1
FBgn000033	cm	-5.2085989	5.74632834	0.81828335	0.36568255	1
FBgn003288	TM9SF2	1.13126861	8.60508747	0.81756535	0.36589296	1
FBgn003657	CG5151	5.83268763	4.99706343	0.8161998	0.3662936	1
FBgn000203	l(2)37Cc	1.23654607	7.96894645	0.81589118	0.36638423	1
FBgn000465	fru	5.09983377	5.45727119	0.8158719	0.3663899	1
FBgn003126	Sf3b1	5.06642508	4.8862272	0.81560693	0.36646774	1
FBgn026326	sel	-5.4534294	4.02524637	0.81498254	0.36665125	1
FBgn002427	trio	-4.5070942	5.3105305	0.81428862	0.36685535	1
FBgn002491	Actbeta	-5.7016162	5.77742082	0.81427583	0.36685911	1
FBgn004028	Aplip1	-1.0251771	9.32256359	0.81340014	0.3671169	1
FBgn026096	Vmat	-2.5294408	9.38822336	0.81211794	0.36749482	1
FBgn003789	CG17734	1.55276814	7.67027112	0.8120788	0.36750637	1
FBgn003210	CG13108	-2.4166599	6.75247677	0.81169274	0.36762027	1
FBgn026672	gammaSnap	-5.1788845	7.12302904	0.8110183	0.36781936	1
FBgn002916	Hml	6.33445255	7.130867	0.80948909	0.36827135	1
FBgn003973	Nph	1.22785132	7.92811298	0.80886894	0.36845486	1
FBgn001441	Vps26	1.87414064	6.6551189	0.80805188	0.36869684	1
FBgn000436	Ptp99A	-3.3711058	5.70868942	0.80707882	0.36898531	1
FBgn003953	Mtl	-5.8381992	5.12500558	0.8062573	0.3692291	1
FBgn003613	Ufd1-like	1.21823212	7.82562102	0.8058057	0.36936321	1
FBgn003376	CG13148	-3.9281572	5.38499495	0.80504724	0.3695886	1
FBgn008667	jeb	-1.6936659	7.65935057	0.80486636	0.36964238	1
FBgn003766	Ibf2	-5.537955	4.9871021	0.80444741	0.36976699	1
FBgn006422	RpL5	0.99672674	9.16722996	0.80268209	0.37029268	1
FBgn003225	RluA-2	-5.2963265	5.20597965	0.80203514	0.37048559	1
FBgn026400	dysc	-3.3915947	7.30465057	0.80040378	0.37097267	1
FBgn026156	ReepA	-2.2347195	6.43388382	0.79997313	0.3711014	1
FBgn003600	path	1.80225834	7.19205336	0.79980575	0.37115145	1
FBgn003698	CG5262	3.46340296	5.35430681	0.79861775	0.37150696	1
FBgn003464	Rae1	-4.6657299	5.63988139	0.79801979	0.37168608	1
FBgn003015	CG1468	2.46425789	6.21875676	0.79774704	0.37176782	1
FBgn003784	Tsp86D	4.35967349	6.2505904	0.79760772	0.37180959	1
FBgn001174	Arp6	-6.2072835	5.71243855	0.79756664	0.3718219	1
FBgn003158	Naprt	-3.4156343	5.46472048	0.79697762	0.37199855	1
FBgn003385	fand	2.53881935	5.76844551	0.79686044	0.37203371	1
FBgn003963	Ctl2	-5.6147073	6.24924534	0.7963208	0.37219567	1

FBgn003724	CG9855	-5.2622064	2.55625314	0.79602539	0.37228437	1
FBgn001034	Cdc42	1.17133527	7.92486857	0.79592697	0.37231394	1
FBgn003124	CG11562	-5.6008421	3.89979096	0.79588967	0.37232514	1
FBgn003214	CG4036	3.76607021	5.33295614	0.79516556	0.37254273	1
FBgn003985	CG11539	-5.1504529	4.15650629	0.79483527	0.37264204	1
FBgn003765	CG11980	-5.6537107	5.91387586	0.79457698	0.37271973	1
FBgn003319	Corin	4.218413	5.01498193	0.79375438	0.37296729	1
FBgn025924	CG42340	-4.6267351	4.37340982	0.792499	0.37334555	1
FBgn025911	asRNA:CR42	-4.9529524	5.481074	0.79061348	0.37391469	1
FBgn000371	tko	-5.3854008	6.74884195	0.79037081	0.37398803	1
FBgn025982	Ca-beta	-3.7252297	5.61856029	0.79011548	0.37406521	1
FBgn005105	CG31051	-5.7387254	5.95316408	0.78741194	0.37488385	1
FBgn000259	RpLP1	0.98559708	10.1029285	0.78619702	0.37525255	1
FBgn003585	MED24	2.86800608	5.92595067	0.78613738	0.37527066	1
FBgn003746	CG1943	1.47190126	7.30526754	0.78610793	0.3752796	1
FBgn001176	Dp	-5.6346872	5.60197057	0.78527568	0.37553249	1
FBgn004038	CG5273	-1.2947947	8.47521431	0.78366609	0.37602226	1
FBgn003166	CG8892	-5.6907158	5.10380007	0.78242892	0.37639932	1
FBgn003542	nSMase	4.16092821	6.4128581	0.78236965	0.3764174	1
FBgn001147	l(3)neo43	-5.4283144	6.26431096	0.78214471	0.37648601	1
FBgn003760	CG8032	-4.6659996	5.19750244	0.78206558	0.37651016	1
FBgn004035	CG11638	-5.9567667	4.76823443	0.781567	0.37666232	1
FBgn003145	CG3077	-3.5572093	5.1462153	0.7814218	0.37670666	1
FBgn026667	Sec5	-5.9995577	3.92635287	0.78075669	0.37690982	1
FBgn005265	Sec16	2.11045904	6.78615231	0.78066205	0.37693874	1
FBgn003940	CG14543	-4.7286496	5.19097401	0.78013874	0.37709871	1
FBgn000863	betaCOP	-5.498365	5.20009694	0.77894516	0.37746394	1
FBgn003537	CG12093	-5.4933689	5.83560948	0.77673936	0.37814022	1
FBgn002899	onecut	-5.1462143	5.83140533	0.77589723	0.37839886	1
FBgn003789	mRpL40	-3.7432491	6.29524641	0.77501858	0.37866898	1
FBgn025090	Cht10	-4.3862346	4.64187814	0.77483214	0.37872633	1
FBgn004038	CG5254	1.71304904	7.54045294	0.7738518	0.3790281	1
FBgn001560	toc	-4.1390974	2.93818493	0.77196207	0.37961076	1
FBgn003301	Atf6	1.51694689	7.16791461	0.77133782	0.3798035	1
FBgn002554	unc-119	-5.8333961	6.8667741	0.77076668	0.37997998	1
FBgn003662	roq	-5.4233217	4.44695293	0.77065653	0.38001403	1
FBgn026484	vih	-5.6741624	4.6395116	0.77044827	0.38007841	1
FBgn003991	mav	-5.2364378	3.51643494	0.76949563	0.38037313	1
FBgn026504	cv-d	3.04483755	4.77720506	0.76926956	0.38044312	1
FBgn002870	Nckx30C	-2.4212403	7.21761302	0.76883891	0.38057649	1
FBgn008678	stmA	-5.1549651	6.53561462	0.76695448	0.38116086	1
FBgn005310	eIF4EHP	-4.733314	6.36143221	0.76677156	0.38121765	1
FBgn001353	MED20	1.76773782	6.89661467	0.76676609	0.38121935	1
FBgn002898	Spn88Ea	2.02629772	7.51801139	0.76661799	0.38126534	1
FBgn003099	CG7453	-5.4853265	4.59706318	0.76604779	0.38144249	1
FBgn027092	VAChT	-3.8811136	6.19768177	0.76603175	0.38144747	1

FBgn000203	CG10561	-5.9494114	5.71719019	0.76491044	0.38179617	1
FBgn000278	mor	-2.2909701	6.06528005	0.76467306	0.38187004	1
FBgn003232	YL-1	1.90438714	6.56500898	0.76455615	0.38190644	1
FBgn003163	CG12194	-6.0519866	5.44603342	0.76306578	0.3823708	1
FBgn000394	Ubi-p63E	1.98750933	6.6122692	0.76245268	0.38256205	1
FBgn001022	Dbp45A	6.27308015	6.21936794	0.76234519	0.3825956	1
FBgn005267	Tango5	-6.1573093	5.41822411	0.76225008	0.38262528	1
FBgn000004	Act5C	0.87988885	11.0031837	0.76026812	0.38324463	1
FBgn003027	Lint-1	-5.6748325	5.4388329	0.76010852	0.38329456	1
FBgn003003	CG1636	-4.8048643	5.30192706	0.75883877	0.38369217	1
FBgn003995	CG41099	-2.6525947	6.66811006	0.75804886	0.38393982	1
FBgn003697	eRF1	-4.9528722	6.56154096	0.75795467	0.38396936	1
FBgn002707	ValRS	-3.1276631	5.7071362	0.75707701	0.38424482	1
FBgn003571	CG8549	2.51739628	5.28125851	0.75685456	0.38431468	1
FBgn005269	LPCAT	-5.7182799	5.36534062	0.7560544	0.38456612	1
FBgn003295	Cul2	-2.2866734	7.11802608	0.75578978	0.38464932	1
FBgn026666	Exo84	-5.4353758	5.51033732	0.75574568	0.38466319	1
FBgn003369	RpS11	1.01648423	8.74800879	0.75548901	0.38474392	1
FBgn003010	CG12118	-5.5391899	5.09761859	0.75529768	0.38480412	1
FBgn003996	Coa7	-1.4186794	7.20934416	0.75483472	0.38494983	1
FBgn003520	CG9149	1.53114693	7.02855868	0.75449938	0.38505543	1
FBgn003513	CG1231	-5.618634	5.68645056	0.75425293	0.38513306	1
FBgn003528	CG12025	-1.0759407	8.25319117	0.7535911	0.38534164	1
FBgn003163	CG15628	-2.5779821	6.00579202	0.75352391	0.38536283	1
FBgn003200	CG8353	5.07907326	3.55899865	0.75336833	0.38541189	1
FBgn028425	Arpc4	1.34002418	7.43470199	0.7531291	0.38548734	1
FBgn003522	Iml1	2.73795792	5.35225473	0.75297737	0.38553521	1
FBgn003882	CG17270	5.22007425	6.41533535	0.75278351	0.38559638	1
FBgn003379	CG3955	-1.3446016	7.41848449	0.75266913	0.38563248	1
FBgn003000	CG2260	-5.3264053	5.35421049	0.75209909	0.38581246	1
FBgn003582	Srp9	-5.708004	5.43939566	0.7520062	0.3858418	1
FBgn002873	eEF1beta	1.06371627	8.41716801	0.75051233	0.38631407	1
FBgn004269	wrd	-4.2485232	6.07074998	0.75040026	0.38634954	1
FBgn028349	InR	-3.8024075	4.95677381	0.75003254	0.38646593	1
FBgn003590	ergic53	-3.8727808	5.81445331	0.74993049	0.38649824	1
FBgn002316	AMPKalpha	-2.0658941	6.3403693	0.7498038	0.38653836	1
FBgn003160	CG15440	-4.9494773	5.98895526	0.74938146	0.38667214	1
FBgn001074	Pfdn2	1.33846481	7.40703104	0.74931933	0.38669182	1
FBgn003213	GlcAT-S	-4.7907768	4.51180759	0.74887814	0.38683165	1
FBgn000406	Prosalpha4	1.61187634	7.58085706	0.74845626	0.38696543	1
FBgn003494	CG10904	-5.9961659	5.34100911	0.74653706	0.38757484	1
FBgn003844	CG14903	1.95058509	6.46870714	0.74636786	0.38762863	1
FBgn003235	Ppt2	-5.356414	5.25230253	0.74607808	0.38772078	1
FBgn003715	CG11523	-5.0338051	5.68603133	0.74597326	0.38775412	1
FBgn003399	CG11807	1.48492416	7.12673523	0.74596474	0.38775683	1
FBgn005242	CG32425	-4.0415594	4.51008686	0.74574196	0.38782771	1

FBgn000327	RpII18	1.07716083	8.23312686	0.74561694	0.38786749	1
FBgn000304	CG12717	-5.1483323	5.30374597	0.74491018	0.3880925	1
FBgn003708	CG7519	-5.6888951	5.25942699	0.74474857	0.38814397	1
FBgn003324	CG2915	-5.4204857	6.36084554	0.74402391	0.38837492	1
FBgn003630	sowah	-2.2482014	5.94810273	0.74251009	0.38885799	1
FBgn006292	hpRNA:CR33	-4.7130233	3.6925826	0.74125318	0.38925974	1
FBgn005322	CG33229	-5.4451491	5.31221922	0.74106081	0.38932128	1
FBgn002562	CG16989	-5.3172999	5.33085325	0.74087348	0.38938122	1
FBgn003765	CG11983	4.3955019	3.71734317	0.74077114	0.38941397	1
FBgn000128	janA	-4.8532151	5.02653668	0.73968	0.38976341	1
FBgn003552	CG11583	-5.0451373	6.11659507	0.73963879	0.38977661	1
FBgn005330	CG33307	5.56449047	3.19614907	0.73961361	0.38978468	1
FBgn001993	RpS20	1.02497732	8.65359551	0.73802756	0.39029345	1
FBgn003036	CG1492	-5.7253688	5.67884444	0.73781586	0.39036144	1
FBgn002736	Stam	2.9887245	5.6335682	0.73776087	0.3903791	1
FBgn003805	CG18549	-1.9674449	6.61037884	0.73690704	0.39065347	1
FBgn002907	icln	-4.2107325	5.76200995	0.7367731	0.39069653	1
FBgn005111	HDAC11	-3.7927449	4.15847874	0.73646223	0.39079651	1
FBgn026675	lncRNA:CR45	-5.2990667	4.35368675	0.73584605	0.39099479	1
FBgn004162	Hexo2	1.41825814	9.42089242	0.73572672	0.39103321	1
FBgn003980	CG12071	4.82940449	5.95695776	0.73530347	0.39116951	1
FBgn004163	Hexo1	-5.5398741	5.52387989	0.73495544	0.39128164	1
FBgn026634	nAChRalpha4	-4.4324999	5.54231558	0.73336794	0.39179368	1
FBgn003278	RtcB	-5.1306735	6.3293776	0.73317125	0.39185719	1
FBgn026195	kdn	-1.2618072	7.74465947	0.7326267	0.3920331	1
FBgn003165	CG14043	-5.2336748	6.77846157	0.73260753	0.39203929	1
FBgn002759	Kank	-5.6467067	5.38304062	0.73209761	0.39220412	1
FBgn026290	asRNA:CR43	-4.0891404	3.43592169	0.73052557	0.39271289	1
FBgn000304	pcx	5.17099653	3.37688294	0.72963187	0.39300254	1
FBgn001604	Tom40	-5.2545249	7.05422359	0.72882975	0.39326278	1
FBgn001163	La	1.1773974	7.7709513	0.72859298	0.39333964	1
FBgn003858	CG7183	-5.9083348	4.73014812	0.72855545	0.39335183	1
FBgn005816	CG40160	-3.5484551	3.0360053	0.72628596	0.39408971	1
FBgn026179	kcc	-2.66925	6.27109136	0.72479644	0.39457508	1
FBgn003702	CG11396	-5.5337522	5.28283831	0.72474469	0.39459196	1
FBgn003972	CG15522	-4.8661975	5.87730042	0.72414537	0.39478751	1
FBgn003691	Usp32	-3.5832178	4.5393595	0.72406094	0.39481507	1
FBgn003474	CG4294	-5.6854005	3.67741378	0.72405356	0.39481748	1
FBgn026450	CG43901	-2.4430721	5.3667558	0.72380452	0.39489879	1
FBgn003733	CG2519	-4.2269988	5.17484528	0.72216763	0.39543381	1
FBgn026187	scaf6	-3.4788242	5.37811954	0.7212218	0.39574343	1
FBgn003836	Arpc3A	-4.5556056	7.42297517	0.72055277	0.39596265	1
FBgn003853	CG7655	-5.3853433	5.22712469	0.72046595	0.39599111	1
FBgn001402	mRpL47	-5.2521608	7.2694312	0.71988618	0.39618125	1
FBgn003572	CG9953	1.1775888	8.72655453	0.71972139	0.39623531	1
FBgn003685	mRpL21	-5.4756816	6.45332399	0.71951692	0.39630241	1

FBgn026644	Kr-h2	-5.5157242	6.7085806	0.71819808	0.39673561	1
FBgn003079	CG4768	-3.2026094	5.6205548	0.71797119	0.3968102	1
FBgn003964	CG11882	2.74850256	5.00596219	0.71752829	0.39695587	1
FBgn003403	CG8207	-5.7404658	5.48005373	0.71751929	0.39695883	1
FBgn003938	CG5890	-3.9891481	5.21418152	0.71616908	0.3974034	1
FBgn003222	GATAd	-5.5958277	6.83493321	0.71533312	0.39767901	1
FBgn005110	CG31109	-5.3207438	5.11661396	0.71518454	0.39772803	1
FBgn003585	UbcE2M	5.47001043	5.59943199	0.71486425	0.39783371	1
FBgn000122	Hsp67Bc	-5.8787397	4.66747248	0.71468244	0.39789373	1
FBgn003387	CG6329	-1.7180531	8.32749753	0.71450281	0.39795303	1
FBgn000349	spz	-5.6177529	3.66347524	0.71448129	0.39796014	1
FBgn004580	Uhg1	-5.8264669	4.57648364	0.71188433	0.39881897	1
FBgn002972	CG3009	-1.2034301	7.66899761	0.71096191	0.39912467	1
FBgn003091	CG6179	-5.4241265	5.52016596	0.70855065	0.39992538	1
FBgn003757	CG7483	2.34237326	5.99921414	0.70849342	0.39994442	1
FBgn003802	GstD9	2.14413295	6.05861318	0.70831724	0.40000301	1
FBgn000450	Fur1	-3.3254395	3.59468398	0.7075683	0.40025226	1
FBgn003764	CG11964	-5.7397085	5.72396407	0.70705279	0.40042396	1
FBgn001028	Trf	2.24227908	5.90055572	0.70659975	0.40057493	1
FBgn002433	MED6	-4.6969383	5.14352514	0.70536728	0.40098607	1
FBgn004006	wds	-5.8492985	5.52999517	0.70441914	0.40130277	1
FBgn003106	CG12531	1.59199627	6.86768797	0.70419982	0.40137608	1
FBgn003006	Uros1	1.29285854	7.46499708	0.70349825	0.40161072	1
FBgn003298	Tif-IA	-5.8161448	6.81875832	0.7034684	0.40162071	1
FBgn001030	pigeon	-3.047398	5.7594015	0.70341541	0.40163844	1
FBgn026481	koko	2.62234533	5.24476424	0.70324693	0.40169483	1
FBgn002631	Ugt35B1	-6.1455483	6.33976333	0.70317326	0.40171948	1
FBgn026180	CG42750	-3.8021506	5.41388648	0.70267697	0.40188565	1
FBgn005124	CG31248	-1.5593023	6.99573196	0.70260374	0.40191018	1
FBgn002749	AdSS	-5.8268933	5.9208904	0.7024191	0.40197203	1
FBgn003867	CG6005	-5.4404716	4.75228353	0.70227253	0.40202114	1
FBgn003986	CG11563	-5.5486247	5.1675477	0.7005994	0.40258236	1
FBgn003407	CG8405	-6.1208184	4.90187645	0.7004637	0.40262793	1
FBgn003527	mRpL46	-3.8070019	6.67788049	0.70042852	0.40263974	1
FBgn003071	CG8924	-4.7312951	5.13471491	0.69953423	0.40294024	1
FBgn003341	prel	-1.1863574	7.70377396	0.69946652	0.40296301	1
FBgn002351	mRpL16	-1.83113	6.6539109	0.69903061	0.40310962	1
FBgn026256	CG43103	5.81777702	5.15623863	0.69900847	0.40311707	1
FBgn003317	sPLA2	1.78189249	6.82720827	0.69876654	0.40319846	1
FBgn025075	CG42232	-3.1446565	5.3419663	0.69841816	0.40331573	1
FBgn002756	CG3149	-5.7135516	5.48568808	0.69644293	0.40398151	1
FBgn002564	DAAM	-4.4726632	4.6286688	0.69570448	0.40423083	1
FBgn003427	PIG-A	2.79023131	5.60537591	0.69447988	0.40464478	1
FBgn026000	wtrw	-1.9589895	6.26056047	0.69356773	0.40495351	1
FBgn026147	slim	1.90953994	6.39849744	0.68934664	0.4063867	1
FBgn003986	CstF50	-4.0747219	5.26402499	0.68871219	0.40660276	1

FBgn005285(	CG32856	-5.4113637	4.71458545	0.68783773	0.40690083	1
FBgn026459:	PsGEF	-4.1818825	5.4151254	0.6877654	0.40692549	1
FBgn000066:	fl(2)d	-6.0580249	6.22536579	0.68714195	0.40713821	1
FBgn008520:	CG34174	5.19519476	4.2885877	0.68684633	0.40723914	1
FBgn025948:	Mob3	-5.6171563	6.39167424	0.68665754	0.40730361	1
FBgn002786(	CG9776	-3.9437211	6.02928892	0.68562689	0.40765583	1
FBgn003268!	CG10413	4.78701698	4.82039603	0.68561974	0.40765828	1
FBgn026667:	Sec10	5.51274149	5.98471428	0.68545489	0.40771466	1
FBgn003729	CG1116	-2.8035086	6.082281	0.68463676	0.40799463	1
FBgn003977(	PH4alphaEFE	1.16984449	7.89678975	0.68444259	0.40806112	1
FBgn003371:	mIF3	-4.6390689	4.78868933	0.68389456	0.40824887	1
FBgn003689(	CG9368	-4.035299	5.19241157	0.68337684	0.40842634	1
FBgn003711	CG11248	-5.1457003	5.48042097	0.68127351	0.40914854	1
FBgn003035	CG1840	-4.8817735	2.65979876	0.68070931	0.40934258	1
FBgn003510	mri	1.8901237	6.47230693	0.68030239	0.40948262	1
FBgn003735:	elm	0.9759372	9.06456714	0.67895271	0.40994759	1
FBgn001330:	Nca	-0.924093	9.56351488	0.67882384	0.40999203	1
FBgn026250!	nrm	-5.8422692	5.45559204	0.67864149	0.41005491	1
FBgn003091:	CG6123	-3.9131269	5.11729582	0.67846318	0.41011643	1
FBgn003048:	CG1998	-1.2638518	7.51309137	0.67831578	0.41016728	1
FBgn003575:	RpL18	0.98083842	8.52980415	0.6780839	0.41024731	1
FBgn000047!	dnc	-1.7263427	7.20011631	0.67754192	0.41043444	1
FBgn003997(	CG17508	0.96329125	8.57164862	0.67446023	0.41150088	1
FBgn005314	CG33144	2.78482395	4.49859029	0.67437995	0.41152871	1
FBgn002499:	CG2662	-4.8512412	5.11400984	0.67418575	0.41159606	1
FBgn002341(	Ac3	-2.2774166	5.70858189	0.67411856	0.41161936	1
FBgn005365:	Cadps	-1.4088369	7.34507367	0.67410099	0.41162546	1
FBgn003669(	CG14057	1.93863544	6.27473783	0.67342457	0.41186019	1
FBgn003114:	Ntf-2	1.1731072	7.63766858	0.67338323	0.41187454	1
FBgn000123	lncRNA:Hsror	-1.0474632	10.9108168	0.67214723	0.41230398	1
FBgn026211	RanBPM	-5.6698835	4.27512995	0.67192611	0.41238088	1
FBgn001027:	Ssrp	-3.1136481	5.8520052	0.67133907	0.41258514	1
FBgn003784:	CG4511	-5.4364961	5.15323443	0.66997246	0.41306121	1
FBgn003606:	CG6685	-5.8266351	4.31864219	0.66876299	0.41348322	1
FBgn000492	Ggamma1	-1.0032022	10.560758	0.66817608	0.41368823	1
FBgn003892	CG6028	1.47501747	6.96967303	0.66715686	0.41404462	1
FBgn003079:	CG4789	1.14502354	7.77735827	0.66655086	0.41425673	1
FBgn003713	CG7407	-1.1028615	7.98142949	0.66602295	0.41444163	1
FBgn005112:	CG31125	-4.9581173	4.69980789	0.66568279	0.41456085	1
FBgn003840(	CG5903	-0.9304265	9.05212509	0.66563329	0.4145782	1
FBgn000199	Ca-alpha1D	-3.4361105	4.79808287	0.66555303	0.41460633	1
FBgn003223	CG5056	1.44279325	7.00936717	0.66480274	0.4148695	1
FBgn003450	CG8929	-1.7251233	6.57697933	0.66453057	0.41496503	1
FBgn003492(	CG5597	-4.987934	3.62874728	0.66451279	0.41497126	1
FBgn005354:	CG33543	-1.9400283	6.99730835	0.66355684	0.41530705	1
FBgn002857	hfp	-1.3572568	7.33473535	0.66312449	0.41545904	1

FBgn025091(	Octbeta3R	-4.2223033	3.91516553	0.66142826	0.41605617	1
FBgn026097:	alc	0.96520283	9.08330211	0.66122929	0.41612629	1
FBgn005000:	GstT2	4.61410847	5.21770822	0.66118026	0.41614358	1
FBgn001401(	Rab5	-1.063005	8.06388878	0.66110643	0.41616961	1
FBgn005169	CG31694	1.20447565	7.81021376	0.66095573	0.41622274	1
FBgn003950:	CG5934	-4.0897065	6.35207422	0.66072584	0.41630381	1
FBgn003169:	Ncoa6	-3.471251	4.41530716	0.6602136	0.41648454	1
FBgn026065:	l(3)76BDm	-5.4017044	4.47088729	0.65892889	0.41693834	1
FBgn003836:	Acyp2	-5.5854874	5.86854568	0.65791837	0.4172958	1
FBgn002909	asf1	2.17742884	6.48343995	0.65689153	0.4176595	1
FBgn003885	Bdbt	-5.4069071	5.43147512	0.65549762	0.41815397	1
FBgn003523:	CG12099	-2.0920339	6.3878421	0.6548007	0.41840151	1
FBgn001582	TfIIEalpha	5.5601355	5.2977113	0.65473633	0.41842439	1
FBgn003265:	CG6870	2.2512646	8.3055802	0.65406107	0.41866447	1
FBgn001669	Pdp1	-1.7005516	7.80922354	0.65371782	0.41878659	1
FBgn008700	e(y)3	-3.6887735	3.03608344	0.65331254	0.41893084	1
FBgn003352:	CG12895	2.5118077	5.50931408	0.65182591	0.41946063	1
FBgn003547	Sc2	1.24096478	7.53029341	0.65094859	0.41977374	1
FBgn003765:	Kcmf1	-3.3332871	6.18571545	0.65045154	0.41995129	1
FBgn025993(	Uhg3	-5.5225604	4.91072005	0.65023586	0.42002837	1
FBgn003199	PGAP5	-5.6130526	5.29871583	0.65007559	0.42008565	1
FBgn003506:	CG3594	-4.2596548	4.89422415	0.65002526	0.42010365	1
FBgn003669	rogdi	-4.7512023	5.70618847	0.64992065	0.42014105	1
FBgn003221	CG4972	-4.8623039	7.00639675	0.6480494	0.42081093	1
FBgn001375!	CASK	-2.0254638	6.57929417	0.64792213	0.42085655	1
FBgn003707	CG7632	-4.0412717	5.52606142	0.64791264	0.42085996	1
FBgn002653:	Dek	1.45425304	7.34614142	0.64697087	0.42119776	1
FBgn008539	trv	-5.4349671	7.03705062	0.64653206	0.42135529	1
FBgn003553	mRpS6	-5.4935304	4.9339942	0.64630905	0.42143539	1
FBgn003942	CG5447	-5.1634838	4.73279278	0.6452721	0.42180812	1
FBgn001041:	RpS19a	1.0661643	7.9429698	0.64397255	0.42227593	1
FBgn003787:	ZnT86D	-5.7942095	5.69218808	0.64300617	0.42262431	1
FBgn003070	CG15916	-5.3453249	6.42036264	0.64296471	0.42263927	1
FBgn003648!	CG7011	-5.7822139	5.37369606	0.64251948	0.42279993	1
FBgn003992:	MED26	-1.0671376	7.99539872	0.64245433	0.42282344	1
FBgn002626	bonsai	-5.849707	5.16748887	0.64237743	0.4228512	1
FBgn003967	CG1907	-3.7635846	6.81079121	0.64151235	0.42316369	1
FBgn002971(	CG3568	5.59510485	4.74840495	0.64062285	0.42348534	1
FBgn003492:	CG5339	2.47050149	6.0053342	0.64026576	0.42361458	1
FBgn000577:	Con	-4.1815806	5.91577829	0.63942131	0.42392043	1
FBgn005329(	CG33296	1.47088304	7.55804812	0.63894345	0.42409365	1
FBgn003780!	CG12818	-3.6879877	3.94474909	0.63886197	0.42412319	1
FBgn002851:	EndoGI	1.75352417	6.49304537	0.63824053	0.42434865	1
FBgn003634	Syx13	1.31697767	7.17962104	0.63781069	0.42450469	1
FBgn001026:	RpS13	0.93553618	8.59783654	0.63758006	0.42458845	1
FBgn026360:	Tasp1	2.71942231	5.31696108	0.63700817	0.42479626	1

FBgn003944	CG6425	-3.5932018	3.56672807	0.63648871	0.42498515	1
FBgn026288	lncRNA:CR43	5.25011861	3.510369	0.63528284	0.42542413	1
FBgn028599	hpRNA:CR18	-3.4952192	4.25199002	0.634023	0.42588348	1
FBgn000352	stau	-3.6536726	5.5441119	0.63265002	0.42638493	1
FBgn004065	CG15456	-4.5871897	5.69397606	0.63230104	0.42651253	1
FBgn002983	CG4660	-5.7252251	3.99268021	0.63197845	0.42663053	1
FBgn026672	Snap24	-5.7609825	6.55744775	0.63074252	0.42708308	1
FBgn003456	CG9344	-5.3411584	4.1057763	0.62920458	0.42764723	1
FBgn005225	PMP34	-4.9002507	5.40757023	0.62904952	0.42770417	1
FBgn001486	Ost48	1.05230493	8.29908683	0.62895406	0.42773923	1
FBgn002793	Akap200	1.05965452	8.08540157	0.62771452	0.42819489	1
FBgn003343	CG12926	-5.145571	5.01880095	0.62648572	0.42864731	1
FBgn003973	Mgat2	-5.8748451	5.67765339	0.62599707	0.42882743	1
FBgn026791	CanA-14F	-2.3321434	6.00363505	0.625788	0.42890453	1
FBgn000037	crn	-5.4133134	6.17749647	0.62542215	0.42903949	1
FBgn001357	mtd	1.10398824	7.71296588	0.62511654	0.42915228	1
FBgn003006	CG1789	-5.311731	5.93913398	0.62444957	0.42939859	1
FBgn002966	CG10803	-2.7564143	4.49590867	0.62443562	0.42940374	1
FBgn002657	hang	3.75438581	4.81213141	0.62406669	0.42954008	1
FBgn002992	CG3040	-5.2050983	6.40330226	0.62347813	0.42975772	1
FBgn003247	CG5287	1.55746218	6.74672045	0.62346361	0.42976309	1
FBgn003655	CG17026	-5.1368326	5.36578466	0.62327546	0.4298327	1
FBgn005195	Sbat	-5.0657342	4.42317101	0.62241129	0.43015264	1
FBgn004118	Atx2	-3.0101635	5.29412875	0.61955226	0.43121372	1
FBgn026559	lncRNA:CR44	-5.5152216	5.03385247	0.61931218	0.431303	1
FBgn005049	CG30491	-5.5537108	5.9393741	0.61882307	0.43148498	1
FBgn001679	fz2	-2.5271043	5.57219613	0.61876483	0.43150665	1
FBgn005352	CG33523	-3.875416	6.05022706	0.61742698	0.43200505	1
FBgn002871	Lnk	-2.7773426	6.68394799	0.61741706	0.43200875	1
FBgn002866	VhaM9.7-b	1.12886571	7.62352302	0.61565783	0.43266546	1
FBgn001485	His3.3A	1.0011426	8.0525487	0.61555711	0.4327031	1
FBgn000123	Hsp68	1.34713884	7.0817147	0.61489745	0.43294977	1
FBgn003225	CG6144	-4.975794	5.79704719	0.61478037	0.43299357	1
FBgn003391	VGAT	-5.7491482	5.5318951	0.61442631	0.43312608	1
FBgn002976	SK	-3.6142593	6.0414278	0.61221513	0.43395499	1
FBgn003390	O-fut1	-4.9557479	4.95795456	0.61111548	0.43436812	1
FBgn001158	Trp1	-1.3376775	7.08584241	0.61104926	0.43439302	1
FBgn008635	Invadolysin	-2.2601399	5.32569442	0.61069038	0.43452799	1
FBgn002867	Vha100-2	1.47659075	7.46361984	0.6084848	0.43535888	1
FBgn003409	mrj	-4.9044055	6.46392337	0.60832806	0.43541802	1
FBgn003692	CG7646	-1.00379	8.90755806	0.60815482	0.4354834	1
FBgn003677	mRpS26	-3.6066227	6.65281076	0.60814962	0.43548536	1
FBgn003976	mRpS18C	1.58723892	6.85964694	0.60806243	0.43551828	1
FBgn003788	Arfip	-2.9943915	6.29016933	0.60806167	0.43551856	1
FBgn005147	sgll	-5.3873375	5.49629799	0.60757052	0.43570402	1
FBgn003047	Rbp1-like	-4.7093349	6.78522867	0.60731364	0.43580107	1

FBgn003666	kud	2.26439062	5.51497916	0.60711297	0.4358769	1
FBgn003932	CG10562	2.30638935	6.2451013	0.60603071	0.43628624	1
FBgn000129	kay	-2.4197712	5.53110869	0.60565831	0.43642723	1
FBgn003785	Tpc1	-4.6167815	5.34505599	0.60524317	0.43658448	1
FBgn000330	rux	2.14569759	5.60874083	0.60498205	0.43668344	1
FBgn003700	CG5104	-4.8570541	4.75263925	0.60184069	0.43787658	1
FBgn003933	XNP	-5.0321368	5.59871154	0.60140167	0.43804372	1
FBgn026527	Inx3	-5.1726913	4.85567542	0.59975542	0.43867136	1
FBgn026631	lncRNA:CR44	3.89535652	4.72742471	0.59917033	0.43889476	1
FBgn026181	pico	-4.1343297	4.31953975	0.59854748	0.43913277	1
FBgn003617	CG7368	4.30503929	2.62411177	0.5984933	0.43915348	1
FBgn026537	lncRNA:CR44	-4.2119323	4.16762784	0.59813211	0.4392916	1
FBgn003831	mRpL9	-5.4902574	6.18768537	0.59710691	0.439684	1
FBgn003102	ND-18	-0.9576612	8.59626429	0.59561058	0.44025771	1
FBgn003305	Pngl	-5.466336	4.02245003	0.59461863	0.44063866	1
FBgn026563	sno	-5.7246089	3.93069341	0.59420512	0.44079762	1
FBgn001011	hig	-0.9233072	8.8449084	0.59349203	0.44107194	1
FBgn003320	mRpL52	-5.5593644	5.73053007	0.59337728	0.44111611	1
FBgn003390	mRpS16	-5.1033385	5.18312245	0.59314299	0.44120631	1
FBgn003990	Zip102B	-1.959024	6.34860019	0.59277489	0.44134809	1
FBgn028594	para	-1.4467437	7.72189486	0.59251529	0.44144812	1
FBgn000349	sr	4.27805066	5.11288228	0.5917996	0.44172407	1
FBgn026310	psq	-3.4746578	5.8237988	0.59122058	0.44194752	1
FBgn004037	CG13375	-5.3108618	4.34205859	0.59000752	0.44241621	1
FBgn003677	Wdr92	-4.9892313	6.83670144	0.58927497	0.44269962	1
FBgn002834	Ptpmeg2	-1.9661839	6.40648515	0.5890956	0.44276906	1
FBgn003502	uri	-2.7832558	5.237711	0.58866609	0.4429354	1
FBgn008543	CG34401	-3.5236201	5.70327553	0.58863648	0.44294687	1
FBgn002848	CG4341	-4.2401917	4.34375062	0.58838146	0.44304568	1
FBgn003274	Ttc19	5.88806941	5.71103065	0.58652878	0.44376457	1
FBgn003337	CG8078	-4.7647034	4.44989249	0.58647876	0.443784	1
FBgn003514	Gale	-1.9355553	6.40516842	0.58647693	0.44378471	1
FBgn005007	Blos1	-4.7250495	5.43860941	0.58530104	0.44424195	1
FBgn000109	bnb	1.86075663	6.14222352	0.58485885	0.44441408	1
FBgn008396	CG34132	-5.5072898	5.4474559	0.58474119	0.4444599	1
FBgn003582	CG8111	-5.5723923	5.07474082	0.58384398	0.44480954	1
FBgn026159	RpS26	0.88043427	9.2296185	0.58348718	0.4449487	1
FBgn001327	Gp150	5.90120259	4.83567656	0.58275994	0.44523254	1
FBgn003775	CG8507	1.19005649	7.39489618	0.58262142	0.44528664	1
FBgn001334	TfIIA-S	-5.1113915	6.82515973	0.58015694	0.44625081	1
FBgn003934	Npl4	1.61606818	6.67408019	0.57995396	0.44633037	1
FBgn003771	CG9396	6.05859308	4.52878669	0.57953182	0.44649589	1
FBgn003781	Cyp12e1	-5.3132115	5.25883729	0.57897606	0.44671396	1
FBgn003753	CG2698	-2.7501895	6.15285371	0.57774636	0.44719703	1
FBgn003122	CG11454	-5.6697014	4.91528132	0.57723415	0.44739849	1
FBgn003995	CG12567	1.60410921	6.6566638	0.57612081	0.44783687	1

FBgn003857	CG12321	2.42706807	5.52944715	0.57599765	0.44788541	1
FBgn003699	CG5199	-4.6603311	4.94967319	0.57493222	0.44830561	1
FBgn001024	Lcch3	-3.0459783	6.42343492	0.57477842	0.44836633	1
FBgn026161	larp	-2.2671169	6.37269097	0.57439227	0.44851881	1
FBgn002615	ApepP	-4.5238273	6.1430922	0.57430657	0.44855266	1
FBgn026456	garz	1.89092062	6.24747161	0.57419664	0.44859609	1
FBgn005318	CG33181	-1.1849786	7.67396685	0.57398242	0.44868073	1
FBgn002567	CaBP1	1.19199145	7.63430953	0.57293919	0.44909332	1
FBgn026559	Bx	-2.2681702	5.84142877	0.57186486	0.44951882	1
FBgn003128	CG4291	-4.3289041	5.19015322	0.57131465	0.44973698	1
FBgn005014	CG30148	5.68748834	4.27760645	0.56918867	0.4505815	1
FBgn000330	rut	-4.148663	3.63693086	0.56909602	0.45061836	1
FBgn003831	CG6276	-3.7115047	6.6974049	0.56901769	0.45064952	1
FBgn003614	GlcAT-P	-5.0522626	5.2476527	0.56833286	0.45092216	1
FBgn000451	Mdr65	-5.6479284	4.38397199	0.56827511	0.45094516	1
FBgn003251	CG6565	1.7204543	6.35300085	0.56769199	0.45117752	1
FBgn003445	CG11007	-2.1439108	6.22475964	0.56762281	0.4512051	1
FBgn003734	CG14671	-5.2725017	5.40483166	0.56711993	0.45140565	1
FBgn003335	Tom7	1.06039058	7.76598276	0.56668697	0.45157844	1
FBgn003558	Fitm	-3.1584086	5.1586579	0.56656478	0.45162722	1
FBgn003514	Hipk	-5.2920066	6.46360791	0.56566995	0.45198471	1
FBgn001333	Sap47	-0.8694504	9.58238199	0.5654775	0.45206166	1
FBgn004269	NfI	4.90477629	5.90053763	0.56534103	0.45211623	1
FBgn026257	CG43114	2.70682342	3.62401091	0.56529273	0.45213555	1
FBgn002966	CG10802	-2.6875282	5.72523532	0.56402708	0.45264221	1
FBgn003335	PAN2	-5.1163201	3.00180948	0.56396474	0.45266719	1
FBgn000542	ewg	-5.8412022	3.90863902	0.56365507	0.4527913	1
FBgn026736	l(2)SH0834	1.76301385	6.60734129	0.56354029	0.45283731	1
FBgn001130	Rsf1	-0.9817922	7.91344638	0.56339727	0.45289466	1
FBgn028595	eEF5	0.98327295	8.00143812	0.56326133	0.45294918	1
FBgn003342	Updo	-5.6219022	4.41855965	0.56324178	0.45295702	1
FBgn025916	CG42268	-3.680406	5.50531684	0.56310234	0.45301296	1
FBgn002729	Idh3a	-0.8800956	8.56588303	0.56184633	0.45351728	1
FBgn000485	RpII15	-5.0295741	5.42738883	0.56156894	0.45362879	1
FBgn002705	CSN4	1.6970197	6.81607058	0.5615176	0.45364942	1
FBgn003624	ssp	-5.5826879	5.07048601	0.56137942	0.45370499	1
FBgn003131	Charon	-4.0255853	4.84216738	0.56112587	0.45380697	1
FBgn003198	CG12375	-4.9758836	5.92719232	0.56104711	0.45383865	1
FBgn003909	CG10184	2.50623676	5.38750163	0.5608381	0.45392276	1
FBgn003320	DCTN4-p62	-5.372205	5.25309187	0.56007551	0.45422982	1
FBgn003710	MED1	-2.831641	3.98850817	0.55964296	0.45440413	1
FBgn003983	mRpL32	5.7736496	5.95610877	0.55949014	0.45446575	1
FBgn003088	CG7772	-2.4087974	4.83032577	0.5583232	0.45493664	1
FBgn000419	ct	-4.7547193	6.50573198	0.55821403	0.45498073	1
FBgn002642	HDAC6	-3.4360816	4.17876648	0.55757303	0.45523976	1
FBgn002705	CSN3	-4.9998557	5.11117839	0.55682341	0.45554298	1

FBgn000424	Syt1	-1.3028628	7.79226534	0.55672319	0.45558354	1
FBgn003279	CG10166	-4.4139646	5.46041645	0.55645124	0.45569364	1
FBgn026335	AP-2mu	0.97397913	7.91394226	0.5564038	0.45571285	1
FBgn003197	baf	-4.7079108	6.12854474	0.55631823	0.4557475	1
FBgn000463	rho	-2.5424238	6.12054376	0.55590197	0.45591613	1
FBgn003210	CG9586	-5.0903325	4.20994801	0.55563374	0.45602484	1
FBgn002570	CG5885	1.31760713	7.02282812	0.55557907	0.45604701	1
FBgn003457	CG10543	-2.0265282	6.46618044	0.55490652	0.45631979	1
FBgn001368	mt:ND6	1.03159689	7.74073302	0.5545504	0.45646434	1
FBgn008644	l(2)37Cg	2.4189124	4.79206227	0.55443905	0.45650955	1
FBgn000015	awd	0.81408893	9.52260011	0.55394487	0.45671029	1
FBgn003674	Crtc	5.15711421	4.84516726	0.55344262	0.45691444	1
FBgn003074	CG9919	-0.9770305	7.91219795	0.55327821	0.4569813	1
FBgn005330	CG33303	-3.7865726	7.25465691	0.55313577	0.45703924	1
FBgn003213	CG4364	-4.4280734	5.72061871	0.55239623	0.45734025	1
FBgn025983	CG34229	-4.802878	4.18503963	0.55180315	0.45758186	1
FBgn001537	RabX1	-4.9793656	5.03843701	0.55162653	0.45765386	1
FBgn026672	Trs33	4.39697617	4.71547737	0.55161842	0.45765716	1
FBgn008670	stv	-2.5803801	4.33025268	0.55098386	0.45791597	1
FBgn003421	Mtap	1.26356753	7.11838236	0.55000661	0.45831499	1
FBgn002970	CG3556	-4.7107072	6.04264672	0.54915438	0.45866342	1
FBgn006119	HSPC300	2.03899514	5.63457826	0.54912593	0.45867506	1
FBgn000194	ifc	-4.6707599	4.34098375	0.54884944	0.45878819	1
FBgn000011	arm	-0.9608921	8.42707708	0.54805045	0.45911537	1
FBgn025970	Nsun5	-5.2020311	4.49038806	0.54799671	0.45913739	1
FBgn000113	gro	-5.9634267	5.76246357	0.54727241	0.45943433	1
FBgn003229	EMC3	1.65303138	6.50534467	0.54708041	0.4595131	1
FBgn003930	CG11857	-2.0970651	7.03425457	0.54684786	0.45960852	1
FBgn001575	Lis-1	-0.9433336	8.54066602	0.54653461	0.45973712	1
FBgn003590	GstO2	1.83881004	6.26885672	0.54635803	0.45980963	1
FBgn000321	rdgB	-3.5898615	5.63603767	0.54483006	0.46043786	1
FBgn003305	CG14591	-3.7823277	6.00604496	0.54445092	0.46059395	1
FBgn028591	red	-2.5853056	5.79867253	0.5443381	0.46064042	1
FBgn003135	CG17660	-2.0794009	5.72525163	0.54416919	0.46071	1
FBgn002950	CHORD	-5.2331332	4.69009272	0.54399983	0.46077978	1
FBgn027860	Dsp1	-1.8658948	6.84834639	0.54381077	0.4608577	1
FBgn003106	CG14232	1.51126004	7.82390231	0.54287786	0.46124249	1
FBgn000338	Sh	-1.3418688	7.7390504	0.54282709	0.46126345	1
FBgn002997	spidey	-0.8521396	8.73205407	0.54138442	0.46185955	1
FBgn005042	Usp15-31	-5.6823731	3.92036072	0.54099853	0.4620192	1
FBgn008678	Vps36	2.44221976	5.41899187	0.54074801	0.46212289	1
FBgn003788	CG17721	-5.1212241	3.93065679	0.5401149	0.46238511	1
FBgn005847	CG40470	-2.7017268	5.65349977	0.53968793	0.46256209	1
FBgn026543	zip	1.24181913	7.14972988	0.53943981	0.46266498	1
FBgn004213	Capr	-2.2461641	5.1548382	0.53795342	0.46328213	1
FBgn003269	CG10333	-2.8307634	6.05456217	0.53703607	0.46366368	1

FBgn003790	CG5281	-5.6008898	5.91734381	0.53673794	0.46378778	1
FBgn001051	wal	1.11771707	7.69685618	0.53632618	0.46395928	1
FBgn003804	CG5641	-5.4721507	5.46073918	0.53497517	0.46452267	1
FBgn003111	RhoGAP19D	-1.7684405	6.14083316	0.53482032	0.46458732	1
FBgn003990	Syt7	0.86604866	9.49682319	0.53481854	0.46458806	1
FBgn003342	CG1814	1.1834962	7.39964985	0.53475642	0.464614	1
FBgn003974	CG7943	2.40138383	5.53447975	0.53435712	0.46478078	1
FBgn005308	CG33080	-2.6537059	4.81187884	0.5342936	0.46480731	1
FBgn005280	CG32803	-4.4476233	3.57868961	0.53419757	0.46484744	1
FBgn004345	Ndf	-1.8550351	6.97440753	0.53401295	0.4649246	1
FBgn004082	dpr6	-3.9808637	5.59509068	0.53392759	0.46496028	1
FBgn003805	CG5608	-2.8600045	6.14584062	0.53338499	0.4651872	1
FBgn003181	CG9536	2.83237111	5.64714115	0.53290245	0.46538916	1
FBgn000347	spn-A	-5.5353748	4.91843822	0.53275739	0.4654499	1
FBgn026159	RpS24	0.80509228	9.53633651	0.53251178	0.46555277	1
FBgn003798	ATP8B	3.33387536	5.31447519	0.531914	0.46580329	1
FBgn004031	Gclc	-3.9623301	4.28562432	0.53121517	0.46609643	1
FBgn003057	sbm	-2.4507941	6.26300922	0.53083536	0.46625587	1
FBgn003858	CG7168	-4.169553	3.78734346	0.52918484	0.46694977	1
FBgn003779	CG6254	-3.7445415	6.09666427	0.5290947	0.46698772	1
FBgn026628	Ns3	4.6392945	6.58352886	0.52875004	0.46713284	1
FBgn003563	Txl	-4.6019628	5.72858606	0.52826876	0.46733562	1
FBgn008539	Fili	3.02939888	2.77744424	0.52790843	0.46748753	1
FBgn003281	CG10366	-5.0844558	5.97059583	0.52744934	0.46768119	1
FBgn003975	RpS7	0.77791342	10.0637837	0.52671617	0.46799073	1
FBgn026086	Vti1a	-3.4608243	4.92081029	0.52671419	0.46799157	1
FBgn025982	CG42404	-5.5497562	4.30508835	0.52646906	0.46809513	1
FBgn003149	SerRS	1.03283369	8.22404675	0.52590877	0.46833199	1
FBgn000538	ash1	-3.5603896	5.20909787	0.52558029	0.46847095	1
FBgn003552	Fdx2	-5.1457625	5.19306395	0.52551481	0.46849866	1
FBgn003221	CG5390	-4.1291728	4.62718931	0.52483231	0.4687876	1
FBgn003895	CG5382	-1.4751846	6.81465899	0.52415193	0.46907594	1
FBgn005171	CG31717	1.00869636	7.70677631	0.52350544	0.46935018	1
FBgn003139	CG11723	-5.3510096	5.0220777	0.52341629	0.46938802	1
FBgn000114	Gs1	-1.0380912	7.67562427	0.52315697	0.46949811	1
FBgn003142	Atxn7	-2.3151931	4.86324246	0.52283679	0.46963409	1
FBgn026444	ab	-2.3477886	5.71666235	0.5214207	0.47023628	1
FBgn003533	CG15877	1.62263102	6.40373751	0.52114572	0.47035336	1
FBgn003448	CG11099	-5.3995861	5.72515252	0.52091088	0.47045339	1
FBgn003754	CG11035	-5.2854876	4.44388405	0.52062876	0.47057359	1
FBgn000351	sqh	0.83705961	8.69327173	0.52052011	0.4706199	1
FBgn002472	Slip1	-1.8931904	6.79557797	0.51975323	0.47094697	1
FBgn003522	CG12091	-0.9208878	8.63764768	0.518811	0.47134932	1
FBgn003772	CG8312	-3.3649462	5.38204757	0.51874284	0.47137845	1
FBgn001983	colt	-5.1293099	5.11075293	0.51732586	0.47198463	1
FBgn003148	CG3165	-4.5947925	5.19147143	0.51718219	0.47204616	1

FBgn028591(	VhaAC39-1	-0.8248114	9.54811918	0.51688099	0.4721752	1
FBgn028596:	CG46338	-5.3132982	6.04821276	0.51551954	0.4727592	1
FBgn003470:	CG11170	-5.0335002	4.54530898	0.51537182	0.47282264	1
FBgn003290	CG9272	-5.4672007	5.48563765	0.51485426	0.473045	1
FBgn003692:	CG14182	-5.01781	6.67607183	0.5139086	0.47345173	1
FBgn003226	Lip4	2.32759967	5.62181144	0.51353864	0.47361101	1
FBgn004630	CG7148	-5.6099776	5.3737064	0.51321156	0.47375189	1
FBgn025920	Mlp60A	2.30520836	5.37252804	0.51298206	0.47385079	1
FBgn003857(	Arp5	-4.0474181	5.86741611	0.51289198	0.47388961	1
FBgn003609	CG11811	-2.2846656	4.88520431	0.51253844	0.47404204	1
FBgn028591	sbb	-4.4651148	6.52452588	0.51252751	0.47404676	1
FBgn003922(	Ude	2.51248262	5.03674616	0.512407	0.47409874	1
FBgn003162	fipi	-4.6066916	5.75289364	0.51114099	0.47464535	1
FBgn002985	wuho	-4.7574235	6.00201529	0.51047053	0.47493524	1
FBgn000112	Got2	1.28824539	7.93161904	0.50971486	0.47526232	1
FBgn003262	beat-IIIc	-2.0832646	6.67659633	0.50969529	0.47527079	1
FBgn003135	CG14352	-5.1142057	4.35546686	0.50959464	0.47531439	1
FBgn003095	CG6961	-5.2030043	3.78263409	0.50951998	0.47534673	1
FBgn003215(	CG13124	1.33291204	7.11362053	0.50920634	0.47548263	1
FBgn003147	CG8814	-1.1948026	7.12357325	0.50863294	0.47573124	1
FBgn003121	CG11377	-5.5339844	5.25527774	0.50860726	0.47574238	1
FBgn003552	CG15012	0.85478396	8.70147696	0.50798489	0.47601249	1
FBgn002710	Dyrk3	3.99562617	6.25852706	0.50788374	0.47605641	1
FBgn001075(	atms	1.95390432	5.88347183	0.50786537	0.47606439	1
FBgn005351	Dop2R	1.2832616	7.15288594	0.50677813	0.47653692	1
FBgn003078	CG9609	5.70408654	4.8583299	0.50672455	0.47656022	1
FBgn003436	CG5323	-5.3628877	4.08703321	0.5051221	0.47725812	1
FBgn026085	Bet3	-4.7548993	3.08384956	0.50470013	0.47744218	1
FBgn003131(	Vps29	1.3085136	6.87897347	0.50468858	0.47744722	1
FBgn026659	Hsc70-4	0.74621996	10.5969623	0.50387059	0.47780434	1
FBgn002913	Prosbeta5	0.80004137	9.02814072	0.50325282	0.47807434	1
FBgn005114	CG31145	1.42892363	7.21680163	0.50272389	0.47830571	1
FBgn005039(	Sgf29	-3.565086	5.25073486	0.50222328	0.47852486	1
FBgn002431	Picot	-5.7632617	6.45065464	0.50186206	0.47868309	1
FBgn002184	Ttc7	1.72719282	6.88953445	0.50158287	0.47880545	1
FBgn001607(	smg	-4.8769082	3.30341455	0.50145092	0.4788633	1
FBgn003985	CycG	-0.7779345	9.31485487	0.49926537	0.47982309	1
FBgn002794	msps	-4.9675604	6.08009008	0.49913084	0.47988227	1
FBgn003963	CG11873	-5.2827948	3.79831563	0.49844803	0.48018284	1
FBgn003732	RpL35A	0.93190367	8.59825459	0.49837517	0.48021493	1
FBgn003334	CG8248	-2.2736786	6.46011234	0.49804802	0.48035906	1
FBgn002733	l(1)G0004	-5.4359599	5.99628258	0.49726347	0.48070501	1
FBgn026527(	l(3)neo38	3.76471191	5.34932519	0.49698945	0.48082593	1
FBgn003449	CG11788	-5.1276296	4.74093315	0.49663607	0.48098195	1
FBgn003171	Coq6	4.97396951	6.07090138	0.49648996	0.48104648	1
FBgn003030	CG1572	2.84255807	4.70248882	0.49641137	0.48108119	1

FBgn002707	Ugt49B1	-1.9351355	6.29401546	0.49634641	0.48110989	1
FBgn001562	Cpr	-2.9301533	4.45232646	0.49578747	0.48135694	1
FBgn001576	p38a	-3.157252	4.79531759	0.49497189	0.4817178	1
FBgn003611	Aps	-1.1635866	7.63335932	0.49491801	0.48174166	1
FBgn028594	RpL27A	0.79065558	9.70358177	0.49432344	0.48200503	1
FBgn003165	CG14044	2.30225255	4.39562404	0.49428834	0.48202058	1
FBgn026155	ps	-0.8287902	9.0343623	0.49426338	0.48203165	1
FBgn003542	RpL28	0.8767605	8.35024942	0.49402486	0.48213738	1
FBgn002637	RpII33	-4.6917972	4.22786437	0.49400567	0.48214589	1
FBgn002952	CR18166	-4.9117552	4.65247974	0.49347403	0.4823817	1
FBgn002488	kin17	-4.8405777	4.7871403	0.49329236	0.48246232	1
FBgn003510	p130CAS	-5.2867136	5.08415598	0.49325114	0.48248062	1
FBgn003069	PGRP-LE	-2.7015571	5.09749161	0.49303694	0.48257571	1
FBgn002641	Lim1	-3.9295221	5.17043197	0.49242655	0.48284687	1
FBgn005241	QC	1.68458055	6.15572017	0.49226557	0.48291842	1
FBgn026495	Piezo	1.74469085	6.41517024	0.49184014	0.48310761	1
FBgn003424	Ns2	-5.0513788	6.36715661	0.49112945	0.48342391	1
FBgn003303	kune	-3.2451044	5.22045202	0.4908421	0.4835519	1
FBgn003123	mRpL10	-5.2466286	5.1194647	0.49013308	0.48386795	1
FBgn000309	Pkc98E	-1.8827667	5.90260898	0.49002992	0.48391396	1
FBgn003145	CG2862	0.85927357	8.2976697	0.48984916	0.4839946	1
FBgn001402	Sep-02	1.06502794	8.01789279	0.4891668	0.48429921	1
FBgn001021	Sod2	-0.8495482	8.37696578	0.48886612	0.48443354	1
FBgn000399	hid	-2.7492164	3.65680782	0.48886003	0.48443626	1
FBgn001080	TrpRS	-5.0370037	5.47042397	0.48802906	0.48480782	1
FBgn003674	CG7484	1.51266192	6.50687754	0.48744486	0.48506932	1
FBgn001174	Arp2	1.11518968	7.30725841	0.48706121	0.48524117	1
FBgn002180	Reph	-1.7930899	6.14908408	0.48698561	0.48527504	1
FBgn004039	CG3939	-2.011113	5.2303644	0.48644224	0.48551865	1
FBgn003799	dpr15	-4.2981617	5.45727995	0.48589568	0.48576388	1
FBgn026001	pds5	-3.4084842	2.73313785	0.48570539	0.48584931	1
FBgn002778	Mtch	1.04930397	7.46952292	0.48496918	0.48618007	1
FBgn003002	sni	1.49811278	6.56950925	0.4846717	0.48631381	1
FBgn002185	l(2)k14505	1.06261412	7.48531878	0.4842782	0.48649083	1
FBgn003043	CG4407	-3.6216692	6.11242115	0.48426138	0.4864984	1
FBgn005166	smog	-5.8201115	4.43156141	0.48418571	0.48653245	1
FBgn003572	CG9948	-4.8704071	4.57610424	0.48333804	0.4869142	1
FBgn004120	key	0.87540982	8.15859623	0.48255298	0.48726819	1
FBgn001041	Taf6	-5.1269557	3.27478819	0.48246732	0.48730684	1
FBgn003216	CG4709	5.37855121	4.24296398	0.48244567	0.48731661	1
FBgn003423	CG4866	1.67996351	6.19829666	0.48205421	0.48749331	1
FBgn028350	Sac1	1.07258943	7.42082	0.48197436	0.48752937	1
FBgn002298	qkr58E-3	-5.5317293	5.97473566	0.48155681	0.48771799	1
FBgn002971	CG3527	1.21074907	7.10530466	0.48095619	0.48798952	1
FBgn026286	Gfrl	-4.0819243	5.2427387	0.48027829	0.48829629	1
FBgn005313	AGBE	-2.8226519	5.2373749	0.48014933	0.48835468	1

FBgn003125	CG4133	1.6459104	6.20430703	0.47996321	0.48843898	1
FBgn028424	l(2)k09848	-5.1684862	5.5635096	0.47912443	0.48881918	1
FBgn026475	Vti1b	-5.0579263	5.14456766	0.4790582	0.48884922	1
FBgn001164	Zyx	1.40336351	7.52086439	0.47885888	0.48893965	1
FBgn005351	dpr3	-2.9773271	3.84395593	0.47804103	0.48931099	1
FBgn003730	CG12173	1.25772976	6.91136548	0.47705527	0.4897592	1
FBgn003316	Eaf	-1.8916812	5.71438402	0.47641827	0.49004919	1
FBgn003275	CG10495	-4.5429672	4.72933839	0.47583346	0.49031567	1
FBgn003219	eEF1delta	1.06713943	7.51598316	0.4755152	0.4904608	1
FBgn003747	CG10098	-5.0416176	7.06323828	0.47540818	0.49050962	1
FBgn000061	e(y)1	-4.6411931	4.34459743	0.47533176	0.49054448	1
FBgn026400	GluRIB	3.4711733	5.25502328	0.4743502	0.49099265	1
FBgn008398	CG34150	-5.3094706	5.49315673	0.47405489	0.49112762	1
FBgn003504	Pof	-5.1692809	5.76627848	0.47381551	0.49123707	1
FBgn003722	CG14641	-5.4626591	5.86211795	0.47274581	0.49172667	1
FBgn002494	RSG7	1.31616357	6.79297156	0.47268013	0.49175676	1
FBgn001438	RhoL	4.11345562	5.33051753	0.47252584	0.49182745	1
FBgn003055	CG13404	-5.7323177	5.97830171	0.47185696	0.49213412	1
FBgn004170	CG15107	-5.2437219	3.9230598	0.47169249	0.49220957	1
FBgn002915	Mkrn1	-1.1621857	7.33259113	0.47164678	0.49223055	1
FBgn003814	CG9813	-0.8293966	8.38048929	0.47092514	0.49256187	1
FBgn003445	TBCB	-5.7277372	5.79550421	0.47056235	0.49272858	1
FBgn002779	Lrpprc2	-4.154203	4.25012686	0.46909916	0.49340189	1
FBgn003847	Ns1	-4.4756985	5.74281906	0.46843179	0.4937095	1
FBgn003676	MED19	-2.9080015	5.50751155	0.46742815	0.49417272	1
FBgn003797	KLHL18	-5.196301	6.89168038	0.46602563	0.49482127	1
FBgn003395	CG10139	-4.9128369	6.64330882	0.4658862	0.49488582	1
FBgn001993	SamDC	-3.4175541	6.77807569	0.4657993	0.49492606	1
FBgn003921	Rpb10	1.26650861	6.86395076	0.46552784	0.4950518	1
FBgn001995	Orct	-3.9404004	6.46531869	0.46534624	0.49513595	1
FBgn003179	AANATL2	2.11007229	5.40079976	0.46516435	0.49522024	1
FBgn025917	5PtaseI	5.42173353	5.81061907	0.46415371	0.4956891	1
FBgn003971	dgt1	3.25038149	4.81396323	0.46281709	0.49631034	1
FBgn026635	CG45002	-3.5820815	5.3009892	0.46254727	0.4964359	1
FBgn002980	CG15771	6.04388468	5.47272856	0.46225353	0.49657266	1
FBgn003290	CG14401	5.53587336	4.57498513	0.46221819	0.49658912	1
FBgn001147	PR-Set7	-4.1417767	3.11473746	0.46213989	0.49662559	1
FBgn003669	beg	-5.1519596	5.16126968	0.46182484	0.49677237	1
FBgn003902	CG7029	-2.2736578	6.01637045	0.46150107	0.49692328	1
FBgn000436	HmgD	1.03440206	7.43564566	0.46112776	0.49709738	1
FBgn026684	wap	-3.0233053	5.52221084	0.46094678	0.49718183	1
FBgn003696	FRG1	-2.7793121	4.98674524	0.46007879	0.49758715	1
FBgn003124	CG3625	-0.9703932	8.14720788	0.45967963	0.49777374	1
FBgn003338	GstE13	-5.2315652	5.962787	0.45942174	0.49789435	1
FBgn003326	Obp44a	1.32078628	6.74770827	0.45936543	0.49792069	1
FBgn001007	RpL23	0.79286222	8.76226747	0.4592312	0.49798349	1

FBgn003926	CG11790	0.96990419	7.63023629	0.45915291	0.49802013	1
FBgn003874	Surf6	-4.7560502	4.51246901	0.45901416	0.49808507	1
FBgn026626	FeCH	1.61248834	6.17513521	0.45863955	0.49826047	1
FBgn003549	Chd64	1.03478226	8.09757832	0.45710595	0.49897963	1
FBgn003775	CG8500	-5.5751529	6.07959715	0.45708556	0.4989892	1
FBgn003436	Atg7	-3.1571939	4.88856109	0.45566309	0.49965784	1
FBgn005257	CG32576	1.78727031	5.83421418	0.45562423	0.49967613	1
FBgn001174	Arp1	1.21811225	6.92772407	0.45549592	0.49973652	1
FBgn026075	CG42553	-5.1079173	5.5110928	0.45546171	0.49975262	1
FBgn026197	CG42806	-4.0111414	2.9387068	0.45538668	0.49978794	1
FBgn004217	CG18858	-5.0272984	4.12361172	0.4553762	0.49979288	1
FBgn003032	FucT6	-1.2083307	7.03245819	0.45524271	0.49985574	1
FBgn026128	SREBP	-3.9163875	4.49833282	0.45494027	0.49999819	1
FBgn003631	CG10960	-1.0629532	7.62117332	0.4540697	0.50040863	1
FBgn005206	Rbfox1	-1.6525732	6.56369612	0.45316518	0.50083568	1
FBgn002786	Ogg1	-4.5766539	5.09273089	0.45212051	0.50132968	1
FBgn000056	Eip75B	-3.372599	6.84267788	0.45174865	0.50150572	1
FBgn005352	PNUTS	-1.245696	6.90194911	0.44952612	0.50256008	1
FBgn025083	CG2016	-4.1381134	6.76032556	0.44905925	0.50278204	1
FBgn002661	Taz	-1.5892963	6.50747916	0.44859009	0.50300526	1
FBgn005034	Prp38	4.85724838	4.31644336	0.44817066	0.50320496	1
FBgn003128	CG3662	-0.6535499	11.3501816	0.44810017	0.50323854	1
FBgn003895	CG5380	1.63906021	6.09033317	0.4471777	0.50367827	1
FBgn005028	CG30285	1.83772636	5.6174723	0.44594418	0.50426732	1
FBgn003532	CG1275	0.77796512	9.08127966	0.44573436	0.50436763	1
FBgn003954	CG12877	-4.6701694	4.83676328	0.44514295	0.50465056	1
FBgn026312	Acsl	-1.4945972	7.39946719	0.44460416	0.50490855	1
FBgn003006	Rbm13	-4.6194166	4.54628943	0.44446329	0.50497605	1
FBgn003289	CG9336	0.92165462	7.78038692	0.44406446	0.50516721	1
FBgn003132	CG5126	2.15549645	5.55800245	0.44369782	0.50534305	1
FBgn000262	RpS3	0.79089729	8.60440662	0.44342079	0.50547599	1
FBgn003406	Rrp42	5.86839865	6.13275016	0.44304384	0.50565697	1
FBgn003181	HemK1	-5.5498903	5.91763959	0.44299892	0.50567854	1
FBgn028678	cass	1.13066148	7.32535402	0.44247303	0.50593124	1
FBgn003795	Kyat	1.42906943	6.70670049	0.44241564	0.50595883	1
FBgn002889	GMF	-3.1895178	4.73307262	0.4423432	0.50599365	1
FBgn003490	CG5532	1.46481198	6.44675178	0.44220848	0.50605844	1
FBgn003219	ova	5.38333669	6.05799674	0.44205489	0.50613231	1
FBgn003768	CG8132	-4.9948025	5.87401261	0.44196058	0.50617768	1
FBgn003023	CG17333	1.70968779	5.98077062	0.44194288	0.5061862	1
FBgn000491	Gnf1	1.54555472	6.83068571	0.44147253	0.50641259	1
FBgn000424	Rdl	-1.0347413	8.49567477	0.44138733	0.50645361	1
FBgn003753	CG10435	-4.3910444	5.91891672	0.44061486	0.50682584	1
FBgn003357	ND-B14	0.73992315	9.20396945	0.44044615	0.5069072	1
FBgn003313	Tsp42Ej	-0.7559416	9.51683067	0.44003001	0.50710797	1
FBgn003024	CG2186	3.7302452	3.35930092	0.43999124	0.50712668	1

FBgn003330!	Lnpk	-2.9661414	6.43693475	0.43984981	0.50719495	1
FBgn005350:	U3-55K	2.11524948	5.67548709	0.43941068	0.50740703	1
FBgn003478!	PIP5K59B	-1.8129424	6.94360453	0.43873531	0.5077335	1
FBgn003075:	CDC50	1.51674402	6.33258657	0.43863883	0.50778017	1
FBgn026086(	Bet5	1.3922297	6.6286176	0.43861527	0.50779157	1
FBgn003027(	Dlic	0.86850498	7.89451943	0.43828904	0.50794942	1
FBgn004586(	bai	-4.1526716	6.79925362	0.4374504	0.50835561	1
FBgn003371(	CG17739	1.46183069	6.76964722	0.4373873	0.5083862	1
FBgn003220:	REPTOR-BP	0.76432308	8.72991857	0.43705781	0.50854596	1
FBgn003877	CG4390	1.23400164	7.27145807	0.43682013	0.50866125	1
FBgn000057:	emc	-0.7783551	8.59711241	0.43670749	0.50871591	1
FBgn026211:	CG17683	-1.4812092	6.99725262	0.43633787	0.50889533	1
FBgn026155·	CG42672	5.04531832	3.39179315	0.43584522	0.50913464	1
FBgn003337:	tsu	-5.2461681	6.04982263	0.43575096	0.50918045	1
FBgn000304:	Pc	-5.1679627	5.98600084	0.43386737	0.51009737	1
FBgn003013(	RpS28b	0.84593286	8.4447339	0.43366835	0.51019441	1
FBgn003335	AIMP1	1.74419614	6.83577752	0.43319003	0.51042778	1
FBgn026806:	ltl	-2.3169341	4.92916824	0.4329899	0.51052548	1
FBgn000416!	up	2.48250945	3.21435076	0.43268217	0.51067578	1
FBgn003040(	CG1463	-5.0098158	5.47763244	0.43237577	0.51082549	1
FBgn000485!	ci	3.60965765	3.48182994	0.43236826	0.51082916	1
FBgn002893:	CG16890	-4.4562046	3.91780131	0.43173964	0.51113657	1
FBgn003287	CG2617	-4.2938882	4.18167718	0.43161137	0.51119933	1
FBgn002755:	CG10863	0.90906855	8.07951553	0.4315839	0.51121277	1
FBgn003491·	CG5554	-1.0508783	7.36883066	0.43155074	0.511229	1
FBgn003131!	CG4896	-2.2255784	4.53455961	0.43150239	0.51125267	1
FBgn003946	CG5500	-4.605093	4.57336676	0.43147587	0.51126565	1
FBgn000488:	Scsalpha1	-0.7475268	9.06834757	0.43122193	0.51138998	1
FBgn005216:	CG32163	-2.7666957	2.89240963	0.42995935	0.51200889	1
FBgn003514:	CG3402	-4.9483879	5.57799563	0.42826151	0.51284324	1
FBgn003294:	Tsp39D	1.43796709	6.61185742	0.42740725	0.51326392	1
FBgn003375(	CG13151	2.02446467	5.82799274	0.42663529	0.5136446	1
FBgn003754!	CG7878	1.18786661	7.8880652	0.42656626	0.51367866	1
FBgn003943	plum	-4.3636796	4.28067294	0.42627113	0.51382435	1
FBgn008527!	CG34250	1.33195519	6.61733547	0.42609931	0.51390919	1
FBgn003894	Sar1	0.8587568	7.98947943	0.42561296	0.51414949	1
FBgn005220(	CG32206	-3.4007955	4.10755472	0.4255693	0.51417107	1
FBgn003935	CG4743	-5.5369396	5.44542196	0.42508932	0.51440843	1
FBgn002853:	Sec71	-3.3881717	4.06036403	0.4249026	0.51450082	1
FBgn000003!	nAChRalpha2	-0.9906114	7.60120337	0.42309283	0.5153978	1
FBgn003443:	CG7461	-4.7726709	6.43242468	0.42246877	0.51570774	1
FBgn001334:	Syx1A	-0.896485	8.03149437	0.42239645	0.51574368	1
FBgn000569:	gcl	-2.9359652	4.57893418	0.42186325	0.51600878	1
FBgn026460	CaMKII	-0.7575163	8.67557522	0.42130139	0.51628839	1
FBgn000063:	Fas2	-3.174024	5.09513235	0.42093992	0.51646842	1
FBgn003543:	PHGPx	0.94652899	7.95244206	0.41967891	0.51709731	1

FBgn005029	CG30291	-4.9554977	6.68224053	0.41967228	0.51710061	1
FBgn003451	GNBP-like3	5.86373265	5.2795772	0.41849984	0.51768655	1
FBgn002866	VhaM9.7-c	-5.1529084	4.43958137	0.41786092	0.51800634	1
FBgn026188	l(2)35Be	-5.0253639	6.81280614	0.41780036	0.51803667	1
FBgn005154	CG31549	1.75029363	5.74069937	0.41722514	0.5183249	1
FBgn001669	Prosalpha5	-4.1988508	6.51164954	0.41545209	0.51921513	1
FBgn003668	CG6664	-4.7718605	6.65678059	0.41464768	0.5196199	1
FBgn003043	mRpL49	1.30165427	6.63133601	0.41419862	0.51984611	1
FBgn003366	Pl31	0.83733001	7.92608102	0.41401547	0.51993842	1
FBgn026443	lncRNA:CR43	-4.4485782	3.24346191	0.41336804	0.52026496	1
FBgn001040	RpL18A	0.75348886	9.67292057	0.41264557	0.52062977	1
FBgn003245	Pih1D1	-5.3651733	5.56157766	0.41256238	0.5206718	1
FBgn028650	Mpi	-4.8403078	5.38912627	0.4124529	0.52072713	1
FBgn003979	CG2246	-2.1412836	6.30767622	0.41182018	0.5210471	1
FBgn003769	Task7	4.42848995	6.58022893	0.41174672	0.52108427	1
FBgn003191	CG5973	1.26606486	6.72715237	0.41146056	0.52122911	1
FBgn005163	CG31635	-5.0647895	3.79235319	0.41013236	0.52190233	1
FBgn003654	sff	-5.7047454	5.60729957	0.40987495	0.52203298	1
FBgn003892	mRpL35	-5.2853794	5.62844973	0.40984024	0.52205061	1
FBgn002733	Rip11	-1.8628071	5.50562496	0.40979404	0.52207406	1
FBgn026098	vtd	0.83661438	7.91851636	0.40975638	0.52209318	1
FBgn002753	lili	2.04609176	4.9313577	0.40960913	0.52216796	1
FBgn003853	CG7523	1.72581627	5.71848791	0.40952213	0.52221216	1
FBgn003217	CG5846	-4.4516274	3.84486949	0.40928436	0.52233296	1
FBgn003843	Sdhaf3	-5.3586963	3.77887324	0.40913474	0.52240901	1
FBgn003355	RpS15Ab	1.3853924	6.49235058	0.4090416	0.52245635	1
FBgn000365	sw	-2.9806137	5.45911695	0.40863537	0.52266295	1
FBgn003106	CG14231	-2.3743617	4.52801595	0.40858813	0.52268699	1
FBgn005171	CG31715	1.1724614	6.88584188	0.40803218	0.52296999	1
FBgn003450	CG13868	-0.9771381	7.50266415	0.40793818	0.52301786	1
FBgn028347	wupA	-4.6416277	6.2337023	0.40764302	0.52316825	1
FBgn026093	par-1	1.79509936	5.84935583	0.40755937	0.52321088	1
FBgn002735	Amph	-2.0785759	6.4027897	0.40697924	0.52350672	1
FBgn004098	CG6115	-1.1042244	7.0489813	0.40666879	0.52366516	1
FBgn004063	CG4186	-4.9238222	3.77900412	0.40656553	0.52371788	1
FBgn002997	dpr14	-2.4225553	5.95320824	0.40652035	0.52374095	1
FBgn002899	Spn27A	1.1196206	6.99077949	0.40606853	0.52397174	1
FBgn003770	mura	-1.9802414	5.46532189	0.40540595	0.52431052	1
FBgn026310	DnaJ-1	-3.2393479	5.42580336	0.40530748	0.5243609	1
FBgn000071	flw	-1.5283193	6.25598106	0.40498403	0.52452646	1
FBgn003860	CG18600	1.7988604	5.61300433	0.40412761	0.52496525	1
FBgn003674	Vps60	0.78327913	8.24826578	0.40368926	0.52519009	1
FBgn003290	sky	-5.2864081	4.5774377	0.40283958	0.52562641	1
FBgn001372	phyl	-4.3245266	3.66698665	0.40251855	0.52579143	1
FBgn002625	cav	1.48997356	6.23454681	0.40173848	0.5261928	1
FBgn008543	CG34408	-2.2650363	3.38302466	0.40075463	0.52669979	1

FBgn026739[illegible]	Yeti	0.77215148	8.83323085	0.40042994	0.5268673	1
FBgn001745[illegible]	U2af38	1.0115736	7.29782181	0.40011534	0.5270297	1
FBgn003558[illegible]	CHMP2B	-4.6667919	6.01387903	0.39928698	0.52745772	1
FBgn002906[illegible]	Dd	-5.7785213	5.73061029	0.39863681	0.52779411	1
FBgn003381[illegible]	CG4679	-4.8341904	5.62315348	0.39778794	0.52823388	1
FBgn003600[illegible]	pall	-4.7024933	5.13628459	0.39728175	0.52849642	1
FBgn003867[illegible]	CG6040	4.52408471	4.3846055	0.39712449	0.52857804	1
FBgn005012[illegible]	CG30122	-5.3120056	4.57257381	0.39700372	0.52864073	1
FBgn002761[illegible]	eIF3j	0.83236159	8.28558848	0.39651849	0.52889276	1
FBgn000465[illegible]	fs(1)h	-1.745318	6.71421538	0.39647103	0.52891742	1
FBgn026185[illegible]	CG42788	-5.3729368	3.79124951	0.39595547	0.52918545	1
FBgn002354[illegible]	PIG-K	0.82075127	8.02094495	0.395914	0.52920702	1
FBgn002026[illegible]	mspo	1.43917941	6.30869599	0.39565313	0.52934275	1
FBgn002973[illegible]	CG6903	-2.03017	5.22966673	0.3950964	0.52963261	1
FBgn003129[illegible]	IA-2	-0.7839552	10.1824241	0.39388084	0.53026649	1
FBgn002991[illegible]	CG14434	-4.6010356	3.72104868	0.39363491	0.5303949	1
FBgn003752[illegible]	CG17816	-1.3017524	7.20702322	0.39343632	0.53049863	1
FBgn002915[illegible]	Las	-5.0269841	5.55937928	0.3931597	0.53064319	1
FBgn003034[illegible]	CG10347	-4.9694632	6.74923894	0.39315071	0.53064789	1
FBgn003585[illegible]	Atg18a	-2.1765212	6.36107516	0.39298157	0.53073631	1
FBgn002614[illegible]	CG12253	1.33981155	6.57603525	0.39292695	0.53076487	1
FBgn001418[illegible]	Oda	-0.6384899	10.7575315	0.39281189	0.53082504	1
FBgn000487[illegible]	bab1	-5.3926074	4.1172506	0.39227771	0.53110457	1
FBgn003365[illegible]	ths	4.04750721	5.03359804	0.39209368	0.53120092	1
FBgn003263[illegible]	Rpb11	0.91085992	7.63394194	0.39198638	0.53125712	1
FBgn003648[illegible]	CG6878	-1.3274158	6.6059441	0.39115359	0.53169365	1
FBgn026140[illegible]	sxc	-0.8333877	8.01842067	0.39067969	0.53194234	1
FBgn003808[illegible]	beat-Vc	-4.744298	6.66531752	0.39055294	0.5320089	1
FBgn001367[illegible]	mt:ND1	0.73815098	8.47263839	0.38970117	0.53245652	1
FBgn026334[illegible]	smash	-2.4347048	4.78716941	0.38932747	0.53265313	1
FBgn003226[illegible]	CG6094	-5.1542668	5.45597817	0.38899913	0.53282597	1
FBgn003085[illegible]	ND-24	0.72285743	8.74743639	0.38897879	0.53283668	1
FBgn003381[illegible]	Pex13	-5.5361613	4.89184251	0.38862503	0.53302303	1
FBgn002537[illegible]	Fpps	-4.4547373	4.55400684	0.38852771	0.53307432	1
FBgn003755[illegible]	CG9636	-0.7921525	8.25522878	0.38832915	0.53317898	1
FBgn003551[illegible]	CG1265	-0.9638422	7.39628544	0.38762125	0.53355243	1
FBgn004026[illegible]	Ugt37D1	-5.4815339	6.32781731	0.38755628	0.53358673	1
FBgn003753[illegible]	CG3223	-5.2104139	6.12623313	0.3872222	0.53376316	1
FBgn001402[illegible]	Rho1	0.92999025	9.09858346	0.38718618	0.53378219	1
FBgn003003[illegible]	CG1440	-2.735795	7.0213591	0.38711802	0.5338182	1
FBgn003738[illegible]	CG2023	-4.3813988	4.62668934	0.38652212	0.53413321	1
FBgn002614[illegible]	CDC45L	-4.8047649	4.78921072	0.38632328	0.5342384	1
FBgn003922[illegible]	Saf-B	-1.6273011	6.42852197	0.38622457	0.53429064	1
FBgn005247[illegible]	mthl8	-3.4927713	6.72099112	0.38541341	0.53472021	1
FBgn003806[illegible]	Octbeta2R	1.89734568	4.92749135	0.38479523	0.53504802	1
FBgn003929[illegible]	CG13663	1.77433992	5.76424247	0.38472647	0.5350845	1

FBgn026449	rdx	-1.5555894	6.21300663	0.38456331	0.53517109	1
FBgn005181	CG31812	-4.1044995	3.33759082	0.38441948	0.53524744	1
FBgn001158	e(r)	0.97234155	7.3414599	0.38433471	0.53529246	1
FBgn000010	Aprt	2.28382444	4.48564714	0.38433413	0.53529276	1
FBgn002709	Manf	0.98441561	7.33312251	0.38396275	0.53549003	1
FBgn003065	CG7872	-4.7370845	4.66940532	0.38357712	0.53569501	1
FBgn003039	Rab40	-2.4541787	5.67828604	0.38322818	0.53588061	1
FBgn026038	CG42514	-4.0257358	4.88590464	0.38297998	0.5360127	1
FBgn005166	CG31663	-3.1656569	4.89428885	0.38280128	0.53610784	1
FBgn026147	nero	1.32007932	6.58063751	0.38213891	0.53646075	1
FBgn026738	PyK	-0.6862219	9.54568952	0.38155544	0.53677196	1
FBgn004075	CG13018	1.75736132	5.31603035	0.38121606	0.53695314	1
FBgn006146	Ube3a	-5.2487325	5.30008238	0.38120019	0.53696162	1
FBgn003236	Dlg5	0.97508962	7.37298933	0.38058464	0.53729051	1
FBgn028592	Imp	-2.977594	5.97217032	0.37897649	0.53815151	1
FBgn005167	CG31673	-5.156802	5.23842575	0.37877127	0.53826157	1
FBgn003317	CG11127	-4.223651	5.62358554	0.3787505	0.53827271	1
FBgn002917	Fkbp59	-0.772149	8.14347433	0.37838287	0.53846997	1
FBgn003621	CG14130	-4.7162228	4.56562674	0.37697041	0.5392291	1
FBgn002759	drpr	5.55636008	4.74265736	0.37622312	0.53963152	1
FBgn003580	CG7506	-5.054909	5.91473959	0.37595618	0.53977541	1
FBgn003196	CG7231	-3.3631218	6.26609822	0.37576155	0.53988036	1
FBgn026251	Mi-2	-5.3799164	3.94617446	0.3753614	0.54009625	1
FBgn003154	Pif1	2.21385234	2.31982817	0.3750163	0.54028257	1
FBgn003284	Taf13	1.3917815	6.49365835	0.37486136	0.54036626	1
FBgn003746	Ufl1	-5.0906455	7.35065852	0.3746509	0.54047998	1
FBgn028678	TER94	-1.3855875	6.65004038	0.37404149	0.54080951	1
FBgn004213	CG18815	-0.6871896	9.08802692	0.37390333	0.54088427	1
FBgn003983	CG12054	-2.1318881	6.22271098	0.37363517	0.54102942	1
FBgn003940	RpL34a	1.17758814	6.77235503	0.37334869	0.54118458	1
FBgn002761	LManII	2.94382692	2.72786355	0.3722265	0.54179313	1
FBgn003670	CG6512	1.02625477	7.53151549	0.37220347	0.54180563	1
FBgn003099	CG7556	1.21761924	6.78572902	0.37211396	0.54185423	1
FBgn003761	Cks85A	-4.4733699	5.32444161	0.37204911	0.54188944	1
FBgn003801	CG10038	-4.8434794	5.18946014	0.37202795	0.54190093	1
FBgn004621	vig2	1.43166528	6.54435732	0.37194039	0.54194849	1
FBgn026641	wake	2.46391458	3.68385961	0.37153	0.54217147	1
FBgn008613	Prosalpha2	0.84903866	7.73339029	0.37131651	0.54228753	1
FBgn003229	CG6443	1.20137409	6.77441924	0.3712487	0.54232441	1
FBgn002752	LTV1	-1.441299	7.30721003	0.37096315	0.54247974	1
FBgn008637	poly	1.32403273	6.54726887	0.37022679	0.54288068	1
FBgn001367	mt:Cyt-b	-0.5632508	12.8083226	0.36923288	0.54342271	1
FBgn004038	CG32795	-5.1823263	6.00877362	0.36898339	0.54355893	1
FBgn003882	Syp	-1.0108322	7.79958488	0.36882376	0.54364612	1
FBgn000122	Hsc70-5	1.08229123	7.30047504	0.36881362	0.54365166	1
FBgn003106	Alr	-4.698559	4.81148056	0.36857654	0.5437812	1

FBgn003729	Sccpdh1	-0.9442919	7.45200583	0.36753462	0.54435119	1
FBgn001367	mt:ATPase6	0.57339488	11.9599132	0.36749392	0.54437347	1
FBgn005236	CG32368	1.16300351	6.83814268	0.36736797	0.54444246	1
FBgn008671	RpL30	0.91459738	7.60381723	0.36703668	0.54462398	1
FBgn000300	or	-4.26988	5.23074835	0.36680013	0.54475366	1
FBgn003690	TORIP	1.2185794	6.66190584	0.36633759	0.54500739	1
FBgn008539	CG34370	-2.1672486	3.56977204	0.3656022	0.54541126	1
FBgn002750	CG8878	-2.7253555	3.8907478	0.36556617	0.54543106	1
FBgn026432	Atg6	-1.3577178	6.85321084	0.36547797	0.54547954	1
FBgn026469	ens	-1.5054354	5.99799976	0.36541838	0.5455123	1
FBgn026716	asRNA:CR45	-5.1449585	3.54723596	0.36534682	0.54555164	1
FBgn003686	Gbs-76A	-4.8222645	3.84954362	0.36509034	0.54569269	1
FBgn003577	Sec63	-2.123228	4.57386141	0.36393475	0.54632905	1
FBgn001367	mt:CoIII	-0.5569506	12.3640858	0.36369833	0.54645942	1
FBgn026079	NaPi-III	-0.7878024	7.97708905	0.36337482	0.5466379	1
FBgn003627	CG10418	-4.6587381	5.19910083	0.36201621	0.54738861	1
FBgn026111	Xrp1	0.83646713	7.82436895	0.3617876	0.54751512	1
FBgn002574	PlexB	4.25074545	4.54032672	0.3614857	0.54768227	1
FBgn003282	CG10466	-3.7830887	3.1495656	0.36141125	0.54772351	1
FBgn001445	Ahcy	1.42227854	6.2667863	0.36133917	0.54776344	1
FBgn003199	PAPLA1	-5.3141419	4.8021846	0.36103031	0.54793458	1
FBgn003302	Not3	-4.3567233	6.4646286	0.36093311	0.54798847	1
FBgn003928	danr	-3.6152364	4.88496497	0.36090082	0.54800637	1
FBgn005321	CG33217	-4.6849056	5.45145412	0.36081588	0.54805347	1
FBgn025999	OtopLa	-1.6017054	6.76643254	0.35975993	0.5486396	1
FBgn025090	beat-VII	1.14871046	6.85013559	0.3595191	0.54877344	1
FBgn002993	CG8300	1.06972179	7.38172077	0.35912895	0.54899039	1
FBgn003244	PICK1	-2.8126839	5.47451449	0.35906424	0.54902639	1
FBgn003548	CG12605	-3.8176972	5.52502539	0.35891501	0.54910943	1
FBgn026106	LSm1	-4.69533	4.714461	0.35848504	0.54934881	1
FBgn003621	CG5946	-0.9453974	7.32494764	0.3583197	0.54944091	1
FBgn000457	Syn	-5.2690499	6.57290281	0.35716422	0.55008539	1
FBgn002551	fidipidine	1.75671961	6.03605378	0.35625754	0.55059208	1
FBgn026599	Doa	4.86901541	6.0700503	0.35563796	0.55093884	1
FBgn002887	nAChRalpha5	-1.7782525	6.36017257	0.35541433	0.5510641	1
FBgn003767	Aduk	-5.1927969	5.79079513	0.35533555	0.55110823	1
FBgn003587	BI-1	0.65898836	9.45590384	0.3544934	0.55158048	1
FBgn003707	CG7324	-4.082119	5.48502346	0.35407911	0.55181308	1
FBgn003523	CG12104	5.22841894	4.83199774	0.35336409	0.55221495	1
FBgn003650	CG7272	1.22720196	6.83650716	0.35321503	0.5522988	1
FBgn000485	Ac76E	-4.1683431	3.43263834	0.35310497	0.55236072	1
FBgn005210	CG32109	-1.2635206	6.93580696	0.35213854	0.55290504	1
FBgn004066	CG12848	1.16359737	6.7232587	0.3519396	0.55301722	1
FBgn026106	LSm7	1.67746616	5.72551868	0.35185081	0.5530673	1
FBgn003072	CG8939	-2.019296	4.57554888	0.35173152	0.55313459	1
FBgn000122	Hsp27	-2.3591488	4.96516232	0.35165226	0.55317932	1

FBgn003099	CG14194	1.53555335	5.98964015	0.35144375	0.553297	1
FBgn003790	CG5276	-4.1292422	5.51887826	0.35135675	0.55334611	1
FBgn000333	Sce	-4.9935242	5.42381351	0.35086758	0.55362243	1
FBgn003789	RpL24-like	-0.6877117	8.6217374	0.35063418	0.55375436	1
FBgn002989	RpL17	-0.7633847	8.00162527	0.35039944	0.5538871	1
FBgn002758	CG7601	2.01129349	5.04787513	0.34997021	0.55413	1
FBgn006422	Rdh	-4.2227237	4.73846238	0.34972018	0.55427157	1
FBgn026490	CG44098	-5.2768417	5.55526995	0.349657	0.55430736	1
FBgn000487	cdi	-1.7228066	5.1574033	0.34941839	0.55444255	1
FBgn001034	Map60	-1.5740647	6.45920072	0.34929749	0.55451108	1
FBgn008691	Rab26	-3.9975703	4.88789476	0.34887299	0.5547518	1
FBgn003896	pinta	1.80335658	6.29002857	0.34873056	0.55483261	1
FBgn003406	casp	-4.535181	4.76149771	0.34828595	0.55508502	1
FBgn003539	CG16753	1.54931947	5.99039901	0.34805069	0.55521867	1
FBgn003883	RpS30	0.71498237	8.65335019	0.34642951	0.55614129	1
FBgn026700	Ten-a	-1.2362455	7.89771219	0.3458492	0.55647226	1
FBgn002570	Srpk79D	-2.6792467	3.27668029	0.34580097	0.55649978	1
FBgn003345	Ntmt	-4.71227	5.62571935	0.34574732	0.5565304	1
FBgn003703	CG11456	-4.6778196	3.19747751	0.34538322	0.55673828	1
FBgn002988	CG3226	1.00459526	7.17890051	0.34534695	0.556759	1
FBgn003398	pcs	-5.2024325	5.25142128	0.34400127	0.55752867	1
FBgn005103	CR31032	-4.947226	5.84271974	0.34366454	0.55772158	1
FBgn008637	sra	0.93297198	7.49510142	0.34359512	0.55776137	1
FBgn002971	mRpL30	1.04633999	6.96612984	0.34242794	0.55843111	1
FBgn002316	SmD3	4.91652671	3.65250326	0.34196986	0.55869438	1
FBgn003887	Nelf-A	1.8137105	4.86378204	0.34196852	0.55869515	1
FBgn003832	Trax	1.34493659	6.60051432	0.34194999	0.55870581	1
FBgn000388	alphaTub84B	-0.6133686	10.3112016	0.34169927	0.55885001	1
FBgn004087	CG12994	-4.6174654	6.63216191	0.3415522	0.55893463	1
FBgn003273	Swip-1	-3.194104	5.35016184	0.34144645	0.55899549	1
FBgn003760	SLIRP2	-4.5654912	4.54984149	0.34136447	0.55904268	1
FBgn003768	RpL34b	0.94508665	7.37498979	0.34122255	0.55912439	1
FBgn002674	mRpL18	1.02388814	6.9984494	0.34112251	0.559182	1
FBgn003487	wmd	-4.7843161	5.88306709	0.3409313	0.55929215	1
FBgn003322	TTLL12	-1.4857198	5.9757896	0.34086601	0.55932977	1
FBgn008542	Epac	-3.7686836	4.1433703	0.34080742	0.55936353	1
FBgn003794	CG14721	-4.7757347	4.89478952	0.34047995	0.55955232	1
FBgn003156	CG16713	2.55954755	4.15356621	0.34031511	0.55964739	1
FBgn004345	CG5180	1.08346998	6.86923688	0.34012623	0.55975637	1
FBgn002309	cyc	-1.5355386	6.7287478	0.33998456	0.55983813	1
FBgn003085	stas	1.4369321	6.39116543	0.33989153	0.55989184	1
FBgn000122	Hsp26	-0.7176149	8.28354545	0.33923517	0.56027103	1
FBgn003551	CG1309	-5.0734005	5.9152443	0.33912764	0.5603332	1
FBgn028683	sov	-2.5451477	3.95031945	0.33861778	0.56062816	1
FBgn002202	l(2)k01209	-4.7231129	4.67301514	0.33852066	0.56068437	1
FBgn026641	sima	-3.2661928	4.46343091	0.3382123	0.56086294	1

FBgn005403[illegible]	CG34039	-2.9140289	3.89831501	0.3382023	0.56086873	1
FBgn003250[illegible]	CG9377	-0.9457915	8.52538299	0.33811172	0.56092121	1
FBgn001127[illegible]	RpL13	0.63692986	9.24328918	0.33782498	0.56108739	1
FBgn002580[illegible]	Paf-AHalpha	-1.6193966	5.42988915	0.3377297	0.56114263	1
FBgn003618[illegible]	CG6083	-5.237242	5.5636938	0.33724068	0.5614263	1
FBgn001038[illegible]	AP-1-2beta	-1.0281459	8.22593528	0.33715223	0.56147764	1
FBgn003552[illegible]	CG11586	-4.3564653	3.97362391	0.3368423	0.56165761	1
FBgn003302	CG10417	-1.6334787	6.88673974	0.33516969	0.56263073	1
FBgn001035[illegible]	Nc73EF	0.9490404	7.41111466	0.33514785	0.56264346	1
FBgn003559[illegible]	CG4603	1.3830283	6.20328485	0.33505745	0.56269615	1
FBgn000367[illegible]	CCT1	0.73462559	8.0617079	0.33476672	0.56286566	1
FBgn002545[illegible]	CREG	2.13351584	3.04259889	0.33466745	0.56292356	1
FBgn001398	His4r	0.54047468	12.1492597	0.3345406	0.56299757	1
FBgn002840[illegible]	Drep4	-5.0035517	4.49852925	0.33439191	0.56308435	1
FBgn001203[illegible]	Aldh	0.99503813	8.30634449	0.33421541	0.56318738	1
FBgn003061[illegible]	CG9065	0.84182671	7.59275586	0.33392111	0.56335927	1
FBgn000422[illegible]	nonA	1.12485104	6.82133962	0.33365403	0.56351534	1
FBgn002911[illegible]	Surf1	-4.6802634	5.38841513	0.33361216	0.56353982	1
FBgn003043[illegible]	Brms1	1.32123844	6.28282804	0.33359568	0.56354945	1
FBgn026646[illegible]	GckIII	-2.6010616	6.17758116	0.33343005	0.56364629	1
FBgn001533[illegible]	CG5861	-5.1696826	5.38725693	0.33335882	0.56368795	1
FBgn001029[illegible]	bys	1.14310552	6.8236149	0.3331073	0.5638351	1
FBgn003263[illegible]	Lrch	-3.3035071	4.39490286	0.33299413	0.56390133	1
FBgn002626[illegible]	bip2	-0.7810897	7.9067266	0.33291433	0.56394804	1
FBgn003029[illegible]	CG1737	-3.8213138	2.73281823	0.33280461	0.56401228	1
FBgn005398[illegible]	Mid1	-5.2430239	5.47466119	0.33279529	0.56401774	1
FBgn000491[illegible]	gol	-1.9532321	4.20000595	0.33236705	0.5642686	1
FBgn028621[illegible]	RpS12	0.66429515	9.30410559	0.33206585	0.56444517	1
FBgn003156[illegible]	CG10031	1.77820378	5.08750403	0.33172976	0.56464231	1
FBgn000049[illegible]	dsh	-4.0390716	2.64111613	0.33063934	0.56528287	1
FBgn003029[illegible]	CG11752	0.85202173	7.56708237	0.33008913	0.56560661	1
FBgn001966[illegible]	qm	-3.4970208	5.47905564	0.33007267	0.5656163	1
FBgn026126[illegible]	CG42613	-0.9696687	7.46883197	0.32993317	0.56569845	1
FBgn025921[illegible]	side-II	-3.5590721	5.21501439	0.32971642	0.56582612	1
FBgn001532	Ubc4	1.1401938	6.69028877	0.32961032	0.56588864	1
FBgn026286[illegible]	Ptr	-2.0082096	3.71642721	0.32952714	0.56593766	1
FBgn003104[illegible]	Naa20A	-5.1522313	5.14295134	0.32944104	0.56598841	1
FBgn002190[illegible]	RFeSP	-0.5742622	10.904131	0.32942579	0.5659974	1
FBgn026551[illegible]	mlt	-2.9922551	6.06216586	0.32915191	0.5661589	1
FBgn003317[illegible]	CG11141	-1.9410195	4.5856845	0.32887338	0.56632324	1
FBgn003985[illegible]	RpL6	0.63606128	10.1235262	0.32858385	0.56649416	1
FBgn003052[illegible]	Clic	1.15468227	7.02462637	0.32800595	0.56683562	1
FBgn002687[illegible]	MED18	1.25841679	6.43602484	0.32761888	0.56706455	1
FBgn003664	Smn	-4.8513882	6.35220426	0.32743152	0.56717543	1
FBgn006349[illegible]	GstE3	4.24019159	4.38843977	0.32713405	0.56735156	1
FBgn003076	CG9784	5.03021284	4.52544083	0.32631697	0.56783588	1

FBgn003577(	pst	-1.7715131	5.19094551	0.32629282	0.56785021	1
FBgn003189:	MICU1	-4.9715249	6.16349047	0.32626289	0.56786797	1
FBgn003173·	CG11147	2.63663597	3.89214861	0.32608389	0.56797419	1
FBgn002640·	Dronc	2.01503758	4.76253151	0.32589134	0.56808849	1
FBgn026096(	Baldspot	-1.4268245	7.57626503	0.3256899	0.56820812	1
FBgn001561·	CanB2	-0.8266396	7.7361574	0.32518906	0.56850577	1
FBgn002777!	VhaSFD	-0.6525832	9.43685061	0.32485636	0.56870366	1
FBgn002037(	TppII	-3.7321754	5.27204643	0.32466125	0.56881978	1
FBgn000053(	eas	-1.8503066	6.52529136	0.32441532	0.5689662	1
FBgn026183(	Msp300	-0.8804636	7.64605576	0.32399409	0.56921717	1
FBgn003175:	CG13999	-5.2378219	5.58040698	0.32372136	0.56937978	1
FBgn003602:	CG16717	-5.027822	5.83530763	0.32365836	0.56941736	1
FBgn003775:	CG12945	-4.5488659	4.34068354	0.3226776	0.57000293	1
FBgn003549'	CG14995	-3.4677973	3.34656271	0.3223783	0.57018187	1
FBgn005238(	SMSr	-2.0493609	6.50502318	0.32077593	0.57114171	1
FBgn002850'	CG3793	-0.9171133	7.40032472	0.32048756	0.57131479	1
FBgn003990!	ND-49	-0.6014083	9.84187957	0.32045579	0.57133386	1
FBgn003515:	RabX6	-4.8069132	4.63215392	0.32043904	0.57134392	1
FBgn003645·	CG17839	1.17447819	6.99674345	0.32028114	0.57143874	1
FBgn002564(	CG13369	1.26827328	6.53441702	0.32012491	0.57153259	1
FBgn000394	RpL40	0.61218821	9.96023741	0.31990479	0.57166487	1
FBgn026339:	hppy	-5.1284645	4.76806649	0.31958881	0.57185486	1
FBgn005154:	CG31548	1.79201896	5.98155531	0.31950966	0.57190247	1
FBgn003453·	maf-S	0.64438946	8.85216634	0.31943369	0.57194818	1
FBgn008542:	GramD1B	-3.4157334	4.33781632	0.31852424	0.57249587	1
FBgn003345	Marc	-4.7108748	5.72304539	0.31850809	0.57250561	1
FBgn001122·	heph	0.86289436	7.47128302	0.31790475	0.57286955	1
FBgn026064:	Rrp40	-4.4541422	4.62068798	0.31743921	0.57315067	1
FBgn003472:	CG6044	-0.857787	8.85128745	0.31723694	0.57327289	1
FBgn026295·	Rpb12	1.00369896	6.98034298	0.3170122	0.57340876	1
FBgn000007	amx	1.32814488	6.22642617	0.31691596	0.57346696	1
FBgn003818:	CG9286	-1.6287497	5.8760368	0.31673029	0.57357928	1
FBgn003229'	CG17124	-1.7090038	4.95462817	0.31639843	0.57378014	1
FBgn005180(	CG31800	-5.0619434	5.50078699	0.31616863	0.57391931	1
FBgn002914:	NHP2	1.28882576	6.42229205	0.31598325	0.57403162	1
FBgn025917	Pde9	-1.4154599	6.08918727	0.31518137	0.57451796	1
FBgn003260(	BuGZ	-3.1369334	5.38455915	0.31512825	0.5745502	1
FBgn003866:	euc	0.70454064	8.24433935	0.31490122	0.57468806	1
FBgn003487!	Rrp4	1.3749066	6.6146156	0.31461888	0.57485959	1
FBgn002583!	ND-B14.5A	-0.8313205	7.70479127	0.31303301	0.57582495	1
FBgn025997:	vlc	-3.900777	6.10665728	0.3128296	0.575949	1
FBgn003413(	DAT	-4.7200255	8.40672687	0.31279801	0.57596827	1
FBgn026481:	Pde1c	-2.0084535	4.95797421	0.3124199	0.57619902	1
FBgn003508:	CG2811	0.97999865	7.04760158	0.31226785	0.57629186	1
FBgn004108'	wun2	-1.8177569	4.30063177	0.31123547	0.57692304	1
FBgn003596:	Use1	-4.7634793	4.62015041	0.31099429	0.57707069	1

FBgn003911	CG10375	-4.5308967	4.07065958	0.31042871	0.57741723	1
FBgn003864	CG7718	1.64656116	5.98290129	0.31035168	0.57746446	1
FBgn003649	Toll-6	-3.4365972	5.16771849	0.31020286	0.57755573	1
FBgn003305	Tbce	3.33640355	4.56553125	0.31001967	0.57766812	1
FBgn004100	CG17715	-4.7869035	4.40552326	0.30997858	0.57769333	1
FBgn000321	rb	-1.825583	4.69201619	0.30914852	0.57820316	1
FBgn026273	AGO1	-0.9293822	7.16736546	0.30887091	0.57837387	1
FBgn001060	l(2)05714	1.22176707	6.55769817	0.3081026	0.57884685	1
FBgn005200	anne	-0.7698541	7.81785769	0.30802774	0.57889298	1
FBgn003198	CG8668	-2.423494	6.20616536	0.30742495	0.57926466	1
FBgn003603	Naa60	-4.32035	5.15885941	0.30700551	0.57952356	1
FBgn003331	Cirl	1.44961641	6.08622964	0.30674339	0.57968548	1
FBgn003498	mRpS17	1.18142486	6.52881422	0.30615934	0.58004659	1
FBgn003200	CG8372	0.90692187	7.22451865	0.30604544	0.58011706	1
FBgn002577	homer	-1.4970077	6.12918782	0.30583721	0.58024595	1
FBgn002749	Cdk5alpha	-0.6915561	8.19428526	0.30526756	0.58059883	1
FBgn003141	eys	1.21859052	6.61651146	0.30493231	0.5808067	1
FBgn000025	capu	-2.396426	4.5997724	0.30450641	0.581071	1
FBgn002091	RpL3	0.6364767	8.8059488	0.30428198	0.58121038	1
FBgn003071	Rrp47	1.18285912	6.49732495	0.30350425	0.58169387	1
FBgn002643	Grip163	-2.5481071	2.13952667	0.30292885	0.58205209	1
FBgn003643	CG9628	-4.7694205	5.07825877	0.30284988	0.5821013	1
FBgn003974	ZIPIC	1.20408158	6.43100076	0.30185143	0.58272406	1
FBgn003201	Ostgamma	0.79613759	8.02468496	0.30071913	0.58343194	1
FBgn003284	CG10747	-4.5644063	5.9250856	0.30070911	0.58343822	1
FBgn003962	CG11841	1.67098418	5.70053573	0.30042585	0.58361558	1
FBgn003692	CG7668	0.90599546	7.24538861	0.30041933	0.58361966	1
FBgn003145	Cwc25	-2.8650128	4.81292135	0.30041088	0.58362495	1
FBgn004158	eIF2D	-1.7355861	4.60000248	0.30033434	0.58367289	1
FBgn002897	betaggt-II	0.71325907	7.93476678	0.30026876	0.58371398	1
FBgn003851	CG5823	1.49813824	6.13536758	0.30019661	0.58375919	1
FBgn001178	mRpL12	-4.9118732	5.33096527	0.30004205	0.58385606	1
FBgn003633	CG11267	0.63501965	8.63492431	0.29988054	0.58395732	1
FBgn002749	wde	1.04166789	6.99627018	0.29971816	0.58405917	1
FBgn002233	dnk	-4.7734297	4.36113266	0.29866999	0.58471742	1
FBgn001039	Gtp-bp	0.92474621	7.11132733	0.29837602	0.58490231	1
FBgn003547	mge	0.84094291	7.5429753	0.29826216	0.58497395	1
FBgn003921	puf	-2.1306589	4.50751562	0.29755558	0.58541893	1
FBgn008703	Sbp2	1.26457743	6.27121144	0.29719574	0.58564581	1
FBgn026601	rudhira	0.82099218	7.50562309	0.29698399	0.58577939	1
FBgn026508	Cdep	-4.3663247	4.87298094	0.296562	0.58604581	1
FBgn003472	Mes4	1.50257805	5.67771028	0.29621132	0.58626739	1
FBgn003778	ohgt	-4.7726165	5.53110721	0.2959245	0.58644874	1
FBgn003873	CG11447	-1.9476236	5.97143158	0.29588263	0.58647523	1
FBgn005275	Snx27	3.85514684	4.9500322	0.29586925	0.5864837	1
FBgn000202	l(2)37Bb	-4.5347607	6.66580076	0.29575674	0.58655488	1

FBgn028347	und	1.03693412	6.78555934	0.29532403	0.5868288	1
FBgn003064	Alg14	1.51874872	5.40667936	0.29456627	0.58730911	1
FBgn005277	CG32772	1.49209463	5.52202907	0.2945652	0.58730979	1
FBgn003369	CG8860	1.52587394	5.27685336	0.29406843	0.5876251	1
FBgn026481	asRNA:CR44	-4.3034186	3.54659146	0.29402998	0.58764952	1
FBgn003504	Mmp1	-1.2664333	6.21366732	0.29399965	0.58766879	1
FBgn003149	alpha4GT1	-4.8873949	6.25514653	0.293981	0.58768063	1
FBgn003986	CG1890	-4.8821033	5.32289818	0.29369944	0.58785954	1
FBgn002758	GCS2alpha	-0.6891838	8.2261147	0.29333417	0.5880918	1
FBgn003618	CG7339	-3.4468786	4.62842996	0.29322395	0.58816192	1
FBgn001368	mt:ND2	0.73938536	7.81281469	0.29278275	0.58844277	1
FBgn025079	alphaSnap	-0.6060608	9.13262142	0.29243018	0.58866741	1
FBgn026398	Stacl	0.98696911	7.49888838	0.29224918	0.58878279	1
FBgn008528	Blos3	1.58932754	5.09392845	0.29223082	0.5887945	1
FBgn002546	Bap60	0.90195608	7.13501485	0.292019	0.58892961	1
FBgn001603	Vsp37A	0.67091713	8.2293198	0.29198799	0.58894939	1
FBgn003306	Ars2	-1.2698736	6.6452623	0.29198677	0.58895016	1
FBgn005242	CG32428	1.51143952	5.97580163	0.29178274	0.58908037	1
FBgn000368	Tbp	-4.83797	6.12770831	0.29176381	0.58909245	1
FBgn002754	CG1927	1.02321876	6.85595612	0.29135563	0.58935312	1
FBgn004002	MED21	-1.0502776	6.79011736	0.29134784	0.5893581	1
FBgn004703	ND-13B	0.6372481	8.4706546	0.29109326	0.5895208	1
FBgn003167	senju	1.74645787	5.22567175	0.29102229	0.58956617	1
FBgn026733	Glut4EF	-2.7641402	7.01482089	0.29101148	0.58957308	1
FBgn004076	luna	-3.4535112	5.39996406	0.29068848	0.58977967	1
FBgn026391	COX8	0.56959943	10.0253023	0.29015192	0.59012319	1
FBgn026295	mt:ND4	-0.552275	10.613065	0.28987417	0.59030118	1
FBgn005254	CG32549	-1.4638746	6.51793755	0.28929734	0.59067116	1
FBgn003030	Spase25	-3.4265926	6.25893512	0.28918991	0.59074012	1
FBgn025923	CG42337	-3.8359431	5.89310867	0.2888901	0.59093266	1
FBgn003125	Ent1	-4.1730576	3.05206092	0.28838822	0.59125526	1
FBgn026143	CSN8	1.52833725	5.43613862	0.28813545	0.59141786	1
FBgn000307	Pgi	-0.5976046	9.07053742	0.28777039	0.59165287	1
FBgn000487	enc	-1.3684693	6.36605869	0.28745388	0.59185678	1
FBgn003229	CG6724	-2.3322856	5.19529365	0.28737267	0.59190913	1
FBgn025978	zld	-1.7183906	4.73352583	0.2869371	0.59219003	1
FBgn003343	CG1902	0.91275583	7.1225694	0.28676008	0.59230427	1
FBgn026323	Cdk7	-1.7923194	4.82053918	0.28675128	0.59230995	1
FBgn000334	sd	-1.5901206	5.05851705	0.28667579	0.59235868	1
FBgn003235	CG6287	-1.3012842	6.08942444	0.28644541	0.59250745	1
FBgn003630	CG10984	5.01166687	5.6338221	0.28619098	0.59267184	1
FBgn003567	CG6610	1.65175822	4.85299305	0.28580671	0.5929203	1
FBgn003782	CG14683	0.88986648	7.53596844	0.28518872	0.59332034	1
FBgn026159	RpS6	0.62962738	9.06813703	0.28511595	0.59336748	1
FBgn003806	Snx3	0.70583719	7.89041759	0.28473997	0.59361117	1
FBgn003633	mRpL20	1.03221812	6.75860373	0.28458219	0.5937135	1

FBgn000204	swm	1.65544748	6.49758881	0.28384404	0.59419269	1
FBgn003165	jet	-4.264192	5.48361703	0.28366638	0.59430815	1
FBgn003436	CG5335	-2.4378713	6.54357072	0.28324536	0.59458194	1
FBgn002562	CG4025	-3.6934314	5.77099949	0.28315982	0.5946376	1
FBgn005152	CG31523	-2.3784224	5.82887255	0.28315954	0.59463778	1
FBgn002952	CG3176	-4.004478	2.70295543	0.28314247	0.59464889	1
FBgn008655	Ubi-p5E	0.66620754	8.33950811	0.28297937	0.59475505	1
FBgn001129	l(2)efl	2.98298664	3.35182808	0.28275514	0.59490106	1
FBgn003646	CG5114	-3.8258301	6.08038757	0.28220608	0.59525891	1
FBgn002607	UBL3	-0.6544717	8.72027173	0.28183349	0.59550199	1
FBgn026323	bel	-2.6705774	5.46468217	0.28179581	0.59552659	1
FBgn025916	CG42271	-4.7102854	5.47178195	0.28167584	0.59560491	1
FBgn003489	mRpL43	0.83243001	7.34490944	0.28142646	0.59576779	1
FBgn005163	CG31638	-2.609388	5.81373197	0.28130969	0.59584408	1
FBgn002187	Xbp1	-1.1055145	7.33991245	0.2807502	0.59620993	1
FBgn003104	Sec61gamma	-0.7888799	7.73529863	0.28051885	0.59636135	1
FBgn003346	CG1418	1.74047463	5.12591113	0.2801808	0.59658274	1
FBgn002837	jbug	4.03528727	4.35713457	0.27996822	0.59672205	1
FBgn026159	RpS29	0.61515739	9.11003516	0.27993162	0.59674604	1
FBgn002202	Vps25	1.2587901	6.15246289	0.27962718	0.59694568	1
FBgn003883	CG17272	1.24139198	6.19278521	0.27960941	0.59695734	1
FBgn000045	Dip-B	-1.32812	6.90474364	0.27956685	0.59698526	1
FBgn005235	Vps11	1.62152765	5.86215714	0.27950445	0.5970262	1
FBgn000038	csw	-1.8698371	6.40183807	0.2788157	0.5974785	1
FBgn025924	CG42342	-4.9095195	5.78692544	0.27825509	0.59784718	1
FBgn026672	Trs20	0.95276513	7.01657269	0.27810942	0.59794306	1
FBgn003819	CG9922	-4.9443572	5.40058472	0.27800251	0.59801344	1
FBgn003691	Pfdn6	-1.1590443	6.54618326	0.27788908	0.59808814	1
FBgn004301	AP-2sigma	0.67791509	7.99023281	0.27776231	0.59817165	1
FBgn003378	CG17019	-1.2336787	6.22450085	0.27757449	0.59829541	1
FBgn026666	Sem1	0.84951106	7.21603938	0.27711974	0.59859529	1
FBgn003346	Sec24AB	-3.2411986	3.52847259	0.27690117	0.59873953	1
FBgn005005	lncRNA:CR30	1.53417209	5.46071186	0.2767231	0.5988571	1
FBgn003682	CG3961	-3.3462072	4.71285459	0.27667732	0.59888734	1
FBgn001129	Taf11	-4.4223477	4.42959803	0.27653381	0.59898213	1
FBgn000031	Chc	1.04824107	7.79648814	0.27638959	0.59907743	1
FBgn003502	Start1	-4.4735659	4.8474919	0.27607456	0.59928571	1
FBgn003334	MrgBP	-3.8235293	4.73446561	0.27540813	0.59972681	1
FBgn003681	Atg3	0.72131269	7.87866404	0.27512575	0.59991392	1
FBgn003344	CG1648	-0.9569441	7.04215121	0.27508079	0.59994372	1
FBgn003847	CG5220	1.53293742	5.33493979	0.27500052	0.59999693	1
FBgn003109	CG9578	-4.9131885	6.0684055	0.27493295	0.60004174	1
FBgn003396	ND-B15	0.6827114	8.00438212	0.27448639	0.60033802	1
FBgn001612	CaMKI	0.62363591	8.73671155	0.27432859	0.60044279	1
FBgn005244	CG32448	1.42406642	5.58968442	0.27422357	0.60051253	1
FBgn002970	CHOp24	0.6044849	8.77238334	0.27379488	0.60079742	1

FBgn003354	CG12935	-4.4244804	4.31565536	0.27310909	0.60125375	1
FBgn001047	tutl	1.24793789	6.6518638	0.27278027	0.60147282	1
FBgn002432	NK7.1	-1.680717	4.63439993	0.27259559	0.60159592	1
FBgn026651	Dpit47	0.98198128	6.82432068	0.27231371	0.60178393	1
FBgn002062	Osbp	-4.2357658	6.92819792	0.27182081	0.60211296	1
FBgn003032	Vlet	-4.426469	5.4204208	0.27154702	0.6022959	1
FBgn003183	COX5B	0.54201831	10.3091608	0.27125841	0.60248887	1
FBgn002786	Tsp96F	0.79307576	7.50693458	0.27098934	0.60266888	1
FBgn003985	CG1635	-3.7872576	2.81585121	0.27054406	0.60296704	1
FBgn003176	IPIP	-4.876276	5.30979016	0.27010711	0.60325993	1
FBgn005161	nolo	-1.9820465	5.69029748	0.27002572	0.60331452	1
FBgn003313	CR12842	1.7326852	3.88875246	0.26972894	0.60351366	1
FBgn001174	Ank	-4.212113	6.31051896	0.26960748	0.6035952	1
FBgn005285	CG32850	1.06647369	6.80183763	0.26959665	0.60360247	1
FBgn003384	cbc	-4.0937858	6.03267998	0.26944764	0.60370254	1
FBgn001040	RNaseX25	-4.5600769	6.56932689	0.26876413	0.60416201	1
FBgn001579	Rab14	0.65390605	8.10198661	0.26866325	0.60422989	1
FBgn003915	mRpS24	-4.6764419	4.61779154	0.26842628	0.6043894	1
FBgn004307	MESK2	0.67645685	7.90140063	0.26816471	0.60456557	1
FBgn003371	Den1	0.97451985	6.87460908	0.26796668	0.60469901	1
FBgn003529	Mfap1	1.203185	6.38725376	0.26785051	0.60477733	1
FBgn001577	rin	-1.1551543	6.4768694	0.26711859	0.60527123	1
FBgn000341	skd	-4.0779441	4.66958579	0.26683167	0.60546508	1
FBgn001101	SsRbeta	1.12531833	6.44865889	0.26661267	0.60561313	1
FBgn004052	COX7A	0.53821696	10.2333207	0.26604704	0.60599587	1
FBgn026339	Ih	-1.1216889	7.21529934	0.26589335	0.60609995	1
FBgn003765	Iru	-1.521977	6.56782174	0.26585671	0.60612477	1
FBgn003163	Fnta	1.14813468	6.37857854	0.26504932	0.60667224	1
FBgn004380	CG32032	0.83185348	7.28546141	0.2644531	0.60707719	1
FBgn003405	bug	-2.414705	4.26716032	0.2643785	0.6071279	1
FBgn000109	Gdh	-0.6515924	8.05826085	0.26427292	0.60719968	1
FBgn002892	NC2beta	1.7691419	4.89339508	0.26418765	0.60725767	1
FBgn002898	tant	-3.9773626	5.45208973	0.26415708	0.60727847	1
FBgn003897	Nrx-1	-1.7148377	5.60666991	0.26353429	0.60770238	1
FBgn003377	Cyp9h1	-3.9060241	5.70881703	0.26335558	0.60782414	1
FBgn008397	CG34136	1.45635692	5.2150116	0.2632126	0.6079216	1
FBgn025998	Uxt	-3.7505331	4.11527487	0.26316261	0.60795568	1
FBgn004170	yellow-g	-4.293317	5.17852511	0.26306346	0.60802329	1
FBgn001756	ND-75	-0.6493418	9.09162208	0.26250961	0.60840124	1
FBgn003933	MED28	-4.4146912	4.98735975	0.26170644	0.60895022	1
FBgn003009	CG12056	-4.8191605	5.58473642	0.26158126	0.60903589	1
FBgn002838	cyp33	1.64952446	6.2936735	0.26128844	0.60923636	1
FBgn003072	Nipsnap	-2.0909818	6.69233542	0.26044336	0.60981572	1
FBgn001603	mael	1.62382857	5.34284834	0.26018132	0.60999561	1
FBgn003160	CG3652	0.70747262	7.73886812	0.26014736	0.61001894	1
FBgn000277	Mlc1	0.82658056	7.23470764	0.26011822	0.61003895	1

FBgn026138	mRpL37	-1.2528576	7.13140223	0.25984935	0.61022367	1
FBgn003392	Tfb1	1.52926274	5.90077451	0.25955256	0.61042772	1
FBgn026065	CG42540	-1.6266463	6.85177373	0.25943412	0.61050919	1
FBgn000488	tws	-1.2551113	6.11062434	0.25862827	0.61106412	1
FBgn026178	Ank2	-0.9562345	7.07220272	0.2585408	0.61112442	1
FBgn003308	geminin	-3.7675421	5.46807738	0.25804912	0.61146362	1
FBgn003744	Zif	0.93554022	7.35849515	0.25797439	0.6115152	1
FBgn000344	snf	0.69925881	7.73081209	0.25734908	0.61194725	1
FBgn003111	CG1695	-1.7160905	5.27618998	0.25728616	0.61199076	1
FBgn003746	Dpck	1.18192088	6.24588112	0.25696582	0.61221238	1
FBgn003781	CG14688	1.59370148	5.6640175	0.25691928	0.6122446	1
FBgn000527	Sam-S	0.60646061	8.50316509	0.25669529	0.61239968	1
FBgn003495	CG3860	-5.4121151	5.97778289	0.25661464	0.61245554	1
FBgn008668	Desat1	0.58318671	9.72397042	0.25654886	0.61250111	1
FBgn025922	ATP8A	-1.5876712	5.08558181	0.25640377	0.61260165	1
FBgn002708	MetRS-m	1.8130453	4.84791798	0.25638459	0.61261494	1
FBgn000559	RpL7	0.56878021	9.30964486	0.25638389	0.61261543	1
FBgn026150	lncRNA:CR42	2.86942327	3.21116797	0.25534595	0.61333573	1
FBgn005240	CG32407	1.60537128	5.44086599	0.25514354	0.61347642	1
FBgn005039	CG30394	1.77001152	4.96277653	0.25509936	0.61350713	1
FBgn003102	Naa15-16	-3.599378	6.2028284	0.25480148	0.61371432	1
FBgn002662	tacc	-1.5444327	6.1754094	0.25476865	0.61373716	1
FBgn001030	brat	1.36795314	5.81094092	0.25469676	0.61378719	1
FBgn008544	CG34417	-1.7859811	4.15837379	0.25455108	0.6138886	1
FBgn026725	CG32700	-1.2295845	6.28816724	0.25437952	0.61400807	1
FBgn000057	ena	-2.2581652	4.80553469	0.25410992	0.61419591	1
FBgn004022	DCTN5-p25	-4.9402845	4.82967779	0.2538954	0.61434547	1
FBgn008667	fne	-0.9313646	8.699734	0.25387804	0.61435757	1
FBgn003302	hrm	-1.0594058	7.52476343	0.25376149	0.61443886	1
FBgn003734	CG2931	-2.485816	5.54186196	0.25365579	0.61451261	1
FBgn003735	CG12171	-5.1560643	6.3110679	0.25319895	0.61483155	1
FBgn002708	TyrRS	-3.3835847	4.45331325	0.25307975	0.61491483	1
FBgn000199	mRpL4	1.17270419	6.24107023	0.25302213	0.61495509	1
FBgn003575	MED4	-2.828378	4.82410771	0.25278875	0.61511825	1
FBgn003449	CG9143	1.56970107	6.95809166	0.25240856	0.61538422	1
FBgn026153	cdm	0.99009435	7.68019863	0.25236775	0.61541278	1
FBgn026046	CG12163	0.58428839	8.60353334	0.25229412	0.61546433	1
FBgn003777	CG11722	-1.0445603	6.55799802	0.25226419	0.61548528	1
FBgn026300	SERCA	-1.3097115	5.79732921	0.25216899	0.61555195	1
FBgn002321	Mnt	-1.5424428	5.46375769	0.25212057	0.61558586	1
FBgn003264	GCS2beta	-2.0258885	5.07885848	0.25199231	0.61567571	1
FBgn003689	PIG-F	-1.3687614	5.75361405	0.25191903	0.61572706	1
FBgn001743	Nelf-E	0.75780252	7.58539695	0.25181055	0.61580309	1
FBgn003599	phol	2.18613962	3.07534165	0.25167733	0.61589649	1
FBgn008660	side-VIII	-2.5683207	4.68534535	0.25158928	0.61595823	1
FBgn003524	metl	-4.5717593	5.67001178	0.25144183	0.61606167	1

FBgn003930:	Nup358	3.82505567	3.10089907	0.25139887	0.61609181	1
FBgn026111!	Prp19	1.15004793	6.42535488	0.25078663	0.61652173	1
FBgn003597(	PGRP-LC	3.19939033	4.82605949	0.25025926	0.61689258	1
FBgn003168:	CG4230	-0.5913414	8.44924765	0.24866195	0.61801882	1
FBgn005267(	stx	-2.7985203	5.5355179	0.24862638	0.61804395	1
FBgn002977	CG12730	0.86955177	7.17196731	0.24847846	0.61814849	1
FBgn002971	Usf	-2.3598621	4.29123863	0.24811332	0.61840669	1
FBgn026703:	mamo	-1.2338448	7.7732406	0.24789693	0.61855982	1
FBgn003287	CG2611	-4.1595167	3.7007942	0.24631763	0.61967997	1
FBgn003391:	RpS23	0.68566696	7.92912172	0.24619769	0.61976522	1
FBgn003034:	CG1847	-3.758958	6.65690562	0.24605391	0.61986745	1
FBgn004402(	Roc2	0.71403672	7.60700448	0.24581624	0.62003652	1
FBgn002586(	CalpB	-2.1956221	5.33392286	0.24527711	0.62042042	1
FBgn026400	bru3	-0.9226619	8.31343738	0.24479456	0.62076448	1
FBgn002794:	ppl	1.17743474	6.16362292	0.24444766	0.62101208	1
FBgn002842!	I-2	-1.7794765	5.99643012	0.24428564	0.62112779	1
FBgn002338:	Dap160	-1.2444119	5.96100098	0.24414833	0.6212259	1
FBgn002631:	X11L	4.81340364	4.52665039	0.24411674	0.62124847	1
FBgn026188:	osa	-1.7288182	4.91457871	0.24407471	0.62127851	1
FBgn003141!	CG15390	-4.1782573	2.64053582	0.24393358	0.6213794	1
FBgn003781:	Rrp46	-3.2517031	4.38701172	0.24379077	0.62148153	1
FBgn003898·	AdipoR	0.64268593	8.71619439	0.24344291	0.62173045	1
FBgn026311	cac	-2.9072881	6.3192576	0.24242712	0.62245859	1
FBgn004507:	Stim	-1.8918416	5.46037815	0.24229781	0.62255142	1
FBgn001528(	Rala	1.13334522	6.57375613	0.24222231	0.62260563	1
FBgn025911	Ndae1	-1.7710408	4.51057314	0.24209686	0.62269574	1
FBgn003702	CG11399	-3.9090056	3.80308336	0.24185101	0.6228724	1
FBgn002750'	CG1344	0.80615658	7.19848197	0.24113539	0.62338728	1
FBgn003424:	CG14483	-1.7490548	2.79676743	0.24067661	0.62371786	1
FBgn002321:	eIF4G1	-0.8294905	7.6023087	0.24033836	0.62396184	1
FBgn003428:	Mapmodulin	-0.6030314	8.23465283	0.23989154	0.62428446	1
FBgn003867:	CG6013	0.93605707	6.78078509	0.23961572	0.6244838	1
FBgn026559:	CG44422	-4.4889108	4.35606137	0.23953946	0.62453894	1
FBgn003122!	CG3436	-4.2378831	5.86836171	0.23944201	0.62460941	1
FBgn026335:	CG11000	-3.9098695	5.82073708	0.23882392	0.62505684	1
FBgn000122·	Hsp23	0.686939	7.66528197	0.23844003	0.62533508	1
FBgn008703:	AGO2	-2.6586215	6.71105763	0.23835024	0.62540021	1
FBgn003596·	Dhpr	0.7431168	7.46270699	0.23810168	0.62558056	1
FBgn002317:	Prosalpha7	-1.0864709	6.70906306	0.23761522	0.62593386	1
FBgn026127(	SelD	1.35865317	6.35975476	0.23723364	0.6262113	1
FBgn001346'	igl	-0.6770818	8.21748321	0.23698226	0.62639424	1
FBgn003054:	CG11590	-3.1745568	5.47701868	0.2367842	0.62653845	1
FBgn003087(	CG6762	-4.3906239	3.28654572	0.23662456	0.62665473	1
FBgn002749(	epsilonCOP	0.80329179	7.16954501	0.23652577	0.62672673	1
FBgn003753:	CD98hc	-0.6195612	8.28321101	0.23651746	0.62673279	1
FBgn003501(	CG4612	-3.9521692	5.74954167	0.23626232	0.62691879	1

FBgn003735	MED27	1.34001091	5.600642	0.23593215	0.62715969	1
FBgn002632	Mad1	1.26271717	6.50715268	0.23563395	0.62737744	1
FBgn003698	CG5282	-0.6949927	7.61991767	0.23528709	0.62763094	1
FBgn005116	SKIP	4.726146	5.45038886	0.23524531	0.6276615	1
FBgn026529	Dscam2	-1.8071816	5.59937561	0.23523044	0.62767237	1
FBgn025075	CG42235	-1.0191368	7.46802683	0.23507758	0.62778417	1
FBgn000028	cg	-1.0555763	6.66722841	0.23486601	0.627939	1
FBgn003008	HP1b	1.67049687	6.43821515	0.23452761	0.62818681	1
FBgn008563	CG41378	1.21395553	6.35851227	0.23452575	0.62818817	1
FBgn003348	mRpL42	1.08531677	6.39802069	0.23414408	0.62846794	1
FBgn002023	14-3-3epsilo	-0.4778344	11.0907426	0.23402351	0.62855637	1
FBgn003994	CG17163	-2.5886168	5.28605665	0.23399416	0.62857791	1
FBgn005046	Pgant9	-3.0213116	2.63077197	0.23398231	0.6285866	1
FBgn026430	orb2	-3.3326578	6.6542287	0.23373216	0.62877019	1
FBgn026210	lncRNA:CR42	-4.7712959	4.49623126	0.23352769	0.62892035	1
FBgn001368	mt:ND3	0.91049876	6.88922243	0.23301995	0.62929356	1
FBgn003190	CG5177	1.55246228	5.19328692	0.23260138	0.62960162	1
FBgn003104	MKP-4	-2.7801624	3.50768883	0.232583	0.62961516	1
FBgn002951	312	1.43088463	5.51565458	0.23257016	0.62962461	1
FBgn003391	CG8531	-2.7013717	6.30995606	0.23222461	0.6298792	1
FBgn001757	Mo25	-1.2022468	5.9975461	0.2321392	0.62994216	1
FBgn008538	CG34355	-4.3388834	4.54816046	0.23199343	0.63004965	1
FBgn003197	Sirup	0.60358009	8.1466779	0.23196991	0.630067	1
FBgn003982	CG15561	-4.619649	4.63939258	0.231334	0.63053645	1
FBgn003271	CG10600	1.72850051	4.75669916	0.23130206	0.63056005	1
FBgn003572	CG10075	-1.2777338	5.7652294	0.23119059	0.63064243	1
FBgn000563	faf	3.14140931	3.08451378	0.23093272	0.63083309	1
FBgn000042	SmF	0.93886879	6.78568573	0.23078069	0.63094556	1
FBgn003424	CG6484	1.76396645	4.11611507	0.23015613	0.63140808	1
FBgn003971	Zip99C	-1.4599765	5.93281045	0.23002601	0.63150454	1
FBgn002868	Rpt1	0.60243654	8.12880431	0.22995347	0.63155833	1
FBgn003104	CG14210	1.44537969	4.94681688	0.22962248	0.6318039	1
FBgn002533	CG4882	0.84962041	7.09412924	0.22955468	0.63185422	1
FBgn003306	CG7849	-4.1593503	3.97705731	0.22934653	0.63200879	1
FBgn005186	Qtzl	-4.6171372	4.52407123	0.22925659	0.63207561	1
FBgn003804	Scgbeta	0.6399643	7.83386981	0.22899732	0.6322683	1
FBgn000440	RpS14a	0.54412048	9.14005075	0.22843141	0.63268936	1
FBgn004432	Cka	-1.0100942	6.57230045	0.22734549	0.63349913	1
FBgn003491	Snap29	0.95200621	6.63907494	0.22730147	0.63353201	1
FBgn003708	barc	3.34196571	6.15068529	0.22681048	0.63389896	1
FBgn003976	CG15536	1.32987865	5.36667797	0.22663146	0.63403288	1
FBgn003604	CG8177	-2.6111723	5.50786082	0.22654163	0.6341001	1
FBgn025973	CG42390	-2.6033723	3.48487688	0.22642877	0.63418458	1
FBgn003749	CG1227	1.45268997	5.68619259	0.22634209	0.63424948	1
FBgn026274	Evi5	-1.9035447	5.69620031	0.22558652	0.63481585	1
FBgn026186	whd	-1.4949829	6.13639708	0.22546856	0.63490437	1

FBgn003693	CG14186	2.64853954	5.52540278	0.2250972	0.63518325	1
FBgn003962	Slu7	-4.5168416	4.04160201	0.22429776	0.63578458	1
FBgn026160	RpL37A	0.63882152	8.13709179	0.22403356	0.63598359	1
FBgn001575	RpL9	0.58274846	8.26461087	0.22396479	0.63603541	1
FBgn003554	CG15019	1.03673529	6.4339624	0.22321546	0.63660075	1
FBgn003269	MESR3	-2.0805208	5.22946359	0.22314987	0.63665029	1
FBgn005167	CG31676	-0.6752175	7.6182786	0.22306471	0.63671462	1
FBgn003890	RpI12	-4.0190282	3.93033475	0.22241968	0.63720239	1
FBgn002676	Trap1	0.68928447	7.56083196	0.22234394	0.63725973	1
FBgn003816	CG9588	1.07579556	6.34463589	0.22163639	0.63779589	1
FBgn001675	Usp47	-2.4417032	6.72571006	0.22143245	0.63795062	1
FBgn002484	Pcd	0.67301478	7.62970644	0.22141904	0.63796081	1
FBgn003404	tun	-2.9613231	5.20378351	0.22107071	0.6382253	1
FBgn003097	Flacc	-2.1050461	7.07331502	0.22090981	0.63834756	1
FBgn003508	CG3776	0.68950355	7.63206063	0.22077526	0.63844984	1
FBgn003199	Acbp1	0.66976696	7.64746226	0.22053564	0.63863209	1
FBgn005107	Lerp	-1.3860001	5.84039886	0.22029924	0.63881201	1
FBgn000009	aop	-1.6372194	5.11176799	0.22023035	0.63886446	1
FBgn002950	Tsp42Ee	0.5161592	9.21840531	0.2201438	0.63893037	1
FBgn026065	CG42542	0.80398731	7.66572934	0.21981095	0.639184	1
FBgn008541	CG34383	-3.5831449	4.02666359	0.21961356	0.63933453	1
FBgn003818	CG9297	-2.39026	3.02447314	0.21960596	0.63934032	1
FBgn003323	CG12159	-4.089481	5.8736673	0.21959371	0.63934967	1
FBgn008644	mib2	-1.4119584	5.31642766	0.2192071	0.63964474	1
FBgn003772	SpdS	1.74747674	3.75623444	0.2191348	0.63969995	1
FBgn003224	CG5188	-4.5664548	5.40911705	0.21908619	0.63973708	1
FBgn001438	sty	0.93924834	6.75745635	0.21906484	0.63975339	1
FBgn000325	r-l	2.69629567	6.92495213	0.21873207	0.64000772	1
FBgn003603	CG16711	3.55657327	4.04501851	0.21866821	0.64005655	1
FBgn008518	CG34159	0.80700393	7.39375632	0.2185782	0.6401254	1
FBgn003793	Ho	0.95466628	6.68524623	0.21850948	0.64017798	1
FBgn000562	ple	-0.5333835	9.03928264	0.21844429	0.64022786	1
FBgn005006	CG30069	3.17495003	4.24794426	0.21834975	0.64030021	1
FBgn001010	comm	-1.4146276	5.42061643	0.21830735	0.64033267	1
FBgn002640	Nipped-B	-0.6534102	7.68427621	0.21794118	0.64061313	1
FBgn026210	lncRNA:CR42	-0.6632395	7.76201128	0.21772816	0.64077642	1
FBgn003043	CG4645	-1.0338002	6.40688309	0.21770283	0.64079585	1
FBgn025914	CG42260	1.00124854	6.49181557	0.21743922	0.64099807	1
FBgn003075	CG13014	-3.4064176	5.38475087	0.21722161	0.64116512	1
FBgn003334	CG13751	0.88554499	6.79416579	0.21712726	0.64123757	1
FBgn003658	MED10	0.75041115	7.19505477	0.21702661	0.64131489	1
FBgn003079	CG4829	-1.3911405	5.5353048	0.21680652	0.64148404	1
FBgn002962	eIF3g1	0.73671876	7.31075339	0.21632601	0.64185369	1
FBgn005268	CG32683	-4.3804903	3.20828045	0.21609737	0.64202976	1
FBgn000371	tkv	-1.5447621	5.40357747	0.21565593	0.64237001	1
FBgn003700	CG5059	0.65918768	7.63761946	0.21543022	0.64254414	1

FBgn003542	eIF1	0.52993519	9.04394716	0.21525417	0.64268004	1
FBgn003242	atilla	-4.5407751	5.39628085	0.21461546	0.64317366	1
FBgn001755	Pdk	0.83389853	6.96301079	0.21427597	0.64343639	1
FBgn003598	CG4452	4.29064492	4.86000627	0.2141733	0.6435159	1
FBgn000228	l(3)73Ah	1.04149835	6.32229034	0.21371637	0.64387001	1
FBgn026044	Not10	1.18608022	6.46803834	0.2129716	0.6444482	1
FBgn003854	CG14321	-0.7136455	7.40419953	0.21293822	0.64447414	1
FBgn000372	Tm1	0.92230568	6.7072219	0.21289559	0.64450728	1
FBgn002317	rnh1	0.96671493	6.77112178	0.21230493	0.64496679	1
FBgn002418	san	0.79512328	7.03471143	0.21182386	0.64534162	1
FBgn003911	CG10214	-2.5352659	5.35418586	0.2117945	0.64536452	1
FBgn004057	CG15922	1.23758323	5.63363229	0.21094647	0.64602658	1
FBgn003505	CG3894	1.03752806	6.3948023	0.21064237	0.64626439	1
FBgn002481	Clc	0.50682466	9.23746407	0.21052585	0.64635556	1
FBgn003880	Srp14	1.18439459	5.85233375	0.21049202	0.64638204	1
FBgn005112	CG31126	0.92280329	6.66524371	0.21031189	0.64652306	1
FBgn028594	RpS10b	0.58643696	8.09093527	0.21022359	0.64659222	1
FBgn025081	UQCR-C2	-0.4957398	9.97835919	0.21017072	0.64663363	1
FBgn003465	eIF3k	0.78477207	7.13740063	0.20984081	0.6468922	1
FBgn001551	mbc	3.14712939	3.87933941	0.20946781	0.64718485	1
FBgn002499	CG2685	-3.7252599	5.24024755	0.20943818	0.64720811	1
FBgn003996	Rab21	-0.8970241	7.01706588	0.20890102	0.64763014	1
FBgn001442	CG11899	1.27608963	6.22975123	0.20889917	0.6476316	1
FBgn008520	CG34172	-3.2990395	3.38589112	0.2081368	0.6482317	1
FBgn026782	Fatp1	0.77412416	7.37381416	0.20810351	0.64825793	1
FBgn002757	CG5009	-3.6770192	5.79886673	0.2080249	0.64831989	1
FBgn026139	Prosalpha3	-0.7204187	7.63590338	0.20801602	0.64832689	1
FBgn005120	CG31207	1.51466311	3.51177436	0.20739071	0.64882027	1
FBgn002563	Hmt4-20	-1.8420376	4.01929367	0.20727952	0.6489081	1
FBgn003348	CG1371	-4.8232181	6.47424245	0.20671165	0.64935708	1
FBgn004221	CG18731	-4.3473844	4.22003171	0.20665658	0.64940066	1
FBgn000112	Got1	0.67281491	8.76594054	0.20633953	0.6496517	1
FBgn003797	CG18547	1.34621935	5.91535099	0.20633254	0.64965724	1
FBgn003887	Idi	0.80027066	7.12660821	0.20579227	0.65008556	1
FBgn026577	PDZ-GEF	-2.2074847	3.75096489	0.20567594	0.65017788	1
FBgn026737	sau	0.67873519	7.50372338	0.20566157	0.65018928	1
FBgn000022	brn	-2.2080428	3.46819143	0.2056376	0.65020831	1
FBgn025082	meigo	-1.3997413	6.03771826	0.2054944	0.650322	1
FBgn026397	Tim17b	-0.5850536	8.71942775	0.20514839	0.65059692	1
FBgn003971	RpS8	0.49080254	9.53680143	0.20471409	0.65094238	1
FBgn003298	RpL21	0.52912264	8.61866239	0.20433364	0.65124536	1
FBgn003603	CG6767	0.71229285	7.48420001	0.2037857	0.65168233	1
FBgn005220	CG32202	-4.5502034	4.95702902	0.203214	0.65213901	1
FBgn003516	CG13887	0.6444941	7.98896928	0.20311789	0.65221586	1
FBgn003080	Ubr1	1.54591609	3.95303286	0.20292421	0.65237079	1
FBgn000025	Cam	-0.4959668	9.95824006	0.20265476	0.65258648	1

FBgn005228	CG32280	-4.1012583	5.69304027	0.20205039	0.65307089	1
FBgn002867	Vha100-1	-1.1174658	7.67063027	0.20204482	0.65307536	1
FBgn025086	CG42239	1.14139892	6.06385186	0.20184745	0.65323375	1
FBgn004109	tai	1.5997315	5.28879019	0.2017039	0.65334901	1
FBgn005042	CG30423	0.59466281	7.84071579	0.20115481	0.65379033	1
FBgn003175	CG9044	1.22362443	5.6321797	0.20114251	0.65380023	1
FBgn003317	p47	0.92453672	6.58364653	0.20100702	0.65390924	1
FBgn002298	qkr58E-2	-1.3620538	6.36964493	0.20095687	0.65394959	1
FBgn005135	Unc-115a	3.70418849	3.02234337	0.20073375	0.65412924	1
FBgn003301	CG10465	0.63757219	7.68789342	0.2003853	0.65441003	1
FBgn026671	EloC	0.50853141	8.92406422	0.20008599	0.65465144	1
FBgn026094	Atg1	-1.1307269	6.38562385	0.20001637	0.65470764	1
FBgn002883	Dak1	0.49254833	9.10528591	0.19967914	0.65497996	1
FBgn003402	CG8187	-4.7635733	5.93560952	0.19944949	0.65516557	1
FBgn004089	ksh	1.23590954	5.4199609	0.1993269	0.6552647	1
FBgn002572	unc-13	-1.0536481	8.12130282	0.19929217	0.6552928	1
FBgn003362	CG12384	0.51404043	8.72434832	0.19870394	0.65576903	1
FBgn005227	CG32278	-0.6367642	7.61372264	0.19869385	0.6557772	1
FBgn001203	Ance	-1.7968297	3.75161021	0.19859342	0.6558586	1
FBgn008668	vvl	-3.1135747	3.36823654	0.19857273	0.65587538	1
FBgn005236	CG32369	-2.0301057	3.59023319	0.19857108	0.65587671	1
FBgn004218	CG18870	-2.7980385	6.28515027	0.19816198	0.65620855	1
FBgn000355	su(f)	-4.7881596	6.2012316	0.19808778	0.65626879	1
FBgn000131	klar	4.59991647	5.42418843	0.19782355	0.65648338	1
FBgn003335	CG8237	-1.4775106	6.55903919	0.19744606	0.65679026	1
FBgn003288	CG9331	0.90797441	7.67260849	0.19735015	0.65686828	1
FBgn026216	magu	1.73120986	5.12631403	0.19734968	0.65686867	1
FBgn026641	lncRNA:CR45	-1.3672486	5.01957892	0.19712848	0.65704871	1
FBgn001755	Rga	1.37841557	6.37437829	0.19704734	0.65711478	1
FBgn003872	CG6184	-2.1256304	6.95393336	0.19699593	0.65715665	1
FBgn001034	Arf79F	0.5018393	9.07228897	0.19686316	0.65726482	1
FBgn001418	Hel25E	0.68591867	8.0889212	0.19667762	0.65741605	1
FBgn003365	S2P	-4.3845316	5.44725512	0.19658025	0.65749545	1
FBgn003532	CG13807	0.84668632	6.7790611	0.19653164	0.6575351	1
FBgn003631	Sf3a2	1.10936951	5.98182492	0.19637786	0.65766056	1
FBgn026063	Diap1	0.52410099	8.7901812	0.19617712	0.65782442	1
FBgn000006	Ald1	-0.4428277	11.1227626	0.19599135	0.65797616	1
FBgn003927	CG11920	-3.9742774	5.4488137	0.19533051	0.65851662	1
FBgn005035	Mal-A5	-3.8956329	7.43998043	0.19529334	0.65854706	1
FBgn003614	CG7607	-0.5158467	8.55301569	0.19491921	0.65885355	1
FBgn001697	l(2)k10201	-4.0594814	4.74039163	0.19491598	0.65885621	1
FBgn008548	Pdxk	1.10013603	7.79696777	0.19488936	0.65887802	1
FBgn025915	Clbn	-1.0652886	7.26046446	0.19456993	0.65914002	1
FBgn001128	RpS4	0.50514912	8.8058682	0.19425789	0.65939619	1
FBgn026074	Tango9	-0.7150555	7.21194993	0.1936798	0.65987144	1
FBgn002640	Mpcp2	-0.4841395	9.27713807	0.19327514	0.66020462	1

FBgn002001	Ppox	1.21234459	6.28018389	0.19327299	0.66020638	1
FBgn026399	cpo	-1.1964085	6.47176247	0.19300712	0.66042552	1
FBgn026529	pAbp	0.49781859	11.0524876	0.19293658	0.66048369	1
FBgn002854	TM9SF4	-0.6949479	7.59973268	0.1927382	0.66064734	1
FBgn003619	CG11658	-5.0317207	5.65291087	0.19270415	0.66067545	1
FBgn000057	Eno	-0.4506487	10.6468103	0.19262114	0.66074396	1
FBgn003921	CG13630	-0.8709456	6.77664923	0.1923778	0.66094492	1
FBgn005274	CR32745	-1.5409039	3.01425855	0.19197884	0.66127472	1
FBgn003067	CG9281	-1.5253439	3.98238961	0.19180536	0.66141826	1
FBgn000443	Cyp1	0.43141412	11.0754047	0.1916994	0.66150597	1
FBgn003692	Tom20	-0.4810101	9.35003415	0.19120833	0.66191283	1
FBgn002569	santa-maria	3.26584914	4.3733386	0.19120041	0.6619194	1
FBgn000212	l(2)gl	-4.0164023	3.27802698	0.19112662	0.6619806	1
FBgn003200	CG8360	0.71986092	7.14080275	0.19097915	0.66210293	1
FBgn003308	CG17266	1.54459233	4.41999954	0.19080237	0.66224965	1
FBgn003666	CG9705	-1.4388931	5.151072	0.19037923	0.66260118	1
FBgn005355	Nipped-A	1.43489122	6.45210293	0.19019915	0.66275092	1
FBgn002783	Dp1	-1.7440193	3.77251839	0.18990093	0.66299909	1
FBgn003472	Liprin-gamm	-1.4332255	5.48700581	0.18984805	0.66304311	1
FBgn005311	CG33116	-4.7320328	5.27486115	0.18974916	0.66312547	1
FBgn003051	CG11151	0.83574267	6.81582694	0.18954697	0.66329394	1
FBgn003429	CG5757	-1.3010728	4.94865536	0.18944816	0.66337631	1
FBgn005136	CG31360	0.88609087	6.61146893	0.18942511	0.66339553	1
FBgn003118	Usp2	-1.3456455	5.89977562	0.18913408	0.6636383	1
FBgn026310	BtbVII	-3.1255185	3.77661717	0.18903485	0.66372112	1
FBgn008644	AsnRS	0.81966127	6.8281911	0.18876247	0.6639486	1
FBgn026273	norpA	-0.8621269	7.01521334	0.18844457	0.66421435	1
FBgn003896	mats	-1.8863737	3.47702035	0.18826286	0.66436638	1
FBgn026066	Mp	4.12816557	5.48143333	0.18807896	0.66452032	1
FBgn003651	AIMP2	-1.7712493	5.38446994	0.188037	0.66455545	1
FBgn003736	CG2182	0.92637137	6.78477326	0.18803587	0.6645564	1
FBgn003788	CG14701	-3.7249903	4.03626236	0.18797601	0.66460653	1
FBgn000325	rl	-1.5666496	5.74202415	0.18760995	0.66491332	1
FBgn002686	Thd1	-4.7267175	4.74704145	0.18754054	0.66497154	1
FBgn026002	CG42495	-1.1851334	5.7644746	0.18753973	0.66497222	1
FBgn000025	car	-3.5298898	5.44727253	0.18719366	0.66526265	1
FBgn003137	Wdr62	3.88205821	3.73118525	0.18717643	0.66527712	1
FBgn003205	PIG-U	0.79996386	7.02264607	0.18675066	0.66563489	1
FBgn006241	Ctr1A	1.05277638	6.18549013	0.18672416	0.66565717	1
FBgn000113	grau	-2.1891667	7.11280796	0.18606626	0.666211	1
FBgn002475	Flo1	-0.6485367	7.57023742	0.18588026	0.66636779	1
FBgn000333	Scm	1.41811779	3.85794122	0.18516126	0.66697475	1
FBgn026212	l(2)41Ab	-4.5601681	4.23447164	0.18514813	0.66698585	1
FBgn005298	CG32982	-1.2883189	5.12628697	0.18509929	0.66702713	1
FBgn002568	CG3164	1.6041008	6.25484959	0.18473166	0.66733807	1
FBgn004030	MTF-1	-1.8515738	4.61367718	0.18467053	0.66738981	1

FBgn003866	Mpc1	-0.6569893	7.6084305	0.18458976	0.66745819	1
FBgn003525	CG7971	0.5115516	8.42503342	0.18458759	0.66746002	1
FBgn028595	mRpS34	-0.926006	6.54225708	0.18415094	0.66783	1
FBgn026074	Chchd2	0.54363075	8.25592152	0.1838415	0.66809249	1
FBgn003185	Aatf	-2.5668111	6.92885107	0.18370819	0.66820566	1
FBgn003920	CG6607	-1.2241239	6.28080129	0.18367427	0.66823446	1
FBgn002062	Pi3K21B	-2.6400673	4.97501821	0.18363794	0.66826532	1
FBgn003325	CG14762	-1.2778359	6.43382143	0.18362117	0.66827956	1
FBgn003758	DppIII	-0.8376214	7.38287014	0.18329378	0.66855777	1
FBgn002854	CG9008	1.22525561	6.04902127	0.182442	0.66928297	1
FBgn002677	Rad23	-0.629385	8.49392426	0.18238609	0.66933064	1
FBgn005009	CG30094	-4.0764089	5.19683615	0.18221362	0.66947775	1
FBgn004487	Gos28	1.11812351	5.8463987	0.18207615	0.66959507	1
FBgn000464	N	-2.8865902	4.34878265	0.18174371	0.66987899	1
FBgn003847	kuk	0.71943576	7.13922061	0.18161418	0.6699897	1
FBgn003617	CG7394	-1.0549419	5.99011051	0.18155233	0.67004257	1
FBgn000355	Su(dx)	2.60468016	7.15713356	0.18123114	0.67031735	1
FBgn005018	AIMP3	-0.781777	6.88735313	0.18060116	0.67085713	1
FBgn001398	MAPk-Ak2	-0.7608571	7.20936875	0.18051039	0.67093499	1
FBgn003270	CG10341	-4.128405	5.91231041	0.18041034	0.67102084	1
FBgn028365	Tlk	-1.3410477	5.89702361	0.18034854	0.67107388	1
FBgn005264	CG32641	1.63413795	4.17977783	0.18024851	0.67115976	1
FBgn003772	p23	0.54453066	8.0069909	0.17997939	0.67139095	1
FBgn003910	CG10365	-1.2864679	4.69361948	0.17933293	0.67194714	1
FBgn002869	Rpn9	-0.7232997	7.08325444	0.17918662	0.67207318	1
FBgn003882	CG4000	2.38197307	8.71716686	0.1789297	0.67229466	1
FBgn002563	SkpA	0.45006964	9.77451585	0.17889866	0.67232143	1
FBgn001176	Fdx1	1.19641335	5.35642575	0.17882858	0.67238188	1
FBgn003320	CG2064	-4.0453718	6.04274791	0.17864743	0.6725382	1
FBgn026104	Vps45	1.01844978	6.40979478	0.17844986	0.67270881	1
FBgn003760	CG11753	-4.3407182	5.36204019	0.17821243	0.67291397	1
FBgn004050	Alk	-1.0941488	6.48332563	0.17819898	0.6729256	1
FBgn000485	Bx42	-0.8901256	6.77931058	0.17812917	0.67298596	1
FBgn002836	kirre	-3.0311149	4.25805855	0.17810705	0.67300509	1
FBgn000037	crc	0.59618534	7.66337431	0.17800363	0.67309453	1
FBgn005315	CG33155	1.37687702	4.40898313	0.17796404	0.67312878	1
FBgn002864	beat-Ic	1.4569589	6.07359664	0.17770988	0.67334877	1
FBgn003884	hdly	0.98146708	6.20467934	0.17762723	0.67342034	1
FBgn003750	CG1142	-4.4422068	6.26895122	0.17727356	0.67372684	1
FBgn003224	RfC3	-1.4026813	4.93453452	0.17721923	0.67377397	1
FBgn003992	CG11076	-0.6179916	7.58338239	0.17703625	0.67393271	1
FBgn003874	CG4538	-3.6937015	5.15015059	0.17659194	0.67431858	1
FBgn000044	Dhod	-1.1192489	5.71459366	0.17645896	0.67443418	1
FBgn003791	wkd	-0.7094385	7.77314028	0.17599902	0.67483441	1
FBgn003772	CG8319	1.44833278	4.53669059	0.17548923	0.67527873	1
FBgn026109	Sytbeta	1.09990076	5.74669183	0.17494411	0.67575468	1

FBgn002321	EloB	-0.9399228	6.52684485	0.17489673	0.6757961	1
FBgn004388	mask	-0.8824348	6.64358679	0.17483972	0.67584594	1
FBgn003913	CG13603	-0.5272812	8.06306031	0.17474323	0.6759303	1
FBgn003052	Pdcd4	-0.4608981	9.2389758	0.17473702	0.67593573	1
FBgn025090	Pgk	-0.4660556	9.06067011	0.17472647	0.67594496	1
FBgn003109	CG17065	-0.594544	7.70100396	0.17450775	0.67613631	1
FBgn005315	CG33158	-4.4061386	4.6078152	0.17425917	0.67635397	1
FBgn003612	Irbp18	-2.1396193	4.27329622	0.17421654	0.6763913	1
FBgn002754	Nulp1	0.70669461	7.17304704	0.17420545	0.67640102	1
FBgn002705	CSN5	-1.1223646	5.61628628	0.17345262	0.6770614	1
FBgn004300	Chrac-14	-3.6033899	5.17053672	0.17326362	0.67722747	1
FBgn005314	CG33143	-2.5227421	3.53250114	0.17323621	0.67725156	1
FBgn026408	sli	-2.1385263	2.41590948	0.17323004	0.67725698	1
FBgn026399	CG43736	1.17687387	5.53407639	0.17301531	0.67744579	1
FBgn000394	RpS27A	0.50924611	8.28805546	0.17292521	0.67752506	1
FBgn000010	RpLP0	0.4667003	9.80634665	0.17259172	0.67781866	1
FBgn003896	CG13850	0.78765184	6.77977398	0.17238313	0.67800247	1
FBgn003973	CG7903	-1.7114485	5.984688	0.17209383	0.67825762	1
FBgn003081	wcy	-4.2399204	4.44285495	0.17208218	0.6782679	1
FBgn003712	CG11247	-4.5405035	5.20758034	0.17196603	0.67837041	1
FBgn002989	CG14441	-2.2198385	3.63482822	0.1719658	0.67837062	1
FBgn001038	Acbp2	1.08938558	6.42298178	0.17191346	0.67841683	1
FBgn002979	CG15772	-4.0376538	5.40274661	0.17076939	0.67942893	1
FBgn003709	Cdk12	-2.3696172	3.53771106	0.17073142	0.67946259	1
FBgn003050	NFAT	-3.1881146	4.84630039	0.17054338	0.67962934	1
FBgn025083	CG12547	0.51247666	8.16823803	0.17050405	0.67966423	1
FBgn003354	CG7220	-0.8242573	6.64263225	0.16888045	0.68110871	1
FBgn003203	Rcd4	1.05802168	5.93484562	0.16861445	0.68134613	1
FBgn003375	Ak6	1.33361827	5.38778554	0.16846568	0.68147902	1
FBgn002559	Gk1	-0.8220652	7.07361558	0.16811868	0.68178923	1
FBgn005270	CG32706	1.42849852	4.12964509	0.16808405	0.68182022	1
FBgn005107	CG31075	1.27939788	5.39115601	0.16793008	0.681958	1
FBgn003731	CG11999	-0.7314543	6.92775629	0.16775457	0.68211515	1
FBgn004023	Best1	-4.5155097	6.03170057	0.16771614	0.68214957	1
FBgn003391	CG8485	-4.7734478	6.05640428	0.16754623	0.68230183	1
FBgn003541	CG14966	-3.76896	4.26135727	0.16730524	0.68251792	1
FBgn003634	CG11279	1.25307892	5.49003316	0.16717112	0.68263826	1
FBgn003295	CG2201	-4.3855985	4.96937249	0.16694024	0.68284555	1
FBgn003367	CG8321	0.98211645	6.1441381	0.16687516	0.68290402	1
FBgn003955	mRpS22	0.78726385	6.86897877	0.1668641	0.68291396	1
FBgn026144	CG3638	-1.2284522	4.99130936	0.16642251	0.68331101	1
FBgn001402	RpL7A	0.49512629	8.9553347	0.16627107	0.68344732	1
FBgn025998	kuz	-2.845764	4.9333571	0.16570892	0.68395393	1
FBgn001164	lark	-0.4999614	8.30684413	0.16561756	0.68403636	1
FBgn000162	dlg1	-1.7602409	6.55791392	0.16560142	0.68405093	1
FBgn003203	CG13384	-0.6122494	7.5149756	0.16544334	0.68419362	1

FBgn026164	salm	-1.9915508	3.29049191	0.16523251	0.68438406	1
FBgn003654	GXIVsPLA2	-1.1160259	5.46373793	0.16476277	0.68480887	1
FBgn003200	Snx6	-1.306228	7.47327303	0.16449338	0.68505281	1
FBgn003405	CG8314	-2.3756835	5.31158893	0.16448739	0.68505824	1
FBgn003387	Echs1	-1.1302026	6.044575	0.16425059	0.68527287	1
FBgn001523	Hr78	-1.1810488	5.33038897	0.16403046	0.68547256	1
FBgn000025	CkIIbeta	0.48560334	8.3907755	0.16400431	0.68549629	1
FBgn003924	CG3744	1.36904818	4.29378098	0.16359192	0.68587083	1
FBgn026656	CG42259	-1.3312843	2.43009944	0.16320751	0.68622045	1
FBgn003427	Vps50	2.98501825	5.25393934	0.16285186	0.68654434	1
FBgn002023	ATPCL	0.65258632	8.47194033	0.16232269	0.68702701	1
FBgn002024	stck	-0.8919717	6.75315996	0.1622877	0.68705897	1
FBgn003270	Jwa	0.6123341	7.53742398	0.16202813	0.68729609	1
FBgn003989	ABCD	1.65986908	6.50295673	0.16170965	0.68758734	1
FBgn000313	Ppn	4.29821549	8.80465429	0.1613889	0.687881	1
FBgn003221	CG4968	0.6809682	7.22236296	0.16135598	0.68791116	1
FBgn003650	CG7841	-0.9259923	6.51853189	0.16079242	0.688428	1
FBgn002423	foi	-4.0944421	5.35777354	0.1604998	0.68869678	1
FBgn005354	Rim	-0.8851536	6.57781852	0.15995541	0.68919758	1
FBgn026385	Dad1	0.82749976	6.58795815	0.15974847	0.6893882	1
FBgn026658	ND-30	-0.4492351	8.99430688	0.1594476	0.68966561	1
FBgn001580	HDAC1	1.01649936	6.21122087	0.15931329	0.68978954	1
FBgn004089	CG17776	0.791355	6.67198823	0.15930206	0.68979991	1
FBgn002833	l(1)G0289	2.9114376	5.0078837	0.15919199	0.68990153	1
FBgn003154	CG17593	1.57585409	6.60383235	0.15916558	0.68992591	1
FBgn003036	Lsm12a	-2.2779821	5.68561115	0.15909578	0.68999038	1
FBgn003177	CG9147	-4.5991176	6.31083698	0.15895767	0.69011798	1
FBgn003168	Pgant5	1.4252614	3.13969808	0.15894657	0.69012824	1
FBgn003289	CG9338	-4.0033864	3.46855184	0.15884424	0.69022283	1
FBgn003865	Octalpha2R	2.25120056	4.60978628	0.15872486	0.69033323	1
FBgn001063	Sec61beta	0.48653707	8.3176785	0.1586847	0.69037038	1
FBgn003265	CG5674	2.81998137	6.36881935	0.15859637	0.69045211	1
FBgn026258	cic	0.87004208	6.42154623	0.15849702	0.69054406	1
FBgn026675	btsz	-2.6194338	3.96585234	0.15823563	0.69078615	1
FBgn003872	CG6195	-0.8280106	6.89161962	0.15804737	0.69096065	1
FBgn002593	Eph	1.45932338	6.17765899	0.1579248	0.69107434	1
FBgn000121	Hsc70-3	0.47545943	8.60580667	0.15775126	0.69123538	1
FBgn003933	CG4553	-4.1757832	5.65858236	0.15747248	0.69149428	1
FBgn026155	CG42668	1.09279079	5.56647138	0.15730339	0.69165146	1
FBgn026179	Trf2	-1.0663904	5.62422865	0.15714904	0.691795	1
FBgn025924	Pka-R1	-1.267664	7.67897969	0.15698653	0.69194624	1
FBgn003972	Capa	-1.0631619	5.91597957	0.15676703	0.69215065	1
FBgn003691	Fibp	0.56610031	7.60532738	0.1562941	0.69259163	1
FBgn008546	ND-MWFE	0.6313723	7.25612845	0.15624192	0.69264033	1
FBgn003892	Cchl	-0.6367006	7.26126751	0.15610153	0.69277141	1
FBgn001693	Dyrk2	-4.4800628	3.83633459	0.15555283	0.69328437	1

FBgn002870	RfC38	1.13315936	5.48051852	0.1553429	0.69348091	1
FBgn001375	Arf51F	0.50052093	8.04230387	0.15507413	0.69373275	1
FBgn005118	LSm3	1.5068797	4.44884973	0.15479085	0.69399845	1
FBgn028346	slmb	-3.5295014	5.54354611	0.15468099	0.69410158	1
FBgn003245	CG5787	1.40260448	5.78318347	0.1541202	0.69462864	1
FBgn000235	l(3)87Df	-0.8259838	6.59323203	0.1540123	0.69473017	1
FBgn003976	CG15535	1.4312387	3.83409131	0.15352172	0.69519234	1
FBgn001028	Uch	-1.9975962	5.832008	0.15349685	0.6952158	1
FBgn026193	SmD1	-2.326422	4.21769885	0.15328865	0.69541221	1
FBgn005100	qless	1.13689513	5.41935503	0.1528847	0.69579374	1
FBgn003621	RpL10Ab	0.42992474	9.39599954	0.15268708	0.69598061	1
FBgn003897	Pfdn5	0.48348563	8.18180911	0.15267632	0.69599078	1
FBgn008538	CG34354	-4.7692319	5.80939715	0.15243267	0.69622137	1
FBgn003067	CG8134	-4.4851009	6.09961395	0.15235193	0.69629783	1
FBgn008670	ncm	-2.2761487	5.19176909	0.15173355	0.69688419	1
FBgn001327	Hsp70Bc	-2.7552685	2.71887431	0.15166676	0.6969476	1
FBgn003284	sNPF	3.71536959	9.73667834	0.15138096	0.69721914	1
FBgn000488	orb	-2.4507342	3.23543003	0.15136927	0.69723025	1
FBgn003881	CG15923	1.99610054	3.35219115	0.15130902	0.69728754	1
FBgn003427	OstDelta	0.68329462	7.35236464	0.15130142	0.69729477	1
FBgn003097	CG7378	-0.6327076	7.34831771	0.15107268	0.69751237	1
FBgn000413	blow	-1.3917698	3.72308615	0.15095671	0.69762276	1
FBgn001581	Spx	-1.222022	5.2648199	0.15030781	0.69824138	1
FBgn005199	CG31999	-1.6838456	4.85672443	0.14970229	0.69882003	1
FBgn002199	Rs1	-1.411135	4.37306388	0.14967094	0.69885002	1
FBgn003343	CG1827	0.95342512	6.30146475	0.14957692	0.69894	1
FBgn002350	D2hgdh	0.81066673	6.64422468	0.14941091	0.69909895	1
FBgn004000	CG17883	-0.5454874	7.86335507	0.14930882	0.69919675	1
FBgn002555	CG4101	-1.3470549	2.76954791	0.14927914	0.69922519	1
FBgn028678	hoip	-3.8962596	4.2629984	0.14923341	0.69926902	1
FBgn003361	CG7741	-2.0478101	6.25517088	0.14913875	0.69935976	1
FBgn003640	CG13484	-2.3023844	4.28999385	0.14828869	0.70017614	1
FBgn000413	boca	0.7710428	6.96732727	0.14795063	0.70050156	1
FBgn000053	eag	1.90608033	5.34248538	0.14794645	0.70050558	1
FBgn002571	Bap55	-0.6409922	7.50700829	0.1478422	0.70060602	1
FBgn002978	Sirt4	1.36677536	3.12117749	0.14751644	0.70092013	1
FBgn003390	ReepB	0.61028196	7.52459322	0.14737258	0.70105897	1
FBgn001054	Aldh-III	-0.7559625	6.69692494	0.14733665	0.70109366	1
FBgn005003	Tret1-1	0.39390473	10.440626	0.14730153	0.70112756	1
FBgn003562	Blimp-1	3.45887029	3.69550834	0.14728343	0.70114504	1
FBgn003074	CG4239	0.6975081	6.88874683	0.14717672	0.70124812	1
FBgn003497	yki	0.80166965	6.56002995	0.14663069	0.70177621	1
FBgn003681	MED11	-4.1372601	4.84606487	0.14588003	0.70250405	1
FBgn003823	Pde6	-0.9975452	5.82380396	0.14579531	0.70258634	1
FBgn001526	Nap1	1.31508136	3.44286605	0.14543952	0.70293218	1
FBgn003762	M1BP	0.65237226	7.10472463	0.14541108	0.70295985	1

FBgn002753	CG7139	-3.8806761	3.35649827	0.14538317	0.702987	1
FBgn004117	Vhl	0.9300831	6.10919898	0.14517852	0.70318618	1
FBgn003457	CG3295	1.06817896	6.23719594	0.14495844	0.70340058	1
FBgn002911	Uba2	1.55440519	6.33468405	0.14456145	0.70378777	1
FBgn003036	Usp7	-1.0124111	5.70571552	0.14437403	0.70397078	1
FBgn001580	ScpX	-0.9512943	6.59418975	0.14422571	0.70411571	1
FBgn000112	Gpdh1	-0.5817827	7.6828062	0.14388149	0.70445238	1
FBgn003438	List	-0.8797472	6.32903758	0.14369445	0.70463551	1
FBgn026260	SmB	0.60913884	7.36973137	0.14334122	0.70498173	1
FBgn005151	CG31510	-2.808423	4.66422456	0.14297513	0.70534106	1
FBgn003251	Ing5	-4.0366285	4.82530063	0.14293227	0.70538317	1
FBgn001375	Bgb	-0.6613263	6.99635007	0.14240058	0.70590608	1
FBgn003822	ATPsynE	0.44303175	8.69138732	0.14208934	0.7062127	1
FBgn025910	futsch	-0.4534481	8.46555546	0.141875	0.70642408	1
FBgn008526	CG34232	-3.2665147	4.72458721	0.14150643	0.70678799	1
FBgn003040	Pits	1.22324228	5.99223101	0.1412885	0.70700342	1
FBgn003350	CAP	-1.254623	4.28832707	0.14073729	0.70754914	1
FBgn026444	CG43867	-1.073309	6.10551844	0.1404366	0.70784736	1
FBgn026323	SP1029	-2.4625703	6.8686764	0.14024747	0.70803512	1
FBgn005226	CG32267	0.68783269	6.97293297	0.14015629	0.70812569	1
FBgn003088	CG6867	1.2859995	4.1367696	0.13994457	0.70833613	1
FBgn003645	CG9425	-1.2881039	4.46932714	0.13921607	0.7090616	1
FBgn026208	Csk	1.85650723	2.70676841	0.13915644	0.70912107	1
FBgn001992	Surf4	0.52196828	7.68070658	0.13864266	0.70963414	1
FBgn002569	ZnT41F	1.14691569	5.49030063	0.13814941	0.71012773	1
FBgn002839	Tob	-0.6384621	7.33793064	0.13814371	0.71013343	1
FBgn003027	CG15201	0.41819264	8.95636917	0.13805027	0.71022705	1
FBgn003496	RpL12	0.49604637	8.18863851	0.13802034	0.71025704	1
FBgn028681	Pdi	-0.5564747	7.50971127	0.13768718	0.71059118	1
FBgn001166	Moe	0.5146881	7.77817562	0.13738536	0.71089428	1
FBgn005103	CG31030	-0.3962084	9.62381762	0.13701144	0.71127031	1
FBgn025971	CG42365	-3.9156675	3.95129599	0.13683165	0.71145132	1
FBgn000045	Dip-C	0.96243297	6.77555212	0.13667082	0.71161336	1
FBgn001376	Chi	-1.1019444	5.23663729	0.13647017	0.71181567	1
FBgn003995	CG17514	-4.4707014	6.35022955	0.1361142	0.71217499	1
FBgn003735	CG12170	-3.6267736	6.61201625	0.13583429	0.71245792	1
FBgn003366	ERp60	0.4121581	9.09211133	0.13564167	0.71265282	1
FBgn004037	AANATL7	0.90071152	6.08668211	0.13560794	0.71268696	1
FBgn003216	CG4598	0.77190252	6.75628379	0.1354684	0.71282826	1
FBgn005259	CG32590	0.64032053	6.9899784	0.13531495	0.71298374	1
FBgn002783	Dgp-1	-0.8354999	6.29428845	0.13526849	0.71303083	1
FBgn003198	RpL36A	0.43320791	8.45906993	0.13524619	0.71305345	1
FBgn003918	Golgin84	0.9160995	6.31018703	0.13475543	0.71355152	1
FBgn003325	CG8712	0.92936872	5.94311309	0.13422515	0.71409086	1
FBgn003425	eIF3c	-1.1258576	5.71612668	0.13414434	0.71417316	1
FBgn003188	Mnn1	-0.6665776	6.88570282	0.13388006	0.71444251	1

FBgn003148	CG17224	0.60445462	7.13348896	0.13381458	0.71450929	1
FBgn003000	alpha-PheRS	-0.5640446	7.39165433	0.13372303	0.71460268	1
FBgn026339	Tet	-2.39363	5.27375837	0.1335557	0.71477349	1
FBgn003187	Fgop2	0.97772127	6.26948429	0.13342636	0.71490559	1
FBgn000317	px	-4.1085858	4.46364269	0.13340306	0.7149294	1
FBgn003071	Slc25A46a	0.81917839	7.21821757	0.1332445	0.71509147	1
FBgn002866	VhaPPA1-1	-0.4327466	9.43375864	0.1329496	0.71539318	1
FBgn003931	CG10513	-0.7550365	6.88906961	0.13284073	0.71550467	1
FBgn000308	phr	-4.4592551	5.85861579	0.13282534	0.71552043	1
FBgn002623	gus	-0.8192286	7.54014134	0.1328144	0.71553163	1
FBgn003310	CG3420	0.72491506	6.66182797	0.13214645	0.71621682	1
FBgn002223	lolal	0.49913348	7.82807416	0.13207224	0.71629308	1
FBgn003784	CG6567	-3.906933	4.94905758	0.1320459	0.71632014	1
FBgn002758	PCB	1.2523817	6.13197602	0.13194024	0.71642876	1
FBgn025922	CG42327	0.63402796	6.98065853	0.13144201	0.71694157	1
FBgn003075	rngo	0.95531731	6.97443429	0.13141345	0.716971	1
FBgn002986	CG15894	-4.160226	4.63761905	0.13139574	0.71698925	1
FBgn000437	Ptp10D	-3.7645075	3.52635186	0.13125394	0.71713544	1
FBgn003697	CG5618	4.31680469	6.97391946	0.13121737	0.71717315	1
FBgn006996	CG40498	0.45920435	8.17456378	0.13121553	0.71717505	1
FBgn003222	Sps2	0.97722066	5.72345376	0.13067297	0.7177353	1
FBgn003143	Arpc5	0.74541878	6.67347193	0.13009465	0.71833392	1
FBgn003397	CG10205	1.31564776	3.40527671	0.13008701	0.71834185	1
FBgn002734	Gfat1	1.19607378	4.88769126	0.12993563	0.71849879	1
FBgn003773	trbd	-1.0447561	5.18551159	0.1290898	0.71937764	1
FBgn004053	Sf3b5	0.7153804	6.63730424	0.12894612	0.71952725	1
FBgn003177	Pfdn1	0.92398456	6.05877478	0.12855847	0.71993138	1
FBgn000374	tra	-1.3551836	4.58240199	0.12806627	0.72044548	1
FBgn001757	RpL14	0.39909624	9.15570699	0.12794427	0.72057308	1
FBgn004158	olf186-F	-2.4023417	5.55387569	0.12794178	0.72057569	1
FBgn001017	RpA-70	4.26201046	7.0011178	0.12771438	0.72081372	1
FBgn004002	Set1	-4.7593583	4.88671794	0.12733398	0.72121244	1
FBgn003273	CG15168	-4.1765138	4.86527514	0.12727514	0.72127417	1
FBgn003376	CG8768	-0.8075466	6.30550104	0.12726284	0.72128707	1
FBgn005001	CG30010	-1.5921138	4.91667928	0.1270072	0.72155549	1
FBgn002608	CG14818	0.52425388	7.55091873	0.12696961	0.72159498	1
FBgn002538	rush	-2.8023855	5.56704592	0.12688498	0.72168392	1
FBgn001754	RpS3A	0.38359719	10.0680561	0.12681749	0.72175487	1
FBgn028595	cv-c	1.91462846	2.72515615	0.12663091	0.72195113	1
FBgn003623	Pop2	-0.5768102	7.42008052	0.12641662	0.72217673	1
FBgn003048	Tim9a	1.06334711	5.22390766	0.12618461	0.72242124	1
FBgn004077	COX7C	0.40866777	8.95909791	0.12577567	0.72285282	1
FBgn026040	mRpS23	0.93663173	5.89277327	0.12555724	0.72308367	1
FBgn003887	CG3301	1.52235267	6.94583482	0.12554591	0.72309565	1
FBgn002968	Vap33	-0.4201957	8.685695	0.12544904	0.72319811	1
FBgn003770	CG8176	4.53941206	4.7957774	0.12520325	0.72345827	1

FBgn005011	GEFmeso	-1.2885054	4.76258829	0.12509034	0.72357787	1
FBgn003838	CG5516	-3.7754971	5.53057857	0.12500681	0.72366639	1
FBgn003748	CG10055	1.28885074	3.22958061	0.12492097	0.7237574	1
FBgn004046	Dlip3	0.70364021	6.69519361	0.12484942	0.72383328	1
FBgn003656	CG5895	0.49795201	7.65181663	0.12465988	0.72403442	1
FBgn026642	AstA-R1	-4.2267815	5.87389889	0.12464157	0.72405386	1
FBgn003419	Gbp1	-1.1442923	2.56135616	0.12461148	0.72408581	1
FBgn003171	Vps52	-2.7363322	4.98727752	0.12425891	0.72446049	1
FBgn004020	krz	-1.2835703	6.1700099	0.12420126	0.72452181	1
FBgn002868	Rpt4	0.47417796	8.09898061	0.12409415	0.72463579	1
FBgn000450	GlyP	0.47339324	7.80209277	0.12399765	0.72473852	1
FBgn025074	Prosbeta7	0.64853099	7.39685814	0.12378341	0.72496676	1
FBgn001400	Rab2	-0.4154203	8.65219056	0.12373595	0.72501735	1
FBgn003829	Mf	0.53450063	7.56562591	0.12367246	0.72508505	1
FBgn003405	CG8320	-3.0865438	5.68058512	0.12347697	0.72529361	1
FBgn000217	CG5504	-0.4378905	8.29518251	0.12342166	0.72535265	1
FBgn003817	Adgf-C	1.05810946	4.56729943	0.12340138	0.7253743	1
FBgn000411	Tm2	0.88594425	5.96723628	0.12336037	0.72541809	1
FBgn000297	numb	-2.1960197	3.67219646	0.12320053	0.72558885	1
FBgn002270	Adk1	-0.5973075	7.07070242	0.12292286	0.72588578	1
FBgn003009	Zpr1	0.75053947	6.60250837	0.12285732	0.72595592	1
FBgn003150	ND-B14.5B	0.42739793	8.32959935	0.12259747	0.72623422	1
FBgn003803	Cyp9f2	0.5919018	7.34463575	0.12224933	0.7266076	1
FBgn008544	spri	1.15619101	4.22834683	0.12200344	0.72687168	1
FBgn002759	MED15	1.25891354	6.02932376	0.12194898	0.7269302	1
FBgn000252	pho	0.48709424	7.80452723	0.12159351	0.72731259	1
FBgn003324	Nup44A	0.95417318	5.82438105	0.12132224	0.72760482	1
FBgn001171	Snr1	0.86148985	6.01825933	0.12118182	0.72775623	1
FBgn003067	CG8206	-1.2038093	5.36975234	0.1211244	0.72781817	1
FBgn003568	fmt	-0.887257	6.02505526	0.12109839	0.72784624	1
FBgn002222	ubl	0.89455875	5.82550802	0.12105232	0.72789595	1
FBgn001157	Cdc37	0.56988412	7.14693697	0.12092399	0.7280345	1
FBgn005241	vito	0.84538117	6.05780613	0.1208361	0.72812944	1
FBgn026124	inaE	1.05683863	4.84653673	0.12074397	0.72822899	1
FBgn001571	Cyp6a17	-1.2437992	5.32279122	0.12026754	0.72874451	1
FBgn003177	ND-51	-0.3713809	9.85819204	0.12023861	0.72877584	1
FBgn003537	mRpS35	0.69992294	6.71228713	0.11999338	0.72904167	1
FBgn001175	PhKgamma	-0.5314654	7.61433714	0.11959246	0.72947692	1
FBgn008533	CG34310	-2.503878	3.34206992	0.11932916	0.72976321	1
FBgn002984	Nep1	-4.6929478	5.23210791	0.11907987	0.73003459	1
FBgn003067	CG12379	1.32151486	3.10617508	0.11907549	0.73003937	1
FBgn002498	ssx	-0.9467846	5.46134441	0.11906383	0.73005207	1
FBgn005279	DIP-alpha	-1.1942859	3.38041601	0.11906377	0.73005213	1
FBgn003584	ERR	-2.455084	3.17024341	0.11885907	0.73027523	1
FBgn002317	Prosbeta2	0.61043977	6.94901084	0.11850165	0.73066528	1
FBgn003693	CG14184	-2.6423735	5.8158839	0.11849158	0.73067629	1

FBgn000041	Sap-r	0.50487028	8.80213908	0.11838588	0.73079177	1
FBgn001758	Lk6	0.41482579	9.08657578	0.11829151	0.73089493	1
FBgn000011	Arl1	-3.9256471	4.66426039	0.11813087	0.73107062	1
FBgn002436	CG12773	-3.9737456	4.14402567	0.11792917	0.73129142	1
FBgn003666	TSG101	0.43736559	8.17206726	0.11788995	0.73133438	1
FBgn002545	Bub3	-1.2458449	3.72619636	0.11786356	0.73136329	1
FBgn003177	CG13994	0.92475869	5.92303122	0.11768442	0.73155962	1
FBgn026612	ghi	0.78908709	6.24647475	0.11756789	0.73168742	1
FBgn003575	unc-13-4A	-1.3653263	6.23530308	0.11721774	0.73207189	1
FBgn002778	NP15.6	-0.465138	7.94246513	0.11696442	0.73235043	1
FBgn003346	Pdrg1	1.06026337	4.82921081	0.11686621	0.73245851	1
FBgn001612	ATPsynD	0.33756911	10.9665444	0.11638626	0.73298742	1
FBgn003068	CG8944	1.08592443	3.75694228	0.11622213	0.73316857	1
FBgn003977	CG15537	-3.4194492	6.91101674	0.1162208	0.73317004	1
FBgn026407	Flo2	0.65057348	7.11980375	0.1158924	0.73353293	1
FBgn003906	CG4467	-1.0609361	5.88377379	0.11564767	0.73380374	1
FBgn003344	CG1663	-1.0924904	5.10024829	0.11550452	0.73396229	1
FBgn003310	CG15237	0.73056613	6.60701349	0.11528212	0.73420884	1
FBgn001503	cype	0.36292441	10.0680483	0.11468224	0.73487517	1
FBgn004023	bc10	4.45825172	4.08482085	0.11433473	0.73526207	1
FBgn001005	Jheh1	-1.0845389	5.10873154	0.11410752	0.73551538	1
FBgn003662	Agpat3	0.69909006	6.5935694	0.11407743	0.73554895	1
FBgn003771	CG16790	2.80612879	3.31001101	0.11392821	0.7357155	1
FBgn000332	sca	-4.5157423	3.94815227	0.11383158	0.73582341	1
FBgn001129	Taf12	-0.443453	7.94940932	0.11374397	0.73592129	1
FBgn003145	Bacc	0.3581099	9.95733752	0.11361349	0.73606714	1
FBgn026111	BHD	-2.1836664	4.36546698	0.11321196	0.73651658	1
FBgn004060	CG13631	0.60366078	7.30618862	0.11312072	0.73661883	1
FBgn005185	CG31855	0.57299786	7.0701703	0.11288573	0.73688239	1
FBgn005009	CG30096	1.02171537	5.35180013	0.11283958	0.73693419	1
FBgn003629	CG10646	0.77696905	6.19495757	0.11233576	0.73750042	1
FBgn003598	Cpsf5	0.75607582	6.29053532	0.11225386	0.73759259	1
FBgn004046	Dlip2	0.88308201	5.77174875	0.11217986	0.73767591	1
FBgn003718	CG11241	1.02770503	5.48083557	0.11191723	0.73797187	1
FBgn026043	Pp2A-29B	0.37489681	8.95807702	0.11183471	0.73806494	1
FBgn028343	CG46280	-0.9632756	5.586923	0.11136901	0.73859089	1
FBgn005800	ND-AGGG	0.66489557	6.65468837	0.11124901	0.73872661	1
FBgn003360	CG9067	0.90155426	5.60584757	0.11104575	0.73895669	1
FBgn025921	cno	-1.0877582	4.65099142	0.1107975	0.73923802	1
FBgn004034	CG3704	-0.5609353	7.08743366	0.11065498	0.73939968	1
FBgn008312	Uhg4	-0.8246158	5.96523642	0.11037484	0.73971779	1
FBgn003485	eIF2Bdelta	-4.018572	5.72807177	0.11032709	0.73977206	1
FBgn026350	lncRNA:CR43	-3.869789	4.43319179	0.11027291	0.73983364	1
FBgn003189	CG4502	0.68131245	6.58449021	0.11006259	0.74007289	1
FBgn003465	LBR	-1.339203	4.51101321	0.10988824	0.74027141	1
FBgn001159	fng	4.17191794	3.50406645	0.10984384	0.740322	1

FBgn001037[illegible]	Akt1	2.04501143	4.3520594	0.10952116	0.74068995	1
FBgn000198[illegible]	ND-B17	-0.40195	8.34664347	0.10924439	0.74100603	1
FBgn001578	P5cr	0.48309114	7.57836288	0.10852761	0.7418267	1
FBgn003777[illegible]	mtTFB2	4.36456294	6.53603774	0.10838292	0.74199272	1
FBgn002752[illegible]	CG3909	0.85094694	6.11255869	0.10786085	0.74259279	1
FBgn003286[illegible]	CG2493	-1.0152127	7.55276364	0.10778513	0.74267996	1
FBgn001962[illegible]	COX5A	-0.3400591	10.3419044	0.10734354	0.74318898	1
FBgn000047[illegible]	Cyp6a2	4.41835403	4.2656385	0.10729267	0.7432477	1
FBgn003988[illegible]	Arl4	-0.4677293	8.07514447	0.10724643	0.74330108	1
FBgn000491[illegible]	TfIIB	-0.491471	7.46004832	0.10699124	0.74359591	1
FBgn003411[illegible]	CG7997	0.88098666	5.63044245	0.1069661	0.74362498	1
FBgn003756[illegible]	tex	-3.2882062	5.20735205	0.1065913	0.74405878	1
FBgn003810[illegible]	CG7488	0.74591225	6.25035178	0.10649809	0.74416678	1
FBgn003061[illegible]	RpL37a	0.36462061	9.43815956	0.10624633	0.74445879	1
FBgn003381[illegible]	CG4646	0.65967355	6.69529524	0.10623661	0.74447008	1
FBgn003149[illegible]	Sf3b2	-3.5025439	4.73294713	0.10615744	0.74456198	1
FBgn005109[illegible]	CG31098	0.8828505	6.0928576	0.10605954	0.74467568	1
FBgn003130[illegible]	MFS3	-4.0232917	5.93685989	0.10596478	0.7447858	1
FBgn002036[illegible]	Rpt6	0.42479557	8.36799364	0.10569295	0.74510197	1
FBgn000343[illegible]	sm	-0.7856105	7.45661633	0.10549695	0.74533021	1
FBgn028353[illegible]	Vha13	0.34281344	10.0958677	0.10531819	0.7455386	1
FBgn000277[illegible]	mnd	-0.9679494	5.58497394	0.10527722	0.74558638	1
FBgn001022[illegible]	HmgZ	0.47841406	8.06239187	0.10505516	0.74584556	1
FBgn002978[illegible]	RpL35	0.37751987	8.72498447	0.10472991	0.74622574	1
FBgn003165	mRpL24	0.44534543	7.78861947	0.10472055	0.74623669	1
FBgn003911[illegible]	RanBP3	0.55558383	7.16716718	0.10441571	0.74659362	1
FBgn003323[illegible]	CG8728	0.92235558	6.00222822	0.10430959	0.74671799	1
FBgn005132[illegible]	CG31324	-0.9030577	5.2113714	0.10423481	0.74680569	1
FBgn003734	CG12746	0.84598789	6.1140591	0.10420475	0.74684095	1
FBgn003651[illegible]	RhoGAP71E	2.94633161	4.77655626	0.10417871	0.7468715	1
FBgn001594[illegible]	hrg	-0.9795355	4.84253231	0.10393302	0.74715995	1
FBgn003030[illegible]	Uba5	0.89462405	6.05118748	0.1039023	0.74719605	1
FBgn001136	ND-ACP	0.38430142	8.40117669	0.10363465	0.74751075	1
FBgn002050[illegible]	CLIP-190	0.51881617	7.44161658	0.10331599	0.74788603	1
FBgn002842[illegible]	Ilk	0.72919188	6.29492397	0.10327716	0.7479318	1
FBgn003562[illegible]	TM9SF3	-0.7063243	6.39858187	0.10306413	0.7481831	1
FBgn000056[illegible]	Eip71CD	0.55754736	7.55037018	0.10295943	0.7483067	1
FBgn001402[illegible]	SdhB	-0.5692371	7.24176681	0.10290233	0.74837414	1
FBgn000465[illegible]	mys	1.69128607	4.81440025	0.10250451	0.74884458	1
FBgn003684[illegible]	Mkp3	0.53178207	7.12044151	0.10233383	0.74904672	1
FBgn002839[illegible]	TMS1	-0.3771428	8.78745048	0.10227064	0.7491216	1
FBgn002647[illegible]	Drp1	-0.4363568	7.7504561	0.10148384	0.75005619	1
FBgn025974[illegible]	CG42394	-1.219085	5.20072703	0.10147336	0.75006865	1
FBgn005316[illegible]	CG33169	-1.1793552	4.90161423	0.10123228	0.75035584	1
FBgn002175[illegible]	SerRS-m	4.37085205	4.17814776	0.10115876	0.75044348	1
FBgn002841	Nxt1	0.7751316	6.08126464	0.1011453	0.75045954	1

FBgn002574	mtm	0.48844192	7.52344573	0.10088208	0.75077366	1
FBgn026167	CG42709	1.98296444	3.3498462	0.10068587	0.75100811	1
FBgn001024	Parp	-4.0665844	6.11800543	0.10067893	0.75101641	1
FBgn003032	prtp	0.53109076	7.33691567	0.10038652	0.75136629	1
FBgn000256	lt	0.81847071	6.35058636	0.10032067	0.75144516	1
FBgn001675	sba	1.95409383	4.8856247	0.10024051	0.7515412	1
FBgn002912	Sras	1.26163453	4.39781131	0.10016183	0.75163552	1
FBgn002637	RpL23A	0.36751502	8.65474205	0.10009299	0.75171808	1
FBgn003296	CG1416	0.52688704	7.22298324	0.09985183	0.75200752	1
FBgn003680	Sgf11	-2.3137356	3.38624585	0.09953486	0.75238854	1
FBgn026335	Unr	-0.4087278	8.4468774	0.09938794	0.75256537	1
FBgn001742	CG5989	4.3198256	5.3392341	0.09881544	0.75325581	1
FBgn003464	CG10321	-2.2901582	5.25837895	0.09870366	0.75339088	1
FBgn000451	Mdr49	-2.9920626	7.38119525	0.09852043	0.75361246	1
FBgn005320	Mical	0.88487317	5.17545473	0.09851685	0.75361679	1
FBgn003371	CG8839	0.57524228	7.23875308	0.09845184	0.75369545	1
FBgn003064	CG6340	-0.6870287	7.29882504	0.09823193	0.75396179	1
FBgn000365	sws	-2.8638369	4.31491903	0.09817055	0.75403619	1
FBgn001021	ATPsynbeta	-0.3037193	11.4450522	0.09809157	0.75413196	1
FBgn003724	CG14647	0.59982139	6.83688732	0.09804717	0.75418582	1
FBgn003571	CG10103	0.73253904	6.44494777	0.0980369	0.75419828	1
FBgn004039	Unc-76	-0.3852991	8.21147487	0.09796927	0.75428033	1
FBgn003655	CG17027	3.19351775	4.38187274	0.09767514	0.7546376	1
FBgn000483	Xpac	-4.0601562	5.08934447	0.09737371	0.75500434	1
FBgn005122	CG31229	0.82138795	5.70228209	0.09712667	0.75530538	1
FBgn005219	CG32196	0.94383529	4.29513019	0.09698431	0.75547904	1
FBgn003666	Zcchc7	-3.109718	3.96994531	0.09693683	0.75553699	1
FBgn003272	L2HGDH	0.87983438	5.30722067	0.09652764	0.75603708	1
FBgn003921	Syx18	-0.7628136	6.05856181	0.09610417	0.75655585	1
FBgn008676	Pcmt	0.74659158	6.24882733	0.09603305	0.75664309	1
FBgn026154	rdgA	-0.8274324	5.66284167	0.09602071	0.75665823	1
FBgn003946	CG14252	-4.015859	6.03450646	0.09557471	0.75720622	1
FBgn003767	CG8861	-0.4820869	8.03513413	0.09555323	0.75723264	1
FBgn003713	CG7414	-0.3784761	8.80601105	0.09537708	0.75744948	1
FBgn003181	retm	-0.8001131	6.7210039	0.09531088	0.75753103	1
FBgn003166	Gmd	-2.4173349	3.11615533	0.09522425	0.75763779	1
FBgn026203	CG42846	3.2338692	4.21342126	0.09502267	0.75788642	1
FBgn004119	Rheb	0.4019348	8.09391292	0.094861	0.75808603	1
FBgn003658	PDCD-5	-3.68837	5.680112	0.09443964	0.75860718	1
FBgn001579	Rab11	-0.3411427	9.06443546	0.09371646	0.75950457	1
FBgn004109	scyl	-0.3764431	8.26983882	0.09361928	0.75962545	1
FBgn002660	Ady43A	0.75999716	6.07771602	0.09330177	0.76002087	1
FBgn001041	RpS18	0.35642883	8.76361311	0.09294609	0.7604647	1
FBgn001697	spen	-0.4323649	7.85751546	0.09287546	0.76055295	1
FBgn026138	CG17528	-0.4523031	7.52088118	0.09263913	0.76084848	1
FBgn000046	Lar	-1.3570334	5.43420979	0.09247498	0.761054	1

FBgn003842	CG17565	-0.5500914	7.54681943	0.09231572	0.76125358	1
FBgn003278	CG10195	-3.638472	3.73354814	0.09202017	0.76162447	1
FBgn003973	CG7920	0.43353568	8.46155328	0.09199936	0.76165061	1
FBgn001403	Su(Tpl)	1.11607207	4.92670003	0.09189734	0.7617788	1
FBgn003272	CG10602	4.49400267	4.60532947	0.09181725	0.76187949	1
FBgn003408	clu	-2.7523953	3.87660955	0.0916611	0.76207594	1
FBgn003842	mRpS33	0.48551997	7.32061047	0.0916087	0.7621419	1
FBgn003702	tzn	-0.5013203	7.26365857	0.09154062	0.76222763	1
FBgn003925	beta4GalT7	0.96026032	5.06501741	0.09153591	0.76223357	1
FBgn003808	beat-Va	3.31151105	5.17460283	0.09149981	0.76227905	1
FBgn001047	Fkbp14	1.09742916	3.21363345	0.09146321	0.76232516	1
FBgn000054	EcR	-2.8799889	4.38134801	0.09126173	0.76257922	1
FBgn003479	EMC8-9	0.41138052	7.81411272	0.09112524	0.7627515	1
FBgn001756	ND-23	-0.3635089	8.41245396	0.0911226	0.76275482	1
FBgn001170	Syx5	-0.4729206	8.04807442	0.09105205	0.76284393	1
FBgn003371	CG8490	-1.1528877	5.75072297	0.09087741	0.76306465	1
FBgn003888	CG16791	-4.0396061	6.11725742	0.09033676	0.76374947	1
FBgn003864	ChT	-2.3818396	3.64140051	0.09029338	0.76380451	1
FBgn003563	kri	0.43431257	7.60461637	0.09022214	0.76389494	1
FBgn025948	Mob4	0.41766627	7.70995469	0.09006124	0.76409932	1
FBgn000109	Gapdh1	0.34731201	10.2568724	0.08982615	0.76439829	1
FBgn002728	l(1)G0193	-2.3115179	6.69637714	0.08972121	0.76453188	1
FBgn003885	CG5793	-1.6838975	5.54058128	0.08964586	0.76462786	1
FBgn003847	CG3995	-1.0362345	4.36460191	0.08874254	0.76578189	1
FBgn003692	RhoGDI	-0.3726813	8.59199696	0.08835464	0.76627941	1
FBgn026797	Der-1	0.5572538	7.15888187	0.08807837	0.76663447	1
FBgn008259	akirin	0.43369223	7.75703891	0.08797408	0.76676867	1
FBgn005122	CG31221	-0.4102123	9.47941052	0.08790028	0.76686368	1
FBgn002874	CG18508	-0.5440994	7.00045462	0.08787717	0.76689345	1
FBgn003099	CG7990	-0.5906638	6.74861431	0.08752954	0.76734165	1
FBgn026000	UQCR-11	0.32459855	9.32314681	0.08736205	0.76755793	1
FBgn000032	clt	-0.9135796	5.32068129	0.0873416	0.76758436	1
FBgn026274	Fs(2)Ket	-0.6334222	6.78368964	0.0870579	0.76795129	1
FBgn000439	CrebA	0.9673986	4.36999039	0.08695895	0.76807941	1
FBgn027092	zf30C	0.83835191	5.9619964	0.08692397	0.76812474	1
FBgn002558	eIF3e	0.52085265	7.02373733	0.08681925	0.76826045	1
FBgn003710	Alg11	-0.4528644	7.54262413	0.08679712	0.76828914	1
FBgn002637	Rgl	0.89804517	5.50692775	0.08675477	0.76834407	1
FBgn003454	galla-1	0.80571517	5.94224651	0.08670216	0.7684123	1
FBgn003897	CG5377	0.61040975	7.22447803	0.08611058	0.76918124	1
FBgn001077	Ref1	0.48283078	7.39332396	0.08605273	0.76925659	1
FBgn026273	dtn	1.00075505	5.43588382	0.08595517	0.76938371	1
FBgn003262	CG12288	-3.6851593	4.28339854	0.08561607	0.7698262	1
FBgn003385	CG6145	4.25352791	5.66029866	0.0855969	0.76985125	1
FBgn003193	CG13796	-2.4400408	5.48197027	0.08540114	0.77010715	1
FBgn002618	prominin-like	-2.3812195	5.14763574	0.08520916	0.77035844	1

FBgn025078	beta-Spec	-0.5444646	6.83323342	0.08510955	0.77048894	1
FBgn003224	TBC1D16	-0.5030883	7.04448358	0.08509021	0.77051428	1
FBgn003829	CG6912	-0.7842624	5.54749296	0.08443061	0.77138062	1
FBgn026004	flr	-0.8690165	6.31955437	0.08428352	0.77157431	1
FBgn026157	raskol	1.53093522	2.96050431	0.08415344	0.77174576	1
FBgn025922	CG42324	1.92420669	5.54438814	0.08405487	0.77187578	1
FBgn005244	Arv1	1.02880302	3.31231124	0.08391566	0.77205953	1
FBgn026595	lncRNA:CR44	1.16006718	4.59752241	0.08378454	0.77223276	1
FBgn003672	UQCR-Q	0.3007287	10.3091854	0.08375467	0.77227224	1
FBgn003496	Not11	0.75296352	5.87220776	0.08367606	0.77237619	1
FBgn003653	CG18081	0.58833919	6.70106853	0.0835723	0.77251347	1
FBgn001090	Spn	-0.7645646	6.08168651	0.08345857	0.77266405	1
FBgn026261	pyd	0.86980459	5.3274763	0.08311614	0.7731181	1
FBgn003421	CG18467	-0.4467111	7.50179335	0.08304743	0.77320933	1
FBgn004066	CG13551	0.34457596	8.64705048	0.08292011	0.77337848	1
FBgn001409	CG4278	0.73653457	5.83082525	0.08288041	0.77343127	1
FBgn001033	Rac1	0.57349161	7.05904297	0.08282356	0.77350686	1
FBgn001368	mt:ND4L	-0.4450073	7.4865493	0.08260403	0.77379904	1
FBgn003474	CG4269	-2.8947447	5.44405974	0.08238087	0.77409649	1
FBgn004170	7B2	0.30563272	10.5845802	0.08236883	0.77411255	1
FBgn000264	Map205	-1.338791	5.32575139	0.08219362	0.77434641	1
FBgn003174	CG7239	0.66204037	6.40647011	0.08190052	0.77473822	1
FBgn003903	Gbp3	1.15878854	4.87872511	0.08170641	0.77499812	1
FBgn001521	eIF4E1	0.3135812	9.14501337	0.08139951	0.77540972	1
FBgn003684	MESR6	-3.1044794	3.93435463	0.08115134	0.77574317	1
FBgn003540	Alg2	-2.7512335	5.93337444	0.08101619	0.77592499	1
FBgn002608	Tip60	4.17151201	3.64802101	0.0809613	0.77599889	1
FBgn001741	cag	0.83491244	5.75234061	0.08089511	0.77608803	1
FBgn003355	CG12343	0.48939182	7.05624132	0.08085387	0.77614359	1
FBgn026662	lncRNA:CR45	0.98829021	4.15760704	0.08074749	0.77628698	1
FBgn003533	CG8993	-0.3996741	7.65627801	0.08066082	0.77640387	1
FBgn026307	CG43347	-0.9853752	4.19615938	0.08052435	0.77658808	1
FBgn003303	CG7791	0.61892133	6.77439868	0.08051674	0.77659835	1
FBgn003983	ATPsynC	-0.2627098	12.5222521	0.08049193	0.77663187	1
FBgn003046	CG1764	0.67592257	6.18358681	0.08031743	0.77686769	1
FBgn026178	SmD2	0.44660504	7.51618428	0.08023828	0.77697477	1
FBgn003301	d4	0.49788487	7.46034399	0.08003814	0.77724573	1
FBgn000017	ben	-0.4557938	7.18955626	0.08003508	0.77724988	1
FBgn026160	RpL8	0.32794856	9.21316415	0.08000813	0.77728639	1
FBgn003031	CG11696	-0.8100985	4.67205537	0.07974175	0.77764769	1
FBgn003005	CAH3	-0.9149549	5.61531175	0.07950264	0.77797255	1
FBgn003143	ND-B17.2	0.30705172	9.35839667	0.07934637	0.77818515	1
FBgn003886	CG7009	0.64040702	6.35980719	0.0792846	0.77826925	1
FBgn000307	Pgm1	-0.3901019	7.71053576	0.07919159	0.77839595	1
FBgn003528	Cpr62Bb	-1.0128823	3.79836236	0.0789781	0.77868707	1
FBgn003560	Pfdn4	0.40325488	7.60541681	0.07882446	0.77889683	1

FBgn003222	Schip1	-0.3825126	7.85115638	0.07873317	0.77902158	1
FBgn003169	Bub1	0.9802377	4.28124749	0.07866505	0.77911471	1
FBgn003462	CG10795	0.90060754	4.9368568	0.07864112	0.77914744	1
FBgn003348	RpLP0-like	0.80513807	5.60079918	0.07859658	0.77920837	1
FBgn003109	Rab35	0.42351156	7.87921425	0.07853231	0.77929632	1
FBgn026097	Ubr3	-0.7618478	5.54189705	0.07851141	0.77932492	1
FBgn028440	trol	-0.868853	5.33657823	0.07839254	0.77948772	1
FBgn026085	Sec22	0.6577637	6.31582772	0.07805887	0.77994541	1
FBgn000360	Su(var)205	0.32716832	8.5392003	0.07790081	0.78016258	1
FBgn002272	Taf8	-3.4965202	5.43495079	0.07759952	0.78057721	1
FBgn002759	cindr	-0.6612663	6.28122846	0.07757458	0.78061158	1
FBgn002959	CG14806	0.48466672	7.21472308	0.07734272	0.7809313	1
FBgn003881	Srp72	0.57165234	6.82792231	0.07731138	0.78097456	1
FBgn003240	CG6770	0.30312317	9.34807788	0.07729304	0.78099987	1
FBgn003413	Syn2	-0.8610483	4.76501504	0.07721592	0.78110638	1
FBgn002966	CG10804	-3.7790996	6.4699519	0.07711742	0.78124248	1
FBgn025917	bun	-0.3979997	7.6549527	0.07710317	0.78126219	1
FBgn003533	mRpL23	0.40753473	7.80499739	0.07708209	0.78129133	1
FBgn000011	bru1	-1.7164351	4.37354468	0.07706189	0.78131926	1
FBgn001557	alpha-Est9	-1.2241508	4.21687018	0.07694142	0.78148591	1
FBgn026048	HIP	0.85243156	4.77063311	0.07690631	0.7815345	1
FBgn003300	CG14464	0.41598198	7.47144636	0.0767608	0.78173604	1
FBgn000458	B52	-0.4892464	7.01052962	0.07669427	0.78182825	1
FBgn003524	ABCB7	1.24697739	4.25976438	0.07650652	0.78208871	1
FBgn003840	CG6126	-0.7502799	7.23217786	0.07619686	0.78251904	1
FBgn003867	CG14286	-3.9106997	4.93369582	0.0761362	0.78260345	1
FBgn000346	Sod1	0.28490276	10.3856546	0.07603241	0.78274796	1
FBgn003689	wnd	3.22052087	3.71961354	0.07598765	0.78281032	1
FBgn003984	CG1607	-0.5340468	7.06728289	0.07591741	0.7829082	1
FBgn005267	Atg8a	0.26703596	11.3762151	0.07560197	0.78334841	1
FBgn003357	Rpb5	-3.9809319	3.92515265	0.07524747	0.78384431	1
FBgn001527	Pi3K68D	-3.6649511	4.35809768	0.07492498	0.78429652	1
FBgn003600	CG3408	-2.5373636	6.01273975	0.07465863	0.78467079	1
FBgn002491	agt	0.67195819	5.98293068	0.07432403	0.785142	1
FBgn003945	Yif1	1.23843215	4.86279769	0.07395385	0.78566464	1
FBgn003640	CG6833	0.76232868	5.60367123	0.0738984	0.78574305	1
FBgn003651	SCCRO	-0.5111834	6.85528093	0.07379187	0.78589377	1
FBgn003109	CG9577	1.2412239	5.08152086	0.07372545	0.7859878	1
FBgn003975	CG1983	0.50319819	7.08902073	0.07360246	0.78616205	1
FBgn003939	CCAP-R	3.1235176	3.32415506	0.07354071	0.7862496	1
FBgn003595	CG4911	0.89376761	5.70606567	0.07338897	0.78646487	1
FBgn003992	CG11360	0.85691966	5.14373057	0.07333057	0.7865478	1
FBgn003475	CG3732	-0.4458651	7.17512574	0.07319256	0.78674391	1
FBgn003504	ND-19	-0.3390769	8.35247341	0.0731785	0.78676388	1
FBgn003352	CG17765	-0.5456729	6.80656632	0.07312636	0.78683803	1
FBgn003318	mEFTu2	0.5118682	6.77996767	0.07294217	0.78710018	1

FBgn026559	rad	1.01518501	5.561312	0.07227416	0.78805393	1
FBgn003153	CG3246	-1.7445195	6.03914537	0.07222609	0.78812275	1
FBgn003516	hng3	0.82293062	5.25831354	0.07199748	0.78845034	1
FBgn001670	Rab4	-0.345587	7.97248056	0.07189877	0.78859197	1
FBgn003001	slpr	-1.5464732	3.81855545	0.07187943	0.78861974	1
FBgn000336	sesB	-0.2677037	10.9037653	0.07181926	0.78870613	1
FBgn001490	Hydr2	-0.4141492	7.3973598	0.07150757	0.78915427	1
FBgn004000	RpL38	0.28977703	9.6927712	0.07096077	0.789943	1
FBgn002733	Kap-alpha3	0.36711068	9.06051398	0.07071289	0.79030163	1
FBgn002423	Fim	-0.3456018	7.98242735	0.07061183	0.79044802	1
FBgn000041	D1	-0.3132831	8.73382668	0.07051928	0.7905822	1
FBgn001082	Gug	0.71989163	5.50235428	0.07038449	0.79077779	1
FBgn005171	Cnot4	0.45975575	7.46555928	0.07030465	0.79089373	1
FBgn003288	CG9328	0.38823947	7.56169738	0.07026167	0.79095617	1
FBgn001560	CadN	-0.7200645	5.7148058	0.07017015	0.79108921	1
FBgn000338	Shaw	-1.2351843	4.79836783	0.06989876	0.79148425	1
FBgn001486	Pglym78	0.28486673	10.0036809	0.06984781	0.7915585	1
FBgn003484	CG3500	-0.867843	5.01606484	0.06973346	0.79172526	1
FBgn000038	cta	0.72619132	5.2807957	0.06955159	0.79199078	1
FBgn003108	Nep3	-3.6841519	6.34618729	0.0694815	0.79209321	1
FBgn004178	Pax	-2.0938076	3.96011211	0.06907933	0.79268202	1
FBgn026457	Glut1	-2.175379	4.91901888	0.06900552	0.79279029	1
FBgn000055	eEF2	0.31852243	10.5260922	0.06887518	0.79298162	1
FBgn003591	GAPsec	4.14727035	4.83230409	0.06879826	0.79309462	1
FBgn003281	Nf-YB	-0.4414529	7.21180401	0.068701	0.79323759	1
FBgn003561	l(3)psg2	-2.2046094	3.8974279	0.06863275	0.793338	1
FBgn001502	CkIalpha	-0.3035222	8.82565886	0.06843317	0.79363189	1
FBgn005235	CG32354	-1.7311691	3.85636625	0.06832901	0.79378546	1
FBgn002309	caps	1.5677474	4.26691026	0.06818222	0.79400209	1
FBgn005112	CG31122	-0.6354614	6.44204867	0.06818017	0.79400512	1
FBgn003131	CG4887	-0.6316711	6.07782125	0.06809886	0.79412522	1
FBgn026378	CG17684	-2.236141	5.17018573	0.06799098	0.79428469	1
FBgn002572	beta'COP	-0.6206724	6.84112006	0.06794273	0.79435606	1
FBgn026360	Hsc20	0.83906792	4.99966278	0.06786735	0.7944676	1
FBgn026065	CG42541	2.56315689	5.26430653	0.06782985	0.79452312	1
FBgn003910	SPE	-0.8900373	5.81330198	0.06744868	0.79508837	1
FBgn002999	UbcE2H	-0.5978721	6.44590529	0.06743257	0.7951123	1
FBgn003355	CG7222	-0.5194265	6.71855963	0.06733709	0.79525418	1
FBgn003491	CG5569	0.5540189	6.4851776	0.06727022	0.79535361	1
FBgn003247	CG16974	1.2681662	5.52084577	0.06726075	0.79536769	1
FBgn000149	l(1)10Bb	0.58091626	6.32040613	0.06725378	0.79537807	1
FBgn002839	Taf10	-0.659631	5.92503546	0.06713382	0.79555659	1
FBgn026339	hts	-0.3861171	7.54036434	0.06664216	0.79629005	1
FBgn000490	Rb97D	-1.5925538	3.31817625	0.06658708	0.79637239	1
FBgn005163	CG31637	-4.0757029	4.13044008	0.06650838	0.79649012	1
FBgn000196	Arpc1	0.44993332	7.01132051	0.06642058	0.79662154	1

FBgn000347	spir	-0.4152035	7.22166485	0.06634706	0.79673166	1
FBgn000278	mod(mdg4)	0.47605509	7.1820296	0.066271	0.79684566	1
FBgn002643	Eaat2	-0.7709958	6.31395421	0.06625833	0.79686465	1
FBgn004093	CG9034	0.30216903	8.5271169	0.06621097	0.79693568	1
FBgn003103	CG14207	0.33269243	7.97967307	0.06570769	0.79769208	1
FBgn026359	l(3)72Ab	3.92259202	3.69124792	0.06566275	0.79775978	1
FBgn026779	HnRNP-K	0.42468923	7.13238427	0.06555029	0.79792929	1
FBgn002950	Coq7	0.30366036	8.77570405	0.06545967	0.79806599	1
FBgn002727	l(1)G0196	3.16032029	4.56028437	0.06541201	0.79813793	1
FBgn001166	msi	-1.7233137	5.75627295	0.06515919	0.79851999	1
FBgn003122	ND-15	-0.3130792	8.2862926	0.06504528	0.79869238	1
FBgn026044	rhea	3.02411637	6.23934934	0.0649627	0.79881748	1
FBgn003862	CG7694	0.65052027	5.97052604	0.06487242	0.79895431	1
FBgn003474	CG17807	-2.203035	5.05654784	0.06478345	0.79908927	1
FBgn005305	CG33051	-0.7656759	5.62748506	0.06471362	0.79919527	1
FBgn000366	Syb	0.62276711	7.14120491	0.06457145	0.79941125	1
FBgn003363	CG9003	3.76627708	4.69721528	0.06442568	0.79963297	1
FBgn003972	eIF2Balpha	-0.8605013	4.99252969	0.06412253	0.80009492	1
FBgn026454	hwt	-2.5218258	5.18868673	0.06411957	0.80009943	1
FBgn003051	CG9941	-1.8710463	6.58084874	0.06403582	0.80022725	1
FBgn003814	Droj2	-0.3277372	9.38994072	0.06389643	0.80044021	1
FBgn002849	CG7985	0.68845979	5.46861585	0.06385152	0.80050888	1
FBgn026518	CG44247	-1.6167216	6.84261704	0.0637937	0.8005973	1
FBgn003114	CG1532	-0.4709553	6.89113486	0.06347807	0.80108084	1
FBgn003436	mRpS28	-0.5663756	6.59183488	0.0634745	0.80108631	1
FBgn003066	CG9240	-0.5450376	6.48690658	0.06326059	0.80141474	1
FBgn026063	eIF4G2	-1.1844311	4.22789312	0.0630313	0.80176745	1
FBgn006336	Gpb5	0.53381592	6.875673	0.06300484	0.8018082	1
FBgn005010	CG30105	-3.6861832	4.40098953	0.0629026	0.80196572	1
FBgn003121	galectin	0.38680961	7.38050686	0.0627605	0.80218489	1
FBgn005817	CG40178	-0.2854197	8.8306652	0.06260851	0.8024196	1
FBgn002234	CG1910	-1.272801	5.83231052	0.06257897	0.80246525	1
FBgn001125	Sema1a	-0.8740561	5.98320603	0.062442	0.80267708	1
FBgn028345	Glo1	0.70813854	5.47825973	0.06224874	0.80297638	1
FBgn008522	Ufm1	0.58681428	6.14443589	0.06212917	0.80316181	1
FBgn028595	caz	0.80046693	4.415295	0.06201999	0.80333128	1
FBgn003205	mRpL51	-0.495621	6.72067631	0.06181999	0.80364217	1
FBgn003406	CG8397	-0.3787393	7.51222361	0.06178109	0.80370269	1
FBgn002194	Coprox	0.93497299	6.45276389	0.06138034	0.80432741	1
FBgn005401	CG34015	3.62025827	3.51863086	0.06107342	0.80480732	1
FBgn003769	CG16779	-2.2493387	5.94852761	0.06078474	0.8052599	1
FBgn003787	SdhC	0.25820326	9.95498751	0.06050503	0.8056995	1
FBgn001176	E2f1	-0.8182721	4.65221663	0.06042624	0.80582352	1
FBgn002989	Pink1	-0.6773138	5.40901092	0.06009591	0.80634442	1
FBgn000438	Klp98A	0.87986198	3.96624842	0.05988635	0.80667567	1
FBgn003913	AP-1sigma	-0.3770711	7.64778093	0.05985474	0.80672567	1

FBgn026314	bin3	0.77647458	4.90427578	0.05984492	0.80674123	1
FBgn004078	CG14104	0.88625891	3.74447855	0.05983564	0.80675591	1
FBgn003788	CG17187	-0.7307682	5.77802061	0.05976766	0.80686355	1
FBgn000194	eIF4A	0.26238288	10.176941	0.0597563	0.80688153	1
FBgn003044	CG2200	-1.2734688	5.04718064	0.05970508	0.80696268	1
FBgn026195	east	-0.7633675	4.98683438	0.05959834	0.80713191	1
FBgn001343	bcn92	-0.5772909	6.22550425	0.05957829	0.80716371	1
FBgn000320	Ras64B	0.38742269	7.37225355	0.05957237	0.8071731	1
FBgn003203	CG13390	-0.8055689	4.79520882	0.05931663	0.80757932	1
FBgn004345	CG5986	0.42214486	7.29983343	0.05928422	0.80763085	1
FBgn003646	mRpL39	-3.5142611	6.33795463	0.05925235	0.80768156	1
FBgn002038	Gcn5	-1.6368495	6.16283246	0.05911876	0.80789423	1
FBgn003846	CG3678	-0.7747049	4.70380721	0.05906333	0.80798256	1
FBgn004301	Fsn	-0.7983718	5.86747461	0.05904466	0.80801232	1
FBgn003204	CG13398	-1.7325046	6.49932751	0.05901072	0.80806642	1
FBgn006151	endos	0.50790204	6.74944007	0.0587761	0.80844093	1
FBgn000371	tipE	-1.5678151	4.6029856	0.05869838	0.80856516	1
FBgn000313	Pp1alpha-96	-0.4376548	6.89099686	0.05867613	0.80860074	1
FBgn026044	GABA-B-R1	2.79504813	4.31511406	0.0586566	0.80863198	1
FBgn001540	kek2	-1.7534267	4.38787557	0.05856052	0.80878573	1
FBgn003804	Srlp	0.31722742	7.967147	0.05840653	0.80903244	1
FBgn004079	CG7630	0.25418218	9.98032101	0.0582581	0.80927058	1
FBgn000541	U2af50	0.43431933	7.00291738	0.05803849	0.80962348	1
FBgn002849	CG31705	-0.3114898	8.11063662	0.05797496	0.80972572	1
FBgn001672	RpL29	0.33920966	7.99501435	0.05783343	0.80995366	1
FBgn005319	dpy	1.13291986	3.39014359	0.05778395	0.81003341	1
FBgn001583	eIF3i	-0.2692948	9.00204969	0.05760639	0.81031994	1
FBgn008397	Nlg4	-1.7032427	3.15909274	0.05757325	0.81037347	1
FBgn003735	CG2911	-1.6335627	3.59645883	0.05737687	0.810691	1
FBgn002988	ND-ASHI	-0.3015024	8.35769793	0.05737264	0.81069785	1
FBgn008524	CG34213	0.73095164	5.02875941	0.05731509	0.81079101	1
FBgn005263	CG32638	4.00386505	4.53015018	0.05727927	0.81084903	1
FBgn025974	mmy	-0.3118586	7.93722788	0.0571708	0.81102481	1
FBgn026339	sqd	-0.3251173	7.82108966	0.05713394	0.8110846	1
FBgn003586	Arl5	0.56455881	6.28523289	0.05708362	0.81116623	1
FBgn000264	mam	1.9362582	5.30841161	0.05699936	0.81130302	1
FBgn002631	Ubc10	-0.5346022	6.31123775	0.05685428	0.8115388	1
FBgn003956	Gp93	2.42027504	6.86122166	0.05675495	0.8117004	1
FBgn001529	Ssb-c31a	0.29285779	8.24492464	0.05652235	0.81207942	1
FBgn003412	CG4409	-0.5995769	6.13958034	0.05645453	0.8121901	1
FBgn001669	ATPsynO	0.23593473	10.9467182	0.05644936	0.81219853	1
FBgn026797	Tim23	-0.3193729	7.8569957	0.056421	0.81224484	1
FBgn003761	CG8043	0.77855891	4.85566685	0.0562456	0.81253147	1
FBgn003442	CG10737	0.66386216	6.72257465	0.05616626	0.81266128	1
FBgn003142	VGlut	0.27179231	9.0480326	0.05612828	0.81272345	1
FBgn003598	CG4080	-0.8397737	3.72655102	0.05603059	0.81288348	1

FBgn003728	CG14657	-1.4666319	4.52764743	0.05595592	0.81300589	1
FBgn005017	CG30172	0.29401934	8.4536398	0.0557535	0.81333817	1
FBgn001061	ATPsynG	0.25731646	9.26299377	0.05562718	0.81354584	1
FBgn002480	DIP2	0.91834383	4.23905393	0.05518115	0.81428116	1
FBgn026393	esn	-1.6274071	6.22633312	0.05503244	0.81452702	1
FBgn026251	VhaAC45	-0.2678948	8.8104855	0.0549141	0.81472291	1
FBgn003424	UQCR-6.4	0.28860663	8.20482191	0.0548669	0.81480112	1
FBgn003723	Vps24	-0.8298953	3.09340596	0.05470993	0.81506142	1
FBgn001065	MFS14	-2.3847248	5.42319765	0.05448138	0.81544113	1
FBgn001367	mt:CoII	-0.2131994	12.7210065	0.05445419	0.81548636	1
FBgn003197	CG12560	-3.1001764	5.62195222	0.05439087	0.81559175	1
FBgn026472	lncRNA:CR43	-1.6244398	3.40401872	0.05429166	0.81575697	1
FBgn002993	CG4615	-1.6269238	3.96752929	0.05418242	0.81593909	1
FBgn000002	acj6	2.92655192	2.47615466	0.0540794	0.81611103	1
FBgn000490	Arl2	0.68916766	4.99245176	0.05401455	0.81621934	1
FBgn004049	grsm	3.84864537	4.75185972	0.05379458	0.81658726	1
FBgn003487	levy	0.24654115	9.75397513	0.0537415	0.81667617	1
FBgn003096	shop	-0.7631915	4.81771198	0.05365342	0.81682379	1
FBgn003277	CG17549	0.67810925	5.7557587	0.05363954	0.81684706	1
FBgn002858	lqf	-0.7711781	5.58223757	0.05357941	0.81694793	1
FBgn001367	mt:CoI	-0.2093669	13.3163491	0.05337445	0.81729219	1
FBgn005283	CG33695	0.56454512	6.26453797	0.0532975	0.81742162	1
FBgn003926	veli	0.36968575	7.51080735	0.05318135	0.81761716	1
FBgn004079	CG13056	-0.9474277	5.44448592	0.05316681	0.81764165	1
FBgn001757	Max	-0.5081239	6.365321	0.05314322	0.8176814	1
FBgn003248	Ski6	-2.0668991	5.12801697	0.05305774	0.81782552	1
FBgn008538	CG34353	3.78186998	5.18135253	0.05297817	0.81795978	1
FBgn003587	CG7182	0.59667035	6.3355419	0.05294995	0.81800742	1
FBgn003825	Hexim	0.46422314	6.68962146	0.05294798	0.81801074	1
FBgn028602	Rnmt	-1.0878857	5.45589476	0.05287076	0.81814116	1
FBgn003219	CG5727	0.61691869	5.62465056	0.05284332	0.81818755	1
FBgn000440	RpS14b	0.33120317	7.67079242	0.05282148	0.81822446	1
FBgn026287	axo	-1.0151277	3.53152599	0.05255446	0.81867648	1
FBgn002674	Yippee	-0.3713947	7.17335907	0.05240136	0.8189362	1
FBgn026505	St3	-0.3573039	7.33744695	0.05236931	0.81899062	1
FBgn002967	HIP-R	-0.8587278	3.90749401	0.05228061	0.81914132	1
FBgn003326	Socs44A	0.6675378	5.86917279	0.05216417	0.81933935	1
FBgn002991	CG14435	-1.8457122	4.23801187	0.05209136	0.81946331	1
FBgn001145	ND-SGDH	0.24631092	9.46144019	0.05179142	0.81997488	1
FBgn026667	Sec8	-0.7250278	7.06101286	0.05173066	0.8200787	1
FBgn026250	CG43078	-2.9790386	3.79869385	0.05165523	0.82020769	1
FBgn003058	CG14407	0.31667416	7.78235233	0.05153204	0.82041854	1
FBgn003824	CG8066	0.5755297	6.41383536	0.05148147	0.82050516	1
FBgn026672	Trs31	0.38049407	7.10820564	0.05122046	0.82095304	1
FBgn002041	Idgf3	-0.7018982	6.84956537	0.05058585	0.82204696	1
FBgn000042	Ddc	-0.2557373	10.3794595	0.05021187	0.82269501	1

FBgn003776	Teh1	-2.8115708	4.35397438	0.05014575	0.82280986	1
FBgn003235	CG4788	0.63783971	5.57051935	0.05000453	0.82305539	1
FBgn003820	CG12402	0.64840052	6.80415247	0.04992209	0.82319889	1
FBgn003246	Yip1d1	-0.4862826	6.41621695	0.04968025	0.8236206	1
FBgn026374	eIF2gamma	-0.6025562	6.41957099	0.04945445	0.82401531	1
FBgn026599	Zasp52	0.76847056	3.53757462	0.04943956	0.82404137	1
FBgn000278	Rpn8	-0.6464231	6.23705872	0.04929288	0.82429832	1
FBgn001668	Nlp	-0.3971867	7.19029012	0.04924498	0.82438231	1
FBgn028443	tyn	1.54726155	3.71811676	0.04921065	0.82444253	1
FBgn000417	mts	-0.2462646	9.25121629	0.04913768	0.82457062	1
FBgn002473	RpL10	0.24347766	9.12264798	0.04901997	0.82477745	1
FBgn003247	DnaJ-H	-0.3515783	7.2460082	0.0486793	0.82537754	1
FBgn003069	CG8974	0.29150524	7.84435321	0.04867173	0.8253909	1
FBgn003135	mRpL48	0.35599185	7.17352417	0.04856959	0.82557125	1
FBgn003682	RpL26	0.30512165	8.27775349	0.04854813	0.82560918	1
FBgn001023	Lac	1.08250073	4.44579621	0.0484755	0.82573757	1
FBgn003089	CG7206	-3.1528367	4.40770244	0.04807127	0.82645406	1
FBgn003653	CG15715	-0.5057869	6.2958969	0.04797286	0.82662897	1
FBgn001397	Gycbeta100E	-3.0079217	4.67488383	0.04793374	0.82669853	1
FBgn002846	rtet	-2.056626	6.0767141	0.04769937	0.82711602	1
FBgn026449	Eip93F	0.49772808	6.48531001	0.04769698	0.82712028	1
FBgn000119	His2Av	0.33397134	7.42506587	0.04740134	0.82764845	1
FBgn002657	Pisd	0.93700868	5.04167432	0.04738071	0.82768538	1
FBgn026324	Mocs1	0.62919647	5.21470595	0.04715956	0.8280817	1
FBgn003911	SdhD	0.2372419	9.20391685	0.047157	0.82808628	1
FBgn004121	HDAC4	-1.5094485	5.2127071	0.0469156	0.82852002	1
FBgn002989	CG3168	0.41787729	7.41299122	0.04662811	0.82903808	1
FBgn005300	mRpL27	0.32712364	7.42391077	0.04653383	0.82920834	1
FBgn028366	Rap2l	0.55532462	5.7893504	0.04629497	0.82964051	1
FBgn003032	CG1703	0.73089234	4.04192009	0.04608956	0.83001308	1
FBgn026302	CG43324	0.33007569	7.42182969	0.04537607	0.83131403	1
FBgn001368	mt:ori	0.7493011	3.29939204	0.04505673	0.83189974	1
FBgn002883	CSN7	0.32165565	7.49004967	0.04500149	0.83200129	1
FBgn001182	CG4038	-1.0234831	3.76595688	0.04494663	0.8321022	1
FBgn005377	CG33774	0.6159624	5.04575979	0.04483362	0.83231027	1
FBgn026572	Nna1	1.36513997	2.94176052	0.04479772	0.83237642	1
FBgn003405	Pex11	0.61909343	5.32872948	0.04469491	0.83256603	1
FBgn003898	CG6937	-2.3683486	5.31149412	0.04465362	0.83264224	1
FBgn026273	mbf1	-0.3212836	7.4091668	0.0445811	0.83277619	1
FBgn025993	Nop60B	1.39334996	4.0387979	0.0439827	0.83388581	1
FBgn003980	dj-1beta	0.27883884	7.83708458	0.04397691	0.83389658	1
FBgn003024	CG1637	0.26893706	8.18554288	0.04391635	0.83400932	1
FBgn003714	CG7470	3.74021296	4.58460157	0.04390179	0.83403643	1
FBgn008316	Neb-cGP	0.22558112	9.6270849	0.04387558	0.83408527	1
FBgn003715	CG7130	-0.7399097	3.85247361	0.04356829	0.83465887	1
FBgn025970	CG42359	-2.7665883	3.69370232	0.0435505	0.83469215	1

FBgn001612	Alp4	-0.506971	6.58286268	0.0435135	0.83476138	1
FBgn003543	PIG-C	0.73492117	3.49339509	0.04340631	0.83496209	1
FBgn003451	CG13428	0.4780809	6.25899	0.04337741	0.83501625	1
FBgn003892	Fadd	-0.7998009	5.58796247	0.0433346	0.83509652	1
FBgn000112	Galphas	0.32304592	7.37052434	0.04324366	0.83526715	1
FBgn003045	CG12096	0.39926039	6.80235678	0.04323461	0.83528414	1
FBgn001059	Prosbeta1	0.35042493	7.30911448	0.04323292	0.83528731	1
FBgn003845	Arl6IP1	0.36304859	8.01145474	0.04305185	0.83562768	1
FBgn003664	Syx8	-0.4109295	7.46313636	0.04303029	0.83566826	1
FBgn000272	Met	0.6905893	4.23181237	0.04286581	0.83597815	1
FBgn002757	mino	-1.1097484	6.16002637	0.04276945	0.83616	1
FBgn003886	CG5862	-0.5089759	5.98938183	0.04269293	0.83630455	1
FBgn003310	CG9436	-0.7051029	5.96110888	0.04251479	0.83664161	1
FBgn026247	FoxP	0.39286811	6.84875099	0.04249526	0.83667861	1
FBgn008543	pan	-2.5467076	6.95223037	0.04249004	0.83668848	1
FBgn003719	CG11137	-0.2810446	7.7861935	0.04246495	0.83673604	1
FBgn003650	CG7945	0.27020063	8.19262297	0.04243096	0.83680048	1
FBgn001176	ctp	0.23326127	9.46906975	0.04239893	0.83686122	1
FBgn003683	CG18135	-1.2788712	6.48223326	0.04231972	0.83701154	1
FBgn026529	SC35	-0.317973	7.37881285	0.04224406	0.83715525	1
FBgn002347	teq	0.45897344	6.88682022	0.04224058	0.83716186	1
FBgn005351	dpr4	-0.7034731	4.17974238	0.04210564	0.83741854	1
FBgn003606	CG6674	0.4629975	6.31133197	0.04207978	0.83746777	1
FBgn026155	CG42674	0.46248794	7.2794229	0.04201879	0.83758395	1
FBgn003940	Vps2	0.39062721	7.22838226	0.04166268	0.83826411	1
FBgn002992	CG4593	0.47760788	7.26547925	0.04152249	0.83853271	1
FBgn003760	CG9773	0.44497171	6.82354242	0.04152032	0.83853687	1
FBgn003041	Tomosyn	-0.3243676	7.58272949	0.04148741	0.8386	1
FBgn003182	Phf5a	0.49560616	6.26959334	0.04144963	0.83867249	1
FBgn003787	CG6689	3.31005439	5.34839161	0.0414443	0.83868272	1
FBgn003049	CG11178	-3.2702807	4.30223489	0.04139298	0.83878125	1
FBgn005203	CG32039	0.55628034	5.40317648	0.04122857	0.83909736	1
FBgn025999	lncRNA:CR42	-0.2476636	8.56220712	0.0411865	0.83917836	1
FBgn003527	CG2021	0.54487004	5.61239804	0.04093939	0.83965496	1
FBgn003667	CG9951	3.17690032	4.36470583	0.040814	0.83989738	1
FBgn001043	mtSSB	0.69839696	3.25005203	0.04074875	0.84002368	1
FBgn003291	CG9253	-3.0860598	3.97965244	0.04069328	0.84013114	1
FBgn002061	Rack1	0.25586167	8.13370582	0.0406601	0.84019544	1
FBgn003035	SelG	0.50035352	6.13118268	0.04056174	0.84038625	1
FBgn003239	Nfs1	0.2744081	7.75819721	0.0404746	0.84055549	1
FBgn003763	IscU	-0.203226	10.7772361	0.04040246	0.84069573	1
FBgn004028	Scamp	0.40780601	6.73055802	0.04038879	0.84072234	1
FBgn002317	RpL39	0.26035201	8.43007117	0.04024078	0.84101055	1
FBgn000005	adp	0.94709796	6.02594693	0.04020798	0.84107448	1
FBgn003006	CG2004	3.19772178	4.30891129	0.04016764	0.84115317	1
FBgn001551	nAChRalpha3	0.534822	5.53267868	0.03970727	0.84205402	1

FBgn002869	RpL15	0.21779516	9.38692724	0.03970686	0.84205484	1
FBgn000110	Gbeta13F	-0.2812077	8.20861238	0.03960407	0.84225671	1
FBgn000436	Ptp4E	1.14416281	4.53394312	0.03952781	0.84240667	1
FBgn003106	COX6B	0.2118697	9.68477358	0.03952594	0.84241034	1
FBgn005199	CG31998	-3.2298937	6.00665346	0.0394909	0.8424793	1
FBgn000320	Ras85D	0.23401502	8.48132599	0.03922608	0.84300144	1
FBgn003873	CG4686	0.44029301	6.35333493	0.03917106	0.84311017	1
FBgn003881	CG5466	-3.4329403	4.90307989	0.03912397	0.84320327	1
FBgn008690	Nacalpha	0.22713133	8.74854376	0.03909657	0.84325748	1
FBgn001023	Klc	-0.294454	7.51250335	0.03908491	0.84328055	1
FBgn004118	Socs36E	-0.6367748	4.73992145	0.03904752	0.84335456	1
FBgn028677	CG46385	0.35077973	7.19094886	0.03903094	0.84338739	1
FBgn025075	kra	-0.2154355	9.22665883	0.03900858	0.84343166	1
FBgn000423	Hrb87F	3.31717376	4.13564826	0.03900518	0.84343841	1
FBgn003309	CG9422	-1.2333442	4.95000176	0.03883084	0.84378416	1
FBgn003237	CycY	0.66466061	6.02727339	0.03866966	0.84410455	1
FBgn005260	dpr8	-2.5895634	4.85891534	0.03857862	0.84428583	1
FBgn026432	spg	-1.974402	4.18513093	0.03845123	0.84453983	1
FBgn003733	mRpL44	-0.5214755	5.62960342	0.03827543	0.84489111	1
FBgn003552	VhaM9.7-a	-0.2125134	10.095767	0.03825071	0.84494056	1
FBgn003747	CG2656	0.45527488	6.17714701	0.03824334	0.84495532	1
FBgn003126	Ipk2	3.54198023	4.74293716	0.03782235	0.8458003	1
FBgn026360	Zn72D	0.47310581	7.0224035	0.03764972	0.8461482	1
FBgn003522	pns	3.38038611	3.65175028	0.03743578	0.8465805	1
FBgn005106	CG31064	0.35414995	7.31201841	0.03730463	0.84684614	1
FBgn005218	CG32187	0.42969273	6.78990717	0.03723495	0.84698748	1
FBgn026182	Asx	-2.0833694	3.39999618	0.03721529	0.84702737	1
FBgn003457	mRpL54	-0.3659446	6.84842342	0.03705732	0.84734839	1
FBgn003290	RPA2	0.48308878	6.08210706	0.03696813	0.84752995	1
FBgn003407	Dg	2.62536698	3.58074937	0.03694689	0.84757322	1
FBgn003729	Vps37B	0.37766577	6.68835644	0.03693557	0.8475963	1
FBgn026155	CG42671	-0.5501476	5.69816153	0.03687764	0.84771438	1
FBgn003126	Tspo	0.59574217	4.77220312	0.03686341	0.8477434	1
FBgn002294	Cbp20	0.39425482	6.74649416	0.03678535	0.84790273	1
FBgn003804	CG17202	0.38496989	6.64568935	0.03666994	0.84813861	1
FBgn026548	mbl	-0.2183144	9.49747407	0.03652031	0.848445	1
FBgn003493	Stoml2	-0.2937775	7.38514274	0.03646203	0.84856452	1
FBgn004080	CG12355	-0.4066434	6.50134465	0.03637143	0.84875048	1
FBgn005846	lncRNA:CR4	0.19873032	10.6102012	0.03633187	0.84883179	1
FBgn002179	Tapdelta	0.29073589	7.90178464	0.03632128	0.84885355	1
FBgn003914	CG18428	-0.519275	5.58131745	0.03613615	0.8492346	1
FBgn003388	CG16935	0.31106054	7.28446026	0.0360939	0.8493217	1
FBgn008395	CG34117	-0.3052884	7.32786988	0.03603536	0.84944249	1
FBgn002751	CG7115	-0.5561466	6.00967537	0.03597266	0.84957197	1
FBgn004218	MCU	3.07272876	3.4492039	0.03594616	0.84962672	1
FBgn003680	Chmp1	-0.23841	8.088226	0.03575621	0.85001982	1

FBgn005317	CG33170	0.47279385	5.97172264	0.03570715	0.85012153	1
FBgn003388	Rpn13	0.32511475	7.23112864	0.03556424	0.85041823	1
FBgn002666	MagR	-0.4160089	6.45423835	0.03540759	0.85074413	1
FBgn003867	mRpL55	-0.4206057	6.57130861	0.03513115	0.85132111	1
FBgn000446	Su(P)	0.32070085	7.43771715	0.03507162	0.85144567	1
FBgn002735	Tim8	0.29451522	7.32530362	0.03474463	0.85213179	1
FBgn002984	raptor	0.63956361	2.90341917	0.03472456	0.852174	1
FBgn003048	GstT4	3.26970283	3.99203758	0.03446241	0.85272666	1
FBgn026127	Ero1L	-0.5890149	5.07407534	0.03445075	0.85275129	1
FBgn003787	CG4820	0.48815691	5.90689272	0.03442612	0.85280333	1
FBgn002234	CG10340	0.23847876	7.97704644	0.03429697	0.85307656	1
FBgn028347	S6k	0.70062061	5.87242518	0.03415368	0.8533803	1
FBgn003974	CG7946	-0.4848843	6.86147145	0.03412497	0.85344125	1
FBgn004432	Chro	-1.3182842	5.02260568	0.0341045	0.85348471	1
FBgn002982	CG16721	-3.151764	5.37724765	0.03389165	0.85393748	1
FBgn003892	CG13409	3.48962715	4.34319883	0.03381472	0.85410148	1
FBgn026600	CG44774	-0.9360978	5.18514276	0.03339902	0.85499105	1
FBgn003251	RpL24	0.19647353	9.68314098	0.0333704	0.85505251	1
FBgn002755	CG8042	-0.3760218	7.02418183	0.03336805	0.85505756	1
FBgn001059	Sply	0.39988352	6.4362492	0.03336431	0.85506559	1
FBgn008312	Uhg5	0.56629034	5.20886762	0.03332407	0.85515205	1
FBgn026351	nclb	0.68013431	6.5278626	0.03313561	0.8555577	1
FBgn003081	CG4991	0.71859715	5.10162288	0.03303855	0.85576707	1
FBgn003881	Nep4	0.76043905	3.66915536	0.03302146	0.85580398	1
FBgn003094	Nup35	-2.3227305	5.98443706	0.03284791	0.85617927	1
FBgn003980	CG15546	-0.6369655	4.08485745	0.03284712	0.85618098	1
FBgn003936	CLS	-0.4220509	6.46981268	0.03279218	0.85629999	1
FBgn003583	CG8038	0.53397593	5.16855883	0.03268104	0.85654106	1
FBgn003518	Trh	-3.3108653	4.45276386	0.03267662	0.85655066	1
FBgn003272	mRpL13	0.50062937	5.53143914	0.0325512	0.85682324	1
FBgn008544	sif	-0.5276322	6.38438936	0.03252395	0.85688254	1
FBgn003397	CG10209	0.39951026	6.74804003	0.03245002	0.85704353	1
FBgn002749	wdb	-0.4748851	6.69416647	0.03241121	0.85712812	1
FBgn003830	mRpS10	0.46141253	5.98943773	0.03240872	0.85713355	1
FBgn003094	CG6617	0.2689914	7.51761132	0.03221751	0.85755111	1
FBgn000556	Shal	-2.0788126	4.82263434	0.03213138	0.85773962	1
FBgn002657	CG8677	-0.4231191	6.5181243	0.03210687	0.8577933	1
FBgn003417	CG6665	-3.196504	5.89924289	0.03208866	0.8578332	1
FBgn003583	CG7565	-0.7301367	5.86729162	0.03188578	0.85827856	1
FBgn002289	Df31	-3.1874064	5.22826986	0.03187863	0.85829429	1
FBgn003340	CG2063	0.35288879	6.79653758	0.03174824	0.85858135	1
FBgn003067	HUWE1	-1.2116912	6.67247692	0.03163575	0.85882946	1
FBgn003015	CG1354	-1.0604635	6.32627804	0.03156255	0.85899117	1
FBgn001122	ox	0.21847455	8.29100039	0.03155882	0.85899941	1
FBgn002234	CG3760	0.4054761	6.38202927	0.03136826	0.85942132	1
FBgn003163	tank	0.60071964	4.3384124	0.03135569	0.85944917	1

FBgn004211	mRpL36	0.28631626	7.17675114	0.03133437	0.85949647	1
FBgn003583	CG8209	2.32153204	5.25277218	0.03125702	0.85966819	1
FBgn003167	SelT	0.21958552	8.19125459	0.03108699	0.86004646	1
FBgn026255	Mdh2	0.19167986	9.41581167	0.03097338	0.86029979	1
FBgn003832	CG6218	-0.2563352	7.72671402	0.03091856	0.8604222	1
FBgn002995	CG15478	-0.6192276	3.75143843	0.03077869	0.86073504	1
FBgn000486	Gdi	0.18876621	9.69232756	0.03076474	0.86076628	1
FBgn026211	IntS3	-2.9217802	5.3599328	0.03069572	0.86092096	1
FBgn003613	mRpL2	0.29710054	7.07146221	0.03066851	0.86098199	1
FBgn003925	Nmnat	-0.3367203	7.16824638	0.03063385	0.86105978	1
FBgn003065	cerv	0.41398746	6.1347549	0.03038844	0.86161178	1
FBgn003194	CG7154	2.7182233	2.26643501	0.0302457	0.86193391	1
FBgn003497	CG4049	-3.0148566	4.09711727	0.02999396	0.86250394	1
FBgn003625	Atg12	-0.507512	5.21799642	0.02979793	0.86294953	1
FBgn003764	ScsbetaA	0.21087035	9.06142088	0.02978362	0.86298211	1
FBgn005240	CG32409	0.56643679	4.73144097	0.02969261	0.86318956	1
FBgn003163	mRpS2	0.31849687	6.87361395	0.02954118	0.86353542	1
FBgn004023	c11.1	2.05405548	6.146265	0.02953855	0.86354144	1
FBgn002709	AlaRS	0.44091216	6.44554956	0.02951497	0.86359537	1
FBgn003925	CG11781	0.35196643	6.56781227	0.0294954	0.86364017	1
FBgn026273	CG33941	0.77997971	5.18254543	0.02943922	0.86376883	1
FBgn003631	Wbp2	0.20832308	8.63336341	0.02943598	0.86377624	1
FBgn003853	CG14322	0.57935247	4.59786659	0.02935015	0.86397305	1
FBgn001327	Hsp70Bb	-0.198621	8.77180574	0.02928784	0.86411612	1
FBgn026251	Rab8	-0.3378169	6.67577257	0.02916357	0.8644019	1
FBgn003551	CG14997	0.29583258	7.36294888	0.02910377	0.86453966	1
FBgn028350	Neurl4	3.21819865	3.57784147	0.0290993	0.86454997	1
FBgn004077	CG14767	0.18229638	9.99927681	0.02909148	0.86456799	1
FBgn008647	RpS25	0.17782391	10.2641348	0.02903812	0.86469106	1
FBgn003232	CG16743	-0.3053098	6.95725401	0.02878637	0.86527323	1
FBgn000307	Pfk	-0.2788118	7.25809477	0.02855758	0.86580459	1
FBgn003270	Irk3	0.51759098	5.30121314	0.02845606	0.86604108	1
FBgn000389	tud	-0.4880451	6.11511496	0.0284478	0.86606034	1
FBgn026271	Arp3	0.23280865	8.14262664	0.02816473	0.86672213	1
FBgn003560	Cyt-c1	-0.1735689	10.3895011	0.02803258	0.86703225	1
FBgn004032	Ephrin	-3.0066719	5.74600771	0.02801876	0.86706472	1
FBgn000071	fog	-1.6646993	3.81008968	0.02799777	0.86711406	1
FBgn000337	sgg	-0.3092849	7.23449181	0.02798934	0.86713389	1
FBgn003558	CG10672	0.37168033	6.50458356	0.02794631	0.86723511	1
FBgn025992	Sep-04	0.27185179	7.35586804	0.02789577	0.86735411	1
FBgn001501	CCT3	0.37658662	7.19652124	0.02784322	0.86747795	1
FBgn003168	ND-13A	-0.1919912	8.72146494	0.02783406	0.86749955	1
FBgn008665	shrb	0.26607015	7.3758919	0.02779269	0.86759713	1
FBgn003281	CG10268	0.54474782	5.87540458	0.02774334	0.86771366	1
FBgn003895	CG7059	-0.6557249	4.83947182	0.02760394	0.86804338	1
FBgn003563	CG10576	2.9339929	5.27748083	0.02759678	0.86806032	1

FBgn002038	Papss	0.28218324	7.10939205	0.027588	0.86808112	1
FBgn003472	mRpS29	-0.3739023	6.50667924	0.02755092	0.86816898	1
FBgn026259	Shab	-3.1247805	5.63212398	0.02747322	0.86835332	1
FBgn003318	CG1603	-0.5702015	4.27030407	0.02741378	0.86849452	1
FBgn026657	tau	-0.1915982	8.78347198	0.02711672	0.86920251	1
FBgn005276	CG32767	0.41766838	6.18565741	0.02696748	0.86955971	1
FBgn008398	mRpL34	-0.4866574	4.94621505	0.02681604	0.8699232	1
FBgn001995	ND-42	0.18233751	9.34597177	0.0267547	0.87007074	1
FBgn003308	CG15908	0.40144837	6.01026632	0.0266498	0.87032343	1
FBgn003542	Larp4B	-1.0432233	4.67708926	0.02663952	0.87034823	1
FBgn003682	Grx1	0.30257342	6.85872946	0.02659986	0.87044393	1
FBgn003838	blp	0.4048814	5.95945661	0.02653342	0.87060439	1
FBgn003833	Ccm3	-0.3558538	6.58663973	0.02591578	0.87210616	1
FBgn003725	CG1074	-2.4169858	3.17496135	0.02577596	0.87244868	1
FBgn005222	Csas	-0.4561444	5.15632753	0.025607	0.87286385	1
FBgn026248	Rbp	0.45580132	5.49275666	0.02559518	0.87289295	1
FBgn026145	capt	-0.5378049	5.14597047	0.02557382	0.87294555	1
FBgn002419	robl	0.17821593	9.00149547	0.02542315	0.87331721	1
FBgn002873	Fmr1	-0.7456069	7.56082978	0.02530216	0.87361647	1
FBgn001077	Xe7	-0.3710992	6.35534313	0.02526383	0.87371141	1
FBgn028593	Rab1	0.21672358	7.7639213	0.02524283	0.87376349	1
FBgn000003	nAChRalpha1	-0.616294	5.13560814	0.02523864	0.87377388	1
FBgn004007	Trx-2	-0.289961	6.8748011	0.02515959	0.87397005	1
FBgn003901	HP1c	0.37510319	6.23591658	0.02511783	0.8740738	1
FBgn003524	CG17249	0.38815429	6.45681928	0.02511395	0.87408345	1
FBgn002620	mei-P26	-0.2419964	8.66331506	0.02506879	0.87419577	1
FBgn000405	uzip	-0.2522711	7.41254934	0.02497114	0.87443899	1
FBgn003406	Ufc1	-0.4411078	5.25556898	0.0249618	0.87446228	1
FBgn003410	prim	0.40292259	5.88701931	0.02485858	0.87471996	1
FBgn000140	egh	-0.2977753	6.77767378	0.02469336	0.87513357	1
FBgn003992	CG11155	-0.7358571	6.98568491	0.02456773	0.875449	1
FBgn006924	eca	0.3624016	6.19222912	0.02448659	0.87565318	1
FBgn003953	CG5611	0.42562761	5.47374057	0.02434268	0.87601615	1
FBgn003928	Bili	2.82949246	5.28600513	0.02421159	0.87634775	1
FBgn002994	CG1677	-2.0528776	5.66427336	0.02397711	0.87694317	1
FBgn003308	CG9410	-0.397569	6.1217826	0.0239514	0.87700864	1
FBgn026438	Ca-alpha1T	1.04893133	5.07983352	0.02340843	0.87839976	1
FBgn003474	Vps20	0.33343989	6.41406809	0.02338361	0.87846372	1
FBgn003135	Rim2	-0.4943459	4.33269511	0.0229759	0.87951977	1
FBgn003764	CG11975	-0.2995118	6.70518049	0.02283517	0.8798865	1
FBgn003023	CG15209	0.18273083	9.36512596	0.02277625	0.88004039	1
FBgn026273	eIF4H1	-0.5496976	5.95437816	0.02271269	0.88020663	1
FBgn003189	CG13784	-0.6424041	4.64275454	0.02270265	0.88023291	1
FBgn000294	nkd	0.55379216	3.96782277	0.02255588	0.88061775	1
FBgn003095	CG18259	0.42688774	5.86328244	0.022548	0.88063845	1
FBgn005136	dpr17	-1.0502137	3.66842975	0.02244566	0.8809076	1

FBgn003827	UQCR-C1	-0.170501	10.3691011	0.02239928	0.8810298	1
FBgn025913	glo	0.21999027	7.97392077	0.02235053	0.88115838	1
FBgn026786	Maf1	-0.2100809	7.69950598	0.02229912	0.88129411	1
FBgn003182	Daxx	2.9501665	3.41441129	0.02226671	0.88137978	1
FBgn003005	CG12772	-2.4494928	6.08472114	0.02216737	0.88164272	1
FBgn003816	Task6	-0.5057053	4.77429147	0.02215669	0.88167103	1
FBgn003391	CG8547	2.53566633	4.75315124	0.02213105	0.88173901	1
FBgn002986	ND-B16.6	0.1664717	8.89913871	0.02192999	0.8822735	1
FBgn000123	Hsp83	-0.1501325	10.8247006	0.02182993	0.88254042	1
FBgn000039	cv	0.63191972	4.5469559	0.0216954	0.8829003	1
FBgn002834	ATPsyndelta	0.16225164	9.2011188	0.02163447	0.88306367	1
FBgn001177	Irbp	2.60294424	3.44109723	0.02143409	0.88360257	1
FBgn003423	APC10	-0.5037542	4.19839458	0.02136731	0.88378276	1
FBgn000458	Rrp1	0.29647301	7.15742154	0.02117983	0.88429012	1
FBgn005000	GstT1	-2.7352042	5.29112061	0.02101954	0.88472573	1
FBgn002030	crol	-0.4695086	5.37879878	0.02095688	0.88489648	1
FBgn004020	kat80	2.69888165	5.12618189	0.02094709	0.88492318	1
FBgn005267	X11Lbeta	-2.2048685	5.17687983	0.02092786	0.88497566	1
FBgn005201	4E-T	-2.8440116	5.33233221	0.02091481	0.88501128	1
FBgn000013	ash2	-0.3943164	4.2391134	0.0207652	0.88542044	1
FBgn001066	Nup214	2.88442646	3.17775829	0.02075256	0.88545507	1
FBgn002855	x16	-0.5446627	5.6788477	0.02074496	0.88547591	1
FBgn028595	RpL19	0.16859679	8.67511149	0.02068372	0.8856439	1
FBgn000018	bic	0.19694191	7.80898123	0.02063471	0.88577854	1
FBgn026392	jvl	0.38650468	5.94291199	0.02060005	0.88587385	1
FBgn004075	CG17059	0.32564981	6.29326327	0.02044829	0.88629215	1
FBgn003854	pasi1	-2.5729462	3.92146977	0.02040525	0.88641105	1
FBgn001399	Calx	1.26765848	4.51297932	0.02013276	0.88716692	1
FBgn026748	Ptp36E	0.27303698	6.82645538	0.02007494	0.88732797	1
FBgn026725	Ggamma30A	-0.150869	10.4708116	0.01999851	0.88754124	1
FBgn028347	SF2	-0.3907571	6.82410817	0.01999228	0.88755864	1
FBgn004030	Jafrac2	0.39677811	6.79476652	0.01998364	0.88758277	1
FBgn003061	Rab3-GEF	-0.6273648	3.51340387	0.01988718	0.88785263	1
FBgn008543	CG34404	0.32768104	6.81968806	0.01985256	0.88794963	1
FBgn008522	CG34200	-0.2861033	6.58926039	0.01975606	0.88822051	1
FBgn000558	Calr	0.14602576	10.2123819	0.01969406	0.88839491	1
FBgn003394	CG12868	-0.3444975	5.98586551	0.01969072	0.8884043	1
FBgn026671	Bruce	-0.4822411	5.41781079	0.01968082	0.88843216	1
FBgn002847	Mtpalpha	-0.4285454	7.1804207	0.01963661	0.88855673	1
FBgn026271	Sap130	-2.3884262	2.99972084	0.01963354	0.8885654	1
FBgn026560	Ric	-0.1670002	8.38955497	0.0195818	0.88871136	1
FBgn003842	CG10311	-0.5438596	4.65796232	0.01955061	0.88879944	1
FBgn003542	CG17746	-2.600846	5.9379293	0.01945606	0.88906692	1
FBgn003990	lgs	0.28834656	6.5456756	0.01928658	0.88954804	1
FBgn001528	RpL22	0.21171404	7.74086908	0.01927561	0.88957924	1
FBgn026085	Bet1	0.38416893	5.25071356	0.019267	0.88960376	1

FBgn002868	Rpt5	-0.2573513	7.34923275	0.01920497	0.88978048	1
FBgn002752	Ublcp1	2.80427352	4.18252579	0.01920353	0.88978458	1
FBgn003288	Pomp	0.2888769	7.40571581	0.01916586	0.88989204	1
FBgn001330	Nmda1	-0.1934365	7.8903011	0.01909079	0.89010651	1
FBgn026000	Dys	2.58734856	3.42532143	0.01905719	0.89020265	1
FBgn002568	MFS18	0.29004218	6.56815126	0.01905634	0.8902051	1
FBgn003060	ND-B18	0.17225636	8.09535147	0.01887021	0.89073922	1
FBgn001582	TfIIEbeta	-0.2928426	6.46600991	0.01871088	0.89119861	1
FBgn026449	CG17646	0.42142855	5.35430321	0.01862087	0.891459	1
FBgn003105	CG14229	0.27716019	6.6071191	0.01857491	0.89159219	1
FBgn002608	Adar	-1.3964443	4.7031266	0.01853532	0.89170706	1
FBgn003223	mRpS7	0.2907396	6.55955526	0.01835435	0.89223378	1
FBgn001528	Rpn10	0.16067399	8.41378829	0.01826094	0.89250667	1
FBgn003554	DOR	0.31818523	6.66911327	0.01822532	0.89261094	1
FBgn003680	MYPT-75D	-0.7178944	4.2645303	0.01803284	0.8931761	1
FBgn000567	spi	0.27891928	7.50700148	0.01800234	0.89326593	1
FBgn001416	fax	0.21235052	7.38572816	0.01784989	0.89371614	1
FBgn005114	CG31140	0.29154606	6.41826324	0.01772911	0.89407419	1
FBgn026179	SmE	-0.2836515	6.57306327	0.01764188	0.89433358	1
FBgn026469	Mhc	0.44702124	4.06958303	0.01762956	0.89437026	1
FBgn025911	mmd	-0.4600599	6.14573475	0.01761147	0.89442414	1
FBgn003363	tou	0.65653194	4.59993233	0.01760522	0.89444277	1
FBgn000436	porin	-0.1363031	10.4625854	0.01758928	0.89449031	1
FBgn003405	Mlf	-0.1446794	9.22741974	0.0175796	0.89451915	1
FBgn028371	LIMK1	-2.0787752	2.17031249	0.01747135	0.89484254	1
FBgn000257	RpL36	0.14960461	9.16113184	0.01731061	0.89532459	1
FBgn026126	mgl	0.34290769	5.94069842	0.01728744	0.89539424	1
FBgn000002	Ace	-0.3947814	6.08080484	0.01726432	0.89546384	1
FBgn025923	CG42336	0.41758888	4.86424463	0.01723641	0.89554789	1
FBgn002952	CG13365	-0.2838392	6.43961958	0.0172176	0.89560457	1
FBgn001373	shot	-0.3732865	7.2428379	0.01709394	0.89597798	1
FBgn005206	CG32069	0.35772046	5.28569664	0.01707281	0.89604194	1
FBgn005115	Rpb7	0.55031065	5.35698353	0.01705387	0.89609929	1
FBgn003450	MED8	-2.5005153	4.6879722	0.01701859	0.8962062	1
FBgn003375	ClC-b	-0.2367221	7.44394257	0.01701184	0.89622668	1
FBgn003071	ND-20	-0.1485007	8.77722141	0.01692433	0.89649244	1
FBgn002992	CG14431	1.8419292	2.98034041	0.01691134	0.89653192	1
FBgn002618	Rok	0.46244353	2.73138541	0.01688234	0.89662018	1
FBgn003505	SiaT	-2.4329309	5.40754451	0.016844	0.89673697	1
FBgn003414	CG5065	-0.4872926	6.45153671	0.01684145	0.89674475	1
FBgn003572	Mis12	0.22115035	7.05487644	0.01681597	0.89682245	1
FBgn002671	Agpat2	-0.4326286	4.63828683	0.01678112	0.89692882	1
FBgn026179	SNRPG	-0.3978062	5.17271105	0.01674126	0.89705063	1
FBgn005804	CG40045	-0.1571716	8.32954085	0.01673093	0.89708221	1
FBgn005115	wge	1.00991362	4.45370337	0.01667641	0.8972491	1
FBgn026535	tn	2.15105371	2.85626231	0.0166582	0.89730492	1

FBgn025917	Nedd4	-2.5140253	3.27532016	0.01663209	0.89738499	1
FBgn025083	roh	0.14888194	8.8245357	0.01663182	0.89738581	1
FBgn002969	CG6379	0.56145927	6.1784501	0.01662012	0.89742171	1
FBgn002986	CG15891	0.25627448	6.63194517	0.01661072	0.89745057	1
FBgn003229	Dpy-30L1	0.2619158	6.59232303	0.01657493	0.89756049	1
FBgn003593	Tsp66E	-1.2193464	6.85300294	0.0165716	0.89757072	1
FBgn008635	Sec61alpha	-0.2211134	7.2706573	0.01651445	0.89774653	1
FBgn003598	Tat	-0.2121564	7.41212708	0.01643844	0.89798084	1
FBgn003886	Smyd5	0.40647105	4.96604333	0.01642592	0.89801947	1
FBgn026126	Cul3	-0.37352	5.63429966	0.01640181	0.89809393	1
FBgn003363	Prip	0.2717928	6.53835428	0.01636915	0.8981949	1
FBgn000486	hop	0.25958621	3.52476638	0.01629539	0.89842328	1
FBgn002492	Tnpo	-0.390099	5.52253342	0.01624103	0.89859191	1
FBgn003917	CG6356	0.42458857	4.10156805	0.01616193	0.89883785	1
FBgn005042	CG30428	-2.6322543	5.06619348	0.01599801	0.8993494	1
FBgn003567	CG13293	-2.501009	3.55282124	0.01579551	0.8999851	1
FBgn003932	CG10550	0.2306913	7.47702071	0.01576778	0.90007245	1
FBgn000023	bur	-0.4555577	5.24464896	0.01566931	0.90038333	1
FBgn026339	Uck	1.99815824	4.76983025	0.01560168	0.90059742	1
FBgn002756	CG18659	0.33519041	6.7921608	0.01524713	0.90172758	1
FBgn002990	pod1	0.41412598	4.48280395	0.01516816	0.90198111	1
FBgn003537	Non2	-0.165851	7.94374492	0.01507956	0.90226639	1
FBgn003723	Suv3	-2.2923416	5.18134578	0.01502368	0.90244672	1
FBgn003553	Cip4	2.79675611	4.87699885	0.01498492	0.90257202	1
FBgn002641	Idgf4	0.14569155	8.51970236	0.01492428	0.90276838	1
FBgn003775	CG8516	2.753012	3.4018973	0.01485894	0.90298041	1
FBgn002687	CG14777	0.2026718	7.12789436	0.01480386	0.9031595	1
FBgn008395	side-VI	-0.3368066	5.34253824	0.01467619	0.90357593	1
FBgn003764	RagA-B	0.29706298	6.1646341	0.0146656	0.90361057	1
FBgn008543	Not1	1.53794634	4.91397247	0.01460733	0.90380132	1
FBgn003868	Cyp12a5	-0.402095	4.6707428	0.01457942	0.90389281	1
FBgn008635	Tpi	0.1302577	10.2259469	0.01456012	0.90395614	1
FBgn003319	CG1358	-0.2570186	7.11358885	0.01455705	0.90396622	1
FBgn003393	CG17385	-0.176496	7.56545371	0.01446373	0.90427305	1
FBgn003600	Jarid2	-0.6677604	5.49876084	0.01444774	0.90432571	1
FBgn003969	Gnpnat	0.29950814	6.09132026	0.01440615	0.90446285	1
FBgn028424	Cyt-c-p	0.12556339	9.89526832	0.01433092	0.90471145	1
FBgn003008	AP-1gamma	0.33533864	5.32623366	0.01430861	0.9047853	1
FBgn003315	Dscam1	1.36567169	2.77025238	0.01427466	0.90489776	1
FBgn001486	Mlp84B	0.39535985	4.12283826	0.01411529	0.90542763	1
FBgn003748	MAGE	0.4146728	3.17706387	0.01396485	0.90593061	1
FBgn008645	su(r)	0.39614687	4.06628609	0.01396275	0.90593766	1
FBgn003293	CG8671	0.2470301	6.770731	0.01386188	0.90627646	1
FBgn026489	RapGAP1	-0.2972368	6.91871414	0.01385003	0.90631635	1
FBgn026408	Slob	-1.7125502	5.2434729	0.01384658	0.90632795	1
FBgn001121	eff	0.12824994	9.53676122	0.01383053	0.90638203	1

FBgn0033528	trsn	-0.2710741	6.37808031	0.01366214	0.90695106	1
FBgn000129	Jra	2.58370671	4.19291383	0.01361575	0.90710844	1
FBgn0035590	Prpk	0.25350704	6.41475895	0.01356743	0.90727269	1
FBgn0004924	Top1	-0.4098065	4.65752177	0.01356665	0.90727533	1
FBgn0023172	RhoGEF2	-1.1382335	3.50042231	0.01355845	0.90730325	1
FBgn0262473	Tl	-2.8473878	4.67306966	0.01350271	0.90749312	1
FBgn005209	CG32091	2.47764238	3.9182848	0.01342666	0.90775283	1
FBgn0050476	ave	-0.3086599	5.73996187	0.01336741	0.90795568	1
FBgn0039580	Gfat2	-0.9907433	6.11027702	0.01327775	0.90826354	1
FBgn0013718	nuf	-0.1761646	7.9073919	0.01317833	0.90860609	1
FBgn0034697	GM130	-0.2484533	6.44151017	0.01312219	0.90880013	1
FBgn0037670	Ibf1	-0.2696778	6.13866912	0.0131134	0.90883056	1
FBgn0027872	rdgBbeta	-0.287342	6.17080265	0.01299631	0.9092367	1
FBgn0003209	raw	-0.3517285	4.73216863	0.01299578	0.90923857	1
FBgn0013688	mt:srRNA	-0.1995681	6.95993063	0.01286552	0.90969263	1
FBgn0260995	dpr21	0.14874919	9.2848487	0.01283249	0.90980812	1
FBgn0040339	MED22	-0.2439308	6.43053561	0.01253121	0.91086868	1
FBgn0038295	Gyc88E	-1.3102859	3.55918533	0.01250556	0.91095959	1
FBgn0028482	bdl	0.22929487	7.04623253	0.01248673	0.91102637	1
FBgn0030723	dpr18	0.14653902	8.02983446	0.01246104	0.91111755	1
FBgn0011300	babo	-2.2295352	3.48261294	0.01243905	0.9111957	1
FBgn0261089	Sytalpha	-1.1212495	6.03760333	0.01238955	0.91137184	1
FBgn0030103	Obp8a	-0.3238927	5.26927274	0.01236584	0.91145634	1
FBgn0041773	tral	0.31115098	5.18624126	0.01230424	0.91167625	1
FBgn0038610	CG7675	2.71864942	4.41415796	0.01227487	0.91178129	1
FBgn0262467	Scox	-0.1492467	7.83557588	0.0122143	0.9119983	1
FBgn0053094	Synd	-1.1663235	5.55708903	0.01217849	0.9121269	1
FBgn0260817	gkt	-0.5078956	3.87867655	0.01212129	0.91233266	1
FBgn0032170	CG4658	0.24684212	6.64715085	0.01209768	0.91241774	1
FBgn0032020	strat	0.25438977	6.25609624	0.01206441	0.91253777	1
FBgn003868	Cyp12a4	-2.0503667	6.28231695	0.01205234	0.91258135	1
FBgn0034802	CNBP	0.19208189	6.97759975	0.01197507	0.91286091	1
FBgn0036510	CG7656	-0.3137471	6.53233795	0.01192442	0.91304465	1
FBgn0266064	GlyS	-2.5359173	4.41534213	0.01187182	0.91323588	1
FBgn0040233	c12.1	0.16383309	7.47477777	0.01186804	0.91324966	1
FBgn003595	CG5068	2.06171407	5.92111421	0.01186286	0.91326853	1
FBgn0004910	Eip63F-1	-0.8749373	4.6627548	0.01185322	0.91330364	1
FBgn0026252	msk	-0.4730739	4.76353116	0.01184902	0.91331894	1
FBgn0266129	lov	-0.5905402	5.32124063	0.01181606	0.91343908	1
FBgn0014464	Cp7Fa	2.50516916	3.32674441	0.01174817	0.91368715	1
FBgn0261622	GLS	-2.4028364	4.96165523	0.01172146	0.91378493	1
FBgn0000592	Est-6	-1.0408089	3.37795186	0.01171177	0.91382043	1
FBgn003089	Ada3	-0.3801065	3.30510222	0.01167245	0.91396466	1
FBgn0052529	Hers	0.67328598	4.70352399	0.01165366	0.91403368	1
FBgn0024432	Dlc90F	-0.2483854	6.20875523	0.01163852	0.91408931	1
FBgn0032979	Clamp	-0.201359	7.10926506	0.01157405	0.91432669	1

FBgn003318	Drat	-0.1384097	8.42987328	0.01155361	0.91440206	1
FBgn004717	CG32147	0.28909441	5.55825046	0.01150431	0.9145842	1
FBgn000054	ed	2.20182635	2.79532118	0.01148512	0.91465518	1
FBgn003918	CG6454	0.27657321	6.66971116	0.01141564	0.91491275	1
FBgn005396	CG33969	-2.0978914	5.63888165	0.01135772	0.91512806	1
FBgn003205	PrBP	0.20491886	6.80945003	0.01131203	0.9152983	1
FBgn003079	CG9125	0.27489856	6.16413735	0.01130493	0.91532479	1
FBgn004111	lilli	-2.2372846	4.94335069	0.01129342	0.91536775	1
FBgn002847	Hrd3	2.04644462	3.57721398	0.01125348	0.91551696	1
FBgn001669	Past1	0.14849858	7.98059628	0.0112519	0.91552286	1
FBgn003350	CG12909	2.3991486	3.48201654	0.01120067	0.91571469	1
FBgn003321	LRR	0.64084734	2.85648216	0.01118661	0.91576742	1
FBgn008637	Alg-2	0.16151546	7.43408024	0.01117072	0.91582704	1
FBgn003148	CG9643	0.29689186	5.59009368	0.01103113	0.91635265	1
FBgn003605	CG6707	0.14430119	7.79703638	0.01099206	0.91650039	1
FBgn003074	CG9921	0.17725081	7.07910606	0.01098735	0.91651819	1
FBgn000564	Eip63E	-2.3417503	4.51375552	0.01097322	0.91657169	1
FBgn002619	myo	-0.5974276	5.54488821	0.01097127	0.91657909	1
FBgn003080	DENR	-0.2769016	6.29500126	0.01096642	0.91659745	1
FBgn003250	CG6523	-0.2718814	6.47095474	0.01088614	0.91690219	1
FBgn001367	mt:ATPase8	-0.131971	8.07028742	0.0108171	0.91716515	1
FBgn004090	mRpL33	-0.2361446	6.26664631	0.01076085	0.91738005	1
FBgn005015	CG30154	-0.3653266	2.45059093	0.0105346	0.91825014	1
FBgn004162	Ku80	2.4324087	4.1851816	0.01050535	0.91836329	1
FBgn026569	lncRNA:CR44	0.35138688	3.48678837	0.01046713	0.91851142	1
FBgn003706	CG12975	0.12931584	8.0189365	0.01022705	0.91944814	1
FBgn003408	CG8435	0.26050907	5.89063876	0.01020765	0.91952432	1
FBgn003203	Wdr82	0.22666494	6.31583479	0.01016564	0.91968956	1
FBgn003720	CG12768	-2.1619948	2.9045322	0.01009812	0.91995581	1
FBgn003268	Ugt301D1	-0.3197301	4.90585969	0.00991284	0.92069107	1
FBgn003057	mRpS25	-0.1539994	7.3563943	0.00984305	0.92096983	1
FBgn001326	Fkbp39	0.15435328	7.41728294	0.00983854	0.9209879	1
FBgn001439	sun	-0.1134044	8.93673625	0.0097834	0.92120888	1
FBgn025970	CG42361	-0.1888737	6.76935734	0.00971995	0.92146396	1
FBgn026654	lncRNA:CR45	-0.3460776	3.74254588	0.00971392	0.92148824	1
FBgn004055	CG11686	0.45007128	4.68027859	0.00968667	0.92159808	1
FBgn003770	RnpS1	0.20226666	6.98271121	0.00964672	0.92175942	1
FBgn026257	Ect4	0.35339684	3.7948904	0.00964555	0.92176415	1
FBgn006762	CG33331	-1.9884565	5.32492345	0.00962863	0.92183257	1
FBgn002196	ND-PDSW	0.1047006	9.75219789	0.00961239	0.9218983	1
FBgn001374	Arf102F	0.12556539	8.16863847	0.00960726	0.92191909	1
FBgn001485	Hr38	-1.6956598	4.11743632	0.00959997	0.92194862	1
FBgn003360	CG13220	-0.2160796	6.49592095	0.00953933	0.92219474	1
FBgn003270	CG10338	-0.3063326	5.0867502	0.00953829	0.92219897	1
FBgn003326	udd	0.31483858	4.60034448	0.00951852	0.92227939	1
FBgn002353	CG17896	0.22140518	8.03696228	0.00943809	0.92260741	1

FBgn003457	ND-B14.7	-0.121307	8.25410402	0.00943726	0.92261081	1
FBgn026578	CrebB	-0.3977748	5.97738734	0.00931546	0.92311026	1
FBgn003407	Asph	2.57817349	4.05857103	0.00914185	0.92382793	1
FBgn003327	CG14757	-0.1347969	8.01718508	0.00910025	0.92400091	1
FBgn003681	CG14073	2.03419003	2.70125328	0.00907062	0.92412435	1
FBgn026685	lncRNA:CR45	1.95493176	2.54129201	0.0090247	0.92431608	1
FBgn026435	SNF4Agamm	0.27254812	6.93690377	0.00897107	0.92454061	1
FBgn003458	Rbpn-5	-1.6614852	4.46184679	0.00896517	0.92456535	1
FBgn026702	lncRNA:CR45	-2.487877	4.17033355	0.00886588	0.92498302	1
FBgn003887	CG3337	0.21593246	6.21535016	0.00880869	0.92522465	1
FBgn000109	Gapdh2	-0.0914596	11.4320839	0.00880575	0.92523711	1
FBgn026655	kis	-0.8780274	5.53125625	0.00877207	0.9253798	1
FBgn002574	PlexA	-1.8567644	4.83552657	0.00873166	0.92555137	1
FBgn004039	CG3655	2.18490631	4.01872217	0.00870847	0.92564999	1
FBgn003880	CG5412	0.2359595	6.04758668	0.00870575	0.92566156	1
FBgn026791	lncRNA:CR34	0.0908761	12.4125603	0.0087014	0.9256801	1
FBgn003878	CG4360	-1.2499877	4.35424936	0.00869444	0.92570973	1
FBgn003437	Ntan1	-0.2301894	6.09769534	0.00864639	0.9259147	1
FBgn003712	CG14567	-0.3287428	2.67935801	0.0085956	0.926132	1
FBgn000486	RpS2	0.09858253	10.3637644	0.00857764	0.926209	1
FBgn003814	CCHa2	2.33880664	3.96002088	0.00856084	0.9262811	1
FBgn003009	fh	0.21185612	6.39366708	0.0085573	0.92629627	1
FBgn000108	fy	2.30943431	3.83056005	0.00855326	0.92631365	1
FBgn000046	Dl	2.05876496	2.62632008	0.00852682	0.92642729	1
FBgn003859	CG14312	-0.2098616	6.26367008	0.00851964	0.92645817	1
FBgn026094	Rbp6	-0.2691857	7.98778377	0.00849629	0.92655875	1
FBgn001060	lwr	-0.1791421	6.91127005	0.00848748	0.92659672	1
FBgn026497	Nrg	0.30303503	5.40834147	0.0084573	0.92672697	1
FBgn002895	CG14478	0.21274373	6.19928874	0.00840699	0.92694465	1
FBgn002982	CG12236	2.16149083	4.55013737	0.00833032	0.92727757	1
FBgn005041	nord	1.90202062	2.60152017	0.00829018	0.92745251	1
FBgn003001	CG2278	-0.3274299	3.2725148	0.00824489	0.92765042	1
FBgn003760	mRpL19	-0.1795204	6.69292419	0.00823785	0.92768125	1
FBgn003783	TTLL15	2.25498669	5.67726744	0.0081856	0.92791032	1
FBgn000008	AnxB10	-0.1340753	7.53546722	0.00815515	0.92804415	1
FBgn000252	LanA	1.75607432	2.99970504	0.00814987	0.9280674	1
FBgn005223	ND-MLRQ	0.10099186	8.89288885	0.0080951	0.92830883	1
FBgn003183	CG11319	0.27410421	5.31127393	0.00807297	0.92840666	1
FBgn003263	CG15141	0.21924315	6.50994508	0.00806501	0.92844185	1
FBgn000271	Men	0.14407159	8.73003249	0.00792637	0.92905795	1
FBgn004092	CR15345	0.3783829	4.52749965	0.00792402	0.92906843	1
FBgn026285	CG43222	0.12475155	7.73625821	0.00774525	0.92987102	1
FBgn003347	oys	0.24994276	5.35849673	0.00773277	0.92992739	1
FBgn002030	dom	0.29084916	4.81287823	0.00771368	0.93001376	1
FBgn008306	CG33947	2.14476199	3.08656575	0.00770083	0.93007189	1
FBgn003194	CG7191	2.2555518	5.66390143	0.00766112	0.93025198	1

FBgn000316	Pu	0.21126602	6.09804021	0.00751288	0.93092834	1
FBgn026280	twr	0.24460669	6.0467173	0.00750525	0.93096336	1
FBgn003739	CG2017	0.18888817	6.49272678	0.00748828	0.93104126	1
FBgn003264	CG5110	-0.2464327	5.58219469	0.00747018	0.93112444	1
FBgn000218	dre4	1.90624283	2.83673316	0.00746167	0.93116359	1
FBgn003025	CG2145	1.98350204	4.95034168	0.00745175	0.93120925	1
FBgn026212	Sec23	0.11462665	7.84821914	0.00743943	0.93126598	1
FBgn002235	Sodh-2	0.26801708	4.6951215	0.00738704	0.93150784	1
FBgn005002	CG30022	2.21302363	3.73818596	0.00738611	0.93151216	1
FBgn003034	BORCS5	-0.1795184	6.64154813	0.00734068	0.93172258	1
FBgn002614	TTLL4A	-0.2280052	5.60078959	0.00731827	0.93182664	1
FBgn003774	Naa80	0.11227973	7.89597583	0.00729057	0.93195545	1
FBgn028425	Letm1	1.6907187	4.16590861	0.00723878	0.93219698	1
FBgn003810	Paip2	-1.7315468	5.31334816	0.0071771	0.93248579	1
FBgn002568	cry	1.86060061	5.32688339	0.00715793	0.93257577	1
FBgn008573	CG40472	0.16774672	6.6800216	0.00715594	0.93258515	1
FBgn003219	CG4908	-1.4884145	4.55447826	0.00711957	0.93275628	1
FBgn003715	olf413	0.87121565	4.3130106	0.00701581	0.93324692	1
FBgn003130	CG4552	-0.9120602	7.27287517	0.0069809	0.93341282	1
FBgn002276	Sin3A	-1.7148348	4.42817286	0.0069681	0.93347372	1
FBgn005037	CG30373	0.19172652	6.26356501	0.00694835	0.93356789	1
FBgn003083	CG8675	0.19980354	6.09476697	0.00693717	0.93362121	1
FBgn004051	zetaCOP	0.10530249	8.13057282	0.00691243	0.93373942	1
FBgn002637	Pten	1.83613813	5.63956822	0.0069017	0.93379075	1
FBgn025992	CG42450	-0.2676257	7.47964213	0.00682936	0.93413784	1
FBgn003968	CIA30	0.19992108	6.07691349	0.00682896	0.93413976	1
FBgn006367	CG40228	0.14359017	7.0773901	0.00682012	0.93418233	1
FBgn003784	CG4565	1.91645788	2.75682448	0.00677344	0.93440742	1
FBgn026680	CG45263	0.17088882	6.68582434	0.00676444	0.93445095	1
FBgn003251	ND-B22	0.08798564	9.50205582	0.00670678	0.93473025	1
FBgn026448	CG43897	0.27604969	3.92833171	0.00669059	0.93480893	1
FBgn003858	mTerf5	-0.274608	4.45473428	0.00666854	0.93491617	1
FBgn003597	UGP	0.11599811	7.98090389	0.006664	0.93493831	1
FBgn005001	CG30015	1.89470598	2.75385758	0.00661164	0.93519385	1
FBgn002030	dbe	0.22760986	6.32425776	0.00661007	0.93520154	1
FBgn003384	mip120	1.94973841	4.20382877	0.00659988	0.93525139	1
FBgn000439	unk	-0.8073935	3.27527188	0.00648916	0.93579562	1
FBgn003365	CG13192	-1.9128647	4.17808697	0.00645866	0.93594633	1
FBgn003961	DIP-gamma	-0.7542569	4.21409905	0.00644935	0.93599241	1
FBgn003503	ATPsynF	-0.0878419	9.31346637	0.00641934	0.9361412	1
FBgn003811	CG7966	-0.2841794	2.33866713	0.00639032	0.93628538	1
FBgn000252	Lam	0.16527914	6.65808188	0.0063138	0.93666721	1
FBgn026106	Rbsn-5	-2.2043513	4.28083519	0.00622527	0.93711188	1
FBgn003546	CG14977	-0.1751883	6.32403751	0.00615117	0.93748652	1
FBgn003089	Frq1	0.13554305	7.0959825	0.00614881	0.93749849	1
FBgn026297	lawc	0.15666399	6.71558437	0.00612588	0.93761489	1

FBgn000346	sog	-0.2688798	3.58502979	0.00608408	0.93782765	1
FBgn026161	nej	0.19297114	6.01653554	0.00606248	0.9379379	1
FBgn005049	Coq9	2.1023822	4.57682154	0.00604193	0.93804294	1
FBgn003078	mRpL22	0.22095155	6.31237039	0.00603126	0.93809755	1
FBgn003808	CG12279	-0.2079111	5.52501586	0.00602155	0.9381473	1
FBgn026251	Vha14-1	-0.0843352	9.43519965	0.00600573	0.93822848	1
FBgn026199	CG42813	-0.1347919	7.28526171	0.00600428	0.9382359	1
FBgn005270	CG32708	0.20813411	5.83594129	0.00599759	0.93827025	1
FBgn026387	sick	1.97000086	4.7591366	0.00597803	0.93837079	1
FBgn000463	Rap1	-0.1738779	6.59430988	0.00590062	0.93877035	1
FBgn003963	Pdhb	-0.1159756	7.45428238	0.00589098	0.93882026	1
FBgn002908	disp	1.60850341	3.58253711	0.00588513	0.93885058	1
FBgn003119	Cp110	-2.1029231	3.57963257	0.00588465	0.93885309	1
FBgn002616	tna	-0.1832389	6.54644224	0.00585897	0.93898641	1
FBgn003718	CG11367	2.11322462	4.26106002	0.00581513	0.93921466	1
FBgn000565	Ets65A	1.84463429	2.94923931	0.00574567	0.93957807	1
FBgn003605	CG10809	-1.8175782	4.55311185	0.00572057	0.93970995	1
FBgn003459	hng1	0.25453139	4.32743532	0.00564666	0.94009993	1
FBgn002870	Mt2	2.01550732	3.51361707	0.00559957	0.94034973	1
FBgn001019	RpS15Aa	-0.1056324	7.68252665	0.00556304	0.94054427	1
FBgn000293	nec	0.15193088	6.57914541	0.00550585	0.9408501	1
FBgn003716	CG11425	0.36016261	6.01250275	0.00548163	0.94098014	1
FBgn003305	SCAP	-0.987113	3.32189409	0.00546282	0.94108129	1
FBgn003368	Prp8	-0.6182595	6.71157351	0.005438	0.94121503	1
FBgn003796	Cad87A	1.64635231	2.92386083	0.00538479	0.94150282	1
FBgn000062	E(z)	2.00167326	5.3115974	0.0053681	0.94159338	1
FBgn026319	Patronin	1.59981855	3.8643335	0.00531521	0.94188133	1
FBgn005264	CG32647	0.19064769	5.7120864	0.00529959	0.94196662	1
FBgn003973	CG15525	0.27815489	5.43004946	0.00525553	0.94220796	1
FBgn000001	abd-A	-0.6404293	3.60808439	0.00522459	0.94237803	1
FBgn005244	CG32441	-0.0824798	8.98598055	0.00521241	0.9424451	1
FBgn003779	CG12817	-0.1951742	5.46314873	0.00518575	0.94259223	1
FBgn008545	Snoo	-0.2485528	4.74472628	0.00516845	0.94268794	1
FBgn003916	CG5515	0.13288652	7.07251742	0.0051662	0.94270037	1
FBgn000116	H	-1.1719655	4.68113883	0.00515287	0.94277422	1
FBgn026187	sdt	0.16316878	6.33518184	0.00512721	0.94291663	1
FBgn003755	Arl8	0.12851983	7.10303557	0.00512038	0.9429546	1
FBgn000309	Pkc53E	-0.2324004	5.16485941	0.00511158	0.94300358	1
FBgn003798	Spt3	0.14466075	6.68636662	0.00508926	0.94312794	1
FBgn002784	CAH1	0.25323633	3.81344708	0.00506379	0.9432702	1
FBgn025972	CG42376	0.12841484	6.88218111	0.00503401	0.94343698	1
FBgn026779	rump	-0.0980955	7.70930765	0.00501911	0.94352058	1
FBgn003170	CG7382	-0.1233189	7.47420687	0.00501824	0.94352549	1
FBgn003017	CG15312	-1.7717816	3.62232943	0.0049669	0.9438146	1
FBgn002296	vimar	1.88761671	4.26607147	0.00494245	0.94395285	1
FBgn003474	RpS16	0.09787063	7.69566892	0.00493929	0.94397072	1

FBgn002609	CG14812	0.13159571	6.80149046	0.00493117	0.94401672	1
FBgn003411	Nup62	0.15948738	6.28779509	0.00490604	0.94415936	1
FBgn003930	CG11858	-0.1868651	5.52780117	0.00490176	0.94418366	1
FBgn003688	CG9231	0.11875388	7.06680734	0.00486764	0.94437793	1
FBgn003825	smp-30	1.85865181	3.45021236	0.00475643	0.94501601	1
FBgn004384	vir-1	-0.0735647	9.93382968	0.00475392	0.94503045	1
FBgn003827	CG3817	-0.9870829	4.71760229	0.00474515	0.94508114	1
FBgn001524	Diap2	0.09581437	7.68589838	0.00471393	0.94526179	1
FBgn008542	DIP-delta	1.48405975	3.3696731	0.00470268	0.94532704	1
FBgn001052	Bka	0.1786708	5.79877242	0.00468366	0.94543757	1
FBgn003571	tow	-0.1789697	5.70087544	0.00466713	0.94553377	1
FBgn003240	Pex19	-0.0881612	7.91275557	0.00461585	0.94583337	1
FBgn002985	CG3566	0.09583915	7.63766389	0.00457464	0.94607535	1
FBgn003966	CG2006	-0.2363729	3.8428884	0.00456509	0.94613157	1
FBgn026738	Ubc7	0.11860843	7.19641894	0.00455739	0.94617695	1
FBgn005248	CG32485	0.08438155	8.11868281	0.00455407	0.94619651	1
FBgn006710	GlcT	2.08454261	3.65357173	0.00454825	0.94623086	1
FBgn001123	poe	2.09733398	5.09309933	0.00450781	0.94647008	1
FBgn002663	ldlCp	-0.2081791	5.1282956	0.00449622	0.94653881	1
FBgn003458	CG10527	0.11586853	7.21633494	0.00449091	0.94657034	1
FBgn000316	pum	-0.1021641	7.5678668	0.00445475	0.94678557	1
FBgn002351	Rbcn-3B	0.17684415	5.74379742	0.00442369	0.94697116	1
FBgn003819	Npc2b	-0.1855649	5.50329488	0.00437019	0.94729233	1
FBgn003258	Tpr2	-0.0986263	7.56915716	0.004346	0.94743815	1
FBgn003509	CG9380	1.29282549	2.19634876	0.00432096	0.94758958	1
FBgn003920	CG13623	0.10811458	7.14162767	0.00430915	0.94766115	1
FBgn001172	twin	-0.3926878	5.01190927	0.00430376	0.94769387	1
FBgn003387	Syngr	-0.1344377	6.83389856	0.00428276	0.94782147	1
FBgn003217	CG5850	1.43862844	2.54566644	0.00427876	0.94784579	1
FBgn005172	Trim9	1.66569691	4.92779755	0.00426762	0.94791363	1
FBgn005397	CG33977	-0.1160153	6.93073399	0.00426446	0.94793289	1
FBgn028689	Tango1	-1.4005884	2.67448091	0.00424864	0.94802941	1
FBgn003655	CG17029	0.10133371	7.49111376	0.00418952	0.94839176	1
FBgn003276	CG17568	1.60891396	4.22299436	0.00418247	0.94843513	1
FBgn003126	Spp	0.10345084	7.19363156	0.00416726	0.94852884	1
FBgn005145	mRpS18A	0.07928538	8.27561636	0.00413895	0.94870375	1
FBgn003953	Tusp	1.54665183	3.30528595	0.0041366	0.94871832	1
FBgn002886	CG4587	1.2929805	5.22734681	0.00413393	0.94873481	1
FBgn026074	CG3281	-1.1364844	5.20371998	0.00408923	0.94901234	1
FBgn005311	Rtnl1	0.06892378	9.79176793	0.00407819	0.94908112	1
FBgn001579	Rab7	0.06796997	9.52545494	0.00402954	0.94938538	1
FBgn000532	nmd	0.08806853	7.81901137	0.00400857	0.94951703	1
FBgn026095	MCPH1	1.45484963	4.34560821	0.00399783	0.94958462	1
FBgn003731	CG1161	0.07522141	8.41928822	0.00399482	0.94960357	1
FBgn003284	Pyroxd1	-0.154114	6.50173506	0.00397813	0.94970887	1
FBgn003252	Hacd2	-0.1123594	6.91701703	0.00397085	0.94975479	1

FBgn003386	CG18368	-1.3477879	2.96872687	0.00396639	0.949783	1
FBgn003842	CG17931	0.16898223	5.56259344	0.00394387	0.9499256	1
FBgn000388	tub	1.3028735	3.37012915	0.00393541	0.94997925	1
FBgn003272	Nedd8	-0.1449343	6.19663128	0.00391649	0.95009945	1
FBgn026384	CG43707	1.25801195	4.05247529	0.00391515	0.95010802	1
FBgn000389	betaTub97EF	2.1997631	5.62021229	0.00390644	0.95016347	1
FBgn008322	Frq2	-1.6020989	4.49865446	0.00390173	0.95019346	1
FBgn000320	ras	-0.1379294	6.74660174	0.00386701	0.95041527	1
FBgn005199	CG31997	-0.157927	6.08339306	0.00385815	0.95047208	1
FBgn002832	l(1)G0320	0.09948076	7.22750148	0.00385767	0.95047513	1
FBgn002708	GlyRS	0.10334393	7.17143349	0.00384419	0.95056161	1
FBgn002049	emb	0.21595522	3.93406264	0.00384023	0.95058707	1
FBgn026182	Bsg	0.09715554	7.66853587	0.00382487	0.95068588	1
FBgn000375	trk	1.96937473	3.67819096	0.00381803	0.95072991	1
FBgn003896	Nop56	0.11660061	6.81564509	0.00380244	0.9508305	1
FBgn003853	alt	0.9637503	4.24783527	0.00375879	0.95111316	1
FBgn001182	Pxn	0.23382901	6.86025524	0.0037374	0.95125228	1
FBgn026311	Mitf	1.16583247	4.87822504	0.00372302	0.95134603	1
FBgn005312	NLaz	-1.2363087	5.81540952	0.00371167	0.95142014	1
FBgn002176	chb	1.83160155	5.11003278	0.00370913	0.95143678	1
FBgn001487	Roe1	-0.104688	7.00313422	0.00366374	0.95173444	1
FBgn002981	CG12239	-0.0837922	7.7048225	0.00364197	0.95187792	1
FBgn000443	Ubc6	0.0850545	7.94833866	0.00362318	0.95200207	1
FBgn000228	Prosbeta6	0.10509542	6.96056128	0.0035849	0.95225596	1
FBgn003767	ATP6AP2	-0.0625502	10.417348	0.00355172	0.95247719	1
FBgn003248	Pect	0.1133232	7.70831384	0.00352498	0.95265618	1
FBgn003672	QIL1	0.06871447	8.61159847	0.003506	0.95278369	1
FBgn026510	Sgt1	0.13804615	6.41049882	0.00350258	0.95280666	1
FBgn003149	GABPI	0.07830461	7.93395169	0.0034897	0.95289344	1
FBgn028345	br	-0.364843	4.2200803	0.00348426	0.95293013	1
FBgn002473	PRL-1	-0.1670285	5.19976229	0.00347668	0.95298125	1
FBgn003461	MFS16	1.66147947	5.81124293	0.00346155	0.95308361	1
FBgn026490	pHCl-1	1.46332964	4.57323876	0.00343977	0.95323124	1
FBgn003997	CG17454	-0.103512	6.94636231	0.00343388	0.95327128	1
FBgn003992	Cals	0.07877546	9.0504203	0.00342518	0.9533304	1
FBgn004611	Gclm	0.16609747	6.00502424	0.00341185	0.95342121	1
FBgn003233	AstC	2.27603214	8.18177355	0.00340335	0.95347923	1
FBgn003335	CG8229	-1.9942543	3.92173189	0.00334405	0.9538858	1
FBgn003990	Asator	-0.4510694	5.39124756	0.00334305	0.95389274	1
FBgn002997	snz	0.81463733	3.90300285	0.00333403	0.9539549	1
FBgn003487	pita	-0.6959926	4.23458183	0.00331635	0.95407702	1
FBgn003078	Rrp45	1.69417051	3.78831097	0.00331585	0.95408044	1
FBgn003220	CG5676	0.07316365	8.00204993	0.00326688	0.95442044	1
FBgn026074	CG12213	-0.0899236	7.25912502	0.00318102	0.95502276	1
FBgn003298	CG3262	0.07743031	7.74196246	0.0031701	0.95509993	1
FBgn003309	CG3270	0.1212041	6.49848931	0.00315144	0.9552321	1

FBgn026192	CG42795	-0.2327692	4.36267021	0.00313632	0.95533952	1
FBgn005111	RabX4	-0.1253965	6.48383356	0.00312681	0.95540723	1
FBgn001234	Dh44	1.68469914	4.63999595	0.00312357	0.95543032	1
FBgn005115	Efa6	-0.2942705	4.61280047	0.00311818	0.95546876	1
FBgn006992	CG41128	-0.0723359	7.93165405	0.00310518	0.95556157	1
FBgn026637	Pde8	1.56293061	2.88483061	0.00310301	0.95557709	1
FBgn025974	wech	0.2282341	4.71467383	0.00309861	0.95560854	1
FBgn002864	mRpL50	-0.1432314	5.78255497	0.00308822	0.95568295	1
FBgn000331	S	-1.2180863	3.16480896	0.00308004	0.95574161	1
FBgn003169	CG14024	1.42150866	4.45176924	0.00305978	0.95588729	1
FBgn003128	Saf6	1.02776035	3.75685952	0.00305758	0.95590317	1
FBgn000482	His3.3B	-0.0556453	10.8642221	0.00305351	0.95593249	1
FBgn000561	msl-2	1.51490957	4.51010243	0.00304316	0.95600717	1
FBgn026641	CG45050	-0.1194219	6.46186325	0.00302157	0.95616328	1
FBgn003491	Pym	0.18815117	4.1358937	0.00300165	0.95630791	1
FBgn003281	CG10631	1.59323986	3.05346317	0.00299686	0.95634275	1
FBgn026500	CG44153	0.81070666	2.83031084	0.00298594	0.95642232	1
FBgn005039	CG30392	1.57462532	5.09721493	0.00296035	0.95660923	1
FBgn003284	mRpS18B	-1.2015713	3.21272357	0.00294775	0.95670159	1
FBgn003421	Camp	1.06719546	2.83792236	0.00292827	0.95684472	1
FBgn026097	CG42594	0.91792643	3.43486058	0.00291114	0.95697105	1
FBgn025974	CG42360	1.16322997	4.12760483	0.00290995	0.95697983	1
FBgn003941	CG12290	-0.4246685	3.66246773	0.0027878	0.95789157	1
FBgn003705	CG10565	-1.4380121	5.23674199	0.00278707	0.95789707	1
FBgn026478	Hph	0.20757322	6.52158499	0.00276934	0.95803109	1
FBgn003913	CG5902	-0.9016638	5.77611647	0.00276575	0.95805829	1
FBgn003239	CG6766	-1.1409999	6.14207158	0.0027655	0.95806013	1
FBgn001120	bol	0.83624212	5.96723512	0.00275091	0.95817081	1
FBgn003234	Reps	1.40111958	6.42820455	0.00274763	0.95819579	1
FBgn003794	CG14722	0.10973226	7.04718973	0.00270849	0.95849433	1
FBgn000410	Pp1-87B	-0.0629461	8.35864985	0.00270522	0.95851933	1
FBgn004451	mRpS5	0.06528203	8.12170107	0.0026927	0.95861537	1
FBgn026456	Hr4	0.30234698	4.24927021	0.00268952	0.95863975	1
FBgn026513	Spn42Da	-0.1735169	4.50981957	0.00267844	0.95872502	1
FBgn003726	MED31	-0.1095566	6.49073442	0.00263733	0.95904271	1
FBgn004072	dpr1	-1.3479479	3.1727873	0.00263679	0.95904685	1
FBgn000243	hyd	1.07242055	3.37382748	0.00263376	0.95907038	1
FBgn003447	Toll-7	-0.4851125	3.68117654	0.00261893	0.9591857	1
FBgn002580	Sbf	1.04668082	3.17452231	0.00261109	0.95924675	1
FBgn026155	CG42673	1.77883643	3.85078888	0.00259305	0.95938766	1
FBgn003737	Hat1	0.11629858	6.25103075	0.00259023	0.95940977	1
FBgn025078	Chd1	-0.2274449	4.09242497	0.00256055	0.95964281	1
FBgn004022	eIF3d1	-0.1283612	5.86759901	0.0025205	0.95995934	1
FBgn008668	Spf45	0.18420886	5.56916884	0.00251622	0.95999336	1
FBgn002027	lig	-0.3724372	4.62078085	0.00250332	0.96009593	1
FBgn005314	Hs3st-A	0.14154701	5.91007033	0.0024943	0.96016787	1

FBgn008541	dpr12	1.31425788	5.07407024	0.00249074	0.96019623	1
FBgn003078	Sap30	1.29971902	3.95103621	0.00245652	0.9604704	1
FBgn005233	CG32333	1.30678017	4.62581715	0.0024379	0.96062038	1
FBgn003726	spartin	1.25233628	4.84846857	0.00241611	0.96079664	1
FBgn002580	Smox	0.07449574	7.75531667	0.00233908	0.96142616	1
FBgn005317	beat-IIIb	-0.181134	6.01586129	0.00233572	0.96145385	1
FBgn003852	sll	1.41913985	4.39365916	0.00233286	0.96147742	1
FBgn003205	Uba4	0.98277289	3.41628194	0.00231482	0.96162655	1
FBgn001537	dod	-0.0906224	6.79659716	0.00231433	0.96163055	1
FBgn002913	REG	0.06251761	7.90701933	0.00231243	0.96164633	1
FBgn005206	Adi1	-0.1189392	5.9648114	0.00231005	0.96166608	1
FBgn003539	CG11486	-0.1006582	6.68700169	0.00227137	0.96198808	1
FBgn001560	BEAF-32	0.08024007	7.11617369	0.00225388	0.96213463	1
FBgn026096	pic	-0.1516138	4.06089367	0.00221908	0.96242783	1
FBgn004336	bchs	0.97980991	3.14040065	0.00219436	0.96263758	1
FBgn003468	CG2921	0.90270584	4.55350445	0.00218899	0.96268324	1
FBgn003152	CG9662	-0.1120411	6.1999622	0.0021838	0.96272753	1
FBgn005178	lncRNA:CR31	-1.2031753	5.01565269	0.00215698	0.96295694	1
FBgn003363	CG9005	-0.1306796	6.28324968	0.00207098	0.96370239	1
FBgn008527	CG34242	-0.1276259	5.24084035	0.00206732	0.96373445	1
FBgn003004	Trf4-1	-0.5059923	3.9933595	0.00205952	0.96380287	1
FBgn002049	Dad	1.21772204	3.83519115	0.00204201	0.96395698	1
FBgn002709	GlnRS	1.12691859	4.85205082	0.00203937	0.96398031	1
FBgn026609	lncRNA:CR44	-0.1101328	7.26075988	0.00201982	0.96415321	1
FBgn003215	nAChRalpha6	-0.0941898	7.29997143	0.00197413	0.96456073	1
FBgn006244	Cisd2	0.05622678	8.08778627	0.00189992	0.96523277	1
FBgn003033	CG9360	0.07735544	7.15250617	0.00188723	0.96534896	1
FBgn001148	Ssdp	0.0671115	7.33656116	0.00182321	0.96594139	1
FBgn003847	mRpS11	0.08471654	6.85479636	0.0018077	0.9660865	1
FBgn026517	CG44242	0.05545496	7.86494775	0.0017571	0.96656426	1
FBgn003373	CG8520	0.24561677	6.59475714	0.00174727	0.96665788	1
FBgn003322	CG12822	-0.0559115	7.83496886	0.00174496	0.96667992	1
FBgn003463	CG10433	-0.0456955	9.2918638	0.00173097	0.96681366	1
FBgn026500	koi	1.08400661	4.94579317	0.0017225	0.96689488	1
FBgn003291	CG9265	1.09092076	4.99688431	0.0017164	0.9669535	1
FBgn003633	MICAL-like	0.66005294	3.71516302	0.00170611	0.96705268	1
FBgn001031	corto	0.62610278	4.35284806	0.00169138	0.96719515	1
FBgn003625	CG5645	1.41618852	4.43392727	0.00168064	0.96729944	1
FBgn002760	Ulp1	0.85478022	3.32429475	0.0016631	0.96747042	1
FBgn003470	CG11275	0.83823913	2.82308609	0.00162592	0.96783585	1
FBgn003438	Mctp	-1.0835925	4.36764134	0.00162371	0.96785772	1
FBgn001578	Rab10	-0.2782823	5.94659463	0.00161796	0.96791463	1
FBgn003325	CG12769	0.70290965	4.01880165	0.00161686	0.96792554	1
FBgn003283	COX4	0.0416696	10.329922	0.00160252	0.96806799	1
FBgn003460	Ugt49C1	-1.567605	4.96521709	0.00156835	0.96841011	1
FBgn004001	Slmap	1.13628082	3.90112128	0.00156241	0.96846992	1

FBgn003390	CG18327	-0.1352467	4.14315956	0.00154746	0.96862108	1
FBgn000374	trc	0.97043361	5.15158503	0.0015351	0.9687466	1
FBgn025917	alpha-Man-Ia	1.15444205	5.53319768	0.00153222	0.9687759	1
FBgn002580	Rad17	-0.8435192	4.8597884	0.00153185	0.96877967	1
FBgn000108	g	0.57085511	3.78778019	0.00151746	0.96892656	1
FBgn002536	lp259	0.05728173	7.67814909	0.00151211	0.96898144	1
FBgn002076	Atet	-0.1905671	4.60054554	0.00150807	0.96902284	1
FBgn000125	ImpL2	-0.0887176	7.0059499	0.00150661	0.96903781	1
FBgn000199	crp	-0.3199906	5.60933652	0.00149368	0.96917089	1
FBgn003774	CG8412	1.3746514	4.5058351	0.00148374	0.96927356	1
FBgn003131	CG14341	0.08293273	6.42433483	0.00146717	0.96944561	1
FBgn001611	ATPsynCF6	-0.0428622	9.00923928	0.00146355	0.96948323	1
FBgn026637	lncRNA:CR45	0.1755738	3.46886839	0.00145627	0.96955927	1
FBgn003493	thoc5	1.59523734	4.34604648	0.00142687	0.9698679	1
FBgn003730	CG1113	1.33523919	4.46218626	0.00141688	0.96997356	1
FBgn003125	shv	0.08407161	6.44435341	0.00138867	0.97027388	1
FBgn001964	ATPsynB	-0.0388564	10.1327674	0.00137902	0.97037721	1
FBgn003842	CG14881	1.00745408	4.06145615	0.00136987	0.97047564	1
FBgn002993	CG12541	-1.0542623	4.27543021	0.00136945	0.97048014	1
FBgn002619	par-6	0.29185566	5.68377228	0.00136582	0.97051925	1
FBgn001741	ari-1	-0.0702751	7.00521764	0.00136056	0.97057614	1
FBgn002854	ics	1.20835241	4.24166738	0.001329	0.97091923	1
FBgn026667	Sec6	0.84971509	3.12572548	0.0013208	0.97100904	1
FBgn005310	CG33108	0.1105609	5.30017031	0.00132072	0.97100988	1
FBgn002481	Crk	0.04140846	8.59114342	0.00127373	0.97153005	1
FBgn002759	CG2790	0.06377691	6.90488674	0.00126959	0.97157634	1
FBgn003201	Scgalpha	-1.3488612	5.21866842	0.00124579	0.97184388	1
FBgn002023	ATPsyngamn	-0.0367747	10.3420858	0.00124471	0.97185614	1
FBgn008669	Plp	0.18248222	3.17074582	0.00121632	0.97217885	1
FBgn003868	CG5555	-0.0730785	6.60354797	0.00120907	0.97226179	1
FBgn026128	Ppcs	1.21517472	3.45178869	0.00120085	0.97235618	1
FBgn003613	CG7638	-0.064478	6.95695202	0.00119988	0.97236735	1
FBgn003640	CG6650	0.06947532	6.70035246	0.00117415	0.97266514	1
FBgn003942	CG6154	-0.0849956	5.86817056	0.00110091	0.97353114	1
FBgn003025	CG1545	0.06022841	6.87590646	0.00109105	0.97364983	1
FBgn002495	Mat1	-0.0598746	6.95774186	0.00107154	0.9738864	1
FBgn005164	CG31648	-0.1137292	4.07394829	0.00106303	0.97399025	1
FBgn003883	Ktl	-0.0440163	7.85568059	0.00106281	0.973993	1
FBgn004336	cbt	0.62093338	3.86284534	0.00105301	0.97411316	1
FBgn026321	Dscam4	-0.1734292	3.84579956	0.00103166	0.97437683	1
FBgn002625	eIF1A	0.0387949	8.49905969	0.0010112	0.97463206	1
FBgn028651	aqz	-0.0337914	10.7479865	0.00100945	0.97465406	1
FBgn003063	CG12608	-1.1098586	4.01459058	0.00100938	0.97465494	1
FBgn003824	CG14853	0.06260795	6.78065341	0.00099508	0.97483504	1
FBgn004403	mRpS14	-0.0546774	6.99881408	0.00098934	0.97490765	1
FBgn002632	Taf10b	-0.0475188	7.41855647	0.00098465	0.97496713	1

FBgn003771	CG9399	0.31652509	3.31069022	0.00097846	0.97504596	1
FBgn002951	Aos1	0.06092868	6.75073313	0.00097354	0.97510874	1
FBgn003062	CG9123	-0.0499338	7.22866088	0.0009726	0.97512074	1
FBgn000342	slo	-0.0667214	7.22111716	0.00096941	0.97516157	1
FBgn002641	Hsc70Cb	0.37404629	5.9216857	0.00095454	0.9753528	1
FBgn003619	Bmcp	0.06369722	6.63455626	0.0009439	0.9754905	1
FBgn000001	Abl	-0.0941013	4.72215198	0.00092517	0.97573477	1
FBgn005242	shep	-0.0393318	9.53932859	0.00092468	0.97574121	1
FBgn003639	upSET	0.25021808	4.81425274	0.00092401	0.97575007	1
FBgn003856	CG7785	-0.0635275	6.51298723	0.00091051	0.97592783	1
FBgn002753	beta4GalNA	0.91126104	4.66868069	0.00090268	0.97603142	1
FBgn002889	CG4935	-1.1656635	4.32729958	0.00087231	0.97643793	1
FBgn008543	NaCP60E	-0.0516338	6.96790354	0.00086171	0.97658154	1
FBgn001527	Pi3K59F	-0.6674233	4.54989875	0.00085809	0.97663084	1
FBgn003440	CG15084	0.1016383	3.95942705	0.00085598	0.97665953	1
FBgn026328	scrib	0.10522624	5.49029945	0.00085312	0.9766985	1
FBgn003683	CG3808	1.39967292	4.33569322	0.00085312	0.97669856	1
FBgn002574	mbt	0.78811653	4.00495466	0.0008323	0.97698454	1
FBgn008532	CG34293	-0.0815401	5.28821768	0.00082886	0.97703211	1
FBgn002494	NTPase	-0.0549345	6.97973505	0.00081687	0.97719879	1
FBgn002189	ytr	-0.0454205	7.199298	0.00080653	0.97734351	1
FBgn008667	Tpst	-0.4154259	4.04441508	0.00079561	0.97749738	1
FBgn003187	Gas41	-0.061607	6.37511344	0.00078741	0.97761364	1
FBgn003526	CG8001	1.06711968	4.47513718	0.00078168	0.97769525	1
FBgn003408	lbk	0.62923197	3.28195868	0.00077531	0.97778631	1
FBgn003495	CG3907	0.0377094	7.80185932	0.00077462	0.97779618	1
FBgn003195	cdc14	0.75790667	3.55738119	0.00076852	0.97788368	1
FBgn002989	CG14439	0.61873442	2.85003338	0.00074456	0.97823119	1
FBgn002758	CG1688	-0.152774	3.66179779	0.00074118	0.97828061	1
FBgn028594	RpL31	0.03325152	8.78447719	0.00073662	0.9783475	1
FBgn001178	BRWD3	1.18799547	5.34995458	0.0007325	0.97840806	1
FBgn003553	CG15014	0.61102797	3.84353075	0.00073157	0.97842189	1
FBgn025915	OtopLc	-0.4059719	4.5148059	0.00072193	0.9785644	1
FBgn028346	Vps4	0.03304389	8.16309977	0.00071211	0.97871064	1
FBgn003968	CG7582	-0.05671	6.4839968	0.00070161	0.97886821	1
FBgn003987	CG2126	-0.3009131	4.39565733	0.0007005	0.97888494	1
FBgn003464	ND-B12	0.03524204	7.79469341	0.00068888	0.9790607	1
FBgn026211	RNASEK	0.02708928	10.3923826	0.00068685	0.97909161	1
FBgn003136	CG17652	2.11961175	5.49511041	0.00068397	0.97913549	1
FBgn025078	alpha-Spec	0.03911146	7.5366676	0.0006729	0.97930501	1
FBgn001080	Chchd3	-0.0300948	8.4789254	0.00065798	0.97953564	1
FBgn003699	Hpd	1.94786249	4.74969014	0.00065658	0.97955741	1
FBgn003003	CG12123	0.05549508	6.54044927	0.00065337	0.97960742	1
FBgn001502	CkIIalpha-i1	-0.7630799	5.46948725	0.00064583	0.97972535	1
FBgn003083	CG8915	0.72930594	2.88923915	0.00064358	0.97976076	1
FBgn002988	CG3224	0.0567663	6.33693833	0.00063212	0.97994168	1

FBgn003381	Qsox1	0.06791927	6.53887301	0.00063016	0.97997285	1
FBgn028352	l(2)10685	1.21498688	4.04322653	0.00062878	0.97999479	1
FBgn026427	Sema2b	0.65060606	3.50055424	0.00061364	0.98023705	1
FBgn003093	Ggt-1	-0.3488388	4.07356758	0.00061064	0.98028539	1
FBgn002899	scro	1.38783742	4.58643979	0.00060151	0.98043328	1
FBgn003445	Oseg6	0.08595719	3.0210831	0.00059923	0.98047043	1
FBgn004038	mRpL14	0.04069445	7.06048339	0.00057292	0.98090385	1
FBgn003234	Csl4	0.05282528	6.35777857	0.00056531	0.98103103	1
FBgn005843	CG40439	-0.0819762	3.58177274	0.00056004	0.98111976	1
FBgn003700	ND-39	-0.024932	9.97480978	0.00054098	0.98144364	1
FBgn001559	AstA	-0.1251224	5.27433314	0.00053526	0.98154202	1
FBgn003523	CG7879	0.08575205	4.57243266	0.00051464	0.98190103	1
FBgn026450	CG43902	0.06022557	5.84019517	0.00051304	0.98192923	1
FBgn001368	mt:ND5	0.02455237	9.36337404	0.00050771	0.9820233	1
FBgn005266	Drak	-0.7238442	3.58333393	0.00050163	0.9821312	1
FBgn002499	CG2680	0.04225542	6.74546382	0.0004686	0.98272949	1
FBgn000386	trx	0.06710453	4.90293054	0.00046756	0.98274854	1
FBgn005327	Urm1	0.06708581	4.41247635	0.00044191	0.98322834	1
FBgn003508	Tina-1	-0.0376142	6.9073087	0.00043351	0.98338847	1
FBgn025968	Jabba	0.16537731	6.46049765	0.00043036	0.98344896	1
FBgn008537	Pde11	0.47046579	3.9751026	0.00041853	0.98367802	1
FBgn003222	CG5045	0.04031913	6.66293531	0.0004154	0.9837391	1
FBgn000535	Su(fu)	-0.0623742	5.23807939	0.00041014	0.9838425	1
FBgn003264	CG5131	0.5351622	5.00920332	0.00040947	0.98385563	1
FBgn002888	CG4168	0.47293415	2.90069355	0.00040271	0.98398935	1
FBgn003684	CNPYb	-0.0405606	6.6088775	0.00039341	0.98417544	1
FBgn006244	CG13197	0.82226918	4.77203981	0.00037586	0.98453232	1
FBgn003102	CG14200	-0.577133	3.77595869	0.00037216	0.98460872	1
FBgn003709	CG7611	0.06177482	5.18341961	0.00037127	0.98462701	1
FBgn008394	side-III	-0.338252	3.47630193	0.00036599	0.98473673	1
FBgn000061	e(y)2	-0.0617495	4.75400796	0.00036589	0.98473875	1
FBgn003846	AdSL	0.72418197	4.79481164	0.00035886	0.98488607	1
FBgn008660	CG9853	-0.0300742	7.39514271	0.00035424	0.98498363	1
FBgn001039	LamC	0.27475772	3.48736377	0.00033678	0.98535831	1
FBgn003275	CG10702	1.14088224	4.24097036	0.00033418	0.98541507	1
FBgn025973	mtgo	0.05055222	5.47398025	0.00032767	0.98555776	1
FBgn005193	CG31935	0.6562861	3.1781374	0.00031825	0.98576691	1
FBgn003463	twz	1.09860499	4.1631503	0.0003122	0.9859028	1
FBgn001552	peng	0.78504865	5.42540494	0.00030819	0.98599351	1
FBgn026164	Axud1	0.33756294	4.73303294	0.00030061	0.98616698	1
FBgn003478	CG13532	-0.0916807	4.36738434	0.00029236	0.98635801	1
FBgn001554	spag	-0.4270001	4.82028604	0.0002886	0.98644605	1
FBgn026171	nocte	-0.1716668	5.18763658	0.00028615	0.98650377	1
FBgn005244	Atox1	0.0300105	7.34126364	0.00028288	0.98658103	1
FBgn003925	CG11771	1.44701755	5.19774288	0.00028233	0.98659408	1
FBgn003768	pasi2	-0.0871428	4.45917888	0.00027794	0.98669877	1

FBgn003168	Cyp28d1	-0.0453698	5.02264476	0.00024402	0.98753667	1
FBgn004172	rho-5	-0.0456137	6.53431568	0.00024261	0.98757272	1
FBgn001679	dos	0.5261252	3.76178623	0.00024005	0.98763856	1
FBgn008302	snoRNA:Psi1	0.05169263	4.09306029	0.00023832	0.987683	1
FBgn004075	Prosap	0.04022828	5.90731821	0.00023721	0.98771166	1
FBgn005130	CG31301	-0.0342762	6.33441623	0.00022916	0.98792213	1
FBgn002617	siz	0.10045339	3.06073495	0.00022294	0.9880871	1
FBgn026273	Vha16-1	-0.0147542	10.9952608	0.00022223	0.98810614	1
FBgn003600	CG3434	-0.4487219	4.26606548	0.00021378	0.9883343	1
FBgn008519	CG34163	-0.0552807	4.03667919	0.00021014	0.98843412	1
FBgn003518	CG9205	-0.0238558	7.18630006	0.00019834	0.98876358	1
FBgn003935	CG5028	0.01627726	8.53707193	0.00019252	0.98892964	1
FBgn001062	DCTN3-p24	-0.0276777	6.66141563	0.00019039	0.98899103	1
FBgn003996	Fis1	-0.0149464	9.30124539	0.0001879	0.98906328	1
FBgn003030	Cyp4g15	-0.0232332	7.35277627	0.00018362	0.98918853	1
FBgn002563	Roc1a	0.01582018	8.70644653	0.00018137	0.98925483	1
FBgn008396	CG34125	-0.0169889	8.05674186	0.00016732	0.98967964	1
FBgn026325	Cngl	0.02681496	7.91986315	0.00016712	0.98968569	1
FBgn003318	PIG-G	0.08707686	4.42280283	0.0001568	0.99000914	1
FBgn001040	RpS9	-0.0180347	7.18605993	0.00012597	0.99104498	1
FBgn003754	CG2747	-0.0295032	6.85817769	0.00012378	0.99112326	1
FBgn003994	CG17168	-0.0286913	5.68492508	0.00011936	0.99128316	1
FBgn003079	CG9132	-0.0157393	7.91485924	0.0001191	0.99129255	1
FBgn003560	Uev1A	-0.0146471	7.74659964	0.00011479	0.99145162	1
FBgn008540	Pvf3	0.02204645	6.75444468	0.00011281	0.99152556	1
FBgn002352	CG2865	-0.6957036	3.67488808	0.00011204	0.99155479	1
FBgn001158	Dop1R1	-0.1734178	3.83160808	0.00011001	0.99163165	1
FBgn003026	CG15203	-0.0244178	6.16707768	0.00010431	0.99185126	1
FBgn003610	Blos4	0.03023794	5.4961821	0.00010024	0.99201185	1
FBgn002562	CG4199	0.86919157	4.92465953	9.27E-05	0.99231824	1
FBgn002608	CG14817	-0.0164277	6.83612795	7.30E-05	0.99318262	1
FBgn003834	Trissin	-0.0356319	5.20950108	7.29E-05	0.99318849	1
FBgn003004	CG12116	0.02388967	6.31403047	7.27E-05	0.9931961	1
FBgn005258	CG32581	-0.928582	3.90040952	7.27E-05	0.99319687	1
FBgn005228	CG32281	0.21682746	5.56692887	6.67E-05	0.99348307	1
FBgn026642	RPA3	-0.0221942	5.16434083	6.22E-05	0.99370528	1
FBgn003920	REPTOR	-0.0183388	6.04465544	5.77E-05	0.99393924	1
FBgn004451	mRpS21	-0.0190481	5.37482213	4.89E-05	0.99441799	1
FBgn002706	Eb1	-0.0083104	8.38816695	4.83E-05	0.9944554	1
FBgn000241	dco	0.02393625	5.24455244	4.56E-05	0.9946113	1
FBgn003230	CG6700	-0.896641	5.88451561	3.95E-05	0.99498421	1
FBgn003580	CG7492	-0.0985904	4.97441164	3.71E-05	0.99513836	1
FBgn003810	CG7518	0.05362782	1.88737822	3.62E-05	0.99519648	1
FBgn003176	CG9109	-0.0177602	4.94715165	3.09E-05	0.99556805	1
FBgn003425	P32	0.00729093	7.73246993	2.64E-05	0.99589682	1
FBgn000483	Hrb27C	-0.0062347	8.533757	2.63E-05	0.99590763	1

FBgn002066	eIF4B	0.01019549	6.59168413	2.26E-05	0.99620588	1
FBgn000564	Pabp2	-0.0217079	5.43275638	2.08E-05	0.99636238	1
FBgn003724	eIF3a	0.09441898	4.94044441	2.03E-05	0.99640123	1
FBgn003348	CG2269	0.00461581	10.0276058	1.92E-05	0.99650299	1
FBgn003571	velo	-0.254915	4.50026855	1.56E-05	0.99685306	1
FBgn003392	CG8613	0.00455034	8.49592399	1.32E-05	0.99710105	1
FBgn001529	Shc	0.00877451	6.77572556	1.26E-05	0.99716705	1
FBgn003392	CG8617	0.00678915	7.06876298	1.11E-05	0.99734587	1
FBgn002432	Npc1a	0.04278927	6.81235031	1.06E-05	0.99740398	1
FBgn001539	kek1	0.00938503	5.95194286	1.03E-05	0.99744121	1
FBgn003906	Rad60	0.00726829	6.31204518	8.83E-06	0.99762958	1
FBgn003623	viaf	-0.0035809	7.22886761	5.54E-06	0.99812189	1
FBgn000396	usp	0.0383464	5.36995885	5.51E-06	0.99812757	1
FBgn003207	CG9515	0.01457185	5.22066995	3.83E-06	0.99843833	1
FBgn003895	CAH8	0.00701701	5.23800997	3.48E-06	0.99851123	1
FBgn026449	CkIIalpha	-0.0023642	8.01481645	3.22E-06	0.99856809	1
FBgn001522	glec	-0.0019343	7.63667874	1.86E-06	0.99891229	1
FBgn005199	gw	0.00187959	7.10853393	1.76E-06	0.99894211	1
FBgn026689	lncRNA:CR45	0.0267391	2.66282472	1.08E-06	0.99917259	1
FBgn000460	zfh1	-0.0048741	3.70846769	8.08E-07	0.99928279	1
FBgn001558	dare	0.00293759	6.10445652	4.88E-07	0.99944257	1
FBgn000553	RpS17	-0.0008707	8.63425806	3.36E-07	0.99953745	1
FBgn026632	lncRNA:CR44	0.05079261	1.93272231	3.12E-07	0.9995546	1
FBgn003319	CG17985	1.6578379	4.37075846	-0.0001624	1	1
FBgn003441	CG15118	-1.6513062	4.33532608	-0.0001346	1	1
FBgn003207	Tsp29Fb	1.64950564	5.47818287	-0.0005514	1	1
FBgn008671	Egm	-1.6035403	4.39859227	-0.0003361	1	1
FBgn005124	CG31249	1.53935049	4.44906093	-0.000596	1	1
FBgn003923	Nct	1.50798691	5.77238114	-0.0013053	1	1
FBgn003139	CG10880	1.44849394	4.55611615	-0.000862	1	1
FBgn001403	Sptr	-1.4279728	4.61806247	-0.0009429	1	1
FBgn003313	Tsp42El	1.4131954	6.09453446	-0.0018738	1	1
FBgn003584	Uxs	-1.413061	4.23309065	-0.000815	1	1
FBgn003801	MBD-R2	-1.3706122	4.77757457	-0.0011373	1	1
FBgn003622	CG5897	1.30075597	4.66076973	-0.0011448	1	1
FBgn026782	Gmer	1.29184395	4.61118332	-0.001132	1	1
FBgn003755	CG7800	1.27914266	4.70079712	-0.0011942	1	1
FBgn001343	beat-Ia	-1.2605056	4.51798191	-0.0011605	1	1
FBgn002749	Smyd4-4	1.2463985	5.07722794	-0.0014504	1	1
FBgn003118	CG14618	1.24167813	4.33299727	-0.0010726	1	1
FBgn003140	papi	1.22731103	3.8889264	-0.0008976	1	1
FBgn005131	CG31313	1.20993463	4.85593182	-0.0013575	1	1
FBgn005318	Ranbp16	-1.2085931	3.77509258	-0.0009153	1	1
FBgn025914	fid	1.18708824	3.94795955	-0.0009593	1	1
FBgn003980	Cpr100A	-1.1816227	5.66278877	-0.0018875	1	1
FBgn002778	Prp18	1.17127019	5.46465605	-0.0017562	1	1

FBgn003544(	CG14969	1.15703949	5.08116219	-0.0015376	1	1
FBgn002562:	Sik2	1.15142326	6.72769472	-0.0023496	1	1
FBgn001558!	Apc	1.14899487	5.14728494	-0.0016016	1	1
FBgn003162:	CG3294	1.14790478	4.7475608	-0.001343	1	1
FBgn002993!	CG9650	1.14465944	3.83530381	-0.0009511	1	1
FBgn001398:	imd	1.14224922	5.46811463	-0.0017841	1	1
FBgn026295:	RpII140	-1.130655	4.6234818	-0.0001441	1	1
FBgn003977:	CG2224	1.12372637	5.06099266	-0.001547	1	1
FBgn003270:	CG10343	1.12124668	4.21399308	-0.0011223	1	1
FBgn003417!	CG6805	1.11628989	4.91710558	-0.0014712	1	1
FBgn003437!	CG15073	1.11551824	5.17989393	-0.0016575	1	1
FBgn000048!	Pka-C3	1.08890852	4.55793847	-0.0004523	1	1
FBgn003847(	CG18213	1.07845206	4.34137482	-0.0012069	1	1
FBgn003521	CG2211	-1.0755261	4.1588185	-0.0011659	1	1
FBgn003070	CG16952	-1.0736579	4.98659678	-0.00158	1	1
FBgn003952:	dsd	-1.072044	4.54536947	-0.0013221	1	1
FBgn002206!	Nnp-1	-1.0622461	4.58019168	-0.0013426	1	1
FBgn003840	CG5916	1.04502507	3.85777458	-0.0010313	1	1
FBgn004114'	ida	1.03040921	5.14438379	-0.0016774	1	1
FBgn025921(	RhoGAP102/	1.02598293	5.73043989	-0.0004415	1	1
FBgn008701	CG41520	1.01961227	3.34042331	-0.0008638	1	1
FBgn001028:	TfIIFalpha	0.99750811	4.06623635	-0.0011297	1	1
FBgn003598:	Cpr67B	0.97668968	3.76057167	-0.0010426	1	1
FBgn003661·	Golgin104	0.97188794	4.37058117	-0.0012827	1	1
FBgn003238:	CG6686	-0.9619906	6.04359024	-0.002348	1	1
FBgn003935:	CG4730	0.96010735	3.748261	-0.0010481	1	1
FBgn005167:	CG31678	-0.9400088	4.34450764	-0.0013165	1	1
FBgn003859'	CG8064	0.91714292	4.64280017	-0.001409	1	1
FBgn003281:	Hakai	0.90675644	5.80637647	-0.0021437	1	1
FBgn003480:	CG9849	0.90674334	3.66988591	-0.0010526	1	1
FBgn003599(	CG3448	0.90570078	3.47658873	-0.0009875	1	1
FBgn003440:	Jheh2	0.90543658	3.23593446	-0.0009012	1	1
FBgn026253!	CG43093	0.90369204	4.67237928	-0.0014267	1	1
FBgn005229(	Mrtf	0.90078573	5.90485743	-0.0021644	1	1
FBgn005110:	CG31102	0.8987824	4.26392561	-0.0012569	1	1
FBgn000389(	tup	0.89709827	4.65482405	-0.0014202	1	1
FBgn003499'	CG3376	0.89628294	4.47101783	-0.0002345	1	1
FBgn003783·	Art1	-0.8675005	4.15342118	-0.0013131	1	1
FBgn003579:	CG7546	-0.8646089	3.15262522	-0.0009392	1	1
FBgn026369'	Uba3	0.86284107	5.23875181	-0.0018191	1	1
FBgn003339:	Cyp4p2	0.86059926	4.48186242	-0.000287	1	1
FBgn003319	CG1598	0.84713225	6.69498866	-0.0020963	1	1
FBgn004046'	Dlip1	0.84414035	4.83908271	-0.0015513	1	1
FBgn025924	CG42339	0.84208653	4.38054698	-0.0013412	1	1
FBgn003889(	CG7956	-0.8413011	5.28315523	-0.0007451	1	1
FBgn002896:	A16	0.83956902	4.80180178	-0.0015362	1	1

FBgn003544	CG12010	0.83666161	4.23084298	-0.0012753	1	1
FBgn026093	Vps15	0.8330333	4.6912411	-0.0014663	1	1
FBgn003435	rswl	-0.8225125	4.82059876	-0.0001016	1	1
FBgn002557	Pli	-0.8222987	5.03581452	-0.0017086	1	1
FBgn000047	DNaseII	-0.8156675	3.6848139	-0.0011345	1	1
FBgn000488	tok	-0.8141417	3.29979564	-0.0010211	1	1
FBgn003461	Panx	0.80553332	4.45680863	-0.0013817	1	1
FBgn002518	az2	0.80378838	5.02501843	-0.0016818	1	1
FBgn003911	Lsd-1	0.79926422	5.10388554	-0.0009416	1	1
FBgn003236	CG6488	-0.7973686	3.94434673	-0.0012123	1	1
FBgn000313	PpV	-0.7934277	5.05670072	-0.000957	1	1
FBgn026583	lncRNA:CR44	0.78833206	4.29397771	-0.0013122	1	1
FBgn003843	CG14883	0.77773722	6.01527826	-0.0017402	1	1
FBgn002353	CG3740	0.77074661	3.48658363	-0.0010574	1	1
FBgn004025	Ugt302C1	0.77054479	4.16939298	-0.0012709	1	1
FBgn003636	CG14109	0.75906088	3.57054381	-0.0010916	1	1
FBgn003225	holn1	0.75763376	5.14013295	-0.0017839	1	1
FBgn003845	CG10324	-0.7571657	3.9676576	-0.0012291	1	1
FBgn004033	brv3	0.75413258	4.0591475	-0.0012252	1	1
FBgn005294	CG32944	-0.7534828	3.19638614	-0.0010133	1	1
FBgn003431	Nup75	0.74839653	4.52220462	-0.0014103	1	1
FBgn002317	amon	0.74080951	6.82982619	-0.001799	1	1
FBgn003510	CG13876	0.74009052	5.14103372	-0.0018096	1	1
FBgn003674	anchor	0.73672146	4.42485618	-0.0013902	1	1
FBgn003630	Hip1	0.73602028	4.23897764	-0.0013093	1	1
FBgn002971	CG11436	0.73476144	5.48016539	-0.0012541	1	1
FBgn005164	DIP-theta	-0.7342162	5.15676525	-0.0018319	1	1
FBgn003570	CG8270	0.73322384	5.18684176	-0.0018275	1	1
FBgn001533	abs	0.73310743	4.07752681	-0.0012438	1	1
FBgn005243	SMC5	0.73079212	3.27735353	-0.0010146	1	1
FBgn003953	CG12883	0.72893394	3.34314862	-0.0010366	1	1
FBgn003292	Mpp6	0.7208673	3.36047953	-0.001046	1	1
FBgn003854	CG17802	0.7183166	5.71895653	-0.0021221	1	1
FBgn001535	CG14906	0.71502794	4.56626762	-0.0014386	1	1
FBgn006762	CG33332	0.70956779	3.52855146	-0.0010973	1	1
FBgn002757	GABA-B-R2	0.70805186	5.49349562	-0.000654	1	1
FBgn003691	Mtr3	0.70373492	3.47423475	-0.0010828	1	1
FBgn002968	CG6428	-0.6916335	3.32016698	-0.0010773	1	1
FBgn002837	heix	0.68703653	4.27276439	-0.0002272	1	1
FBgn003733	Pi4KIIalpha	0.68686252	4.54216347	-0.0004771	1	1
FBgn006503	AlkB	0.67870701	3.26849669	-0.0010322	1	1
FBgn003326	coil	0.67343048	4.25093668	-0.0013261	1	1
FBgn003490	CG4797	-0.6716713	3.18726413	-0.0010425	1	1
FBgn008636	alph	0.66455862	4.99364274	-3.32E-05	1	1
FBgn003452	CG9945	-0.6608811	5.34158222	-0.0002389	1	1
FBgn005200	CG32006	0.65680245	3.57945716	-0.0011302	1	1

FBgn003063	CG6227	-0.6540685	2.94124555	-0.0009649	1	1
FBgn003673	Edc3	-0.651579	5.56735026	-0.0020487	1	1
FBgn004300	Chrac-16	0.65107838	3.41630169	-0.0010846	1	1
FBgn003907	CG4393	0.65106854	3.42281496	-0.0010864	1	1
FBgn003635	CG10222	0.64942017	3.38084485	-0.0010745	1	1
FBgn003199	CG8460	0.64801218	5.6182289	-0.0020744	1	1
FBgn003676	TrpRS-m	0.64711744	4.72161205	-0.0015354	1	1
FBgn026198	RASSF8	-0.6462921	5.42675255	-0.0019976	1	1
FBgn003966	CG2310	0.63951532	3.39951141	-0.0010827	1	1
FBgn003271	CG17597	0.63272621	4.80472094	-0.0016091	1	1
FBgn026327	Fic	0.63207075	3.9283145	-0.0012254	1	1
FBgn003470	Vps35	-0.6278178	5.77681399	-0.0020836	1	1
FBgn026137	CG33228	0.62443433	2.64758309	-0.0008265	1	1
FBgn002221	Cse1	0.61999092	4.5503012	-0.0014516	1	1
FBgn005319	pen-2	0.61756556	3.99489571	-0.0012404	1	1
FBgn005234	Atac3	0.61717033	4.75510772	-0.0015594	1	1
FBgn003360	CG9062	0.61284616	4.27101255	-0.0013499	1	1
FBgn003204	CG13088	0.61130422	3.95128871	-0.0012379	1	1
FBgn003681	HipHop	-0.605651	4.01966093	-0.0001841	1	1
FBgn003582	HP4	0.60195494	4.40673721	-0.0014169	1	1
FBgn000044	Hr3	-0.6006648	2.82667289	-0.0009437	1	1
FBgn003876	trem	0.59829649	3.86166216	-0.0012114	1	1
FBgn003564	CG13288	0.59622878	4.29855929	-8.45E-05	1	1
FBgn008644	PIG-Q	-0.5916515	3.89838361	-0.0012492	1	1
FBgn028368	Tcs3	0.58439481	3.3521484	-0.001092	1	1
FBgn003916	CG5510	0.5819683	5.17656369	-0.001859	1	1
FBgn026187	dpr2	0.58145368	5.77431503	-0.0021707	1	1
FBgn003468	CG11474	0.58095685	5.55945785	-0.0020417	1	1
FBgn006610	LpR1	0.57241008	5.68210053	-0.0008327	1	1
FBgn003212	CG33298	-0.5707782	4.09747301	-0.0013098	1	1
FBgn003639	CG17364	0.56878337	3.48185422	-0.0011278	1	1
FBgn002027	mre11	-0.5684271	4.69680471	-0.0015509	1	1
FBgn003126	cold	0.56481896	4.78629443	-0.0015859	1	1
FBgn001158	Ms	-0.5614681	5.71100496	-0.0003374	1	1
FBgn003247	CG5439	0.55923764	5.46419376	-0.0003027	1	1
FBgn000481	fs(2)ltoPP43	-0.5584821	4.95845026	-0.0003121	1	1
FBgn008685	CG11555	0.55487798	4.5056419	-0.0014537	1	1
FBgn002318	Orc4	0.55379368	4.92024939	-0.0016709	1	1
FBgn026642	Ote	0.55350232	4.02938276	-0.0012609	1	1
FBgn002784	CstF64	0.55090931	5.53472509	-0.0020398	1	1
FBgn002840	Sin	0.54984544	4.96859047	-0.0002447	1	1
FBgn003177	CG9154	0.54802987	3.7579076	-0.0012005	1	1
FBgn003417	AsnRS-m	0.54718772	6.01686389	-0.0024079	1	1
FBgn003046	CG15743	0.53980206	3.77876158	-0.0012047	1	1
FBgn003518	CG9119	0.5361753	6.1029577	-0.0024778	1	1
FBgn003188	Rab30	0.53443546	5.82804559	-0.0006302	1	1

FBgn003402[illegible]	eIF2Bgamma[illegible]	-0.531854	5.0487836	-0.0017586	1	1
FBgn026236[illegible]	CG43064	0.52697575	4.14609602	-0.0013225	1	1
FBgn003354[illegible]	Elp2	0.52381442	3.81464186	-0.0012143	1	1
FBgn003701[illegible]	CG4074	-0.5186946	4.29652542	-0.001393	1	1
FBgn026438[illegible]	opm	0.51816456	3.43950822	-0.001128	1	1
FBgn003127[illegible]	CG13689	0.51710439	3.6478146	-0.0011827	1	1
FBgn003620[illegible]	CG10907	0.51663378	5.49233646	-0.0020167	1	1
FBgn000107[illegible]	ftz-f1	0.51203843	5.11259631	-0.0010243	1	1
FBgn003380[illegible]	CG4627	0.49794584	5.04343456	-0.0017475	1	1
FBgn003690[illegible]	CG14102	0.49567096	3.92526621	-0.0012501	1	1
FBgn003388[illegible]	DJ-1alpha	0.49566096	2.97122568	-0.0009924	1	1
FBgn008644[illegible]	DCTN6-p27	0.49406473	4.67192864	-0.0015192	1	1
FBgn003956	mfrn	0.4934683	5.49166406	-0.001668	1	1
FBgn002340[illegible]	B4	0.4886654	3.26470101	-1.78E-06	1	1
FBgn003906[illegible]	wda	-0.4858623	4.35047396	-0.001428	1	1
FBgn003549[illegible]	Fit1	-0.484803	6.33353773	-0.000454	1	1
FBgn004007[illegible]	pont	0.48149637	5.08829191	-0.0017893	1	1
FBgn001034[illegible]	140up	-0.4803959	4.33095777	-0.0014166	1	1
FBgn003965[illegible]	Mesh1	0.47742782	4.15606623	-0.0013289	1	1
FBgn005042[illegible]	CG30424	-0.4736829	4.48500566	-0.0014719	1	1
FBgn003002[illegible]	CG2147	0.47196116	3.83658735	-0.0012287	1	1
FBgn003051[illegible]	CG11134	0.47032571	4.66053189	-0.0015151	1	1
FBgn003906[illegible]	CG13827	0.46985259	4.99023432	-0.0017459	1	1
FBgn002864[illegible]	beat-Ib	-0.4694058	4.48278832	-0.0014726	1	1
FBgn003770[illegible]	CG9386	0.46805629	6.12635287	-0.0021154	1	1
FBgn026199[illegible]	CG42808	-0.4677585	3.24347072	-0.0011115	1	1
FBgn002423[illegible]	Hs2st	0.46620497	2.63735956	-0.0008848	1	1
FBgn003376[illegible]	ZnT49B	-0.46283	6.58848446	-0.0031353	1	1
FBgn003605[illegible]	iPLA2-VIA	0.46169669	4.7511624	-0.0015753	1	1
FBgn025086[illegible]	CG42238	-0.4538838	5.16409522	-0.00124	1	1
FBgn000361[illegible]	Su(var)2-10	0.45063937	5.05521758	-0.0006563	1	1
FBgn003410[illegible]	CG7747	0.44248394	5.24360246	-0.0019057	1	1
FBgn003847[illegible]	cal1	0.44177002	4.37508731	-0.0014226	1	1
FBgn026252[illegible]	nsl1	-0.4416961	4.0519707	-0.0008152	1	1
FBgn003149[illegible]	CG17219	0.43528775	3.9888504	-0.000858	1	1
FBgn003397[illegible]	HPS1	-0.4348308	6.05843711	-0.002182	1	1
FBgn003771[illegible]	bocks	0.42558986	5.49487543	-0.0005917	1	1
FBgn003389[illegible]	CG13016	-0.4090364	4.39477746	-0.0014483	1	1
FBgn025923[illegible]	Camta	-0.4073629	4.63268962	-0.0012071	1	1
FBgn003699	CtIP	-0.4013795	2.61861341	-0.0009252	1	1
FBgn003718[illegible]	CG7369	0.40134551	4.02334542	-0.0004856	1	1
FBgn003854[illegible]	CG17806	0.39638995	4.03826172	-0.0012788	1	1
FBgn002484[illegible]	p38b	0.39624373	4.75371201	-0.0015763	1	1
FBgn003690[illegible]	Ccdc58	-0.3959555	3.51837986	-0.0011882	1	1
FBgn003036[illegible]	Cyp311a1	0.39237273	4.00275212	-0.0012685	1	1
FBgn003644[illegible]	CG9384	0.39126384	4.76178008	-3.47E-05	1	1

FBgn003287	CG2608	-0.3910306	4.709222	-0.0015681	1	1
FBgn003185	CG17378	0.38992837	4.33102951	-0.0014013	1	1
FBgn003139	CG15385	0.38991624	4.29570603	-0.00138	1	1
FBgn026128	bou	0.3854817	2.60384298	-0.000886	1	1
FBgn003430	CG10915	0.38455691	4.89013469	-0.0006933	1	1
FBgn003759	CG11737	-0.3832559	3.90326162	-0.0012692	1	1
FBgn026074	Utx	-0.3832049	2.90328988	-0.0010211	1	1
FBgn002030	dve	0.37683176	2.39471967	-0.0008078	1	1
FBgn003819	CCHa1	0.37439847	6.48835785	-0.0029362	1	1
FBgn003581	CG7409	-0.3659847	5.92104173	-0.0022919	1	1
FBgn003876	MED25	-0.3652209	4.67829683	-0.0015432	1	1
FBgn003408	CG8441	0.36520142	4.67861819	-0.0015272	1	1
FBgn003023	CG15211	0.36279537	3.4797635	-0.0011538	1	1
FBgn002423	gprs	-0.3584209	4.97322852	-0.0017301	1	1
FBgn026497	Slh	-0.3513543	3.78418828	-0.000759	1	1
FBgn001372	pck	-0.3495288	2.65840752	-0.000946	1	1
FBgn005305	CG33054	0.3491896	2.86639481	-0.0009767	1	1
FBgn002510	Bem46	-0.3465884	4.24701009	-0.0013777	1	1
FBgn003948	CG14259	0.34644544	4.48045344	-0.0014522	1	1
FBgn002837	kek3	-0.3429441	4.23103802	-0.0013764	1	1
FBgn005010	CG30109	0.33897841	4.32590547	-0.0013916	1	1
FBgn002480	Dbp80	0.32424035	6.74897971	-0.0034149	1	1
FBgn003784	CG6574	-0.3238368	4.33371431	-0.0014169	1	1
FBgn003626	Rh7	-0.3237337	3.83522591	-0.001285	1	1
FBgn002829	ric8a	0.32117114	4.13627521	-0.001321	1	1
FBgn003636	CG10089	-0.3200593	3.18004944	-0.0011037	1	1
FBgn002187	Zfrp8	-0.3191495	4.02671844	-0.0006082	1	1
FBgn005118	CG31189	0.31836697	3.31945911	-0.0011119	1	1
FBgn001077	ppan	0.31632571	5.14820712	-0.0018231	1	1
FBgn003840	Fer2	0.31628989	4.08610129	-0.0012952	1	1
FBgn003552	CG1316	0.31364491	6.03700092	-0.0008854	1	1
FBgn004158	tamo	-0.3103997	2.91556556	-0.0010282	1	1
FBgn003149	CG3542	-0.3103215	5.9457945	-0.0023096	1	1
FBgn003146	G6P	-0.3095641	4.59837049	-0.0014929	1	1
FBgn003611	Blos2	0.30892249	4.32597994	-0.0013866	1	1
FBgn001058	dock	-0.3066775	5.36259411	-0.0019705	1	1
FBgn003408	CG10731	0.29824914	3.35866983	-0.0011233	1	1
FBgn026111	APP-BP1	0.29318923	3.77688249	-0.0012093	1	1
FBgn005195	CG31957	-0.2929742	3.3803506	-0.0011513	1	1
FBgn003310	CG15236	-0.291377	4.1656093	-0.0013397	1	1
FBgn002497	CG2701	-0.2910329	4.29799072	-0.0001736	1	1
FBgn005363	CG33639	0.28795724	3.90900219	-0.0012388	1	1
FBgn003930	RIOK2	0.28785121	3.35055026	-0.0011179	1	1
FBgn005245	SPoCk	-0.2846521	5.45689746	-0.0001483	1	1
FBgn003287	Cen	-0.2846412	5.45523272	-0.0019931	1	1
FBgn003732	CG14669	-0.2844432	4.17580363	-0.0013439	1	1

FBgn003698	CG5274	0.28320127	4.84017132	-0.0016088	1	1
FBgn006611	GlcAT-I	0.28239374	4.63518179	-0.0006075	1	1
FBgn003017	Naxe	0.27679437	4.59320736	-0.001465	1	1
FBgn003119	CG17600	-0.2710472	3.61032555	-0.0012041	1	1
FBgn002958	CR14798	0.26984366	5.00292386	-0.0017118	1	1
FBgn008541	Rgk2	-0.2629063	3.16933771	-0.0010944	1	1
FBgn003221	CYLD	-0.262164	5.73464009	-0.0012069	1	1
FBgn003077	CG4678	-0.2574193	6.05366046	-0.0023962	1	1
FBgn000005	Pfas	0.25148863	6.42027663	-0.0028033	1	1
FBgn005207	Alg10	0.25133949	4.85214456	-0.0014236	1	1
FBgn000161	Grip91	0.24527541	4.53167376	-0.0014267	1	1
FBgn003423	eIF3b	-0.2423068	5.73553461	-0.0012087	1	1
FBgn003137	CG15362	0.23996395	2.91078873	-0.0009667	1	1
FBgn026442	CG43845	-0.239714	3.40356885	-0.001144	1	1
FBgn003512	wac	0.2381166	2.66183405	-0.0008838	1	1
FBgn003203	CG13392	0.23740876	3.34517314	-0.001098	1	1
FBgn003833	CG14868	-0.2355306	5.8378347	-0.0021917	1	1
FBgn003055	Fbxl4	-0.2344911	4.12153333	-0.0013048	1	1
FBgn003449	CG10444	-0.234155	3.38444103	-0.0011374	1	1
FBgn003449	Hsl	0.23315027	6.0479184	-0.0023591	1	1
FBgn005217	CG32176	-0.2287854	3.63317657	-0.0011934	1	1
FBgn003861	CG7685	0.22876655	4.12505344	-0.0012779	1	1
FBgn026108	Syt12	0.22875895	4.30499899	-0.0013429	1	1
FBgn003794	prd1	0.22784933	5.6513112	-0.0004368	1	1
FBgn002041	JIL-1	0.22573649	4.53642239	-0.0014157	1	1
FBgn003654	CG17032	0.22389601	3.84302849	-0.0011935	1	1
FBgn005015	CG30158	0.22212964	3.54729066	-0.0011343	1	1
FBgn003761	TMEM216	-0.2194502	2.67920381	-0.0009388	1	1
FBgn003182	KFase	-0.2150327	3.7408584	-0.0012078	1	1
FBgn003065	CG7860	0.21297157	4.75737459	-0.001518	1	1
FBgn003841	CG14879	0.21208089	3.31923353	-0.0010667	1	1
FBgn002986	CG3847	0.20917471	3.97136413	-0.0012135	1	1
FBgn003177	CG13995	-0.2063908	4.13509131	-0.0013028	1	1
FBgn005400	CG34001	-0.2059407	2.48145698	-0.0008657	1	1
FBgn003529	SCOT	0.19961589	4.71865557	-0.0014891	1	1
FBgn004670	Haspin	-0.1977649	5.51280335	-0.0019737	1	1
FBgn025982	CG42402	0.19485161	4.60995704	-0.0009964	1	1
FBgn004263	Sox21b	0.19046735	2.62605288	-0.0008273	1	1
FBgn002051	Abi	0.18943767	4.27624642	-0.0012996	1	1
FBgn026769	Pak	0.18625037	4.30962017	-0.0008548	1	1
FBgn001601	Faa	-0.1845288	2.63757239	-0.0009108	1	1
FBgn003737	kat-60L1	0.18374629	5.32538084	-0.0018408	1	1
FBgn005111	CG31111	0.18167615	3.60263659	-0.0011091	1	1
FBgn003190	CG5181	0.17814181	4.65768032	-0.0014205	1	1
FBgn003682	CG6843	-0.1777885	4.36945767	-0.0013713	1	1
FBgn026253	CG43092	0.17569599	4.87459416	-0.0015493	1	1

FBgn000865	lbl	-0.1739226	2.74962862	-0.0009375	1	1
FBgn026449	gpp	0.17240553	4.99342522	-0.0009839	1	1
FBgn003973	CG11504	-0.1678749	5.08415234	-0.0016941	1	1
FBgn000460	zfh2	0.16651322	5.50941839	-0.0014003	1	1
FBgn003029	CG1738	-0.162798	4.07915989	-0.001245	1	1
FBgn003861	CG12333	0.16100272	4.21810118	-0.0012504	1	1
FBgn003704	CG10581	-0.1575607	2.96679717	-0.0009872	1	1
FBgn003125	CG11885	0.15394473	4.12115259	-0.0011925	1	1
FBgn001074	Srp54k	-0.1529314	6.15532706	-0.0023892	1	1
FBgn000254	robo2	0.14755101	2.43557684	-0.0006693	1	1
FBgn003827	Sdr	-0.1437835	3.9322775	-0.0011857	1	1
FBgn005148	pb	-0.143139	3.04763383	-0.0009962	1	1
FBgn003876	CG4936	0.1430414	5.29669218	-0.001755	1	1
FBgn002029	stumps	0.14233243	3.39183005	-0.0003703	1	1
FBgn000396	vap	0.14129478	4.66925614	-0.0012579	1	1
FBgn004670	Liprin-alpha	-0.1392557	2.62369383	-0.0008683	1	1
FBgn002968	CG2938	-0.1375625	4.78948762	-0.0014833	1	1
FBgn002061	SA	-0.1363244	4.37862549	-0.0013253	1	1
FBgn003251	CG9302	0.1358695	5.40701451	-0.0017823	1	1
FBgn006149	loj	-0.1324538	4.19059129	-0.0012472	1	1
FBgn002913	Patsas	-0.1280779	2.87639941	-0.000925	1	1
FBgn026335	CG31688	0.12636784	4.05706243	-0.0011105	1	1
FBgn002298	qkr58E-1	-0.1254867	4.44387916	-0.0013225	1	1
FBgn003032	CG2247	-0.1245072	4.53723531	-0.0013352	1	1
FBgn003788	CG17726	0.12203941	4.23664513	-0.0011703	1	1
FBgn026048	Ziz	0.12162717	3.37720694	-0.0003725	1	1
FBgn004630	CG10650	-0.1196268	4.90815286	-0.0015204	1	1
FBgn004032	GNBP2	0.11803008	3.52898622	-0.0009546	1	1
FBgn003035	sicily	0.11661645	4.52413297	-0.0011657	1	1
FBgn003242	CG6583	-0.1143243	3.68157396	-0.0010932	1	1
FBgn003770	CG8149	0.10756049	5.07494967	-0.0014938	1	1
FBgn001030	hep	-0.1056551	4.4908496	-8.21E-08	1	1
FBgn003626	thoc6	0.10499688	5.32057537	-0.0016425	1	1
FBgn003347	CG1407	0.10382583	4.89745589	-0.0013844	1	1
FBgn003934	CG5079	0.10263796	3.28819975	-0.0008232	1	1
FBgn008541	CG34384	-0.0986306	4.36316578	-0.001026	1	1
FBgn026138	IntS6	-0.094358	2.57095448	-0.0007808	1	1
FBgn001035	Taf1	-0.0930606	2.81084471	-0.0008493	1	1
FBgn003453	CG16799	-0.0916024	2.01641675	-0.0005889	1	1
FBgn002187	Nle	0.09014612	4.04567614	-0.0009749	1	1
FBgn003073	CG3632	0.08952404	4.7236625	-0.0012258	1	1
FBgn003734	Rev7	0.08568762	4.19379485	-0.0010116	1	1
FBgn003619	CG14135	0.08065243	5.19353415	-0.0014418	1	1
FBgn003739	CG11459	-0.0803026	2.63944676	-0.0007691	1	1
FBgn003002	Corp	-0.0785549	2.49261112	-0.0007185	1	1
FBgn004390	pygo	-0.0772559	2.34160232	-0.0006696	1	1

FBgn003720	Ssl1	0.07682885	3.55192714	-0.0007542	1	1
FBgn001580	Reg-5	0.07465689	4.53125461	-0.0010523	1	1
FBgn026273	mub	-0.0724411	6.22299839	-0.0021168	1	1
FBgn026362	asRNA:CR43	-0.0720809	3.67159984	-0.0009782	1	1
FBgn003749	CG10053	0.06839103	3.46357015	-0.0006512	1	1
FBgn003369	CG8858	-0.0663947	2.09693073	-0.0005582	1	1
FBgn000490	Prat	-0.0611303	4.83467629	-0.0013119	1	1
FBgn003722	TwdlG	0.05402851	5.96532908	-0.0003895	1	1
FBgn028682	lncRNA:CR43	0.05060824	3.5161892	-0.0004635	1	1
FBgn000334	RanGAP	-0.0459972	3.49466047	-0.000824	1	1
FBgn003736	Rab23	0.04570053	3.55639371	-0.0004049	1	1
FBgn002619	nop5	-0.0434794	2.92110228	-0.0006991	1	1
FBgn002985	CG3781	-0.0404856	3.95980957	-0.0008612	1	1
FBgn001402	Rlb1	0.04044731	4.24581538	-0.0005858	1	1
FBgn002351	CG14805	-0.0397547	4.5483104	-0.0009921	1	1
FBgn004170	CG3253	0.03456013	5.27894533	-0.0009056	1	1
FBgn003671	CG6479	-0.0340034	4.3820066	-0.0009145	1	1
FBgn026366	Lpt	0.03297177	3.81851708	-0.0002773	1	1
FBgn005207	CG32075	-0.0319151	4.68067484	-0.0009576	1	1
FBgn026349	mld	0.02951106	2.44358958	-1.83E-05	1	1
FBgn003955	CG12259	0.0294658	5.69981248	-0.0018428	1	1
FBgn024351	cnir	0.02892931	3.19926248	-9.34E-06	1	1
FBgn026104	Dscam3	0.0285379	4.46987439	-0.0004222	1	1
FBgn003393	Dh44-R1	-0.024967	5.64331149	-0.0016323	1	1
FBgn026645	Kr-h1	-0.0245925	2.25491564	-0.0004319	1	1
FBgn003604	Ilp2	0.02212875	2.0977836	-7.51E-05	1	1
FBgn001562	Clp	0.01922504	3.7828482	-9.62E-07	1	1
FBgn003405	CG8366	-0.0175432	4.54594252	-3.12E-05	1	1
FBgn003386	CG13337	-0.0172341	3.07072393	-0.0005369	1	1
FBgn003927	CG11836	0.01722498	4.46303173	-7.15E-05	1	1
FBgn002642	Su(var)2-HP2	-0.0170447	1.94190114	-0.0003136	1	1
FBgn000489	fd96Cb	0.01646497	4.95063695	-0.0001831	1	1
FBgn005208	CG32085	0.0135072	5.47680991	-0.0002199	1	1
FBgn003915	CG13605	0.01336219	4.94006901	-4.63E-05	1	1
FBgn003201	CG7810	0.01224658	5.58553396	-0.0001862	1	1
FBgn004096	lncRNA:CR13	0.01220656	2.92424407	-0.0001577	1	1
FBgn003046	CG1622	0.01169489	4.3366653	-3.05E-05	1	1
FBgn003494	CG16787	0.01035575	5.00424802	-4.34E-06	1	1
FBgn003805	CG12267	0.01028297	4.36751248	-6.53E-05	1	1
FBgn026399	CG43737	0.00980399	5.61734953	-0.0005808	1	1
FBgn003618	Duba	0.00965595	5.01166354	-1.85E-05	1	1
FBgn001033	128up	-0.0092445	4.92494354	-0.0006779	1	1
FBgn003330	Cyp6a13	0.00869337	3.07929373	-0.000207	1	1
FBgn003494	PPP1R15	0.00765411	5.75368175	-4.48E-06	1	1
FBgn026541	Neto	-0.0056287	3.79166212	-3.09E-07	1	1
FBgn002567	CkIIalpha-i3	-0.004732	4.30949072	-0.0004752	1	1

FBgn003748[illegible]	CG1234	0.00368389	5.0190459	-0.0002795	1	1
FBgn003850[illegible]	Sur-8	0.0030625	6.08522235	-1.18E-06	1	1
FBgn004629[illegible]	CG11534	0.00143551	2.62294567	-0.0002856	1	1
FBgn026170[illegible]	gce	-0.0008567	4.13820926	-6.57E-08	1	1
FBgn026634[illegible]	CngB	0.0001133	3.76857856	-0.0003469	1	1

TH_GFP_1	TH_GFP_3	TH_UbxRi_3	TH_UbxRi_1b
-2.9534214	-2.9534214	10.4819851	12.3840427
-2.9534214	-2.9534214	12.133087	7.91098474
-2.9534214	-2.9534214	10.8265427	8.44436766
-2.9534214	-2.9534214	10.8170592	7.92364518
-2.9534214	-2.9534214	10.4315489	6.6205267
-2.9534214	0.41054961	11.0966997	13.8932039
-2.9534214	-2.9534214	9.96802859	7.06859165
-2.9534214	-2.9534214	10.1124713	6.05196925
-2.9534214	-2.9534214	10.2273244	4.34216457
-1.0415346	-2.9534214	11.842449	8.91897737
-2.9534214	-2.9534214	8.27507606	9.26949346
-2.9534214	-2.4449602	9.89467798	6.72341303
-2.9534214	-2.9534214	7.66157233	9.42068686
-2.9534214	-2.9534214	9.32454144	7.59773921
-2.9534214	-2.9534214	9.0153547	8.17502519
-2.9534214	-2.9534214	9.44588295	6.83867507
-2.9534214	-2.9534214	8.86711787	7.87976732
-2.9534214	-2.9534214	9.43383024	5.69911188
-2.9534214	-2.9534214	8.34191414	8.57507445
-2.9534214	-2.0697503	9.77883671	4.29282823
-2.9534214	3.09250094	10.9211073	6.85764939
2.34015605	3.09250094	11.5792516	9.11346441
-2.9534214	-2.9534214	9.25791317	5.26266914
-2.9534214	3.45021042	11.0948115	13.0923362
-2.9534214	-2.9534214	8.55382976	7.55536633
-2.9534214	-2.9534214	9.17051261	5.09370617
0.79434634	2.56348907	10.7178749	7.49484232
3.32164624	2.37912504	13.1612964	9.02117971
-2.9534214	-1.5257463	9.46069583	4.51465971
-2.9534214	-2.4449602	8.98639955	6.43084683
-2.9534214	-2.9534214	8.7897425	6.40914607
-2.9534214	5.40244653	11.4758689	7.37813764
-2.9534214	4.57970594	10.8785729	7.94331837
-2.9534214	-2.9534214	9.00487392	3.7181568
-2.9534214	-2.9534214	8.89520749	4.82342191
-2.9534214	-2.9534214	6.0137781	8.82978744
7.83293183	7.91984975	-2.9534214	-2.9534214
-2.0115616	-2.9534214	11.0218273	3.7181568
8.64813736	7.8949376	-1.2416692	-0.9773123
-2.9534214	-2.4449602	8.84147644	5.44935696
-2.9534214	-2.4449602	8.43333343	7.04229592
-2.9534214	-2.9534214	6.93304524	8.36569739
3.71687841	2.28537678	10.436298	9.04393034
-2.9534214	-2.9534214	8.58892571	5.84316031
-2.9534214	-0.3516332	9.176568	4.76029215

-2.0115616	3.13791053	9.61351438	5.92459562
-2.9534214	-2.9534214	8.50537469	5.1926647
7.80941841	7.81681323	-2.9534214	-2.2872455
-2.9534214	-2.9534214	7.2255824	7.84263092
-2.9534214	4.33262153	9.84496501	6.19609178
8.20983615	7.04650859	-2.9534214	-1.8332537
5.06272305	5.93217859	12.8589346	8.64144974
-2.9534214	-2.9534214	8.15753816	5.63236873
-2.9534214	4.80192595	9.81080577	7.30744288
4.24604466	4.71769805	9.8031169	9.29477466
-2.9534214	-2.9534214	8.31255289	4.56632356
-2.9534214	-2.9534214	7.37349054	7.2243597
-2.9534214	-2.9534214	8.34387221	4.12756888
-2.9534214	-2.9534214	7.99260295	5.99483892
-2.9534214	-2.9534214	7.91964771	6.19310701
7.63572091	7.39166097	-2.9534214	-2.2872455
-2.9534214	-2.9534214	7.93098095	6.06835058
-2.9534214	-2.9534214	7.60772339	6.82138217
-2.9534214	1.74309531	8.89364884	6.01863778
-0.2472184	-2.9534214	8.68035283	7.13152823
-2.9534214	-2.9534214	7.56415675	6.75006006
-2.9534214	-2.9534214	7.43846083	6.94096458
8.29722791	5.6450663	-2.9534214	-1.8332537
8.53799808	7.38178547	-2.9534214	1.72736257
1.02258002	1.30727289	10.4424028	5.04818166
-2.9534214	-2.9534214	6.59777943	7.65688784
-2.9534214	-2.9534214	7.45667111	6.79504784
-2.9534214	-2.9534214	8.08914682	4.81179359
-2.9534214	-2.9534214	8.06323479	4.90958885
-2.9534214	-2.9534214	7.11527349	7.21261671
7.92262407	6.85312909	-2.3036717	-2.9534214
-2.9534214	-2.9534214	8.12298775	4.43098864
7.69404224	6.08258692	-2.9534214	-2.9534214
-1.4465118	2.05858306	8.53052219	7.46236168
7.23799858	6.93837322	-2.9534214	-2.9534214
-2.9534214	-2.9534214	7.70182529	5.95662898
-2.9534214	-2.9534214	8.01571458	4.43098864
-2.9534214	-2.9534214	7.59572249	6.20204286
5.38558931	6.56888718	11.158915	9.15163367
7.4016492	8.75797456	-2.3036717	1.76000118
-2.9534214	-2.9534214	7.80540103	5.29086398
6.83562706	7.16475981	-2.9534214	-2.9534214
-2.9534214	0.82014925	8.56590316	5.79081305
-2.9534214	-1.3152731	8.26270932	5.60568039
7.77928206	6.75234652	-2.9534214	-1.8332537
-2.9534214	-2.9534214	8.00585489	3.96209569

-2.9534214	-2.9534214	8.05369564	3.31882493
-2.9534214	-0.6895489	8.20031481	6.2415831
-2.9534214	-2.4449602	7.81013001	6.07647202
6.73051136	7.14376567	-2.9534214	-2.9534214
-2.9534214	-2.9534214	7.04560075	6.77522626
-2.9534214	-1.7722575	7.73104535	6.79011791
6.81215789	7.01703863	-2.9534214	-2.9534214
-2.9534214	-2.9534214	7.76991635	4.70688394
10.6028305	12.4057198	7.96233337	7.81571512
3.11937512	-0.6895489	6.76005577	8.9750075
8.44236623	6.00260679	-1.8572999	0.64950605
1.95402673	6.12717926	10.3450724	6.25452598
8.55383268	7.531788	-2.9534214	3.14437975
-2.9534214	-2.9534214	7.97754755	3.18099711
-2.9534214	-2.9534214	7.44029224	5.81431329
6.74737418	6.97948984	-2.9534214	-2.9534214
-2.9534214	-1.1316325	8.2471003	4.97708254
6.49597553	7.15153594	-2.9534214	-2.9534214
-2.9534214	-2.9534214	7.01315663	6.50853563
-2.9534214	-2.9534214	7.64260834	5.01137656
-2.9534214	-2.9534214	7.16411283	6.15986037
6.4047763	7.62363785	-2.3036717	-2.9534214
10.191725	8.8165747	-2.3036717	7.1377511
7.69941669	4.39753848	-2.9534214	-2.9534214
4.93676553	5.5441108	9.17693418	9.63160282
-2.9534214	-2.4449602	6.6930234	7.48083667
7.04690878	6.33056944	-2.9534214	-2.9534214
1.54760822	-1.5257463	8.69064726	7.04643636
-2.9534214	-2.9534214	7.20418505	5.96540235
7.47724238	6.39651121	-2.9534214	-2.2872455
6.62062146	6.81309704	-2.9534214	-2.9534214
1.71370892	1.42917742	8.77215801	7.12449527
-2.9534214	-2.9534214	7.59352974	4.54306909
-0.8749787	2.48131403	8.67050462	6.48676241
-2.9534214	-2.4449602	6.96052994	7.10795046
7.1579799	5.96907926	-2.9534214	-2.9534214
-2.9534214	-2.9534214	7.30946934	5.47906836
6.68288715	6.66818151	-2.9534214	-2.9534214
6.69204096	6.63926203	-2.9534214	-2.9534214
5.49613854	7.40704431	-2.9534214	-2.9534214
6.61887715	6.6455642	-2.9534214	-2.9534214
6.63363701	6.55723481	-2.9534214	-2.9534214
5.7876576	7.15485329	-2.9534214	-2.9534214
-2.9534214	-2.9534214	6.66196885	6.28568373
-2.9534214	-2.9534214	7.50763498	4.25897437
-2.9534214	-2.9534214	6.70017428	6.12895657

10.3887072	10.4986879	4.30297829	8.18369751
-2.9534214	-2.9534214	7.10453429	5.31302972
-2.9534214	-2.9534214	6.93477846	5.5507753
-2.9534214	-2.9534214	6.66301485	6.01695082
7.00612171	5.51686898	-2.9534214	-2.9534214
-2.9534214	-2.9534214	7.06155324	5.20457055
6.41486391	6.4622504	-2.9534214	-2.9534214
6.31174596	6.51311139	-2.9534214	-2.9534214
6.13141433	6.64713545	-2.9534214	-2.9534214
6.49977167	6.27474322	-2.9534214	-2.9534214
6.51204059	6.24184502	-2.9534214	-2.9534214
7.19883588	4.96702586	-2.9534214	-2.9534214
-2.9534214	-2.9534214	7.1678021	4.77633546
8.39116917	8.09239506	11.9131409	11.2204914
10.7769278	9.50581572	-1.8572999	8.31030239
7.93984242	6.59435877	-0.8115387	-2.9534214
6.04961209	6.55639888	-2.9534214	-2.9534214
7.8795679	8.66370572	13.1217699	10.2146485
5.95738047	6.58455207	-2.9534214	-2.9534214
-2.9534214	5.43918791	9.20071862	7.33071593
6.47395173	6.07676897	-2.9534214	-2.9534214
7.88538351	6.09875347	-2.9534214	-0.0551215
6.72808622	5.55921125	-2.9534214	-2.9534214
-2.9534214	-2.9534214	7.19625916	4.2417448
7.84305296	7.96779096	-2.9534214	2.64755465
0.02980441	-2.4449602	8.83615391	5.09050164
1.45678855	6.13615849	9.71647486	6.20055739
-2.9534214	-2.9534214	6.78621231	5.1032773
6.49216938	7.04472151	-2.3036717	-2.9534214
6.91025937	4.687475	-2.9534214	-2.9534214
6.97577889	4.58954551	-2.9534214	-2.9534214
6.58889656	5.50824933	-2.9534214	-2.9534214
-2.9534214	-2.9534214	7.25432149	3.2400335
-2.9534214	-2.9534214	6.98248202	4.27034749
-2.9534214	-2.9534214	5.56094691	6.58794626
-2.9534214	2.28537678	8.20893489	6.85198325
10.2144988	9.99263779	2.90181097	8.2765084
-2.9534214	-2.9534214	7.07340291	3.76714835
-2.4069576	7.30174634	9.912269	7.72408318
6.04442334	6.20177832	-2.9534214	-2.9534214
9.47211715	7.04591315	-2.9534214	6.47943104
6.17242428	6.02936091	-2.9534214	-2.9534214
6.82280417	4.68135366	-2.9534214	-2.9534214
-2.9534214	2.61578213	8.33799	6.45845416
6.4506062	5.58731014	-2.9534214	-2.9534214
-2.9534214	-1.5257463	7.74197582	4.76431974

-2.9534214	4.24402717	8.46546363	7.97276929
3.65012807	-2.9534214	9.97751882	7.20226259
-2.9534214	-2.4449602	7.37157228	4.89128977
-2.9534214	-2.9534214	6.10588706	6.07322893
6.43085879	5.55586922	-2.9534214	-2.9534214
3.90611811	-2.9534214	5.81167462	8.90604788
-2.9534214	4.69052595	9.31891157	0.50294321
6.4642703	6.8700475	-2.9534214	-2.2872455
6.89304299	4.11371847	-2.9534214	-2.9534214
7.13619539	3.08324495	-2.9534214	-2.9534214
8.48679643	8.64101758	-2.9534214	5.1032773
-2.9534214	-2.4449602	7.29739515	4.86527474
-2.4069576	-2.9534214	7.88330986	4.57092982
-2.9534214	-2.9534214	5.36404948	6.49647999
5.64924882	6.38713918	-2.9534214	-2.9534214
-2.9534214	-2.9534214	6.94599391	3.7181568
7.97606112	4.08154148	-2.9534214	-0.1734029
6.58978729	5.07369541	-2.9534214	-2.9534214
6.63536357	6.50186641	-2.9534214	-2.2872455
-2.9534214	5.40430599	9.02677597	7.10557135
-2.9534214	-1.5257463	7.49476809	5.44935696
8.89060273	8.95937841	4.01913607	6.01863778
-2.9534214	-2.9534214	6.81567049	4.21256355
-2.9534214	-2.9534214	6.23106434	5.56007248
6.72078623	4.52947552	-2.9534214	-2.9534214
-2.9534214	-2.9534214	5.99230062	5.82592141
5.2569162	6.42884204	-2.9534214	-2.9534214
6.46232619	5.01649171	-2.9534214	-2.9534214
5.73226426	6.07676897	-2.9534214	-2.9534214
-2.9534214	-0.96875	7.64842939	5.16551178
4.30797781	3.03605273	7.58362093	8.98172558
-2.9534214	-2.9534214	5.9258804	5.82592141
6.35534899	5.16864877	-2.9534214	-2.9534214
6.51579475	4.76766735	-2.9534214	-2.9534214
-2.9534214	-2.9534214	6.82317595	3.54168051
-2.9534214	5.3260316	9.07943377	3.6414014
6.27677436	6.40210535	9.84634693	9.4455119
6.3171345	5.17083512	-2.9534214	-2.9534214
6.77348098	6.15505578	-2.3036717	-2.9534214
-2.9534214	0.15185078	7.39568377	6.81944788
6.47008695	6.37865197	-2.3036717	-2.9534214
6.1712346	5.36092002	-2.9534214	-2.9534214
-2.9534214	-2.9534214	6.32921153	4.88760193
5.77361547	5.84724022	-2.9534214	-2.9534214
7.61618173	8.34562293	12.1139954	9.88021835
6.87196197	4.95438977	-2.9534214	-2.2872455

5.4240542	6.1226686	-2.9534214	-2.9534214
3.40497957	6.90000948	-2.9534214	-2.9534214
-2.9534214	2.30143067	8.24744901	5.29086398
6.05478225	6.86600514	-1.8572999	-2.9534214
6.31498149	4.83263067	-2.9534214	-2.9534214
7.02665938	6.74942367	11.0956349	9.23417373
6.55554675	3.9953467	-2.9534214	-2.9534214
5.85884732	6.50793228	-2.3036717	-2.9534214
-2.9534214	-2.9534214	6.74827666	2.91724899
5.40596176	6.13839459	10.6424556	7.74104293
5.98337145	5.3666536	-2.9534214	-2.9534214
-2.9534214	4.35201983	8.41296022	7.14162681
-2.9534214	-2.9534214	6.32921153	4.58009843
5.78454891	5.57745526	-2.9534214	-2.9534214
-2.9534214	-2.9534214	5.76761989	5.58533506
8.74447445	8.04371778	-2.9534214	5.81819305
11.2460875	12.1261216	6.54655771	9.99370726
6.30525303	4.67520624	-2.9534214	-2.9534214
5.95599947	5.34551812	-2.9534214	-2.9534214
5.11810471	6.1747985	-2.9534214	-2.9534214
6.50071915	6.340315	-2.3036717	-2.2872455
8.03578341	4.46323192	-2.9534214	1.55207983
6.54548142	6.18996931	-2.3036717	-2.2872455
-2.9534214	-2.9534214	6.41117885	4.10868549
4.98901575	6.19427469	-2.9534214	-2.9534214
-2.9534214	-2.0697503	6.40619009	6.00167875
-2.9534214	4.85727556	8.63149451	6.60264234
-2.9534214	-2.9534214	6.3553301	4.21256355
6.4555011	5.86219949	-2.3036717	-2.9534214
5.36492505	8.06826171	11.463615	7.83355494
6.26232141	4.47743295	-2.9534214	-2.9534214
5.99417647	6.91116845	-2.3036717	-1.8332537
6.48835316	3.58091222	-2.9534214	-2.9534214
-2.9534214	-2.9534214	5.04902415	5.96889681
7.28164921	3.17323639	-2.9534214	-1.8332537
5.99148279	6.20604866	-2.9534214	-2.2872455
5.13772779	5.10375907	10.1799737	7.37879455
8.22223926	8.4510276	-0.2116394	5.58760987
6.36266347	5.65134321	-2.9534214	-2.2872455
5.45566875	5.66226253	-2.9534214	-2.9534214
6.76404217	5.04060282	-2.9534214	-1.8332537
-2.9534214	-2.9534214	6.56009622	2.90270615
-2.9534214	4.26464531	8.43822936	6.4708304
6.04831665	4.82709638	-2.9534214	-2.9534214
-2.9534214	-2.9534214	4.99356139	5.77293319
-2.9534214	2.25272292	8.16494086	4.58466098

6.57096542	5.53734842	-2.3036717	-2.9534214
-2.9534214	-2.9534214	6.24230518	4.03057878
8.57107489	8.34852312	-1.8572999	6.16443937
6.02479758	4.7056847	-2.9534214	-2.9534214
-2.9534214	-2.0697503	7.0968142	3.16889429
5.32054209	5.67309983	-2.9534214	-2.9534214
5.74191258	5.03100667	-2.9534214	-2.9534214
6.29108397	6.73398078	-1.2416692	-2.9534214
0.18875116	5.61487221	8.60479844	7.6022464
-2.9534214	-2.9534214	5.94664499	4.38464608
5.93512419	4.77055351	-2.9534214	-2.9534214
6.03267995	6.03297106	-2.9534214	-2.2872455
4.91133903	-2.9534214	11.1212143	7.39317127
-2.4069576	-1.5257463	7.46748796	4.5241918
6.00088874	4.39005107	-2.9534214	-2.9534214
-2.9534214	-2.0697503	6.56121871	5.23681391
6.87999452	5.63718155	-1.8572999	-2.9534214
-2.9534214	-2.9534214	6.26865631	3.29674874
5.72741579	4.73553257	-2.9534214	-2.9534214
5.8138144	4.54304223	-2.9534214	-2.9534214
6.34694393	3.3611116	-2.9534214	-2.9534214
-2.9534214	-2.9534214	5.08068028	5.44183251
-2.9534214	3.32229932	7.88510383	7.20077735
3.88305033	6.1660573	10.6657983	5.94780194
5.77204676	4.57310874	-2.9534214	-2.9534214
-2.9534214	3.47165113	9.28330325	-2.2872455
6.35325231	3.19917447	-2.9534214	-2.9534214
4.90276296	5.63242993	-2.9534214	-2.9534214
5.85736865	4.3208561	-2.9534214	-2.9534214
-2.9534214	5.34745237	8.88381289	2.48134042
4.02151984	2.83014301	9.50657585	6.48432276
5.93231786	6.31186952	-1.8572999	-2.9534214
6.28119246	5.71565281	-2.3036717	-2.9534214
4.82622064	5.66070768	-2.9534214	-2.9534214
-2.9534214	-2.0697503	6.40619009	5.44935696
-2.9534214	-2.4449602	6.68068168	3.78311601
-2.9534214	-2.9534214	6.14084449	3.21670803
5.01046202	5.4873496	-2.9534214	-2.9534214
6.80143246	4.60256142	-2.9534214	-1.4884482
6.77661358	3.53412523	-2.9534214	-2.2872455
3.07934537	6.44157419	-2.9534214	-2.9534214
4.96175166	5.42460345	-2.9534214	-2.9534214
5.64582675	6.14174226	-2.3036717	-2.9534214
3.52159522	6.18565105	-2.9534214	-2.9534214
5.73226426	4.44888971	-2.9534214	-2.9534214
-2.9534214	-2.4449602	6.39867446	4.73177979

8.05364814	6.99869412	1.39300121	2.88801522
6.75612904	5.29027853	-2.9534214	-0.9773123
0.79434634	4.77343391	8.86029419	5.58077464
5.49423693	4.83263067	-2.9534214	-2.9534214
5.47700873	6.238722	-2.3036717	-2.9534214
4.93396239	5.40987004	-2.9534214	-2.9534214
2.28842343	-2.9534214	9.78184903	4.99433145
-2.9534214	-2.9534214	6.06056755	3.32973763
2.98499848	-2.4449602	9.28449313	5.92639403
5.72092572	4.41977033	-2.9534214	-2.9534214
2.55971076	6.24288453	9.43918247	6.34739341
6.06890539	6.47469235	-1.2416692	-2.9534214
5.71440631	4.30899393	-2.9534214	-2.9534214
5.8910004	3.90367309	-2.9534214	-2.9534214
5.15227191	5.13096485	-2.9534214	-2.9534214
9.20720171	7.32794313	-2.3036717	7.38338452
-2.9534214	-2.4449602	6.32525264	4.80398897
-2.9534214	-2.9534214	5.2545112	4.86902002
5.63551157	6.13167586	-2.9534214	-2.2872455
7.33998776	4.71769805	-1.8572999	-2.9534214
5.59171967	3.87713921	9.03123828	8.17615932
-2.9534214	-2.9534214	5.47779245	4.4758888
7.77693759	5.3987204	-2.9534214	2.02462691
6.41184502	5.20323879	-2.9534214	-1.8332537
5.73870364	4.05337823	-2.9534214	-2.9534214
-2.9534214	-2.9534214	5.37174718	4.63391918
4.87672456	5.30428797	-2.9534214	-2.9534214
8.13766663	7.21064291	-2.9534214	4.65142231
5.73064991	4.01495106	-2.9534214	-2.9534214
5.57921591	4.34041218	-2.9534214	-2.9534214
6.28119246	4.87347466	-2.9534214	-2.2872455
-2.9534214	-2.9534214	5.29285694	4.48079253
-2.9534214	-2.9534214	3.57050692	5.91737946
5.70457163	3.9953467	-2.9534214	-2.9534214
5.42605053	4.57970594	-2.9534214	-2.9534214
5.12549468	4.95184922	-2.9534214	-2.9534214
5.52622823	4.36353484	-2.9534214	-2.9534214
5.99148279	6.57054415	-1.5168928	-1.8332537
6.5436438	6.74722763	-1.8572999	-0.4437536
6.64567981	5.06431759	-2.9534214	-1.2103612
7.88792048	6.22405786	-2.9534214	3.06823308
4.28616277	5.5889461	-2.9534214	-2.9534214
-2.9534214	-2.9534214	5.68969926	3.76714835
5.87062243	6.11132987	-2.9534214	-1.8332537
-2.9534214	-2.9534214	4.48906098	5.1926647
6.7656196	3.42111796	-2.9534214	-1.8332537

5.55022229	4.1227816	-2.9534214	-2.9534214
5.51314115	4.21465761	-2.9534214	-2.9534214
6.44766126	5.50824933	-2.9534214	-1.2103612
-1.7015481	-2.9534214	6.68994786	5.45185642
8.17108146	7.85674372	-2.9534214	5.41390215
5.59704542	3.95023793	-2.9534214	-2.9534214
4.89124832	4.92100841	-2.9534214	-2.9534214
8.52780914	8.66331756	-2.9534214	6.43842864
-2.9534214	-1.1316325	6.41739064	5.77692569
4.39616093	5.37615922	-2.9534214	-2.9534214
3.92882286	5.69756929	-2.9534214	-2.9534214
6.7003125	4.36353484	-2.9534214	-1.4884482
5.97657699	5.35708493	-2.9534214	-2.2872455
-2.9534214	0.72812905	7.27704419	5.86395412
7.12824721	6.25117372	-2.9534214	0.84472723
5.27031239	6.25633038	-2.9534214	-2.2872455
5.38763955	4.14963431	-2.9534214	-2.9534214
0.93556977	4.87615691	8.46306352	6.77821691
5.12549468	4.4667953	-2.9534214	-2.9534214
-2.9534214	-2.9534214	5.69584487	3.13196448
6.2932729	6.07092746	-1.5168928	-1.8332537
4.27734355	5.2411863	-2.9534214	-2.9534214
-2.9534214	-2.9534214	5.37430399	3.63261538
9.25725639	7.65560376	-0.6366176	7.86905374
6.02874415	5.44281111	-2.3036717	-2.9534214
9.47344497	11.2393938	9.74082787	-2.2872455
6.59067747	6.74722763	-1.2416692	-0.3022547
4.80796504	4.60256142	-2.9534214	-2.9534214
3.73631579	5.52543744	-2.9534214	-2.9534214
4.57952363	4.71769805	-2.9534214	-2.9534214
-2.9534214	-2.9534214	5.25173282	3.48423469
-2.9534214	-2.4449602	4.37112722	6.68992628
4.63235338	4.54977803	-2.9534214	-2.9534214
-2.9534214	-2.9534214	4.98354861	3.75100198
-2.9534214	-2.9534214	5.20078468	3.39353517
-2.9534214	-1.7722575	6.40243717	4.82727728
7.64432077	7.5864505	10.6686002	9.78170715
1.02258002	2.70296801	8.1260252	6.42577006
5.18092752	3.89311802	-2.9534214	-2.9534214
4.32091024	4.77917751	-2.9534214	-2.9534214
-2.9534214	-1.7722575	5.6138345	6.26735377
5.10321023	3.89840521	-2.9534214	-2.9534214
4.52095449	4.48799274	-2.9534214	-2.9534214
6.11542074	4.69052595	-2.9534214	-2.2872455
-2.9534214	-2.0697503	6.51214496	3.79890887
9.12878132	7.12640968	1.77995038	7.23962874

-2.9534214	4.38252461	8.46875728	2.59424966
7.07198666	6.13727697	-0.0938779	-2.2872455
8.38605066	7.75804122	5.08380802	1.30906412
-2.9534214	-2.9534214	2.90181097	5.37492235
4.91133903	3.96038469	-2.9534214	-2.9534214
5.36075645	3.42844626	-2.9534214	-2.9534214
4.63580752	4.26464531	-2.9534214	-2.9534214
-2.9534214	-2.9534214	4.01258603	4.3529028
-2.9534214	4.32871037	7.78440739	7.71110083
5.15227191	3.58747391	-2.9534214	-2.9534214
9.3038694	9.37752049	-1.5168928	8.11281383
6.1326373	4.56648123	-2.9534214	-2.2872455
-2.9534214	4.12729191	8.43026501	2.22496144
5.54472051	3.03605273	-2.9534214	-2.9534214
5.01842289	3.64522363	-2.9534214	-2.9534214
8.51332875	9.37076055	2.00780786	7.29635958
6.51391889	5.23701887	-2.3036717	-1.4884482
8.24701244	8.77280427	2.08475918	6.54176514
7.53711439	7.17949319	0.74110411	2.63000425
7.23913449	6.57797705	0.21080887	0.33978981
5.3958115	3.11991784	-2.9534214	-2.9534214
-2.9534214	4.92100841	8.42565011	5.35101871
5.99013406	4.73257541	-2.9534214	-2.2872455
9.22221463	8.88325749	-2.9534214	7.6974741
5.67297869	6.42518355	-2.9534214	-1.2103612
5.28578565	2.95717935	-2.9534214	-2.9534214
6.30633722	4.54304223	-2.9534214	-1.8332537
3.28691672	5.15325089	9.03326205	6.72134272
5.76575482	5.19251823	-2.9534214	-2.2872455
5.64067838	5.40244653	-2.9534214	-2.2872455
-2.9534214	-1.7722575	5.6095005	6.10374787
6.98731686	5.60520443	-2.9534214	0.64950605
16.939857	16.2019056	14.4348377	15.5754116
8.18145918	7.83932405	-2.9534214	5.77493082
6.36994105	5.28018783	-2.9534214	-0.9773123
4.1430086	4.21889004	-2.9534214	-2.9534214
-2.9534214	-2.0697503	6.33184477	4.12130184
6.23979945	4.89214656	-2.3036717	-2.9534214
5.74671266	6.13167586	9.23593947	8.51568753
6.00088874	4.57970594	-2.9534214	-2.2872455
-2.9534214	2.65379444	7.77621368	5.47661565
-2.9534214	0.98825706	7.22487424	5.38542037
6.11418309	4.21889004	-2.9534214	-2.2872455
-2.9534214	-2.9534214	4.03214761	3.77515427
4.23242016	3.95023793	-2.9534214	-2.9534214
1.29143213	4.30899393	9.40348514	3.83765084

-2.9534214	4.47743295	7.95935634	7.21114209
8.14586608	6.84013709	-2.9534214	4.99775662
8.17286581	7.4445741	-2.3036717	5.66073462
6.75215614	6.59272894	-2.9534214	0.9043414
4.37564862	3.65774892	-2.9534214	-2.9534214
-2.9534214	1.62048238	7.36000886	5.76892962
7.14591802	3.00697818	-2.3036717	-2.9534214
5.31839417	5.56753259	-2.9534214	-2.2872455
5.80156437	5.49609465	-2.9534214	-1.8332537
6.49407371	6.51741315	-2.9534214	0.50294321
8.38091392	7.42776069	2.75257428	5.36434737
8.40160569	8.10588402	0.0149924	6.63159288
4.40022863	3.25794247	-2.9534214	-2.9534214
3.7992784	3.97547228	-2.9534214	-2.9534214
5.35657576	5.37236449	-2.9534214	-2.2872455
5.63895817	5.74236008	-1.8572999	-2.9534214
-2.9534214	-2.9534214	4.10168617	2.98784227
6.47298651	3.08324495	-2.9534214	-1.8332537
-2.9534214	1.69529321	7.19408997	6.34201375
4.29055225	3.24969219	-2.9534214	-2.9534214
-2.9534214	4.17599634	8.51351607	0.25072817
2.35699608	5.00673329	9.13080423	5.55310522
7.27335679	8.25372856	13.5784959	8.13670133
-2.9534214	-2.4449602	5.2236507	5.25981912
5.63205672	6.58208995	-2.9534214	-0.6006549
6.87049669	4.09541959	-2.9534214	0.15580464
-2.9534214	-2.9534214	3.8084137	3.16889429
6.53626979	4.53627482	-2.9534214	-0.6006549
-2.9534214	5.15103771	8.28156047	6.30378372
6.53903946	4.51233575	-2.9534214	-0.6006549
2.88404723	4.25230982	-2.9534214	-2.9534214
5.80463664	5.16426612	-2.9534214	-1.8332537
4.20012017	6.46492558	-2.9534214	-2.2872455
6.74177511	5.38183268	-2.3036717	0.42367165
8.51191968	7.67691217	-2.9534214	6.56272977
-2.9534214	2.40906789	7.71550139	4.35824208
5.84548442	5.49783731	10.2199692	7.35829014
7.78745782	7.75876813	-2.9534214	5.10645361
7.10662805	4.99690842	-2.9534214	1.26426187
6.57456952	5.81963768	-0.3398746	-2.9534214
-2.9534214	5.41909604	8.3926105	5.70121431
6.07145845	5.42093418	-2.3036717	-1.4884482
9.88206833	11.2399471	7.6740779	8.70569071
7.3719248	6.56473641	-2.9534214	2.85817682
5.6628565	4.21465761	-2.9534214	-2.2872455
3.12921138	-2.9534214	6.52600872	7.72408318

5.50182837	5.62925347	-1.8572999	-2.9534214
8.05751667	8.4635663	-2.9534214	6.173554
-2.9534214	6.75234652	8.87526391	3.27432947
-2.4069576	4.70266566	8.30821371	4.30393859
6.18780155	4.99197078	-2.9534214	-0.9773123
8.5820833	7.86689318	-2.9534214	6.86141449
6.3730488	5.95899367	-2.9534214	0.42367165
5.73709649	6.059173	-0.8115387	-2.9534214
2.88404723	3.66397101	-2.9534214	-2.9534214
6.71017616	6.50879676	-2.9534214	1.35251687
-2.4069576	0.7748727	6.53746094	6.13829741
6.44667828	4.13178815	-2.9534214	-0.7767262
-2.9534214	3.24139446	7.65264817	6.0437083
9.14220159	9.61803036	-1.5168928	8.31374172
5.85736865	3.15568158	-2.9534214	-2.2872455
5.68135993	5.35708493	-1.8572999	-2.9534214
7.13558554	5.75989414	-2.3036717	1.85370754
4.91702822	5.28625071	-2.3036717	-2.9534214
6.21807294	4.86539784	-1.8572999	-2.2872455
-2.0115616	5.11742609	8.25683241	5.4215733
-2.9534214	2.21931282	7.15967321	6.44597082
6.3321162	6.02694912	9.44086051	8.39619044
7.84267937	9.41062183	5.21226299	6.27018896
5.76890422	6.11927633	-0.8115387	-1.8332537
0.79434634	3.02642617	9.69847858	2.29345235
-2.9534214	5.26798508	8.29545461	5.19565039
5.25915756	5.43191411	-1.8572999	-2.9534214
5.90251701	5.1421509	-2.9534214	-1.2103612
0.02980441	-0.4557187	7.7853683	4.73177979
5.48469112	4.06752858	-2.9534214	-2.2872455
-2.9534214	4.72664287	8.05489149	6.17959858
5.55022229	5.24534173	-1.8572999	-2.9534214
-2.9534214	6.5804462	8.54449511	6.85103672
6.00222746	4.7531493	-2.9534214	-1.2103612
-2.9534214	0.15185078	6.55334284	4.89128977
6.13751881	7.70697839	9.74910132	8.95069259
5.06528706	5.05013556	-2.3036717	-2.9534214
5.74830914	4.74142875	-2.9534214	-1.4884482
-2.9534214	-2.0697503	5.80221128	3.57874506
6.94687132	7.37989673	-0.2116394	2.94590212
5.20670437	4.33262153	-2.9534214	-2.2872455
5.43599099	5.09917447	-2.3036717	-2.2872455
-2.9534214	1.62048238	7.33856548	2.87317316
5.67801327	8.09268338	9.8026424	8.86303235
6.23639063	6.38054234	8.70924729	9.13772938
5.96701066	5.02859763	-2.9534214	-0.9773123

7.94958999	7.10541736	-0.6366176	5.44685317
3.27810212	-2.9534214	7.5574224	7.15779202
-2.9534214	5.98656113	7.95295622	8.12695915
6.87561867	5.86625251	-2.9534214	1.76000118
5.26808831	5.87968094	-1.5168928	-2.2872455
6.12773921	5.49783731	-2.3036717	-0.3022547
6.15688135	4.96953985	-2.9534214	-0.4437536
8.21677196	6.87273613	-2.9534214	6.04536428
5.85440676	5.92312832	-2.3036717	-0.3022547
-2.9534214	3.71280473	7.76115134	5.44183251
7.07771248	5.67155662	-2.9534214	2.15305622
6.42587941	4.69660858	-2.9534214	0.15580464
7.47193776	4.74730093	-1.8572999	2.35883854
6.36786548	5.05250893	-1.0106329	-2.9534214
8.09187445	7.46119381	-1.5168928	6.28287885
2.21642809	-2.9534214	7.1183272	6.80973732
6.02216053	7.08353495	1.92651965	-2.9534214
-2.9534214	3.92455433	9.13721642	-2.9534214
6.9825771	4.02465423	-1.8572999	0.42367165
10.2589253	10.8330578	-1.8572999	9.72587697
7.77145231	6.07443521	-2.9534214	4.34754367
5.52250116	4.21889004	9.73325384	6.8453445
8.45048002	8.04937323	-2.9534214	7.05878683
7.93984242	7.60556813	-2.9534214	5.89183481
6.36994105	7.31317323	-2.9534214	2.12826873
6.23069129	5.23074506	-2.9534214	0.15580464
7.82008485	8.07703127	-2.9534214	5.99483892
6.69866198	6.69425456	-2.9534214	2.1030479
7.55683573	7.7875484	0.0149924	5.26835236
-2.9534214	7.27557307	8.53109517	8.63073499
4.45600398	4.72068582	-2.3036717	-2.9534214
-2.9534214	2.26914223	6.96732007	6.33796579
5.62685889	4.77055351	-2.3036717	-1.8332537
5.90681215	3.7944775	-2.3036717	-2.9534214
1.18228212	-0.6895489	7.11297895	6.2079695
-2.9534214	3.40634873	7.92008526	1.55207983
6.71752989	4.56648123	-1.8572999	0.33978981
6.38848796	0.82014925	10.6750306	7.21850016
-2.9534214	5.60843423	8.5436435	0.84472723
6.77191213	4.20615531	-2.9534214	1.21802358
5.45371301	7.60839388	-2.3036717	1.39469903
7.22372381	5.13992062	-2.9534214	2.55758639
5.32910193	4.2356967	-2.3036717	-2.9534214
8.84283413	8.66603254	4.32429212	7.68690371
5.95461714	3.32229932	-2.9534214	-1.2103612
-2.9534214	-2.4449602	3.6401495	5.27965208

4.00558836	6.58455207	-2.3036717	-1.4884482
5.27917456	4.91840834	-2.9534214	-1.4884482
4.80796504	5.46792185	-1.5168928	-2.9534214
8.51473645	7.60556813	11.5658925	9.49437079
6.86167361	3.26614584	-2.9534214	1.30906412
5.87355125	5.62925347	-2.9534214	0.05419288
-2.9534214	6.47822758	8.40015771	6.16443937
-2.9534214	7.45223397	8.61104475	8.15981759
-2.9534214	5.42276998	8.27576001	3.35131846
5.61640669	4.8408925	-1.5168928	-2.9534214
7.68156272	7.05422716	-1.8572999	5.22517226
6.28998825	3.86638749	-1.8572999	-2.2872455
9.12740173	8.30464474	6.92347513	5.00458266
2.92978863	2.00076476	6.48164183	7.88023133
6.85872055	5.2761317	-2.3036717	2.12826873
8.25377222	5.54579645	-2.9534214	6.12426337
7.23287592	8.16520766	-1.5168928	5.47169768
3.63639923	5.433736	-2.9534214	-2.2872455
5.57742078	5.27409936	-1.0106329	-2.9534214
8.02528668	6.86465517	-2.9534214	5.94247967
-2.9534214	5.38371888	8.03644531	6.11798192
5.72903376	4.94164203	-1.2416692	-2.9534214
9.8098471	12.389017	6.28233439	9.77098788
8.07193198	9.14514239	5.06810106	6.8155715
2.99579136	-2.9534214	8.92297894	5.13783798
10.081897	8.88741806	6.92783305	7.91732884
7.71419613	8.24726818	-1.0106329	6.44471653
5.52622823	7.42318269	-2.9534214	1.43568281
4.73897916	4.65660523	9.38664584	6.35141503
-2.9534214	3.08324495	7.30679491	5.46676289
5.27696412	4.1976026	-2.3036717	-2.2872455
7.51992637	7.72420093	-2.9534214	5.40876529
6.62323398	6.37486379	-0.8115387	1.88363757
-2.9534214	3.65774892	7.80634807	2.0512437
-1.7015481	2.09588014	6.52024833	6.53588716
8.63438948	9.37052278	2.70461738	8.26695925
7.55318699	8.32956678	2.29367983	6.04039063
6.1067348	7.36851209	-2.9534214	2.24815491
-0.1488474	-2.9534214	6.85097873	4.76833611
2.15997119	3.6824779	8.1941776	6.01018319
7.87665128	8.61636357	1.17569549	6.93293573
3.20557665	5.10375907	-1.8572999	-2.9534214
7.06110883	4.60256142	-2.9534214	2.59424966
5.12057224	4.23151336	-2.9534214	-1.4884482
4.98360399	3.64522363	-2.3036717	-2.9534214
-2.9534214	5.01649171	8.02875687	4.62951001

-2.4069576	4.91580357	9.38014096	-1.4884482
-1.7015481	4.46323192	8.44341315	1.88363757
6.20068627	6.86330393	-2.9534214	2.07737831
5.18092752	5.80140861	-2.9534214	-0.6006549
3.75549476	-1.5257463	7.69927839	6.81847976
4.80489985	5.08068899	-1.8572999	-2.2872455
6.4047763	7.81016625	-2.9534214	3.444626
5.41604111	5.41909604	-2.3036717	-0.6006549
-2.0115616	-1.5257463	5.17461963	5.23100482
-2.9534214	1.39965331	6.44075448	5.29643742
8.52245432	8.31555483	-2.9534214	7.71318587
3.00650411	4.93651123	-1.8572999	-2.9534214
-2.9534214	5.37994401	8.1941776	2.87317316
6.08289162	5.82381188	-2.9534214	1.17025416
-2.9534214	6.56307276	8.41327106	4.62951001
-2.9534214	4.42710546	7.43968203	6.84248995
7.6038891	3.70075039	-0.2116394	-2.9534214
-2.9534214	3.41375224	7.04640258	6.74089168
-2.9534214	2.58987252	7.17148197	3.11944144
6.43482995	5.71565281	-1.8572999	1.85370754
7.35001139	9.09982003	3.67374938	6.32847616
3.60854328	3.56104615	-2.9534214	-2.2872455
-2.9534214	7.09566887	8.57036992	4.56170256
-2.9534214	4.88683634	8.18145937	0.9043414
3.06916195	3.74837496	8.21607915	6.43084683
4.61495753	3.18193433	8.41885478	6.92486194
5.23202872	3.85555504	-2.9534214	-1.2103612
6.22496936	4.23151336	-1.2416692	-2.9534214
5.49423693	6.69044972	10.1298818	6.82427875
-2.9534214	5.21175825	8.07113618	3.79890887
-2.9534214	4.05811045	7.49123893	5.85075642
6.03136921	4.58299326	-2.9534214	0.33978981
1.66043892	-2.0697503	8.28631987	4.42591237
8.2063557	6.35769281	1.39300121	5.76290335
2.96316742	5.29830058	-2.9534214	-2.2872455
4.79874987	3.91415149	-2.9534214	-1.4884482
6.7648311	7.60556813	10.6528256	8.05027468
9.4435774	9.56851754	7.13123386	7.92409529
10.4952286	11.0920863	8.54506257	9.35693855
5.87793336	5.3742631	-2.9534214	0.57808499
7.67695971	7.91692199	-2.9534214	6.16900388
6.63708808	5.3987204	9.04734949	8.19231802
9.68630573	9.32805333	7.58472527	7.47838696
7.26780191	5.02134627	-2.3036717	3.43455156
5.72903376	3.24139446	-2.9534214	-0.6006549
-2.4069576	-2.0697503	3.99939605	4.73998384

6.25335477	5.31816269	-1.5168928	0.96158967
7.51195998	7.7301435	-2.9534214	5.73649465
4.62194106	5.21388028	-2.9534214	-1.2103612
-2.9534214	5.48559421	7.79303265	6.75816111
4.65295524	5.05487841	-2.9534214	-1.2103612
7.32133889	7.52111671	-0.0938779	5.17159008
8.81053225	9.88589113	3.73072955	8.67980357
2.96316742	4.4949899	-2.9534214	-2.2872455
-2.9534214	4.90007541	8.09264449	1.1208487
6.13141433	6.7138796	2.24979738	-0.7767262
5.57021784	3.78308925	-1.8572999	-2.9534214
7.34685355	6.86059765	-1.5168928	4.79220231
-2.9534214	4.85998807	7.72654981	5.70959352
-2.4069576	6.29593201	8.02387982	6.62385548
7.25606658	6.94285789	-2.9534214	4.55706671
1.45678855	-2.9534214	6.87103221	5.74467202
5.78299206	3.49277786	-1.8572999	-2.2872455
-2.9534214	4.15847529	7.19336618	6.82041535
-2.9534214	5.70663938	7.95551967	6.06997853
-2.9534214	3.16448568	6.93130993	5.9810612
7.67360282	6.31384939	-2.9534214	5.21931602
-1.7015481	-2.9534214	3.20731949	4.48079253
-2.9534214	3.91936229	6.70830355	7.59378384
5.85736865	3.8001381	-1.8572999	-1.4884482
3.43701025	2.02029613	8.48333833	5.66720215
4.08859285	4.13178815	9.07629652	5.76892962
2.90709921	4.79627238	-1.5168928	-2.9534214
7.74930021	8.26605197	5.45864424	4.93124571
-2.0115616	5.55251944	8.04087786	4.78825193
5.37735909	3.10169792	8.14524003	7.50811299
2.88404723	5.14882114	8.15493821	6.84248995
7.73568287	7.4149055	-1.2416692	6.35275309
7.53942147	5.13768688	-2.9534214	4.50506423
6.80220121	3.11083664	-1.5168928	-0.0551215
7.98285801	7.43505541	-2.9534214	6.73474689
4.87964103	3.70679015	-1.8572999	-2.9534214
5.0780392	6.1033394	-0.2116394	-0.9773123
8.15522314	7.33234478	3.3902412	5.91737946
5.47315213	5.89298553	9.25756699	7.19183363
7.62620148	7.32500122	-2.9534214	5.84886114
9.87560034	10.2769332	7.98509489	8.55487116
4.12345771	4.34041218	-1.5168928	-2.9534214
2.86062093	4.98453248	-2.3036717	-2.2872455
-0.2472184	5.30627827	7.90469171	6.08293642
6.76955564	4.9594575	-2.9534214	2.88801522
7.91979329	7.87261301	0.11622035	7.03731166

6.71099509	7.03094647	-2.9534214	3.65013424
6.05993395	6.95812891	-1.8572999	2.61223772
-1.7015481	-2.9534214	4.51246228	3.96909215
7.4092379	6.13839459	9.30121727	8.77943334
5.92668876	6.07092746	-2.9534214	1.65978297
4.83826395	5.08068899	-0.8115387	-2.9534214
3.24229507	2.42380945	7.91482595	6.39492844
6.89880464	6.93837322	9.92027408	8.23139482
5.52250116	4.82986618	-2.9534214	0.15580464
5.88955434	5.795753	-2.9534214	1.43568281
6.6745148	5.40058467	-2.9534214	2.85817682
7.15135835	7.50170719	-2.9534214	4.98054886
1.83886594	5.77002479	8.46306352	5.71168074
7.6643309	6.94029693	-1.5168928	5.89551185
8.25994098	7.33087905	-2.9534214	7.33207331
-2.9534214	3.31440997	7.14102633	4.08955166
-2.9534214	5.00673329	7.50413719	6.74802767
6.70771656	5.85269792	-2.9534214	3.05514185
7.88211511	5.67925621	5.42697596	13.6865775
3.57294953	3.63891997	-2.9534214	-1.8332537
-2.9534214	-1.7722575	3.34924049	5.40102555
7.02731704	2.98726444	-2.9534214	3.09406445
5.0498342	4.16726241	-2.9534214	-0.6006549
7.13802338	4.97455473	-2.9534214	3.65013424
-0.7256766	1.17410899	6.75614008	4.63831491
7.51711972	5.75261416	-2.3036717	4.8464011
-2.9534214	1.51428893	5.74803792	6.27160448
5.82141818	7.3546347	10.2614777	7.13308647
6.48548435	5.17737434	-1.0106329	1.58888154
-2.9534214	4.73257541	8.67232382	-2.2872455
3.39685954	3.89840521	-2.9534214	-1.8332537
-0.4667031	3.0739292	6.63982596	6.75917054
5.7241744	7.92439228	-1.2416692	3.37258123
7.55227337	6.18996931	11.1249199	7.94730959
-2.9534214	3.92455433	8.82755016	-2.9534214
6.95449851	6.06858422	9.62737975	8.08611276
7.6205467	8.03714154	-2.9534214	6.54878726
6.41486391	7.41720949	9.51195565	8.40846684
9.82996502	9.71728981	7.36194254	8.60990331
6.73615423	5.37615922	1.47151796	-2.9534214
8.23368208	9.25509874	5.06810106	7.60561757
4.42039683	5.63718155	-2.9534214	-0.6006549
7.6702381	7.41905003	-1.0106329	6.41428158
8.49704073	7.10598876	3.80083084	6.42067536
5.5934971	5.84997165	-2.3036717	1.17025416
4.43633082	6.82007885	-2.9534214	0.50294321

3.54382842	5.10832914	-2.9534214	-1.4884482
6.32785157	5.40058467	-2.3036717	2.27098141
6.32357429	5.07603037	9.5719359	7.23600789
-2.9534214	5.30428797	7.39757026	7.29078584
-2.9534214	4.53966249	8.98890432	-2.9534214
6.51579475	6.22300469	-1.8572999	3.10680874
-2.9534214	5.70362235	7.78968451	6.29545808
1.32605442	-2.9534214	7.26744793	5.01476155
7.84155801	5.9373248	-2.9534214	5.89917955
-2.9534214	5.30229493	7.73353683	5.58305665
6.60572716	6.31285979	-2.9534214	3.26298783
-2.4069576	5.44642523	8.15158852	2.1030479
8.02660295	7.75658629	1.3099641	7.17831322
6.17598746	5.08764883	-1.8572999	1.55207983
3.90038566	4.76477541	-2.9534214	-1.2103612
7.93984242	7.16695205	-2.9534214	6.85292915
8.34469922	8.55344836	-2.3036717	8.14946108
8.59056298	6.80818959	10.4874572	9.28971881
7.71215246	6.90723984	-0.6366176	6.15065854
8.04523056	7.07188318	-1.8572999	7.28097967
-2.9534214	4.23986795	7.02540884	6.71198941
6.48930816	5.02134627	0.74110411	-0.9773123
9.42010666	9.56322186	5.14797126	9.26078881
3.96222373	-2.9534214	7.41132962	6.72857582
-2.9534214	5.09457526	7.72003143	4.43604712
-0.7256766	5.02134627	7.63676372	5.8807471
5.83800676	4.61866789	-2.9534214	1.17025416
8.37395019	6.75599178	-2.9534214	7.73387222
7.41226224	6.85516981	-2.9534214	5.38803298
6.1067348	6.39931099	2.73676511	-2.9534214
-2.9534214	3.47872781	6.94341346	4.46107681
6.13385922	6.7183708	-2.9534214	2.97399725
-2.9534214	4.69052595	7.48415461	5.24836237
6.72241164	6.96822052	-2.9534214	4.07016065
7.66263867	7.92309588	-2.9534214	6.7891299
6.21576679	7.17132656	-2.9534214	3.40389911
6.64653618	5.32406839	-1.5168928	3.08120658
6.32998546	5.46079184	-1.8572999	2.48134042
7.6685528	6.47822758	-2.9534214	5.86019565
-2.9534214	6.50446919	8.21857131	2.35883854
-2.9534214	3.72475918	6.83064257	6.2516598
6.2111434	5.62287949	-2.3036717	2.51996704
7.77850099	7.4709858	3.75447713	5.35101871
3.72986571	3.96543144	-2.9534214	-1.2103612
7.17529241	8.10731162	0.3831957	6.18861824
8.38476819	7.8272465	-2.9534214	8.05439231

7.32722598	6.91051443	0.53716524	5.04155929
6.09924786	4.88417389	-2.3036717	1.7919177
-2.9534214	3.62622942	6.75614008	6.19460016
7.29481872	7.21170513	-2.9534214	5.49854138
10.293097	10.8090891	7.89314923	9.72652223
6.02742983	4.50542242	10.523249	6.25595693
-2.9534214	6.03176868	8.04087786	3.2400335
6.45256615	8.24545407	10.7639385	7.63395887
12.3504661	12.9625418	10.3041738	11.961698
-2.9534214	3.19917447	6.38859232	6.47820551
9.89797774	9.90915909	7.26607181	9.07324357
-2.9534214	4.32871037	7.14102633	6.06835058
4.32091024	4.39005107	-1.0106329	-2.2872455
6.08920444	5.50305272	-2.9534214	2.29345235
7.05596133	6.99437996	-2.9534214	4.89863728
3.29567779	4.55313418	8.85938193	5.08728996
9.83128374	8.48041066	12.0880188	10.2701394
7.95167022	6.8700475	-2.9534214	7.01128172
5.6372359	3.44299212	-2.9534214	0.64950605
6.64139034	7.03575272	-2.9534214	4.14620829
6.58443462	5.14882114	-2.3036717	3.28558265
4.57952363	7.8066555	-2.9534214	2.02462691
5.55387852	4.31295883	-1.0106329	-0.9773123
5.14744014	6.71986476	-2.9534214	1.8231434
7.05273479	6.82633372	-2.9534214	4.80007078
7.58659077	6.72953799	-2.9534214	5.95838793
5.77518247	4.61866789	-2.9534214	1.39469903
8.40312667	9.31699095	0.21080887	8.68592097
8.09470101	6.95178561	-2.9534214	7.46978009
2.12106726	5.10832914	8.39387112	5.23681391
-0.7256766	2.23611458	6.54315314	5.18967283
5.12549468	5.88501751	9.35883556	6.45969657
5.57021784	4.59606815	7.58472527	8.14367518
-2.9534214	2.75052062	6.33971577	5.52489472
5.25467136	5.24534173	-1.5168928	0.33978981
7.35263765	6.97511778	-2.9534214	5.59893044
5.62338323	5.85269792	8.36940869	7.978848
7.3383987	7.57370092	0.86262618	5.94425594
7.52970685	6.33837115	-2.9534214	5.67578065
5.76417754	6.77120207	-2.3036717	2.87317316
8.90641881	10.0837839	5.78117083	8.84508023
8.16242587	7.87362004	-2.9534214	7.79961564
-2.9534214	4.48799274	6.9588274	6.77422799
10.0792038	10.362214	9.25427405	7.87558452
6.45843009	6.25941555	-2.9534214	3.49396925
7.1386322	0.82014925	-2.9534214	3.5132435

7.89802401	5.21811499	-1.2416692	6.10056558
5.99552142	5.84860658	-2.9534214	2.48134042
1.02258002	0.67982005	6.63663476	5.26266914
-2.9534214	4.64721401	7.32673336	5.30752013
7.69072491	6.67050113	0.11622035	6.14294526
4.9507001	4.59606815	-2.9534214	0.25072817
-0.1488474	4.84637421	8.04609882	3.9829841
8.41849965	7.37136669	10.9896198	8.92145925
-2.9534214	3.58747391	7.23123516	0.57808499
7.13619539	7.96779096	-2.9534214	6.03872893
7.14954719	4.6597221	-2.9534214	4.34216457
7.50678173	8.37720889	-2.9534214	6.94629243
-1.7015481	5.22444384	7.4366271	6.05526043
5.19738417	5.74676361	9.00817021	6.87730744
11.3947987	11.0890548	9.44497108	10.0729692
7.70723581	7.37800552	-1.2416692	7.01551199
7.8645575	4.12729191	-2.9534214	6.10056558
4.38388857	4.17599634	-0.6366176	-2.9534214
-2.4069576	4.25643338	7.27225404	3.96209569
6.45158651	7.24736314	0.53716524	3.95506514
7.36621767	6.88809925	-2.9534214	5.66935156
-2.9534214	-1.1316325	3.552558	3.50363856
-2.9534214	4.12729191	6.93304524	5.99483892
-2.9534214	4.11371847	7.0229667	5.29086398
3.17741124	4.31691287	-2.3036717	-1.4884482
9.45045899	8.73968078	10.660047	10.809216
5.0498342	-2.9534214	7.42988339	7.1268434
7.08783593	5.63084257	-1.2416692	4.26467214
8.28957313	8.12630641	3.72272602	7.66606599
5.02370583	5.19681602	0.53716524	-2.9534214
-2.9534214	3.94001929	6.72141669	6.40657145
7.78745782	4.84363596	-2.9534214	6.06997853
4.73897916	6.51052416	8.43517135	7.67465124
5.94907455	6.45778071	2.8439553	-0.3022547
5.60587822	5.00428334	0.67627567	-2.9534214
7.19239963	6.12379759	-2.9534214	4.94904802
-2.4069576	7.81855736	8.19959413	7.23092337
3.17741124	4.76477541	-1.8572999	-1.4884482
6.11912734	5.45182948	-2.9534214	2.71569611
7.27501909	6.48263446	-2.9534214	5.36434737
11.3867491	9.38695502	8.85869735	8.36138312
8.19674095	6.73545867	3.1724863	6.28428197
8.32450711	7.56375231	3.53438297	7.32049466
1.9759955	6.97010486	8.26993613	6.65564375
5.90108245	5.0968767	-2.9534214	2.02462691
6.30308221	6.3927697	-1.8572999	3.53226365

4.53211885	4.45248865	-2.9534214	0.05419288
10.3667894	9.47871666	8.49336987	8.02153814
6.9613975	4.6597221	-2.9534214	4.12130184
4.84125914	5.96782241	-2.9534214	0.9043414
5.27696412	5.73646771	7.94179497	8.0731835
3.46834521	-2.9534214	7.14327672	6.32029222
-2.9534214	5.66070768	7.58527713	5.88260098
6.12159312	6.11246776	2.8439553	-2.9534214
7.97606112	6.63926203	-2.3036717	7.39382137
7.37244252	5.08996134	-2.9534214	5.03156838
6.99809248	6.40210535	-2.9534214	4.69004478
6.52978647	6.07092746	-2.9534214	3.76714835
-2.9534214	3.85010816	6.79196104	4.7842907
7.29645649	6.38148659	-2.3036717	5.52726678
7.38327179	6.40953062	-0.8115387	5.53435965
5.19270139	4.85727556	-2.3036717	0.78254314
4.39616093	7.80348851	-2.9534214	2.1030479
8.0393741	6.00995271	-2.9534214	7.33275152
6.78752441	5.13096485	2.00780786	-2.9534214
7.47531568	7.83278022	0.0149924	6.88287517
-2.9534214	4.95184922	7.33200406	5.57848902
7.24762538	7.8420705	-2.9534214	6.3189237
7.56047525	5.76569179	-2.9534214	5.84696336
17.3629952	16.8689722	15.7544826	16.5405087
6.28998825	3.97046059	8.85435412	7.22582088
5.01046202	6.05207408	8.33635179	7.61233637
10.5015729	12.0321802	8.14186772	10.6818603
8.37575883	5.79716898	3.77784014	4.25897437
6.54088296	3.85555504	-2.9534214	3.2284179
7.07389779	7.21541677	-0.6366176	5.52013887
5.62685889	7.40239999	9.32668825	7.5378272
3.67719993	2.72694023	8.00296642	6.02871833
6.23525256	4.40126766	-1.0106329	1.55207983
-2.9534214	5.39123914	7.46569078	5.5646987
-2.9534214	4.77630857	6.82877954	7.10159742
6.94408777	4.62186772	-2.9534214	4.23595558
7.20116923	7.08990354	5.1539358	-2.9534214
1.71370892	5.34551812	7.43540331	7.07103248
-2.9534214	7.07012727	7.88555198	6.84914181
5.88810682	4.08618237	0.0149924	-2.9534214
6.42986429	4.61866789	-2.9534214	3.2284179
0.52309424	6.57962362	7.94437832	6.34336054
4.43633082	3.6824779	-2.9534214	0.05419288
5.81533836	4.03429258	-0.8115387	-0.1734029
-2.9534214	5.4373729	8.60180148	-2.2872455
-2.9534214	4.34429178	7.48947111	0.25072817

7.81780581	6.1473045	-2.9534214	6.80485743
7.03453161	7.49648691	0.0149924	5.66289369
6.85650178	6.99930937	-2.9534214	4.96663333
7.00278162	5.31816269	2.58609983	-2.2872455
-2.9534214	4.32871037	6.7881311	6.40140838
7.10600557	6.74209052	-2.9534214	5.34565257
6.7867478	3.58747391	-0.48063	1.58888154
2.87238163	3.83915232	-0.8115387	-2.9534214
-2.9534214	3.67633528	6.41490913	5.90283794
-2.9534214	4.01495106	6.95028444	2.97399725
-2.9534214	4.35586839	6.85647556	6.09737626
9.26633817	9.18015809	7.51692116	7.68424897
6.92020819	8.20895058	-2.9534214	6.22121607
8.34575417	6.35673285	5.37685627	0.64950605
5.25017117	-2.9534214	8.2274369	6.57882754
3.6637275	-1.3152731	7.13877242	6.21092371
4.41236335	3.61342625	-2.9534214	0.15580464
7.87482539	5.44281111	-2.9534214	6.86985021
-2.9534214	6.15063158	7.73254075	5.20457055
8.73141679	7.7714302	7.62285429	1.72736257
7.1841663	8.01510309	-2.9534214	6.55228554
6.40578824	4.63776126	-2.9534214	3.35131846
7.65286966	8.64475826	5.20940196	6.57080111
2.10121467	5.35516356	7.56918696	6.84248995
8.5196526	9.54426888	-2.9534214	8.86420562
8.89295014	8.63943965	7.8157844	5.88999276
5.63205672	7.72457305	-2.9534214	3.79103405
7.72761221	7.38743689	-2.9534214	7.25043709
-2.9534214	2.00076476	5.28200462	6.16748398
7.84080995	6.93194227	2.81413349	6.13363455
8.93122879	7.1099822	12.8289144	8.57335379
7.63701416	7.51166023	-2.9534214	7.13464302
3.69717557	3.29047973	-1.0106329	-2.9534214
8.4583046	8.15143446	11.3830689	8.88493166
-2.9534214	5.26389445	7.51518455	3.5510363
6.4594051	4.69052595	-2.9534214	3.50363856
4.31230151	5.45900381	-1.0106329	-0.1734029
6.6275777	6.45688511	-2.9534214	4.3741424
5.11067669	-2.9534214	8.34028038	6.33931637
5.73064991	4.01495106	-2.9534214	1.88363757
-2.9534214	4.82154078	7.02946989	6.09097639
6.82431868	7.05185661	-2.9534214	5.12223114
9.46885277	9.6753634	6.55897285	9.19125187
5.10570335	4.51921611	-2.9534214	1.1208487
-0.7256766	-2.9534214	5.6138345	3.37258123
6.72403523	6.11019108	-2.3036717	4.50506423

7.69776515	7.08179318	9.30674917	8.97413837
-2.9534214	7.05363489	7.64895741	7.5194766
6.73454423	3.17323639	-2.9534214	3.83765084
-0.7256766	2.52298655	6.19680657	5.34833813
6.88653351	7.19944208	-2.9534214	5.41133601
-2.9534214	4.23151336	7.18901582	0.71755754
8.08557332	9.43481663	-1.5168928	8.60513935
8.40312667	8.6555322	-2.3036717	8.85101023
7.68281554	7.72754664	4.61978935	5.76692366
0.69188115	-2.4449602	5.44167851	4.70269254
5.56297875	5.66536722	2.08475918	-2.9534214
5.43003494	3.34571176	-1.8572999	0.42367165
6.50921855	7.82516588	-2.3036717	5.42412132
8.36200808	8.88359078	-2.9534214	8.69834166
6.57096542	7.31416261	8.94443041	8.52610263
-2.9534214	6.32665271	7.72654981	5.11908941
4.83826395	3.03605273	-1.2416692	-1.4884482
7.65286966	7.52838176	-0.8115387	7.46236168
2.17903669	3.24969219	7.96445607	4.81179359
7.38942364	5.16206979	-2.9534214	5.62574279
4.2817599	6.56307276	8.48600053	6.99337786
6.96894848	6.340315	-2.9534214	5.07112348
5.86768765	6.96318354	8.45644255	8.33522295
-2.9534214	4.65035121	7.48651991	0.15580464
9.52220105	9.79011957	11.426832	10.8003558
8.46122791	7.89460692	7.74395431	2.1030479
-2.9534214	7.07597202	7.93011231	5.46428915
5.74351438	6.31285979	2.82912145	0.15580464
5.14744014	-2.9534214	8.46875728	6.1826114
3.91751515	6.00137882	9.40536145	4.98400687
10.2974655	11.0468992	9.19476397	9.34354094
7.83180286	8.07148341	-2.9534214	7.8054908
7.4372215	7.50647591	-2.9534214	6.87171813
9.7192403	9.21182458	11.9685767	10.2289275
6.30308221	6.06153157	-2.9534214	3.9409004
4.92833972	4.08618237	8.06363089	6.88009399
3.11937512	-2.9534214	7.08827411	5.70750327
0.69188115	2.93676833	6.5544706	5.63016346
7.60212443	8.10531258	10.0577502	8.87890759
5.74671266	8.18616451	0.21080887	4.66441169
6.71263157	6.26761057	3.08778101	1.55207983
7.01277876	6.48702792	-2.9534214	5.33486004
6.42488147	4.20615531	0.53716524	0.25072817
6.74817227	8.0466971	2.20453818	5.72620734
-2.9534214	4.58954551	6.93823866	4.8949682
7.28385249	6.48087332	-2.9534214	5.96715064

8.92947073	8.9664753	10.9792647	9.97471379
-2.9534214	5.75698656	7.66888042	2.12826873
6.39970592	7.15485329	-2.9534214	4.64706639
4.65975767	3.43573752	-2.3036717	0.42367165
8.53545758	8.8211058	-2.9534214	8.98064413
7.81094702	7.14376567	-1.0106329	7.76184014
-2.9534214	5.92442469	8.0961337	-0.7767262
5.1738168	3.47165113	-2.9534214	1.35251687
5.07295187	6.59028074	8.20247466	7.89821221
7.58391098	6.59598675	-2.9534214	6.79799772
9.5203335	9.78200794	11.4186108	10.7557255
6.84162215	8.08052422	-1.5168928	6.40011472
4.83225486	5.36474494	-2.9534214	1.35251687
3.33870244	4.11825715	7.88555198	6.09417987
-2.9534214	5.49783731	7.53016619	2.76476989
5.0780392	6.47114844	-2.3036717	2.51996704
5.09319446	5.71265459	-2.9534214	1.88363757
-2.9534214	4.92878064	7.05996591	5.33756575
7.54540266	1.02739222	-2.9534214	5.32671233
5.57021784	6.61538064	8.87029124	7.34759209
7.10911527	7.2952474	-2.9534214	6.08132303
-2.9534214	3.58091222	6.35791613	4.90958885
6.59777924	7.71224183	-2.9534214	5.51057988
-2.9534214	5.23283936	6.93391211	6.72444706
8.09281725	8.77100391	4.54913232	7.99393366
-2.9534214	5.02859763	7.10376414	5.33756575
7.6643309	6.26965207	-2.9534214	6.91221222
5.99686513	7.70735498	-1.8572999	4.7481415
8.92506618	8.4874446	3.08778101	9.41092505
7.1097364	4.50195329	-2.9534214	5.05805857
7.59060114	5.22444384	-0.8115387	5.98968768
4.45990662	4.30899393	8.04047546	6.61607636
7.89298109	7.05185661	3.23008299	6.51691504
7.00411859	7.54238093	-1.0106329	6.25881459
-2.9534214	2.09588014	5.47541277	5.00458266
3.12921138	4.65035121	0.21080887	-2.2872455
7.47193776	7.59298711	3.46890427	6.084548
7.85902664	8.20415524	-2.3036717	8.19231802
-2.9534214	3.89311802	6.18082904	6.25022457
7.27557277	8.11783218	9.78977149	8.76493303
-2.9534214	4.40126766	6.90325739	2.51996704
5.93792507	5.69605204	-2.9534214	3.35131846
7.07071116	6.71237943	1.95412777	4.73998384
7.74130558	8.7267357	-1.8572999	8.21714028
5.28798264	5.83350496	-2.9534214	2.29345235
9.54958456	9.30969264	8.3238402	7.52007222

7.06367571	6.49228248	-2.9534214	5.60568039
6.52234111	6.40024305	-2.3036717	4.63391918
6.03921585	6.51397277	-2.9534214	3.9409004
-2.9534214	4.20188529	7.46988073	-1.2103612
7.72680266	6.34903007	-2.3036717	7.32459186
-0.7256766	6.0365722	7.31812717	6.67823332
4.33372776	-2.9534214	7.29131995	6.30930757
2.23476654	-2.4449602	3.84574144	6.00678743
-2.9534214	0.82014925	4.58489335	3.05514185
-2.9534214	6.1033394	7.31546875	6.66859535
5.16905678	6.82633372	9.10351888	6.7831876
5.26362984	3.45021042	-2.9534214	1.62476777
6.94548021	6.97073243	9.13909695	8.27156488
6.47105412	6.44157419	-2.9534214	4.53837287
6.74177511	4.83538987	-0.8115387	4.08955166
7.22027674	7.82897807	-1.2416692	7.05385932
7.6600966	7.95832932	1.17569549	7.70010464
8.67209565	8.76540847	6.8583032	7.34960402
7.22142668	4.99197078	-2.9534214	5.48151691
6.82128808	5.05013556	-2.3036717	4.76833611
6.59689343	4.15406157	1.35207999	-2.9534214
5.47508172	4.84910728	-1.5168928	1.88363757
7.3965678	7.32106932	-2.9534214	6.93114545
4.93676553	6.48175416	8.86052217	6.71926944
6.40173621	4.90796092	-2.9534214	3.97605485
-2.9534214	5.89298553	7.46088731	4.69427303
7.39248975	8.77657773	-1.0106329	7.83547042
-2.9534214	5.00917909	6.98666555	5.48396131
4.98089049	5.50305272	-2.9534214	1.88363757
2.39009767	-2.9534214	4.86856191	5.82399319
6.20882613	3.69468524	-1.8572999	2.6989601
4.33372776	5.21599919	-2.9534214	0.96158967
6.65166372	4.51578003	-1.2416692	4.00357438
-2.9534214	5.21599919	8.0616493	-1.8332537
7.0279744	6.24080477	-2.9534214	5.57390688
7.14894296	7.08121212	-0.0938779	6.1583308
4.67326705	5.86894822	-2.3036717	1.76000118
0.89001421	5.08996134	7.4323393	5.71999956
5.96838116	3.49975191	-2.9534214	2.87317316
9.64458712	9.02127051	7.62177876	8.17691491
7.2351549	7.07655519	1.616789	5.88999276
-2.9534214	4.70869743	6.63343649	6.28147436
7.47483361	5.97534713	-2.9534214	6.49889919
4.09362543	6.86937456	-1.2416692	1.7919177
5.57562341	5.32013394	-2.9534214	2.68202765
-2.9534214	3.32229932	5.88880732	5.72827068

7.50631005	4.94930418	-2.3036717	6.33390645
6.51579475	6.43976218	-1.8572999	4.80007078
6.73373856	5.58239112	-2.9534214	4.85398021
7.22372381	6.04494006	-2.9534214	5.98451799
-2.9534214	5.48383668	7.89582108	-1.2103612
11.0211985	10.6202636	9.54467136	9.47499462
6.74976714	7.44050235	-2.9534214	5.78090716
6.73212586	7.8543652	7.12820737	0.78254314
-2.9534214	4.92360381	7.63569851	-0.9773123
-2.9534214	4.83263067	6.54769078	6.77622383
7.35001139	5.10832914	-2.9534214	6.0084863
6.58532812	6.06741117	-2.9534214	4.76431974
6.73695855	5.96782241	-2.9534214	4.96663333
6.86535645	7.03934693	-1.0106329	5.76892962
9.06743521	8.41873577	7.30746398	6.99937053
5.52622823	-2.9534214	7.25015136	6.95160067
6.06890539	5.20323879	-1.8572999	3.50363856
6.61887715	5.71864482	-1.2416692	4.60276824
4.8292409	4.96702586	-2.9534214	1.58888154
8.12850115	8.09297164	1.43279367	8.70149584
7.44314801	8.04491024	-2.9534214	7.54017811
-2.9534214	2.31730788	5.67732895	3.26298783
7.34790693	5.80422811	-2.3036717	6.32711539
-2.9534214	2.67859072	5.54514896	5.48396131
7.12702052	7.20691895	-2.9534214	6.42067536
3.36391451	3.80577657	-2.9534214	0.05419288
7.43375309	6.42059735	-2.9534214	6.69309889
6.68538941	2.58987252	-2.9534214	4.32044533
6.68538941	5.50131634	-2.9534214	4.86902002
10.8216401	11.3042537	9.34976987	10.2217142
3.88885195	2.70296801	7.51344585	6.19609178
7.60300703	5.53904199	1.47151796	5.39584256
6.11046375	5.90090978	8.2523221	7.88901924
7.43027632	5.50824933	3.56156037	0.71755754
2.64445705	4.34816098	7.46389135	6.1021576
-1.7015481	5.06666776	7.88330986	1.39469903
4.85020756	6.78981436	0.53716524	2.42138932
7.11159821	6.23663623	-2.9534214	5.89734686
-2.0115616	5.79291687	8.180364	0.64950605
8.30429973	8.52382044	-2.3036717	9.07871201
8.32370423	9.49309448	5.62461287	8.51120079
4.55419169	3.83364304	-2.9534214	1.30906412
5.08816037	3.78879461	-2.9534214	1.85370754
7.56863078	7.44502581	-2.9534214	7.61791157
10.1596155	9.78718325	11.9646584	10.7709438
7.19181309	4.93136211	-0.48063	5.06133591

7.72152945	7.74195573	4.60680206	6.40914607
7.21797411	9.15124531	0.0149924	7.93351531
4.50970305	5.90090978	1.07869645	0.42367165
4.32519541	4.08154148	-2.9534214	1.1208487
4.13326627	5.04060282	-2.9534214	1.01665269
6.6127554	4.51921611	-2.9534214	4.64269729
-2.9534214	4.86539784	8.60125591	-2.9534214
3.65012807	7.45223397	-2.9534214	1.94169689
8.37988438	9.08627399	-2.9534214	9.12349382
5.70621541	4.20615531	-2.3036717	2.68202765
4.98360399	3.68859448	0.29957556	-2.9534214
6.49977167	5.52714504	-0.0938779	4.1023357
-2.9534214	1.96089042	5.08380802	4.99775662
4.30364111	3.95023793	5.79268546	8.22811779
5.65436675	3.65149987	7.08593619	7.69694741
5.85736865	6.43157983	-0.8115387	3.91938907
6.00356493	3.6069818	-0.6366176	1.55207983
-2.9534214	2.94700994	5.69175071	5.20457055
6.39665512	7.04531745	-2.9534214	5.04155929
10.1273886	10.6767888	12.3444353	11.0415411
6.45647809	4.58627311	-2.3036717	4.32044533
7.07517047	5.65290819	-2.9534214	5.67578065
6.22954872	6.07676897	-2.3036717	4.50986995
6.63190837	5.35708493	-1.5168928	4.74406844
-2.9534214	3.56104615	6.07478518	4.95258228
6.80450501	3.82256096	-1.5168928	4.49055026
-2.9534214	3.61342625	7.13952412	-1.8332537
6.79912374	7.44502581	-2.3036717	6.20204286
4.86793961	5.38371888	8.15716702	6.87171813
-2.9534214	4.63459652	6.59558981	4.73588764
3.21484424	5.64349279	8.06758591	6.00508654
4.29055225	5.10146859	-1.2416692	0.84472723
6.8879826	6.23454744	-2.9534214	5.53906892
10.0621361	9.90497876	8.05767795	9.30258504
-2.9534214	4.57641111	6.48164183	5.34565257
6.65762292	7.40750792	-1.8572999	5.94070121
-2.9534214	3.42111796	5.82294929	5.58760987
3.53645535	6.34225624	8.48422627	5.87144181
6.18544646	6.54632982	-2.3036717	4.64269729
1.35986526	-2.9534214	6.05420334	4.33134582
5.14016201	4.9027087	-2.9534214	2.29345235
-2.9534214	4.73848367	7.95381121	-2.2872455
7.2611077	6.05799227	-2.9534214	6.37925682
6.72808622	6.51569398	3.04989883	3.45463059
6.50639098	4.71170388	-2.9534214	4.66009488
-2.9534214	5.41171997	6.8755512	6.23143553

6.68871902	6.69121149	-2.9534214	5.47415876
7.31488909	5.16426612	-2.9534214	6.24734983
-2.9534214	5.2096331	6.92870306	4.81955621
-2.9534214	1.74309531	4.68302791	5.33756575
7.55774647	6.93323076	3.07526381	6.10692315
2.87238163	5.46079184	-2.9534214	0.05419288
8.83177057	8.58494152	6.8222399	7.64710573
5.10321023	6.77408117	8.10693538	7.82875508
6.29873075	6.77408117	-2.9534214	4.86527474
6.27788016	3.80577657	1.12801103	-1.8332537
6.16048307	6.05681058	-2.9534214	4.46107681
6.74657564	6.29091522	-2.3036717	5.47415876
5.08816037	6.88277409	-2.9534214	3.40389911
6.41083732	6.2757593	-2.9534214	4.8727556
-2.9534214	3.76583651	5.56542902	6.61607636
4.63235338	5.8982732	-1.0106329	1.88363757
-2.9534214	4.14519343	6.32260733	4.62508733
7.73407234	6.19319955	-2.9534214	7.81765442
7.11035727	6.53957785	9.1916868	7.90415638
6.5427241	6.67281701	-2.9534214	5.21637896
7.01211443	6.49315638	2.13387043	4.88760193
6.29217885	5.60520443	3.44963694	-2.9534214
-2.9534214	5.09457526	6.87645331	3.79103405
6.37201363	7.91072156	-0.8115387	5.98624328
8.20257572	7.60839388	9.16258393	9.53191659
7.98217976	8.96930431	-1.8572999	8.9092343
7.04755728	6.95622885	-2.9534214	6.37531216
7.07644203	5.67464139	-2.9534214	5.93535263
7.19239963	7.33624611	-0.48063	7.02141361
6.50827664	5.14437774	-2.9534214	4.8464011
7.16457119	6.50446919	-2.9534214	6.44722403
5.82596128	7.45088518	-2.9534214	4.69004478
-2.9534214	6.04851148	7.06075979	6.46836363
5.54104097	7.37468992	9.4781837	6.69942325
5.14259214	7.30523368	-2.9534214	3.70144947
8.49133195	7.8348499	8.10693538	2.76476989
6.36162081	4.87883419	-2.3036717	4.49055026
5.3958115	4.03429258	-2.9534214	2.55758639
6.61450713	5.51170338	-2.9534214	5.08084509
7.89694487	8.25192256	3.0240799	8.26944103
-2.9534214	3.19917447	6.40619009	-0.3022547
-2.9534214	6.21666951	6.74630409	7.68637315
12.7879634	12.9449243	11.1855913	12.399258
7.23002212	7.10884234	0.0149924	6.87358364
-2.9534214	5.64663809	7.00575505	5.53435965
6.60748743	6.69273383	-2.9534214	5.45435155

-2.9534214	2.40906789	5.08068028	5.25696345
4.08859285	-2.9534214	7.23334925	5.78289379
-2.9534214	3.56104615	7.30143112	-2.2872455
6.77504813	4.9619847	0.3831957	4.24751088
7.15978051	5.79433563	-2.9534214	6.22706462
7.31273273	7.37895144	2.6380896	6.50613255
-2.9534214	5.12646603	7.42125465	-0.7767262
8.17137901	6.69273383	2.79898818	7.71214372
7.0160958	4.82709638	-2.9534214	5.6736408
6.971001	5.74676361	-2.9534214	5.75280329
6.23069129	8.08950873	1.12801103	5.86957353
6.07527958	8.14422009	2.31513045	5.44434502
8.34628136	7.89361444	9.98632039	9.12290578
-0.3527908	-2.0697503	3.13679123	3.3405684
6.40982892	5.89298553	-2.9534214	4.91322098
5.84548442	5.30627827	-2.9534214	3.75909775
6.99338805	8.03384214	1.74876101	6.99766088
8.53799808	8.40440662	8.01571458	5.45684237
6.36266347	5.80422811	-1.8572999	4.84259657
4.63580752	4.25230982	-0.3398746	0.50294321
-2.9534214	5.6450663	6.99333406	5.39843638
6.36786548	4.93651123	-2.9534214	4.66009488
6.41285202	2.5767411	-2.9534214	4.2417448
7.06623803	7.8024313	1.86966605	6.86235423
6.52513762	4.21889004	7.24457237	8.00971168
6.49312186	4.03429258	-1.0106329	3.92659521
5.76259853	6.35673285	-2.9534214	4.21844721
4.74218749	6.71911797	0.53716524	2.61223772
5.92245248	5.21811499	-2.9534214	3.89019908
5.97521424	-2.9534214	10.7353837	5.50095713
6.43383819	6.40024305	-2.9534214	5.12223114
5.68803004	5.45003031	-2.9534214	3.58786429
7.08025001	6.17152675	-2.3036717	6.37925682
2.17903669	6.33837115	7.34248806	7.11664045
6.9551899	4.67212268	-1.2416692	5.2711856
7.71664471	7.25614162	-1.8572999	8.30443665
6.60308271	8.21187324	-2.9534214	6.70047462
6.77739567	6.94924047	-2.3036717	6.13051758
8.66069618	7.58972251	6.57573222	6.21239855
7.4549285	8.200682	3.01099506	7.57441625
4.92552015	5.70513165	7.46868484	7.71942298
7.1906393	7.3584763	-2.9534214	7.17453496
6.06890539	5.72461028	-0.3398746	4.02387492
6.91666291	6.44609429	1.02763635	5.43426861
-2.0115616	3.96543144	5.82668801	5.92998415
7.04040772	7.14153789	-2.9534214	6.70676681

3.94004262	7.34062253	9.20071862	5.48396131
6.1117046	4.84363596	-2.9534214	4.16460995
6.21230063	7.72047434	-2.3036717	5.7588718
7.87409439	7.06602182	5.2901515	5.15020303
-2.9534214	6.42518355	7.22487424	5.9083082
5.60235159	7.35415378	8.77723856	7.53370387
5.70127845	7.32352802	-1.2416692	4.62508733
-2.9534214	5.56919112	6.27140232	7.67785758
8.63934522	9.53528166	6.45169087	8.69623504
6.24546299	6.28688918	-2.9534214	4.90594756
4.82319403	3.90367309	8.00544261	6.30930757
2.77549695	2.50922903	7.23897172	5.21931602
3.7992784	9.47030917	8.92210547	8.4023417
-2.9534214	3.92972776	6.16614885	3.9829841
6.74417737	6.67281701	-2.9534214	5.85453952
6.60131705	5.9114082	-0.48063	5.03490637
7.21624473	6.64870499	-2.9534214	6.84629477
-2.9534214	6.34999517	7.04158491	6.72651292
4.35484045	7.22228458	-0.8115387	2.97399725
2.58851621	6.70786951	7.97502297	6.2035268
6.6820521	6.01482929	-1.8572999	5.48883773
5.58100881	4.27687621	-1.8572999	2.88801522
5.94211623	5.82658798	2.91591949	1.26426187
-1.7015481	6.10905139	6.86012852	6.78417968
9.22537051	7.92439228	9.72513915	10.0984176
-2.9534214	5.56919112	6.53060052	7.05385932
-2.9534214	5.7260978	7.53131221	-0.4437536
-2.9534214	5.25567824	6.72342355	4.92045792
7.13984906	8.1141445	8.8116028	9.31465108
6.51110052	5.32995001	-2.9534214	5.12849415
-2.9534214	5.55419531	6.75024654	6.13984836
6.69618266	5.42643459	-2.9534214	5.43173847
6.81596916	6.23767949	-1.8572999	5.88260098
0.39849386	4.87883419	6.62379891	6.26593408
-2.9534214	7.02067971	8.0584731	-0.6006549
-2.4069576	5.07135666	6.50982133	5.3402664
-2.9534214	4.20188529	6.30930754	3.40389911
6.03660504	6.76831721	-2.3036717	4.90229705
6.71589897	6.38901846	-2.9534214	5.73854334
5.92245248	6.89009113	2.90181097	3.38309624
8.9017639	9.15690897	10.8459004	9.77735426
-2.9534214	6.08953764	7.02946989	5.93535263
5.75943532	3.34571176	-1.5168928	2.59424966
5.36700485	4.66593571	-2.9534214	3.02859755
6.25335477	3.08324495	-1.8572999	3.56033181
6.24997786	7.53730603	5.8994972	2.12826873

6.20650514	5.11288479	-2.9534214	4.58466098
4.33797509	6.65808654	7.6916106	7.65417723
6.05478225	4.67212268	-2.9534214	4.1585021
-2.9534214	3.33794975	6.72642863	-1.8332537
-2.9534214	2.84116722	5.13295112	5.34565257
-2.9534214	6.62975686	7.47584535	2.91724899
6.94617593	6.92029383	8.92407003	8.06708279
7.88066011	7.57370092	9.65721251	8.65398622
-2.9534214	4.63142484	6.07478518	6.02704312
7.84529249	8.08835258	2.98446389	8.29576701
7.03779907	7.31811335	1.71688244	6.48919795
8.13461794	7.30074841	4.44595762	7.2468433
7.86529335	8.72543478	6.48282703	7.00788854
6.70113705	4.29302445	-2.9534214	5.38018091
7.1097364	5.84038894	-2.3036717	6.53824123
7.55729117	7.04769876	-2.9534214	8.08731896
-2.9534214	5.64034062	6.87103221	5.23391229
6.70113705	7.33039015	9.30172104	7.74308518
-2.9534214	5.80844707	6.95028444	5.31577664
5.94768557	7.72866015	2.85863818	4.71523038
6.13629998	6.52170212	9.54637286	6.56504048
6.59156711	7.66881333	8.99742961	8.19418524
7.21797411	5.63401554	-1.8572999	6.64148038
6.55463461	7.4921222	-1.5168928	6.30516666
-2.9534214	3.56769861	7.95765241	-2.9534214
-2.9534214	5.92830685	6.78332934	6.57653883
-2.9534214	6.42426747	7.05758163	6.46217819
3.48376111	5.48910286	-2.9534214	1.1208487
5.50182837	6.18673183	-0.6366176	3.73467286
10.7166855	9.95697597	11.9334925	11.2000546
-2.9534214	6.74649488	8.18255391	-1.4884482
6.89087642	4.36353484	-1.8572999	5.51775506
4.71631881	3.64522363	-2.9534214	2.22496144
5.94211623	5.51686898	0.0149924	3.75100198
6.66186447	6.42426747	-2.9534214	5.76088898
-2.9534214	5.34745237	6.86649901	2.0512437
7.25830926	7.4921222	9.74256058	7.92724213
4.72931113	2.36391729	7.13198949	6.53352924
4.33372776	5.00428334	6.8899176	7.58811443
6.02742983	7.4445741	6.84822244	-2.9534214
7.01874396	4.22731785	-1.2416692	5.48640158
-2.9534214	6.09300048	7.12820737	4.71938556
6.62410377	5.80844707	-2.9534214	5.52013887
-2.9534214	4.49847579	8.28530132	-2.9534214
7.01078487	7.91170235	-2.9534214	7.38469326
3.85960771	6.48087332	-1.0106329	1.94169689

5.7876576	7.26844453	-2.9534214	4.85019564
4.63235338	2.91606439	7.04880539	6.68143172
-2.9534214	7.38931578	7.50996214	5.56700626
7.10288915	6.24080477	-2.9534214	6.68036637
6.25447865	7.77287024	-1.0106329	6.06182035
7.20408063	6.66972834	-0.0938779	6.885651
-2.9534214	5.60843423	6.75712	5.63896438
6.3771821	5.20750481	-2.9534214	5.11908941
6.61100155	5.29228822	-2.9534214	5.48151691
7.46612858	7.91854926	8.89543001	9.20354018
3.68388926	4.70266566	-2.9534214	1.35251687
7.12394922	6.06858422	-2.3036717	6.75715096
-2.4069576	5.22654731	6.37205707	6.03706531
5.74990385	4.89214656	-2.9534214	3.84527593
6.10797886	4.90007541	-1.0106329	4.08955166
6.37924431	7.4195098	-1.5168928	6.03039159
-2.0115616	6.06741117	6.62272408	7.02477516
7.28164921	7.04591315	9.10178424	8.19791246
4.1185283	3.47872781	-2.9534214	1.69396844
5.91962137	5.92830685	-2.9534214	4.62065105
7.34104617	8.44087229	10.211415	8.21787472
-2.9534214	6.69653266	7.22345689	5.76692366
-2.9534214	5.32799214	6.60867796	4.85398021
6.72889505	0.98825706	-2.3036717	4.09595784
-0.8749787	6.00505959	7.51865569	3.66744291
6.39053409	7.58808743	1.47151796	5.76892962
-2.9534214	3.02642617	5.03616461	5.79672409
3.33019955	5.36283374	-1.2416692	0.84472723
6.2111434	6.14841438	-2.3036717	5.16551178
6.93640519	6.26351888	-2.9534214	6.41043166
3.23320271	7.56541518	7.90070668	7.21556143
6.83937691	4.77630857	-2.9534214	5.80652233
-2.4069576	4.74436782	6.28369509	4.86527474
5.13284699	5.01649171	-1.2416692	2.71569611
7.73366943	8.33542607	5.80410892	6.92216061
6.79294904	6.53279414	-2.9534214	6.22998003
5.10570335	4.49847579	-2.9534214	2.85817682
6.12282444	6.02453328	-2.9534214	4.98400687
6.29655009	4.57310874	-2.9534214	4.8464011
-2.9534214	5.00917909	6.18956599	6.2616666
6.71426619	3.9089218	-2.9534214	5.56007248
6.68788733	7.44412225	-2.3036717	6.68249627
7.34685355	6.93323076	0.21080887	7.53252361
5.90824103	6.15615971	0.91975246	4.06363863
6.59512017	7.61362707	-2.9534214	6.54995429
6.06250292	6.75526346	7.64048576	8.60289203

6.84908115	3.25794247	-2.9534214	5.71168074
7.1579799	6.48702792	-2.9534214	7.00873759
4.39208173	4.11825715	-2.9534214	2.1030479
11.3838954	11.1269904	10.3923525	9.95711734
9.05458857	8.60529198	8.60968915	5.97238283
6.01023376	6.25117372	-2.9534214	4.91322098
8.5107444	8.60771486	10.3122801	9.3420259
-0.3527908	4.94675465	6.90237189	4.45111698
7.27833796	7.61563479	9.11483907	8.54554028
5.10570335	5.90748024	-2.3036717	3.43455156
10.5779081	9.74644295	12.2753247	10.6509227
4.68331671	3.63258865	-2.9534214	2.37999157
6.13751881	4.9619847	-2.3036717	4.69427303
6.64995657	6.94413666	-2.9534214	6.23143553
10.4696894	12.0449754	7.94351771	11.4329507
7.36413674	6.63768218	-2.9534214	7.66982833
-2.9534214	3.3611116	5.29285694	5.4215733
5.80770238	4.77917751	2.05956205	0.33978981
8.84133895	8.92076482	6.33578564	8.73266404
6.08035873	4.21465761	-2.9534214	4.42591237
6.49026253	7.36708268	-2.9534214	6.2079695
5.72741579	6.96884891	8.2971399	7.78680345
4.25055772	3.96543144	-1.5168928	1.51431472
6.68955022	5.19036453	-2.9534214	5.71376495
6.42088279	5.62925347	-1.8572999	5.35101871
7.13130939	5.90090978	4.77877537	-2.9534214
-1.7015481	4.38252461	6.84269401	2.02462691
6.44569463	5.18604745	0.0149924	4.70688394
7.11035727	7.2058532	-1.2416692	7.43735446
6.13751881	5.01162075	-1.5168928	4.55241591
5.25915756	6.20391507	8.12716259	7.1768031
3.08945741	5.90616854	-2.9534214	1.06969116
6.43482995	4.33652211	-1.5168928	4.86151971
8.69462788	8.46044183	6.52945394	7.96316498
-2.9534214	5.65134321	6.62164845	5.94247967
5.35448086	5.53904199	7.22911797	7.86014299
5.46928521	5.34938403	7.93920699	7.08156194
-0.0567579	5.61165681	6.58127569	6.70676681
7.67108002	7.64777655	9.48588855	8.52372865
-2.9534214	5.55251944	6.99333406	0.78254314
6.99001832	6.1878118	-2.9534214	6.64476122
-2.9534214	5.33580776	6.46254498	5.22517226
-2.9534214	2.45284824	4.69534966	3.93376554
7.51711972	6.34225624	6.21117963	10.5796449
7.87116667	8.51867971	5.80600407	7.46421986
-2.9534214	6.08026256	6.90414234	5.40102555

2.87238163	-1.7722575	7.58362093	3.95506514
6.89952323	-2.9534214	1.95412777	7.6762553
6.99406105	7.36374185	0.86262618	7.038975
6.96002034	4.53627482	-1.2416692	5.79081305
8.95700068	7.83347044	9.85036998	9.51184415
-2.9534214	4.36353484	6.00059907	4.68155092
6.12159312	6.05444427	-1.2416692	5.05148149
6.2789851	4.49149556	-1.8572999	4.79220231
5.51126183	4.2356967	7.19191752	7.35495551
-2.9534214	6.11701038	6.75712	6.33661395
6.72646719	5.01649171	-1.8572999	5.74467202
6.76167278	7.09048111	-1.0106329	6.66644481
5.97248489	6.61858776	8.29815013	7.66337259
7.33574637	7.92795141	7.0229667	4.50506423
-2.9534214	3.62622942	6.29992419	-0.9773123
6.9825771	7.87797573	-2.9534214	7.60730021
-2.9534214	4.58954551	6.23528993	3.3405684
9.8855182	11.418317	8.20211491	10.4364139
5.77361547	4.50888322	-2.9534214	4.07016065
7.07071116	6.45329713	-1.5168928	7.02141361
5.83800676	5.41171997	-2.3036717	4.54306909
6.42787323	4.93136211	-1.0106329	5.10645361
9.66575562	10.1612888	9.23101064	8.38870892
6.76088212	5.85541905	4.55815602	-0.1734029
-2.4069576	5.79007514	9.03790597	-2.2872455
7.03845167	4.92878064	3.23008299	1.7919177
7.54494345	8.04103112	-1.5168928	8.72338408
5.19035429	5.48207701	-2.9534214	3.49396925
6.03529787	5.01405828	-0.48063	4.13380881
6.67031034	3.89840521	-2.9534214	5.70121431
1.76508181	6.78268417	8.06481853	4.91322098
6.50921855	4.94420061	-2.9534214	5.57619977
-2.9534214	6.34128595	7.01397669	5.2134359
0.58157832	0.15185078	3.20731949	5.40876529
8.08430979	6.66972834	9.65261218	8.16019974
6.41083732	6.46937322	-1.2416692	5.69911188
6.50639098	6.12379759	7.86342749	8.13864189
8.28847627	7.57535237	9.35252798	9.09479415
6.11542074	5.29027853	-2.9534214	5.03823667
5.78143352	5.06666776	-2.9534214	4.33134582
8.01205744	7.12584632	4.73169581	6.89026555
1.71370892	7.46431666	7.42249049	7.16314043
5.75785111	6.77838909	5.21226299	0.42367165
6.42687666	5.12421135	0.53716524	4.56632356
9.27064943	10.6265496	8.83684927	8.85573673
6.67283449	8.49138617	-2.3036717	7.57269479

-2.9534214	7.2095799	7.05678599	7.06451441
-2.9534214	5.5575412	6.54428889	4.92765874
5.27917456	4.45248865	-2.9534214	3.46456627
6.34377937	6.68587062	-2.9534214	5.73238853
6.54731671	-2.4449602	7.83957133	6.56157303
8.68757228	7.87864467	11.2962425	8.35405251
4.46768038	6.6265745	2.55036907	2.02462691
8.01670148	6.56390483	3.54349911	7.27746125
7.2402695	7.28569598	8.8061687	8.44624915
6.99271473	6.50446919	-2.9534214	6.95336576
5.71603893	5.8252006	-2.9534214	4.54775007
6.01023376	4.48448139	-1.2416692	4.17674861
6.68871902	4.94164203	-1.5168928	5.69489781
7.00345026	8.45842966	6.92260196	5.30752013
-2.9534214	5.06431759	6.92434777	-0.9773123
4.87672456	6.00628442	8.17670675	6.5992642
6.70278474	7.2972502	-2.9534214	6.78814122
9.66036006	8.40533538	10.5371496	10.0078962
6.75771514	6.14174226	-1.0106329	6.23579323
2.12106726	5.61487221	3.25249288	-2.9534214
7.31542767	7.41813005	-2.3036717	8.23466442
6.03267995	6.76325478	9.04374012	6.88194871
5.2479158	4.88417389	-2.9534214	3.56033181
10.6218971	10.6819238	9.45723297	9.78133456
6.16885228	4.54304223	-2.9534214	4.98400687
6.55827969	7.5889052	-2.9534214	6.76320126
3.54382842	7.21594623	7.67873969	7.2900876
4.81407594	4.35201983	6.00885006	7.94065141
5.87355125	6.4949026	-2.9534214	5.07112348
6.07018249	3.94513766	-1.8572999	4.33676534
-2.4069576	7.6591122	4.63266077	-2.9534214
6.14602191	6.1495234	2.13387043	4.10868549
5.5243659	4.45607864	-2.9534214	3.9048679
6.979182	2.85210783	-1.2416692	5.24836237
6.80680514	3.95023793	-1.5168928	5.63676918
6.45354513	3.70679015	-2.9534214	5.48151691
3.04857689	5.35131312	-2.9534214	1.21802358
10.6438812	10.9041837	8.25683241	10.970705
6.64910223	7.09739399	-2.9534214	6.62274674
8.20170201	8.75178067	9.59033424	9.70612209
6.21807294	5.77721766	-2.9534214	5.41902078
6.59512017	6.47380719	1.12801103	5.51297557
8.51403277	7.86250384	5.47779245	8.13708965
6.11789286	4.34429178	-0.6366176	4.08955166
6.64482294	6.2573595	-2.9534214	6.24446935
5.96701066	5.73646771	-2.9534214	5.07112348

6.82128808	4.54977803	-2.3036717	6.1021576
5.55205156	-1.1316325	7.07261595	6.29545808
6.13751881	6.43976218	-2.9534214	5.46428915
4.79874987	6.1878118	-1.5168928	3.45463059
7.14712876	7.50431026	-1.2416692	8.069933
6.75692231	5.15546067	-2.9534214	6.14449121
3.01713789	5.51170338	-1.8572999	1.17025416
6.68288715	5.53225575	-2.9534214	6.09417987
6.23979945	4.67212268	-2.9534214	5.26266914
4.40832977	3.98544371	-1.8572999	2.22496144
8.54421494	9.57296748	7.60337097	8.17010028
6.46135315	4.78489834	-0.48063	5.06133591
6.66271128	3.74837496	-2.9534214	5.84125502
10.5676025	11.0115788	9.45088797	10.0906196
-2.9534214	4.76477541	5.8690011	5.71792434
-2.9534214	6.0365722	7.20633912	0.15580464
6.46524137	6.530242	5.68969926	-2.9534214
7.37451151	8.93530601	7.84833381	5.8393472
6.1326373	4.68441757	-2.9534214	5.04155929
6.89664672	6.64084016	-2.9534214	6.93561698
-2.9534214	3.82256096	5.56542902	4.03725163
5.36075645	4.94675465	-2.9534214	3.85286094
4.05286452	5.06666776	2.41784535	-1.2103612
9.57915164	8.97525851	10.7438119	10.1381461
7.0279744	5.74676361	-2.9534214	6.95600934
5.71603893	6.43431244	-1.5168928	4.94195344
6.22267421	5.41541272	-2.9534214	5.41902078
5.30109473	5.29830058	8.82778337	5.7568518
-2.9534214	4.26873381	5.83969778	4.18277985
6.54180382	5.33190524	9.15479734	6.53941682
4.83526253	5.75989414	4.01258603	-2.9534214
-2.9534214	3.83364304	5.54514896	4.00357438
10.8145138	10.1315005	10.9704726	12.0453811
6.37408324	7.023102	-1.5168928	6.22852306
7.19239963	8.07586507	9.9541025	7.84215466
8.57467871	9.13187693	6.98916985	8.30409087
6.53349479	2.71500391	-2.9534214	5.58533506
1.83886594	-0.078038	5.41460832	4.44108792
6.78985175	7.15595738	0.53716524	6.76017928
6.88943024	5.83350496	9.98673818	6.42704092
9.47874406	8.52253698	7.96488024	7.67518613
3.33019955	3.94001929	-1.2416692	0.9043414
5.55387852	6.07443521	9.3444008	5.74263202
2.67163453	8.48129178	8.03280852	7.26187744
6.59067747	6.63213903	-0.8115387	6.28988085
7.70230235	7.72680382	-2.9534214	8.98862771

-2.9534214	3.82811264	5.30898388	5.52489472
6.78363715	5.93088917	-2.9534214	6.51213277
2.32311713	4.81036498	7.10530403	5.57390688
6.65251655	7.35271006	-2.9534214	6.94717849
7.00878821	9.04842724	-2.9534214	8.2852942
6.33954912	5.91271515	-2.9534214	5.73238853
8.19936952	9.84334752	6.80149162	8.5822219
6.47588025	6.84425248	-2.9534214	6.30654829
5.01577414	5.87432456	-2.9534214	3.70982732
6.19601422	3.57432054	-2.9534214	5.05477378
-1.0415346	3.69468524	4.4937717	7.14471989
7.42379715	6.24184502	-2.9534214	8.27439182
5.23430904	4.37495867	-2.9534214	3.61488117
2.51539463	4.86269549	6.81095967	6.20649012
6.45354513	5.1421509	1.22185416	4.44108792
7.19532876	5.64349279	9.04734949	7.23673278
6.45647809	5.71714959	-2.9534214	5.86957353
7.40570147	2.50922903	-0.48063	5.28246323
6.46037946	7.11510043	2.15781303	5.65640678
5.94768557	7.3176201	-2.9534214	5.72620734
4.7064972	5.01649171	8.02510063	6.1021576
5.94768557	4.56315603	-2.9534214	4.77633546
6.35115258	6.99005286	8.7484557	7.51709169
5.74990385	7.3522285	7.43601533	-2.2872455
6.4787682	8.05026418	3.21874614	6.43337853
6.18897765	5.93989104	-1.0106329	5.38018091
9.09310444	10.2211268	8.83870192	8.38380869
-2.9534214	4.4949899	5.48963229	6.18711887
7.80290367	8.27013649	8.90629197	9.56404042
6.55736929	6.96318354	-2.3036717	6.64148038
4.06828513	6.0976047	-2.9534214	2.6989601
6.23979945	7.11680248	-2.9534214	6.08937197
-2.9534214	3.78308925	5.74803792	0.84472723
6.80680514	4.69357047	-0.2116394	5.52963495
4.54319748	5.20110105	8.10578192	5.95838793
5.54288191	3.39142674	-2.9534214	3.96209569
7.1300853	5.4355556	4.65386118	-0.9773123
6.60308271	5.85541905	-2.9534214	6.20055739
5.9628913	2.84116722	-2.9534214	4.51465971
5.07295187	3.5066924	7.44941456	6.40657145
10.8274103	11.7117844	9.01310057	11.3071168
4.9727192	4.74142875	2.78368219	-2.9534214
6.97031715	3.91415149	-2.9534214	6.53470868
7.09538218	4.34816098	9.42999502	6.47329296
2.39009767	3.08324495	5.99396413	5.69278614
6.61625673	4.54304223	-1.2416692	5.63016346

6.82583159	6.57550367	-2.9534214	6.92216061
5.81228882	5.88101693	-2.9534214	5.07112348
6.51485713	7.14153789	8.7885449	7.67250971
6.09172186	8.24882131	-2.9534214	6.72237824
6.55554675	5.13992062	-2.9534214	6.00338365
3.26030968	-2.9534214	-2.9534214	7.73130259
6.5371936	5.52372781	-2.9534214	6.03039159
8.71456949	8.57424473	10.549787	9.10634262
6.94478416	7.21541677	2.15781303	6.76822386
6.7906267	6.59923723	2.41784535	5.64334479
6.30850315	4.56315603	-2.9534214	5.59441287
6.27456023	5.46792185	-2.9534214	5.66504952
6.90382728	6.05562791	-1.5168928	6.90857755
4.02679147	3.01673495	-1.2416692	1.30906412
5.75467746	5.08996134	-2.9534214	4.69004478
6.0339895	-1.1316325	-2.9534214	3.72643846
6.7003125	7.37136669	-2.9534214	7.1768031
6.6845558	5.24741496	-2.3036717	6.25881459
6.19367251	5.0968767	7.60118982	7.48267122
0.89001421	6.12153873	7.25432149	5.29086398
3.7992784	4.2356967	7.60609277	5.5646987
6.83262015	4.89479435	-2.3036717	6.44597082
5.77047634	5.38748391	8.2397579	6.72134272
6.28669611	4.27687621	-2.9534214	5.53671621
5.87501343	4.31295883	-1.8572999	4.46603105
5.70457163	1.17410899	-2.3036717	3.10680874
3.7992784	3.51359967	1.50922995	-2.9534214
-2.9534214	3.69468524	7.71499717	-2.9534214
6.14238386	6.12605292	-2.9534214	5.61463147
6.51391889	7.13707197	-2.9534214	6.68143172
5.22516605	3.6069818	-2.9534214	3.60593164
5.97929861	6.70711649	2.39787909	4.52893433
5.20205179	4.80192595	-2.3036717	3.79103405
6.20650514	5.52030247	8.10347222	7.20670918
-2.9534214	5.77146624	6.43341722	5.14093919
5.58994004	6.10448361	-0.48063	4.62508733
7.36621767	4.28093025	7.63303204	7.77985715
2.90709921	2.84116722	6.76688277	4.99775662
6.63017766	7.39447017	-2.9534214	7.13852707
7.95754795	6.6980494	4.44108789	6.78417968
6.43185261	7.28670438	-1.2416692	6.66429106
6.98122002	7.84172748	-2.3036717	8.11004717
6.37614988	3.18193433	-2.9534214	5.57848902
5.04464625	5.11969139	-2.3036717	3.7181568
7.58256922	8.66912913	5.87081294	7.48450345
6.25110438	5.41725555	-2.9534214	5.70331368

8.01006255	9.56706576	0.3831957	9.79739252
6.68705517	5.71864482	-2.9534214	6.43337853
7.00945407	7.21435728	7.81295997	-1.8332537
10.7473456	10.6125434	10.7824653	8.29889409
8.75662198	9.31538602	7.92576128	7.99221755
8.94486864	9.18611497	10.4932763	9.80352138
-2.9534214	4.78489834	6.64301011	-1.2103612
5.64067838	5.63084257	-2.9534214	4.79614191
-2.9534214	6.37011454	6.59229913	6.33931637
4.77701612	4.1227816	-2.3036717	3.11944144
8.04782582	8.15226457	7.03028073	6.44220466
7.51477667	7.48993488	3.43010881	7.73848605
6.88435714	6.21349146	-2.9534214	7.07021933
7.0067888	6.26761057	-2.9534214	7.39252088
7.25830926	6.72508148	3.49733123	6.09257902
5.94490359	-2.9534214	2.73676511	6.91764712
9.12847486	9.2394296	9.80453947	5.67578065
3.11937512	6.06623717	7.60064402	6.07647202
-2.9534214	3.25794247	4.85763946	4.48567964
5.86327426	5.59221246	-2.3036717	5.20753173
6.03136921	7.00605994	6.86740679	-2.2872455
6.75454119	6.44066847	3.33880563	4.80398897
4.06316322	-0.078038	7.66731751	4.65576511
6.80986629	7.28922229	0.0149924	7.33410699
-2.0115616	4.91580357	5.87442982	5.30475742
4.44028697	4.14073883	7.04158491	6.53470868
4.08859285	6.71237943	-2.3036717	3.36198902
5.44389433	7.44818381	8.08992481	7.87883886
6.64653618	4.92360381	-1.2416692	5.97412269
4.28616277	5.76134574	-2.9534214	3.02859755
5.61640669	4.13178815	-2.9534214	4.38986938
-2.9534214	7.44186088	6.95712285	6.90402129
5.27917456	6.08142521	-2.9534214	4.48079253
5.20902504	6.48439345	8.18328314	6.89118669
5.60939626	6.25530052	-1.8572999	5.0743713
6.40072142	7.46208675	-2.9534214	6.76420718
5.77047634	4.39753848	-2.9534214	4.66009488
6.31928429	7.92827453	-2.9534214	7.00788854
-2.9534214	4.82709638	8.11192308	-2.9534214
6.38438698	5.63242993	7.84603305	7.56809418
5.94768557	7.82134358	8.77167321	-2.9534214
6.80373749	7.49909941	-2.9534214	7.64218969
5.20205179	6.70636307	8.12260762	7.07913888
7.10039117	6.61136169	1.98121746	6.67502781
5.09821103	5.2411863	6.95370769	7.52897702
4.04249178	4.65660523	7.96784591	5.39324406

5.25242302	5.39123914	-2.9534214	4.12130184
4.2595417	7.34595353	8.03280852	6.96128202
6.67535423	6.56224021	-2.9534214	6.8453445
6.65507199	6.50446919	-2.3036717	6.83101492
7.10413653	6.75161636	-2.9534214	7.97407403
5.82293414	3.42111796	-2.9534214	4.61173735
6.36994105	5.07603037	-1.2416692	5.65857232
6.00890246	6.29493005	8.62801701	6.88380104
5.92950606	8.15004988	8.62855256	8.07683158
6.8006633	5.19466873	-2.9534214	6.69521009
5.93091264	6.86059765	-2.9534214	5.69067139
5.82898209	4.68135366	-2.9534214	4.88760193
6.24206753	7.99642565	-2.9534214	6.9595266
6.36682658	7.96054259	9.10928601	7.69166997
5.96701066	5.20750481	-2.9534214	5.32125485
6.24885047	5.5339553	4.75542762	-2.2872455
5.75626515	7.63080285	-2.9534214	5.91918688
5.49613854	5.74382942	-2.9534214	4.71938556
8.08019564	8.47665988	6.10126431	7.8871736
6.7656196	6.11246776	-2.9534214	6.91221222
3.96771613	5.92959859	7.68287088	6.38056931
-2.9534214	4.17599634	5.55419753	3.65013424
5.06272305	6.08606647	-2.9534214	4.22430697
3.02769386	3.88248517	-2.9534214	1.51431472
7.67947227	5.98531943	8.28190095	8.22446789
9.86441814	8.59209688	10.9399459	9.88103001
6.09674355	5.24741496	0.29957556	4.74406844
2.77549695	7.23540072	7.92706797	5.95133924
7.25494394	6.9832268	-2.9534214	8.40782331
6.36682658	5.58074771	-0.6366176	5.71168074
7.78978526	9.29435777	-2.9534214	9.44692213
6.6127554	5.38371888	0.0149924	5.76088898
6.15447519	7.0005391	-2.3036717	6.23434213
4.24604466	6.05799227	7.86615491	6.34605037
6.11912734	7.57452688	0.67627567	6.2630905
6.48930816	6.63055136	-2.9534214	6.56388559
8.74866794	9.57152017	10.5683286	9.82934249
6.98731686	6.15063158	-2.9534214	7.46731152
6.25110438	5.05250893	-2.9534214	5.79672409
6.07018249	1.13880476	-1.8572999	3.39353517
5.37942105	5.51514917	-1.8572999	4.38986938
6.49787485	5.65759294	-2.9534214	6.23579323
5.64582675	6.8435674	-2.9534214	5.33486004
5.25915756	2.43840191	-2.9534214	3.75100198
6.12159312	5.84038894	-2.9534214	5.71999956
5.60235159	5.29429513	-2.9534214	4.7481415

5.63378518	4.76477541	-2.9534214	4.61173735
-2.4069576	5.70965012	6.73142323	2.02462691
5.1738168	6.06623717	2.27190545	2.97399725
2.91848853	-2.9534214	-1.2416692	5.49612158
5.39784727	4.08154148	-2.9534214	4.16460995
-0.3527908	1.74309531	4.12008362	4.3741424
6.83262015	7.71299218	8.85824078	8.14367518
6.49026253	5.34745237	-2.9534214	6.18561795
8.14465428	8.71872574	7.06155324	7.46421986
7.3073277	4.21465761	-0.3398746	6.74191327
3.32164624	4.71769805	2.94372925	-2.9534214
6.54823348	5.94756247	-2.9534214	6.39362896
5.85292353	8.24025832	-2.3036717	6.72237824
7.49636911	6.73102044	-2.9534214	8.82810492
5.49613854	4.97955223	-2.3036717	4.46603105
8.32129289	6.15505578	4.62409259	6.73166466
6.21345694	4.14073883	-2.9534214	5.64553002
4.78947547	4.42344256	-2.9534214	3.444626
6.7003125	5.71265459	-2.3036717	6.75512854
4.28616277	-2.9534214	7.27019624	4.94904802
6.44372531	6.82077517	-2.9534214	6.66644481
6.01819589	6.3528866	7.83540203	7.66337259
7.38327179	4.69357047	9.96802859	6.40399222
6.82885267	6.56307276	8.06719089	8.14483422
7.87263127	8.33566969	9.24242685	9.17356765
4.09362543	-2.9534214	5.61815551	5.38280302
5.6475388	4.30899393	1.43279367	2.177425
5.76733038	5.34745237	8.31920317	6.48676241
6.30850315	5.89166058	-2.9534214	6.06182035
6.31282528	5.07369541	7.9387752	7.1134865
3.30438598	6.21666951	-2.9534214	2.29345235
7.15617703	2.36391729	-0.0938779	4.67728517
3.14868499	6.76976036	-1.5168928	2.44165109
5.54288191	6.51052416	-1.2416692	5.15634603
6.19835214	5.6761813	-2.9534214	5.91012702
6.24997786	7.16749959	-1.2416692	6.54645035
6.68871902	7.43186851	-2.3036717	7.64874069
5.28578565	2.70296801	-2.9534214	3.91938907
4.76760048	5.39498462	-2.9534214	3.72643846
9.24486924	8.31555483	10.1592744	9.60110516
7.98928553	7.60879711	8.13208098	3.5510363
6.38438698	5.63876195	0.6083965	5.44434502
6.77739567	7.12415491	-2.9534214	7.58640925
8.18382078	7.92439228	6.16614885	7.36493636
10.741045	10.218355	9.59828469	9.49739858
6.43482995	5.38560263	-1.8572999	6.14294526

6.71589897	5.81545137	-2.9534214	6.85387443
6.63708808	6.66895513	-2.9534214	7.01297532
8.23851793	6.17806285	9.49369946	7.88671182
-2.4069576	3.81698783	5.26281438	3.83765084
-2.9534214	4.9053372	5.59641984	5.71999956
6.89808569	6.53534177	-1.2416692	7.53723887
6.11418309	6.81169661	-2.9534214	6.11640728
7.11716925	7.62881614	2.55036907	7.72304888
9.21660308	9.10868553	8.29410496	8.00673985
-2.9534214	4.51921611	7.92793844	-2.9534214
6.59156711	4.9619847	-2.9534214	6.43464272
8.25377222	7.4282177	9.35641283	8.67606719
-2.9534214	5.05013556	5.7201688	5.46428915
7.52552333	6.77048139	8.70188692	8.15445683
-2.4069576	5.43191411	5.75197564	6.3923283
-2.9534214	5.25155252	6.0669038	3.69302268
5.12549468	3.83915232	-2.9534214	3.86791235
5.69301246	6.07443521	-1.8572999	5.35903067
6.32891891	6.04016437	-2.9534214	6.22268043
9.38692574	9.99649498	8.19670785	9.22507003
8.35154267	10.3089877	3.46890427	10.2126234
4.64612059	8.18994235	7.89804386	8.12930328
7.07834728	7.08874771	2.10952377	7.33884113
7.0488534	7.10713087	-2.9534214	8.24550989
4.87964103	-2.9534214	8.94270876	4.15236829
7.44265507	6.80114986	8.64426238	8.14290197
3.20557665	6.68204357	7.85795713	5.85075642
6.02874415	6.61377441	0.53716524	5.5507753
5.61115206	5.79858358	-2.9534214	5.16855413
6.83412439	7.20638618	8.65297519	7.91098474
5.87939111	6.01726141	-1.0106329	5.46676289
7.18298626	8.74702615	4.79794728	7.83499179
5.81838147	4.98949561	-2.9534214	5.24836237
8.3612258	8.70068094	9.8580401	9.28552122
6.07145845	4.79059657	-2.9534214	5.69911188
6.74417737	8.13724901	0.21080887	8.04862433
6.82658746	7.2095799	3.42024471	6.24734983
5.49613854	5.21811499	8.76997514	5.63457063
-0.2472184	5.35131312	6.05579703	5.87144181
6.23183296	5.82242182	-2.3036717	6.1021576
4.30364111	3.76583651	7.76748696	5.30475742
5.81076163	-0.96875	7.50996214	5.84886114
5.41201782	7.05541097	-2.9534214	5.29921609
4.45990662	7.16585634	1.07869645	3.87537958
-2.9534214	5.76713757	7.31480338	-1.8332537
6.54548142	6.5804462	-2.9534214	6.90766745

3.26923333	3.29850066	-2.9534214	1.94169689
6.00490117	7.98217935	8.8714229	7.54486846
4.81712171	4.20615531	-2.9534214	3.62377553
-2.9534214	5.96530542	6.57239588	1.39469903
5.3418472	3.39142674	-2.9534214	4.21844721
6.42388284	6.12605292	-2.9534214	6.45222599
-1.2298595	4.53627482	5.24056548	6.17657946
5.17619094	4.79059657	-2.3036717	4.2066558
6.4218835	4.27281076	-2.9534214	6.17657946
6.79140123	6.96822052	-2.9534214	7.67839128
6.5727686	4.52606382	-1.8572999	6.25881459
6.3321162	6.39744508	-2.9534214	6.42704092
10.0910478	8.78926302	10.4701568	10.4698627
-2.9534214	4.28093025	7.75233276	-2.9534214
6.09924786	6.37675913	8.39607454	7.10953435
6.17835803	5.82935875	-2.9534214	6.04701836
9.23280256	8.2764955	8.57454499	6.43337853
5.7241744	5.50651921	-2.9534214	5.30752013
6.34377937	-0.8224059	7.45727418	6.22268043
6.65336886	6.26658874	-2.9534214	7.06859165
6.24093394	3.48576994	-2.9534214	5.86207611
5.19270139	5.65603304	-2.9534214	4.50986995
5.61115206	5.95899367	-0.2116394	4.93839302
4.17184697	5.1287172	-2.9534214	3.06823308
3.44490803	4.63142484	8.56086147	3.80674094
6.22382224	3.6069818	-2.9534214	5.84506309
5.90395015	5.27816119	-2.9534214	5.59893044
6.43383819	5.78151623	-2.9534214	6.46589264
5.58815821	7.06072615	-2.3036717	5.69278614
6.83036084	5.80844707	-1.5168928	7.20300464
5.29891764	5.26594122	-1.8572999	4.44611116
7.43027632	4.85727556	8.54250725	7.25688345
3.87137663	3.31440997	-2.3036717	2.46163224
4.87380218	7.26946505	7.80303072	7.45863812
6.09924786	8.09958574	-2.9534214	7.13464302
5.4220551	4.29703342	-2.9534214	4.56170256
5.43202302	4.93907891	-2.9534214	4.66441169
4.55053626	3.47165113	-2.9534214	3.38309624
1.73962402	3.24969219	5.02318941	6.08293642
6.46329857	8.43497203	2.70461738	7.45116204
6.86241093	6.16276564	-2.9534214	7.56809418
6.49312186	6.79123618	-2.3036717	7.07509137
6.04702005	5.13320901	8.0124355	6.78715185
7.29208497	4.09080837	8.74079787	6.77821691
5.54104097	4.4949899	-2.9534214	4.73588764
6.66862511	6.04969999	-1.5168928	7.00958613

5.87501343	6.61055656	-2.9534214	5.84696336
7.48444465	0.7748727	-1.8572999	6.27866128
4.78325925	5.37046337	7.85978289	6.18411546
5.38558931	6.62338511	-2.9534214	5.23100482
6.01156384	4.35586839	7.50996214	6.91402613
5.83350148	-2.9534214	7.45365198	5.85264921
4.99440728	6.84562168	-2.9534214	4.84259657
10.3736584	10.5033039	11.687332	11.0378546
5.94211623	5.85405912	-2.9534214	5.79475643
5.61640669	6.05799227	-2.3036717	5.4593289
6.59334473	7.51811455	1.86966605	7.05303643
6.93079205	5.82103042	-2.9534214	7.6858424
6.5637301	5.4482289	-0.0938779	6.10374787
3.16789923	3.77736123	6.33840691	5.70331368
5.80156437	5.92312832	-2.9534214	5.64334479
6.68372172	8.12461554	2.81413349	7.5194766
6.17242428	5.4373729	-2.9534214	6.07647202
8.31591989	7.91724759	9.22730294	9.12682152
5.79075959	5.2597922	-2.9534214	5.43931561
7.56455878	7.88365187	6.09041993	6.61161224
5.60939626	6.35384913	7.95935634	7.15088624
6.20534324	5.93088917	-2.9534214	6.18711887
8.495378	8.84286484	7.53474481	7.74104293
6.52047375	6.66197759	2.22734526	5.91737946
-2.9534214	3.54090271	4.74361051	3.10680874
6.501666	6.64084016	7.57808648	8.33081643
0.02980441	5.40616306	6.65567691	4.18277985
-2.9534214	7.39820728	6.8755512	5.96365194
7.88936818	7.93825557	6.68377702	6.54528047
6.09799625	5.09917447	-2.9534214	5.92819021
5.88375555	5.95265419	-2.9534214	5.76088898
8.33118049	7.07888553	8.58506806	9.040198
6.22496936	6.73988329	-1.5168928	6.52287067
5.84548442	4.8408925	-1.5168928	5.20457055
6.06250292	6.56805798	-0.8115387	6.05032084
7.27058202	6.34516321	3.38009974	6.20649012
6.69783601	4.93136211	-2.9534214	6.91674272
6.09172186	6.96822052	8.14935106	7.67358087
5.70292598	3.01673495	-2.9534214	4.86151971
6.44273964	5.59872307	9.05134934	6.29962691
5.27475028	6.51655382	-2.9534214	5.13783798
4.38799097	5.3666536	7.82702712	5.9083082
5.97793844	5.11060876	-2.9534214	5.78090716
6.49216938	4.73553257	-2.9534214	6.47820551
5.65946659	6.6510561	7.40571664	7.9600956
4.33797509	5.94628673	-0.8115387	3.40389911

7.63916701	7.49866432	8.71707397	8.66264344
6.11912734	4.72366742	-2.9534214	5.94780194
5.70621541	5.81405322	-2.9534214	5.52726678
6.61713073	6.47734458	-2.9534214	7.2052285
6.22611556	7.01703863	4.48433482	4.36356168
4.94514242	2.43840191	-2.9534214	3.81453072
5.95184851	5.37236449	-2.9534214	5.81236949
6.05864775	5.09917447	-2.9534214	5.91194355
-1.7015481	2.18511063	3.38009974	5.31577664
6.04572228	3.74837496	-2.9534214	5.62574279
6.71426619	5.33385782	-2.9534214	7.07265741
-2.9534214	2.61578213	3.74660462	4.16460995
8.48751351	7.4929962	8.94141616	9.17697081
6.05864775	6.19104686	-2.9534214	6.07970784
-2.4069576	6.23559221	6.8881296	1.7919177
7.6248985	5.2096331	1.81047971	7.21556143
6.29655009	5.88101693	-1.8572999	6.34201375
5.18800337	4.18467771	-1.5168928	3.9409004
5.3418472	4.08154148	-2.9534214	4.53837287
8.04393118	7.28468688	11.0246275	7.22728058
-2.4069576	6.5001286	6.30797079	6.41428158
6.06378569	5.72758379	-2.9534214	6.03373231
5.38148007	6.75380573	-1.5168928	5.35101871
6.74817227	5.85949113	-2.9534214	7.33003676
7.85013292	8.33420735	7.88689558	5.86395412
6.5763682	6.9832268	7.6916106	-2.9534214
5.83800676	5.84724022	-2.9534214	5.74670913
4.4715516	3.42111796	-2.3036717	3.25155631
-2.9534214	3.29850066	4.43129883	3.36198902
6.81368361	7.31663309	-2.9534214	8.1120239
6.79526765	7.28468688	8.25266954	8.26908676
5.06528706	5.67309983	-2.9534214	4.50506423
4.05286452	3.30647725	-1.5168928	2.44165109
6.32785157	7.22807038	2.00780786	6.32847616
5.80463664	5.60196739	-2.9534214	5.71168074
5.04464625	7.41582754	-1.8572999	5.28246323
5.77361547	3.38390747	-1.8572999	4.66441169
4.1430086	4.62186772	-2.9534214	3.18099711
6.36370537	5.73203259	3.31770671	4.00357438
5.34817786	6.14174226	-1.5168928	5.13473009
5.29236659	6.61938843	0.21080887	4.95962491
6.9371053	7.72568887	-2.3036717	8.56067245
5.8823022	5.23074506	-2.9534214	5.74874338
6.8879826	7.59053935	-2.9534214	8.35505433
7.32401782	7.56749108	10.180339	7.15319184
6.53349479	6.80114986	-2.9534214	7.27039856

6.49026253	7.32843287	-2.9534214	7.49180916
5.90681215	6.4949026	0.6083965	5.57161034
4.82016107	-2.4449602	0.0149924	6.69837111
5.78454891	6.1495234	-2.9534214	5.77692569
5.50182837	6.03896797	-1.2416692	5.28526892
6.38027431	7.6956345	-2.9534214	7.50028599
5.84846467	4.97455473	-2.9534214	5.63676918
-2.9534214	6.21561094	6.24649802	5.5577538
5.04464625	5.56753259	-2.9534214	4.46603105
7.18062329	9.62243023	4.9254582	8.53083889
4.20477896	4.50542242	-2.9534214	3.26298783
5.03943957	5.38748391	-2.9534214	4.4105755
8.22367459	8.00900595	6.95285264	6.96303531
6.0904637	5.55251944	-2.9534214	6.12426337
1.95402673	6.12153873	6.99582683	5.81431329
4.40022863	4.69660858	6.21117963	7.25115478
3.67719993	4.95692586	0.29957556	1.94169689
5.97929861	6.36152629	-2.9534214	6.11640728
4.90562732	3.73069946	6.25345911	6.94096458
5.47893316	5.85133543	-2.3036717	5.34565257
5.62338323	6.45509223	-2.9534214	5.67791734
4.42039683	6.26146868	8.27985687	5.66720215
5.61815399	7.06072615	8.69959501	6.65021274
5.90538186	8.11528018	-2.9534214	7.03397923
6.70443056	4.62824616	-0.8115387	6.41939886
5.39377285	-2.9534214	-1.5168928	6.82620658
4.89413561	8.02238582	-2.9534214	5.5507753
5.94490359	5.74822848	-1.2416692	5.80847402
7.90161533	7.03575272	6.27140232	5.63896438
5.97793844	6.75453478	-2.9534214	6.2530936
5.535504	4.79910194	-2.9534214	5.05477378
5.66454847	5.56587216	-2.9534214	5.54844161
6.16646603	4.6996403	4.03214761	-2.9534214
6.73936884	6.62018865	-1.5168928	7.74461496
6.07273329	3.77736123	-2.9534214	5.87144181
8.02495742	8.24389731	7.36323023	6.62274674
6.68038054	7.70056114	4.62838304	6.18411546
5.21365521	-2.9534214	5.63956857	5.76088898
5.88955434	5.01405828	8.04288816	6.50853563
6.11665733	5.35516356	-0.2116394	5.66504952
5.76890422	6.1495234	-2.9534214	5.81431329
5.95184851	4.39753848	3.4784423	-1.4884482
-2.9534214	3.98544371	7.42926876	-2.9534214
7.29427239	6.30491851	4.33483211	5.44434502
1.35986526	6.54548755	7.7944652	3.96209569
5.83199659	5.45362641	-1.8572999	5.74670913

5.49613854	4.74730093	-2.9534214	5.03156838
5.18329	6.66508289	-1.2416692	5.19565039
5.29017628	7.72233884	-2.9534214	5.8936745
-2.9534214	4.55313418	5.34331917	3.84527593
6.28889171	7.0730526	-2.9534214	7.04146641
6.51860397	5.71115314	-1.8572999	6.90766745
-2.9534214	5.40058467	5.91010845	3.42440627
-2.9534214	6.70937439	6.39238138	6.14449121
6.55189475	6.40953062	0.97470261	6.53588716
7.41226224	6.73324126	8.80023876	7.77237739
5.85588847	3.18193433	-2.9534214	5.39324406
5.26362984	6.22616188	-1.2416692	5.14093919
6.16048307	5.01405828	-2.9534214	6.23724288
3.51410735	2.93676833	-2.9534214	2.55758639
5.60587822	4.72664287	7.35871829	6.83580728
8.23965343	8.39110628	10.0968815	8.57364071
9.41257506	10.3614365	8.62989055	9.38451143
5.47315213	6.45149979	-2.9534214	5.5577538
5.65266279	5.51170338	-1.5168928	5.44434502
7.46758307	8.31777631	7.42001775	5.92819021
8.30104012	7.50344309	8.69013428	9.16388154
6.8006633	8.26554059	4.34529565	7.35895614
7.21393565	8.50812683	9.43428978	8.3020144
-0.1488474	3.53412523	6.11661623	2.73224021
5.97385022	4.10916546	-2.9534214	5.85642736
6.52513762	6.26556618	4.39146455	3.94800015
6.43383819	8.7450099	6.06848352	6.38319071
5.29673726	4.64406997	6.88095538	7.01720063
6.96826366	6.85380965	8.02672677	8.22848227
9.24458648	12.4200072	9.50249127	10.1556923
4.78947547	5.01162075	-2.9534214	4.1585021
5.81228882	6.94477563	0.91975246	5.76892962
5.68469884	5.3987204	-2.9534214	5.63676918
4.53951405	5.39311309	-2.9534214	3.95506514
10.1569885	11.4125257	8.77409557	10.8764611
5.93792507	6.05088753	7.80918545	7.21482582
7.30949214	6.78910293	8.36298637	8.11281383
8.18588402	8.52681077	6.54542375	8.05068697
5.67297869	3.83364304	-2.9534214	5.23391229
9.3173764	9.45827677	7.89225751	9.11148966
5.08057616	5.82797403	-0.0938779	4.38986938
5.48660534	6.58373183	7.8377198	7.20300464
7.4651581	6.61618309	4.38640703	6.34066569
4.61845352	3.35343223	-2.9534214	3.80674094
7.0488534	7.48774425	3.10019053	7.65471976
7.46806757	8.09958574	5.74606502	7.19780227

4.2817599	4.92360381	-1.8572999	3.43455156
7.66306191	8.60610006	10.3400946	7.94686667
-2.4069576	5.63718155	5.65226549	5.71168074
8.39065814	8.4557424	6.63556945	8.1742686
7.11097787	6.66740748	8.89809765	7.37682294
6.28889171	6.93323076	-1.5168928	7.07103248
5.50372	3.86098143	-1.5168928	4.56632356
5.21365521	4.64721401	-2.9534214	4.71938556
6.11542074	6.3168141	-2.9534214	6.46959755
5.740309	3.71879433	7.72854956	6.16443937
5.8910004	5.62287949	-2.9534214	6.04039063
6.72322366	7.0933655	3.3070404	6.42704092
-2.9534214	5.76424456	5.75590263	5.71999956
4.49836318	4.20188529	10.1899863	3.08120658
6.93570474	8.96427112	4.43129883	8.15675673
2.86062093	4.48448139	-1.2416692	1.76000118
4.18605283	2.95717935	6.77947639	5.45185642
6.73856586	6.34225624	6.79578083	8.85337542
6.36786548	6.57962362	-0.3398746	6.88102165
5.41604111	5.34938403	-2.9534214	5.23681391
5.54104097	5.97784668	-2.9534214	5.65206591
4.7453887	6.1660573	-1.5168928	4.50024244
5.77831138	6.47734458	-2.3036717	6.10850817
6.84982493	6.64870499	-2.9534214	8.30028173
6.23639063	7.81506699	3.83835276	6.25738647
5.75943532	6.16934146	-1.5168928	5.92819021
2.7245002	8.28333189	8.13547621	5.85264921
6.38130358	7.85300429	-2.9534214	7.84691022
5.43400837	5.34551812	-2.9534214	5.29643742
4.13326627	5.76134574	5.09315077	-2.9534214
7.44265507	7.10484574	8.51641261	8.2509022
5.63895817	6.6455642	-2.9534214	5.94247967
6.43284574	6.69121149	-2.9534214	7.33546119
5.31624305	6.40675062	-2.9534214	5.48396131
7.89982079	8.83926105	7.01315663	7.68105681
4.97544811	6.37296597	-2.9534214	5.03490637
7.42579384	6.46759581	7.42001775	-0.7767262
-2.9534214	4.98701618	5.35372156	5.2134359
-2.9534214	4.82432125	7.78584852	-2.9534214
5.54472051	7.10941238	-1.2416692	5.9810612
5.75943532	5.94628673	9.15163364	5.57848902
4.59024548	4.63459652	-1.2416692	3.61488117
5.39377285	5.28018783	7.20633912	7.0571462
5.19504468	7.22123012	8.00585489	7.12918772
8.21417494	7.82759298	8.80261366	9.19815366
-2.9534214	3.74250715	7.13651499	-2.9534214

4.17659782	4.34429178	-1.2416692	3.10680874
-2.9534214	3.52047402	4.30297829	3.9048679
4.45990662	5.41909604	-0.3398746	3.53226365
5.76733038	6.24599856	-0.8115387	5.87703219
5.92245248	6.17261816	-2.3036717	6.28147436
4.20942276	5.08301267	-2.9534214	3.58786429
5.77204676	7.78790454	-2.9534214	6.83962974
4.85910083	4.34429178	-2.9534214	4.27034749
6.0904637	4.61866789	-2.9534214	6.28287885
8.22195202	6.9312976	8.88829209	8.45748684
7.29645649	7.30274358	9.10101261	7.70482756
5.71277185	4.74436782	-2.9534214	5.66504952
5.39784727	6.84082381	-2.9534214	5.73649465
6.58264598	6.38713918	2.22734526	6.19609178
6.25223001	6.53279414	-2.9534214	6.99251972
6.24772219	7.62603014	8.5175696	7.65091777
6.26789738	5.957728	-2.9534214	6.78417968
5.62859358	4.45965971	0.86262618	3.89755213
-1.7015481	5.17519789	5.62031118	4.82342191
6.4969255	6.40767788	-2.9534214	7.42213939
5.1007128	6.33154696	7.39568377	7.23528262
5.92668876	5.12421135	-2.9534214	6.14139764
5.29236659	4.04386697	-2.9534214	4.88760193
5.95461714	5.41356752	-0.0938779	5.69489781
5.42005322	5.55419531	-2.9534214	5.41646374
6.03921585	6.48263446	-2.9534214	6.57653883
6.77973939	2.31730788	-1.5168928	6.15526679
5.88375555	6.94987718	-1.0106329	6.47329296
5.98201511	7.88996948	1.50922995	6.93114545
6.40275029	7.38178547	8.27883374	7.85022981
7.11159821	7.75221268	8.5720414	8.3815162
5.92527805	6.48527214	7.84280581	7.34759209
6.33105123	5.13544968	-2.9534214	6.77022801
5.43003494	6.53109322	-2.9534214	5.74874338
5.97793844	6.83394191	-0.6366176	6.52524606
5.71440631	4.35970671	-2.9534214	5.68218123
7.20349882	7.91464072	6.43341722	6.10056558
4.45209075	6.76759509	4.80175128	2.02462691
6.58532812	5.60358682	8.84332316	6.38449963
6.62323398	5.94500985	-2.9534214	7.6536345
8.88915627	9.40180675	6.96307998	9.40851445
6.53903946	6.7618051	-1.2416692	7.73130259
4.43633082	6.99190892	7.24875863	7.43672369
6.7529516	5.45182948	-0.8115387	7.36957056
5.83950539	5.77146624	-2.9534214	6.15526679
6.09924786	7.6918333	-2.3036717	7.50811299

2.55971076	5.13544968	6.40243717	5.88260098
5.17143875	3.3763488	7.65632951	5.58077464
6.06121901	6.20924313	2.22734526	5.32398617
6.07782139	4.05811045	-2.9534214	6.30239944
5.57202195	-2.9534214	-2.3036717	7.26614429
5.61815399	3.91415149	-2.9534214	5.43173847
4.86793961	6.32665271	6.36178652	-2.9534214
5.52250116	4.72366742	-2.9534214	5.45185642
-2.0115616	5.15546067	5.53375784	4.65576511
5.09570493	4.33652211	-2.3036717	4.65142231
-2.9534214	2.91606439	3.73072955	1.91295928
5.30977038	2.05858306	-2.9534214	4.73998384
6.06890539	6.81029482	-2.9534214	6.90675677
8.8672835	8.84697247	6.87284151	9.01485459
4.44423231	4.74730093	3.91761877	-2.9534214
3.82975838	5.23701887	-2.9534214	3.35131846
9.76569834	8.38852537	8.14897782	8.24730956
5.82293414	6.35095963	-2.9534214	6.29545808
5.72903376	5.00428334	-2.9534214	5.94070121
7.27944255	5.37994401	1.92651965	6.93918424
6.96826366	8.87339072	5.11777295	7.91958785
-2.9534214	6.03417244	6.03332363	3.02859755
6.20418041	5.9114082	-1.0106329	6.57653883
4.96450137	4.63776126	-2.9534214	4.65576511
6.51298005	6.81239699	7.91482595	-2.9534214
6.72727693	7.05954669	3.42024471	6.53470868
6.17004393	4.40869723	-0.48063	5.804568
5.55934554	6.74429438	-0.3398746	5.86582969
3.42906901	6.00628442	-1.5168928	3.08120658
-2.9534214	5.81963768	5.42697596	6.89302721
5.36700485	3.55436287	-2.9534214	5.07761183
7.11716925	6.26351888	2.75257428	6.86516979
6.08668263	4.40869723	-2.9534214	6.41684249
4.29492841	5.46436125	6.9304415	6.62938643
5.60587822	6.05444427	-2.9534214	5.96889681
5.2388589	5.84860658	-2.9534214	5.38280302
9.14856298	8.15364702	9.68605501	9.46574921
-2.9534214	4.51921611	4.61978935	6.15986037
6.61187874	6.66585817	7.50588715	8.23865056
6.48739752	7.04888794	-2.9534214	7.94819503
4.65636047	6.48087332	7.0229667	7.42976698
3.62253849	-2.9534214	5.87803765	4.24751088
5.59171967	5.56420981	-2.9534214	5.90283794
6.3321162	6.63213903	0.86262618	6.76017928
4.93396239	5.91793113	-2.9534214	5.00117368
4.1907572	6.74942367	7.38811306	6.84153718

4.99440728	2.43840191	6.38732709	5.96014474
5.73709649	3.85555504	-2.9534214	5.80261102
6.78051978	5.28221162	-1.2416692	7.64819591
6.64739203	6.77120207	2.05956205	6.94185392
6.0560719	6.64870499	-2.3036717	6.94540582
5.22287121	4.54641406	7.33463221	6.29684903
7.95409336	7.93246854	5.42204162	8.18482485
7.06559787	6.40675062	8.67440011	7.26969039
6.5646365	5.96530542	2.03391701	6.08293642
5.50560916	5.55251944	-2.3036717	5.80065138
4.08859285	-2.9534214	5.01009646	4.77633546
4.16229809	4.27687621	-2.3036717	3.65013424
6.86019783	6.55807025	7.58085635	-1.2103612
5.740309	5.04299191	-2.9534214	6.06997853
4.43633082	5.46969886	-2.9534214	4.24751088
4.09362543	5.82103042	7.59517462	5.8393472
6.65507199	6.61938843	2.49505681	6.60264234
5.72579601	5.6132654	-2.9534214	6.13363455
6.7592995	7.4149055	7.7815208	8.60092275
5.93652531	4.51578003	-1.2416692	5.86770282
6.25335477	6.87474932	-2.9534214	7.40997928
9.31643501	10.3998062	8.47651251	9.58951799
-2.9534214	6.95939423	6.40868663	5.08728996
1.6330477	1.7893642	4.58489335	4.24751088
5.66623846	6.81729018	1.616789	5.62352736
3.72986571	3.49975191	2.70461738	-2.9534214
2.94100092	5.59384287	-2.9534214	2.57603449
4.37151092	6.17806285	6.29723199	7.86155362
8.24785914	8.3514175	7.2536273	7.47162875
7.5231939	7.24684509	8.58589557	8.23828864
4.52095449	5.4446193	-2.9534214	4.3529028
5.54104097	6.30392277	-1.0106329	5.89551185
8.88244746	8.94587717	7.85108988	8.30616435
5.8910004	5.13544968	-2.9534214	6.31755388
3.65012807	7.27455686	7.10991388	7.28869012
5.12549468	5.94373185	-2.9534214	5.342962
5.80617032	2.34854753	-2.9534214	5.72414105
6.28339644	5.45182948	-2.9534214	6.97611748
-2.9534214	4.0628272	7.18319485	-2.9534214
5.46346531	5.75261416	-2.9534214	5.83743686
4.65975767	4.77055351	0.80314429	3.15668909
-2.9534214	6.29593201	5.64804559	7.02141361
4.85614249	6.09645502	3.04989883	3.20490233
5.78610409	7.56292015	-2.3036717	7.04726302
5.28798264	2.7387786	-2.3036717	4.66441169
5.66116254	4.81316706	-2.9534214	5.92459562

6.53626979	9.22002973	5.832278	7.40740614
3.60149444	6.31582654	6.38352471	-2.9534214
6.9209162	7.67190396	8.52045802	8.10767152
6.45452345	5.19681602	-2.9534214	7.33003676
6.43681144	6.42701395	1.17569549	6.74904422
4.91986442	4.20615531	-2.9534214	4.66009488
6.15206502	6.13167586	6.95028444	8.05439231
7.59237996	6.11587606	5.99230062	3.75909775
7.48060791	6.61858776	7.66679617	8.58279216
3.67047944	2.50922903	5.33546786	5.73444305
9.16494261	8.87975332	9.98767775	9.77348785
-2.9534214	5.49434987	5.50371302	4.72352881
8.53499519	7.98962975	7.04560075	7.61456904
7.61705578	6.62178777	8.19164291	-1.2103612
5.85440676	6.11132987	-2.3036717	6.4634174
5.05500357	3.57432054	-2.9534214	4.76431974
5.63032619	4.67828322	-2.9534214	5.8393472
-2.4069576	5.12195314	5.21226299	5.15020303
5.01842289	5.10146859	1.84037637	3.26298783
6.14723256	6.39557674	7.99509698	7.16085068
4.22326514	7.78790454	-1.8572999	5.11908941
3.69717557	4.65035121	6.71739456	5.78090716
5.47508172	4.26054518	3.80083084	-2.2872455
9.05168225	8.38476307	8.36812651	7.23528262
5.08310866	4.64406997	-2.9534214	4.97360788
4.5938018	7.35319146	-2.9534214	5.22809149
4.59734937	5.58731014	7.86524634	5.70750327
6.82280417	7.39025431	2.13387043	7.99264677
8.41121161	9.24890323	7.13500807	8.72725798
4.05286452	5.91793113	6.5363198	7.19183363
5.91109454	5.9114082	-1.2416692	6.35007574
5.6863654	6.95622885	-1.0106329	6.46712867
6.03529787	6.5001286	1.43279367	6.06997853
6.78207931	5.23701887	2.18136476	5.80065138
5.22974479	4.85998807	-2.9534214	5.22517226
6.34272297	7.97562853	3.67374938	7.00618896
6.62236367	5.40801774	9.4142408	5.72414105
6.82507533	8.98755888	5.45864424	7.76032852
9.5049514	8.36484836	7.43601533	8.81869475
3.52159522	5.36092002	-2.9534214	3.2400335
-2.9534214	4.17163599	7.20633912	-2.9534214
4.5687215	4.18034356	-2.9534214	4.26467214
6.55280862	5.75698656	5.44897398	1.58888154
4.99977873	6.78554048	8.48895279	5.86395412
6.44667828	3.35343223	0.21080887	5.32125485
6.72403523	3.58747391	-1.8572999	7.22946736

5.65266279	6.81589382	-2.9534214	6.41939886
7.10475981	6.61055656	8.72185006	7.31638579
5.46928521	6.1660573	-2.9534214	5.99997183
7.43524057	5.11742609	3.32829474	5.79081305
8.1485889	9.62781181	9.29970488	10.2974085
6.05478225	6.73324126	8.62426262	6.61607636
-2.9534214	4.34429178	7.31546875	-2.9534214
5.85440676	5.01892103	3.91059006	1.7919177
4.52840699	2.70296801	-2.9534214	4.14620829
7.32455301	7.00238173	7.58085635	3.13196448
5.11810471	6.81938218	-1.2416692	5.67578065
6.35744263	6.27270892	0.11622035	7.00533842
-0.0567579	6.30591357	6.24230518	5.59893044
4.9589967	5.80704212	-2.9534214	5.18065978
9.34150704	8.72189779	9.94284249	9.81921464
-0.3527908	5.42093418	5.23494923	6.3923283
4.63580752	5.0334117	-1.8572999	4.39507384
6.33530643	6.83738696	1.5459812	6.85387443
4.84125914	2.09588014	-2.9534214	4.42591237
6.1326373	6.32763289	-1.5168928	7.0197299
6.86314787	-2.0697503	7.6142275	5.84696336
5.53735202	6.57302604	-2.3036717	6.24878792
-2.9534214	3.12894223	4.1442542	-0.7767262
6.1326373	5.88235169	-2.9534214	6.99166108
9.90345111	9.25200431	10.3913272	10.4084578
5.77361547	3.47165113	-2.3036717	5.78090716
4.35902616	5.34745237	-2.9534214	4.26467214
7.11963838	7.25459635	9.5555546	6.97089884
5.60411598	5.33969975	-1.5168928	5.91918688
5.50938007	5.35131312	-2.9534214	5.96014474
5.57742078	2.48131403	-2.9534214	5.48640158
7.1537697	7.33673303	6.7938722	4.83878197
6.80986629	7.35319146	8.25197456	8.01563703
5.24339446	5.5575412	-2.9534214	5.56007248
8.01504461	8.11101678	7.38240884	6.75715096
5.69632454	5.40058467	3.62305148	2.96001808
6.36162081	5.79999678	1.47151796	6.13363455
5.03682917	5.58239112	7.30210269	6.64476122
5.73226426	6.26556618	-2.9534214	6.43464272
4.85020756	4.92619455	-2.9534214	4.84259657
8.06778233	8.30165489	9.17711723	8.95444318
6.15808292	7.99827354	3.57050692	6.89486539
9.35976813	8.43724422	8.51119465	7.53252361
5.6251221	5.81265373	5.832278	-0.7767262
5.4595723	5.23910409	-2.9534214	5.86957353
5.76890422	4.21465761	-2.9534214	6.21828288

9.49095396	10.1445608	8.32152355	9.75298575
5.95184851	6.49053309	-2.9534214	6.90766745
5.16905678	4.43075909	-2.9534214	5.18065978
5.40191022	6.2573595	-2.9534214	6.01863778
6.3160584	6.83463158	-2.9534214	7.84263092
-2.9534214	4.52264403	7.39190338	-2.9534214
4.99977873	6.4983887	-2.9534214	5.5898811
2.84876357	4.10001611	4.67889716	6.94629243
6.14116913	5.52543744	-2.3036717	6.92306162
5.43003494	5.70663938	2.65501136	4.03057878
5.34607072	7.86520655	-1.8572999	6.76822386
-2.9534214	3.63258865	6.83992183	-2.9534214
6.19484384	4.65348161	-2.9534214	6.91402613
6.25896545	5.29629924	-2.9534214	7.18884003
7.43375309	5.87432456	9.25444755	6.71823168
6.08162573	7.1099822	7.59681762	1.55207983
4.87964103	4.56315603	-2.9534214	4.87648153
4.17184697	4.44888971	-2.9534214	3.96909215
6.66271128	5.60034614	8.10154465	6.91311946
7.6387367	7.50517691	6.29047942	6.55461302
4.99440728	6.28588092	-2.9534214	5.55310522
7.19825195	9.00894993	9.0644707	8.94049479
-2.9534214	7.63595552	6.62057202	3.9048679
3.60854328	4.63776126	7.37029202	4.85775489
5.29236659	7.20904811	7.17221681	7.98231008
-2.9534214	4.83263067	4.64117835	6.07322893
4.32946789	4.91057988	-2.9534214	4.22430697
3.64327998	3.45739279	-2.9534214	3.39353517
6.5288579	5.23701887	6.42604245	8.04158921
10.3034506	9.93439208	11.1729345	10.6458832
8.7092513	8.37673544	9.30054529	9.47376532
2.90709921	5.8252006	8.98368114	2.68202765
5.55387852	5.46792185	-1.5168928	5.98279063
4.99977873	7.97312514	-2.9534214	6.33931637
6.64224926	4.18899889	8.04007295	6.32983565
3.7992784	3.77161038	-2.9534214	3.59692625
9.77306924	9.4598422	8.82661694	8.93915932
6.17361298	5.86084595	-2.9534214	7.20003415
4.02679147	4.66593571	-2.9534214	3.87537958
8.52455205	7.28871906	9.37248803	8.39261717
6.77034157	6.63213903	7.02946989	1.94169689
5.67801327	6.77838909	-2.9534214	6.67395772
-2.9534214	6.50533574	6.09352665	2.48134042
5.18092752	4.89479435	-2.9534214	5.39584256
-2.9534214	7.19622581	6.25345911	5.73033107
5.00513027	-0.8224059	3.77784014	5.87330768

6.36890364	7.12415491	3.27455999	6.44847615
3.86550417	5.53734842	4.69534966	-0.7767262
5.59704542	4.09080837	-1.5168928	5.49854138
7.76712776	7.90515114	5.13596768	8.29889409
9.608015	8.75906485	10.2070291	9.83018327
-2.9534214	4.34429178	5.95350064	-2.2872455
8.93193142	9.70442434	7.9567997	9.11030351
8.09030175	7.50647591	8.83081167	8.60738317
4.98360399	3.63258865	-2.9534214	4.94904802
5.25915756	4.35201983	-2.9534214	5.40876529
5.38148007	4.34429178	-2.9534214	5.62130853
5.22745723	5.61165681	-2.9534214	5.69911188
5.43599099	5.76279588	0.67627567	5.29921609
5.2569162	5.76569179	-1.8572999	5.76088898
4.9311538	5.1882076	6.77464569	6.79504784
2.7245002	5.11288479	8.13359096	3.26298783
6.17598746	6.48263446	-2.9534214	7.48205996
4.5687215	6.62577782	0.0149924	4.92045792
5.24112847	5.62287949	-2.9534214	5.77493082
6.64910223	6.19212361	3.43990592	5.73854334
7.9151814	7.62841847	8.45191076	8.89805092
7.7781103	6.52512414	9.03002266	7.49059411
8.36902956	8.98321124	8.29612897	7.44615638
4.94792394	1.74309531	-1.8572999	3.86791235
5.70949735	5.44642523	-2.9534214	6.40657145
5.88520743	5.15103771	6.87464854	7.27039856
5.09067961	7.35895578	8.45975683	6.30516666
6.78363715	6.4030356	3.41031271	6.29127718
6.29873075	5.07369541	2.6380896	4.82727728
3.54382842	7.247881	-1.5168928	4.25325401
2.53031844	3.70679015	2.88756311	-2.2872455
6.49407371	7.11680248	8.10462753	7.69166997
5.05500357	6.09069284	6.72342355	7.50269883
5.81076163	7.6339759	8.16715429	7.48937803
5.44192255	6.44157419	7.13802034	-1.8332537
2.86062093	6.21349146	-2.9534214	3.01513998
5.19035429	4.24817444	-2.9534214	5.33756575
7.2504446	7.86824106	8.95557137	8.00163101
3.24229507	4.39753848	-2.9534214	3.14437975
-2.9534214	4.48096148	4.51246228	5.03490637
5.2569162	4.87078741	7.07889962	6.49041418
6.95657168	6.54969399	7.89759958	7.84072492
5.44389433	6.99376259	-2.9534214	6.47329296
6.14723256	6.3187872	6.68892122	8.28494379
5.00779861	6.20070876	-1.8572999	5.63236873
6.13019033	0.35009269	-2.9534214	6.30792859

3.69054771	5.71415448	7.55967068	5.2134359
5.66454847	5.81405322	-2.9534214	6.43337853
5.39988018	5.28018783	-2.9534214	5.97586045
3.70377313	8.51631741	7.57308725	7.53723887
3.78690379	5.13096485	0.11622035	3.09406445
5.92950606	6.31582654	7.20490343	7.64164242
6.98189872	4.17599634	1.68428351	5.82399319
5.41201782	3.73069946	-2.9534214	5.66504952
7.12886017	6.53109322	8.13472241	7.74665216
6.2111434	7.58440172	-2.9534214	8.18745189
5.05500357	3.6824779	-2.9534214	5.14403375
-1.2298595	5.84313336	5.41460832	5.83360858
5.42804411	6.09415291	-2.9534214	6.19609178
5.80617032	5.99275368	-2.9534214	6.69204212
5.51501801	6.7183708	-2.9534214	6.4708304
4.96998512	6.62975686	7.78007534	6.53588716
5.06015448	-1.5257463	6.57016736	4.7842907
4.89124832	7.40936087	-2.9534214	5.95486789
4.12345771	4.74730093	-2.9534214	4.10868549
8.0549388	7.3333211	7.07733127	6.07647202
8.64792356	8.76649315	7.610437	8.23430149
4.85020756	7.19192626	-2.9534214	5.78487768
4.53582119	4.89214656	-2.3036717	4.59374302
6.11046375	4.53627482	-2.9534214	6.97524901
6.09297892	4.52606382	-1.2416692	6.52405885
5.91820373	7.72977281	-2.9534214	7.79519352
6.20185193	6.75161636	-2.9534214	7.86577723
6.2723427	6.93451809	-1.2416692	7.99864247
5.58815821	6.51138708	7.4941805	7.22362856
4.13326627	6.72210281	7.81060206	-2.9534214
4.63925341	3.08324495	6.41739064	5.59441287
-2.9534214	6.52939029	5.65015709	6.49405673
5.64239655	-2.9534214	0.6083965	5.92998415
5.60411598	5.19251823	-1.8572999	6.22998003
3.74273715	-2.9534214	7.20418505	3.36198902
4.66990154	-2.9534214	5.30630855	4.83112238
3.95117581	2.31730788	-2.9534214	3.80674094
5.25242302	5.57745526	-2.9534214	5.91556976
5.7241744	7.12189661	2.31513045	6.09577895
6.72565699	6.88610462	7.77524665	7.99221755
6.06378569	4.48096148	-1.5168928	6.59135098
5.01312053	5.98158789	0.6083965	5.01137656
8.02429868	8.72077904	6.2576197	8.63514042
5.77047634	6.42792829	-2.9534214	6.85953316
4.87964103	6.65964422	7.665231	6.5992642
8.5798435	7.95642952	9.1058285	9.10435809

6.52234111	7.49866432	8.19815171	7.84975605
5.11067669	4.08618237	-2.9534214	5.33486004
4.14785521	5.8252006	7.19770347	6.07970784
7.4041832	6.80467402	9.36591885	6.99937053
5.25915756	7.27506505	-2.9534214	6.44597082
5.78299206	0.864048	-2.9534214	5.93713769
3.70377313	5.45721357	6.68686574	6.12426337
5.1738168	6.89737125	7.78920557	6.80290084
5.55570316	5.77721766	-2.9534214	6.40785934
3.79310436	2.0395666	-2.9534214	3.6414014
4.20012017	4.75897407	4.52171754	-2.9534214
7.33998776	7.55079964	5.54287792	7.02225473
2.81259531	6.29493005	-1.2416692	3.00155569
7.1812144	8.22639838	8.22779039	5.14403375
6.27345189	7.64108986	4.18560214	6.51810813
5.55570316	4.29703342	-2.9534214	6.16291465
6.06121901	7.46030033	-0.8115387	7.87232283
6.41887928	7.85130133	7.0670952	4.80398897
6.50921855	5.38560263	-2.9534214	8.30719997
6.83337246	7.00483491	3.98608437	6.76922628
4.908486	-2.9534214	3.81595691	5.12223114
11.2670513	11.9231465	11.1938925	10.7778086
4.88255162	5.17737434	6.64194951	6.77722071
4.05802306	4.58299326	6.67861442	5.71376495
8.52385315	8.51265895	9.93874569	8.74213998
5.3418472	4.25230982	-2.9534214	5.80652233
7.63183419	7.26997504	8.17340728	3.82998523
5.16905678	3.86098143	-2.9534214	5.44434502
7.13069747	7.21329701	7.57141699	8.70333259
-2.9534214	4.64091906	7.30344488	-2.9534214
8.39830472	7.82793938	8.97402079	8.92213539
5.9280981	-2.9534214	7.45968394	5.08084509
5.22516605	7.05244961	-2.9534214	6.32302536
2.84876357	8.25424414	8.20678469	5.23100482
5.39173131	5.56753259	8.0340218	5.86770282
5.65095682	0.82014925	-2.9534214	5.75280329
5.03159415	5.82103042	7.42310802	6.44220466
-2.9534214	6.83463158	5.69379924	6.7970151
5.87939111	5.86760099	-2.9534214	6.94894899
4.51346329	5.1421509	6.13029466	7.02477516
5.30977038	5.06431759	8.38121514	5.16855413
7.87336302	9.3540215	6.20974875	9.03583136
6.16765965	4.63459652	-2.9534214	7.23600789
8.88662147	8.60731133	9.67947986	9.38254762
9.52592889	9.99364165	8.28767682	9.80864958
-2.9534214	4.17599634	4.34007336	1.47553441

6.99742136	6.0976047	7.09216223	0.25072817
-2.9534214	4.8159637	7.41257399	-2.9534214
7.93704526	6.25014017	7.11221329	3.54168051
5.37322631	5.18604745	6.19246658	7.48328222
5.73226426	5.58074771	7.61854734	6.56388559
5.91251918	8.11442851	8.93666673	6.91402613
6.10548967	7.07772083	-2.3036717	8.0206966
6.75692231	4.61224686	7.46629009	6.91130441
8.19586369	8.35334387	9.07492183	9.09099085
7.00010398	5.61487221	8.2719943	6.89853473
8.99094331	8.72413269	7.45606779	8.79132436
7.17766412	6.87206444	3.21874614	7.96666486
-2.9534214	3.54090271	3.53438297	4.32590587
5.67801327	6.66275454	-2.9534214	6.92035691
5.72255097	6.43613131	-2.9534214	6.93114545
8.48440357	7.22544335	8.96874335	8.52135077
7.09035573	3.55436287	7.66314147	6.55228554
5.07549778	5.93603997	1.86966605	4.63831491
0.97973075	1.74309531	3.01099506	4.65142231
5.5243659	5.63242993	-2.9534214	6.44220466
-2.9534214	4.31691287	4.48906098	1.21802358
4.62542019	3.24139446	-2.9534214	4.72352881
4.92833972	3.01673495	-2.9534214	5.09370617
6.77112706	7.47762425	9.37647899	6.77722071
7.95097714	7.54280304	6.97828633	6.50372546
6.16527142	1.71939224	0.11622035	3.54168051
5.15708754	3.9089218	-2.9534214	5.50095713
9.01551344	9.68218087	8.28121991	9.01019848
3.91182789	2.18511063	-1.8572999	3.2400335
5.65095682	5.56919112	-2.9534214	6.61496162
2.28842343	7.36946425	7.5708598	5.02486912
-2.9534214	5.09227014	7.54556112	-2.9534214
6.4417533	6.18889096	9.47013871	5.62130853
3.84774198	5.57250246	-2.3036717	4.15236829
5.27917456	6.2335019	7.26331562	6.99766088
6.92445105	7.97469027	8.16862802	8.53585419
4.49456332	4.63459652	-1.5168928	4.46107681
4.68331671	6.7183708	-2.9534214	5.62352736
2.94100092	5.05960572	4.66643348	-2.2872455
7.59193546	8.11528018	7.89091889	5.92459562
5.98337145	6.32959125	7.58803325	-2.9534214
7.1758857	9.22055749	7.17368539	7.5194766
3.43701025	6.24807085	0.6083965	3.39353517
8.5414552	9.3778754	8.16531	8.32775786
4.89413561	6.07676897	-0.48063	5.40361008
7.00278162	7.09739399	9.19909706	6.88102165

6.77895857	6.21772731	7.73004755	-2.9534214
4.13326627	-2.9534214	6.90591061	3.62377553
4.53582119	5.10375907	-2.9534214	4.94904802
5.92950606	7.40053806	-2.9534214	7.89958611
4.37151092	4.6597221	6.78044059	5.82977011
6.35430103	5.19036453	-2.9534214	8.01816903
6.37924431	6.32665271	7.83075531	-2.9534214
5.3418472	6.22616188	7.34444536	6.91130441
7.3293608	7.52068821	8.17267304	8.43965317
3.76183151	3.25794247	-2.9534214	3.9048679
9.75263339	8.51524235	10.2112362	9.65226157
5.08816037	6.51224949	-2.9534214	6.12269557
5.47700873	5.32013394	-2.9534214	6.44597082
5.8967702	6.6957737	7.18392375	1.26426187
-2.9534214	8.0350428	6.35144233	5.66935156
5.53735202	6.3207576	7.1236557	7.35161316
3.86550417	4.62824616	-2.9534214	4.09595784
4.09864051	7.22544335	-2.9534214	5.17159008
5.27031239	6.40024305	-2.9534214	6.33931637
6.15928349	5.8362625	2.87317313	5.50577653
6.97236773	7.8272465	8.34550191	8.21493472
-2.9534214	5.56753259	6.19825034	-1.8332537
4.1430086	5.02134627	6.34624229	6.34739341
4.47541247	3.33014576	-2.9534214	4.66871562
3.06916195	5.25361685	-2.9534214	3.31882493
5.86621802	5.13768688	6.216889	7.64164242
6.5564583	4.99444171	5.35372156	0.42367165
5.71113553	-1.3152731	-2.9534214	5.23681391
6.22037541	6.09875347	7.25015136	7.49968216
8.24955107	7.40658055	6.79578083	6.99594919
6.074007	5.76279588	-2.9534214	7.56347886
3.21484424	5.88101693	6.39490191	6.36474006
6.48069029	6.49140805	1.58181946	7.35895614
7.62967037	7.27353993	6.06215422	6.72341303
4.02151984	4.34429178	-2.9534214	4.25897437
4.55419169	7.57988414	2.15781303	5.24259969
5.06784652	6.90592792	8.12716259	6.17052218
5.61989918	6.38148659	2.85863818	5.29086398
5.25467136	6.13839459	-0.8115387	6.07647202
5.96975037	7.42867455	5.00350516	5.43173847
-2.4069576	6.13951134	6.93910242	-0.6006549
7.89153701	7.64463373	8.32052956	8.87005768
4.42039683	5.5134273	-2.9534214	4.94195344
4.30797781	3.0739292	-2.9534214	4.50986995
4.83526253	5.71265459	6.76200964	6.84819342
0.58157832	5.01162075	3.11249423	-1.4884482

8.7406698	7.86216565	9.45466812	8.78067657
6.54639936	6.81659216	7.56695347	7.90598043
5.93932348	5.57250246	-2.9534214	7.29774966
3.35555935	7.55791711	-2.9534214	4.5241918
5.63551157	5.50305272	-2.9534214	6.74599242
5.32268682	4.88150651	-2.9534214	6.21387188
5.18564861	-2.9534214	3.42024471	5.14403375
4.32519541	5.59384287	-2.9534214	4.8727556
7.96683423	7.40704431	9.10775036	8.0185906
6.58532812	7.11169029	2.73676511	7.57212052
2.88404723	5.34358127	-2.9534214	3.19299923
7.25494394	7.74965527	7.9452384	8.78860798
1.95402673	6.27677467	7.78296482	3.38309624
5.86180012	5.34551812	1.68428351	5.59667342
9.92089843	10.6800543	8.27712692	10.8436467
4.17659782	3.72475918	-2.9534214	4.45610549
7.12025501	7.49779375	4.03214761	8.07440055
5.41804856	5.88501751	-2.9534214	6.52643228
7.02006621	6.08258692	7.7577283	-2.9534214
6.11046375	6.98881416	1.74876101	7.11821484
6.36162081	4.98701618	6.90148586	7.21114209
10.8733996	11.1426696	10.6210184	10.2066234
3.47607375	5.5134273	-2.9534214	3.87537958
-2.9534214	6.25220653	5.56766486	3.2400335
6.76246301	7.35078285	7.60173542	8.42063965
7.07771248	6.93516133	4.71161665	6.73986936
8.84264732	7.66105765	7.44333943	7.48267122
6.03791104	6.70032152	-2.9534214	7.98231008
8.0629794	7.94593568	8.34908079	9.22415649
4.91986442	5.70513165	1.12801103	4.7842907
5.14985805	6.12153873	-2.9534214	6.24446935
8.43395856	8.1907506	9.06585538	9.09279366
3.84177232	6.3908953	-2.9534214	4.62951001
5.0524212	3.8001381	-2.9534214	5.58305665
8.73202206	8.34924726	8.08564064	7.50028599
7.9254493	7.59624434	6.66510456	7.0188873
7.41528025	5.26594122	6.58569518	-2.9534214
5.46928521	6.9098601	-2.9534214	6.96828242
8.34417145	7.64620599	10.4802801	7.45739479
7.47868571	6.20498226	7.93791125	0.42367165
5.13528945	3.69468524	-2.9534214	5.69700638
5.02106678	4.77055351	-2.9534214	5.72620734
6.31282528	7.28972534	3.77009444	6.67395772
5.49233282	6.48351422	8.11115687	-2.2872455
1.99763475	4.52947552	6.24928649	4.12130184
4.08354266	4.39005107	7.17954478	4.92045792

6.36266347	7.01703863	4.25383874	6.11009146
3.96222373	2.67859072	-2.9534214	4.21844721
6.70360788	6.80185538	7.64895741	7.88809672
-2.9534214	3.25794247	6.26865631	-2.9534214
6.3605774	7.0933655	7.60500467	7.90461261
4.98901575	4.50542242	-2.9534214	5.61463147
5.28578565	6.77480005	1.39300121	5.89551185
4.85317808	5.41356752	-2.3036717	5.63016346
3.96771613	6.3908953	-0.2116394	4.50024244
6.27345189	5.14882114	7.03513623	7.11190694
9.99421236	9.59430106	9.39956828	8.90513619
4.10861867	4.14073883	6.24230518	5.59667342
4.77074586	6.27677467	-2.9534214	5.85642736
6.81977039	6.70937439	7.96742261	7.59886733
5.6628565	6.5370377	-0.3398746	6.84248995
4.85910083	6.04613152	-2.9534214	5.92099205
5.21365521	6.32665271	-2.3036717	6.42957931
8.74926602	8.074114	7.408214	7.9600956
4.88255162	4.43075909	-2.9534214	5.49854138
-2.9534214	4.52947552	3.86768313	6.22560471
5.56116329	6.32665271	-2.9534214	6.92935295
5.46928521	7.16804692	-0.8115387	7.03481306
2.92978863	5.51514917	-2.9534214	3.37258123
0.39849386	1.30727289	3.13679123	2.91724899
5.35238292	5.40058467	5.73417066	7.8871736
8.2652474	5.8417618	7.99468161	-2.9534214
7.2023345	6.82910498	6.74037015	4.61173735
9.28323114	9.41512453	9.0110483	8.40169543
7.59903105	5.03820976	5.61599662	2.85817682
6.2723427	5.79291687	7.10067941	7.40547328
-2.9534214	3.60050843	3.73868892	-0.0551215
6.17242428	6.64084016	8.92603191	6.02536597
6.07655105	5.795753	7.72954839	6.66429106
5.4595723	4.69052595	-2.9534214	6.56041535
5.13284699	5.37994401	-2.9534214	6.19310701
3.10947132	7.25922719	7.21063764	6.09577895
3.23320271	0.52435582	4.43620166	4.21844721
7.06367571	7.32451032	8.3821682	7.79174469
5.41403087	6.58208995	6.98582981	7.49847372
7.85050459	6.89340483	7.94007016	4.01037301
5.21365521	6.11474086	6.54315314	7.51828464
12.9852049	12.9690442	11.7142289	13.2020034
5.34607072	6.80114986	-2.9534214	6.84439361
7.25438229	8.67376159	5.7637247	8.23139482
7.1386322	5.86355176	8.14074186	7.11269694
5.02370583	4.66283225	-2.9534214	5.82592141

7.8181859	7.75804122	7.34900208	6.42831067
11.7534406	11.4556144	11.9187622	12.5255918
5.90395015	6.21878433	6.68686574	7.84785945
7.43969384	6.55973968	8.17817076	7.73848605
5.44192255	4.2231101	-2.9534214	6.51572096
6.19132699	5.52030247	6.67031564	7.56347886
4.61495753	3.73069946	2.05956205	2.1030479
4.47926303	4.08154148	-0.8115387	4.30946185
7.69030971	6.58291113	6.87645331	4.77234132
6.59067747	6.47734458	5.03616461	5.11594082
2.6983095	5.03581273	-2.9534214	3.15668909
4.12837033	4.21465761	4.32957174	-2.9534214
5.41201782	4.93907891	3.86768313	2.35883854
12.8444144	12.1860378	11.3655063	12.6395914
7.87372875	8.67395429	7.3728514	7.68849421
5.22287121	5.04775827	-2.9534214	6.32847616
6.99271473	7.34643719	8.81372363	7.28589109
-2.9534214	5.64349279	5.09625162	3.37258123
3.81764355	2.05858306	-2.9534214	4.11500746
5.0498342	5.40244653	7.3651596	6.01526189
4.18605283	5.99768864	-2.9534214	5.04818166
6.08289162	5.70211146	-2.9534214	7.94863755
5.41000196	2.42380945	-2.9534214	6.16748398
5.4240542	5.25567824	-2.9534214	6.6260704
6.06250292	7.85300429	3.69026036	7.11585261
5.83049013	6.21030638	7.85429867	6.56965082
7.18770062	5.90485565	8.32350947	6.99594919
5.35238292	6.04016437	-0.2116394	6.23143553
4.56510275	6.80185538	-1.5168928	5.79672409
4.92269506	4.61224686	-2.9534214	5.70959352
7.08341566	8.16575586	7.14327672	6.30516666
5.1738168	7.75985782	-2.9534214	7.17755836
-2.9534214	5.6132654	7.69417106	-2.9534214
-2.9534214	7.27150393	5.77150459	6.11009146
6.34588985	6.81519513	2.62096701	7.08880681
5.15948935	6.63055136	-2.9534214	6.56388559
8.4267484	9.63614344	9.6425721	9.82681718
3.76814055	5.84997165	6.71437058	-2.9534214
-2.9534214	5.35323962	4.53091381	5.76692366
5.90824103	5.72461028	8.05688237	6.11640728
3.92318008	2.99715498	-2.9534214	4.3529028
7.24197034	6.62338511	3.552558	7.88208587
5.92245248	6.08606647	7.04238897	7.37879455
4.58310644	4.35201983	6.91208254	5.45684237
5.80617032	2.76216784	-0.0938779	4.88019786
5.33336287	5.22233731	0.46223352	5.57161034

3.18686093	5.32406839	7.5093807	4.14620829
5.65776865	4.17599634	-2.9534214	6.90219476
5.07295187	5.97033501	-2.9534214	6.33796579
9.29078262	8.33420735	7.673559	8.66506895
8.62116294	7.72680382	6.59558981	8.31511516
-2.9534214	4.2231101	6.77851154	-2.9534214
4.17184697	5.88900702	-2.3036717	5.03490637
4.79257357	6.62018865	-2.9534214	6.10533638
5.88955434	5.50305272	3.29629464	4.7562533
5.67297869	7.07772083	7.29064334	2.42138932
4.6899778	5.53055419	-2.3036717	5.63236873
5.28138163	3.76583651	7.78248364	4.95258228
2.50031482	6.59517299	-2.3036717	3.41418912
4.24151744	5.63242993	6.11049505	6.94274271
8.5561132	7.80560061	9.17345173	8.70673752
7.34421673	8.07352984	3.95225768	9.08254778
3.23320271	4.02465423	3.6057484	-1.2103612
5.2479158	5.68079121	6.68892122	-2.9534214
3.04857689	4.1227816	-2.9534214	3.56033181
6.69866198	6.25633038	8.04288816	7.00788854
4.52840699	6.32763289	-2.9534214	5.7649149
5.43400837	6.20391507	-2.9534214	6.91402613
6.53349479	6.87407857	8.62238175	6.73782256
5.74671266	6.85992028	7.37540625	7.40997928
3.96771613	-2.9534214	4.27588543	3.81453072
-1.4465118	5.01162075	6.33447321	0.50294321
5.96013852	5.1287172	7.74049017	6.12269557
10.4242841	10.6815405	9.63125967	10.336313
5.86474689	6.63213903	-2.9534214	8.00290992
5.10570335	5.53734842	6.28776951	-1.4884482
4.81102372	4.28093025	-2.9534214	5.58305665
6.72646719	6.69273383	8.26512228	7.13152823
6.36266347	6.34806433	7.05120421	7.79371646
5.59527235	5.88767841	-2.9534214	7.09281607
7.51618296	0.52435582	-0.48063	5.79475643
4.22326514	4.6597221	-2.3036717	4.81179359
4.88545635	5.06196358	-2.9534214	5.88445248
0.84297312	2.95717935	3.08778101	5.82977011
6.06121901	6.27474322	7.62231662	6.9665355
6.76876929	6.65730707	2.68827083	7.76987549
3.01713789	7.08063082	7.05996591	5.98451799
8.84451436	8.23452129	7.63516561	8.09773064
5.31839417	6.55973968	-2.9534214	6.85859158
4.20012017	4.72664287	0.0149924	4.07016065
4.78637071	4.73257541	-2.9534214	5.64771194
3.02769386	6.14285642	-0.6366176	3.60593164

3.92318008	4.80755745	-0.8115387	4.09595784
9.11415201	7.99272276	9.07727765	6.22414331
9.06823434	8.61576185	9.2001783	9.84594706
6.34694393	5.32799214	3.26356862	5.27683544
4.77701612	6.61055656	-2.9534214	6.15065854
5.9860803	8.41528207	9.58026497	2.85817682
4.37977448	3.55436287	-2.9534214	4.95962491
6.05993395	7.88797743	3.3070404	7.46792906
6.65166372	6.64713545	8.48452214	6.79897968
5.32696672	5.23283936	7.07418944	6.33526083
7.72761221	7.51424542	8.28564092	8.51329634
2.27075861	0.90665033	3.76230692	3.66744291
11.9354569	11.715978	12.2884953	12.5324162
4.29055225	5.26184478	-2.9534214	5.12536604
6.06634781	6.8780984	9.97993411	5.03823667
6.94199657	7.17731994	8.23202556	7.65851176
4.75494994	7.16311344	7.45968394	6.79209191
5.22745723	5.37046337	-2.9534214	6.55228554
5.31193117	5.59872307	6.73640059	6.9003659
6.35010157	5.98407666	7.0702525	0.25072817
4.65295524	6.4030356	-2.9534214	5.99312389
4.96450137	7.0730526	7.20274722	7.20596903
4.81712171	4.59281052	4.86493027	-1.4884482
7.79210896	8.57568931	6.97828633	7.91642425
3.21484424	2.28537678	-2.9534214	3.65881453
4.61845352	6.59109727	-2.9534214	5.98968768
5.64067838	2.02029613	0.0149924	3.97605485
9.3218061	8.39391656	9.73685957	9.39623846
3.49140772	3.82811264	6.41366678	4.53366132
2.92978863	2.78518393	-2.9534214	3.42440627
6.71671466	7.3623077	7.6314298	8.23901239
5.70949735	2.86296609	-2.9534214	6.94451866
7.6600966	7.51079747	5.086929	8.23430149
4.55053626	6.24910588	-1.5168928	5.75482896
5.61640669	6.34516321	7.12441531	7.20300464
5.25915756	-0.3516332	-1.8572999	3.444626
4.44423231	6.19212361	-2.9534214	5.68430846
11.2776274	10.9482251	10.180613	10.9853365
4.67662472	7.48905903	0.21080887	6.16900388
-2.0115616	5.93346686	5.16579142	-2.9534214
9.96636151	9.40343464	10.7520069	9.97829417
4.96450137	7.3156454	6.7967342	7.87930316
6.0560719	5.15546067	6.55559748	7.25688345
5.60763831	6.71612695	-2.9534214	7.54193878
4.59734937	-2.4449602	5.29285694	4.1585021
-2.9534214	5.1882076	4.40152671	5.2397097

5.47700873	5.92183077	2.82912145	5.13473009
5.39784727	6.75962785	-2.9534214	7.20670918
-1.2298595	4.53966249	4.58489335	3.65881453
2.17903669	3.08324495	6.40993328	2.93164669
6.36474652	8.63766241	4.01913607	8.2182418
7.63313093	8.22166078	8.09922816	6.1916123
9.5323137	9.6979957	9.74280794	8.27085728
4.64954196	6.19104686	-1.8572999	5.94603003
6.11294438	6.64399124	7.55235093	1.62476777
7.47145456	8.32270072	6.18665954	7.93574913
4.67662472	6.68968754	-2.9534214	6.12739386
5.20205179	6.49140805	-2.9534214	6.7831876
4.51346329	4.28093025	-2.9534214	5.33756575
3.9731877	1.64585281	-2.9534214	4.46603105
6.36994105	6.41967836	7.51170504	1.30906412
7.87299719	7.53221321	7.37923006	6.28147436
10.0454323	10.1229862	9.62925412	9.42498666
4.59024548	4.97705564	-2.9534214	5.52726678
5.84697531	5.68996707	8.41637579	5.55543138
6.05478225	5.25155252	7.41319578	6.51452588
9.97414247	9.15456332	10.2168563	10.2249144
4.20012017	5.81125287	-2.9534214	5.24836237
4.96724585	3.66397101	7.4323393	4.86151971
8.72129139	8.18264763	8.9448605	9.33611888
1.5766539	5.09227014	5.43434596	-2.9534214
5.41201782	5.7260978	-2.9534214	6.94274271
5.55387852	7.93665039	8.12374771	7.17906769
4.81407594	4.2356967	-2.9534214	5.74263202
8.1811637	9.58582593	6.58348713	9.55476885
5.98337145	6.72656852	7.14252698	7.66121425
5.40191022	5.34164181	6.95456224	6.52880182
5.97929861	6.65574687	3.49733123	5.9793297
4.56147489	6.10448361	4.69534966	2.66489412
4.8292409	6.34128595	-2.9534214	6.28988085
6.25447865	4.85455793	6.85006055	6.92035691
6.72159916	6.18131982	3.86040625	6.30930757
5.26362984	5.42826341	-1.5168928	6.49647999
7.64817404	7.91170235	7.97712709	9.07972241
6.13385922	4.76187765	7.81154569	5.91918688
7.99299368	7.6956345	5.76177316	8.35405251
5.90966849	4.45965971	-2.9534214	7.6682171
6.38951139	9.23877844	-2.9534214	9.53324487
4.38799097	6.3168141	2.35709797	4.44611116
7.11716925	5.54242317	6.86922064	7.83642721
5.66792647	-2.4449602	-2.9534214	4.66009488
4.56510275	4.93136211	-2.9534214	5.54610414

4.23242016	4.13627043	-2.9534214	5.00798361
-2.9534214	5.66381571	7.6174686	-2.9534214
5.16905678	4.72366742	-2.3036717	6.41428158
-2.9534214	5.84450361	5.14198196	1.69396844
4.28616277	4.91057988	-2.9534214	5.22517226
5.60587822	4.89479435	3.67374938	3.28558265
7.39605867	7.58440172	5.90658003	7.30537124
4.90276296	6.56224021	6.70017428	7.29287852
8.09030175	8.07031271	7.88375856	6.82620658
2.76291576	6.45060028	7.6174686	-2.2872455
5.12794961	4.57970594	-2.9534214	6.46217819
3.54382842	6.42609904	6.61841675	6.32847616
5.88810682	5.68232458	7.13726786	6.88102165
6.86462063	6.45778071	7.7175165	7.5057092
6.95449851	6.09530443	5.9258804	4.22430697
8.13248001	8.79139604	7.59407824	8.03576971
5.06528706	5.73794306	8.01038228	5.31302972
5.25017117	3.64522363	-1.0106329	5.54141781
8.83459139	7.92049956	8.72861006	9.38761533
5.99686513	5.48910286	6.66823347	7.268273
3.01713789	-0.5679016	-2.9534214	3.11944144
4.78637071	5.44100065	-2.9534214	6.09577895
5.19270139	6.07209766	7.21420999	6.54293787
7.91837582	6.86735385	8.87751856	7.58811443
5.44192255	5.73499085	7.1236557	-2.9534214
5.10819216	-2.9534214	-2.9534214	7.51530042
6.58175083	6.64635004	8.10655099	7.020572
4.95623646	4.82986618	-1.2416692	5.78289379
5.93372171	-2.4449602	-2.9534214	5.03823667
6.94060076	7.96306789	8.55157235	7.86624576
5.52250116	4.39753848	7.28725555	5.71168074
4.73897916	6.02694912	-2.9534214	6.22706462
8.38322772	8.11953101	8.73782261	9.12310182
7.08594319	5.41725555	7.50472075	7.16846907
4.17184697	4.93651123	-2.9534214	5.16246299
6.37924431	6.64320411	7.83725654	7.12135847
7.3421038	6.62418312	8.17157098	7.56463407
5.39173131	2.79655573	-1.0106329	5.36699839
6.01951864	4.81316706	6.36049754	7.08478638
6.17835803	7.51122891	8.41389254	7.00448737
2.90709921	6.59435877	-2.3036717	4.16460995
4.04249178	5.33190524	6.00390513	6.45472049
5.06784652	5.85813505	-2.9534214	6.64476122
6.03660504	5.99645648	1.3099641	6.9665355
-2.9534214	6.32174179	5.18921431	3.30782906
5.70621541	6.6957737	-2.9534214	8.07155917

5.14501818	6.86397971	7.62016396	6.54761927
4.01622887	4.83814381	5.82481986	6.14449121
3.68388926	5.30627827	6.29992419	5.75077476
4.66652817	3.89311802	5.65226549	6.03539977
3.43701025	3.86638749	4.33483211	-2.9534214
7.33042703	5.33969975	4.0704926	6.16291465
5.29673726	6.87608988	-2.9534214	7.33884113
4.72282959	5.99522327	-2.9534214	6.24302695
7.87263127	8.81062786	8.64161171	6.75208959
5.0524212	2.16770059	-2.9534214	5.97586045
7.35263765	7.16968767	7.24806176	8.84055708
9.22594357	9.47319093	9.19186799	8.38380869
5.26139543	8.06738182	7.18901582	8.20163007
3.57294953	4.41239771	6.07163779	5.16551178
6.074007	7.07129812	7.47762994	7.5582689
7.98184052	7.66726552	8.50683188	8.57220553
6.74257631	6.93965598	7.19191752	4.04389377
3.16789923	6.41967836	6.38479328	6.29962691
6.33530643	5.54073357	7.15075278	-2.9534214
5.81533836	6.36248307	7.86660898	6.44346114
6.88072254	7.46431666	5.69584487	6.63928901
8.24475212	7.92601115	8.60806076	8.92528651
-2.9534214	4.72961219	6.89793626	-2.9534214
5.33336287	8.08545814	0.21080887	7.83929378
3.75549476	6.96444443	-1.0106329	5.21637896
9.7767895	9.59358821	10.2759076	10.322124
10.7431482	10.9045532	9.88898984	10.7427709
3.04857689	7.18599337	7.70994528	-0.1734029
5.57742078	6.33545045	-1.8572999	7.57269479
4.87672456	6.8953894	-1.8572999	6.62717659
3.09949907	3.38390747	-2.9534214	3.89019908
3.77442211	5.07603037	5.74408943	6.24013781
9.20284187	9.42007281	8.86711787	8.64936586
5.9280981	7.45895906	4.43620166	6.30654829
5.49423693	4.37116077	-0.8115387	6.37794313
5.49803764	4.96450748	2.62096701	4.51943363
7.10787219	5.92442469	7.30612552	7.63835448
5.86032447	3.0739292	0.11622035	5.46181116
5.7451144	5.46969886	3.96588368	4.17069204
6.70771656	6.38431565	4.84294636	5.74058913
-2.9534214	6.5538882	4.97346585	6.35275309
3.79310436	3.76003944	5.57657375	5.35101871
7.01940524	6.51998806	8.63069275	6.77522626
8.76354598	8.45731058	9.8215018	8.74672749
6.75533534	7.85742257	8.79619244	7.31569984
5.63895817	6.41507459	7.6647089	6.53941682

5.53919767	3.86638749	8.02916255	4.76833611
5.29017628	4.80755745	-2.9534214	6.88102165
6.35430103	6.09530443	7.71398821	0.05419288
4.96175166	6.55556247	-1.8572999	6.66752048
5.79540012	5.42826341	-0.3398746	7.07265741
2.77549695	2.96727758	3.41031271	-2.9534214
7.36881462	8.45372366	8.35005531	8.89300148
-2.9534214	4.54641406	4.01913607	0.71755754
5.78143352	4.43440349	-2.9534214	7.67518613
8.33224538	9.49287605	7.78007534	8.82497501
4.65295524	6.59923723	0.21080887	5.90283794
5.51501801	5.19251823	6.48282703	-2.9534214
6.61363153	7.02370693	7.96784591	7.42150193
4.13326627	7.39587272	0.91975246	5.54141781
7.09600925	7.5090704	4.11397716	8.37032916
7.97025848	7.71898101	5.75981897	8.44122638
-2.9534214	4.62186772	6.79959054	-2.9534214
-2.9534214	5.60034614	4.64541836	3.35131846
9.11291327	8.27268346	9.58040338	9.12427749
4.25055772	6.13503914	7.08515604	5.89917955
5.40596176	5.42276998	6.78717203	6.60825506
7.79905768	6.21772731	7.16485144	4.2872408
5.5299457	3.66397101	6.91208254	5.60343391
5.41000196	7.57741403	2.55036907	6.80192155
4.99171403	6.45509223	-2.9534214	6.7831876
6.03136921	6.7317611	6.81848961	7.93708777
4.5687215	6.28184079	-2.9534214	6.20944736
6.98189872	6.70032152	4.52171754	6.93561698
7.21739788	7.89857004	8.5342425	8.025319
7.34051706	5.41171997	4.30833634	6.07160465
6.73454423	6.65418497	7.27977429	7.89500133
3.14868499	4.56648123	-1.8572999	3.87537958
4.24604466	4.29703342	6.68274598	5.00798361
4.15749984	5.70059899	-1.5168928	5.25696345
4.41638568	4.35970671	-2.9534214	5.54141781
7.60565162	5.65759294	9.05134934	6.39492844
4.7064972	4.77055351	-2.9534214	6.02704312
4.29929134	5.46079184	-2.3036717	5.52726678
7.58971091	7.18491204	8.5720414	7.73335866
7.75962743	6.92742349	6.75809925	6.10533638
4.71305236	3.87713921	6.17497488	5.5577538
5.07549778	6.80256056	-2.9534214	7.07184517
7.6025658	7.54491171	8.56702115	8.03368562
7.22830712	9.25239148	9.09598698	8.72027744
4.71305236	4.57310874	5.60732861	6.60713426
8.1615275	8.65181938	8.79690732	9.31379256

4.30797781	4.30103128	-2.9534214	5.39843638
3.41305416	7.52154508	6.90767671	6.60151717
5.23658577	5.19251823	3.44963694	3.72643846
6.29655009	6.15284537	7.07104075	7.42849851
6.35220283	5.97409573	8.34647885	-2.9534214
5.07295187	5.21388028	-2.9534214	6.73679806
9.9581935	10.0207883	10.4350739	10.7163775
7.21220141	6.82216682	7.95722612	7.75071795
3.00650411	2.64123462	-2.9534214	3.84527593
4.50593299	6.13839459	-2.9534214	6.16291465
5.33336287	6.54969399	1.81047971	6.22998003
-2.9534214	4.50888322	5.15987579	-2.2872455
4.79257357	7.43732747	-2.3036717	7.03231013
4.63925341	7.31168789	7.09526521	7.07346919
5.60587822	5.04775827	-2.9534214	7.57384266
5.50182837	6.75380573	-2.9534214	7.93351531
7.33733835	8.07323767	8.38057942	8.42573447
4.36319976	5.01649171	-2.9534214	5.59214876
7.12702052	7.84823095	8.27610186	8.13980498
5.27696412	6.59191334	8.08681032	5.81625448
5.55570316	5.46614264	7.4193989	6.09417987
6.58264598	7.16475981	5.10551439	6.42322495
3.60149444	6.00260679	-2.3036717	4.8727556
5.88084739	5.62447563	6.81284584	7.02812889
9.88152286	9.95126369	10.3789671	10.6186815
6.22611556	8.34247445	4.87579787	7.53900313
6.50827664	6.55807025	7.47644046	2.64755465
5.29455358	6.03297106	6.8071799	6.90128062
5.69962904	7.28418206	8.35976445	6.37399488
4.77701612	3.56769861	-1.2416692	5.25696345
8.15009937	8.4966249	7.69825837	7.7343856
0.26210614	5.65603304	5.80221128	3.61488117
5.27253304	4.29302445	-2.9534214	6.94096458
5.64239655	6.19319955	2.37763262	6.13984836
6.02084019	5.17083512	6.64935741	-2.2872455
4.30364111	4.26054518	5.88880732	5.66504952
4.41638568	2.77372178	-2.9534214	5.40876529
4.57233121	5.10146859	6.37333576	-2.9534214
9.45254157	8.96174792	9.50365947	7.70010464
4.72931113	4.88949389	-2.9534214	6.22706462
4.82319403	6.19963841	-2.9534214	6.61161224
5.7876576	3.58747391	7.66261861	5.09050164
5.64582675	7.42226535	1.3099641	7.69694741
-2.9534214	4.98701618	6.98499359	-2.9534214
7.12271887	7.45940629	8.32747314	7.75730051
7.37915593	5.80985065	6.88454695	8.04449018

6.34166579	7.92439228	3.9315743	7.91913633
-2.9534214	5.68385632	7.50996214	-2.9534214
6.03791104	7.48949702	2.53216626	7.84024803
6.5637301	6.54211354	5.78309633	4.90594756
6.86756165	7.79784107	5.4801682	7.36228154
3.63639923	5.50131634	6.85281333	-2.9534214
7.2521335	5.90090978	8.31288614	6.79799772
7.43919971	7.09163556	7.47941232	8.53762014
4.82016107	6.43885532	2.70461738	5.22809149
5.80002577	5.19681602	6.5544706	6.89026555
5.21134198	3.61984204	6.77173949	5.32398617
7.76830847	8.33396348	9.2839833	8.11911777
2.37364181	7.89162742	-2.9534214	4.4105755
9.23237482	9.22002973	10.6440456	9.062653
4.81407594	5.94117244	5.33546786	2.35883854
5.86768765	-2.9534214	-1.5168928	5.79081305
9.21544929	8.67914739	9.70293517	9.41765337
-2.0115616	3.96543144	3.33880563	3.60593164
4.65295524	3.61984204	6.69199895	4.86902002
6.00490117	-2.9534214	-1.5168928	5.87889084
6.59334473	5.33580776	7.18538046	-1.8332537
6.1712346	6.43522216	2.58609983	7.03731166
4.17659782	5.62607	6.02195428	6.5981364
5.22974479	4.05337823	3.04989883	2.78076375
6.36578693	6.95305651	4.4984671	6.45472049
3.52904442	5.41171997	5.80789673	-0.9773123
3.9731877	5.88235169	-2.9534214	5.43679432
7.64517794	9.47827538	5.77344303	9.57253518
6.61800421	8.45753446	4.89729051	8.14676388
5.25915756	5.74529726	-0.3398746	6.64038511
7.54264519	4.52264403	6.49697401	-2.9534214
4.726074	2.25272292	-2.9534214	5.86582969
5.14744014	5.76279588	7.08515604	6.25452598
4.81407594	4.69660858	-1.5168928	5.95486789
7.26668837	3.30647725	8.02184283	5.77293319
6.7867478	5.99027985	9.15256486	5.69067139
8.06969904	8.44629735	8.86325508	8.90946163
6.83937691	7.08353495	6.05738896	5.86770282
4.06828513	7.18274694	5.81732297	2.91724899
6.84236979	4.31691287	4.04504284	4.12756888
5.2479158	5.18604745	0.6083965	5.95486789
6.33636827	6.1033394	5.35631048	4.44108792
6.07145845	8.00073371	7.83910867	7.8359489
-2.9534214	4.19330716	3.91059006	-0.9773123
4.64269108	5.06431759	-0.6366176	5.53906892
5.07295187	5.77865194	-2.9534214	6.94451866

-2.9534214	4.20188529	3.28546824	2.79658224
5.7338768	5.46969886	7.40258886	6.18711887
3.08945741	-0.5679016	6.57573222	2.22496144
8.513094	8.86277997	7.90910665	8.37296922
4.4715516	6.70410045	-0.2116394	6.03706531
5.61115206	6.71762324	8.49953116	5.65423797
2.68503367	5.98904134	0.86262618	3.25155631
6.72646719	8.32319224	3.78554449	8.88562513
3.67047944	3.71280473	-2.9534214	4.78031856
7.8571783	7.05777569	8.51583377	7.85826001
6.67786957	5.60034614	7.88779062	-2.2872455
7.32562279	7.02793441	7.8783649	7.99092912
4.63235338	5.5905802	6.39867446	-0.3022547
7.31327212	9.11508623	5.53604327	9.13345398
5.79075959	5.0334117	6.45893601	6.80387947
5.27475028	7.07012727	2.47613788	6.4155626
6.26232141	6.58291113	5.39207584	5.05805857
-2.9534214	5.30229493	4.02565651	5.32943335
5.35238292	5.06196358	1.616789	5.47415876
2.06067066	3.67633528	-2.9534214	3.11944144
4.35902616	4.2356967	-2.3036717	5.53906892
7.39961879	8.25913301	10.0300472	7.00107816
4.29492841	7.80806083	0.0149924	6.48310138
5.19971987	5.76134574	6.31331037	1.01665269
6.69204096	4.78204076	7.14851403	6.67716561
3.91751515	5.87432456	2.62096701	3.74286052
1.95402673	5.50997739	-2.3036717	3.09406445
6.39563675	6.83463158	8.06798081	6.86141449
3.88305033	-2.0697503	-2.9534214	3.62377553
8.22911584	7.26589006	7.87566048	6.04867054
8.44704338	8.51696205	7.8321509	8.01479204
6.34060784	5.95265419	6.52600872	7.72563324
4.0372773	4.72068582	-2.9534214	5.38280302
5.32268682	5.32210251	-2.9534214	7.35094376
3.94561995	7.29825055	-1.8572999	5.98451799
7.48683744	6.97511778	7.63782814	8.28564452
5.90108245	5.97784668	8.14186772	-1.2103612
7.49826791	6.42701395	7.46569078	8.03326844
7.26835836	6.67512919	6.91559755	4.94550509
6.68121656	3.78308925	6.99166982	6.27160448
7.56183771	9.13817577	7.05439644	8.38053259
4.46379874	5.36283374	6.78621231	-2.2872455
2.68503367	4.26464531	-2.9534214	3.86791235
6.78441544	6.5505338	7.72254199	7.30606212
8.85492538	8.11386045	8.09225628	7.72614955
4.63235338	6.63055136	-2.3036717	6.65130058

4.78637071	4.61866789	6.4792685	5.68643255
-2.9534214	6.61698509	8.01898622	-2.9534214
7.96717702	7.82793938	7.23756816	7.25688345
-2.9534214	6.54548755	4.72770227	5.9618994
4.96724585	6.71237943	2.29367983	5.88260098
8.00238981	6.92029383	8.11460164	4.85775489
6.94826108	6.76107971	4.38133172	7.31020046
7.30841032	2.11417321	6.62487293	6.2035268
4.12837033	6.75890136	6.45169087	6.94629243
6.02742983	5.40430599	6.82598046	6.86141449
11.0751477	11.4934193	10.7018695	10.9996076
4.49456332	6.22405786	-0.2116394	5.99655192
2.28842343	3.48576994	-2.3036717	3.20490233
4.5759319	7.95832932	-1.0106329	7.2243597
3.75549476	5.43918791	-2.9534214	5.19565039
6.74177511	5.85269792	7.38558064	7.11664045
-2.9534214	4.90007541	6.82036598	-2.9534214
4.67997459	2.83014301	3.33880563	-0.9773123
4.03204391	4.70869743	4.77491022	6.90766745
6.16527142	5.67155662	7.51692116	-0.7767262
6.89232116	5.35708493	3.6057484	6.33796579
4.3166123	4.51921611	-2.9534214	5.74670913
7.16457119	8.33030049	8.54562981	8.244429
4.12345771	5.19681602	-2.9534214	5.61686059
2.81259531	3.65774892	3.65704726	6.33796579
6.06250292	6.37959746	5.37430399	4.60725977
-2.9534214	4.38252461	3.44963694	1.43568281
6.35848831	5.99522327	7.46868484	6.84914181
-2.9534214	3.47165113	5.81355986	-2.9534214
5.68303035	6.800444	3.40031186	6.2616666
7.50725324	7.98342375	8.6663378	8.11715074
3.74273715	4.30103128	5.24615995	5.76290335
5.66961251	7.3560765	7.20202776	7.60954067
3.88885195	3.87177336	-2.9534214	5.14093919
4.00023853	5.13544968	-2.9534214	5.46181116
7.31381131	7.57370092	7.80160666	8.45375066
9.31603137	8.20815247	9.26602476	9.43481205
6.97373316	6.78196921	4.98689393	6.84058377
6.1240547	5.99768864	6.90944066	7.20226259
-1.2298595	6.76976036	4.58489335	7.37484863
3.74273715	5.21811499	6.00720364	5.69067139
4.75494994	6.26658874	-0.3398746	6.39752391
3.26923333	4.26464531	-1.2416692	4.06363863
5.25242302	3.87713921	6.75614008	5.35903067
4.65975767	7.00299541	2.13387043	5.83360858
6.43582104	6.13167586	6.77464569	2.90270615

-0.7256766	6.57302604	4.9768346	6.0084863
3.58729258	4.69660858	-2.9534214	4.90958885
8.50674127	6.76397907	8.29207812	8.34500465
8.12819463	7.68687672	8.38248575	8.68618635
5.92950606	5.66226253	6.88185411	6.86891534
-0.1488474	5.65603304	6.598873	-2.9534214
7.45101235	8.27217443	7.44151189	7.020572
3.87137663	3.61984204	-2.9534214	5.15940776
7.05402628	7.25150084	7.86751669	7.90415638
5.9806575	5.34745237	6.85281333	6.71198941
6.39970592	5.85405912	8.05209963	-0.4437536
7.24197034	7.20851611	8.02550733	7.85873098
3.58729258	6.67204546	-0.6366176	5.29086398
7.55774647	5.73499085	8.08290772	1.1208487
7.42728955	6.94477563	8.1770729	7.66175413
7.28824899	7.19138791	8.08797904	7.81910718
6.35115258	5.28423258	2.24979738	6.72857582
4.7517699	5.68691493	5.78501926	1.51431472
8.76196628	9.04634486	9.15516909	9.77386247
7.10725025	6.50186641	8.90208989	6.40270088
1.73962402	5.81824359	-1.8572999	3.00155569
3.55846245	-0.1635983	-2.9534214	4.33676534
5.2569162	5.15987012	-2.9534214	7.41447125
6.9074042	2.21931282	1.89837289	4.51465971
3.48376111	1.5946578	4.36085003	4.24751088
4.56510275	5.86490277	-2.9534214	6.57195048
6.40881981	5.83901477	7.50238512	6.7109464
6.20418041	0.72812905	8.55664656	3.79103405
-2.4069576	4.84363596	3.63162582	4.77633546
7.1841663	6.3148383	8.30151247	6.89853473
5.54655677	6.23663623	7.78776779	5.98968768
9.32635585	8.9824335	7.77862843	9.55215045
-2.9534214	5.68385632	4.4984671	1.8231434
5.99417647	5.11969139	2.29367983	6.10533638
7.83631343	8.06620779	7.33134628	7.3536195
7.13192104	8.72636413	7.21278211	7.40804986
6.69535527	6.90527152	5.43434596	6.19011605
5.4595723	6.27677467	6.98164386	6.84248995
5.10321023	4.98453248	6.5801687	-2.2872455
6.48165038	6.0111734	6.96562553	7.38469326
6.68038054	6.81729018	7.39505439	7.69061216
5.8350048	5.90616854	3.11249423	5.94957167
6.58622105	7.14821095	7.66888042	7.62623418
2.32311713	4.37495867	-2.9534214	3.6414014
6.0339895	3.28241395	-0.0938779	6.56504048
6.89376445	7.52282943	8.23273022	7.58981759

8.64514119	8.7183521	9.67520933	8.83625887
4.53951405	5.83350496	-2.9534214	6.57424648
6.66946797	6.20924313	7.47941232	7.15395956
-2.9534214	6.15284537	4.50314726	4.71938556
5.35657576	5.98158789	6.91910401	6.58680957
5.88810682	4.90796092	7.71095708	5.54141781
7.48204789	7.88265182	6.65672748	7.37023138
5.14259214	6.96633371	7.22416574	6.81460077
5.2569162	6.08606647	8.15345042	5.20753173
6.50639098	6.61618309	7.32011778	7.47961234
4.78325925	5.77865194	-0.6366176	6.43842864
4.89124832	4.83263067	6.17056861	6.11009146
5.76890422	5.65759294	7.17001115	6.41684249
9.0201464	8.83702561	9.57846441	9.38090907
5.17619094	5.04775827	6.08418635	6.59135098
5.75149681	6.30591357	7.50238512	6.48676241
7.91660201	7.53857643	8.4723418	8.24370796
10.0667819	10.2089439	10.1669448	9.20557809
6.7960397	6.42426747	7.84326728	7.03397923
-2.9534214	5.29228822	4.13824941	1.69396844
5.81838147	4.41239771	6.06532234	-2.2872455
7.01277876	7.25356525	7.61638904	4.94550509
5.80002577	4.46323192	7.41070704	5.48883773
3.51410735	2.09588014	-2.9534214	4.76029215
0.02980441	7.28013717	8.94572031	-0.0551215
4.05802306	5.45003031	7.28996642	-2.9534214
7.78318106	7.47231593	5.37174718	8.53231579
-2.9534214	4.35201983	4.63266077	-2.2872455
4.38388857	4.32478858	0.67627567	4.52893433
7.46272905	4.67828322	7.07576121	-2.9534214
3.75549476	4.61224686	-2.9534214	5.29086398
8.32182909	7.58029542	9.99475707	7.34019089
5.3958115	4.22731785	7.08046627	5.31851834
6.51766817	6.46046418	8.11536604	6.54995429
3.27810212	4.86809514	0.0149924	3.95506514
6.5924562	7.50820608	8.10000074	7.44552945
2.68503367	4.02465423	4.36599777	-1.4884482
5.50749585	7.03395225	3.46890427	6.37003579
8.54742799	9.06041663	7.13349957	9.39234003
9.61699957	8.79936688	9.51166536	9.97471379
8.30104012	8.28535128	9.04233404	8.69069047
5.54288191	8.15337064	7.86978347	7.33816577
4.08859285	5.60520443	-2.9534214	5.99140681
6.71834467	8.25887611	7.5602322	8.79452805
2.77549695	3.16448568	4.4214429	4.66871562
9.58876281	10.6331057	9.25912418	10.0592041

4.99977873	5.17954751	-2.9534214	7.20670918
6.40881981	8.4786468	4.12616433	8.61241905
5.14744014	5.26798508	7.12820737	5.65423797
2.06067066	5.84450361	5.15690885	6.00678743
7.07644203	7.83553914	8.35265081	7.83642721
1.02258002	5.57580621	7.14327672	1.94169689
6.26455438	5.54916186	6.13180651	7.61679826
6.56915999	6.24910588	7.15075278	3.04193075
-2.9534214	4.18899889	2.90181097	2.94590212
7.07834728	7.40053806	8.42965054	7.41190612
8.67692376	8.54546647	9.3147579	9.01295163
6.5763682	6.46046418	3.70658451	7.16466491
8.43445449	9.1663906	9.40988584	9.26434808
4.4715516	6.88277409	-2.9534214	6.85859158
3.97863859	4.99197078	6.4863768	5.03490637
6.52978647	9.14012982	4.73964987	8.90900693
8.42749595	8.41366751	8.95258033	9.00488914
7.7859041	7.96401374	7.21206764	7.3536195
4.72282959	3.61342625	3.41031271	1.47553441
1.93171825	4.88683634	-1.8572999	3.21670803
2.82475243	4.94675465	0.53716524	3.36198902
5.74191258	5.5339553	7.65370094	-1.4884482
4.44816688	7.264867	-2.9534214	7.05468174
-2.9534214	6.5538882	4.46527383	5.98451799
6.85798134	6.06153157	7.4530474	2.61223772
6.61887715	6.00014981	9.19873647	5.32125485
6.41787649	-2.9534214	-1.0106329	5.64553002
5.21134198	5.73203259	3.40031186	4.90958885
6.06634781	3.9348827	7.66418661	5.17764288
6.67619316	5.86084595	8.30386145	0.15580464
8.4570848	9.04902165	8.91663429	9.68144132
8.31914609	6.58701	8.85618444	7.50991321
4.57952363	5.65603304	-2.9534214	6.75208959
6.1326373	6.36343921	7.39316461	6.88287517
4.92269506	5.66070768	6.26176833	6.62385548
8.19293565	8.54736092	9.60265246	8.27403876
11.398489	11.1782202	11.0548265	10.7247985
4.82319403	4.80474445	-2.9534214	6.97611748
6.74177511	5.84038894	8.97612638	-2.9534214
9.20036543	8.02752227	7.39379481	8.89139112
4.13814566	4.52606382	5.98227905	-2.9534214
6.48165038	5.28018783	9.4611469	4.58466098
7.16815379	6.86735385	8.32879195	3.66744291
8.95734582	7.85877933	8.40797763	9.50674453
7.19649875	7.71374215	7.27293932	6.29684903
9.26173456	9.63752738	9.34895766	8.67071267

5.90681215	4.52606382	7.45123212	5.49126977
5.75149681	7.51252247	4.16212005	6.80192155
10.7192316	10.3733517	12.0297616	10.1464638
7.86529335	7.62961115	8.00214007	6.1916123
3.48376111	7.17296359	-2.9534214	5.78487768
10.6126692	10.9814568	10.7911022	9.99027245
6.84908115	7.1972987	7.0229667	5.37228586
3.78067645	3.11991784	1.07869645	2.90270615
5.18564861	3.30647725	2.18136476	3.38309624
10.7425476	10.8969379	11.3091618	11.2816578
9.37146976	9.70536732	8.77457955	9.42879798
-1.7015481	4.50195329	2.99779047	5.82012902
2.73741937	3.85010816	-2.9534214	4.24751088
6.66946797	6.75817452	7.45183747	7.50269883
-2.9534214	4.05337823	6.06215422	-2.9534214
5.32054209	5.56087936	2.41784535	5.69700638
2.94100092	7.13034707	5.62675891	7.39512068
3.33870244	4.51233575	4.32429212	0.25072817
4.98901575	6.56473641	3.18419106	5.60343391
3.78067645	6.39931099	-2.9534214	5.99312389
6.78597076	5.75553056	7.02865859	7.27110639
5.61815399	5.20537338	4.04504284	4.2760006
5.62164226	6.50620178	6.98415689	7.00193122
5.91820373	6.26556618	2.97101307	6.62385548
1.5766539	5.52885062	-1.5168928	2.94590212
6.87999452	5.58567233	6.58679794	7.60449472
7.38121532	6.97824203	7.43907156	5.28526892
3.38869355	4.29703342	0.29957556	3.82227867
-2.4069576	5.67309983	3.4784423	6.98736018
4.75494994	6.30491851	1.65093092	5.98968768
2.48507573	7.93985897	7.36258653	4.98400687
3.88885195	3.29047973	-2.9534214	5.42920388
5.25467136	6.57054415	2.43753906	6.35942481
3.49140772	5.87432456	-2.9534214	5.39324406
5.85440676	6.35384913	5.88343254	4.05050546
4.44028697	5.91793113	-2.3036717	6.64366843
-2.9534214	4.99197078	3.52520886	3.5132435
5.50560916	7.39306625	7.05598991	7.52956872
4.20012017	5.78722781	7.55460712	4.62065105
6.04051949	5.97033501	8.66398867	-0.7767262
8.31376507	7.17731994	7.09138545	7.19183363
-2.9534214	4.78775027	4.9114431	-2.2872455
3.21484424	5.2597922	-2.9534214	4.93839302
5.28138163	5.82797403	3.57050692	5.06133591
5.09319446	3.33014576	7.88330986	3.82998523
5.05758132	-0.8224059	-2.9534214	6.03872893

8.05170997	7.54785868	7.74197582	6.63269485
7.81361821	8.42241063	8.57036992	8.77694366
7.29317909	6.38713918	6.0494117	5.86957353
7.74969878	7.95294005	7.71095708	9.16711747
5.96151557	5.7394169	8.46875728	-1.4884482
5.64924882	7.26997504	3.91059006	6.65455918
2.78796937	6.65808654	-0.0938779	4.49540449
5.89244501	7.115668	3.32829474	7.2192339
8.32423953	6.49925892	7.46509122	5.77293319
-2.9534214	6.14619376	7.56471653	-2.9534214
7.61092628	9.40099212	6.88544345	8.95620479
4.44423231	4.99937092	-2.9534214	6.63379597
4.0372773	6.04016437	6.50048918	5.8936745
3.88885195	4.70266566	-1.8572999	5.43679432
5.68469884	7.53942274	7.51807774	7.23165083
6.47105412	6.29993292	5.03616461	5.71792434
7.57629111	0.28699106	8.34680435	4.72352881
7.0815171	7.28468688	5.96711496	6.92576126
7.65542448	7.10083804	7.52038813	5.80261102
5.33548864	5.14437774	7.29469822	-2.9534214
5.18329	7.05304237	3.65704726	5.99140681
3.23320271	2.40906789	5.49434115	3.474434
7.68114487	7.68649473	8.29882323	8.24587
5.76575482	-2.4449602	-2.9534214	5.57390688
7.56772688	6.97824203	5.75981897	7.42976698
-2.9534214	10.65064	6.96138045	4.43604712
9.46242336	9.55257182	9.81939239	10.1841568
3.98406897	5.19251823	5.645931	6.00338365
5.00513027	5.61647722	-2.9534214	7.61010025
5.43994807	5.52543744	7.64842939	5.43931561
4.34220995	5.68691493	-2.9534214	6.64585317
6.36786548	6.82077517	3.80083084	7.29427195
7.28605242	5.2597922	2.94372925	7.92274454
3.05890614	5.53055419	-1.5168928	4.62951001
7.05853738	7.49081021	8.25162695	7.57785301
2.99579136	4.05337823	4.99023151	4.88019786
2.88404723	6.5589052	5.30630855	7.12762526
5.06272305	6.09184712	6.76103304	6.44094709
4.77701612	6.71537822	-2.9534214	7.5812816
6.47202064	-2.9534214	-2.9534214	4.80789656
7.38481221	8.23765341	7.02459525	7.45116204
1.76508181	7.01946704	-1.8572999	3.9409004
6.29655009	5.85405912	4.26490419	5.74263202
7.53063487	7.75585827	8.70188692	5.25410211
4.07338892	-2.9534214	-2.9534214	6.3189237
5.80463664	3.18193433	4.17390887	7.08960955

6.60748743	6.9033005	6.87915624	4.84259657
7.20291677	6.64792043	6.58900094	5.58760987
8.16092827	8.39975378	7.71348346	7.88162246
9.23735716	9.60100497	8.68705256	9.31808007
9.06983127	9.40738043	9.03568683	8.59810482
8.50130754	7.13259219	6.97323533	7.56116564
3.74913006	5.2096331	-2.9534214	5.6736408
4.113582	4.60256142	-0.48063	5.21931602
6.49216938	6.72284805	8.11651186	6.5992642
6.40781	5.54579645	4.55815602	5.24548391
3.78690379	4.76477541	6.63023111	-2.9534214
3.08945741	4.20615531	-1.5168928	4.35824208
2.21642809	3.05511526	-1.5168928	3.10680874
3.30438598	5.93989104	6.11814247	5.55543138
8.06426174	7.85810111	8.1007729	8.92820639
9.89230304	9.05124852	10.241398	9.72445638
5.80770238	6.71687529	7.40008174	6.78616181
5.40798327	6.30790162	1.3099641	7.11585261
3.72338668	4.80755745	-2.9534214	5.62130853
3.80542613	6.15063158	-2.9534214	6.11955485
3.44490803	4.07688562	-1.0106329	4.51465971
5.88084739	4.92100841	7.665231	5.40102555
4.89413561	4.72068582	6.74729072	-2.9534214
3.13898104	4.21041272	-1.2416692	4.33676534
3.1583241	6.25117372	-1.5168928	5.11908941
7.0897262	8.43701716	8.77336929	7.86624576
6.41285202	7.45133491	6.18956599	6.11640728
5.96426573	4.40869723	5.90834532	6.66321297
4.32519541	3.86638749	6.79291693	4.14620829
7.81247402	6.58701	7.25847959	5.58533506
2.32311713	4.03429258	4.54913232	4.72352881
-2.9534214	5.38936275	6.93823866	-2.9534214
6.78441544	7.6128232	6.60867796	6.4155626
6.41787649	4.72961219	7.11068076	-2.9534214
3.89463033	7.36755931	-2.9534214	6.63269485
3.87137663	3.71879433	-2.9534214	5.69278614
3.46057504	5.6761813	5.36918584	6.35141503
3.87722529	5.5889461	-2.9534214	6.08776577
4.7064972	5.89563178	7.25154274	5.39843638
5.86915578	6.42884204	7.98467663	5.97412269
5.2479158	5.60682024	3.67374938	4.82342191
9.13153655	8.02782384	9.40379802	8.83434443
3.72338668	5.96152169	-1.2416692	5.68855353
8.74147161	8.04043341	7.08359448	8.78737157
5.02370583	7.5679059	1.77995038	7.3977158
7.29645649	8.08226753	7.69825837	6.54528047

2.53031844	4.52606382	-0.0938779	3.41418912
6.5763682	3.57432054	2.08475918	5.94957167
6.93500395	6.52512414	8.86938528	2.07737831
4.55783788	5.58239112	-0.8115387	6.54645035
6.05219949	6.35384913	6.62701859	3.61488117
6.03136921	5.39123914	5.02318941	4.13380881
8.18794431	8.01419016	8.56001947	8.70149584
3.4527628	2.95717935	3.56156037	5.65423797
3.48376111	-2.4449602	-2.9534214	3.29674874
-1.7015481	6.3187872	4.1680265	5.94603003
7.6702381	7.22702014	9.87574244	6.41299941
7.18946455	6.64635004	7.1581903	4.98400687
6.32035798	7.90021813	7.69263533	7.84024803
2.17903669	7.10541736	4.97008921	7.70115551
5.61989918	-2.4449602	10.7004057	1.94169689
6.46135315	6.95432628	6.63023111	8.23466442
6.20998523	6.97948984	7.15447634	4.2760006
7.04431187	1.94053221	4.0704926	-2.2872455
5.99148279	7.61000612	5.50137575	6.42067536
6.09548977	6.05562791	7.14477504	6.74802767
9.25248304	9.03753596	8.23343453	9.24682316
5.77047634	5.07135666	7.22416574	5.73649465
6.3730488	5.97784668	6.66510456	7.35495551
-2.4069576	6.49925892	4.48906098	4.33676534
2.14065035	7.45223397	5.24336543	7.57613565
4.97817186	5.51858675	-0.8115387	7.09601548
7.23572409	4.35586839	7.01888728	-2.9534214
0.97973075	3.27430282	3.83092604	3.28558265
2.64445705	4.14963431	-2.9534214	4.45610549
-1.0415346	5.78722781	4.37112722	4.62065105
8.10812974	7.94561649	8.54279139	8.57220553
6.21461233	-0.1635983	7.82796007	3.79890887
5.50749585	6.26454289	6.86195153	6.73577284
3.33870244	4.6597221	-1.5168928	4.86151971
7.65201705	6.43885532	6.97828633	5.54610414
1.54760822	7.41766984	5.80410892	6.12739386
7.31758001	8.6927148	9.64535333	7.40353781
5.43400837	4.72366742	6.96477751	-2.9534214
4.54319748	5.19466873	5.60297499	6.40270088
6.04442334	5.98407666	4.48906098	5.50095713
5.93372171	8.46044183	3.1724863	8.77519833
6.1067348	6.33056944	6.11202778	4.29282823
6.83262015	7.65443238	8.04409299	7.66283331
6.80910161	6.94605271	7.2940232	7.81134214
7.60344813	7.32941184	6.21546377	7.53370387
2.32311713	5.16864877	-2.9534214	4.21256355

4.70977851	4.55982314	6.52830645	5.09050164
7.64731865	7.84891382	8.17267304	8.41264279
4.49075344	2.09588014	5.22081219	4.43604712
4.10861867	6.28386227	-2.3036717	6.6469443
0.26210614	3.97547228	7.60989469	-0.4437536
6.52792873	7.25046753	5.62675891	6.59021696
-2.9534214	3.56104615	5.45139763	-2.9534214
5.36908165	4.38252461	-1.2416692	7.44928695
3.9731877	4.04386697	-1.5168928	5.47169768
4.20012017	4.64091906	6.67135561	4.5241918
9.43008801	8.80799645	9.48100371	9.70533672
7.96099429	7.82759298	8.32648325	8.51718001
3.46834521	7.23696667	-2.9534214	6.19609178
6.61713073	4.56315603	7.12744976	-2.9534214
4.82016107	3.18193433	-2.3036717	6.84058377
3.36391451	2.13223724	3.38009974	-2.2872455
-2.9534214	11.5364717	7.35030136	8.77868689
3.04857689	2.90559992	-2.3036717	4.51943363
4.67326705	-2.9534214	-0.6366176	3.74286052
7.98217976	5.42093418	7.31679858	7.54076524
7.47676094	7.57864962	7.62875542	6.25022457
4.76444823	-0.6895489	-2.3036717	4.60276824
6.73936884	-2.9534214	-2.9534214	5.35101871
4.50970305	5.42643459	1.02763635	5.69067139
3.13898104	5.21599919	5.13295112	0.57808499
5.77518247	5.31816269	7.3352885	5.75077476
5.85440676	6.09530443	7.68183919	5.97586045
7.55683573	8.45977142	6.80623341	8.20829773
3.33870244	3.15568158	1.65093092	2.29345235
3.60854328	4.18899889	-2.9534214	5.62795482
5.98201511	5.40244653	7.55685979	-0.0551215
4.22326514	2.71500391	-2.3036717	5.79081305
5.77518247	6.75161636	5.95691628	8.19940066
1.10463976	5.67464139	3.94539611	6.67502781
7.53109866	7.61201888	8.1107736	5.8393472
2.65810979	1.83419519	3.71467784	3.5510363
6.73695855	7.32500122	6.81095967	8.55893451
6.57995885	7.38272891	6.28641265	6.25452598
9.99547326	10.4159101	10.6036226	10.6840545
-2.4069576	3.84464064	2.03391701	5.17461965
6.71426619	6.18348706	6.88185411	7.49786912
5.44192255	6.65262139	6.91208254	6.87171813
3.82975838	5.12421135	-2.9534214	6.15219626
5.87062243	6.52170212	4.93241502	5.5577538
2.06067066	3.12894223	4.12616433	3.74286052
6.84236979	6.54042358	7.62392902	7.15088624

2.42245677	4.04863044	-2.9534214	4.36886174
4.92269506	6.11474086	6.36949628	6.61272956
6.29217885	4.37495867	7.14552361	5.74263202
5.47508172	3.04561548	7.93184906	3.77515427
5.18800337	6.61136169	5.87983819	3.98988023
6.4555011	6.66120022	7.44638019	3.86040627
7.015433	8.0779053	8.52045802	5.49854138
5.6475388	7.43095667	5.67318183	5.73649465
7.74969878	6.00137882	7.23123516	7.82586748
4.09864051	4.21889004	-2.3036717	6.12269557
3.87137663	4.91840834	-2.9534214	6.20204286
7.02204733	7.44140818	7.02703463	6.2035268
8.11433333	8.69916696	8.51177536	9.27603174
8.26022075	8.9928206	7.86706291	8.56789143
4.18133307	3.18193433	-2.9534214	6.37531216
3.27810212	6.49315638	5.33808972	6.96128202
5.08310866	4.32871037	3.42024471	3.58786429
5.86768765	6.67512919	3.73072955	6.76017928
7.2739111	4.99197078	8.0144858	-0.4437536
6.97850202	6.03417244	7.45546422	7.020572
4.86205311	-2.4449602	-1.2416692	5.36169147
4.47926303	5.24534173	2.73676511	4.51943363
-1.0415346	3.42111796	2.47613788	3.36198902
6.99001832	7.71673811	8.06560975	7.77287725
-2.9534214	5.62128158	6.99333406	-2.9534214
6.82431868	8.13948342	6.18082904	7.59491505
8.6906893	8.7183521	9.24137682	9.09239323
8.28215308	7.3391652	8.73185361	7.9697203
7.44068159	8.02087162	7.1470196	7.29078584
6.4447103	8.28408949	8.11613002	7.72975861
9.42660239	10.0781892	9.71382754	9.08717748
3.11937512	4.24817444	-2.9534214	5.19862991
7.10538283	4.49149556	7.6589533	5.94957167
6.15206502	4.91580357	4.73567832	4.22430697
3.77442211	7.64384696	-2.9534214	7.01635656
5.65266279	5.11060876	8.18946676	-2.9534214
8.78800674	8.80834758	8.66920378	9.83975763
5.79694365	5.25567824	6.52600872	6.41299941
6.0904637	6.0111734	7.86660898	5.86582969
7.015433	5.87834371	6.31730212	7.94420626
6.76167278	6.42518355	5.30630855	6.30101384
8.60825366	7.61683809	8.04569788	9.13403773
5.68469884	5.33969975	7.44212132	5.50095713
5.55934554	5.18171741	6.39992979	6.28428197
6.60043341	5.28018783	5.66485165	4.05708699
7.18534537	7.08527463	6.05738896	6.97002722

6.82280417	6.72433739	7.43479103	7.40869328
7.8571783	7.33039015	6.81472955	7.33410699
3.53645535	6.51397277	-0.3398746	5.86207611
5.52250116	5.05250893	7.55854698	5.05805857
3.36391451	4.96450748	-2.9534214	5.66289369
7.4791665	5.15103771	8.17743894	6.17959858
8.94173241	10.0892126	8.45372518	9.80021503
7.38583825	6.66585817	7.24037392	5.39064087
3.88305033	6.85312909	-2.9534214	6.82620658
6.07909062	8.63608081	5.05541119	8.10925572
6.35115258	6.04851148	6.54428889	7.3918702
6.54180382	8.33591328	8.034426	7.92948568
4.66314689	4.07221469	6.26452747	-2.9534214
1.21959057	4.26464531	7.95722612	0.15580464
6.5325686	6.68127694	7.1604141	7.40869328
7.5421851	4.23986795	7.73652093	6.04536428
3.46834521	5.16645911	-2.9534214	5.83552399
2.63067388	3.94001929	-2.9534214	4.72352881
6.31282528	-2.9534214	8.61995988	2.97399725
7.56229158	8.1872449	7.95978201	8.79625017
5.68803004	4.46323192	5.99396413	6.19460016
6.78907639	5.53565286	6.98666555	6.91583776
-2.9534214	3.25794247	4.98689393	-2.9534214
-2.9534214	4.92878064	6.41986789	-2.9534214
5.84548442	4.77343391	2.90181097	5.73238853
6.20998523	6.19534903	6.66301485	7.25973928
4.87672456	5.95519333	5.76761989	6.99680529
4.65636047	4.92360381	1.71688244	5.27965208
9.27189867	8.50097603	9.22517996	9.43433817
4.68331671	4.72961219	-1.8572999	7.17377811
6.5371936	-2.9534214	8.59934479	3.14437975
1.60512633	6.68663481	-2.9534214	4.1585021
3.51410735	5.45542111	-2.9534214	6.06182035
5.8823022	7.20745154	6.0526079	5.51297557
4.71957787	4.71170388	0.29957556	6.14757817
7.72882569	6.58373183	7.28861162	8.16019974
5.91678469	4.33262153	6.65357349	-1.4884482
9.94014675	9.67892018	10.5131338	9.96150921
3.82371368	-2.0697503	1.89837289	2.91724899
7.55592441	7.531788	7.83122065	6.1916123
4.56147489	4.25643338	6.27687871	4.7481415
8.97654279	8.40115121	9.26671301	9.01675504
5.17143875	5.27002605	6.81567049	5.58305665
2.94100092	3.24139446	8.83267208	0.50294321
8.42849208	9.06247881	9.12853428	7.6106596
-2.9534214	5.4373729	2.97101307	6.0437083

5.85143877	4.10001611	4.3965044	3.08120658
4.27291364	5.13992062	-2.9534214	7.1268434
-2.9534214	3.11083664	4.80175128	-2.9534214
5.47893316	6.95749583	7.05280121	6.87730744
6.66524875	7.90449438	8.16641686	7.4869428
4.1907572	6.31186952	-2.9534214	7.21996728
3.47607375	-2.4449602	-2.9534214	3.59692625
3.55846245	5.84724022	-1.5168928	5.9810612
5.59171967	5.91271515	7.39757026	1.65978297
8.09061643	7.38461394	8.70315863	7.80841944
6.56825643	8.06473891	8.630158	7.03731166
7.64045717	5.94245272	8.14186772	6.82716953
8.75006307	8.82700969	8.81537101	8.01943337
7.8857462	6.42884204	7.62714841	7.84453439
3.94561995	6.4861503	-1.8572999	6.78417968
8.82478886	8.03173859	7.9888538	8.08087421
5.66116254	-2.9534214	-2.3036717	5.41646374
5.73226426	4.21889004	4.45564805	7.35161316
2.46967395	4.88150651	-0.8115387	4.12756888
7.3457994	7.25562671	6.37588975	7.1205732
5.8350048	6.08722445	7.64048576	5.86957353
8.20896683	7.93150177	8.48511368	8.59838686
6.5509803	6.87876729	8.45794998	6.24302695
3.21484424	3.81698783	4.68714687	4.7276602
0.63778364	4.10916546	7.86388242	-0.4437536
6.87780826	8.05943852	7.65527866	8.34433217
5.01046202	5.07603037	6.92521988	5.1532778
5.37735909	5.54073357	6.72942746	5.99655192
5.06272305	5.93860849	2.58609983	6.11325282
5.60235159	6.98198222	5.12386339	5.77692569
5.50938007	4.97455473	5.7201688	6.6370943
6.67535423	6.94349742	7.2326449	7.61345314
5.05758132	5.99891975	6.51446486	6.31755388
1.1439832	5.31816269	6.05420334	2.55758639
5.73709649	5.12195314	5.93802937	2.53889933
10.257945	9.70649808	9.67572765	9.63463118
5.31408872	7.11055179	6.92957254	6.98131731
7.68739993	7.70810788	8.11000678	6.31068523
2.21642809	4.31295883	-1.8572999	4.12756888
6.88145019	1.71939224	4.23707977	-2.9534214
2.71146428	1.56836252	4.08928955	2.87317316
4.99709551	6.4622504	5.09315077	4.56632356
-0.4667031	4.97204947	3.32829474	-0.9773123
2.03996322	4.17599634	-1.8572999	3.91214675
6.28998825	6.83463158	7.4704783	6.96215893
4.38388857	6.06270941	-1.5168928	7.21703154

7.31542767	8.06532664	10.2747106	6.20204286
8.89673406	8.53744018	9.86521529	8.48703298
4.59024548	3.72475918	5.91186943	-2.9534214
2.95212674	4.7056847	-2.9534214	5.32398617
7.47627935	8.08545814	8.99078004	7.53370387
5.23202872	-2.9534214	-2.9534214	3.69302268
5.17619094	5.17519789	6.99416546	5.29086398
4.65975767	6.48263446	7.09526521	5.70959352
5.41604111	6.35481101	1.616789	7.544283
7.85347449	8.27013649	6.86740679	8.41456608
3.35555935	4.51233575	5.96202463	4.1023357
10.6278719	10.9015942	11.0058907	11.2872231
4.85317808	-2.9534214	-2.3036717	4.59374302
4.2595417	7.68190304	-2.3036717	8.14946108
7.00144342	6.04851148	8.05568817	6.38319071
5.65776865	6.31285979	3.26356862	6.68356005
6.53626979	-2.9534214	-2.9534214	5.29643742
6.88435714	5.24741496	6.84361689	6.83580728
9.98128783	9.67517089	10.4424028	10.0283458
-2.9534214	4.35970671	2.03391701	5.5577538
6.54180382	-2.9534214	-2.9534214	5.31302972
5.96151557	6.34225624	6.45652501	7.33207331
-2.0115616	3.26614584	1.26658167	4.8949682
0.79434634	5.7540731	-2.9534214	3.14437975
8.44482978	7.28569598	6.24510176	8.68777765
9.24727052	10.1978639	10.0601342	10.1896894
3.73631579	4.26054518	-1.5168928	5.72827068
7.31111335	6.26044248	8.11000678	6.7561401
6.66440342	6.79973779	7.18465229	7.51290858
4.61145305	4.33262153	6.52715804	4.54306909
5.37942105	6.05562791	6.65252062	6.42195072
5.70949735	5.97409573	6.53060052	6.75107518
5.37322631	5.08068899	6.27551156	6.02871833
-2.9534214	5.72461028	6.94169059	-2.9534214
8.66935956	8.80007327	8.98368114	9.32389071
5.83049013	5.84587257	5.88163648	3.96209569
6.27012176	6.25220653	6.92085405	7.01804421
4.71305236	5.22654731	6.47808037	5.34833813
5.08563673	4.82432125	2.87317313	4.96663333
7.10662805	7.52454011	6.72141669	6.87730744
5.99013406	6.56805798	5.68764489	5.31302972
5.66792647	4.26873381	7.05678599	-2.9534214
4.48310334	3.41375224	5.6416925	-2.9534214
5.78454891	6.46937322	7.24457237	6.45845416
4.70320842	-1.3152731	-2.9534214	6.06835058
-2.9534214	3.84464064	1.5459812	4.93482379

-1.2298595	5.63084257	3.78554449	4.39507384
8.52245432	8.44765044	7.65264817	8.60232965
7.38121532	7.17568785	7.95893055	7.65200507
5.59881631	-2.9534214	8.23695093	2.33737074
6.07909062	4.91840834	6.7967342	5.98796651
2.76291576	6.70032152	9.27374867	-1.4884482
10.5491169	10.3906315	9.92529567	10.4229416
-2.9534214	4.6996403	2.22734526	5.66073462
7.18180526	9.01990674	7.1091466	8.24623003
7.55090184	7.77106997	7.02784684	7.40030626
3.03817316	5.78580204	6.89615818	3.76714835
5.96838116	3.44299212	6.06215422	-2.2872455
5.11067669	6.34709793	6.89348695	6.14139764
9.7939928	8.4782055	9.36318624	9.72678025
6.82885267	5.66226253	6.89615818	7.00107816
3.65694381	4.18034356	-2.9534214	6.33661395
5.85440676	4.88417389	7.0663048	5.52251876
5.97929861	4.28497292	7.24736456	-2.9534214
8.1686989	7.81051686	9.15776862	7.75931989
5.68135993	8.66951574	4.94278775	7.926793
9.60724543	10.1561942	10.0241573	10.5135123
5.19504468	6.46046418	4.02565651	5.78685885
6.86976349	8.30986212	7.19264203	9.03228676
9.01799723	7.81576574	8.49160466	9.18563149
7.43027632	7.48598934	7.28045601	8.61437271
5.50938007	7.06895548	8.43333343	5.342962
-2.9534214	6.37011454	7.41195194	-2.9534214
5.78920943	5.27206414	4.27588543	5.0743713
5.43994807	5.28018783	7.24107451	5.25696345
-1.7015481	5.13544968	2.60363875	6.21975022
5.27696412	5.15325089	3.96588368	4.60276824
5.56116329	4.92619455	7.18610827	5.17461965
8.0481499	8.29865883	8.57898511	8.62465548
6.38335991	7.20371932	8.58396397	6.12739386
3.78067645	6.64635004	7.4530474	4.51465971
7.24423501	5.63401554	4.87579787	6.68143172
7.21046509	6.61618309	8.65218529	6.37662825
7.90841431	6.76976036	7.34248806	6.28568373
0.26210614	5.83074215	4.02565651	5.42412132
5.87208758	7.49474261	7.69621616	6.82138217
8.66577374	9.13621908	7.99468161	9.1414116
7.7998277	8.05382246	7.59517462	7.47470459
5.1007128	4.6090256	0.91975246	6.50733459
4.9727192	4.687475	1.47151796	5.96365194
-2.9534214	4.54641406	2.00780786	5.61908627
5.58100881	5.60682024	6.67964842	6.14449121

7.76041877	6.57962362	7.65159463	7.75325326
4.7064972	-2.4449602	1.81047971	3.2284179
3.87722529	6.79903123	-2.9534214	7.38338452
5.74351438	8.00318969	7.38050241	7.51649485
5.2658608	5.47678509	4.60244686	7.8639016
0.63778364	4.12729191	2.00780786	6.68249627
5.80617032	-2.9534214	-1.5168928	4.64706639
8.62941665	8.21796519	8.7163184	8.95444318
7.61618173	5.96278403	7.10530403	7.61512667
2.90709921	1.10261487	-2.9534214	5.08084509
7.90519773	9.11636297	7.46509122	8.82762383
-2.9534214	6.10676931	3.01099506	6.55577536
3.12921138	6.54464479	-0.48063	5.85264921
5.71603893	5.15103771	9.11235241	-2.9534214
4.89124832	4.35586839	5.2901515	5.80847402
5.97248489	6.6957737	8.58699817	5.31851834
7.18946455	6.8700475	8.35394681	4.42081818
3.01713789	4.83263067	-2.3036717	5.54376288
11.5209315	11.6100942	11.3017935	11.3103485
6.30308221	6.49402975	7.60772339	6.47575133
5.38353615	5.92701396	7.6647089	5.19565039
8.32370423	8.40997028	7.83586588	8.21603792
5.2479158	-2.4449602	5.05859413	3.00155569
3.20557665	5.83074215	-2.9534214	6.26735377
4.63925341	5.69908493	6.25623417	5.80847402
5.47893316	-2.9534214	-2.3036717	5.00798361
3.09949907	3.0645529	-2.9534214	5.60343391
5.96151557	1.83419519	3.23008299	6.38449963
6.44372531	6.12040798	4.73567832	6.39492844
4.47926303	6.82147116	6.53746094	6.34066569
5.66792647	6.24599856	5.58321949	4.76833611
6.38848796	4.03908772	7.2262902	-2.9534214
6.71344911	9.17322261	6.86649901	8.14444797
9.33727011	8.42103365	8.86530138	8.22483329
7.08657438	6.19534903	7.48947111	4.28163165
1.39290183	4.41977033	-1.8572999	3.57874506
5.67129658	-2.9534214	3.08778101	3.444626
8.18352579	7.44050235	8.43975594	8.07642669
3.98947899	5.43918791	-2.9534214	7.29566404
9.2265164	9.12752167	8.42040199	9.34000336
8.16541643	8.62256668	8.39764636	7.72614955
6.48644125	4.34816098	5.69379924	6.74497371
8.82194879	8.22981033	8.98765248	8.86537793
7.50536624	8.39180936	6.82504623	8.2961148
7.30461757	8.04699469	7.51634252	7.0197299
4.18133307	6.44519141	1.86966605	6.15526679

7.9770827	7.03094647	8.15976296	5.83552399
2.34015605	4.25230982	-2.9534214	4.98054886
7.7843487	7.76565561	9.68939555	6.81460077
2.61675775	5.33775507	4.38133172	5.87703219
-2.9534214	4.34429178	1.5459812	6.32711539
3.08945741	2.31730788	-2.9534214	5.58305665
7.11531463	6.8927427	7.81201727	7.25473786
7.14167243	7.57163394	7.70233413	6.09737626
5.15708754	-2.9534214	-2.9534214	6.67288684
-2.0115616	4.58299326	1.5459812	6.98822139
-0.4667031	4.08618237	4.04504284	1.65978297
2.87238163	5.03820976	-2.3036717	5.5577538
7.13924076	7.95389256	7.69263533	8.31133505
8.24983286	8.83943286	7.91658118	8.53408608
8.05009285	7.77466828	8.91202241	6.05526043
6.19484384	5.14437774	6.53974052	2.59424966
3.55846245	7.04650859	-2.9534214	7.21776604
2.71146428	3.64522363	-2.3036717	5.04155929
7.14167243	7.46965443	8.16899622	7.39901161
7.325088	7.58152854	7.60446031	8.22556383
10.4642355	9.42076192	10.7804185	9.88785308
4.99440728	4.93651123	8.39764636	-2.2872455
4.49075344	5.43008992	-0.2116394	7.007039
-2.9534214	5.97284325	4.41648957	-2.9534214
8.15070311	9.16994728	7.67096166	8.99292484
5.60058504	7.70018276	6.63982596	7.86343231
7.79172194	7.04650859	8.33241247	7.46112156
8.36200808	10.3954307	8.04730098	9.97764385
7.02928823	7.16475981	5.56318971	7.63230706
6.80143246	7.0005391	7.08671592	5.62352736
8.84208674	9.40122492	9.29768589	9.68330377
6.6845558	-2.9534214	-2.9534214	5.69911188
7.86124153	5.63242993	6.27687871	6.04039063
4.26847008	1.10261487	1.43279367	5.93535263
5.77674777	4.72366742	6.93737439	5.32398617
6.88072254	6.49315638	7.48415461	7.0188873
7.79790188	7.14710092	5.72818649	8.40040203
3.58729258	6.00383371	5.31698025	6.15065854
5.85143877	4.82432125	6.71840114	5.65423797
7.31488909	5.71265459	8.2263759	6.11009146
7.49684405	6.55556247	7.91701966	7.18884003
3.26923333	5.79291687	4.65806413	6.48676241
2.78796937	4.61866789	-2.9534214	5.65206591
-2.9534214	6.19534903	2.79898818	7.06777712
5.62685889	3.96543144	6.72542764	-1.4884482
3.23320271	6.05681058	-1.2416692	6.1826114

6.5537219	7.43914254	6.92521988	6.06835058
6.82128808	7.63872244	6.79004734	6.75310329
5.81990062	5.89033441	4.78263019	5.45185642
6.16407582	6.07909897	7.33921999	3.26298783
-0.8749787	3.92455433	2.41784535	3.49396925
0.26210614	6.1660573	3.43010881	6.82524299
3.27810212	-2.9534214	-2.9534214	4.95962491
4.70977851	2.46715134	5.52687975	4.03725163
10.2134155	10.0336845	10.3507636	10.5863433
4.57233121	6.14619376	6.22257586	6.08615778
6.4506062	6.12830473	7.36965147	6.47329296
5.61640669	6.91443415	4.77491022	6.31755388
3.40497957	5.88634857	5.38955037	5.71999956
7.70436001	7.56208752	7.72103618	6.74089168
9.01501617	7.69753135	8.58865051	8.90148362
4.85910083	5.83901477	6.3936422	5.86207611
5.2456569	7.76420835	6.64194951	7.6022464
7.3904464	7.84891382	6.57684262	7.86155362
3.96771613	3.81698783	-2.9534214	7.268273
10.1284617	10.519586	9.96156064	11.3863455
4.62889095	5.07836156	0.46223352	6.74599242
5.70785731	5.94373185	7.41257399	5.64989057
5.10570335	2.46715134	5.81920085	4.14002187
8.22367459	9.46908823	9.72951427	8.67873702
2.78796937	3.33014576	-2.9534214	5.56007248
5.41403087	4.26464531	7.43295262	-2.2872455
-2.9534214	3.25794247	0.53716524	5.40102555
5.79540012	4.40126766	6.51330538	5.43679432
6.90311083	6.41599652	6.42480965	7.96053448
4.17659782	-2.9534214	-2.9534214	5.79081305
8.07479784	7.47364484	7.00987172	7.82827421
5.57021784	2.13223724	1.95412777	3.88280836
6.47298651	5.83350496	7.81954171	5.81236949
12.3543861	12.8649179	12.0494432	12.7327514
5.91678469	5.57250246	6.76882742	6.1583308
6.64482294	7.71823377	7.1236557	8.22117507
7.15135835	6.60571629	8.52621757	6.31481035
6.88508296	7.76818484	7.26124502	6.56965082
9.2952946	8.61013367	9.68425305	8.96585539
5.35657576	-2.4449602	-2.9534214	5.94425594
5.60058504	5.28018783	2.39787909	6.76118731
9.13168946	9.21632995	9.4817449	9.56879789
6.01289269	5.85405912	7.00410508	6.22998003
8.34680836	7.1042739	6.72342355	8.03660251
-2.9534214	5.40244653	3.85309248	-2.9534214
7.57898508	7.12528274	7.66261861	7.93619548

7.81247402	8.08893077	6.91910401	8.30616435
-2.9534214	4.50542242	5.74606502	-2.9534214
5.08057616	-2.9534214	-2.9534214	6.52287067
4.87964103	4.90796092	6.69711396	4.7842907
7.09538218	7.36612894	9.07727765	6.3189237
5.11563295	6.44066847	5.09934582	5.02486912
3.10947132	6.9137816	4.50781229	3.86791235
3.87722529	6.28082899	7.69058514	3.86791235
8.32664595	8.35934724	8.68319113	8.74545464
4.94235552	3.76583651	6.92783305	-2.9534214
6.06634781	6.9033005	5.20940196	6.52761754
6.5445629	6.90658403	6.1557831	8.35271566
6.03791104	6.3168141	7.97502297	5.61016287
9.04731175	9.28214839	9.3401565	9.68396835
3.10947132	5.88368521	5.22648364	2.15305622
3.38869355	7.51209142	-2.9534214	7.50269883
7.59593102	7.93343466	7.92358084	6.89853473
7.08783593	7.70169567	6.84546087	8.84745516
3.09949907	5.21175825	-0.2116394	5.32671233
2.08108509	5.85677769	2.20453818	3.38309624
4.15268559	4.37116077	5.00680457	5.30198942
7.3750283	7.05541097	7.49300459	7.8573176
7.03060086	7.33721979	6.22257586	7.25616861
1.1439832	5.02618456	3.71467784	4.98400687
8.10999362	5.64977654	7.80776746	4.53837287
5.50182837	2.97730562	5.978923	-2.9534214
10.2814976	10.1060197	10.1582548	10.887524
5.09570493	-2.9534214	3.24133144	2.63000425
7.33786862	6.8585646	7.62929069	5.58760987
10.986701	11.4358652	11.4809565	11.5642169
1.51796573	5.26389445	-2.9534214	4.48567964
2.58851621	4.8408925	-2.9534214	5.68855353
5.13528945	4.3050181	7.59517462	-2.2872455
4.19544628	6.30093142	6.95028444	4.97360788
1.02258002	5.95899367	4.25383874	5.23100482
5.19270139	4.88949389	6.461343	5.23100482
6.4447103	7.37563801	7.3207807	5.56700626
4.89413561	2.34854753	6.41366678	3.38309624
7.02731704	6.27270892	7.87520925	6.51930024
-2.9534214	4.78489834	1.98121746	3.35131846
3.22405267	6.4702611	5.22931102	6.33255079
3.70377313	5.25567824	4.62838304	6.12739386
7.6248985	7.02189136	7.0229667	6.82138217
8.31618901	8.52659738	7.37604426	8.87728142
6.79989373	6.91573836	5.16283664	7.55187552
6.06506732	6.86397971	7.02784684	7.01212876

2.96316742	6.04016437	4.43620166	6.67395772
6.41887928	4.31295883	7.17588545	0.25072817
5.49233282	-2.9534214	-2.9534214	6.80485743
8.08209594	8.141993	7.72904906	7.89270346
3.22405267	3.63891997	-1.0106329	5.18667473
10.8694163	11.1459032	10.6177853	10.9506864
6.73373856	8.02510739	7.8797152	6.13984836
9.92778348	9.01519942	9.66962567	9.9415285
9.16210079	8.00961682	9.9413345	8.05233497
7.82160221	8.3101101	8.27233704	7.25688345
9.07969298	8.5775445	8.72159908	8.39781174
4.53211885	5.40616306	6.31464218	5.14712169
7.41678689	6.54126881	8.36427312	6.60151717
6.66440342	7.89460692	7.88151365	7.54193878
4.02151984	7.07071281	5.47779245	4.26467214
3.48376111	5.9373248	1.74876101	5.32125485
9.83260126	9.76048401	10.4642436	9.78691333
4.78947547	5.32013394	5.72818649	2.74859673
4.10861867	5.33580776	6.08418635	4.8949682
3.38048107	7.19622581	-2.9534214	7.54603867
2.94100092	3.36875031	-2.3036717	5.5577538
2.23476654	6.39837834	-0.6366176	5.22224711
10.4108012	9.79420266	11.4515588	9.4791358
-2.9534214	5.11060876	1.77995038	6.37399488
7.09412721	7.52624878	6.09197413	7.7343856
3.37222157	2.94700994	5.29555731	-2.2872455
9.03510155	8.81150394	8.90849868	8.41968236
2.86062093	3.31440997	5.27654769	-1.8332537
-1.2298595	5.75115375	3.63162582	3.7181568
7.37812513	5.5905802	6.22824039	5.56007248
6.92868148	5.32799214	4.85031161	6.23434213
2.50031482	6.53534177	-0.8115387	5.71168074
6.21230063	6.71462911	7.99468161	3.78311601
-2.9534214	5.39498462	6.39490191	-2.9534214
-2.0115616	6.59109727	3.24133144	5.79278609
5.49423693	5.62925347	6.89081076	2.55758639
6.38848796	6.67435888	6.97996607	4.97708254
6.65422068	7.03695178	7.0663048	5.67578065
6.00756993	6.29693328	6.86012852	6.63928901
-0.3527908	7.13315292	4.79030906	4.43098864
5.94629525	5.99768864	6.33578564	6.85670657
6.11542074	6.2335019	5.8690011	5.25123509
7.58122621	7.73679978	7.74987356	6.88472632
4.08354266	2.20231308	3.99275557	-0.6006549
0.84297312	5.79291687	-2.9534214	4.01713976
6.70689576	6.76107971	7.39757026	7.05796675

7.0922427	7.63040573	7.31812717	6.66321297
6.83712817	6.41783861	8.23484212	6.06018317
1.02258002	6.39557674	7.82141671	-1.4884482
8.47551532	8.20014691	8.57454499	8.79378936
7.42179769	8.11754885	7.96869212	8.32435182
-2.9534214	3.96038469	0.91975246	5.21931602
5.16905678	7.86588144	7.80634807	6.16443937
8.53776731	8.51265895	9.38775351	8.35838873
-2.9534214	6.22405786	3.0240799	3.95506514
3.78067645	6.51224949	0.11622035	6.92396206
8.23851793	8.57259201	7.86842383	8.40524632
3.48376111	-1.3152731	8.11651186	-0.0551215
5.14016201	5.93088917	5.71412616	6.74395428
3.55846245	4.25230982	-2.3036717	6.77422799
6.92727272	-2.9534214	-2.9534214	6.14449121
6.41184502	5.52372781	8.60452624	4.66441169
-2.9534214	5.96907926	2.62096701	5.32943335
4.95623646	5.04060282	2.45696755	5.92639403
2.48507573	6.43340215	-1.5168928	5.93535263
6.44667828	4.43075909	8.00544261	-0.6006549
9.7662894	8.89057208	9.94510154	8.14367518
-0.4667031	6.02694912	6.76396087	0.15580464
4.08354266	7.70018276	0.67627567	8.01310057
7.34895954	6.72805402	6.85189632	6.37267639
6.98189872	6.39931099	6.96562553	5.37228586
6.98934343	6.36439472	6.19680657	6.15679961
3.18686093	5.23493063	1.35207999	4.83112238
5.48660534	7.21859059	6.03978041	7.82104193
4.9507001	2.42380945	5.46105171	-2.9534214
6.70443056	7.55079964	7.68183919	7.43482973
8.83571818	9.03153308	8.35297492	9.03207798
5.89388818	4.67828322	7.04238897	1.1208487
5.2658608	6.26761057	2.75257428	7.13464302
4.66652817	5.2411863	4.93241502	6.47697894
5.94768557	5.59547144	6.02195428	6.78120138
3.85960771	4.0198108	4.78647473	4.77234132
7.0047866	6.77551857	6.78908954	6.08937197
7.7162369	7.39306625	6.31995716	8.12108211
5.4240542	4.00518218	8.06837561	3.31882493
-2.9534214	4.30103128	1.17569549	4.71938556
3.11937512	7.09394169	-0.8115387	6.83962974
9.86119407	9.72484278	9.9421964	10.2487376
3.14868499	6.02815552	4.12008362	3.5132435
3.82975838	6.67050113	5.40713653	3.82998523
2.40636795	4.29302445	4.00600611	4.51943363
7.18180526	8.20228607	6.56121871	8.1571397

4.82622064	3.89311802	5.56094691	4.79220231
3.55846245	2.69083085	4.4937717	3.59692625
5.32268682	6.43066781	4.01258603	6.3713567
10.1792036	9.79500018	10.5245444	10.0628877
5.05500357	4.19330716	6.216889	0.64950605
5.03943957	5.28826602	3.35960042	5.53906892
6.15085842	6.69880717	5.33808972	6.44722403
6.88145019	7.32303661	7.00740313	8.05972772
6.12651209	-2.9534214	-2.9534214	5.24259969
7.16217784	6.6455642	8.1107736	6.62938643
3.20557665	7.14432208	-2.9534214	7.58185224
4.49836318	4.64721401	7.83679314	-1.4884482
5.2658608	4.68441757	4.44595762	4.05050546
5.27696412	6.33739824	3.3070404	6.80485743
6.69948748	4.2890043	7.78776779	-0.0551215
5.49042618	7.17459876	6.35662369	7.41190612
2.17903669	6.11927633	0.6083965	4.7110632
6.74097347	7.35751686	6.86649901	6.44597082
-2.9534214	6.05325967	2.82912145	3.2284179
2.82475243	5.06901412	-0.8115387	5.54376288
8.02100047	7.48730572	8.18072922	8.05027468
10.0111007	9.60858542	9.91852334	10.2980164
7.13741431	6.24910588	7.34444536	6.99851596
8.49704073	7.89262127	8.12374771	7.73181688
8.5375365	6.74576175	8.1107736	7.93931607
0.97973075	3.53412523	2.10952377	4.70269254
9.4657023	8.62834555	8.45553733	9.18863175
1.18228212	4.47389578	-1.0106329	3.63261538
0.11146565	2.90559992	5.26281438	-2.9534214
8.30131203	8.48985461	7.32541266	8.95135517
3.44490803	5.51686898	5.24615995	5.25696345
-2.9534214	4.74730093	1.3099641	6.10374787
8.07575186	8.0341424	7.89225751	7.68265377
8.1513066	7.69980429	7.78824721	7.50149292
6.26789738	5.504787	6.83992183	6.14911918
-0.7256766	6.2889036	2.99779047	6.32983565
5.91962137	6.98819441	6.40619009	5.52251876
7.05014836	-0.6895489	8.23870593	2.87317316
8.09156005	7.98249055	7.90513381	8.84293946
6.49502494	6.08258692	6.41739064	5.05148149
10.8330634	10.7574246	10.8503265	11.3005449
7.55820163	8.35934724	7.36901063	7.99864247
-2.9534214	5.29830058	6.22824039	-2.9534214
5.99820758	5.12646603	8.17524125	4.36356168
8.32718017	9.36790476	8.88851569	9.43875505
2.60270609	4.98949561	3.18419106	2.82770822

0.93556977	6.36343921	1.12801103	3.32973763
7.82463215	8.58001439	7.88689558	9.19442706
9.09090405	9.11778024	9.24539778	9.56634903
2.86062093	3.61984204	2.67173693	5.72620734
5.25242302	4.50888322	7.82562656	-0.9773123
8.71558998	9.1056888	8.46126081	8.91286732
8.11711626	7.93472181	8.24814618	8.46925464
3.49901402	3.24969219	3.07526381	2.12826873
6.25784505	2.88444049	6.29183246	-1.4884482
12.9045343	12.5323678	11.809086	13.2898873
4.71305236	3.56769861	7.23897172	-2.9534214
2.21642809	3.67633528	-1.5168928	4.73177979
3.02769386	0.41054961	-2.9534214	5.81236949
-2.9534214	3.56769861	0.46223352	3.32973763
3.40497957	5.46079184	5.52687975	4.79614191
-2.9534214	5.44642523	1.81047971	6.06508916
9.51166454	9.6929652	9.73983682	10.0463405
6.13019033	-2.9534214	-2.9534214	5.30475742
5.48277435	4.79910194	6.74926194	4.97360788
4.65295524	6.54885368	3.70658451	6.04536428
13.4318232	13.0755609	12.1064461	14.0705853
4.72282959	6.03777058	7.5602322	4.41570593
7.38070075	6.97699314	8.2383551	6.94629243
6.35430103	4.18899889	5.88701795	2.85817682
6.26789738	6.85244821	6.22682634	5.96715064
4.74858283	3.44299212	6.58900094	-1.8332537
-2.9534214	5.76424456	1.86966605	6.58339411
5.83650656	5.44100065	7.5035534	5.05477378
6.54639936	6.20070876	7.21278211	6.54995429
4.54319748	-0.2545545	-2.9534214	7.3252736
4.32091024	6.00505959	6.08105942	5.56238745
7.79713084	7.08004929	7.88375856	7.74410521
3.63639923	3.33014576	4.38133172	0.71755754
7.24649614	7.37279187	6.58679794	7.37879455
6.9510366	7.8889738	7.51286581	6.70152523
3.44490803	4.77055351	2.45696755	4.14620829
-2.9534214	6.44428795	7.17588545	-2.9534214
2.86062093	4.0198108	-1.8572999	5.79868907
9.33248865	9.24307068	9.16959291	9.98704487
7.64045717	5.45362641	7.98760192	3.74286052
1.99763475	3.67633528	-2.9534214	5.38803298
7.50583822	7.55205822	8.38818966	7.39121923
6.21576679	5.40430599	7.4372386	5.4215733
6.85056833	6.88210707	7.5000457	7.08156194
7.59415658	5.32799214	7.57864088	4.02387492
9.93033495	10.8072017	9.32139805	10.9925433

1.39290183	5.73499085	-2.3036717	4.90594756
5.32054209	4.86269549	6.35403534	5.18967283
6.66609358	4.81316706	8.09768176	4.76431974
5.76733038	7.14376567	6.74334018	5.28526892
6.82128808	5.88501751	7.10067941	4.49055026
-1.7015481	4.54641406	4.4748358	-0.4437536
5.82747248	6.07326691	7.3207807	3.38309624
7.88502072	6.52084535	7.27156843	6.42831067
-0.2472184	3.81698783	1.35207999	5.25696345
9.56200627	9.08583957	9.17601856	9.067958
9.09906006	8.81255452	8.94141616	9.54558358
6.91595281	7.79500904	7.22416574	6.87265119
7.77458931	7.53942274	7.7429654	8.2422648
7.0279744	6.82910498	6.86377224	7.79322377
8.62855006	7.27811046	8.75974453	7.84596036
5.90966849	-1.5257463	1.86966605	4.2760006
2.28842343	4.41977033	-2.9534214	5.98624328
6.96483467	5.88368521	5.86537057	6.06182035
0.93556977	6.33154696	-2.3036717	4.62951001
5.84846467	3.81139309	7.5421542	-1.4884482
5.68803004	6.4622504	5.80600407	7.42659372
6.85872055	7.5591695	7.31546875	7.85873098
2.43836816	5.45182948	3.57050692	6.26735377
6.74417737	-2.9534214	-1.8572999	5.11278535
8.92488972	9.18665528	9.1469685	9.52748012
3.33870244	5.65290819	-0.3398746	6.57080111
5.70949735	6.72359291	8.92013824	4.43604712
6.91949983	7.48818264	7.81437288	7.33546119
4.68331671	6.06270941	6.34493934	5.59667342
3.74273715	3.45021042	4.48433482	4.24751088
7.01874396	7.34450157	7.00245323	8.10885983
3.58013888	1.9198826	3.3070404	3.75100198
7.28770016	7.25768523	7.91174915	7.36493636
3.32164624	3.49975191	4.93588089	0.05419288
4.88835524	4.39753848	4.86493027	5.73649465
-1.7015481	4.34041218	1.95412777	2.91724899
4.83526253	4.76477541	6.27551156	4.64706639
2.90709921	5.3666536	-2.3036717	6.89853473
7.76357983	7.1870739	6.63130036	7.76586341
5.25242302	-0.8224059	3.95908677	3.06823308
7.66728752	7.6128232	7.50763498	8.41840499
7.82614474	8.02087162	8.88560624	7.58754626
5.01577414	-2.9534214	5.80600407	1.51431472
9.61961865	9.33574683	9.75671531	9.73834376
5.16428099	-2.9534214	-2.9534214	4.33134582
1.73962402	3.45739279	-2.9534214	5.33214925

6.87561867	6.19319955	7.25640204	4.88760193
3.75549476	-2.9534214	-2.9534214	5.01813862
6.21576679	5.70663938	6.42973455	6.53588716
5.40596176	5.06196358	6.72742894	2.24815491
7.35944364	6.96444443	7.31746303	7.74053192
6.78441544	6.28789674	7.16632753	6.79209191
7.27002643	6.74576175	7.94953117	6.8548191
6.35534899	8.48678662	6.58348713	9.07243166
7.98590619	6.57550367	8.13547621	5.69067139
3.20557665	4.38252461	4.41648957	4.62951001
0.52309424	4.7531493	7.95722612	-2.9534214
6.66186447	5.16645911	4.31367457	6.58908206
5.6475388	5.54242317	7.11450905	2.76476989
6.09297892	7.06602182	6.66614828	7.36626194
2.68503367	8.16602988	-1.8572999	7.84405876
7.809036	7.93953843	7.17148197	8.10370338
7.07453427	8.7928163	8.03159422	8.46462092
8.5465107	9.97343047	9.30658185	9.76848357
3.65694381	7.51424542	1.22185416	7.4869428
7.97025848	6.51569398	5.8994972	8.03992889
6.15085842	6.0305653	8.19272975	4.99433145
4.01091842	4.62824616	4.60244686	2.73224021
5.81838147	6.23245561	6.20544758	6.85859158
5.42804411	7.6595015	5.62890177	8.47048779
7.26166674	6.22511026	8.13848751	6.21681405
6.87999452	5.74236008	8.60778918	5.05805857
6.93570474	5.74969185	7.65159463	6.01187807
7.91411502	7.26076751	8.01694231	6.59700771
6.23525256	5.51514917	6.95114101	5.87703219
-2.9534214	6.74722763	2.47613788	5.87144181
1.8146881	4.81875494	-2.9534214	5.68855353
4.93676553	5.17954751	5.27381147	6.04039063
8.77396803	7.97750323	6.83436144	9.51034613
5.65266279	4.86809514	5.74803792	5.94780194
-2.9534214	5.87163889	2.03391701	4.57092982
3.35555935	2.20231308	3.25249288	3.76714835
1.68731977	4.27687621	-2.9534214	5.4593289
8.46098453	7.35511546	7.96190846	8.45437403
6.3171345	5.21599919	6.48401126	6.1367448
7.95616711	6.98881416	7.0968142	8.48520397
10.8554109	10.8364619	10.4247814	10.9491945
6.12651209	6.63768218	6.02195428	7.64601472
8.83026387	7.48905903	8.73409488	8.19493146
-2.4069576	4.72961219	7.81907258	-2.9534214
5.66116254	-2.9534214	4.2867837	2.15305622
4.37151092	5.84313336	6.12878122	5.2397097

9.23294511	9.26011307	9.58151019	9.43560152
7.41024671	8.91507009	7.12972141	8.72105473
6.22267421	-2.9534214	-0.48063	3.56956781
9.594987	9.49145541	9.53413421	10.0645218
2.54508945	6.83532092	-2.9534214	7.24971905
7.91162373	8.62516007	9.02677597	8.06626741
6.41385832	5.69149072	6.20688273	6.86610708
1.68731977	5.97909483	-2.9534214	5.90648709
8.62703227	8.52809044	8.38280322	9.31499435
7.33733835	7.88698038	7.55347947	7.17074677
4.79257357	5.00428334	5.09625162	3.52278492
6.2375278	7.45716877	6.4504798	8.03410268
9.34889185	9.25522753	8.92254227	9.33932855
-2.9534214	5.38371888	1.5459812	4.8727556
3.26923333	5.4482289	0.21080887	6.23869107
2.91848853	4.82432125	7.74444851	1.43568281
1.8146881	5.92959859	-2.3036717	5.85453952
5.08310866	1.83419519	4.95991161	-1.4884482
5.98201511	5.61808046	4.79030906	5.85831274
9.80083632	10.4696785	9.45481912	10.4790053
5.16428099	6.41138098	6.85464561	5.71792434
-2.9534214	5.36283374	1.26658167	6.12895657
7.39300014	5.19251823	7.94050156	5.68005086
-2.9534214	4.3208561	0.53716524	4.98745661
7.64945618	6.17370874	7.92750327	6.69204212
7.6421756	4.75022808	6.6930234	6.64585317
0.02980441	5.02376745	-1.8572999	3.39353517
6.62584178	7.30274358	6.18665954	7.09920781
5.99148279	5.46436125	6.69097377	5.81819305
-2.9534214	5.22444384	1.50922995	3.28558265
6.03660504	6.77120207	7.25432149	6.39622676
7.01211443	3.43573752	3.95225768	5.48396131
4.2595417	4.4949899	4.46527383	5.5646987
6.11665733	6.60409925	5.83413653	7.76234367
6.34588985	6.38431565	6.1939147	7.39382137
9.4553544	9.3847199	8.166048	10.3256205
7.42778777	7.57122019	7.0367511	7.46360073
6.7003125	6.61618309	6.4235758	6.16900388
11.0183895	9.78727232	10.4424028	10.8492864
8.12604715	6.94860348	8.60615863	7.1377511
4.80796504	6.53364385	5.30630855	5.08728996
7.21682142	6.90592792	7.72003143	7.11269694
7.39453022	7.32008467	6.25206958	7.94109624
5.58458794	6.19856726	3.73072955	7.03231013
-2.9534214	4.54977803	0.74110411	4.38464608
8.21012581	8.08516837	7.45062652	8.4565537

5.19504468	6.14508217	5.98227905	6.38972346
7.24875373	6.61698509	7.87295099	6.73063578
5.89388818	7.22281152	5.99396413	6.38056931
6.3605774	6.75817452	4.98689393	7.38076347
7.3563064	6.74061941	8.30285521	6.52287067
7.41377203	6.83876268	7.36451676	7.56521133
2.23476654	2.79655573	3.35960042	3.01513998
-2.9534214	5.12195314	1.17569549	4.90594756
6.30090812	-2.9534214	-2.9534214	5.72414105
4.53582119	5.89033441	7.05280121	4.43604712
8.06937976	7.45582459	7.48000596	8.61046274
5.72741579	3.90367309	7.15447634	3.96209569
5.20902504	7.95230469	4.87218442	7.40933643
3.02769386	5.51858675	-0.2116394	6.36075546
2.06067066	6.10562691	-2.9534214	6.68568525
-2.9534214	4.61866789	0.53716524	5.91194355
3.90038566	5.19036453	0.74110411	6.63819207
9.80016304	9.27898482	9.69630897	9.86435761
7.74210704	6.14619376	7.31413771	5.91012702
6.99473374	4.8408925	5.58321949	7.13852707
1.88603897	6.42151575	5.50837622	3.98988023
-2.4069576	5.86219949	8.23378655	-2.9534214
2.40636795	6.03176868	4.20296624	5.74670913
1.6330477	4.26054518	2.97101307	4.50024244
4.12345771	3.35343223	7.75331527	-2.9534214
-2.9534214	5.52543744	4.50781229	-2.9534214
6.77112706	6.26249414	6.90325739	5.37492235
4.7517699	4.70266566	6.01868937	4.58920915
1.10463976	5.61487221	-2.9534214	5.36434737
5.2569162	5.08301267	6.19825034	5.22809149
4.87672456	7.51166023	4.64541836	6.77821691
6.81444586	5.77721766	7.59243212	5.88630161
5.65946659	7.29574836	4.44595762	7.64874069
5.03421403	6.28588092	5.78885745	6.54059146
7.3682956	7.31909935	7.79589632	7.51768829
1.54760822	6.10905139	-1.5168928	5.55543138
7.58926559	5.13768688	5.96032386	6.01018319
1.54760822	7.3478872	-2.9534214	6.42449807
3.68388926	7.23121651	3.42024471	6.12739386
1.51796573	6.37106564	-2.9534214	6.18411546
6.5646365	6.40024305	7.52442242	6.23434213
4.55419169	4.40126766	8.44280426	-1.4884482
5.53180085	4.45965971	7.90203624	0.15580464
8.34786177	7.93472181	8.35232662	8.45561996
6.59423273	5.56753259	6.65987459	6.40011472
4.73897916	-2.9534214	-2.9534214	5.79672409

7.03779907	6.88942748	6.77367761	7.81425893
-1.7015481	5.58239112	1.26658167	6.80192155
8.17375716	7.74855785	7.48592895	8.96388665
9.64437279	8.84782676	9.1653547	9.79874311
8.03120049	7.47983029	8.01161456	7.07509137
5.00513027	-2.9534214	-2.9534214	4.34216457
9.52418266	9.58869058	10.0021118	9.57683544
2.21642809	5.55921125	-2.9534214	6.93650962
7.13802338	6.50879676	6.59668504	7.73233099
7.59814601	6.55723481	7.74494254	5.82399319
5.02106678	6.38901846	6.92870306	5.39584256
-2.9534214	2.895059	-0.8115387	3.61488117
1.25595849	4.67212268	-2.9534214	5.47415876
5.78143352	4.9027087	5.2545112	4.50506423
9.23251741	8.41804569	9.60333373	8.55719448
5.17143875	-2.9534214	-2.9534214	6.16900388
6.45745442	6.83256158	6.4863768	7.52838507
6.4047763	-0.8224059	7.49182772	2.0512437
3.90611811	5.99891975	7.98968779	2.88801522
6.12159312	6.59028074	6.64194951	6.86141449
4.45209075	3.04561548	6.70627552	2.44165109
8.66619606	8.21928613	9.11062836	8.28002914
-2.9534214	5.20750481	5.88163648	-2.9534214
8.60979132	9.03753596	8.24465699	9.09379426
6.71997283	6.88277409	6.15875235	6.85764939
7.6205467	6.46937322	7.93618179	6.83389224
-2.9534214	3.96543144	-0.0938779	5.09050164
9.5596214	10.0603896	10.5723704	9.50057093
10.4913609	9.69837465	9.9279087	10.7060106
7.12579278	6.96759186	7.12820737	6.44346114
6.00890246	7.79784107	8.1545664	5.18367039
-2.9534214	5.98656113	6.5420165	-2.9534214
6.50071915	5.56420981	6.78332934	4.39507384
8.42425375	7.33039015	8.64479193	7.66391167
10.4200959	10.4725061	10.4032694	10.230748
1.83886594	6.76976036	-2.9534214	6.83771977
0.11146565	5.50131634	-1.0106329	3.58786429
6.38027431	7.96338325	6.01868937	7.79371646
4.7738844	7.34498572	6.67964842	6.26877206
7.03322255	7.12809845	8.08642053	6.70676681
8.00406127	5.02859763	7.64366846	6.22998003
8.55884507	9.01808637	8.54279139	8.73881768
6.62062146	7.72308396	7.17588545	7.7882876
6.1240547	4.28497292	6.78717203	4.79614191
3.59441098	5.88235169	2.94372925	5.31851834
-2.9534214	6.68357561	1.89837289	5.83169062

6.17242428	7.26946505	7.97922815	6.11483091
6.49787485	6.5471716	7.06788517	5.31851834
0.97973075	7.19084936	-2.9534214	5.92819021
5.49803764	-2.9534214	-2.9534214	4.91322098
8.67020199	8.9412418	9.12398364	8.19269165
4.85317808	6.75161636	5.78693963	6.74089168
5.12303556	5.08301267	4.05144749	5.27401328
9.07969298	9.27276404	9.8644198	8.94093968
5.70457163	5.74676361	5.5986082	6.74293414
2.23476654	5.97784668	1.02763635	5.23100482
6.62931153	6.61216638	6.34754406	7.58697787
5.36075645	6.04969999	6.25484731	6.05196925
6.54914967	6.57880057	7.2262902	5.27965208
3.12921138	0.72812905	4.58489335	-2.9534214
5.36908165	5.28423258	5.14198196	4.69004478
2.78796937	6.5538882	5.92762225	4.40025959
5.72579601	4.53287918	5.5986082	3.67601999
9.14780714	8.4782055	8.45342294	9.6174786
7.21220141	5.06901412	9.07078986	1.8231434
5.94907455	6.63213903	7.05996591	4.86902002
7.11345761	8.03354182	8.18656012	7.48205996
3.52904442	6.32469035	3.21874614	5.49369771
7.02402573	6.93901474	6.84269401	6.62938643
4.85317808	6.89472818	6.46614495	6.11798192
6.60396473	5.47855122	7.20920623	5.73649465
7.32722598	9.55811547	7.22345689	9.26559175
6.01422032	8.4959711	7.29537292	6.79897968
5.79540012	4.52606382	3.56156037	5.97238283
5.93932348	5.01649171	5.02318941	6.82524299
5.70785731	7.52368502	7.33200406	5.39324406
3.67719993	8.29089008	2.94372925	7.64383023
5.92245248	6.00014981	5.9989432	6.73577284
5.17619094	5.22444384	5.92936199	5.40876529
-2.9534214	6.66740748	1.71688244	5.89734686
2.19785352	6.36056887	-1.8572999	6.99937053
5.9860803	6.20177832	4.526323	6.95776904
-0.1488474	6.54295779	3.14878798	5.27683544
5.70621541	6.58782838	6.9588274	6.08937197
3.60149444	5.03100667	4.80932932	2.93164669
6.73534945	6.84698957	6.92172827	6.15065854
9.47778203	8.77801265	9.98360183	8.72622596
5.92245248	5.1287172	7.09060824	2.96001808
3.43701025	5.5339553	2.41784535	5.36699839
2.84876357	4.17599634	3.35960042	4.85019564
6.02611431	4.88683634	6.54655771	5.30752013
1.51796573	4.71170388	-0.2116394	4.55241591

9.91041973	10.4348526	11.2272025	8.86514355
6.96620724	7.73938005	9.18296273	6.05032084
7.48683744	7.95325762	8.06045905	7.04808921
-2.9534214	3.85010816	-0.48063	4.90958885
5.59527235	2.60288549	7.67925673	-2.9534214
3.38869355	5.99891975	4.03860963	4.42591237
-2.9534214	6.48527214	1.77995038	4.32590587
8.80766394	8.73894419	8.61239907	9.35560438
11.0723622	10.6443109	10.7287179	10.7761889
0.97973075	3.52731577	6.2576197	-0.3022547
8.96027614	9.19379544	8.88605423	9.68104191
-2.9534214	4.80755745	0.3831957	4.03057878
5.39173131	-2.9534214	-2.9534214	4.89863728
7.11963838	6.13951134	8.18656012	5.75280329
1.32605442	6.14841438	-2.9534214	6.52998513
5.05758132	5.23701887	2.91591949	6.53824123
-2.4069576	3.69468524	-1.0106329	7.08639589
1.32605442	6.18996931	-2.3036717	6.27443134
0.74402323	6.7391468	-2.9534214	5.94247967
3.92882286	-2.0697503	-2.9534214	5.97759612
-2.9534214	3.27430282	-1.0106329	4.81955621
6.36786548	4.44888971	6.34232992	3.474434
8.9964956	8.02299106	8.5253551	8.91988037
7.55410004	7.67806544	7.16411283	8.54992164
5.91109454	1.36951236	0.6083965	7.56924571
6.12159312	5.8982732	5.63744151	7.12135847
0.84297312	4.18899889	-2.9534214	5.46428915
-1.7015481	6.18673183	2.43753906	4.08955166
6.78285844	6.44428795	7.2980686	6.56272977
10.0421821	10.9106984	9.90289168	10.8367304
5.07549778	7.84993752	4.72770227	7.50390373
6.64910223	5.17737434	8.06006207	4.64706639
2.03996322	4.69357047	1.77995038	3.79890887
6.66609358	5.83074215	7.90998802	5.32943335
6.83111434	6.53109322	6.09507751	6.78417968
10.0858484	10.14115	10.1410916	10.4664676
5.81228882	6.39183281	6.04460409	5.56007248
6.69369906	3.40634873	4.79794728	4.42591237
7.51571435	5.45182948	8.29410496	3.9048679
-0.1488474	3.67633528	-2.9534214	4.32590587
7.83481148	8.93015258	8.59907156	7.92049047
4.53582119	4.35586839	5.63956857	2.27098141
1.54760822	6.91899373	-2.9534214	7.08478638
6.074007	6.65808654	6.6173379	6.77921242
7.90912813	7.04114068	8.36137631	7.10080132
4.84723092	5.19681602	5.45623275	5.44183251

6.90095934	7.12922319	8.41637579	5.18667473
-2.9534214	5.35131312	0.6083965	5.10009397
5.91678469	7.63119986	5.56318971	8.65127015
7.20175198	8.54672971	7.24037392	8.20940602
-2.9534214	4.67828322	0.11622035	4.21256355
5.74830914	6.63213903	7.44698758	5.60118393
8.03904804	7.91072156	7.93358371	8.44906679
6.67870705	6.38619862	5.6978876	6.87078447
6.41486391	2.56348907	4.2867837	5.60792337
6.9371053	5.76424456	6.27551156	7.06777712
-0.4667031	6.59272894	-0.0938779	3.474434
6.62671	6.0305653	7.00327939	6.32302536
8.25039629	8.36054492	8.56813827	8.45250311
7.20291677	5.26798508	7.17661806	6.02032277
3.26923333	3.57432054	-0.2116394	5.86207611
7.97878372	5.87029418	8.31521669	6.18110578
6.59954923	7.81121781	7.20562146	7.71838533
5.07549778	7.07129812	6.82691409	5.98968768
6.58711344	6.47380719	5.22081219	7.36228154
2.77549695	4.57970594	4.526323	3.79890887
4.08859285	7.41259783	4.18560214	6.48310138
2.25287481	4.80755745	2.45696755	5.99483892
10.5709331	10.4258988	10.4781274	10.3346211
9.16777884	9.38177367	9.17161548	9.17715964
-1.0415346	3.13791053	-2.9534214	3.26298783
9.45901508	8.56159464	9.28143144	9.12740796
4.5938018	6.62178777	5.98395416	6.00338365
5.01577414	6.85040364	4.35568384	6.80778735
3.11937512	5.69149072	4.9768346	4.7481415
6.7906267	6.26556618	6.14683851	6.43084683
7.20175198	5.81684815	8.51032315	3.85286094
4.7738844	5.33969975	5.62675891	5.28806916
6.21692032	2.62856451	5.87623486	4.14620829
4.63925341	1.36951236	6.14384461	-2.9534214
7.83330795	6.75526346	8.08212593	6.12112606
8.58811345	9.05376816	8.7670595	8.6664147
-2.4069576	3.32229932	-0.8115387	4.50986995
0.33191111	3.69468524	3.23008299	1.72736257
1.48770139	5.73646771	-2.9534214	6.95688946
3.65012807	7.90613572	4.0704926	6.59700771
7.38429892	6.03896797	6.08418635	7.86952121
5.79075959	-2.9534214	-2.9534214	5.40618999
4.45990662	5.94883709	4.19141343	5.51297557
8.6252957	7.96998977	7.52096515	8.84864115
-0.2472184	5.61968191	2.08475918	5.24836237
-2.9534214	4.32478858	-0.2116394	3.2400335

-0.4667031	4.38252461	-2.9534214	4.26467214
8.60935216	8.77280427	9.24365092	8.52461935
1.8146881	4.22731785	8.00544261	-0.6006549
6.66186447	6.25220653	6.50749395	7.007039
6.51579475	6.32174179	6.4235758	7.02561433
1.95402673	8.33931909	0.67627567	7.30744288
7.64517794	6.85992028	7.61476819	6.53588716
7.07771248	7.44547737	8.22212409	5.96540235
5.54839069	-2.9534214	-2.9534214	6.31481035
6.6508104	4.34041218	5.09315077	5.2397097
6.16407582	6.44338393	7.15075278	6.08776577
-2.9534214	5.20750481	3.32829474	-2.2872455
5.29455358	5.35900375	3.34924049	6.6205267
2.65810979	4.48448139	4.96331213	3.10680874
0.46213879	6.36725747	-2.9534214	5.84696336
-0.1488474	4.48448139	-2.9534214	4.71523038
7.86786587	6.28588092	8.16715429	6.53706467
2.99579136	6.69425456	3.93850176	4.92406282
-2.0115616	5.30428797	0.46223352	6.22560471
7.20058625	5.01162075	7.70943912	5.25981912
3.91182789	4.18467771	5.42204162	3.58786429
7.38891198	8.50791065	7.45425631	8.19940066
1.32605442	5.25773668	-2.9534214	6.82716953
-2.9534214	4.53287918	-0.6366176	6.66644481
8.55223417	8.25140615	8.7646253	8.41904382
-2.9534214	3.02642617	-1.5168928	5.18967283
7.1097364	6.8592426	7.37221198	7.09041185
4.93676553	5.74382942	5.86173088	4.23595558
5.65776865	5.97284325	7.03271052	5.27965208
-2.0115616	6.4702611	1.74876101	5.11278535
5.80617032	-2.9534214	-2.9534214	5.46428915
10.1749066	10.0087692	10.6613562	9.87102636
7.24649614	6.41507459	8.04208437	5.1926647
7.5231939	7.67575797	6.96223047	7.97233411
3.33019955	6.18348706	1.58181946	6.70990264
6.73051136	4.69660858	5.7181574	6.39492844
9.77981737	9.8347551	9.82091616	10.1332882
4.34220995	5.79007514	5.39711356	5.49126977
-2.4069576	4.09541959	0.0149924	3.38309624
5.13040038	-1.5257463	-0.8115387	5.05148149
3.72986571	-2.9534214	-2.9534214	4.53366132
3.52159522	4.07221469	4.5626468	3.9048679
4.31230151	7.95959446	6.80149162	6.05032084
5.83049013	6.3187872	8.33929923	3.30782906
1.29143213	6.08953764	-1.8572999	6.4879807
9.21559356	9.63624234	9.17894649	10.017841

5.58637418	6.70259007	7.25640204	4.58009843
-2.9534214	5.04060282	-0.0938779	5.44685317
6.39461766	6.04255419	6.03008438	7.00193122
3.77442211	5.53225575	3.5970184	4.98054886
3.41305416	1.24222681	4.90791795	-1.8332537
-1.4465118	6.56473641	-2.9534214	3.58786429
5.01842289	-2.9534214	4.54005182	0.25072817
5.41201782	6.24184502	5.1539358	5.97238283
5.30977038	3.33014576	7.7454364	-0.7767262
8.44359853	7.10713087	8.34224067	6.94363096
6.47491631	6.57302604	6.5363198	6.10374787
5.80463664	6.59761291	6.30663281	5.64553002
6.24433206	6.02936091	6.77270887	5.01476155
1.71370892	5.92312832	5.33020982	1.69396844
7.43474491	6.21984058	5.7080581	7.6367077
8.18086817	9.79508876	8.27849254	10.0830816
6.53626979	6.41230527	5.65226549	6.89761827
0.26210614	4.55982314	-1.8572999	4.62951001
6.980541	6.24496129	8.01653318	5.75482896
7.26445869	8.50075879	7.8381829	8.30892436
-0.2472184	4.14963431	-2.9534214	4.84259657
-1.2298595	8.03923723	1.12801103	3.28558265
5.15708754	5.34745237	5.87803765	4.07016065
3.35555935	5.91793113	5.8690011	4.10868549
-2.9534214	3.84464064	-1.2416692	6.2516598
7.77302166	7.91202913	8.17377426	7.30121901
2.14065035	6.059173	-0.8115387	7.00107816
1.95402673	5.19036453	2.20453818	4.01037301
6.67535423	5.93603997	7.45183747	5.74058913
5.36908165	6.72508148	6.28505451	6.40528241
6.95242236	1.45810943	7.39127235	-2.9534214
6.83186744	6.85652869	7.24666702	6.91402613
7.35525914	5.08996134	6.99416546	4.91322098
-0.5903867	5.95138294	-2.9534214	4.77234132
7.07771248	7.57741403	7.90557578	7.16542654
-2.9534214	7.51381488	1.5459812	6.12112606
2.51539463	3.65149987	-1.5168928	6.47329296
3.17741124	5.76713757	2.65501136	5.43679432
-0.0567579	3.30647725	7.16485144	-2.9534214
-2.9534214	3.30647725	-1.5168928	5.01813862
-0.0567579	6.35384913	-2.9534214	5.60792337
0.11146565	4.69660858	-1.0106329	4.02387492
4.42838582	-2.9534214	-2.9534214	4.1023357
1.4251988	5.24948522	2.05956205	6.02536597
6.04312323	6.55221197	6.14234533	6.04536428
7.18828884	6.79123618	7.82188508	5.84316031

8.54559284	8.1734089	8.9799087	7.55012694
5.36908165	5.37994401	5.8470796	5.56700626
-2.9534214	3.61342625	-1.2416692	4.05708699
5.28358532	5.91010007	8.15084309	3.68454639
5.81838147	1.36951236	7.02703463	-2.9534214
6.27788016	6.99992437	6.39867446	7.37682294
5.99283026	5.60520443	7.04640258	5.18967283
0.58157832	5.63559941	-2.9534214	6.30930757
-2.9534214	5.15546067	0.0149924	3.75100198
7.91553668	7.59420944	8.67880231	7.21556143
-2.9534214	4.05811045	-1.0106329	4.91684399
0.11146565	3.33794975	0.6083965	4.15236829
7.55820163	7.05954669	7.51865569	7.51051278
5.87501343	4.77343391	5.95178978	5.37755402
7.92967681	7.40425951	8.0497023	7.66175413
6.02479758	7.63437205	7.52959285	6.57195048
0.02980441	5.56587216	-2.9534214	5.59214876
3.32164624	6.0837477	1.47151796	6.86141449
6.72241164	5.63401554	6.87915624	5.01137656
6.58354058	5.91793113	7.3450972	4.70269254
8.0393741	8.12743255	7.49770248	8.49371959
6.98189872	5.29228822	5.21511836	6.6760971
3.55846245	-2.9534214	-2.9534214	3.25155631
-2.9534214	4.71769805	-0.6366176	5.65206591
3.62253849	2.87374325	3.54349911	3.73467286
8.04750167	7.79819468	8.05369564	8.13902969
5.06272305	6.06270941	6.67550797	5.05805857
5.93372171	6.34613089	6.75907785	6.06182035
-1.4465118	4.27687621	-2.9534214	3.5510363
5.99148279	-2.9534214	-2.9534214	5.77093279
7.31650424	7.49953437	7.13500807	7.46174175
6.96002034	7.61322519	7.07104075	7.89912829
8.82232779	9.02187621	8.23413849	9.47161151
6.93500395	6.7183708	6.77076945	6.59361633
3.44490803	4.20188529	2.91591949	4.13380881
4.7064972	2.9264535	5.8690011	2.74859673
6.10797886	6.55221197	8.2355454	4.91684399
3.41305416	3.24969219	2.49505681	4.96313338
0.79434634	5.73351248	-2.9534214	6.85103672
9.87386454	9.28795129	9.01125366	10.4483541
7.93844452	8.05056104	7.38431275	8.94427195
-0.0567579	5.25773668	-2.3036717	5.08728996
6.01687191	7.02370693	5.86355187	6.83389224
6.16287923	-2.9534214	-2.9534214	5.96540235
-2.4069576	5.40430599	5.75590263	-2.2872455
7.99669232	6.18673183	9.32139805	5.39324406

8.17494476	8.31530778	7.55967068	8.77644521
6.55554675	4.27687621	6.88544345	3.2400335
-2.9534214	3.15568158	-1.8572999	5.95310464
7.37296004	8.49945463	7.03755787	8.65317194
-2.9534214	3.47872781	-1.5168928	3.99674355
-2.9534214	3.44299212	-1.5168928	3.73467286
7.96065002	4.21041272	7.26262575	5.53906892
0.26210614	5.40244653	-2.3036717	5.63457063
-1.7015481	5.99151729	-2.9534214	3.54168051
6.17242428	5.97784668	6.19536136	6.47452267
11.3140615	11.5753395	11.0739257	11.7210894
1.76508181	6.60733151	0.21080887	6.54645035
-0.3527908	6.3286124	-2.3036717	5.29086398
-2.9534214	5.06431759	-0.48063	5.1032773
6.05219949	5.62287949	6.56234033	5.67149778
13.0365358	11.3664121	12.1706895	12.502834
5.75467746	-0.078038	4.40653159	-0.9773123
5.60058504	6.37865197	6.80054139	4.80789656
3.76814055	-2.9534214	-2.9534214	3.5132435
10.7315422	9.73614475	10.2768474	10.4768593
-2.9534214	4.13178815	-1.2416692	5.59214876
5.96701066	6.25426993	7.19625916	5.53671621
-2.9534214	4.10916546	-1.2416692	5.41902078
-2.9534214	3.02642617	-1.8572999	4.14620829
6.52047375	6.02815552	5.48254005	6.73577284
5.52250116	8.64259379	4.47959313	9.23435522
5.97111828	7.72271145	6.29723199	7.12762526
4.33797509	5.40987004	3.80083084	6.64038511
5.77047634	6.34128595	6.0669038	6.55809722
-2.9534214	5.433736	-0.3398746	5.7888373
-2.9534214	3.54764849	-1.5168928	3.75909775
3.38048107	2.70296801	1.22185416	4.33134582
6.91808207	6.56556752	6.65987459	6.55344975
-2.9534214	5.28625071	-0.8115387	7.48328222
7.75328097	7.10941238	6.4074389	8.27580322
-2.9534214	4.47743295	-0.8115387	3.42440627
8.75563012	8.87841615	8.77094571	9.15355427
2.42245677	6.59191334	5.37430399	4.25897437
6.57366934	5.7260978	7.348352	5.47661565
9.31185381	7.18977167	9.40739138	7.47224444
-1.7015481	4.3208561	6.12574958	-2.2872455
7.47097119	7.70358458	7.08281306	8.42922676
5.91678469	4.21465761	5.15690885	5.56007248
3.52159522	5.47147368	4.3965044	5.26266914
-2.9534214	3.21621102	-1.8572999	4.7276602
-2.9534214	5.10375907	-1.0106329	7.5057092

6.67954404	4.89479435	5.56094691	6.52287067
4.98360399	6.33545045	4.85031161	7.04643636
5.72255097	6.62098843	7.33266155	5.47661565
6.43582104	4.51578003	5.15987579	5.38803298
-2.9534214	3.71879433	-1.5168928	4.05708699
-2.9534214	5.88101693	-0.2116394	6.15526679
7.92967681	7.58152854	7.88510383	7.94376238
4.12837033	4.91057988	4.73964987	4.92045792
-2.9534214	3.88248517	-1.5168928	5.38018091
7.0922427	6.16056702	6.79959054	6.1826114
5.23430904	6.00137882	5.9414818	4.92765874
7.7055932	7.87093306	8.26270932	7.62678732
-2.9534214	5.92183077	0.21080887	3.75909775
0.58157832	5.55084163	-2.9534214	6.91674272
-2.9534214	6.34128595	0.0149924	6.45969657
6.36474652	6.74282552	6.38732709	7.14394724
-0.3527908	6.00628442	-1.8572999	5.08084509
-0.0567579	4.05811045	-0.2116394	6.04867054
4.2595417	2.71500391	9.15070182	-2.9534214
-0.8749787	5.99645648	-2.3036717	4.7110632
6.1067348	6.11701038	6.66823347	6.02704312
6.05864775	5.86760099	6.19246658	6.22121607
8.19995299	7.64384696	7.19625916	8.94648919
-2.9534214	6.54632982	0.11622035	6.86985021
4.55419169	8.65181938	4.52171754	8.2361152
4.99171403	6.45419496	6.85556088	5.07761183
6.66016934	7.21647549	6.58900094	7.66283331
-2.9534214	3.29047973	-1.8572999	4.21256355
5.10321023	7.60799054	6.66614828	6.47452267
9.35112621	9.51552552	9.58992182	9.541189
3.92882286	3.41375224	3.28546824	4.69427303
5.51689244	-2.9534214	-2.9534214	5.34833813
7.93844452	7.55205822	8.722101	7.08880681
-2.9534214	3.31440997	-1.8572999	4.19476721
6.8182511	4.92100841	7.00162659	5.28246323
-2.9534214	5.26594122	-0.6366176	5.27965208
-0.5903867	4.79910194	-0.8115387	3.54168051
-0.5903867	5.11060876	-2.9534214	5.37228586
1.48770139	4.18467771	-1.2416692	5.84125502
9.20865207	9.44015118	8.9562115	9.60546155
3.39685954	-2.9534214	-2.9534214	3.20490233
5.54472051	6.81029482	7.68955895	5.08407113
-1.7015481	6.04135977	-2.9534214	3.95506514
6.53811683	6.20924313	6.07949342	6.40657145
5.91109454	7.83863662	7.15447634	6.95424749
6.16168165	6.9832268	6.32921153	7.21703154

3.08945741	4.20615531	3.25249288	3.59692625
6.5673523	4.92619455	5.5986082	6.36871368
-2.9534214	4.66283225	-1.2416692	6.24591031
4.92269506	6.74061941	4.82810207	7.36427311
5.70621541	5.39311309	4.79413323	5.9793297
9.16240019	9.72892912	9.2082619	9.60293364
7.48348642	6.91769246	7.8321509	6.38711391
5.76101779	5.45362641	6.4792685	5.2397097
-2.9534214	5.29429513	-0.8115387	6.23434213
5.78454891	7.23800969	5.62031118	7.14317418
7.14591802	7.71860744	7.83075531	6.89026555
-2.4069576	3.16448568	-1.8572999	5.38280302
-2.0115616	5.31223272	-2.9534214	3.35131846
5.39173131	7.42318269	5.5986082	6.93918424
-2.9534214	4.2356967	-1.5168928	5.96540235
-2.9534214	3.49975191	-1.8572999	4.40025959
-0.4667031	5.48559421	-2.9534214	5.75280329
5.02106678	-2.9534214	-2.9534214	5.59214876
-2.9534214	3.61342625	-1.8572999	5.17159008
7.79249588	7.4195098	6.71336118	8.37593352
7.124564	4.4667953	4.31367457	7.670365
5.68803004	3.58747391	7.47644046	2.61223772
-0.5903867	4.45607864	-1.8572999	4.2760006
5.88955434	3.19917447	8.37548353	-0.3022547
-2.9534214	4.05811045	-1.5168928	3.9048679
-2.9534214	5.07135666	-1.2416692	7.08156194
-2.9534214	5.46257765	-0.48063	4.03725163
3.01713789	6.60167029	6.54995427	3.53226365
5.7176697	3.8001381	6.43341722	3.72643846
-0.5903867	5.30428797	0.11622035	3.30782906
9.23934529	8.71741758	9.01903569	8.86138818
4.38388857	6.16166675	6.03816892	4.2066558
4.51721375	4.57310874	5.645931	3.04193075
7.59548762	6.0976047	7.29604732	6.75208959
0.63778364	4.71170388	-2.3036717	6.34470609
5.58994004	6.77623674	6.14234533	6.63819207
5.79540012	7.92244724	6.84453917	7.2192339
4.04249178	6.04613152	4.1442542	5.56931014
6.95588095	6.11701038	6.89615818	6.55461302
3.83577786	3.32229932	4.37623849	3.37258123
6.75533534	6.83187092	6.83621729	7.09601548
7.35316233	8.0779053	7.7815208	7.54193878
6.65422068	7.96810528	6.19536136	8.2680234
-1.4465118	4.90007541	-2.9534214	4.44108792
-2.9534214	4.78775027	-1.2416692	5.74670913
7.72639771	7.50777373	7.6916106	7.82683066

6.34904979	7.03395225	7.02540884	6.70885812
5.40393742	6.55807025	7.07497554	5.29365339
5.66792647	5.50997739	5.26557152	5.62795482
6.8182511	7.12753574	7.29874173	6.97264048
-2.9534214	3.77161038	-1.8572999	5.02150779
10.3894108	9.37254256	9.61891483	10.0845923
0.58157832	5.44100065	-1.5168928	6.05196925
7.68198045	7.53899965	7.70538341	7.79568553
-2.9534214	4.87883419	-1.0106329	3.75909775
4.78014106	6.41045609	5.90658003	5.72827068
5.12057224	6.27981647	5.85442384	5.27683544
7.82463215	8.04222581	7.75380627	8.025319
7.45052208	7.65677419	7.34248806	8.04490413
4.88255162	-2.9534214	-2.9534214	4.76029215
7.63959719	6.33350003	7.53302953	6.76621691
8.28875057	7.66997309	7.50530407	8.71065633
-2.9534214	2.97730562	-2.3036717	5.51057988
-2.9534214	4.25643338	-1.8572999	6.98304643
6.14602191	-2.9534214	-2.9534214	6.08132303
7.23799858	6.81029482	7.84142049	6.52643228
6.90668953	8.14727672	7.04720396	7.89086253
5.54655677	5.55084163	5.02318941	6.51572096
5.65946659	4.74142875	3.54349911	6.57997054
7.31542767	7.68381802	8.12032473	6.77022801
-2.9534214	3.49975191	-1.8572999	2.93164669
7.2498812	6.85108549	7.13651499	7.26898187
2.86062093	5.19896012	0.91975246	6.4976901
6.04051949	7.43823529	5.89237942	7.40482841
-2.9534214	3.67016639	-1.8572999	3.53226365
-2.9534214	5.69605204	-0.8115387	6.2630905
7.27944255	6.48527214	6.22965306	7.3918702
-1.7015481	3.48576994	-2.9534214	3.94800015
7.01277876	7.49255926	8.27678531	6.51930024
-2.9534214	5.48207701	-0.8115387	5.07112348
7.71133418	5.93475399	5.95691628	7.98446968
8.23624426	8.09152977	7.70284279	8.87192533
-2.9534214	4.41977033	-1.5168928	4.32044533
-2.4069576	6.48702792	0.0149924	6.07970784
0.46213879	5.46614264	-2.3036717	6.7960318
9.44407003	9.98791801	9.47625104	10.1821825
9.50270673	9.42454616	9.37759449	9.77411217
7.37606131	8.00196222	7.18392375	8.45873008
-2.4069576	4.6996403	-1.2416692	5.90648709
8.24107154	8.45596653	8.63149451	8.30443665
6.69948748	5.94628673	7.24107451	5.18367039
6.73856586	7.15319557	6.89615818	6.85953316

-1.4465118	3.51359967	-2.9534214	4.41570593
5.32910193	5.53734842	5.90127117	5.39064087
-2.9534214	4.99444171	-1.0106329	3.46456627
5.97793844	6.49925892	5.97387424	6.30239944
-2.4069576	5.16864877	-0.6366176	5.06460581
-2.9534214	3.98046661	-2.3036717	7.60449472
-1.0415346	5.6945332	-2.9534214	5.43679432
6.16048307	6.54211354	7.84695379	4.66871562
5.49613854	6.61136169	5.33020982	6.54995429
7.36413674	6.8927427	6.97154773	7.57269479
6.36890364	7.36946425	8.0144858	6.01863778
2.35699608	4.81036498	4.0767855	3.59692625
6.76798251	8.15364702	6.85464561	8.34936811
-2.9534214	3.14682342	-2.3036717	5.5081802
6.8006633	6.63451728	7.05200293	6.70467246
-1.7015481	5.24741496	-0.2116394	5.36964455
5.83650656	4.69660858	8.48363437	2.61223772
-1.7015481	5.33775507	-0.6366176	6.38841927
0.46213879	5.86219949	-2.9534214	7.49544819
-2.9534214	4.94420061	-1.5168928	6.86047413
7.31596606	6.62498069	6.41615042	7.40418326
7.66179181	7.76818484	7.82375703	7.52720045
7.08720529	8.46735123	7.30946934	8.50399286
7.14833848	6.48175416	6.40619009	7.52245221
9.99488498	10.8129877	10.1459628	10.6252707
7.93809483	6.48965759	8.66659858	6.07485139
8.33941285	8.76088016	8.69269735	8.63348996
5.62164226	6.83187092	5.80789673	7.00958613
7.32775998	8.14616597	8.60507058	7.1134865
0.39849386	5.91402091	1.98121746	3.62377553
5.52808817	4.92360381	5.1950111	5.01137656
5.81838147	-2.9534214	7.29064334	-0.7767262
-2.9534214	5.92959859	-0.6366176	5.28246323
6.55827969	7.34062253	7.04880539	6.73166466
7.69155496	9.85027007	8.22247888	9.56548373
6.31820979	5.05487841	6.67964842	5.11278535
-0.4667031	3.39890703	-2.9534214	10.2699624
-2.4069576	5.77721766	-2.9534214	3.09406445
-0.1488474	7.25717088	2.00780786	4.17069204
-1.7015481	5.11515722	-0.0938779	4.77234132
0.52309424	4.85727556	-1.2416692	5.61463147
-2.9534214	4.14519343	-1.8572999	5.34833813
8.08588903	7.6956345	7.46028575	8.55603328
7.4187933	7.12076614	7.05917158	7.38926453
7.56727472	7.77574603	7.94007016	7.64601472
5.65436675	6.76614977	7.28385979	5.46676289

2.08108509	5.74236008	3.28546824	4.14620829
6.42288351	6.39931099	7.0702525	5.5898811
-2.9534214	4.91580357	-1.5168928	6.24013781
3.04857689	4.46323192	0.91975246	6.14449121
8.14526031	7.69031001	7.5708598	8.20866726
-2.9534214	3.20771789	-2.3036717	4.45111698
3.05890614	5.30029914	3.18419106	5.71584614
5.46540789	6.1572628	5.83969778	5.58533506
-1.4465118	3.60050843	-2.9534214	4.67300676
-2.9534214	5.62287949	-1.0106329	5.41902078
-2.9534214	5.58731014	-0.6366176	2.98784227
10.5024601	11.2112239	10.7113171	10.97961
-2.9534214	5.31025063	-1.2416692	5.8220624
6.39970592	6.49315638	5.73417066	7.00873759
4.87964103	-2.9534214	-2.9534214	5.35903067
-2.9534214	3.27430282	-2.3036717	4.66441169
-2.0115616	3.3763488	-1.5168928	4.26467214
-2.9534214	5.49783731	-1.2416692	6.64476122
-1.4465118	3.64522363	-2.9534214	4.72352881
-2.9534214	4.43440349	-1.5168928	3.00155569
-2.0115616	4.09541959	-1.2416692	4.81568012
-2.9534214	5.85133543	-0.6366176	3.91214675
1.18228212	3.58091222	-1.0106329	5.28246323
-1.0415346	6.61377441	-2.9534214	5.92819021
4.9727192	4.77917751	8.1876508	2.07737831
5.86621802	0.864048	7.21063764	-1.8332537
0.26210614	6.66430719	-2.9534214	7.62789296
-1.4465118	7.41720949	0.91975246	6.50853563
6.73293244	-2.9534214	7.77911089	-0.3022547
7.45737071	5.93346686	7.7424707	5.95662898
8.31699609	8.40301234	8.46845817	8.21419878
7.90053887	8.20974826	7.83864586	8.49068406
3.46057504	2.37912504	5.79077273	0.78254314
5.50938007	-2.9534214	-2.9534214	5.48151691
6.85798134	6.09069284	6.11049505	6.70781284
-2.0115616	4.41608873	-2.9534214	4.03725163
-2.9534214	5.02618456	-1.2416692	3.39353517
0.52309424	4.35586839	-0.8115387	4.99089813
-2.4069576	3.60050843	-1.8572999	4.69004478
-2.9534214	3.49975191	-2.3036717	5.64334479
5.04724256	6.94860348	6.63130036	5.68430846
3.30438598	2.8078386	0.11622035	5.73854334
6.36474652	4.6996403	4.13221953	6.76621691
3.9731877	5.99768864	3.70658451	6.72237824
4.79566503	2.13223724	5.91889192	0.42367165
4.75812299	6.5242694	5.09625162	6.55925675

-2.9534214	5.97784668	-1.0106329	6.31068523
-2.9534214	5.05250893	-1.5168928	4.99433145
-2.9534214	5.82658798	-1.0106329	5.56007248
-2.9534214	5.98407666	-1.0106329	5.86207611
6.46815067	7.74672695	8.97043423	5.48151691
3.40497957	7.36851209	4.27040526	6.22414331
-2.9534214	5.10832914	-1.5168928	5.7649149
-2.9534214	5.11060876	-1.2416692	3.29674874
6.91453155	6.71012623	6.66510456	6.86704378
7.48540224	8.17667639	8.11115687	7.764356
6.07782139	5.91271515	5.87442982	5.96715064
6.10548967	7.10255699	5.7637247	7.33139478
7.03714617	7.00299541	7.48592895	6.80290084
-1.7015481	5.06196358	5.0426087	-1.8332537
-2.9534214	4.75022808	-1.5168928	3.28558265
-1.4465118	4.61866789	-1.2416692	6.22414331
6.6945274	5.62607	6.14234533	6.0437083
-0.8749787	5.8982732	-2.9534214	6.26735377
6.61450713	5.30826581	7.11985164	4.62951001
5.53735202	4.97204947	5.18047523	5.16246299
-0.0567579	5.23910409	-0.48063	4.79614191
-2.9534214	5.07603037	-1.5168928	4.7110632
-2.9534214	5.92053206	-1.2416692	5.93892055
7.8325556	5.64034062	8.09225628	5.24836237
5.77831138	8.13389088	5.76567361	8.04697209
7.74930021	8.24882131	7.57974904	8.61910633
7.45785865	6.48439345	7.62768428	6.55809722
7.18239588	7.34450157	7.60554882	7.14394724
6.02611431	7.33478434	6.15726849	7.46174175
8.1431381	7.41167373	7.68390184	8.07033971
7.44117521	2.39417415	7.48533774	2.96001808
7.48060791	8.17177238	7.60500467	8.02405982
9.68443064	8.77621878	8.90009515	9.55781765
-2.9534214	6.3393434	-0.8115387	5.60343391
-2.9534214	6.36152629	-0.8115387	5.70331368
5.23430904	-2.4449602	3.3902412	-0.3022547
-1.7015481	5.77002479	0.0149924	4.92406282
-2.9534214	4.80192595	-1.8572999	5.99483892
-2.9534214	4.97705564	-1.5168928	3.35131846
-2.9534214	4.3787466	-1.8572999	3.10680874
-1.2298595	5.0334117	-2.9534214	5.76692366
5.66961251	3.10169792	7.44820158	0.9043414
-2.9534214	5.89166058	-0.8115387	3.00155569
10.2211249	10.3379152	10.4869405	10.2439683
-2.0115616	6.69956454	-2.9534214	4.76833611
-2.9534214	4.84637421	-1.8572999	5.09690361

5.11810471	-2.9534214	-2.9534214	5.14093919
-2.9534214	6.24807085	-1.2416692	6.21239855
-2.9534214	6.36248307	-1.0106329	6.83101492
-0.5903867	4.62506046	-2.9534214	6.62163715
-0.8749787	3.48576994	-1.5168928	5.54844161
7.72030982	7.39212955	8.16457163	7.15088624
3.24229507	3.65149987	0.3831957	6.30101384
7.65584985	5.6945332	7.57753186	5.68430846
0.63778364	7.28720831	1.86966605	5.45185642
-2.9534214	4.88949389	-1.8572999	6.06018317
6.60131705	5.96530542	5.80031114	7.020572
9.10124805	8.83098985	8.4437175	9.49133663
3.92318008	1.96089042	4.29220212	2.02462691
-2.9534214	4.02948146	-2.3036717	6.13363455
-2.9534214	4.91319409	-1.8572999	5.98451799
6.28998825	6.26146868	7.08593619	5.72207179
9.96867453	10.2828203	9.91006805	10.3522047
-2.9534214	5.08996134	-1.8572999	5.18367039
-1.4465118	5.08301267	-2.9534214	5.58077464
0.39849386	7.26589006	2.45696755	5.79278609
6.7960397	6.81379674	7.83168585	5.72620734
-2.4069576	3.75421899	-2.3036717	6.06508916
-2.9534214	4.62186772	-1.8572999	3.56033181
5.39784727	4.74436782	5.98395416	4.46603105
8.15582475	8.90771511	8.65718072	8.57765161
6.84162215	6.85312909	6.76979876	7.1408525
-1.7015481	6.81029482	-2.9534214	5.47415876
10.0434831	10.6187129	10.2173014	10.459886
1.45678855	3.29850066	0.67627567	4.56170256
5.83350148	6.83669861	7.47822431	5.13473009
-2.9534214	3.98046661	-2.3036717	4.92765874
6.71426619	6.89605032	7.69058514	5.87889084
6.37201363	6.68127694	7.37412939	5.90648709
6.18780155	5.18604745	4.63266077	6.66105439
6.77661358	6.85448989	7.04560075	6.7940632
7.32669178	6.49577491	7.04880539	6.74293414
-2.9534214	5.21388028	4.9289408	-2.9534214
8.54490405	6.80326539	7.21634915	8.13242287
-2.9534214	5.66226253	-1.2416692	3.31882493
0.69188115	5.39123914	2.22734526	3.54168051
8.40768002	8.39672137	8.96068448	8.00928751
10.1299934	11.2549023	10.3640904	11.0421633
-2.0115616	5.32406839	-2.9534214	4.80398897
6.01951864	6.90723984	7.68596154	5.45435155
7.50536624	6.22721275	6.24649802	7.46545732
7.58212169	7.19783484	6.99914383	7.77437579

-1.0415346	4.47389578	-0.2116394	4.19476721
6.89448556	5.99522327	5.36661995	7.73387222
7.19298593	7.26844453	7.16632753	7.28378824
5.81228882	7.86588144	5.46585463	8.16553924
0.74402323	6.19963841	0.29957556	7.50510763
6.78207931	6.26249414	7.01397669	6.24591031
3.92318008	5.37805286	4.90791795	4.29282823
8.55929988	10.084364	8.32185473	10.3306372
0.02980441	6.31285979	1.71688244	5.10962295
5.32696672	7.20211684	7.09449009	5.39584256
-2.9534214	6.04969999	-1.2416692	5.34833813
-2.0115616	5.76134574	-2.9534214	4.91684399
6.64224926	7.22333827	7.2777272	6.57309894
-1.0415346	5.3260316	-2.3036717	5.87517113
4.76760048	-2.9534214	-2.9534214	5.12536604
6.40982892	3.48576994	5.78693963	4.40542677
-2.9534214	4.28497292	-2.3036717	6.05196925
-2.9534214	5.54242317	-1.5168928	4.36886174
5.87355125	4.53287918	5.00019818	5.33214925
0.74402323	6.22616188	8.69500024	-1.4884482
7.3293608	7.02612415	6.83436144	7.51888074
-1.2298595	5.65759294	-0.6366176	4.16460995
6.31928429	6.44699662	6.25345911	6.47820551
-2.9534214	5.23074506	-1.8572999	5.8220624
-2.9534214	4.89214656	-1.8572999	3.444626
7.38840015	8.09152977	7.51402565	8.13008381
-2.9534214	4.58954551	-2.3036717	4.67300676
-2.4069576	3.29850066	-2.3036717	4.3529028
0.58157832	5.16645911	1.95412777	3.52278492
9.30441211	7.97406442	8.87931974	8.55458048
5.62859358	-2.9534214	6.71336118	-1.4884482
-2.4069576	5.02376745	-1.5168928	4.79220231
0.02980441	4.50542242	-1.8572999	6.19758185
7.99837039	8.2498558	8.10808792	8.29472314
6.14723256	6.72061116	6.88275228	5.96014474
0.46213879	4.91057988	-0.8115387	5.84125502
7.81094702	7.68573047	7.62821995	8.02741521
10.2736201	10.4355347	10.4545354	10.3973165
-2.9534214	3.33014576	-2.9534214	7.51589775
7.64731865	7.26691239	7.83493803	7.24612346
8.02232066	8.85905618	8.64452718	8.26518394
-2.9534214	2.88444049	-2.9534214	6.75006006
5.85143877	7.00176778	6.07321234	6.97958611
-0.7256766	5.90485565	-2.9534214	7.0002246
-2.9534214	4.16287554	-2.3036717	3.70982732
6.52792873	5.99027985	6.45410996	6.26593408

7.31758001	4.85998807	6.98666555	5.41646374
-2.9534214	4.34429178	-2.3036717	5.63236873
-2.9534214	5.05250893	-1.8572999	3.82227867
4.30364111	-0.078038	5.42697596	-1.4884482
-2.9534214	4.45607864	-2.3036717	6.32711539
3.83577786	-2.9534214	-2.9534214	4.17069204
6.91808207	7.11623535	6.95797537	7.24612346
6.71997283	5.76713757	6.00390513	6.67716561
3.19624914	3.91936229	3.0240799	4.01713976
10.1465083	9.73623703	10.1046975	9.81667152
3.04857689	4.92878064	0.91975246	6.94540582
3.52904442	4.93651123	3.1606858	5.56700626
4.73576368	6.12717926	6.79578083	4.2760006
9.4711507	9.11338215	8.90695433	9.8152163
-2.0115616	4.26464531	-2.9534214	4.97012482
6.9730506	6.36821045	6.85097873	6.66321297
4.12837033	5.41725555	4.44108789	5.32671233
4.35484045	4.35201983	4.28134486	4.64706639
6.95588095	6.71537822	6.16467258	7.52007222
6.09172186	2.60288549	8.02306537	1.1208487
-2.9534214	5.71565281	-1.5168928	3.7181568
6.62844487	6.60733151	6.54995427	6.8548191
-2.9534214	6.34419487	6.13633258	-2.9534214
-1.4465118	5.33190524	-1.5168928	6.58794626
-2.4069576	3.53412523	-2.3036717	4.30393859
6.62062146	6.5370377	6.88633939	6.27866128
-2.4069576	5.26184478	-1.8572999	6.2758427
-2.4069576	4.86809514	-2.9534214	4.83495726
3.91182789	5.94756247	4.35568384	5.71376495
-1.7015481	4.9053372	-1.5168928	4.03057878
5.09067961	4.29703342	5.00019818	4.3529028
5.5934971	-2.9534214	5.81167462	-1.8332537
7.10101607	7.40889786	8.07467774	6.4634174
-1.7015481	4.54641406	-1.2416692	4.54775007
-2.9534214	4.55982314	-2.3036717	5.82399319
5.27253304	5.3666536	4.55365123	6.2758427
-2.9534214	4.44888971	-2.3036717	4.01037301
-2.9534214	4.56315603	-2.3036717	5.70959352
-2.9534214	6.36152629	-1.5168928	6.64038511
-2.9534214	6.57550367	-1.0106329	3.9829841
2.53031844	4.40498723	1.02763635	5.85642736
-1.0415346	4.41239771	-2.9534214	6.6260704
1.10463976	5.63718155	-0.0938779	6.67181517
6.44667828	7.79960827	6.25900391	8.13592437
-2.9534214	4.77055351	-2.3036717	7.01382137
1.83886594	5.6884418	2.45696755	4.97012482

5.38968688	4.41239771	4.1972014	5.60343391
4.69660828	5.49434987	8.15121585	2.0512437
-2.9534214	5.33580776	-1.8572999	4.03725163
3.85368704	4.1976026	4.36599777	3.87537958
4.85910083	6.65183896	5.78309633	5.89551185
5.49993425	6.66197759	7.13726786	5.04487427
-0.1488474	4.21465761	0.46223352	3.82998523
10.6354944	11.2539359	11.2951466	10.6533001
-2.4069576	5.35131312	-2.9534214	5.33756575
4.45990662	3.22465444	4.5936968	3.06823308
7.48060791	6.69880717	7.5574224	6.66213409
8.16571514	8.79494407	8.7820606	8.29958807
7.04496154	6.15505578	6.39741804	6.83580728
9.19803078	9.37325474	9.30842133	9.32150093
7.6807269	6.72582519	7.8565863	6.59135098
8.99699929	8.28029749	8.75509245	8.64254421
7.75129196	8.20442207	7.32276765	8.68751255
5.74990385	8.82214942	5.91362826	8.80238387
-2.9534214	6.33154696	-1.2416692	3.16889429
7.76436902	6.14841438	6.1939147	7.77137715
6.84759243	5.51686898	8.05648441	4.33676534
6.39767277	3.92455433	3.83835276	6.51572096
7.92791685	7.44683123	8.7165703	6.71407317
7.54540266	7.90843046	7.81766428	7.69536621
5.15468173	7.78505292	6.38606074	6.68568525
-2.4069576	4.87347466	-2.9534214	4.61173735
-1.7015481	5.53055419	-0.48063	3.65881453
5.7338768	6.46759581	5.53604327	6.70467246
5.60763831	5.02376745	6.14534234	4.63391918
-2.9534214	5.71565281	-1.8572999	6.25022457
6.4969255	6.79336628	5.56989725	7.77537395
6.05219949	3.31440997	5.72418322	3.65881453
5.73064991	5.8252006	7.51112431	4.18277985
-2.4069576	5.32995001	-2.9534214	4.49540449
-1.4465118	3.71879433	7.40634139	-2.9534214
5.30326854	4.88949389	4.54459922	5.69278614
6.16287923	5.86084595	5.7201688	6.35542548
5.47700873	5.17083512	4.86493027	5.83360858
8.25489577	8.48525017	8.31953488	8.49250614
4.5759319	4.97955223	3.1606858	6.53588716
-1.7015481	7.56333629	-1.8572999	5.88999276
-2.9534214	6.94860348	-0.8115387	2.94590212
-2.4069576	2.91606439	-2.3036717	2.94590212
5.84399199	-2.9534214	-2.9534214	6.03373231
8.01106034	7.2831719	7.99592737	7.40159975
7.58435796	9.25187523	8.47829628	8.43397538

6.32035798	6.59598675	5.64804559	7.37748044
2.91848853	6.5589052	3.43010881	6.08937197
-1.4465118	6.01726141	-1.2416692	6.01357097
9.85017802	10.1456728	10.1312051	9.96238598
-2.4069576	6.34613089	-1.0106329	3.70982732
8.55131994	8.23216774	8.99618515	7.88486322
7.32401782	5.45900381	7.45727418	5.43173847
6.22726085	7.03874851	8.15158852	5.21637896
5.77987329	2.42380945	8.52794096	-0.0551215
4.71631881	6.67820634	4.9008417	6.60039113
5.22974479	6.66275454	7.21919645	4.77633546
6.9209162	7.38131352	6.54995427	7.83307567
-0.2472184	6.30292635	-0.3398746	6.56734749
5.4595723	2.48131403	6.48873847	1.58888154
4.21866576	5.20110105	6.39490191	3.13196448
7.74969878	8.08429873	7.21420999	8.7035948
7.53711439	7.66842653	7.83586588	7.45428178
5.77518247	8.03053518	6.88364989	7.01466693
-2.9534214	3.71280473	-2.9534214	3.75100198
2.34015605	4.58954551	2.88756311	4.11500746
5.47893316	5.99768864	7.13047783	4.44108792
8.91905433	8.1915584	8.87706791	8.32059587
-2.9534214	2.96727758	-2.9534214	3.01513998
-2.9534214	2.96727758	-2.9534214	6.33119386
-2.9534214	6.26761057	-2.9534214	2.90270615
-2.9534214	4.05811045	-2.9534214	7.443019
-2.9534214	6.32370816	-2.9534214	3.04193075
-2.9534214	3.23304873	-2.9534214	6.38972346
-2.9534214	4.57641111	-2.9534214	7.71370666
-2.9534214	3.48576994	-2.9534214	6.47943104
-2.9534214	6.51138708	-2.9534214	3.5510363
4.88255162	-2.9534214	7.8565863	-2.9534214
-2.9534214	6.12153873	-2.9534214	3.19299923
-2.9534214	6.65886559	-2.9534214	3.80674094
-2.9534214	3.83364304	-2.9534214	6.54995429
-2.9534214	3.8001381	-2.9534214	6.4976901
-2.9534214	3.9089218	-2.9534214	6.58453349
-2.9534214	6.37106564	-2.9534214	3.73467286
-2.9534214	4.33262153	-2.9534214	6.95424749
-2.9534214	3.61342625	-2.9534214	6.2035268
-2.9534214	3.20771789	-2.9534214	5.75077476
-2.9534214	4.17599634	-2.9534214	6.72134272
-2.9534214	5.61004642	-2.9534214	3.08120658
-2.9534214	3.33014576	-2.9534214	5.79868907
-2.9534214	7.49692265	-2.9534214	5.01137656
-2.9534214	4.83814381	-2.9534214	7.32186169

-2.9534214	4.48448139	-2.9534214	6.93204087
6.18426747	-2.9534214	8.37292882	-2.2872455
-2.9534214	4.56315603	-2.9534214	6.99594919
-2.9534214	4.17163599	-2.9534214	6.59361633
-2.9534214	3.29047973	-2.9534214	5.67149778
-2.9534214	4.88949389	-2.9534214	7.31638579
-2.4069576	6.26761057	-2.9534214	4.73177979
-2.9534214	4.51921611	-2.9534214	6.90128062
-2.9534214	3.69468524	-2.9534214	6.04701836
-2.9534214	4.39005107	-2.9534214	6.75411627
-2.9534214	4.65035121	-2.9534214	7.01804421
-2.4069576	3.16448568	-2.9534214	6.51691504
-2.9534214	3.88781139	-2.9534214	6.16138832
-2.9534214	5.95519333	-2.9534214	3.68454639
-2.9534214	6.78554048	-2.9534214	4.51465971
-2.9534214	6.34225624	-2.9534214	4.07665333
-2.9534214	6.37391519	-2.9534214	4.12756888
-2.9534214	3.47165113	-2.9534214	5.66073462
-2.9534214	4.7531493	-2.9534214	6.95336576
-2.9534214	6.03896797	-1.8572999	7.32322742
-2.9534214	3.01673495	-2.9534214	5.12536604
-2.9534214	3.74837496	-2.9534214	5.85453952
-2.9534214	3.48576994	-2.9534214	5.53671621
-2.9534214	4.08618237	-2.9534214	6.15219626
-2.9534214	7.80454495	-2.9534214	5.75482896
-2.9534214	3.49975191	-2.9534214	5.51775506
-2.9534214	6.09300048	-2.9534214	4.08955166
-2.9534214	4.4380387	-2.9534214	6.40528241
5.43003494	-2.9534214	7.41008418	-2.9534214
-2.9534214	3.5066924	-2.9534214	5.41646374
-2.9534214	3.32229932	-2.9534214	5.21931602
-2.9534214	3.09250094	-2.9534214	4.97360788
-2.9534214	4.48799274	-2.9534214	6.42957931
5.25467136	-2.4449602	7.57253071	-2.9534214
-2.9534214	4.09541959	-2.9534214	6.01526189
-2.9534214	4.48096148	-2.9534214	6.40914607
-2.9534214	5.01162075	-2.3036717	5.94957167
5.69632454	-2.9534214	3.83835276	-2.9534214
-2.9534214	4.86269549	-2.9534214	3.01513998
-2.9534214	5.11060876	-2.9534214	6.98218213
-2.9534214	5.06196358	-2.3036717	5.93892055
-2.9534214	6.96884891	-1.8572999	8.30409087
-2.9534214	4.74436782	-2.9534214	6.57195048
-2.9534214	4.29703342	-2.9534214	6.10850817
-2.4069576	7.004222	-2.3036717	5.14093919
-2.9534214	4.71470408	-2.9534214	6.53234884

-2.9534214	4.15847529	-2.9534214	5.95486789
-2.9534214	4.61546095	-2.9534214	6.41812124
-2.0115616	6.13503914	-2.9534214	5.6123989
-2.9534214	6.73767268	-2.9534214	4.96663333
-2.9534214	5.37805286	-2.9534214	3.62377553
-2.9534214	4.98949561	-2.9534214	3.2400335
-2.9534214	4.42710546	-2.9534214	6.16900388
-2.9534214	4.98949561	-2.9534214	6.74089168
5.37735909	-2.9534214	6.47093097	-2.2872455
-2.9534214	5.63084257	-2.9534214	3.91214675
-2.4069576	6.57385239	-2.9534214	5.49126977
-2.9534214	4.29302445	-2.9534214	5.99655192
-2.9534214	6.3168141	-2.3036717	7.61233637
-2.9534214	3.53412523	-2.9534214	5.16855413
-2.9534214	4.1976026	-2.9534214	5.86207611
-2.9534214	3.63258865	-2.9534214	5.24836237
-2.9534214	5.17301815	-2.9534214	6.83485008
-2.9534214	5.63559941	-2.9534214	3.99674355
-2.9534214	4.11371847	-2.9534214	5.74263202
-2.9534214	4.85727556	-2.9534214	3.2284179
-2.9534214	4.57641111	-2.9534214	6.2079695
-2.0115616	7.09854292	-1.5168928	8.44028266
5.0289695	-2.9534214	6.66510456	-2.9534214
-2.9534214	4.49847579	-2.9534214	6.10374787
-2.9534214	4.31691287	-2.9534214	5.91556976
-2.9534214	5.94500985	-2.3036717	7.00193122
-2.9534214	6.81798785	-2.9534214	5.22224711
-2.9534214	5.25567824	-2.9534214	6.86985021
-2.9534214	4.16287554	-2.9534214	5.75077476
-2.9534214	3.39142674	-2.9534214	4.93482379
-2.9534214	3.45739279	-2.9534214	5.00117368
-2.9534214	3.48576994	-2.9534214	5.01476155
5.63032619	-2.9534214	7.23616323	-2.9534214
-2.9534214	4.66903251	-2.9534214	6.23579323
-2.9534214	3.66397101	-2.9534214	5.18065978
-2.9534214	6.26454289	-1.8572999	6.83580728
-2.9534214	3.61984204	-2.9534214	5.12223114
-2.9534214	4.95184922	-2.9534214	3.444626
-2.9534214	5.12195314	-2.3036717	5.55310522
-2.9534214	5.30826581	-2.3036717	5.88445248
-2.9534214	3.45739279	-2.9534214	4.89863728
-2.9534214	4.41977033	-2.9534214	5.89551185
-2.9534214	4.80755745	-2.9534214	3.3405684
-2.9534214	6.28990976	-1.5168928	5.81042307
-2.0115616	6.98260464	-1.8572999	5.4593289
-2.9534214	3.78879461	-2.9534214	5.20457055

-2.9534214	4.54977803	-2.9534214	3.11944144
-2.9534214	7.1870739	-2.9534214	5.7568518
-2.9534214	3.63891997	-2.9534214	5.03490637
-2.9534214	3.64522363	-2.9534214	5.04155929
-2.9534214	3.6069818	-2.9534214	4.99775662
-2.9534214	5.80704212	-2.9534214	7.25973928
-2.9534214	4.92100841	-2.9534214	6.35675982
-2.9534214	7.04352889	-2.9534214	5.62352736
-2.9534214	3.63891997	-2.9534214	5.01137656
4.85317808	-2.9534214	6.27002997	-2.9534214
-2.9534214	4.16287554	-2.9534214	5.54610414
-2.9534214	7.50517691	-2.3036717	5.61463147
-2.9534214	2.93676833	-2.9534214	4.23014303
-2.9534214	4.79059657	-2.9534214	6.16900388
-2.9534214	4.24817444	-2.9534214	5.60568039
-2.9534214	4.99690842	-2.9534214	6.37399488
-2.9534214	4.52606382	-2.9534214	5.88260098
-2.9534214	4.21465761	-2.9534214	5.5577538
-2.4069576	5.22233731	-2.9534214	4.95610789
-2.9534214	4.67520624	-2.9534214	6.01357097
-2.9534214	4.40498723	-2.9534214	3.08120658
-2.9534214	4.14519343	-2.9534214	5.4593289
-2.9534214	5.24741496	-2.3036717	5.49854138
-2.9534214	5.4803152	-2.9534214	4.17069204
-2.9534214	3.67016639	-2.9534214	4.93124571
-2.9534214	5.46436125	-2.9534214	6.77821691
-2.9534214	6.05681058	-2.9534214	7.37945115
-2.9534214	5.84450361	-2.9534214	7.16314043
-2.9534214	6.55137313	-1.8572999	6.95336576
-2.9534214	5.66846524	-2.9534214	4.40025959
-2.9534214	3.81698783	-2.9534214	5.05477378
-2.9534214	6.26863168	-2.9534214	5.00458266
-2.9534214	5.10146859	-2.9534214	6.37531216
-1.4465118	6.63689161	-2.9534214	6.93561698
-2.9534214	6.42059735	-1.8572999	6.66321297
-1.7015481	5.74822848	-2.9534214	6.28428197
-2.9534214	4.83814381	-2.9534214	6.08615778
-2.9534214	5.24948522	-2.9534214	6.50372546
-2.9534214	4.37116077	-2.9534214	5.60343391
-2.9534214	5.86219949	-2.9534214	7.1205732
-2.9534214	6.37201612	-1.5168928	5.61686059
-2.9534214	4.11371847	-2.9534214	5.32398617
-2.9534214	6.34613089	-2.9534214	7.6028088
-2.9534214	4.14519343	-2.9534214	5.3402664
-2.9534214	6.44699662	-2.9534214	7.68265377
-2.9534214	7.10941238	-1.2416692	6.67074269

-2.9534214	6.6000487	-2.9534214	5.40876529
-2.9534214	4.52264403	-2.9534214	5.70541
-2.9534214	4.20188529	-2.9534214	5.36699839
-2.9534214	5.83763929	-2.9534214	4.67300676
-2.9534214	3.84464064	-2.9534214	4.98054886
-2.9534214	4.04863044	-2.9534214	5.1926647
-2.9534214	5.86760099	-2.9534214	7.0571462
-2.9534214	5.92959859	-2.3036717	6.42195072
-2.9534214	5.4482289	-2.9534214	6.59361633
-2.9534214	4.34816098	-2.9534214	5.46181116
-2.9534214	3.42111796	-2.9534214	4.48567964
-2.9534214	5.08533261	-2.9534214	6.21681405
-2.0115616	5.17737434	-2.9534214	7.28238464
-2.9534214	4.65660523	-2.3036717	3.91214675
-2.9534214	5.87163889	-2.9534214	4.77234132
6.48930816	-0.2545545	7.75331527	-2.9534214
-2.9534214	5.51514917	-2.9534214	6.62828193
-2.9534214	5.84860658	-2.9534214	4.76029215
-2.9534214	4.59931844	-2.9534214	5.68430846
-2.9534214	5.99891975	-2.9534214	4.92406282
-2.9534214	4.29302445	-2.9534214	5.35636496
-2.9534214	5.10604592	-2.9534214	6.19011605
5.26139543	-2.9534214	6.35920742	-2.9534214
-2.9534214	5.993989	-2.9534214	4.92765874
6.61363153	-2.9534214	7.37923006	-2.2872455
-2.9534214	4.74730093	-2.9534214	3.68454639
-2.9534214	3.13791053	-2.9534214	4.12130184
-2.9534214	8.18264763	-2.3036717	6.84914181
-2.9534214	5.20750481	-2.9534214	6.2758427
-2.4069576	6.45688511	-2.9534214	5.99140681
-2.9534214	6.32469035	-1.8572999	5.91194355
-2.9534214	5.72163063	-2.9534214	6.75917054
-2.9534214	4.86269549	-2.9534214	5.88260098
-2.9534214	5.86219949	-2.3036717	3.49396925
-2.4069576	3.61984204	-2.9534214	5.78685885
-2.9534214	7.76312194	-1.8572999	5.98279063
-2.9534214	6.66508289	-1.8572999	6.47943104
-2.9534214	5.86625251	-2.9534214	4.92045792
-2.9534214	6.36248307	-2.3036717	4.45111698
-2.9534214	4.07221469	-2.9534214	3.14437975
-2.9534214	5.31421208	-2.3036717	4.84259657
-2.9534214	4.58954551	-2.9534214	5.51057988
-2.9534214	5.29629924	-2.9534214	6.23434213
-2.9534214	4.97705564	-2.9534214	4.05708699
-2.9534214	4.55982314	-2.9534214	5.47169768
-2.9534214	5.87029418	-2.3036717	5.81236949

-2.9534214	6.16934146	-2.9534214	5.25981912
-2.9534214	4.88683634	-2.9534214	5.80261102
-2.9534214	4.85183518	-2.9534214	5.76692366
-2.9534214	3.20771789	-2.9534214	4.03057878
-2.9534214	6.26249414	-1.8572999	5.59214876
-2.9534214	5.35516356	-2.9534214	4.46107681
-2.9534214	4.34816098	-2.9534214	3.45463059
-2.9534214	3.01673495	-2.9534214	3.80674094
-2.9534214	7.04829347	-2.9534214	7.96360293
-2.9534214	7.36565184	-2.9534214	6.50733459
-2.9534214	6.12040798	-2.9534214	5.26266914
-2.9534214	5.26184478	-2.9534214	6.1367448
-2.9534214	4.08618237	-2.9534214	4.91684399
-2.9534214	6.41138098	-2.9534214	5.56700626
5.39988018	-2.9534214	3.13679123	-2.2872455
-2.9534214	4.07688562	-2.9534214	3.25155631
-2.9534214	3.5066924	-2.9534214	4.2760006
-2.9534214	5.67464139	-2.9534214	4.85398021
-2.9534214	5.08996134	-2.9534214	5.92279496
-2.9534214	5.65603304	-2.9534214	4.84259657
-2.9534214	4.94675465	-2.9534214	5.76088898
-2.4069576	7.24009349	-2.9534214	8.26020139
-2.9534214	5.74529726	-2.9534214	4.97012482
5.07295187	-2.9534214	4.29760027	-2.9534214
-2.9534214	4.78204076	-2.9534214	5.55543138
-2.9534214	4.58299326	-2.9534214	3.81453072
-2.4069576	4.84363596	-2.9534214	5.32943335
-2.9534214	3.98544371	-2.9534214	4.71938556
-2.9534214	5.79007514	-2.9534214	6.57424648
-2.9534214	4.73848367	-2.9534214	5.50095713
-2.9534214	7.19192626	-1.8572999	7.04146641
-2.9534214	4.30899393	-2.9534214	3.56033181
-2.9534214	7.35030064	-2.9534214	6.60376663
-2.9534214	6.00014981	-2.9534214	5.25410211
-2.9534214	4.98453248	-2.9534214	5.73854334
-2.9534214	6.76397907	-2.9534214	6.02368686
-2.9534214	4.04863044	-2.9534214	4.74406844
-2.9534214	4.46323192	-2.9534214	5.16855413
-2.9534214	4.76477541	-2.9534214	4.05050546
-2.9534214	5.55251944	-2.9534214	4.84259657
-1.7015481	4.25643338	-2.9534214	6.0084863
-2.9534214	4.59931844	-2.9534214	5.29921609
-2.9534214	4.05337823	-2.9534214	4.7276602
-1.7015481	7.05185661	-1.0106329	5.69489781
-2.9534214	6.84013709	-2.9534214	6.14449121
-2.9534214	5.5575412	-2.9534214	4.86151971

-2.9534214	5.52543744	-2.9534214	6.23869107
-2.9534214	6.153951	-1.8572999	5.05805857
-2.9534214	5.28826602	-2.9534214	5.98451799
-2.9534214	4.97955223	-2.9534214	4.30946185
-2.9534214	5.70362235	-2.9534214	6.3923283
-2.9534214	4.52947552	-2.9534214	3.87537958
-2.0115616	6.64635004	-2.9534214	6.97264048
-2.9534214	7.41813005	-2.9534214	6.77821691
6.95933127	-2.9534214	7.64154744	-2.9534214
-2.4069576	5.12646603	-2.9534214	6.45721067
-2.9534214	5.26594122	-2.9534214	5.89734686
-2.4069576	6.90855057	-2.9534214	6.71926944
-2.9534214	3.6824779	-2.9534214	4.23595558
-2.9534214	4.74730093	-2.9534214	4.14002187
-2.9534214	3.44299212	-2.9534214	3.97605485
-2.9534214	4.10916546	-2.9534214	4.68155092
-2.9534214	7.18491204	-2.9534214	6.58908206
-2.9534214	5.46436125	-2.9534214	4.86902002
-2.9534214	4.72366742	-2.9534214	4.12756888
6.61100155	-2.9534214	7.25293278	-2.9534214
-2.9534214	4.96953985	-2.9534214	4.38464608
-2.9534214	4.88417389	-2.9534214	5.47169768
-2.9534214	5.06196358	-2.9534214	5.65423797
-2.9534214	6.76831721	-2.3036717	6.69731821
-2.9534214	5.29429513	-2.9534214	5.88630161
-2.9534214	4.61224686	-2.9534214	5.18065978
-2.9534214	4.32478858	-2.9534214	4.87648153
-2.9534214	4.00027282	-2.9534214	3.43455156
-2.9534214	5.06666776	-2.9534214	4.50986995
-2.9534214	5.52885062	-2.9534214	6.0989718
-2.9534214	4.11371847	-2.9534214	4.63391918
-2.9534214	4.75606462	-2.9534214	5.29921609
-2.9534214	5.45542111	-2.9534214	4.91684399
-2.9534214	3.78879461	-2.9534214	3.25155631
-2.9534214	5.50651921	-2.9534214	6.04867054
-2.9534214	6.82910498	-2.9534214	6.30930757
-2.9534214	5.95265419	-2.3036717	5.35903067
-2.9534214	3.46453958	-2.9534214	3.89755213
-2.9534214	5.08068899	-2.9534214	5.59214876
-2.9534214	5.71714959	-2.3036717	4.94904802
-2.9534214	3.92972776	-2.9534214	3.43455156
-2.9534214	6.12830473	-2.9534214	6.6469443
-2.9534214	4.42710546	-2.9534214	4.89863728
-2.9534214	5.47147368	-2.9534214	5.96889681
-2.9534214	5.66691706	-2.9534214	5.18667473
-2.9534214	5.68996707	-2.9534214	6.18561795

-2.9534214	4.03429258	-2.9534214	3.56033181
-2.9534214	6.50360212	-1.8572999	5.43931561
-2.9534214	6.37486379	-2.9534214	5.91556976
-2.9534214	6.72582519	-2.3036717	6.43968841
-2.9534214	5.36283374	-2.9534214	4.91322098
-2.9534214	5.057244	-2.9534214	5.5081802
-2.9534214	4.23986795	-2.9534214	3.79890887
-2.9534214	4.96953985	-2.9534214	5.40361008
-2.9534214	7.43414559	-2.9534214	7.007039
-2.9534214	3.33014576	-2.9534214	3.65881453
-2.9534214	5.19896012	-2.9534214	4.78825193
-2.9534214	4.30899393	-2.9534214	3.89755213
-2.9534214	6.14841438	-2.9534214	6.58225382
-2.9534214	5.00182922	-2.3036717	3.56956781
-2.4069576	5.05487841	-2.9534214	6.22560471
-2.9534214	3.87713921	-2.9534214	3.474434
-2.9534214	6.05325967	-2.9534214	5.65640678
-2.9534214	5.64034062	-2.9534214	5.24548391
6.09172186	-2.9534214	6.52370732	-2.9534214
-2.9534214	5.4482289	-2.9534214	5.06133591
-2.9534214	4.1227816	-2.9534214	3.74286052
-2.9534214	4.93907891	-2.9534214	5.31302972
-2.9534214	5.69605204	-2.9534214	5.32398617
-2.9534214	5.78865218	-2.9534214	5.41902078
-2.9534214	5.12195314	-2.9534214	5.49126977
-2.9534214	5.00182922	-2.3036717	3.5132435
-2.9534214	6.15615971	-2.9534214	5.79672409
-2.9534214	4.43075909	-2.9534214	4.76431974
-2.4069576	4.9027087	-2.9534214	6.08293642
-2.9534214	4.92100841	-2.9534214	4.57092982
-2.9534214	5.97033501	-2.9534214	6.32575334
-1.2298595	5.14660115	-1.8572999	5.8936745
-2.9534214	6.21772731	-2.9534214	6.57080111
-2.9534214	5.79858358	-2.9534214	6.14294526
-2.9534214	4.21465761	-2.9534214	4.50024244
-2.9534214	5.96025823	-2.3036717	4.59826269
-2.9534214	3.78308925	-2.9534214	3.474434
-2.9534214	4.02465423	-2.9534214	3.7181568
-2.9534214	3.21621102	-2.9534214	2.91724899
-2.9534214	4.97204947	-2.9534214	5.26551355
-2.9534214	5.64349279	-2.9534214	5.95310464
-2.9534214	5.12421135	-2.9534214	5.41133601
-2.9534214	6.12153873	-2.9534214	6.41812124
-2.9534214	3.83915232	-2.9534214	3.56033181
-2.9534214	3.68859448	-2.9534214	3.41418912
-2.9534214	3.53412523	-2.9534214	3.26298783

-2.9534214	4.50542242	-2.9534214	4.74406844
-2.9534214	5.47147368	-2.9534214	5.74263202
-2.9534214	7.42730355	-2.9534214	7.1715052
-2.9534214	4.87078741	-2.9534214	4.61173735
-2.9534214	4.43075909	-2.9534214	4.64269729
-2.9534214	3.27430282	-2.9534214	3.02859755
5.85292353	-2.9534214	5.62675891	-2.9534214
-2.4069576	6.98571274	-2.3036717	7.10874262
-2.9534214	4.50888322	-2.9534214	4.67300676
-2.9534214	4.66593571	-2.9534214	4.46603105
-2.9534214	4.55648254	-2.9534214	4.70688394
-2.9534214	4.08618237	-2.9534214	3.89019908
-2.9534214	5.12646603	-2.9534214	4.94195344
-2.9534214	5.23910409	-2.9534214	5.40876529
-2.9534214	5.71565281	-2.9534214	5.53435965
-2.9534214	6.27067174	-2.9534214	6.44847615
-2.9534214	5.54242317	-2.9534214	5.37492235
-2.9534214	4.83538987	-2.9534214	4.95610789
-2.9534214	5.83901477	-2.9534214	5.67791734
-2.9534214	3.5066924	-2.9534214	3.5132435
6.13629998	-2.0697503	6.98164386	-2.9534214
-2.9534214	4.24817444	-2.9534214	4.29839411
-2.9534214	5.48207701	-2.9534214	5.61686059
-2.9534214	7.16201481	-1.8572999	6.07647202
-2.9534214	3.39142674	-2.9534214	3.2400335
-2.9534214	3.17323639	-2.9534214	3.14437975
-2.9534214	4.8408925	-2.9534214	4.88019786
-1.7015481	5.73351248	-1.5168928	5.49369771
-2.9534214	4.20615531	-2.9534214	4.07665333
-2.9534214	5.50824933	-2.9534214	5.57848902
-2.9534214	3.0645529	-2.9534214	2.93164669
-2.9534214	5.98780177	-2.9534214	6.07647202
-2.9534214	6.51483363	-2.9534214	6.60376663
-2.9534214	5.99151729	-2.9534214	6.05196925
-2.9534214	6.6265745	-2.9534214	6.70990264
-2.9534214	4.01495106	-2.9534214	3.97605485
-2.9534214	5.41171997	-2.9534214	5.4215733
-2.9534214	6.07326691	-2.9534214	6.0989718
-2.9534214	5.4482289	-2.9534214	5.44685317
-2.4069576	6.74942367	-2.3036717	6.64585317
-2.9534214	6.08490755	-2.9534214	6.1021576
-2.9534214	6.05207408	-2.9534214	5.95838793
-2.9534214	4.17599634	-2.9534214	4.12756888
-2.9534214	6.82355712	-2.9534214	6.85008958
-1.4465118	5.25155252	-0.6366176	4.27034749
-2.9534214	5.42826341	-2.9534214	5.34833813

-2.9534214	6.1181438	-2.9534214	6.08293642
4.63580752	5.057244	7.64842939	2.15305622
-2.9534214	3.73069946	-2.9534214	3.65013424
-2.9534214	5.20110105	-2.9534214	5.23391229
-2.9534214	4.87883419	-2.9534214	4.81179359
