# Supplementary material for: A novel post-developmental role of the Hox genes underlies normal adult behavior
Source: Proc Natl Acad Sci U S A. 2022 Dec 1;119(49):e2209531119. doi: 10.1073/pnas.2209531119 (PMC9894213; doi:10.1073/pnas.2209531119)
Supplement: Supplementary file 3 — Dataset S02 (PDF) [file pnas.2209531119.sd02.pdf]

FlyBase ID **FBgn0029950**
Annotation S **CG9657**
Symbol **CG9657**

	Adult Male			Adult Female		
Tissue	FPKM	SD	Enrichment	FPKM	SD	Enrichment
Whole body	6.2	1.27	1	0.71	0.18	-1
Head	27.75	3.17	4.48	14.89	2.22	7.44
Eye	6.38	0.71	1.03	4.91	0.28	2.46
Brain / CNS	79.35	4.09	12.8	50.92	2.85	25.46
Thoracicoabc	113.3	20.34	18.27	72.92	10	36.46
Crop	0.55	0.46	0.09	0.84	0.72	-1
Midgut	0.06	0.05	0.01	0	0	-1
Hindgut	0.2	0.08	0.03	0.02	0.03	-1
Malpighian T	4.48	0.5	0.72	0.32	0.55	-1
Fat body	0.17	0.29	0.03	0	0	-1
Salivary glan	1.73	0.37	0.28	0.31	0.03	-1
Heart	0	0	0	0.07	0.06	-1
Trachea	-	-	-	-	-	-
Ovary	-	-	-	0.05	0.01	-1
Virgin Sperm	-	-	-	0.16	0.14	-1
Mated Sperm	-	-	-	0.07	0.12	-1
Testis	0.75	0.43	0.12	-	-	-
Accessory gla	0.04	0.03	0.01	-	-	-
Carcass	5.33	0.38	0.86	1.74	1.05	-1
Rectal pad	0.18	0.03	0.03	0.17	0.17	-1

Male v. Female		Larval		
M/F	p value	FPKM	SD	Enrichment
3.1	$p > 0.05$	0.32	0.21	-1
1.86	$p > 0.01$	-	-	-
1.3	n.s.	-	-	-
1.56	$p > 0.01$	9.97	5.8	4.98
1.55	n.s.	-	-	-
1	n.s.	-	-	-
1	n.s.	0	0	-1
1	$p > 0.05$	0	0	-1
2.2	$p > 0.01$	0	0	-1
1	n.s.	0.04	0.07	-1
1	n.s.	0	0	-1
1	n.s.	-	-	-
-	-	0.06	0.11	-1
-	-	-	-	-
-	-	-	-	-
-	-	-	-	-
-	-	-	-	-
-	-	-	-	-
2.7	$p > 0.05$	1.06	0.54	-1
1	n.s.	-	-	-

FlyBase ID **FBgn0035293**
Annotation S **CG5687**
Symbol **CG5687**

	Adult Male			Adult Female		
Tissue	FPKM	SD	Enrichment	FPKM	SD	Enrichment
Whole body	13.2	1.04	1	5.32	0.26	1
Head	19.17	3.18	1.45	19.71	4.28	3.7
Eye	20.34	3.31	1.54	18.58	1.8	3.49
Brain / CNS	18.73	0.46	1.42	20.82	2.24	3.91
Thoracicoabc	49.7	5.73	3.77	43.84	0.52	8.24
Crop	1.38	0.62	0.1	0.97	0.09	0.18
Midgut	0.06	0.07	0	0	0	0
Hindgut	0.34	0.15	0.03	0.86	0.45	0.16
Malpighian T	0.17	0.12	0.01	0.06	0.02	0.01
Fat body	1.07	0.17	0.08	0.52	0.31	0.1
Salivary glan	2.62	0.15	0.2	0.86	0.35	0.16
Heart	1.7	0.16	0.13	1.17	0.59	0.22
Trachea	-	-	-	-	-	-
Ovary	-	-	-	0.16	0.13	0.03
Virgin Sperm	-	-	-	6.38	3.21	1.2
Mated Spern	-	-	-	19.86	4.92	3.73
Testis	5.31	0.56	0.4	-	-	-
Accessory gla	17.78	2.26	1.35	-	-	-
Carcass	8.5	1.46	0.64	8.79	1.04	1.65
Rectal pad	3.83	0.25	0.29	3.31	0.16	0.62

Male v. Female		Larval		
M/F	p value	FPKM	SD	Enrichment
2.5	p > 0.01	1.15	0.24	-1
0.97	n.s.	-	-	-
1.09	n.s.	-	-	-
0.9	n.s.	6.59	1.35	3.3
1.13	n.s.	-	-	-
1	n.s.	-	-	-
1	n.s.	0.01	0.02	-1
1	n.s.	0.11	0.05	-1
1	n.s.	0.02	0.02	-1
1	n.s.	0.24	0.11	-1
1.31	n.s.	0.06	0.03	-1
1	n.s.	-	-	-
-	-	0.47	0.25	-1
-	-	-	-	-
-	-	-	-	-
-	-	-	-	-
-	-	-	-	-
-	-	-	-	-
0.97	n.s.	5.58	1.02	2.79
1.16	n.s.	-	-	-

FlyBase ID **FBgn0037895**
Annotation S **CG6723**
Symbol **CG6723**

	Adult Male			Adult Female		
Tissue	FPKM	SD	Enrichment	FPKM	SD	Enrichment
Whole body	5.72	0.93	1	1.84	0.46	-1
Head	14.14	1.5	2.47	11.96	1.46	5.98
Eye	13.41	0.23	2.34	11.02	0.56	5.51
Brain / CNS	28.35	1.23	4.96	26.55	2.45	13.28
Thoracicoabc	60.55	8.69	10.59	53.55	1.8	26.78
Crop	0.76	0.76	0.13	0.43	0.3	-1
Midgut	0	0	0	0	0	-1
Hindgut	0.01	0.01	0	0.01	0.02	-1
Malpighian T	0	0	0	0.02	0.03	-1
Fat body	0.22	0.25	0.04	0.16	0.17	-1
Salivary glan	2.16	0.13	0.38	0.8	0.04	-1
Heart	1.96	0.62	0.34	1.16	0.05	-1
Trachea	-	-	-	-	-	-
Ovary	-	-	-	0	0	-1
Virgin Sperm	-	-	-	0.05	0.08	-1
Mated Spern	-	-	-	0	0	-1
Testis	0.02	0.03	0	-	-	-
Accessory gla	0.03	0.03	0.01	-	-	-
Carcass	6.7	0.38	1.17	6.15	1.87	3.08
Rectal pad	0.04	0.04	0.01	0.04	0	-1

Male v. Female		Larval		
M/F	p value	FPKM	SD	Enrichment
2.9	p > 0.01	0.49	0.2	-1
1.18	n.s.	-	-	-
1.22	n.s.	-	-	-
1.07	n.s.	8.17	2.62	4.08
1.13	n.s.	-	-	-
1	n.s.	-	-	-
1	n.s.	0	0	-1
1	n.s.	0	0	-1
1	n.s.	0	0	-1
1	n.s.	0	0	-1
1.08	n.s.	0.01	0.02	-1
1	n.s.	-	-	-
-	-	0.02	0.03	-1
-	-	-	-	-
-	-	-	-	-
-	-	-	-	-
-	-	-	-	-
-	-	-	-	-
1.09	n.s.	0.42	0.2	-1
1	n.s.	-	-	-

FlyBase ID **FBgn0037238**
Annotation S **CG1090**
Symbol **CG1090**

	Adult Male			Adult Female		
Tissue	FPKM	SD	Enrichment	FPKM	SD	Enrichment
Whole body	9.64	0.37	1	2.12	0.13	1
Head	35.13	1.45	3.64	32.19	5.13	15.18
Eye	54.05	7.39	5.61	37.58	3.28	17.73
Brain / CNS	64.39	2.88	6.68	57.78	10.99	27.25
Thoracicoabc	67.39	9.44	6.99	75.25	15.69	35.5
Crop	0.71	0.4	0.07	0.79	0.46	0.37
Midgut	1.03	0.56	0.11	0.3	0.16	0.14
Hindgut	0.05	0.03	0.01	0.04	0.01	0.02
Malpighian T	0.07	0.04	0.01	0.07	0.09	0.03
Fat body	0.33	0.57	0.03	0.1	0.11	0.05
Salivary glan	1.92	0.3	0.2	0.82	0.36	0.39
Heart	0.45	0.14	0.05	0.21	0.08	0.1
Trachea	-	-	-	-	-	-
Ovary	-	-	-	0.07	0.05	0.03
Virgin Sperm	-	-	-	0.26	0.02	0.12
Mated Sperm	-	-	-	0.3	0.18	0.14
Testis	0.53	0.14	0.05	-	-	-
Accessory gla	0.03	0.03	0	-	-	-
Carcass	3.83	1.46	0.4	2.61	1.5	1.23
Rectal pad	0.12	0.07	0.01	0.31	0.22	0.15

Male v. Female		Larval		
M/F	p value	FPKM	SD	Enrichment
4.5	p > 0.01	0.38	0.06	-1
1.09	n.s.	-	-	-
1.44	n.s.	-	-	-
1.11	n.s.	7.76	1.36	3.88
0.9	n.s.	-	-	-
1	n.s.	-	-	-
1	n.s.	0	0	-1
1	n.s.	0.03	0.02	-1
1	n.s.	0.01	0.02	-1
1	n.s.	0.32	0.22	-1
1	n.s.	0.03	0.01	-1
1	n.s.	-	-	-
-	-	0.09	0.02	-1
-	-	-	-	-
-	-	-	-	-
-	-	-	-	-
-	-	-	-	-
-	-	-	-	-
1.47	n.s.	0.7	0.09	-1
1	n.s.	-	-	-
